# New alignment method for remote protein sequences by the direct use of pairwise sequence correlations and substitutions

**DOI:** 10.3389/fbinf.2023.1227193

**Published:** 2023-10-12

**Authors:** Kejue Jia, Mesih Kilinc, Robert L. Jernigan

**Affiliations:** ^1^ Roy J. Carver Department of Biochemistry, Biophysics, and Molecular Biology, Iowa State University, Ames, IA, United States; ^2^ Bioinformatics and Computational Biology Program, Iowa State University, Ames, IA, United States

**Keywords:** protein sequences, sequence alignment algorithm, coevolution information, disordered proteins, function from sequence alignment

## Abstract

Understanding protein sequences and how they relate to the functions of proteins is extremely important. One of the most basic operations in bioinformatics is sequence alignment and usually the first things learned from these are which positions are the most conserved and often these are critical parts of the structure, such as enzyme active site residues. In addition, the contact pairs in a protein usually correspond closely to the correlations between residue positions in the multiple sequence alignment, and these usually change in a systematic and coordinated way, if one position changes then the other member of the pair also changes to compensate. In the present work, these correlated pairs are taken as anchor points for a new type of sequence alignment. The main advantage of the method here is its combining the remote homolog detection from our method PROST with pairwise sequence substitutions in the rigorous method from Kleinjung et al. We show a few examples of some resulting sequence alignments, and how they can lead to improvements in alignments for function, even for a disordered protein.

## Introduction

Protein sequence alignments are commonly used to identify the similarities and differences between proteins, crucial procedures in bioinformatics analyses. Alignments are vital for understanding protein function, evolution, and the various relationships among mutations. The amino acid substitution matrix used in a protein sequence alignment is the central component that affects the quality of any resulting alignment. The usual substitution matrix is a matrix with each element representing the propensity of one amino acid type to change to another specific type of amino acid. The early PAM (Point Accepted Mutation) substitution matrices were introduced by Margaret Dayhoff and colleagues in the early 1970s (Dayhoff, 1972). PAM matrices are based on the observation that the conservation of a particular amino acid decreases with the evolutionary distance between sequences. The most commonly used BLOSUM (BLOcks SUbstitution Matrix) family of amino acid substitution matrices was developed by Steven and Jorja Henikoff in the late 1980s (Henikoff and Henikoff, 1992). These matrices are based on short, non-gapped sequence alignment “blocks” that are relatively well-conserved across evolutionary distances. The BLOSUM matrices are named according to the percentage of identity among the sequences used to generate the matrix. For example, the BLOSUM62 matrix was derived from a set of protein sequences that are at least 62% identical to each other. The BLOSUM62 matrix has become the most commonly used matrix in many sequence alignment-based tools, such as BLAST (Altschul et al., 1990), Clustal Omega (Sievers and Higgins, 2014), MAFFT (Katoh et al., 2002), and MUSCLE (Edgar, 2004). Later, more advanced matrices were developed for universal application (Kann et al., 2000; Prlic et al., 2000; Muller et al., 2002; Crooks and Brenner, 2005; Lemaitre et al., 2011; Yamada and Tomii, 2014; Leelananda et al., 2016; Keul et al., 2017). More specific substitution matrices were developed for different families of proteins or different types of structures (Yu et al., 2003; Vilim et al., 2004; Edgar, 2009; Song et al., 2015; Trivedi and Nagarajaram, 2019).

In contrast to substitution matrix-based tools, many protein sequence alignment tools, including HMMER (Johnson et al., 2010), SAM (Karplus et al., 1998), HHpred (Soding et al., 2005), and HHblits (Remmert et al., 2011) are based on Hidden Markov Models (HMMs). HMM approaches employ a statistical model that is trained on a group of protein sequences to develop a “profile” for identifying sequence motifs. The HMM profile contains information about the frequency of each amino acid at each position in the reference protein sequences. This data is utilized to produce a scoring system that reflects the probability of observing a specific amino acid at a particular position in the query sequence. However, it requires the use of a set of seed sequences to create the profile.

The relationship between protein sequences is unambiguous when the pairwise sequence identity is high (>40%). However, as the identities become lower in the range of 20%–35%, commonly referred to as the twilight zone protein sequence (Rost, 1999), the alignments and relationships become less certain. In this range named ProtSub, the similarity between proteins is more challenging to ascertain, and the boundaries between similar and non-similar structures are blurry (Rost, 1999; Weisman et al., 2020).

Coevolution methods effectively identify evolutionary correlations between residue positions that exhibit dependent sequence variations. However, this information is usually ignored by sequence alignment methods. Many successful applications of coevolution methods have been developed. EVcouplings (Marks et al., 2012; Hopf et al., 2019) and others (Dunn et al., 2008; Morcos et al., 2011; Jones et al., 2012; Ovchinnikov et al., 2014; Seemayer et al., 2014; Ovchinnikov et al., 2017) have been successful in determining 3D structures by predicting intramolecular residue contacts based on residue pairs that are correlated. Based on the principle of interacting residues are often coevolving, protein-protein interactions can also be inferred from intermolecular coevolved protein residues (Cong et al., 2019; Green et al., 2021). EVmutate utilizes a probabilistic graphical model to infer the effects of mutations at given positions (Hopf et al., 2017). Recently, we have integrated coevolutionary dependence information into a substitution matrix, which generates improved sequence alignments consistent with structure alignments for twilight zone protein sequences (Jia and Jernigan, 2021). The ProtSub matrix allows more substitutions, as are observed in the correlated pairs. The results are more compact alignments with fewer gaps/insertions. Using the correlated pair information, we have developed a double-point amino acid substitution matrix named ProtSub400 (PS400), consisting of 400 × 400 elements (See Methods section for more information). These newly introduced substitution matrices incorporate a certain degree of structural information.

Previous protein sequence alignment tools have the significant limitation that they depend, in the substitution matrix, on similarities among single amino acid types and do not account for any information from protein structures. To overcome this limitation, people have begun to utilize protein structures to generate sequence alignments. Recent advances in deep learning-based protein structure prediction have increased the number of available protein structures significantly, with these structure predictions reaching near-experimental quality. The Protein Data Bank (Bittrich et al., 2021) includes ∼1 million Computed Structure Models (CSMs) from AlphaFold (Jumper et al., 2021) and RoseTTAFold (Baek et al., 2021). The European Bioinformatics Institute has deposited over 214 million predicted structures, while the ESMAtlas database contains over 617 million metagenomic structures predicted using ESMFold (Lin et al., 2023). Structure-based homolog detection tools such as FoldSeek (van Kempen et al., 2022) are also being used to obtain more accurate protein homolog matches. As a result, structure alignments are now being used to improve sequence alignments. However, structure-based alignments do have some limitations. They do not usually account for the dynamic nature of structures, which can undergo conformational changes as they function. In addition, sequence variations can create conformational differences for proteins within the same family. Structure alignment algorithms for sequence alignments depend on the 3D coordinates of the amino acids aligning closely. Large domain motions are well-known to make structure alignment difficult to perform globally, resulting in some poor alignments. Furthermore, protein structure alignments cannot be applied to disordered proteins since they lack well-defined three-dimensional structures and typically have a dynamic ensemble of conformations. Finally, structure alignment algorithms can be computationally intensive, particularly for large proteins.

In this study, we present a novel approach named PROSTAlign to accurately align homologous proteins, especially for proteins with low sequence identities and structural differences. First, we employ our newly developed homolog search tool, PROST, to identify the homologous proteins (Kilinc et al., 2023) (not aligning them). PROST utilizes a protein embedding distance, which is generated by using a large protein language model, to evaluate accurate homolog relationships, and it outperforms all other traditional homolog search tools. Next, we adopt a new dynamic programming-based algorithm that utilizes a 20 × 20 amino acid substitution matrix and a 400 × 400 substitution matrix to obtain more accurate protein sequence alignments. The 20 × 20 matrix, as a conventional substitution matrix, describes single amino acid substitutions, while the 400 × 400 matrix describes correlated paired substitutions, i.e., a pair of amino acids changing to a different pair. The approach used here was previously introduced by Kleinjung et al. (2004) to utilize a 400 × 400 substitution matrix plus a contact matrix to generate accurate sequence alignments. That contact matrix was extracted from protein structures. However, instead of inferring protein structure information, we use a contextual correlation matrix generated from the pre-trained protein language model, ESM-1b (Rives et al., 2021). The advantage of using this correlation matrix is that it contains not only residue proximity information but also additional contextual dependencies between amino acid positions such as long-range allosteric effects. The ESM-1b architecture contains 34 layers, each containing a self-attention module. The self-attention module is responsible for computing context vectors that capture dependencies between different parts of the input sequence. These context vectors are then transformed into a final position-wise correlation map. This map, similar to coevolution correlations, reflects not only residue proximity information but also other contextual dependencies. Our results show that this new approach achieves better congruence between sequence alignments and structure alignments for twilight-zone pairs of protein sequences. Additionally, it can generate correct sequence alignments for homologous proteins having different conformations since it uses only sequences. Moreover, this approach has the ability to align disordered proteins correctly based on their functional domains.

## Results and discussion

### Congruence between sequence alignment and structure alignment

Proteins with the same functions generally have similar folds, which implies that structural alignment data can be used to evaluate the quality of protein sequence alignments, especially for alignments of twilight zone protein sequence pairs. Our previous work introduced a novel 20 × 20 substitution matrix (ProtSub) that incorporated coevolution information and produced sequence alignments that agree better with structure alignments for twilight zone protein sequence pairs. In this study, we improve our alignments.

Further by incorporating the protein language model correlation map and using the pair-to-pair 400 × 400 substitution matrix for those pairs. We select a set of 2,002 non-redundant protein pairs from the CATH S20 database (Sillitoe et al., 2021) having different fold characteristics. Each protein pair belongs to the same homologous family, with a sequence identity of around 20%, and the two have nearly identical structures. In the PROSTAlign procedure, we evaluate two types of double-point amino acid matrices (400 × 400). PS400 represents the log-odds ratio-based score obtained from strongly coevolved residue positions (See Methods section for more information). The second matrix, CAO120, is derived from a Markov model of protein side-chain contact evolution (Kleinjung et al., 2004). To make a comparison, we collect a set of classical and newly developed amino acid substitution matrices to align the test protein pairs. The alignment process is performed using the Needleman-Wunsch algorithm, as implemented in the EMBOSS software (Rice et al., 2000). To obtain a comprehensive evaluation, we iterate through a wide range of gap penalties, including gap opening and gap extension. For each pair of proteins, we calculate the RMSD between aligned sequence segments from their structures. The value of RMSD is normalized by the number of aligned residues. As shown in Table 1, PROSTAlign yields lower RMSD values than the others (highlighted in bold in Table 1), which demonstrates the gains in the agreements between sequence and structure alignments. The results show strong similarities from the use of either the PS400 or the CAO120 matrix.

**TABLE 1:** Comparison of normalized root mean squared deviations (RMSD) for 2,002 non-redundant protein pairs from the CATH S20 database (Sillitoe et al., 2021), based on sequence alignments with different substitution matrices. Bold numbers are the best cases for each column.

	Average RMSD (gap opening, gap extension)					
Substitution matrix	4, 0	4, 2	8, 0	8, 2	12, 0	12, 2
BLOSUM45 Henikoff and Henikoff, (1992)	0.276	0.220	0.276	0.268	0.299	0.478
BLOSUM62 Henikoff and Henikoff, (1992)	0.286	0.273	0.341	0.828	0.433	2.612
Crooks Crooks and Brenner, (2005)	0.310	0.272	0.370	1.561	0.476	4.208
EPAM120 Dayhoff, (1972)	0.300	0.295	0.384	2.637	0.566	5.467
EPAM250 Dayhoff, (1972)	0.248	0.221	0.281	0.311	0.293	0.451
MIQS Yamada and Tomii, (2014)	0.240	0.210	0.266	0.280	0.285	0.394
moll60 Lemaitre et al., (2011)	0.305	0.292	0.383	1.446	0.462	3.879
Optima Kann et al., (2000)	0.268	0.234	0.303	0.353	0.332	0.965
PFASUM100 Keul et al., (2017)	0.288	0.274	0.342	0.992	0.440	3.017
PFASUM50 Keul et al., (2017)	0.267	0.214	0.267	0.267	0.314	0.463
Prlic Prlic et al., (2000)	0.257	0.213	0.251	0.213	0.250	0.235
ProtSub Jia and Jernigan, (2021)	0.236	0.203	0.245	0.215	0.263	0.314
VTML250 Muller et al., (2002)	0.275	0.232	0.281	0.333	0.311	0.511
PROSTAlign (CAO120)	0.162	**0.143**	**0.151**	0.142	0.148	0.142
PROSTAlign (PS400)	**0.161**	0.145	0.152	**0.140**	**0.147**	**0.140**

### Aligning remote protein homologs with different conformations

The use of structure alignment as a metric for assessing sequence alignment precision can lead to erroneous conclusions, primarily because of conformational changes. Proteins are not static but have important dynamics that are a critical aspect of their functional mechanisms such as catalysis, regulation, and signaling. Furthermore, conformational changes can also arise from sequence variations among homologous proteins within the same family, leading to differences in their conformations. Protein structure alignments rely on the 3D coordinates of atoms to align protein structures. Anishchenko *et al.* Anishchenko et al. (2017) have pointed out that conformational changes can result in false predictions of residue contacts.

We demonstrate that our approach is able to correctly align sequences of two homolog proteins with conformational differences. In the SwissProt database (Bairoch and Apweiler, 2000), the structures of the 54S ribosomal protein L24 from baker’s yeast (UniProt ID: P36525) and fission yeast (UniProt ID: O60091) are AlphaFold2 predictions.

**FIGURE 1:**
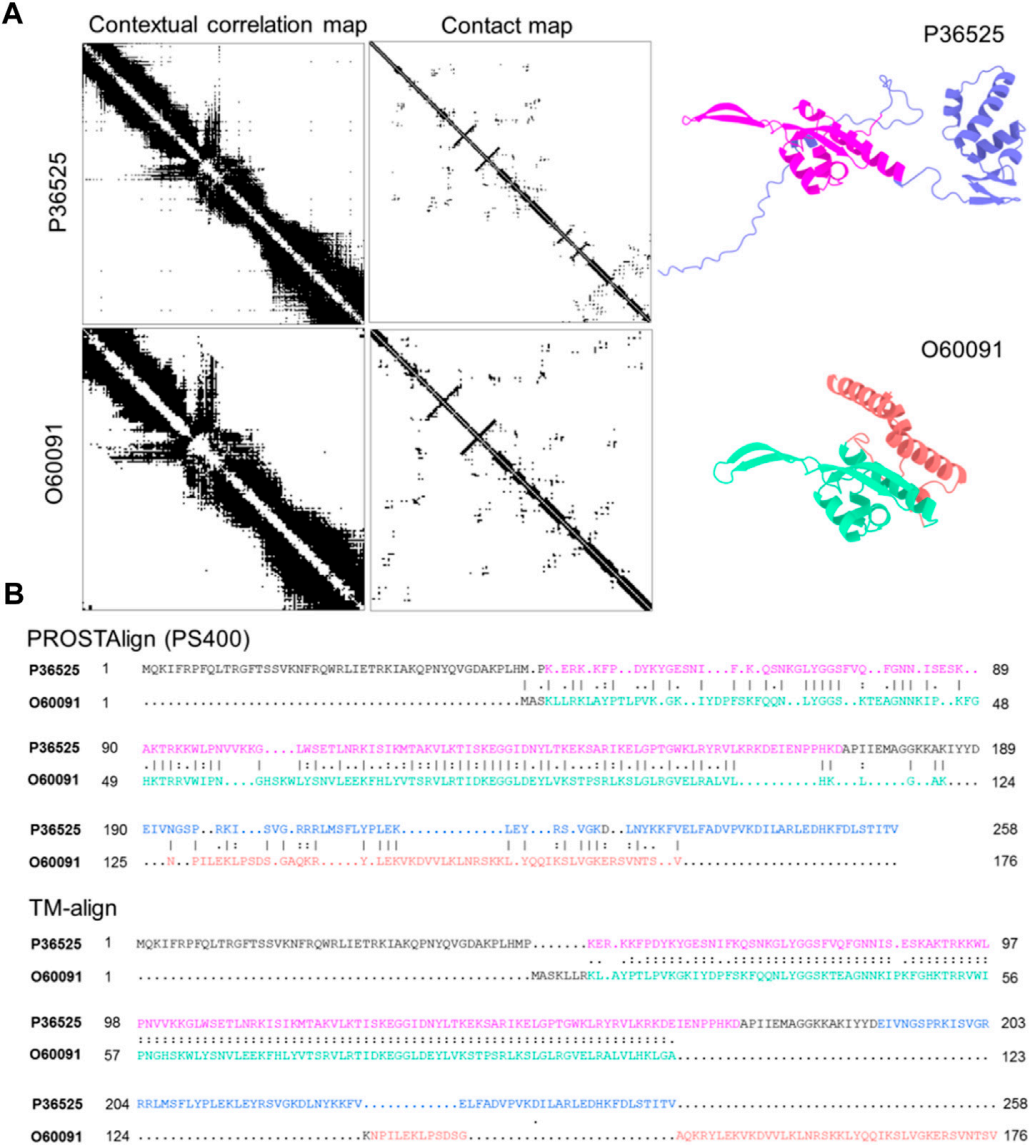
Two homologous protein structures of the 54S ribosomal protein L24 with conformational differences align better with PROSTAlign. **(A)** we show that the contact maps for two structures have some significant differences, but their contextual correlation maps are similar. On the right, two protein structures are colored according to their structural domains. The two parts can be aligned using TM-align are highlighted in green and magenta. Other domains cannot be aligned structurally due to conformational changes are in blue and red-brown. **(B)** shows the sequence alignment generated by PROSTAlign. Our method correctly aligns the two sequences. The sequence alignment generated based on the structure alignment is shown at the bottom, which fails to align the conformational differences (highlighted in blue and red).

Although these two structures have nearly identical secondary structure arrangements in sequential order, the TM-score (Zhang and Skolnick, 2005) of the two structures is 0.39, which would indicate the two proteins have different folds. There is one domain that can be superimposed together, while the second domain cannot. Their sequence identity is 30.2%. We use FATCAT (Li et al., 2020), a structure matching tool that takes into account the protein dynamics, to measure structural similarity and the result indicates that both structures share significant similarities, with a *p*-value of 1.89e-05. As shown in Figure 1. A (right), the two structures exhibit distinct conformations, with the part of the structures (highlighted in green and magenta) that superimposes well. The sequence alignment generated by PROSTAlign, as shown in Panel B (top), correctly aligns the sequences of the superimposed parts well. In addition, the sequence alignment aligns the second domains better as well for the helix domains showing in red-brown and blue separately on each structure. The sequence alignment based on a structure alignment generated using TM-align is shown in Panel B (bottom), containing long mismatched segments where there are structural differences and is also a less compact alignment.

In Panel A (left), the residue contact maps of the two proteins are clearly different due to the conformational differences. These differences explain why the structure alignment does not provide the correct sequence alignment. However, the contextual correlation maps generated from ESM-1b are somewhat more similar, providing pairwise information that helps overcome the problem of conformational changes and generate a better sequence alignment. Contextual dependence is a key aspect of the protein language models that captures the global relationships between different parts of a protein sequence. Therefore, aligning two proteins by using this map is analogous to aligning two networks described by position correlations, which we use here to an improved sequence alignment.

### Aligning intrinsically disordered proteins

Intrinsic disorder refers to the lack of any single stable, ordered structure in a protein or a region of a protein. Proteins that contain a significant extent of disordered regions are referred to as intrinsically disordered proteins (IDPs). IDPs do not adopt any single, well-defined 3D structure but instead usually are represented as ensembles of rapidly interconverting conformations that are highly flexible and dynamic. Despite being unstructured, IDPs play important roles in many cellular processes, including signaling, transcriptional regulation, and molecular recognition. In addition, IDPs are also involved in a number of diseases, including cancer and neurodegenerative disorders. Uversky (2013) estimated that 30%–40% of eukaryotic proteins contain significant disordered regions based on various computational and experimental approaches (Uversky, 2013). The DisProt database (Quaglia et al., 2022) collects experimentally characterized disordered proteins and protein regions.

The distribution of conformations of an IDP can be different in different contexts or under different conditions, which makes it difficult to establish a consistent alignment across multiple structures. In well-folded proteins, the residue contact map, as predicted from the sequence correlations, can provide useful information about the protein’s 3D single dominant structure and function. However, since IDPs exist as an ensemble of alternative conformations that are highly flexible and dynamic, the contact map of a disordered protein can vary widely depending on the specific conformation(s) that are present in the ensemble for a specific condition. Therefore, using the correlation map from the protein language model is more reliable for IDPs.

Aligning the sequences of intrinsically disordered proteins is challenging due to their lack of a well-defined 3D structures, since structure usually imposes strong constraints on sequence. While computational methods such as generating specialized substitution matrices for IDPs have shown promise in predicting and aligning disordered protein functional domains, the accuracy of these methods is still limited by the complexity and dynamic nature of disordered proteins (Trivedi and Nagarajaram, 2019). Also, the availability of predicted structures cannot help align IDP sequences because structure alignment algorithms are designed to align protein sequences based on their 3D structure similarities. IDPs lack a stable, well-defined structure, so it is not possible to use structure alignments to generate a reliable sequence alignment. Despite these problems there can be advantages from achieving the proper alignment of disordered protein sequences as we show in the following example that demonstrates that our new alignment procedure can provide useful sequence alignments even for disordered proteins.

**FIGURE 2:**
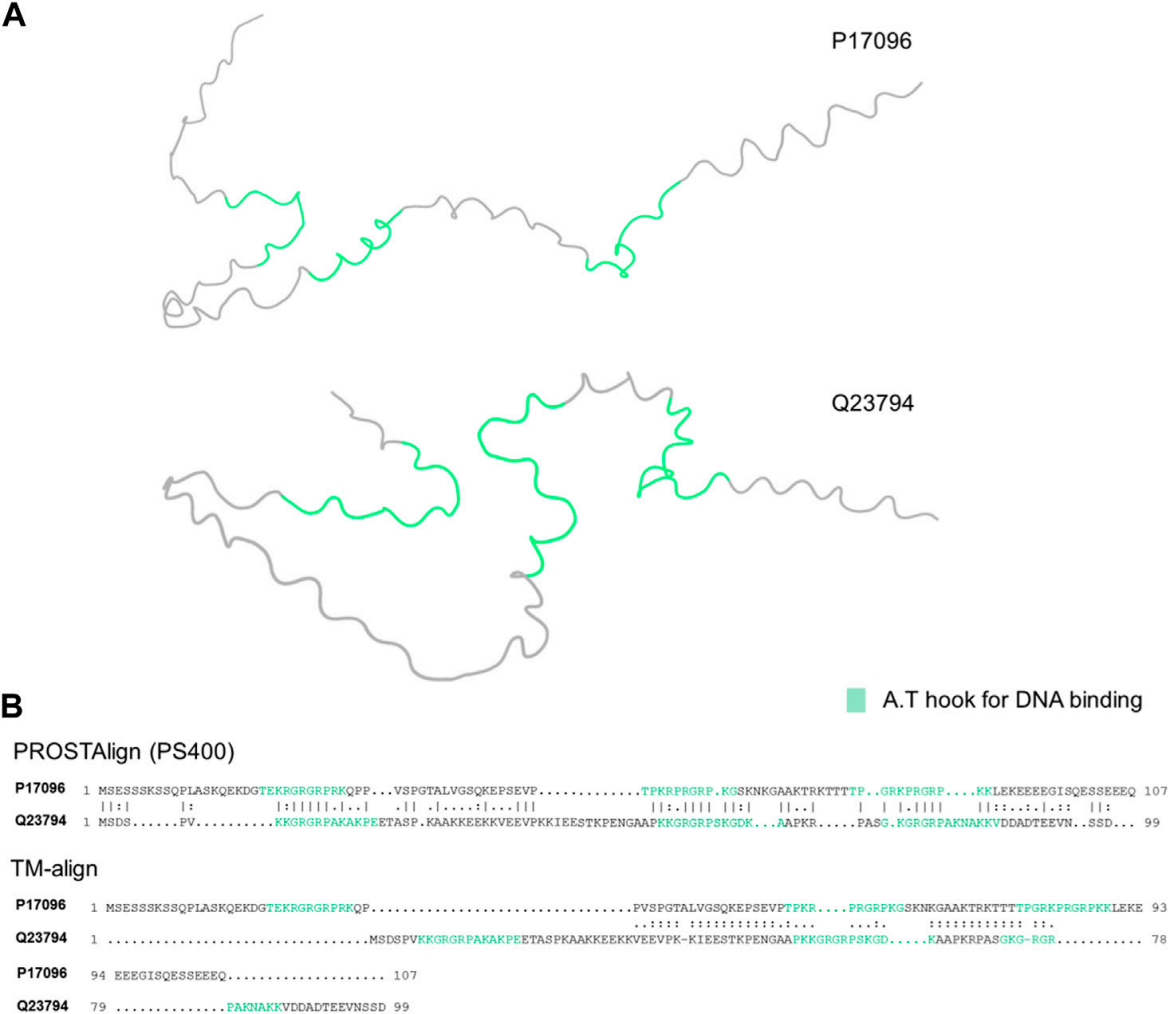
Comparisons of the human (P17096) and *Chironomus tentans* (Q23794) High Mobility Proteins. **(A)** Visualization of the two protein structures with A.T hook parts highlighted in green. **(B)** The sequence alignments at the top is generated by PROSTAlign, showing the three hook parts between the human and *Chironomus tentans* are aligned well. The sequence alignment at the bottom is generated by TM-align with only one A.T hook region partially aligned but other two are not aligned.

Here, with the help of the predicted contextual correlation map and the ps400 substitution matrix, PROSTAlign aligns intrinsically disordered proteins accurately according to their known functional domains. The high mobility group (HMG) proteins are a family of non-histone chromatin-associated proteins that play crucial roles in DNA organization and gene regulation. HMG proteins exhibit disordered regions or domains within their overall structure, which contribute to their ability to interact with a variety of different proteins and nucleic acids. These proteins are highly conserved across species and are found in both eukaryotic and prokaryotic organisms (Reeves and Nissen, 1990). Figure 2 demonstrates PROSTAlign’s capability to align sequences of disordered proteins based on their functional motifs. In this example, we have aligned the sequences of HMG-I from *Chironomus tentans* (UniProt ID: Q23794) and HMG-I/HMG-Y from humans (UniProt ID: P17096), both of which have three DNA binding motifs referred to as A.T hooks. These motifs are named for their ability to specifically bind to AT-rich regions of DNA. The two predicted protein structures we collected from the UniProt database are found to be highly disordered, making structural alignment inaccurate (Figure 2.A). Due to the disordered characteristics, contact maps of disordered proteins contain mostly trivial contacts formed from residues next to each other along the sequence. Sequential contacts do not provide much that is useful in terms of defining the structures. The contextual correlation matrix generated by ESM-1b captures dependent information between residues with long sequence separations, and PROSTAlign utilizes this matrix to generate precise sequence alignments. As shown in Figure 2. B, the sequence alignment generated by PROSTAlign correctly matches the three known A.T hook regions together, whereas the structure alignment approach can only partially matches one of the three regions.

## Conclusion and discussion

With the explosive growth in reliably predicted protein structures, sequence alignments can be improved by referencing structure alignments. However, in our study, we highlight the limitations of structure-based alignments and propose a language model correlation-based alignment procedure. By integrating the contextual correlation map from the protein language model, single-point and double-point substitution matrices are used in the alignment procedure, we can achieve significantly better agreements between sequence alignments and structure alignments. Our proposed procedure overcomes the difficulties caused by conformational changes by methods that rely upon the structural contact map, by instead using the correlations derived from the large protein language model ESM-1b. Moreover, for proteins not having any structural information, such as intrinsically disordered proteins, our procedure can generate alignments that more accurately match known functional domains as shown in the example in Figure 2. This is a powerful tool for accurately aligning protein sequences, particularly for those with conformational variations and those lacking structural information. Conservation is an important consideration for sequences and this study suggests the importance of considering correlated pairs in sequence substitutions as a type of conservation. Strongly correlated positions in a protein sequence often exhibit compensatory mutations, where a mutation at one position is accompanied by a specific mutation at another position, maintaining the protein’s overall properties and functional stability without causing significant changes. Therefore strongly correlated positions can be considered to be an additional type of conservation that is particularly important for structures.

The major strength of our new method, as well as its limitation, is that it generates global alignments. Global alignments are suitable for comparing sequences of complete proteins but cannot provide local alignments that capture the similarities between subdomains of input sequences. The reason is that ESM1b, the model upon which our method is based, was trained using a large set of complete protein sequences rather than functional or structural subdomains. Therefore, the contextual information captured by our model pertains to the complete protein sequence. The contextual information may not be accurate when using partial sequences, such as specific functional or structural domains and may lead to unreliable alignments in those cases. To address these limitations, we are developing a new version of PROST to provide contextual information for subdomains, which later can be used by PROSTAlign to generate local alignments.

## Methods

### Main workflow

**FIGURE 3:**
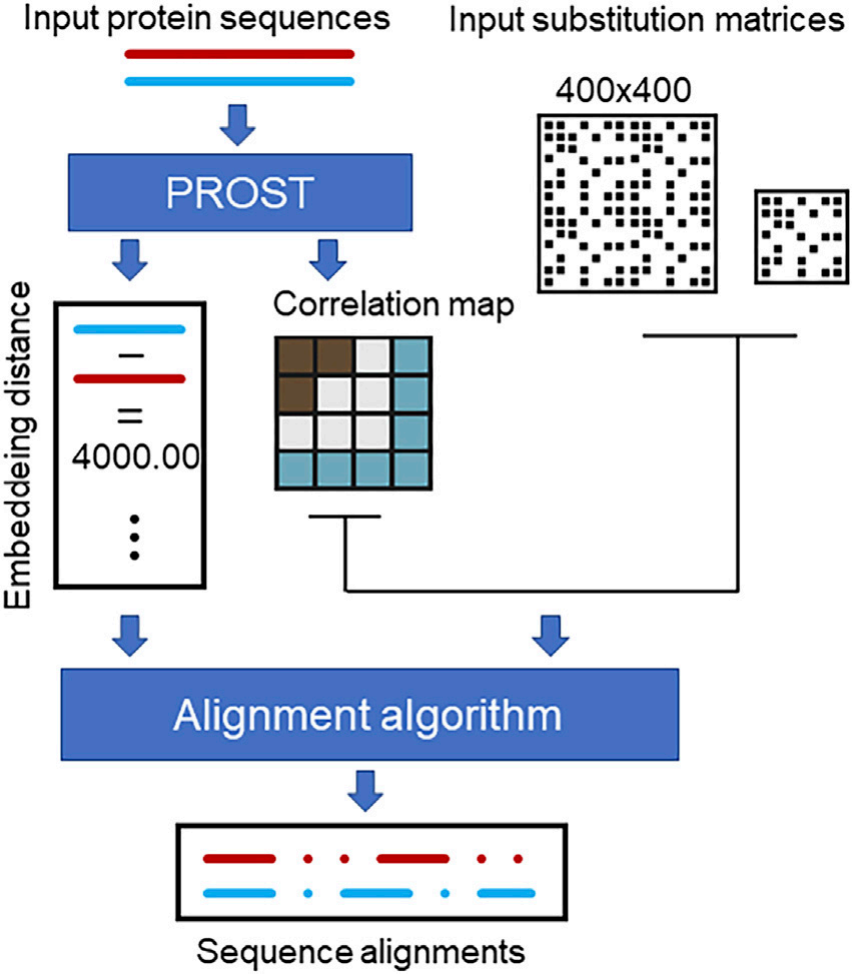
The workflow of PROSTAlign. Two input protein sequences are used with PROST to develop their homolog relationship and to generate a contextual correlation map based on one of the sequences. Subsequently, the alignment algorithm employs the correlation map, a 400 × 400 pairwise substitution matrix, and a 20 × 20 conventional substitution matrix to produce a global alignment for the two input sequences.

A schematic fur the sequence alignment method is depicted in Figure 3. The input to the PROSTAlign tool is a pair(s) of protein sequences to be aligned. The first step is to calculate the embedding distance to determine the input relationship. In the second step, a contextual correlation matrix is generated based on either one of the input sequences. If the proteins are closely related, a smaller cutoff is applied to generate the correlation matrix and uses a larger weight for the 400 × 400 pair substitution matrix. However, if the proteins are not close homologs, the 400 × 400 matrix will have a smaller weight, and the 20 × 20 substitution matrix will be the primary matrix used to generate the alignments. This is because if two proteins are homologs, they share a similar correlation matrix, which is protein-family specific. This correlation matrix can be used as a generalization of the contact map (Jia et al., 2023) to enhance the alignment. In cases where the proteins are more distantly related, we put more weight on the conventional 20 × 20 substitution matrix (ProtSub), which is derived from a large set of protein families. The final sequence alignment is then generated by the algorithm introduced by Kleinjung et al. (2004). Instead of using a contact map, PROSTAlign uses a correlation map as an input to the algorithm. In the aligning process, each substitution score is a result of combining scores from single-point mutations and double-point mutations. The single-point mutation score is evaluated using a 20 × 20 amino acid substitution matrix ( $S_{20\times 20}$ ), while the double-point mutation score is evaluated using a 400 × 400 pairwise amino acid substitution matrix ( $\left.S_{400\times 400}\right)$ . Four parameters significantly influence the final score: a weight for single-point mutations ( $w_{1}$ ), set to 1.0 by default, the relative weight for the double-point mutations ( $w_{2}$ ), ranging from 0.0 to 0.9 and is set to 0.1 by default, the gap-opening penalty ( p ), and the gap extension penalty ( q ). Thus, at each step, a pair of mutation scores are calculated as following:

$$S(i,k)=w_{1}S_{20\times 20}(i,k)+w_{2}S_{400\times 400}(i,j,k,l)$$

$$S(j,l)=w_{1}S_{20\times 20}(j,l)+w_{2}S_{400\times 400}(i,j,k,l)$$

Here the i,j,k,l are amino acid types.

### PROST tool and contextual correlation matrix

PROST is a fast homolog search tool based on a pretrained optimized deep learning model described in Reference (Kilinc et al., 2023). It compares protein sequence embeddings by taking vector differences to evaluate the relationship between a pair of proteins. For a protein with N residues, the ESM1b embedding results in a matrix with dimensions of 34 × N × 1,280, where 1,280 represents the embedding length of a residue and 34 is the number of output layers from the language model. Our optimization study found that the combination of layers 26 and 14 yielded the highest accuracies for homolog detection. To achieve the smallest memory footprint while retaining the accuracy of homolog detection, we applied a 2-dimensional inverse discrete cosine transform (2D-iDCT) to reduce the embedding matrix of layer 26 to 5 × 44 and layer 14 to 3 × 85. To evaluate the embedding distance, we take the sum of absolute differences between each element in the two sets of representation matrices. As a result, PROST is faster and more accurate than traditional sequence matching tools in identifying putative remote homologs for proteins with relatively low sequence identities. The main advantage of PROST is its high efficiency in identifying remote homologs, while not using any sequence or structure alignments. Its compact representation of protein sequences makes the searching procedure computationally efficient. First, PROST can be used to determine their similarities by evaluating their embedding distance. Then our procedure generates a pairwise contextual correlation matrix using ESM-1b (Rives et al., 2021) for the first input sequence. The correlation matrix describes the contextual dependence information between each pair of amino acid positions for the input sequence and is later used in the sequence alignment procedure.

### The CAO contact matrix

The CAO matrix was introduced previously by Kleinjung et al. (2004). It is a 400 × 400 amino acid scoring matrix used in protein sequence alignments and is based on a Markov model of protein side-chain contact evolution. It provides scores for evolutionary transitions (mutations) between possible combinations of residue contacts in a matrix with 400 × 400 elements, with each cell containing a score for the transition from a contact pair of amino acids to another sequence pair for that contact. CAO scores are intermediate between sequence-based PAM scores and structure-based Root Mean Square Deviation (RMSD) values, and can be used to score alignments of template and query sequences by summing up the CAO substitution matrix values of all contacts.

### The contact-based alignment algorithm

Kleinjung *et al.* Seemayer et al. (2014) developed an alignment algorithm that incorporates the CAO contact scores (a 400 × 400 matrix) into a dynamic programming (DP) procedure. The algorithm uses a sliding window approach to probe for potential positions of a contact in the query sequence by testing all possible contacts, assuming that the query residues are in contact. CAO scores are used to assign positive or negative scores to each hypothetical contact, or correlation. The optimal alignment is found by forward score addition and back-tracing. The algorithm has routines for local and global alignments and is complemented with PAM-type substitution matrix scores to compensate for potentially missing contact information in the template.

### ProtSub matrix

The ProtSub matrix (Jia and Jernigan, 2021) is an amino acid substitution matrix that effectively incorporates interdependent amino acid substitutions and includes structural information. The construction of this matrix involves three main steps: First, we calculated the evolutionary correlation between position pairs in a multiple sequence alignment (MSA) for a given protein family. In (Jia and Jernigan, 2021), mutual information is used for evaluating the correlation information. Second, pairs are filtered to only include those with significant correlations and that are spatially proximate in the corresponding protein structure. Finally, a 20 by 20 amino acid substitution matrix is derived as log-odds ratios based on the interdependent amino acid substitutions from the selected pairs. The resulting matrix permits more substitutions than BLOSUM62.

### ProtSub400 matrix (PS400)

The double-point substitution matrix is calculated by using highly correlated substitutions extracted from 5,050 Pfam (Mistry et al., 2021) multiple sequence alignments. There are 400 × 400 elements in the matrix. The coevolution correlation is measured using Direct Coupling Analysis (DCA) (Marks et al., 2012). In contrast to the marginal probability based correlation method, mutual information, DCA detects the direct coevolution signals from multiple sequence alignments. The transitive effects are removed in its global statistical model, leading to a better prediction of direct residue contacts. For each protein family, the 15% top-ranked position pairs from the multiple sequence alignment are selected for the pairwise substitution frequencies. The elements of the matrix are calculated as log-odds ratios, where the foreground probability (alternative hypothesis) is evaluated using the frequency of substitutions of highly correlated amino acid pairs and the background probability (null hypothesis) is evaluated by counting the joint frequency of two pairs of amino acids within the dataset.

## Data Availability

The original contributions presented in the study are included in the article/Supplementary Material, further inquiries can be directed to the corresponding author.

## Supplementary material

Supplementary Files Please find “cao120.txt” in “1227193__Table 3.DOCX” in the Supplementary Files. Please find “ps400.txt” in “1227193__Table 4.DOCX” in the Supplementary Files.
