# Supplementary material for: New alignment method for remote protein sequences by the direct use of pairwise sequence correlations and substitutions
Source: Front Bioinform. 2023 Oct 12;3:1227193. doi: 10.3389/fbinf.2023.1227193 (PMC10602800; doi:10.3389/fbinf.2023.1227193)
Supplement: Supplementary file 1 [file DataSheet3.docx]

AAAA 9.79016254

AAAC 6.97116731

AAAD 5.93417417

AAAE 6.26171667

AAAF 5.41958689

AAAG 6.67411349

AAAH 5.69104367

AAAI 6.06988657

AAAK 6.04952156

AAAL 5.94424132

AAAM 6.06898511

AAAN 5.83795099

AAAP 6.15811742

AAAQ 6.12890081

AAAR 5.98683420

AAAS 7.01886809

AAAT 6.53448807

AAAV 6.62780828

AAAW 5.07983881

AAAY 5.40201196

AACA 6.90403691

AACC 2.96546567

AACD 4.30289031

AACE 4.70857561

AACF 4.02991366

AACG 4.80184431

AACH 3.65568257

AACI 4.74785863

AACK 4.54299171

AACL 4.61725137

AACM 4.76349171

AACN 4.29629492

AACP 4.16648200

AACQ 4.58748428

AACR 4.41994591

AACS 5.36657387

AACT 5.04300688

AACV 5.18225893

AACW 3.39221233

AACY 4.02512765

AADA 5.94671264

AADC 4.36917432

AADD 4.30199027

AADE 4.58570808

AADF 3.58206873

AADG 4.26608869

AADH 3.92674708

AADI 4.14908730

AADK 4.41063332

AADL 4.11486587

AADM 4.12598754

AADN 4.12657639

AADP 4.21415008

AADQ 4.48715292

AADR 4.35686014

AADS 4.71760589

AADT 4.46165271

AADV 4.48519554

AADW 3.33813714

AADY 3.60219051

AAEA 6.30664179

AAEC 4.75283488

AAED 4.60245906

AAEE 4.91456286

AAEF 3.82913317

AAEG 4.68059538

AAEH 4.24382524

AAEI 4.44084082

AAEK 4.77194085

AAEL 4.39081431

AAEM 4.40576131

AAEN 4.52134803

AAEP 4.49742213

AAEQ 4.79748708

AAER 4.64559133

AAES 5.06035118

AAET 4.76795085

AAEV 4.76756572

AAEW 3.56942016

AAEY 3.88140664

AAFA 5.36416637

AAFC 4.01575320

AAFD 3.52794927

AAFE 3.79075032

AAFF 3.34190753

AAFG 4.10587320

AAFH 3.29876226

AAFI 3.86234840

AAFK 3.72894347

AAFL 3.73785379

AAFM 3.79856014

AAFN 3.58410992

AAFP 3.59225228

AAFQ 3.77266623

AAFR 3.54483784

AAFS 4.29699987

AAFT 3.99449107

AAFV 4.07337231

AAFW 2.96672098

AAFY 3.24331882

AAGA 6.71006038

AAGC 4.89479145

AAGD 4.28984499

AAGE 4.65222461

AAGF 4.12206649

AAGG 4.86281924

AAGH 4.08549856

AAGI 4.69058083

AAGK 4.44277889

AAGL 4.63804801

AAGM 4.73873288

AAGN 4.29342994

AAGP 4.51154736

AAGQ 4.63161823

AAGR 4.40316356

AAGS 5.29296843

AAGT 4.91633625

AAGV 5.05339492

AAGW 3.66215775

AAGY 3.96284217

AAHA 5.60687717

AAHC 3.75952968

AAHD 3.91640406

AAHE 4.18760783

AAHF 3.28869875

AAHG 4.03696444

AAHH 3.46210194

AAHI 3.89026892

AAHK 3.99658006

AAHL 3.87747542

AAHM 3.83118303

AAHN 3.74799261

AAHP 3.84136556

AAHQ 4.11193494

AAHR 3.93047495

AAHS 4.42662431

AAHT 4.14612525

AAHV 4.19823092

AAHW 3.07821337

AAHY 3.30775048

AAIA 6.06107554

AAIC 4.79465600

AAID 4.09324297

AAIE 4.40579462

AAIF 3.85424735

AAIG 4.69361786

AAIH 3.90057265

AAII 4.34235275

AAIK 4.30183805

AAIL 4.25745570

AAIM 4.26173562

AAIN 4.16767359

AAIP 4.16168716

AAIQ 4.30790409

AAIR 4.09172806

AAIS 4.92460901

AAIT 4.55392553

AAIV 4.60600046

AAIW 3.50783736

AAIY 3.83313173

AAKA 6.03115556

AAKC 4.60511293

AAKD 4.41777482

AAKE 4.71814261

AAKF 3.72497327

AAKG 4.42946444

AAKH 4.00025116

AAKI 4.29478379

AAKK 4.52306657

AAKL 4.19761538

AAKM 4.20134596

AAKN 4.33689685

AAKP 4.24793210

AAKQ 4.49357418

AAKR 4.17061649

AAKS 4.83893462

AAKT 4.48640290

AAKV 4.53709053

AAKW 3.38055047

AAKY 3.82440500

AALA 5.90968569

AALC 4.66439849

AALD 4.07402340

AALE 4.33025067

AALF 3.74692092

AALG 4.57584493

AALH 3.91304372

AALI 4.24203730

AALK 4.19659575

AALL 4.17066471

AALM 4.20933987

AALN 4.04359559

AALP 4.11403733

AALQ 4.27022185

AALR 4.11838228

AALS 4.78615106

AALT 4.49887850

AALV 4.54249193

AALW 3.45842847

AALY 3.70296278

AAMA 6.04342926

AAMC 4.76009314

AAMD 4.06302858

AAME 4.35262322

AAMF 3.75610409

AAMG 4.67463847

AAMH 3.84347301

AAMI 4.24350204

AAMK 4.17479059

AAML 4.17795238

AAMM 4.19172492

AAMN 4.05322331

AAMP 4.18157558

AAMQ 4.23998819

AAMR 4.08927485

AAMS 4.81202253

AAMT 4.49485920

AAMV 4.58137235

AAMW 3.51784625

AAMY 3.78484005

AANA 5.77076742

AANC 4.37072102

AAND 4.08996804

AANE 4.37562078

AANF 3.58270453

AANG 4.25610223

AANH 3.76877579

AANI 4.14154517

AANK 4.28423672

AANL 4.02358937

AANM 4.03594314

AANN 4.13707694

AANP 4.07045394

AANQ 4.28437006

AANR 3.96863775

AANS 4.70510176

AANT 4.35003918

AANV 4.38203319

AANW 3.20593939

AANY 3.62340253

AAPA 6.26867407

AAPC 4.43457513

AAPD 4.32123813

AAPE 4.59291509

AAPF 3.66850336

AAPG 4.52347560

AAPH 3.94898140

AAPI 4.25608690

AAPK 4.31451177

AAPL 4.20398337

AAPM 4.27546870

AAPN 4.11668092

AAPP 4.41421956

AAPQ 4.48283632

AAPR 4.28699223

AAPS 4.94495426

AAPT 4.62599746

AAPV 4.67050995

AAPW 3.39292849

AAPY 3.65543033

AAQA 6.12296540

AAQC 4.60066754

AAQD 4.54219788

AAQE 4.73174524

AAQF 3.77468865

AAQG 4.60115594

AAQH 4.13550227

AAQI 4.33573537

AAQK 4.51129698

AAQL 4.28012716

AAQM 4.27117432

AAQN 4.33311066

AAQP 4.38701134

AAQQ 4.67647035

AAQR 4.40355471

AAQS 4.91045546

AAQT 4.62965020

AAQV 4.63486617

AAQW 3.58424610

AAQY 3.78624585

AARA 5.93616625

AARC 4.45705531

AARD 4.31853983

AARE 4.58120822

AARF 3.55950405

AARG 4.38913120

AARH 3.96066554

AARI 4.09872511

AARK 4.16597538

AARL 4.13696570

AARM 4.11823209

AARN 4.01936556

AARP 4.19057754

AARQ 4.38438468

AARR 4.28080350

AARS 4.68187357

AART 4.41786980

AARV 4.47869171

AARW 3.37951737

AARY 3.54375883

AASA 6.96669471

AASC 5.43930823

AASD 4.72141086

AASE 4.98222113

AASF 4.33277567

AASG 5.25751118

AASH 4.45190780

AASI 4.92507703

AASK 4.83922124

AASL 4.79204524

AASM 4.86261548

AASN 4.73104474

AASP 4.87784991

AASQ 4.88390424

AASR 4.67678321

AASS 5.58238538

AAST 5.18625907

AASV 5.28639936

AASW 3.97405341

AASY 4.30345415

AATA 6.48820413

AATC 5.04382176

AATD 4.43743167

AATE 4.67885140

AATF 4.01094718

AATG 4.91377552

AATH 4.15250717

AATI 4.56407259

AATK 4.49743590

AATL 4.50705276

AATM 4.50998088

AATN 4.39610499

AATP 4.57056932

AATQ 4.59369818

AATR 4.42085553

AATS 5.17696322

AATT 4.84252596

AATV 4.91169071

AATW 3.69037624

AATY 3.93650122

AAVA 6.61886485

AAVC 5.16262851

AAVD 4.43249069

AAVE 4.73114231

AAVF 4.06524566

AAVG 5.05029426

AAVH 4.19278635

AAVI 4.59198956

AAVK 4.54577214

AAVL 4.54997585

AAVM 4.60013067

AAVN 4.38437099

AAVP 4.57662559

AAVQ 4.63093946

AAVR 4.47403841

AAVS 5.26635644

AAVT 4.91117042

AAVV 4.98077257

AAVW 3.73506866

AAVY 4.01538366

AAWA 5.06138112

AAWC 3.43980789

AAWD 3.28305525

AAWE 3.57348213

AAWF 2.97317453

AAWG 3.72135278

AAWH 3.08738325

AAWI 3.54477662

AAWK 3.43057288

AAWL 3.51581302

AAWM 3.52593748

AAWN 3.20407906

AAWP 3.26055007

AAWQ 3.61680213

AAWR 3.42213611

AAWS 3.96492276

AAWT 3.74227034

AAWV 3.79348501

AAWW 2.65103616

AAWY 2.83083665

AAYA 5.33814126

AAYC 3.96762783

AAYD 3.54006705

AAYE 3.85190693

AAYF 3.24941045

AAYG 3.95537126

AAYH 3.33418309

AAYI 3.79006454

AAYK 3.79864518

AAYL 3.68506226

AAYM 3.78087569

AAYN 3.64388111

AAYP 3.56982210

AAYQ 3.82637941

AAYR 3.53116193

AAYS 4.25200492

AAYT 3.93567388

AAYV 4.00771312

AAYW 2.83215046

AAYY 3.24417328

ACAA 6.97116731

ACAC 12.55742193

ACAD 4.62076170

ACAE 4.56455146

ACAF 6.06409537

ACAG 5.65459501

ACAH 5.54874144

ACAI 6.40986688

ACAK 4.88338764

ACAL 6.25844190

ACAM 6.23172341

ACAN 5.55778584

ACAP 4.84700857

ACAQ 4.96033399

ACAR 5.20248712

ACAS 6.55443395

ACAT 6.38901357

ACAV 6.80852973

ACAW 5.49386626

ACAY 6.00005135

ACCA 6.01767543

ACCC 6.61099990

ACCD 3.95505297

ACCE 3.86748166

ACCF 5.05079788

ACCG 4.38884348

ACCH 4.01492771

ACCI 5.42362059

ACCK 4.10409412

ACCL 5.28979506

ACCM 5.32110852

ACCN 4.54588799

ACCP 3.55257946

ACCQ 4.21002194

ACCR 4.20468455

ACCS 5.43855832

ACCT 5.41421919

ACCV 5.77614112

ACCW 4.13090621

ACCY 4.89796159

ACDA 4.44109627

ACDC 7.96933616

ACDD 2.72594024

ACDE 2.76999498

ACDF 3.84126467

ACDG 3.25204877

ACDH 3.50997089

ACDI 4.24331767

ACDK 2.98949913

ACDL 4.09686556

ACDM 3.99194892

ACDN 3.35259663

ACDP 2.86665575

ACDQ 3.09633017

ACDR 3.21467421

ACDS 3.99886369

ACDT 3.88239155

ACDV 4.40805531

ACDW 3.35164183

ACDY 3.79281684

ACEA 4.74029570

ACEC 8.23897976

ACED 2.94581626

ACEE 2.94408221

ACEF 4.15319638

ACEG 3.60825418

ACEH 3.81188730

ACEI 4.51812611

ACEK 3.18071764

ACEL 4.37455630

ACEM 4.24000140

ACEN 3.63977989

ACEP 3.10668071

ACEQ 3.29222499

ACER 3.37541831

ACES 4.30339461

ACET 4.22633644

ACEV 4.74648211

ACEW 3.77100306

ACEY 4.08749295

ACFA 4.44528375

ACFC 7.42045255

ACFD 2.60344308

ACFE 2.56220170

ACFF 3.79299432

ACFG 3.48521361

ACFH 3.27115150

ACFI 4.10905313

ACFK 2.81807187

ACFL 3.97649020

ACFM 3.99460049

ACFN 3.28094295

ACFP 2.71315060

ACFQ 2.95456392

ACFR 2.89859667

ACFS 4.02454462

ACFT 3.92716756

ACFV 4.27220829

ACFW 3.18357064

ACFY 3.61236076

ACGA 5.41088406

ACGC 9.04198076

ACGD 3.15884689

ACGE 3.23030396

ACGF 4.65498642

ACGG 4.00446311

ACGH 4.07866280

ACGI 4.95721719

ACGK 3.40051039

ACGL 4.88425940

ACGM 4.86891907

ACGN 3.99216804

ACGP 3.43964035

ACGQ 3.66055714

ACGR 3.61174924

ACGS 4.89816991

ACGT 4.78196412

ACGV 5.17295751

ACGW 3.98771366

ACGY 4.40354694

ACHA 4.49622489

ACHC 7.30427617

ACHD 2.82571671

ACHE 2.86332013

ACHF 3.82277491

ACHG 3.41917557

ACHH 3.29132025

ACHI 4.23510094

ACHK 2.94381235

ACHL 4.13593406

ACHM 3.92468163

ACHN 3.36782270

ACHP 2.86552469

ACHQ 3.13158719

ACHR 3.08538955

ACHS 4.07859571

ACHT 3.96364628

ACHV 4.39470296

ACHW 3.38989711

ACHY 3.69209341

ACIA 5.03274388

ACIC 8.21467563

ACID 3.04968410

ACIE 3.11117701

ACIF 4.41684247

ACIG 4.04085498

ACIH 3.85772337

ACII 4.58184607

ACIK 3.29505890

ACIL 4.49250672

ACIM 4.39479440

ACIN 3.74421328

ACIP 3.26579421

ACIQ 3.38590814

ACIR 3.42880738

ACIS 4.60248206

ACIT 4.47567513

ACIV 4.77627970

ACIW 3.92156908

ACIY 4.22091845

ACKA 4.62485686

ACKC 8.16540039

ACKD 2.85349555

ACKE 2.82448927

ACKF 4.13228554

ACKG 3.54838623

ACKH 3.68823715

ACKI 4.41230102

ACKK 3.09280204

ACKL 4.23969017

ACKM 4.10242725

ACKN 3.49518358

ACKP 3.05491890

ACKQ 3.13001368

ACKR 3.21746473

ACKS 4.18203628

ACKT 4.05675775

ACKV 4.56658095

ACKW 3.58123942

ACKY 4.04853853

ACLA 4.86206475

ACLC 7.88810584

ACLD 2.91994735

ACLE 2.93121902

ACLF 4.17589227

ACLG 3.86036125

ACLH 3.75452276

ACLI 4.46060777

ACLK 3.12601525

ACLL 4.32676962

ACLM 4.29661748

ACLN 3.58949301

ACLP 3.15428005

ACLQ 3.25089629

ACLR 3.28880221

ACLS 4.39279897

ACLT 4.31557489

ACLV 4.67391114

ACLW 3.67742294

ACLY 4.06119041

ACMA 4.92292078

ACMC 8.04066447

ACMD 3.02916848

ACME 2.99273598

ACMF 4.21201261

ACMG 3.96845382

ACMH 3.75495676

ACMI 4.51489935

ACMK 3.17568125

ACML 4.40072383

ACMM 4.30789026

ACMN 3.63829401

ACMP 3.23767797

ACMQ 3.30498784

ACMR 3.33315783

ACMS 4.50022021

ACMT 4.37746814

ACMV 4.74490260

ACMW 3.87902607

ACMY 4.16893228

ACNA 4.51048660

ACNC 8.12601687

ACND 2.82946142

ACNE 2.82838572

ACNF 3.99378307

ACNG 3.47802936

ACNH 3.49930249

ACNI 4.30071816

ACNK 3.05991767

ACNL 4.14842078

ACNM 4.07770609

ACNN 3.49979615

ACNP 2.93265529

ACNQ 3.08633945

ACNR 3.15755898

ACNS 4.12697418

ACNT 3.97974387

ACNV 4.41700697

ACNW 3.41488926

ACNY 3.88755790

ACPA 4.86309903

ACPC 8.83422757

ACPD 3.05706033

ACPE 2.98896151

ACPF 4.12787164

ACPG 3.66438687

ACPH 3.77038389

ACPI 4.45981733

ACPK 3.13641475

ACPL 4.37451959

ACPM 4.32749998

ACPN 3.70822111

ACPP 3.14679353

ACPQ 3.44094133

ACPR 3.43407064

ACPS 4.52784822

ACPT 4.42209695

ACPV 4.72650779

ACPW 3.57462170

ACPY 4.04093687

ACQA 4.63985564

ACQC 8.15593712

ACQD 2.92873986

ACQE 2.87863456

ACQF 4.22237762

ACQG 3.63912481

ACQH 3.74005634

ACQI 4.42870904

ACQK 3.09914953

ACQL 4.28817181

ACQM 4.17803455

ACQN 3.48485349

ACQP 3.10643813

ACQQ 3.23435692

ACQR 3.28109861

ACQS 4.22480526

ACQT 4.16469706

ACQV 4.61029581

ACQW 3.80701755

ACQY 4.05713336

ACRA 4.57317375

ACRC 7.89764480

ACRD 2.81551499

ACRE 2.80305278

ACRF 3.94410142

ACRG 3.45005549

ACRH 3.55265044

ACRI 4.24228206

ACRK 2.88956237

ACRL 4.18156176

ACRM 4.03731252

ACRN 3.37323974

ACRP 2.93805944

ACRQ 3.06066596

ACRR 3.15632936

ACRS 4.08819470

ACRT 4.01017725

ACRV 4.49835186

ACRW 3.52437381

ACRY 3.79248416

ACSA 5.57384962

ACSC 9.48488111

ACSD 3.50406182

ACSE 3.49154185

ACSF 5.05261052

ACSG 4.41081336

ACSH 4.38768967

ACSI 5.22709373

ACSK 3.73252028

ACSL 5.07470320

ACSM 5.01160453

ACSN 4.32399275

ACSP 3.75147802

ACSQ 3.85298969

ACSR 3.96357172

ACSS 5.16511609

ACST 4.99504283

ACSV 5.43807147

ACSW 4.42211997

ACSY 4.80292855

ACTA 5.17530905

ACTC 8.84506747

ACTD 3.16374692

ACTE 3.11495620

ACTF 4.51151045

ACTG 4.05852195

ACTH 4.00944455

ACTI 4.80103901

ACTK 3.33468618

ACTL 4.70227001

ACTM 4.59809257

ACTN 3.94233154

ACTP 3.39216967

ACTQ 3.48861974

ACTR 3.60798848

ACTS 4.75140085

ACTT 4.54176562

ACTV 4.99068795

ACTW 4.07394678

ACTY 4.35837746

ACVA 5.43460659

ACVC 8.93140996

ACVD 3.35413420

ACVE 3.39078648

ACVF 4.63875545

ACVG 4.33262237

ACVH 4.18823857

ACVI 4.87255802

ACVK 3.52967910

ACVL 4.81350964

ACVM 4.75350561

ACVN 4.05219280

ACVP 3.52418490

ACVQ 3.69715545

ACVR 3.74989407

ACVS 4.96624422

ACVT 4.82955095

ACVV 5.13704388

ACVW 4.18285787

ACVY 4.49062135

ACWA 4.13501458

ACWC 6.68924176

ACWD 2.36068365

ACWE 2.47085517

ACWF 3.34363880

ACWG 3.11463678

ACWH 3.03293088

ACWI 3.78179586

ACWK 2.55177030

ACWL 3.65471015

ACWM 3.73901867

ACWN 2.88327193

ACWP 2.46395141

ACWQ 2.78643990

ACWR 2.69120697

ACWS 3.74556089

ACWT 3.62499660

ACWV 3.94922655

ACWW 2.87396886

ACWY 3.16618307

ACYA 4.31310761

ACYC 7.42510864

ACYD 2.64549715

ACYE 2.59482539

ACYF 3.64711615

ACYG 3.34355078

ACYH 3.21410701

ACYI 4.04431806

ACYK 2.80874277

ACYL 3.88279027

ACYM 3.86716980

ACYN 3.21389217

ACYP 2.70209734

ACYQ 2.94498671

ACYR 2.81999770

ACYS 3.89119205

ACYT 3.79811313

ACYV 4.19477562

ACYW 3.08490106

ACYY 3.51200431

ADAA 5.93417417

ADAC 4.62076170

ADAD 10.73563757

ADAE 7.53773286

ADAF 4.06049459

ADAG 6.31362958

ADAH 6.18551455

ADAI 4.18334831

ADAK 6.25287615

ADAL 4.32338098

ADAM 4.61436344

ADAN 7.25840908

ADAP 6.03666860

ADAQ 6.44569811

ADAR 5.94918696

ADAS 6.47212978

ADAT 6.03602028

ADAV 4.49178135

ADAW 4.29079217

ADAY 4.79122089

ADCA 4.44293464

ADCC 1.10332104

ADCD 7.69587462

ADCE 6.07568646

ADCF 2.83587276

ADCG 4.44555234

ADCH 4.23836116

ADCI 3.01989247

ADCK 4.92879189

ADCL 3.12853648

ADCM 3.49207456

ADCN 5.69950046

ADCP 4.25482012

ADCQ 5.09938146

ADCR 4.57079818

ADCS 5.10531635

ADCT 4.70193748

ADCV 3.28414215

ADCW 2.86480844

ADCY 3.55286021

ADDA 4.71908056

ADDC 3.14420665

ADDD 6.72200017

ADDE 5.62830324

ADDF 2.88778334

ADDG 4.66536129

ADDH 4.51101193

ADDI 3.12270990

ADDK 4.76689486

ADDL 3.21813835

ADDM 3.45148446

ADDN 5.39334009

ADDP 4.47757185

ADDQ 4.98624215

ADDR 4.51745764

ADDS 4.96306354

ADDT 4.64480412

ADDV 3.39023327

ADDW 2.99209315

ADDY 3.38146996

ADEA 4.87077573

ADEC 3.35900884

ADED 7.02859677

ADEE 5.86222404

ADEF 3.04818896

ADEG 5.01233905

ADEH 4.74221154

ADEI 3.26887441

ADEK 4.98362755

ADEL 3.36749257

ADEM 3.62933279

ADEN 5.68215525

ADEP 4.73968173

ADEQ 5.15730543

ADER 4.68628055

ADES 5.15544046

ADET 4.82194856

ADEV 3.53841560

ADEW 3.12208986

ADEY 3.56380420

ADFA 3.50160722

ADFC 2.36948629

ADFD 6.20789228

ADFE 4.84889046

ADFF 2.01707170

ADFG 3.77719505

ADFH 3.76072528

ADFI 2.19513027

ADFK 4.04742971

ADFL 2.24607212

ADFM 2.49846574

ADFN 4.72803339

ADFP 3.69599412

ADFQ 4.13835400

ADFR 3.69262982

ADFS 4.11499361

ADFT 3.74560530

ADFV 2.38520110

ADFW 2.18788681

ADFY 2.66894878

ADGA 4.56203799

ADGC 3.20809740

ADGD 7.53870226

ADGE 5.91792511

ADGF 2.89200285

ADGG 4.54205557

ADGH 4.68331340

ADGI 3.10191757

ADGK 4.85265818

ADGL 3.19516471

ADGM 3.49032795

ADGN 5.66548758

ADGP 4.55633419

ADGQ 5.07877257

ADGR 4.59063475

ADGS 5.04494449

ADGT 4.66822663

ADGV 3.32039169

ADGW 3.02767369

ADGY 3.49892133

ADHA 4.09372406

ADHC 2.41092739

ADHD 6.42122591

ADHE 5.24881648

ADHF 2.44272578

ADHG 4.18195778

ADHH 3.98584825

ADHI 2.66275985

ADHK 4.31966706

ADHL 2.85280983

ADHM 2.93924611

ADHN 4.95766976

ADHP 4.12129911

ADHQ 4.52355241

ADHR 4.09042784

ADHS 4.47048128

ADHT 4.12841605

ADHV 2.86741921

ADHW 2.61975683

ADHY 2.96944879

ADIA 3.94879160

ADIC 2.89006548

ADID 6.86738884

ADIE 5.47253216

ADIF 2.60230807

ADIG 4.29949032

ADIH 4.34285062

ADII 2.62921816

ADIK 4.58511083

ADIL 2.69000224

ADIM 2.95678105

ADIN 5.29957150

ADIP 4.24817720

ADIQ 4.66402076

ADIR 4.19696222

ADIS 4.63314917

ADIT 4.21707490

ADIV 2.75435021

ADIW 2.73113559

ADIY 3.25202683

ADKA 4.46469801

ADKC 3.20405914

ADKD 6.77675607

ADKE 5.62111366

ADKF 2.91060860

ADKG 4.70273201

ADKH 4.49944812

ADKI 3.13852596

ADKK 4.78572079

ADKL 3.19696785

ADKM 3.39930219

ADKN 5.48911265

ADKP 4.45801636

ADKQ 4.85106473

ADKR 4.29389929

ADKS 4.90193651

ADKT 4.53446969

ADKV 3.26713968

ADKW 2.93479687

ADKY 3.48557943

ADLA 3.99556612

ADLC 2.76051829

ADLD 6.67331565

ADLE 5.32525528

ADLF 2.42194231

ADLG 4.29219386

ADLH 4.25297642

ADLI 2.52776211

ADLK 4.42914944

ADLL 2.59685127

ADLM 2.90749569

ADLN 5.09998120

ADLP 4.21966117

ADLQ 4.56265230

ADLR 4.16482460

ADLS 4.55511432

ADLT 4.18797498

ADLV 2.76577631

ADLW 2.61168872

ADLY 3.04568588

ADMA 3.98083247

ADMC 2.87835744

ADMD 6.84557087

ADME 5.45885078

ADMF 2.50850953

ADMG 4.32970394

ADMH 4.33400754

ADMI 2.63546044

ADMK 4.46594622

ADML 2.73736790

ADMM 2.94313732

ADMN 5.17273040

ADMP 4.26891925

ADMQ 4.55220691

ADMR 4.17921543

ADMS 4.55245931

ADMT 4.17461934

ADMV 2.83604856

ADMW 2.73683422

ADMY 3.20424322

ADNA 4.20572992

ADNC 3.06887683

ADND 6.67830161

ADNE 5.48742816

ADNF 2.79923175

ADNG 4.43786714

ADNH 4.33408645

ADNI 3.02860367

ADNK 4.65095473

ADNL 3.05995465

ADNM 3.26074496

ADNN 5.30916395

ADNP 4.25562442

ADNQ 4.74597798

ADNR 4.18155566

ADNS 4.74349967

ADNT 4.37570412

ADNV 3.16383330

ADNW 2.79945077

ADNY 3.33269890

ADPA 4.68465623

ADPC 3.01754200

ADPD 7.27366903

ADPE 5.84706050

ADPF 2.85762356

ADPG 4.71533376

ADPH 4.63795779

ADPI 3.05536464

ADPK 4.73887839

ADPL 3.17707703

ADPM 3.41419711

ADPN 5.51955341

ADPP 4.48274383

ADPQ 5.02963396

ADPR 4.47160233

ADPS 5.03796481

ADPT 4.70348698

ADPV 3.38148347

ADPW 2.95274564

ADPY 3.39878104

ADQA 4.60492051

ADQC 3.13568751

ADQD 6.87749381

ADQE 5.67472023

ADQF 2.86454787

ADQG 4.76707249

ADQH 4.56454917

ADQI 3.06372825

ADQK 4.73297199

ADQL 3.16976800

ADQM 3.35425632

ADQN 5.41842494

ADQP 4.57484260

ADQQ 4.95181131

ADQR 4.46346942

ADQS 4.90011534

ADQT 4.58189147

ADQV 3.31573458

ADQW 3.09873575

ADQY 3.40249510

ADRA 4.48125676

ADRC 2.98161181

ADRD 6.62176249

ADRE 5.43855131

ADRF 2.65340380

ADRG 4.57788318

ADRH 4.36591215

ADRI 2.82301065

ADRK 4.42722151

ADRL 2.98066496

ADRM 3.16495113

ADRN 5.11709545

ADRP 4.39727044

ADRQ 4.70293771

ADRR 4.33546933

ADRS 4.71161156

ADRT 4.38101591

ADRV 3.13891867

ADRW 2.84485339

ADRY 3.12967693

ADSA 4.87235102

ADSC 3.66469807

ADSD 7.80712843

ADSE 6.24639975

ADSF 3.27898968

ADSG 5.14971418

ADSH 5.01121967

ADSI 3.44521583

ADSK 5.19661038

ADSL 3.54674060

ADSM 3.78493934

ADSN 6.02849059

ADSP 4.90543764

ADSQ 5.33526507

ADSR 4.83826006

ADSS 5.37341491

ADST 4.97681384

ADSV 3.67893952

ADSW 3.38514474

ADSY 3.91842996

ADTA 4.57718529

ADTC 3.34686623

ADTD 7.27434978

ADTE 5.84783842

ADTF 2.94078849

ADTG 4.81604904

ADTH 4.67139694

ADTI 3.06192168

ADTK 4.83834438

ADTL 3.18013303

ADTM 3.42328377

ADTN 5.63286022

ADTP 4.64646409

ADTQ 5.01884491

ADTR 4.55607724

ADTS 5.03010845

ADTT 4.65517049

ADTV 3.31796386

ADTW 3.16165732

ADTY 3.51526831

ADVA 4.41360951

ADVC 3.19172527

ADVD 7.47937876

ADVE 5.89304051

ADVF 2.75788255

ADVG 4.75520744

ADVH 4.69470047

ADVI 2.82167553

ADVK 4.84971926

ADVL 2.98167524

ADVM 3.29514677

ADVN 5.66260442

ADVP 4.61453248

ADVQ 5.01427832

ADVR 4.57084240

ADVS 5.00923903

ADVT 4.60138467

ADVV 3.06975308

ADVW 3.01278699

ADVY 3.45909453

ADWA 3.36011405

ADWC 1.92530282

ADWD 5.80311271

ADWE 4.60334706

ADWF 1.71315052

ADWG 3.51784302

ADWH 3.48546727

ADWI 2.04129857

ADWK 3.70572991

ADWL 2.09360939

ADWM 2.42845179

ADWN 4.27965808

ADWP 3.41543076

ADWQ 3.92319022

ADWR 3.48586790

ADWS 3.84673860

ADWT 3.54238856

ADWV 2.20900186

ADWW 1.91369037

ADWY 2.30472763

ADYA 3.59659204

ADYC 2.46142515

ADYD 6.17285470

ADYE 4.93733471

ADYF 2.22208142

ADYG 3.90446334

ADYH 3.81652651

ADYI 2.44107910

ADYK 4.16665239

ADYL 2.49313577

ADYM 2.75033353

ADYN 4.83186343

ADYP 3.75372243

ADYQ 4.23341032

ADYR 3.72153156

ADYS 4.20766919

ADYT 3.89470632

ADYV 2.58566371

ADYW 2.27294061

ADYY 2.82447302

AEAA 6.26171667

AEAC 4.56455146

AEAD 7.53773286

AEAE 10.24464880

AEAF 4.23718073

AEAG 5.78205549

AEAH 6.06319832

AEAI 4.80437455

AEAK 6.78784988

AEAL 4.87745912

AEAM 5.16102603

AEAN 6.35370015

AEAP 5.93441510

AEAQ 7.13135032

AEAR 6.44549561

AEAS 6.24197039

AEAT 6.09705617

AEAV 5.08698694

AEAW 4.50490903

AEAY 4.93679347

AECA 4.66896649

AECC 0.86266302

AECD 5.91105446

AECE 7.18457674

AECF 3.01243282

AECG 3.93285248

AECH 4.10100565

AECI 3.55232089

AECK 5.33685072

AECL 3.60789220

AECM 3.95599420

AECN 4.87119668

AECP 4.09466939

AECQ 5.57687184

AECR 4.96092123

AECS 4.82971231

AECT 4.70105128

AECV 3.78916237

AECW 2.99899883

AECY 3.74648943

AEDA 4.91111468

AEDC 3.11487996

AEDD 5.52974999

AEDE 6.39129436

AEDF 3.01546043

AEDG 4.17782091

AEDH 4.40937699

AEDI 3.51573444

AEDK 5.11687948

AEDL 3.56158955

AEDM 3.78642376

AEDN 4.77474492

AEDP 4.36085515

AEDQ 5.37570178

AEDR 4.85527354

AEDS 4.76388028

AEDT 4.64273361

AEDV 3.76740783

AEDW 3.09289737

AEDY 3.47523635

AEEA 5.22737919

AEEC 3.44245949

AEED 5.90115155

AEEE 6.74124174

AEEF 3.28634258

AEEG 4.61461011

AEEH 4.75300827

AEEI 3.84741348

AEEK 5.50071162

AEEL 3.89263458

AEEM 4.13329063

AEEN 5.19736122

AEEP 4.66905233

AEEQ 5.71145024

AEER 5.18605180

AEES 5.09576028

AEET 4.96772348

AEEV 4.07835025

AEEW 3.37295225

AEEY 3.77871835

AEFA 3.62185223

AEFC 2.17966335

AEFD 4.77396354

AEFE 5.71374259

AEFF 2.12164965

AEFG 3.28109116

AEFH 3.58869166

AEFI 2.56718221

AEFK 4.34604235

AEFL 2.56439609

AEFM 2.85085434

AEFN 4.02403500

AEFP 3.48543304

AEFQ 4.52945041

AEFR 3.96425060

AEFS 3.87493332

AEFT 3.67454381

AEFV 2.68744659

AEFW 2.25108157

AEFY 2.76539910

AEGA 4.69298600

AEGC 2.87537186

AEGD 5.68167892

AEGE 7.05204528

AEGF 2.95662165

AEGG 3.99080150

AEGH 4.45325738

AEGI 3.46155189

AEGK 5.13984583

AEGL 3.54407020

AEGM 3.85059820

AEGN 4.75496639

AEGP 4.35014437

AEGQ 5.51153791

AEGR 4.87104948

AEGS 4.71656072

AEGT 4.58782844

AEGV 3.70248511

AEGW 3.13252675

AEGY 3.50080774

AEHA 4.25807410

AEHC 2.24055682

AEHD 5.15019131

AEHE 6.03309021

AEHF 2.53841535

AEHG 3.73158782

AEHH 3.85330975

AEHI 3.05911207

AEHK 4.66124831

AEHL 3.16327414

AEHM 3.24544741

AEHN 4.29691046

AEHP 3.94352933

AEHQ 4.93961494

AEHR 4.38876034

AEHS 4.27116818

AEHT 4.14481399

AEHV 3.27990238

AEHW 2.70796102

AEHY 3.03991099

AEIA 4.15837787

AEIC 2.80342503

AEID 5.44983047

AEIE 6.41227395

AEIF 2.69714100

AEIG 3.87311513

AEIH 4.26059173

AEII 3.03104067

AEIK 4.96235225

AEIL 3.09891325

AEIM 3.37617014

AEIN 4.67189036

AEIP 4.06141347

AEIQ 5.13465628

AEIR 4.56977155

AEIS 4.45807766

AEIT 4.25109140

AEIV 3.16321642

AEIW 2.88806541

AEIY 3.39027686

AEKA 4.77667660

AEKC 3.19786637

AEKD 5.67472558

AEKE 6.46690065

AEKF 3.13143353

AEKG 4.26893935

AEKH 4.47673517

AEKI 3.64478257

AEKK 5.19177957

AEKL 3.65427693

AEKM 3.84978466

AEKN 4.95554351

AEKP 4.37127990

AEKQ 5.35529702

AEKR 4.69847956

AEKS 4.79662258

AEKT 4.64161636

AEKV 3.77171326

AEKW 3.13680264

AEKY 3.66664322

AELA 4.21980714

AELC 2.69128550

AELD 5.31966542

AELE 6.21142431

AELF 2.55060797

AELG 3.87484962

AELH 4.18136859

AELI 2.93707180

AELK 4.82433827

AELL 2.97718092

AELM 3.28812397

AELN 4.48813390

AELP 4.05433340

AELQ 5.03027753

AELR 4.51476879

AELS 4.37870566

AELT 4.19391042

AELV 3.12870696

AELW 2.75701967

AELY 3.20266779

AEMA 4.22356568

AEMC 2.82054069

AEMD 5.47258123

AEME 6.42937548

AEMF 2.63814643

AEMG 3.90947101

AEMH 4.22057787

AEMI 3.05266229

AEMK 4.85421907

AEML 3.12074937

AEMM 3.35377911

AEMN 4.53396452

AEMP 4.09344540

AEMQ 5.07322572

AEMR 4.54989412

AEMS 4.37738174

AEMT 4.19830424

AEMV 3.24390566

AEMW 2.93146047

AEMY 3.34598389

AENA 4.36769628

AENC 2.91913760

AEND 5.38587185

AENE 6.29771835

AENF 2.88066545

AENG 3.92406543

AENH 4.22710458

AENI 3.39993952

AENK 4.97686406

AENL 3.38968392

AENM 3.57675506

AENN 4.67679914

AENP 4.09533743

AENQ 5.12668282

AENR 4.47481810

AENS 4.52812178

AENT 4.35399062

AENV 3.51711291

AENW 2.90771673

AENY 3.43741817

AEPA 4.78621418

AEPC 2.81985083

AEPD 5.65246024

AEPE 6.69806860

AEPF 2.86443464

AEPG 4.11069665

AEPH 4.38704226

AEPI 3.40535108

AEPK 4.99779545

AEPL 3.44930985

AEPM 3.65682872

AEPN 4.64320009

AEPP 4.34062159

AEPQ 5.37313676

AEPR 4.73590824

AEPS 4.71289787

AEPT 4.58042212

AEPV 3.73705316

AEPW 2.98272099

AEPY 3.38747490

AEQA 4.90131010

AEQC 3.15096018

AEQD 5.76487769

AEQE 6.55492659

AEQF 3.05768449

AEQG 4.33777143

AEQH 4.54709450

AEQI 3.59029638

AEQK 5.16397213

AEQL 3.62625811

AEQM 3.79903568

AEQN 4.87053613

AEQP 4.46043764

AEQQ 5.46728214

AEQR 4.87120817

AEQS 4.79990742

AEQT 4.69367833

AEQV 3.77876514

AEQW 3.21547648

AEQY 3.55484597

AERA 4.73164541

AERC 2.96796674

AERD 5.44199322

AERE 6.26000909

AERF 2.81297351

AERG 4.13358840

AERH 4.30845429

AERI 3.29759974

AERK 4.79837555

AERL 3.40386334

AERM 3.60984882

AERN 4.51133873

AERP 4.24706819

AERQ 5.15018080

AERR 4.69143556

AERS 4.54864085

AERT 4.44159503

AERV 3.58779538

AERW 3.03298663

AERY 3.26421580

AESA 5.05564781

AESC 3.55457354

AESD 6.18642654

AESE 7.33767287

AESF 3.40788170

AESG 4.62713574

AESH 4.88719221

AESI 3.89971359

AESK 5.56785217

AESL 3.94412708

AESM 4.19310896

AESN 5.26207564

AESP 4.76764341

AESQ 5.83318713

AESR 5.18344599

AESS 5.13518961

AEST 4.96541326

AESV 4.12594651

AESW 3.50478905

AESY 3.99879015

AETA 4.76039233

AETC 3.19271777

AETD 5.79774130

AETE 6.83149900

AETF 3.03566787

AETG 4.30687656

AETH 4.55227947

AETI 3.50449073

AETK 5.18532694

AETL 3.57397421

AETM 3.79494190

AETN 4.89845901

AETP 4.48714325

AETQ 5.48470948

AETR 4.90104908

AETS 4.79506504

AETT 4.66658248

AETV 3.74186782

AETW 3.23929779

AETY 3.59376237

AEVA 4.63047849

AEVC 3.08986843

AEVD 5.85616756

AEVE 6.95482332

AEVF 2.90429238

AEVG 4.27857711

AEVH 4.55439873

AEVI 3.29970497

AEVK 5.26197571

AEVL 3.39023295

AEVM 3.69581873

AEVN 4.90799461

AEVP 4.47125070

AEVQ 5.51580044

AEVR 4.95613806

AEVS 4.78919623

AEVT 4.63189680

AEVV 3.52181972

AEVW 3.15684441

AEVY 3.57256006

AEWA 3.44465685

AEWC 1.75493225

AEWD 4.46121131

AEWE 5.38898848

AEWF 1.88562362

AEWG 3.04261787

AEWH 3.32190188

AEWI 2.33866293

AEWK 3.96175670

AEWL 2.35045104

AEWM 2.65715197

AEWN 3.57812854

AEWP 3.18943964

AEWQ 4.26299392

AEWR 3.73272269

AEWS 3.56844588

AEWT 3.44494952

AEWV 2.48999278

AEWW 1.99646067

AEWY 2.36064761

AEYA 3.72487833

AEYC 2.39927598

AEYD 4.79139184

AEYE 5.72123001

AEYF 2.30045460

AEYG 3.41679309

AEYH 3.66899373

AEYI 2.78168039

AEYK 4.45928228

AEYL 2.77808360

AEYM 3.15552676

AEYN 4.14893886

AEYP 3.58819682

AEYQ 4.62435462

AEYR 3.99876957

AEYS 3.94414978

AEYT 3.79703784

AEYV 2.90413032

AEYW 2.33863740

AEYY 2.89656050

AFAA 5.41958689

AFAC 6.06409537

AFAD 4.06049459

AFAE 4.23718073

AFAF 11.11297640

AFAG 4.53729292

AFAH 6.06763184

AFAI 6.78574488

AFAK 4.39790659

AFAL 7.18834356

AFAM 6.86974354

AFAN 4.71439425

AFAP 4.60609856

AFAQ 4.70228934

AFAR 4.65770970

AFAS 4.98837329

AFAT 5.24964991

AFAV 6.36005365

AFAW 7.52241693

AFAY 8.28493555

AFCA 4.46565392

AFCC 2.29710836

AFCD 2.98820066

AFCE 3.33189674

AFCF 7.90753037

AFCG 3.06618270

AFCH 4.17109807

AFCI 5.60643967

AFCK 3.39689308

AFCL 5.90018304

AFCM 5.62725589

AFCN 3.51946002

AFCP 3.22567127

AFCQ 3.68082178

AFCR 3.55836575

AFCS 3.86681921

AFCT 4.23184749

AFCV 5.24423717

AFCW 5.71292909

AFCY 6.53309182

AFDA 3.45304089

AFDC 3.69585829

AFDD 2.40309170

AFDE 2.54086214

AFDF 6.87835972

AFDG 2.51858358

AFDH 3.81925797

AFDI 4.60643268

AFDK 2.62071430

AFDL 4.85230464

AFDM 4.53170254

AFDN 2.78821944

AFDP 2.78581789

AFDQ 2.87099813

AFDR 2.82886541

AFDS 2.99846626

AFDT 3.24901180

AFDV 4.26747952

AFDW 5.26175673

AFDY 5.63233811

AFEA 3.64643895

AFEC 4.06414503

AFED 2.54901705

AFEE 2.71205552

AFEF 7.20479289

AFEG 2.82357028

AFEH 4.06702686

AFEI 4.81353047

AFEK 2.78964355

AFEL 5.04636477

AFEM 4.74017309

AFEN 3.03724882

AFEP 2.93994509

AFEQ 3.01900467

AFER 3.00277845

AFES 3.23301401

AFET 3.47835931

AFEV 4.46436334

AFEW 5.42524703

AFEY 5.89223536

AFFA 3.82066002

AFFC 3.85906209

AFFD 2.35274432

AFFE 2.53411777

AFFF 6.41788386

AFFG 2.97937603

AFFH 3.70402487

AFFI 4.74541161

AFFK 2.69496788

AFFL 4.85705499

AFFM 4.64251339

AFFN 2.92506196

AFFP 2.86447713

AFFQ 2.97388426

AFFR 2.83436552

AFFS 3.24600689

AFFT 3.51405515

AFFV 4.40922546

AFFW 5.03117012

AFFY 5.35972968

AFGA 4.31278721

AFGC 4.28216389

AFGD 2.73321621

AFGE 3.04666487

AFGF 7.83883123

AFGG 3.19864806

AFGH 4.42460835

AFGI 5.48397485

AFGK 3.13053057

AFGL 5.80198201

AFGM 5.53389922

AFGN 3.43088075

AFGP 3.35203727

AFGQ 3.56842849

AFGR 3.38010857

AFGS 3.75767425

AFGT 4.01705025

AFGV 5.06289368

AFGW 5.88758779

AFGY 6.44998843

AFHA 3.42186966

AFHC 3.32100975

AFHD 2.29565097

AFHE 2.46282645

AFHF 6.59383374

AFHG 2.51729748

AFHH 3.49542509

AFHI 4.49318556

AFHK 2.50496604

AFHL 4.74210332

AFHM 4.39894449

AFHN 2.71830146

AFHP 2.68458677

AFHQ 2.79366332

AFHR 2.78891892

AFHS 2.95208673

AFHT 3.20110061

AFHV 4.16751441

AFHW 5.00296493

AFHY 5.43464656

AFIA 4.06640515

AFIC 4.37937708

AFID 2.69693407

AFIE 2.89016405

AFIF 7.15677216

AFIG 3.24858346

AFIH 4.19190839

AFII 5.13156028

AFIK 3.04844595

AFIL 5.35380003

AFIM 5.05147303

AFIN 3.29503010

AFIP 3.22839781

AFIQ 3.27907745

AFIR 3.21389686

AFIS 3.58265575

AFIT 3.83434155

AFIV 4.76980198

AFIW 5.61023453

AFIY 6.00311706

AFKA 3.52911824

AFKC 3.97543697

AFKD 2.41346865

AFKE 2.56451793

AFKF 7.01665444

AFKG 2.71783285

AFKH 3.89876826

AFKI 4.73793415

AFKK 2.69317125

AFKL 4.94004693

AFKM 4.62190320

AFKN 2.95225668

AFKP 2.87582233

AFKQ 2.84598647

AFKR 2.82250378

AFKS 3.08435013

AFKT 3.30826850

AFKV 4.33279204

AFKW 5.27792843

AFKY 5.75924523

AFLA 4.02638489

AFLC 4.24782034

AFLD 2.60168274

AFLE 2.74913490

AFLF 6.91879091

AFLG 3.18057329

AFLH 4.10719083

AFLI 4.99761454

AFLK 2.88235923

AFLL 5.16487210

AFLM 4.89516168

AFLN 3.16912558

AFLP 3.14380013

AFLQ 3.14014519

AFLR 3.10984195

AFLS 3.45697355

AFLT 3.76020176

AFLV 4.70227977

AFLW 5.41352649

AFLY 5.77900580

AFMA 4.04021899

AFMC 4.38988708

AFMD 2.70226029

AFME 2.86189457

AFMF 7.11836063

AFMG 3.27406657

AFMH 4.18334458

AFMI 5.03531229

AFMK 2.91238955

AFML 5.27261678

AFMM 4.96295217

AFMN 3.23422969

AFMP 3.23368105

AFMQ 3.16562903

AFMR 3.15053864

AFMS 3.50148804

AFMT 3.76681960

AFMV 4.74072989

AFMW 5.54758061

AFMY 5.93638933

AFNA 3.49187630

AFNC 3.78412305

AFND 2.44156777

AFNE 2.58207873

AFNF 6.87963548

AFNG 2.73365819

AFNH 3.84263114

AFNI 4.69411087

AFNK 2.72165479

AFNL 4.90499775

AFNM 4.60167962

AFNN 2.98353789

AFNP 2.85075161

AFNQ 2.93604259

AFNR 2.81199425

AFNS 3.12202440

AFNT 3.31675734

AFNV 4.28594170

AFNW 5.26729045

AFNY 5.67521484

AFPA 3.81602451

AFPC 3.87278269

AFPD 2.58689848

AFPE 2.76569637

AFPF 7.39420054

AFPG 2.88056454

AFPH 4.18927650

AFPI 4.97059949

AFPK 2.88708986

AFPL 5.24482774

AFPM 4.92454855

AFPN 3.13818967

AFPP 3.07531301

AFPQ 3.23776852

AFPR 3.14948094

AFPS 3.38326340

AFPT 3.63286402

AFPV 4.61330157

AFPW 5.49384794

AFPY 6.04437805

AFQA 3.63495983

AFQC 3.98068696

AFQD 2.52431670

AFQE 2.63246902

AFQF 7.10857474

AFQG 2.88034761

AFQH 3.99900230

AFQI 4.78624256

AFQK 2.73036471

AFQL 5.02022689

AFQM 4.70132161

AFQN 2.96903182

AFQP 2.97728728

AFQQ 2.94945727

AFQR 2.97942798

AFQS 3.17197723

AFQT 3.43337508

AFQV 4.44090289

AFQW 5.43307102

AFQY 5.85423802

AFRA 3.57179519

AFRC 3.88268247

AFRD 2.40136963

AFRE 2.55204934

AFRF 6.84137468

AFRG 2.69534275

AFRH 3.89655062

AFRI 4.61498390

AFRK 2.53383369

AFRL 4.87037323

AFRM 4.56685627

AFRN 2.83537831

AFRP 2.84985722

AFRQ 2.86355379

AFRR 2.83980476

AFRS 3.07590275

AFRT 3.32589059

AFRV 4.33643361

AFRW 5.19363759

AFRY 5.58896296

AFSA 4.31953064

AFSC 4.77714422

AFSD 3.02812814

AFSE 3.16847376

AFSF 8.20901156

AFSG 3.48648358

AFSH 4.75355380

AFSI 5.59002300

AFSK 3.33043203

AFSL 5.89181681

AFSM 5.59411911

AFSN 3.64917120

AFSP 3.52760631

AFSQ 3.61711067

AFSR 3.54428992

AFSS 3.88296910

AFST 4.09477579

AFSV 5.15754374

AFSW 6.21938193

AFSY 6.76567845

AFTA 4.05318097

AFTC 4.44595810

AFTD 2.75189337

AFTE 2.88619489

AFTF 7.67543678

AFTG 3.21660543

AFTH 4.38353992

AFTI 5.27035961

AFTK 3.02626876

AFTL 5.56970600

AFTM 5.23535555

AFTN 3.34098727

AFTP 3.29710233

AFTQ 3.33932302

AFTR 3.28478191

AFTS 3.58910547

AFTT 3.80959606

AFTV 4.87167686

AFTW 5.84786549

AFTY 6.29829393

AFVA 4.31342613

AFVC 4.62528825

AFVD 2.92947936

AFVE 3.14154447

AFVF 7.78099634

AFVG 3.42264883

AFVH 4.55834921

AFVI 5.42068209

AFVK 3.25026670

AFVL 5.70431624

AFVM 5.41925395

AFVN 3.50330656

AFVP 3.46127257

AFVQ 3.55242906

AFVR 3.51161956

AFVS 3.80802638

AFVT 4.07390844

AFVV 5.06219950

AFVW 5.98019647

AFVY 6.45618005

AFWA 3.55356388

AFWC 3.23354886

AFWD 2.26616048

AFWE 2.33149636

AFWF 5.99146135

AFWG 2.66141214

AFWH 3.35460168

AFWI 4.34524448

AFWK 2.38853438

AFWL 4.48883874

AFWM 4.29417960

AFWN 2.63257317

AFWP 2.54355603

AFWQ 2.77344662

AFWR 2.65374129

AFWS 2.93259222

AFWT 3.24859859

AFWV 4.06218350

AFWW 4.54386450

AFWY 4.85398177

AFYA 3.52228189

AFYC 3.56803733

AFYD 2.31735431

AFYE 2.50537678

AFYF 6.37240257

AFYG 2.68815839

AFYH 3.62572101

AFYI 4.54864681

AFYK 2.65130287

AFYL 4.67559771

AFYM 4.43203797

AFYN 2.86000155

AFYP 2.81105984

AFYQ 2.87737141

AFYR 2.69819934

AFYS 3.04109650

AFYT 3.28789445

AFYV 4.19717400

AFYW 4.83357382

AFYY 5.22692006

AGAA 6.67411349

AGAC 5.65459501

AGAD 6.31362958

AGAE 5.78205549

AGAF 4.53729292

AGAG 11.06018676

AGAH 5.56539147

AGAI 4.42846022

AGAK 5.76710580

AGAL 4.50301972

AGAM 4.87953829

AGAN 6.52944264

AGAP 5.75686604

AGAQ 5.67199010

AGAR 5.56154844

AGAS 6.54861453

AGAT 5.67917723

AGAV 4.80559890

AGAW 4.47633529

AGAY 4.66100374

AGCA 5.37046872

AGCC 1.88526858

AGCD 4.86598674

AGCE 4.50679033

AGCF 3.35166674

AGCG 7.63355025

AGCH 3.64664102

AGCI 3.30529434

AGCK 4.53111840

AGCL 3.36672630

AGCM 3.78795594

AGCN 5.06499165

AGCP 4.00174288

AGCQ 4.37481069

AGCR 4.26244701

AGCS 5.18542296

AGCT 4.45207752

AGCV 3.63773420

AGCW 3.06802507

AGCY 3.45643950

AGDA 4.53912645

AGDC 3.43325365

AGDD 4.49641122

AGDE 4.14368992

AGDF 2.84057259

AGDG 6.77018153

AGDH 3.80303436

AGDI 2.82590487

AGDK 4.06073371

AGDL 2.92829566

AGDM 3.18286550

AGDN 4.59138790

AGDP 4.02026843

AGDQ 4.05840193

AGDR 3.89056519

AGDS 4.48638115

AGDT 3.96587482

AGDV 3.15213449

AGDW 2.85383211

AGDY 3.01769148

AGEA 4.68161569

AGEC 3.65899042

AGED 4.72758062

AGEE 4.28504037

AGEF 2.99838576

AGEG 7.19078585

AGEH 4.01497344

AGEI 2.94886565

AGEK 4.21812169

AGEL 3.03057108

AGEM 3.29379687

AGEN 4.87795503

AGEP 4.20601022

AGEQ 4.16947179

AGER 3.98461186

AGES 4.66004082

AGET 4.08874724

AGEV 3.26015685

AGEW 2.89569489

AGEY 3.17691234

AGFA 4.32051700

AGFC 3.19342751

AGFD 3.99795070

AGFE 3.59935631

AGFF 2.67709480

AGFG 6.43261399

AGFH 3.29018156

AGFI 2.74858981

AGFK 3.63649820

AGFL 2.71687529

AGFM 2.96238627

AGFN 4.19132276

AGFP 3.55849966

AGFQ 3.55434423

AGFR 3.38183707

AGFS 4.21622828

AGFT 3.61032295

AGFV 2.94015470

AGFW 2.55640057

AGFY 2.74493335

AGGA 5.40404770

AGGC 4.06031904

AGGD 4.81264301

AGGE 4.50561931

AGGF 3.53740392

AGGG 7.71980164

AGGH 4.14944402

AGGI 3.49601665

AGGK 4.43639910

AGGL 3.58212733

AGGM 3.93826618

AGGN 5.04380973

AGGP 4.37198186

AGGQ 4.46682348

AGGR 4.26350281

AGGS 5.20826353

AGGT 4.50940150

AGGV 3.76898904

AGGW 3.39578601

AGGY 3.51757351

AGHA 4.29286253

AGHC 2.84637658

AGHD 4.22569538

AGHE 3.85235781

AGHF 2.63648606

AGHG 6.54493681

AGHH 3.43373273

AGHI 2.60143432

AGHK 3.82460263

AGHL 2.76478561

AGHM 2.95775946

AGHN 4.34023806

AGHP 3.74962517

AGHQ 3.81416848

AGHR 3.65626004

AGHS 4.25242322

AGHT 3.64669471

AGHV 2.86690565

AGHW 2.58646535

AGHY 2.77488801

AGIA 4.74560603

AGIC 3.85102356

AGID 4.47995809

AGIE 4.09292606

AGIF 3.13679258

AGIG 7.15819161

AGIH 3.83393618

AGII 3.00470224

AGIK 4.11251502

AGIL 3.05078269

AGIM 3.30271537

AGIN 4.70651389

AGIP 4.02882346

AGIQ 3.98247687

AGIR 3.83274384

AGIS 4.70719629

AGIT 4.02280328

AGIV 3.24241810

AGIW 3.05967500

AGIY 3.23770656

AGKA 4.49828033

AGKC 3.60052993

AGKD 4.60855409

AGKE 4.13278849

AGKF 2.96238594

AGKG 7.09212021

AGKH 3.89001625

AGKI 2.90111633

AGKK 4.20022722

AGKL 2.94161694

AGKM 3.17863897

AGKN 4.89256431

AGKP 4.02974916

AGKQ 4.00271797

AGKR 3.78547800

AGKS 4.54409623

AGKT 3.91752573

AGKV 3.11686293

AGKW 2.80998807

AGKY 3.17560144

AGLA 4.62455987

AGLC 3.68317163

AGLD 4.40952812

AGLE 3.97622815

AGLF 2.98213191

AGLG 6.97567400

AGLH 3.78346556

AGLI 2.93955194

AGLK 3.98338324

AGLL 2.94496343

AGLM 3.21354935

AGLN 4.57364971

AGLP 4.02041948

AGLQ 3.89705537

AGLR 3.80577913

AGLS 4.55765836

AGLT 3.95115010

AGLV 3.19833185

AGLW 2.92310059

AGLY 3.05815755

AGMA 4.70338713

AGMC 3.79526034

AGMD 4.42877185

AGME 4.02062239

AGMF 3.06379180

AGMG 7.11402543

AGMH 3.76763133

AGMI 2.93920394

AGMK 4.00713366

AGML 3.00362551

AGMM 3.22000512

AGMN 4.57390806

AGMP 4.05907171

AGMQ 3.89809209

AGMR 3.80701648

AGMS 4.60719797

AGMT 3.96039908

AGMV 3.21244219

AGMW 3.15100172

AGMY 3.16129439

AGNA 4.49642269

AGNC 3.54847018

AGND 4.46475011

AGNE 4.08252495

AGNF 2.93415619

AGNG 6.86364133

AGNH 3.79748662

AGNI 2.92770484

AGNK 4.13414861

AGNL 2.95255295

AGNM 3.23691381

AGNN 4.73927299

AGNP 3.97890682

AGNQ 4.02077598

AGNR 3.77918696

AGNS 4.54604903

AGNT 3.93176602

AGNV 3.15460914

AGNW 2.83684714

AGNY 3.14667403

AGPA 4.93744076

AGPC 3.61004674

AGPD 4.71309964

AGPE 4.33602323

AGPF 3.10701697

AGPG 7.41301468

AGPH 4.00824855

AGPI 3.01118485

AGPK 4.18335081

AGPL 3.15592766

AGPM 3.45626149

AGPN 4.85364630

AGPP 4.11335617

AGPQ 4.22948151

AGPR 4.04892172

AGPS 4.81833669

AGPT 4.21293674

AGPV 3.37581704

AGPW 3.03198935

AGPY 3.26026558

AGQA 4.61761523

AGQC 3.58889192

AGQD 4.61446160

AGQE 4.16567670

AGQF 3.00345205

AGQG 7.09627598

AGQH 3.95730635

AGQI 2.92969791

AGQK 4.12925021

AGQL 2.99857705

AGQM 3.23858388

AGQN 4.75263373

AGQP 4.18150223

AGQQ 4.12180343

AGQR 3.93860974

AGQS 4.58268933

AGQT 4.00335240

AGQV 3.19452512

AGQW 2.94830336

AGQY 3.14803235

AGRA 4.50690198

AGRC 3.47528786

AGRD 4.44758484

AGRE 4.02406579

AGRF 2.79575316

AGRG 6.86533581

AGRH 3.81579175

AGRI 2.73746807

AGRK 3.89541704

AGRL 2.87169977

AGRM 3.07331566

AGRN 4.51203051

AGRP 4.01984059

AGRQ 3.90672315

AGRR 3.83953761

AGRS 4.41448747

AGRT 3.84315673

AGRV 3.08381359

AGRW 2.83806989

AGRY 2.94651164

AGSA 5.44639693

AGSC 4.46469985

AGSD 5.10696924

AGSE 4.67129407

AGSF 3.66279758

AGSG 8.12266593

AGSH 4.38970115

AGSI 3.58845065

AGSK 4.65939921

AGSL 3.64159204

AGSM 3.94501075

AGSN 5.33136645

AGSP 4.65205909

AGSQ 4.55908044

AGSR 4.43848665

AGSS 5.32586039

AGST 4.61301443

AGSV 3.85338097

AGSW 3.63983403

AGSY 3.76672475

AGTA 5.07422063

AGTC 4.09487337

AGTD 4.80316624

AGTE 4.35354487

AGTF 3.34308188

AGTG 7.50877129

AGTH 4.08527236

AGTI 3.25942038

AGTK 4.31039988

AGTL 3.34644296

AGTM 3.63141101

AGTN 4.95323372

AGTP 4.35613052

AGTQ 4.25339288

AGTR 4.14878722

AGTS 4.96223192

AGTT 4.28265719

AGTV 3.54232326

AGTW 3.27377568

AGTY 3.41298262

AGVA 5.16601277

AGVC 4.14649492

AGVD 4.87339233

AGVE 4.42633758

AGVF 3.30345562

AGVG 7.72484301

AGVH 4.12418584

AGVI 3.19644266

AGVK 4.38654740

AGVL 3.31123248

AGVM 3.60594504

AGVN 5.01016795

AGVP 4.37880356

AGVQ 4.31246924

AGVR 4.21379846

AGVS 5.04357092

AGVT 4.33525659

AGVV 3.50022503

AGVW 3.27459982

AGVY 3.41693453

AGWA 3.96727613

AGWC 2.63343658

AGWD 3.74228379

AGWE 3.33697791

AGWF 2.44794384

AGWG 5.94906580

AGWH 3.03767671

AGWI 2.47432510

AGWK 3.38183094

AGWL 2.49008018

AGWM 2.76914644

AGWN 3.77235582

AGWP 3.32876055

AGWQ 3.34462877

AGWR 3.21743067

AGWS 3.89896837

AGWT 3.39770840

AGWV 2.69932160

AGWW 2.16941236

AGWY 2.43675190

AGYA 4.17379964

AGYC 3.14849729

AGYD 4.00848162

AGYE 3.62474377

AGYF 2.60190163

AGYG 6.34357862

AGYH 3.35685256

AGYI 2.59097744

AGYK 3.69036935

AGYL 2.59585453

AGYM 2.92299978

AGYN 4.24983561

AGYP 3.58390236

AGYQ 3.57571911

AGYR 3.34527829

AGYS 4.13597550

AGYT 3.54071093

AGYV 2.79258431

AGYW 2.48922128

AGYY 2.75095237

AHAA 5.69104367

AHAC 5.54874144

AHAD 6.18551455

AHAE 6.06319832

AHAF 6.06763184

AHAG 5.56539147

AHAH 11.30595974

AHAI 5.10312347

AHAK 6.25686804

AHAL 5.39036454

AHAM 5.48219063

AHAN 6.79410879

AHAP 5.42651533

AHAQ 6.72952393

AHAR 6.52959944

AHAS 6.01784609

AHAT 5.81335981

AHAV 5.17238907

AHAW 6.00907935

AHAY 7.17961060

AHCA 4.50044847

AHCC 1.79694616

AHCD 4.86194385

AHCE 4.94252053

AHCF 4.77826883

AHCG 3.90634330

AHCH 7.67969976

AHCI 4.01885171

AHCK 5.04215067

AHCL 4.23072625

AHCM 4.39277768

AHCN 5.44009848

AHCP 3.80115101

AHCQ 5.38065712

AHCR 5.14789956

AHCS 4.82387246

AHCT 4.70340302

AHCV 4.06552475

AHCW 4.30947973

AHCY 5.73961141

AHDA 4.08466692

AHDC 3.69159371

AHDD 4.24737296

AHDE 4.23355048

AHDF 4.12084676

AHDG 3.70357388

AHDH 6.80283360

AHDI 3.53019039

AHDK 4.35771274

AHDL 3.69167588

AHDM 3.75285807

AHDN 4.66944538

AHDP 3.65277897

AHDQ 4.58857850

AHDR 4.55670193

AHDS 4.20243502

AHDT 4.07679588

AHDV 3.61151506

AHDW 4.08400276

AHDY 4.92290815

AHEA 4.32441330

AHEC 3.99801277

AHED 4.55739537

AHEE 4.48402457

AHEF 4.43094531

AHEG 4.10702246

AHEH 7.09118535

AHEI 3.80420402

AHEK 4.62206197

AHEL 3.93580712

AHEM 4.01741376

AHEN 5.00687140

AHEP 3.96128502

AHEQ 4.81687683

AHER 4.79497191

AHES 4.46696781

AHET 4.34718822

AHEV 3.84848506

AHEW 4.32705315

AHEY 5.26768657

AHFA 3.42915231

AHFC 3.07699899

AHFD 3.85816104

AHFE 3.83207241

AHFF 3.51598099

AHFG 3.25234738

AHFH 6.51795672

AHFI 2.98334408

AHFK 3.99283453

AHFL 3.08537470

AHFM 3.25273221

AHFN 4.29183760

AHFP 3.16852757

AHFQ 4.19909331

AHFR 4.05559344

AHFS 3.79077154

AHFT 3.59225273

AHFV 2.96475186

AHFW 3.53026692

AHFY 4.41425995

AHGA 4.32468704

AHGC 3.80526291

AHGD 4.57468288

AHGE 4.64165320

AHGF 4.52109313

AHGG 3.87103089

AHGH 8.10296171

AHGI 3.83924818

AHGK 4.73338331

AHGL 4.06253159

AHGM 4.23165537

AHGN 5.12162112

AHGP 3.97761141

AHGQ 5.22857815

AHGR 4.95749659

AHGS 4.57534565

AHGT 4.42666866

AHGV 3.87732507

AHGW 4.44766592

AHGY 5.43236936

AHHA 3.91510473

AHHC 3.12212515

AHHD 4.21433885

AHHE 4.16380799

AHHF 3.97931025

AHHG 3.62208927

AHHH 6.42321831

AHHI 3.44956886

AHHK 4.22731481

AHHL 3.60896012

AHHM 3.62504923

AHHN 4.51239241

AHHP 3.55135836

AHHQ 4.55201108

AHHR 4.42803521

AHHS 4.09647751

AHHT 3.94616991

AHHV 3.48508175

AHHW 3.87409691

AHHY 4.74348102

AHIA 3.84625667

AHIC 3.68392509

AHID 4.37431752

AHIE 4.34710577

AHIF 4.06610152

AHIG 3.71488896

AHIH 7.17586599

AHII 3.37340261

AHIK 4.47732209

AHIL 3.54519539

AHIM 3.69951037

AHIN 4.82608664

AHIP 3.70370036

AHIQ 4.73812275

AHIR 4.57098767

AHIS 4.25898167

AHIT 4.05659379

AHIV 3.37014675

AHIW 4.07562029

AHIY 5.06256827

AHKA 4.06912638

AHKC 3.92743160

AHKD 4.41762938

AHKE 4.36348155

AHKF 4.33309993

AHKG 3.93387124

AHKH 6.93199230

AHKI 3.70691249

AHKK 4.47878672

AHKL 3.77828925

AHKM 3.84663377

AHKN 4.91038482

AHKP 3.76435108

AHKQ 4.62702126

AHKR 4.53457949

AHKS 4.33294173

AHKT 4.17872810

AHKV 3.68010833

AHKW 4.14993550

AHKY 5.20318095

AHLA 3.86741094

AHLC 3.66145993

AHLD 4.26346826

AHLE 4.20896795

AHLF 3.95410213

AHLG 3.71471695

AHLH 6.95017495

AHLI 3.29609655

AHLK 4.35780087

AHLL 3.44239546

AHLM 3.63576695

AHLN 4.66160260

AHLP 3.64591371

AHLQ 4.59028574

AHLR 4.49789478

AHLS 4.15252413

AHLT 4.01332611

AHLV 3.35843271

AHLW 3.98408456

AHLY 4.89359908

AHMA 3.89909620

AHMC 3.88852214

AHMD 4.36793784

AHME 4.30452123

AHMF 4.09618246

AHMG 3.80249286

AHMH 7.18372674

AHMI 3.42769476

AHMK 4.42248647

AHML 3.60066596

AHMM 3.64601180

AHMN 4.81668538

AHMP 3.76989929

AHMQ 4.69100725

AHMR 4.58768805

AHMS 4.21240748

AHMT 4.02616198

AHMV 3.47707294

AHMW 4.12668927

AHMY 5.05329631

AHNA 3.89593416

AHNC 3.68380663

AHND 4.23298458

AHNE 4.24749050

AHNF 4.14916840

AHNG 3.66859012

AHNH 6.90437364

AHNI 3.54812933

AHNK 4.38152963

AHNL 3.63742157

AHNM 3.71148225

AHNN 4.70673691

AHNP 3.62544171

AHNQ 4.61910223

AHNR 4.43677704

AHNS 4.18427634

AHNT 4.03670204

AHNV 3.51313633

AHNW 3.96508663

AHNY 4.98769951

AHPA 4.20273699

AHPC 3.66092437

AHPD 4.49519049

AHPE 4.43544165

AHPF 4.30799135

AHPG 3.85423483

AHPH 7.59037445

AHPI 3.64740013

AHPK 4.51300558

AHPL 3.83285131

AHPM 4.02970044

AHPN 4.92511990

AHPP 3.85655303

AHPQ 4.92514114

AHPR 4.75853999

AHPS 4.42174467

AHPT 4.30426074

AHPV 3.74007777

AHPW 4.17299252

AHPY 5.19358416

AHQA 4.19946285

AHQC 3.93144238

AHQD 4.48717106

AHQE 4.40502680

AHQF 4.35966697

AHQG 4.02283886

AHQH 6.99354108

AHQI 3.66942985

AHQK 4.51380841

AHQL 3.84440130

AHQM 3.87889880

AHQN 4.85953353

AHQP 3.85842588

AHQQ 4.75418064

AHQR 4.68040952

AHQS 4.34934432

AHQT 4.20937743

AHQV 3.70518068

AHQW 4.26813374

AHQY 5.17853279

AHRA 4.18784121

AHRC 3.77010060

AHRD 4.39425474

AHRE 4.31324780

AHRF 4.17846075

AHRG 3.90829350

AHRH 6.81437659

AHRI 3.52891411

AHRK 4.27047436

AHRL 3.74256915

AHRM 3.82107261

AHRN 4.67878962

AHRP 3.72509466

AHRQ 4.59808244

AHRR 4.54997332

AHRS 4.26317746

AHRT 4.13138630

AHRV 3.65645994

AHRW 4.13851979

AHRY 4.93467163

AHSA 4.60250967

AHSC 4.52202544

AHSD 4.98420626

AHSE 4.93668888

AHSF 4.97811460

AHSG 4.40820069

AHSH 8.27950603

AHSI 4.17647714

AHSK 5.08047417

AHSL 4.38665388

AHSM 4.47217310

AHSN 5.54179403

AHSP 4.31462917

AHSQ 5.45886805

AHSR 5.26960724

AHSS 4.89378254

AHST 4.70802264

AHSV 4.19685546

AHSW 4.83689838

AHSY 5.92260256

AHTA 4.26868568

AHTC 4.12532325

AHTD 4.60924319

AHTE 4.56041523

AHTF 4.53078737

AHTG 4.06202152

AHTH 7.61725687

AHTI 3.79888391

AHTK 4.69554897

AHTL 4.00682030

AHTM 4.12883496

AHTN 5.07595305

AHTP 3.99747451

AHTQ 5.06110608

AHTR 4.90670564

AHTS 4.53545022

AHTT 4.35420892

AHTV 3.84398722

AHTW 4.45475344

AHTY 5.43653821

AHVA 4.22991645

AHVC 3.99111912

AHVD 4.72929967

AHVE 4.66491922

AHVF 4.45310385

AHVG 4.06709226

AHVH 7.78467879

AHVI 3.68155540

AHVK 4.81109807

AHVL 3.87737765

AHVM 4.02530268

AHVN 5.17760929

AHVP 4.05440222

AHVQ 5.14265173

AHVR 4.99016829

AHVS 4.59383872

AHVT 4.41346290

AHVV 3.69577429

AHVW 4.42811895

AHVY 5.43350313

AHWA 3.35550090

AHWC 2.58403316

AHWD 3.59977096

AHWE 3.64258721

AHWF 3.21902936

AHWG 3.04128024

AHWH 6.24110344

AHWI 2.79003394

AHWK 3.67982629

AHWL 2.89343392

AHWM 3.18369011

AHWN 4.01336323

AHWP 2.88148018

AHWQ 3.93739629

AHWR 3.83122588

AHWS 3.53492058

AHWT 3.43415856

AHWV 2.80664368

AHWW 3.41956921

AHWY 3.94512614

AHYA 3.49618778

AHYC 3.15798755

AHYD 3.83913304

AHYE 3.86920747

AHYF 3.66128923

AHYG 3.30605298

AHYH 6.38018363

AHYI 3.10150772

AHYK 4.03270680

AHYL 3.20572541

AHYM 3.40559000

AHYN 4.34173038

AHYP 3.17061552

AHYQ 4.21876673

AHYR 4.02007145

AHYS 3.81726554

AHYT 3.63225540

AHYV 3.10262605

AHYW 3.51306683

AHYY 4.43851454

AIAA 6.06988657

AIAC 6.40986688

AIAD 4.18334831

AIAE 4.80437455

AIAF 6.78574488

AIAG 4.42846022

AIAH 5.10312347

AIAI 10.59751809

AIAK 5.11162847

AIAL 7.86796839

AIAM 7.47333954

AIAN 4.79399623

AIAP 5.01013048

AIAQ 5.12258233

AIAR 5.10133696

AIAS 5.17979545

AIAT 6.13485728

AIAV 8.34552498

AIAW 5.68706190

AIAY 5.93711951

AICA 4.98917051

AICC 2.59221223

AICD 2.99821426

AICE 3.66855916

AICF 5.43998787

AICG 2.96883256

AICH 3.29763123

AICI 7.66965186

AICK 3.87626415

AICL 6.44539982

AICM 6.10430678

AICN 3.52885577

AICP 3.48007312

AICQ 3.91842265

AICR 3.87836658

AICS 4.01101762

AICT 4.88259046

AICV 6.80420938

AICW 4.14747946

AICY 4.69739289

AIDA 3.91765754

AIDC 3.94927698

AIDD 2.43902924

AIDE 2.88298346

AIDF 4.49491429

AIDG 2.38135597

AIDH 3.08813031

AIDI 6.41778213

AIDK 3.09817326

AIDL 5.33172852

AIDM 4.91300843

AIDN 2.79228610

AIDP 2.99688578

AIDQ 3.15214879

AIDR 3.16760561

AIDS 3.12508601

AIDT 3.83981223

AIDV 5.60007162

AIDW 3.60092039

AIDY 3.84384625

AIEA 4.18352553

AIEC 4.32997049

AIED 2.68648320

AIEE 3.14362753

AIEF 4.76713678

AIEG 2.73870990

AIEH 3.35228034

AIEI 6.74308614

AIEK 3.34006451

AIEL 5.60595158

AIEM 5.19633405

AIEN 3.10761083

AIEP 3.25056640

AIEQ 3.38888374

AIER 3.39596214

AIES 3.39169335

AIET 4.12096952

AIEV 5.89630519

AIEW 3.86737615

AIEY 4.07843389

AIFA 4.04858273

AIFC 3.96791517

AIFD 2.31330817

AIFE 2.74434844

AIFF 4.51886746

AIFG 2.79943665

AIFH 2.99695447

AIFI 6.07723815

AIFK 2.99057212

AIFL 5.17973678

AIFM 4.82820297

AIFN 2.79279302

AIFP 2.97210422

AIFQ 3.06139670

AIFR 2.94952844

AIFS 3.23112448

AIFT 3.87850515

AIFV 5.43256528

AIFW 3.59875584

AIFY 3.86087416

AIGA 4.77943330

AIGC 4.48975989

AIGD 2.77379298

AIGE 3.38520537

AIGF 5.37374765

AIGG 3.01339768

AIGH 3.66577045

AIGI 7.37178491

AIGK 3.60118505

AIGL 6.24036155

AIGM 5.86728719

AIGN 3.38975520

AIGP 3.55322280

AIGQ 3.79932234

AIGR 3.62286806

AIGS 3.86391194

AIGT 4.61624195

AIGV 6.49882374

AIGW 4.26631279

AIGY 4.54478779

AIHA 3.81077270

AIHC 3.41154485

AIHD 2.31814625

AIHE 2.80666084

AIHF 4.34846690

AIHG 2.35990506

AIHH 2.75212271

AIHI 6.21261136

AIHK 2.97457404

AIHL 5.20394739

AIHM 4.72770172

AIHN 2.64912604

AIHP 2.87413665

AIHQ 3.03362297

AIHR 2.99086025

AIHS 3.04710836

AIHT 3.73895216

AIHV 5.41234700

AIHW 3.51237703

AIHY 3.66677023

AIIA 4.56607324

AIIC 4.63586569

AIID 2.78925702

AIIE 3.33484047

AIIF 5.14843876

AIIG 3.17919379

AIIH 3.54314375

AIII 6.74111328

AIIK 3.53016693

AIIL 5.84121823

AIIM 5.43269472

AIIN 3.30632475

AIIP 3.48248481

AIIQ 3.57895369

AIIR 3.52437652

AIIS 3.75606546

AIIT 4.40976376

AIIV 6.05858228

AIIW 4.19890656

AIIY 4.42399249

AIKA 4.05919468

AIKC 4.21644950

AIKD 2.57213619

AIKE 3.03180382

AIKF 4.74295323

AIKG 2.61954784

AIKH 3.23937635

AIKI 6.59846904

AIKK 3.24198477

AIKL 5.49757453

AIKM 5.03880243

AIKN 3.01639325

AIKP 3.17384101

AIKQ 3.21768336

AIKR 3.20108004

AIKS 3.28066022

AIKT 3.97461996

AIKV 5.72550288

AIKW 3.71544864

AIKY 4.06943151

AILA 4.43984285

AILC 4.46731220

AILD 2.64395763

AILE 3.10170432

AILF 4.92678387

AILG 3.07664669

AILH 3.43026694

AILI 6.52013498

AILK 3.33547918

AILL 5.60415670

AILM 5.23385799

AILN 3.12156319

AILP 3.31873225

AILQ 3.39643946

AILR 3.35792389

AILS 3.56763430

AILT 4.23911541

AILV 5.88963049

AILW 4.05614171

AILY 4.20333645

AIMA 4.45438695

AIMC 4.51740357

AIMD 2.71671135

AIME 3.17234601

AIMF 5.00619343

AIMG 3.14410283

AIMH 3.45029971

AIMI 6.68072765

AIMK 3.37563993

AIML 5.72398396

AIMM 5.27645750

AIMN 3.18986863

AIMP 3.41185507

AIMQ 3.41493910

AIMR 3.39771591

AIMS 3.57407479

AIMT 4.27126938

AIMV 5.95921420

AIMW 4.16032941

AIMY 4.30089326

AINA 3.93160573

AINC 4.01212311

AIND 2.49508368

AINE 2.93195079

AINF 4.59494431

AING 2.56790229

AINH 3.13505820

AINI 6.44979445

AINK 3.16740489

AINL 5.37475798

AINM 4.96806206

AINN 2.95366145

AINP 3.03855233

AINQ 3.19313776

AINR 3.10741571

AINS 3.25378653

AINT 3.90564056

AINV 5.58861036

AINW 3.63075264

AINY 3.93275408

AIPA 4.22429990

AIPC 4.02831356

AIPD 2.64164601

AIPE 3.09754614

AIPF 4.80751096

AIPG 2.64997060

AIPH 3.34231135

AIPI 6.86210474

AIPK 3.29105851

AIPL 5.69246779

AIPM 5.27588305

AIPN 3.06580243

AIPP 3.30156810

AIPQ 3.43884086

AIPR 3.38056862

AIPS 3.43855847

AIPT 4.20950120

AIPV 6.01138573

AIPW 3.82020577

AIPY 4.09033910

AIQA 4.12226416

AIQC 4.19726479

AIQD 2.64177733

AIQE 3.03942767

AIQF 4.75133382

AIQG 2.76520935

AIQH 3.29245266

AIQI 6.63339348

AIQK 3.23610478

AIQL 5.55459944

AIQM 5.10885765

AIQN 3.01881310

AIQP 3.24434376

AIQQ 3.30942266

AIQR 3.30824286

AIQS 3.33400703

AIQT 4.06984585

AIQV 5.79472823

AIQW 3.89975013

AIQY 4.04250118

AIRA 4.02235761

AIRC 4.08011966

AIRD 2.48517276

AIRE 2.94573290

AIRF 4.52671458

AIRG 2.55696139

AIRH 3.16809708

AIRI 6.38151335

AIRK 2.99242375

AIRL 5.34917945

AIRM 4.90677207

AIRN 2.81347125

AIRP 3.08596826

AIRQ 3.13823643

AIRR 3.13255972

AIRS 3.17428905

AIRT 3.88389477

AIRV 5.63710290

AIRW 3.69163546

AIRY 3.83791103

AISA 4.82945994

AISC 4.99545064

AISD 3.10855538

AISE 3.58673391

AISF 5.53417996

AISG 3.35500117

AISH 3.91308256

AISI 7.69409426

AISK 3.83241706

AISL 6.40624417

AISM 6.01292400

AISN 3.68317980

AISP 3.83047932

AISQ 3.93704003

AISR 3.85144831

AISS 4.00261543

AIST 4.79847027

AISV 6.68757244

AISW 4.50306448

AISY 4.74831440

AITA 4.55332884

AITC 4.66604806

AITD 2.84673630

AITE 3.28729665

AITF 5.19942372

AITG 3.11374405

AITH 3.64118500

AITI 7.17160688

AITK 3.52768513

AITL 6.07012263

AITM 5.63783206

AITN 3.35309655

AITP 3.55529158

AITQ 3.63235267

AITR 3.58906368

AITS 3.74266773

AITT 4.43929124

AITV 6.28062608

AITW 4.24379456

AITY 4.40485445

AIVA 4.86445303

AIVC 4.91054173

AIVD 3.03615597

AIVE 3.56241989

AIVF 5.44116790

AIVG 3.35351769

AIVH 3.81182444

AIVI 7.31663960

AIVK 3.79201817

AIVL 6.26076587

AIVM 5.86806821

AIVN 3.53123694

AIVP 3.75007290

AIVQ 3.90209754

AIVR 3.81238362

AIVS 3.96190939

AIVT 4.71132356

AIVV 6.53979877

AIVW 4.46218692

AIVY 4.65374327

AIWA 3.73423561

AIWC 3.25626061

AIWD 1.99953899

AIWE 2.47351241

AIWF 4.00647592

AIWG 2.40986223

AIWH 2.76433655

AIWI 5.63124099

AIWK 2.64562955

AIWL 4.77424279

AIWM 4.46087154

AIWN 2.45078409

AIWP 2.62707333

AIWQ 2.85645241

AIWR 2.69083015

AIWS 2.90064548

AIWT 3.54019100

AIWV 5.00389109

AIWW 3.14288544

AIWY 3.33729777

AIYA 3.84557601

AIYC 3.75656755

AIYD 2.31763271

AIYE 2.74330053

AIYF 4.37452700

AIYG 2.58729115

AIYH 2.94172732

AIYI 6.03593056

AIYK 2.96835874

AIYL 5.06234130

AIYM 4.71887685

AIYN 2.73655647

AIYP 2.87976756

AIYQ 3.00119341

AIYR 2.89425968

AIYS 3.07767796

AIYT 3.71297599

AIYV 5.32015284

AIYW 3.33952133

AIYY 3.70509830

AKAA 6.04952156

AKAC 4.88338764

AKAD 6.25287615

AKAE 6.78784988

AKAF 4.39790659

AKAG 5.76710580

AKAH 6.25686804

AKAI 5.11162847

AKAK 10.34826611

AKAL 5.15034616

AKAM 5.52021931

AKAN 6.54163189

AKAP 5.84033960

AKAQ 7.00223314

AKAR 7.70329077

AKAS 6.23940732

AKAT 6.14878164

AKAV 5.16362579

AKAW 4.66443464

AKAY 5.11723863

AKCA 4.56375451

AKCC 1.07378983

AKCD 4.83441779

AKCE 5.37478148

AKCF 3.12962468

AKCG 3.96232040

AKCH 4.37292735

AKCI 3.81362330

AKCK 7.21345341

AKCL 3.86423940

AKCM 4.21171558

AKCN 5.00458374

AKCP 4.06490468

AKCQ 5.47945194

AKCR 6.10898963

AKCS 4.84093108

AKCT 4.76223475

AKCV 3.87146897

AKCW 3.15123195

AKCY 3.85415700

AKDA 4.45874787

AKDC 3.10924352

AKDD 4.45786486

AKDE 4.88379962

AKDF 2.89401189

AKDG 3.89821967

AKDH 4.30808973

AKDI 3.51357002

AKDK 6.24933571

AKDL 3.53487699

AKDM 3.81995669

AKDN 4.64188821

AKDP 4.03202040

AKDQ 5.04314199

AKDR 5.37712010

AKDS 4.48669435

AKDT 4.43398165

AKDV 3.60015576

AKDW 2.97895638

AKDY 3.43584841

AKEA 4.78736660

AKEC 3.45517071

AKED 4.81949510

AKEE 5.24946761

AKEF 3.19741579

AKEG 4.36375000

AKEH 4.65749461

AKEI 3.82148026

AKEK 6.58067165

AKEL 3.87936883

AKEM 4.16726986

AKEN 5.06061526

AKEP 4.36422257

AKEQ 5.37281125

AKER 5.73332420

AKES 4.84211510

AKET 4.74880665

AKEV 3.91219865

AKEW 3.22668628

AKEY 3.73971751

AKFA 3.49658310

AKFC 2.30091053

AKFD 3.90618601

AKFE 4.26738809

AKFF 2.16747964

AKFG 3.27185966

AKFH 3.66160232

AKFI 2.70839211

AKFK 5.71129773

AKFL 2.71508704

AKFM 2.99210903

AKFN 4.08862715

AKFP 3.42807766

AKFQ 4.40624276

AKFR 4.78987705

AKFS 3.84076494

AKFT 3.69981531

AKFV 2.74316977

AKFW 2.41248300

AKFY 2.80781510

AKGA 4.49267229

AKGC 3.06928253

AKGD 4.57979336

AKGE 5.12418774

AKGF 3.05548914

AKGG 3.95421763

AKGH 4.56637688

AKGI 3.63147757

AKGK 7.07396927

AKGL 3.71059439

AKGM 4.04995838

AKGN 4.88279687

AKGP 4.23141687

AKGQ 5.34914882

AKGR 5.86078241

AKGS 4.68537065

AKGT 4.60988645

AKGV 3.72849272

AKGW 3.19371021

AKGY 3.64770312

AKHA 4.02349478

AKHC 2.40718932

AKHD 4.16755946

AKHE 4.58180529

AKHF 2.56471828

AKHG 3.61374538

AKHH 3.88243872

AKHI 3.23246553

AKHK 6.02988545

AKHL 3.22907073

AKHM 3.41972886

AKHN 4.32853167

AKHP 3.81560198

AKHQ 4.76334764

AKHR 5.14560801

AKHS 4.18988239

AKHT 4.05108622

AKHV 3.21760490

AKHW 2.71962902

AKHY 3.14210343

AKIA 4.04956475

AKIC 2.98637134

AKID 4.53790304

AKIE 4.96709537

AKIF 2.80812464

AKIG 3.85008710

AKIH 4.36834739

AKII 3.22258059

AKIK 6.41296496

AKIL 3.29740858

AKIM 3.64913164

AKIN 4.75350293

AKIP 4.03505819

AKIQ 5.03995666

AKIR 5.47222485

AKIS 4.44975992

AKIT 4.27896590

AKIV 3.23583449

AKIW 3.03875636

AKIY 3.50968920

AKKA 4.76369637

AKKC 3.46028241

AKKD 4.92177324

AKKE 5.32369075

AKKF 3.29704252

AKKG 4.42873958

AKKH 4.72938854

AKKI 3.88715005

AKKK 6.50147631

AKKL 3.94671655

AKKM 4.19477368

AKKN 5.11886580

AKKP 4.42563350

AKKQ 5.36862370

AKKR 5.65230967

AKKS 4.88017557

AKKT 4.76438553

AKKV 3.93050601

AKKW 3.29749614

AKKY 3.83376229

AKLA 4.02344808

AKLC 2.87338940

AKLD 4.38771786

AKLE 4.76864270

AKLF 2.61906200

AKLG 3.81248928

AKLH 4.23613568

AKLI 3.09849433

AKLK 6.23827136

AKLL 3.10117893

AKLM 3.48205898

AKLN 4.55341223

AKLP 3.94991945

AKLQ 4.89310209

AKLR 5.31796001

AKLS 4.33033966

AKLT 4.17357545

AKLV 3.16437693

AKLW 2.84785224

AKLY 3.28875659

AKMA 4.09968421

AKMC 3.07307700

AKMD 4.50672828

AKME 4.89167057

AKMF 2.73079186

AKMG 3.91271735

AKMH 4.31854571

AKMI 3.24131634

AKMK 6.43435072

AKML 3.28786512

AKMM 3.60430210

AKMN 4.62973987

AKMP 4.02099754

AKMQ 4.96308244

AKMR 5.50190952

AKMS 4.35996812

AKMT 4.21298206

AKMV 3.31230978

AKMW 3.13508717

AKMY 3.44760371

AKNA 4.23158021

AKNC 3.08512387

AKND 4.47883128

AKNE 4.87412248

AKNF 2.96874828

AKNG 3.94022187

AKNH 4.32775485

AKNI 3.56148970

AKNK 6.32425248

AKNL 3.57234248

AKNM 3.80105971

AKNN 4.73515029

AKNP 4.04755076

AKNQ 5.01538388

AKNR 5.39263627

AKNS 4.51680587

AKNT 4.39500258

AKNV 3.58200164

AKNW 2.99031173

AKNY 3.53599709

AKPA 4.45474626

AKPC 2.93401590

AKPD 4.52301189

AKPE 4.92880846

AKPF 2.91713209

AKPG 3.95895247

AKPH 4.38210659

AKPI 3.51425535

AKPK 6.74717160

AKPL 3.55138612

AKPM 3.86410320

AKPN 4.70877803

AKPP 4.17235033

AKPQ 5.13795514

AKPR 5.59757244

AKPS 4.57331281

AKPT 4.52488485

AKPV 3.68751517

AKPW 3.01615974

AKPY 3.45795847

AKQA 4.63447938

AKQC 3.30471580

AKQD 4.77462512

AKQE 5.12362246

AKQF 3.10265206

AKQG 4.25484030

AKQH 4.64122073

AKQI 3.69983787

AKQK 6.51627591

AKQL 3.73975387

AKQM 4.01188665

AKQN 4.88039180

AKQP 4.31647972

AKQQ 5.28139214

AKQR 5.69180186

AKQS 4.69407025

AKQT 4.60972647

AKQV 3.75341988

AKQW 3.23760207

AKQY 3.62798378

AKRA 4.45885682

AKRC 3.16702071

AKRD 4.50708484

AKRE 4.91442905

AKRF 2.87745986

AKRG 4.05116854

AKRH 4.37412862

AKRI 3.43897544

AKRK 6.17236581

AKRL 3.52169272

AKRM 3.78231580

AKRN 4.60683652

AKRP 4.08519994

AKRQ 5.01540336

AKRR 5.37170652

AKRS 4.47569355

AKRT 4.36618703

AKRV 3.57518561

AKRW 2.99859299

AKRY 3.36590485

AKSA 4.88138774

AKSC 3.79914572

AKSD 5.07798280

AKSE 5.49884712

AKSF 3.50900389

AKSG 4.61470678

AKSH 5.04928535

AKSI 4.10686308

AKSK 7.45951827

AKSL 4.14625176

AKSM 4.45083536

AKSN 5.38803608

AKSP 4.72753439

AKSQ 5.71413297

AKSR 6.28730377

AKSS 5.11540606

AKST 5.01433834

AKSV 4.16983137

AKSW 3.67265515

AKSY 4.12051318

AKTA 4.54527330

AKTC 3.38665277

AKTD 4.74952117

AKTE 5.15573037

AKTF 3.13475941

AKTG 4.24311420

AKTH 4.68422785

AKTI 3.68808159

AKTK 6.92224118

AKTL 3.73850686

AKTM 3.99399588

AKTN 5.00444744

AKTP 4.38129191

AKTQ 5.34197201

AKTR 5.86991388

AKTS 4.76226626

AKTT 4.66160980

AKTV 3.76561962

AKTW 3.29336384

AKTY 3.72766366

AKVA 4.45246699

AKVC 3.25144770

AKVD 4.83841589

AKVE 5.26804620

AKVF 3.03562270

AKVG 4.21330270

AKVH 4.71640056

AKVI 3.49515016

AKVK 7.01354425

AKVL 3.58945571

AKVM 3.96235016

AKVN 5.02746652

AKVP 4.40657146

AKVQ 5.39475175

AKVR 5.91688393

AKVS 4.76422329

AKVT 4.64032567

AKVV 3.59944721

AKVW 3.27818572

AKVY 3.71008969

AKWA 3.27386074

AKWC 1.83468730

AKWD 3.54088073

AKWE 3.92077730

AKWF 1.83125828

AKWG 2.96935420

AKWH 3.35514696

AKWI 2.41720409

AKWK 5.32920821

AKWL 2.45137069

AKWM 2.79359397

AKWN 3.64480026

AKWP 3.06751497

AKWQ 4.08495694

AKWR 4.41699662

AKWS 3.48240339

AKWT 3.42836844

AKWV 2.47160355

AKWW 2.01779127

AKWY 2.41042709

AKYA 3.61944252

AKYC 2.48612045

AKYD 3.96717113

AKYE 4.36937882

AKYF 2.36758083

AKYG 3.38036464

AKYH 3.80882048

AKYI 2.95492343

AKYK 5.74041363

AKYL 2.94386873

AKYM 3.24446513

AKYN 4.19454776

AKYP 3.52015275

AKYQ 4.51175782

AKYR 4.81645454

AKYS 3.92616457

AKYT 3.80402660

AKYV 2.94745217

AKYW 2.55700485

AKYY 2.94828500

ALAA 5.94424132

ALAC 6.25844190

ALAD 4.32338098

ALAE 4.87745912

ALAF 7.18834356

ALAG 4.50301972

ALAH 5.39036454

ALAI 7.86796839

ALAK 5.15034616

ALAL 10.10844856

ALAM 7.72284535

ALAN 4.77165091

ALAP 4.94481818

ALAQ 5.41436545

ALAR 5.33412090

ALAS 5.08722198

ALAT 5.73155271

ALAV 7.25503747

ALAW 6.07021950

ALAY 6.19787081

ALCA 4.85741026

ALCC 2.39474151

ALCD 3.08506194

ALCE 3.73783817

ALCF 5.69424889

ALCG 3.00393050

ALCH 3.49353127

ALCI 6.41226582

ALCK 3.93192482

ALCL 7.21503937

ALCM 6.24390243

ALCN 3.51519448

ALCP 3.35316114

ALCQ 4.14392005

ALCR 4.00323212

ALCS 3.90039283

ALCT 4.54772471

ALCV 5.88586525

ALCW 4.31954653

ALCY 4.82610291

ALDA 3.99598354

ALDC 3.95201649

ALDD 2.67671217

ALDE 3.07639648

ALDF 4.85112520

ALDG 2.56241152

ALDH 3.36929188

ALDI 5.44371104

ALDK 3.26745985

ALDL 6.19134188

ALDM 5.20579259

ALDN 2.93602214

ALDP 3.09444741

ALDQ 3.45225831

ALDR 3.46320192

ALDS 3.18978567

ALDT 3.67875148

ALDV 4.95913110

ALDW 3.99775217

ALDY 4.07052623

ALEA 4.26584657

ALEC 4.32251986

ALED 2.91924795

ALEE 3.35040987

ALEF 5.17913240

ALEG 2.91680735

ALEH 3.65138680

ALEI 5.73966809

ALEK 3.56004685

ALEL 6.49696525

ALEM 5.52186390

ALEN 3.25023412

ALEP 3.30657340

ALEQ 3.71806683

ALER 3.70022224

ALES 3.47845341

ALET 3.97141595

ALEV 5.23452195

ALEW 4.26713992

ALEY 4.38577208

ALFA 4.01210192

ALFC 3.84129789

ALFD 2.40916467

ALFE 2.81281837

ALFF 4.71954528

ALFG 2.79204767

ALFH 3.17829966

ALFI 5.25436809

ALFK 3.07221714

ALFL 5.74636042

ALFM 5.01034773

ALFN 2.85955007

ALFP 2.92557769

ALFQ 3.25369798

ALFR 3.09265712

ALFS 3.18944796

ALFT 3.65798306

ALFV 4.79726138

ALFW 3.82377433

ALFY 3.97600759

ALGA 4.65009640

ALGC 4.31705315

ALGD 2.89113499

ALGE 3.43529228

ALGF 5.59266798

ALGG 3.02161059

ALGH 3.81777824

ALGI 6.19424335

ALGK 3.61590385

ALGL 7.04860323

ALGM 6.05717640

ALGN 3.34649363

ALGP 3.52089714

ALGQ 3.97520535

ALGR 3.78058134

ALGS 3.73424130

ALGT 4.27516101

ALGV 5.65650919

ALGW 4.50838522

ALGY 4.68553067

ALHA 3.83605918

ALHC 3.33619810

ALHD 2.50124353

ALHE 2.90100936

ALHF 4.68119393

ALHG 2.45991720

ALHH 3.02149910

ALHI 5.24815901

ALHK 3.05464587

ALHL 5.96394748

ALHM 4.98423950

ALHN 2.77666260

ALHP 2.90689235

ALHQ 3.30963540

ALHR 3.21614738

ALHS 3.06092065

ALHT 3.54753023

ALHV 4.75458702

ALHW 3.84734505

ALHY 3.90856420

ALIA 4.44769348

ALIC 4.48275889

ALID 2.88288644

ALIE 3.35537670

ALIF 5.36636250

ALIG 3.16715395

ALIH 3.71088282

ALII 5.80346764

ALIK 3.58431654

ALIL 6.45885857

ALIM 5.59982105

ALIN 3.32866733

ALIP 3.41395942

ALIQ 3.74569536

ALIR 3.62691386

ALIS 3.65819853

ALIT 4.12811428

ALIV 5.31873089

ALIW 4.40809893

ALIY 4.57075376

ALKA 4.04099831

ALKC 4.15054969

ALKD 2.75673366

ALKE 3.15956460

ALKF 5.05717013

ALKG 2.73537168

ALKH 3.46218245

ALKI 5.61348659

ALKK 3.37880066

ALKL 6.31319005

ALKM 5.32158232

ALKN 3.14935581

ALKP 3.12608456

ALKQ 3.48243694

ALKR 3.38330945

ALKS 3.29155752

ALKT 3.74144388

ALKV 5.02181078

ALKW 4.07979257

ALKY 4.30163715

ALLA 4.45999592

ALLC 4.38941589

ALLD 2.83964693

ALLE 3.25204099

ALLF 5.19818503

ALLG 3.17655941

ALLH 3.69201042

ALLI 5.67211185

ALLK 3.46345069

ALLL 6.25804283

ALLM 5.46458298

ALLN 3.20582415

ALLP 3.35829330

ALLQ 3.67012821

ALLR 3.58813411

ALLS 3.57807570

ALLT 4.09266624

ALLV 5.25971606

ALLW 4.35016060

ALLY 4.41718098

ALMA 4.37627295

ALMC 4.38012907

ALMD 2.83621966

ALME 3.26020659

ALMF 5.22410701

ALMG 3.16747568

ALMH 3.63614010

ALMI 5.70027248

ALMK 3.43327712

ALML 6.39070118

ALMM 5.45827448

ALMN 3.20705434

ALMP 3.38819446

ALMQ 3.64426774

ALMR 3.56451100

ALMS 3.51834354

ALMT 4.03027793

ALMV 5.24065186

ALMW 4.35446372

ALMY 4.49412730

ALNA 3.87071624

ALNC 3.91772176

ALND 2.64464335

ALNE 3.03564327

ALNF 4.87635941

ALNG 2.62543110

ALNH 3.34559679

ALNI 5.46411517

ALNK 3.27853048

ALNL 6.14583433

ALNM 5.20218153

ALNN 3.01809383

ALNP 3.05139943

ALNQ 3.41983336

ALNR 3.27050865

ALNS 3.21588309

ALNT 3.64276936

ALNV 4.88605170

ALNW 3.92648924

ALNY 4.14889345

ALPA 4.18507491

ALPC 3.91973907

ALPD 2.79471430

ALPE 3.20822075

ALPF 5.07261870

ALPG 2.75488153

ALPH 3.56457924

ALPI 5.66395425

ALPK 3.34178401

ALPL 6.52198681

ALPM 5.49313286

ALPN 3.05030377

ALPP 3.30301237

ALPQ 3.66957254

ALPR 3.55664277

ALPS 3.40012180

ALPT 3.92359437

ALPV 5.21314679

ALPW 4.09353151

ALPY 4.25084538

ALQA 4.17765396

ALQC 4.18264476

ALQD 2.86994582

ALQE 3.23092514

ALQF 5.10456549

ALQG 2.92362785

ALQH 3.59442153

ALQI 5.66224307

ALQK 3.39973526

ALQL 6.40906854

ALQM 5.41740543

ALQN 3.14154531

ALQP 3.29022755

ALQQ 3.62004330

ALQR 3.57159680

ALQS 3.38383598

ALQT 3.87879763

ALQV 5.13210716

ALQW 4.31480398

ALQY 4.32639513

ALRA 4.11751358

ALRC 4.10974247

ALRD 2.72330469

ALRE 3.12989040

ALRF 4.88965524

ALRG 2.76286469

ALRH 3.48138563

ALRI 5.42732769

ALRK 3.15477672

ALRL 6.18140407

ALRM 5.23750754

ALRN 2.94612717

ALRP 3.14210962

ALRQ 3.45409383

ALRR 3.41078986

ALRS 3.23883759

ALRT 3.75500287

ALRV 5.01323254

ALRW 4.07183656

ALRY 4.08708961

ALSA 4.71307939

ALSC 4.86860530

ALSD 3.23634632

ALSE 3.66272122

ALSF 5.86925876

ALSG 3.40397139

ALSH 4.12075041

ALSI 6.46137143

ALSK 3.90573672

ALSL 7.34419901

ALSM 6.24911070

ALSN 3.70079675

ALSP 3.78500067

ALSQ 4.17501742

ALSR 4.01983953

ALSS 3.92798827

ALST 4.46485801

ALSV 5.84826693

ALSW 4.81565635

ALSY 4.95547539

ALTA 4.46634305

ALTC 4.53572996

ALTD 2.96019070

ALTE 3.35843329

ALTF 5.50578920

ALTG 3.16305389

ALTH 3.81035916

ALTI 6.03719350

ALTK 3.56970497

ALTL 6.89224298

ALTM 5.83881041

ALTN 3.35515110

ALTP 3.54338089

ALTQ 3.85905008

ALTR 3.76301354

ALTS 3.65168926

ALTT 4.13390198

ALTV 5.49521141

ALTW 4.53505313

ALTY 4.58685996

ALVA 4.73841420

ALVC 4.73818159

ALVD 3.11989710

ALVE 3.59997688

ALVF 5.66749216

ALVG 3.36867448

ALVH 3.96689213

ALVI 6.17174429

ALVK 3.79916864

ALVL 6.98514689

ALVM 6.02772439

ALVN 3.50117772

ALVP 3.70188275

ALVQ 4.04917793

ALVR 3.96841320

ALVS 3.85005977

ALVT 4.39174295

ALVV 5.71097385

ALVW 4.69277716

ALVY 4.80359161

ALWA 3.78100572

ALWC 3.18154676

ALWD 2.16813821

ALWE 2.60054772

ALWF 4.23682324

ALWG 2.47950071

ALWH 2.90685341

ALWI 4.82304468

ALWK 2.78343401

ALWL 5.38840657

ALWM 4.65008798

ALWN 2.49634845

ALWP 2.66828411

ALWQ 3.04527381

ALWR 2.91670027

ALWS 2.89766363

ALWT 3.38359500

ALWV 4.40548268

ALWW 3.41351635

ALWY 3.46326445

ALYA 3.79026895

ALYC 3.61785336

ALYD 2.38843896

ALYE 2.81463836

ALYF 4.60578367

ALYG 2.55278362

ALYH 3.10259466

ALYI 5.15267059

ALYK 3.08151587

ALYL 5.70298293

ALYM 4.92106744

ALYN 2.81057339

ALYP 2.83767354

ALYQ 3.19249723

ALYR 3.02228619

ALYS 3.04816564

ALYT 3.50578458

ALYV 4.63771946

ALYW 3.60642755

ALYY 3.85123511

AMAA 6.06898511

AMAC 6.23172341

AMAD 4.61436344

AMAE 5.16102603

AMAF 6.86974354

AMAG 4.87953829

AMAH 5.48219063

AMAI 7.47333954

AMAK 5.52021931

AMAL 7.72284535

AMAM 10.86620123

AMAN 5.26009440

AMAP 4.80780024

AMAQ 5.96703938

AMAR 5.48619107

AMAS 5.55341440

AMAT 6.05148335

AMAV 6.83988051

AMAW 5.92215299

AMAY 6.05103853

AMCA 4.92380413

AMCC 2.32487641

AMCD 3.40354904

AMCE 4.09779188

AMCF 5.40125046

AMCG 3.35483908

AMCH 3.67816262

AMCI 5.97889297

AMCK 4.24175506

AMCL 6.18217979

AMCM 7.63647930

AMCN 3.96424709

AMCP 3.39280686

AMCQ 4.66625196

AMCR 4.19605605

AMCS 4.32424698

AMCT 4.77000211

AMCV 5.48987859

AMCW 4.30419105

AMCY 4.73702694

AMDA 4.01100399

AMDC 3.81505570

AMDD 2.86265793

AMDE 3.27429344

AMDF 4.53295625

AMDG 2.80961296

AMDH 3.42256527

AMDI 4.99137182

AMDK 3.51940479

AMDL 5.19391697

AMDM 6.44616939

AMDN 3.28529222

AMDP 2.95254986

AMDQ 3.80781305

AMDR 3.56022799

AMDS 3.47756154

AMDT 3.82530155

AMDV 4.53857112

AMDW 3.82360700

AMDY 3.90730862

AMEA 4.27233942

AMEC 4.22165258

AMED 3.14112741

AMEE 3.55639550

AMEF 4.86417990

AMEG 3.19930493

AMEH 3.72735729

AMEI 5.29030537

AMEK 3.80470277

AMEL 5.48615971

AMEM 6.75007089

AMEN 3.59072202

AMEP 3.19328898

AMEQ 4.08469500

AMER 3.82590103

AMES 3.77315594

AMET 4.12721740

AMEV 4.82278779

AMEW 4.06900749

AMEY 4.19337632

AMFA 4.05434895

AMFC 3.75379263

AMFD 2.68999797

AMFE 3.04509319

AMFF 4.38043671

AMFG 3.03836282

AMFH 3.27329747

AMFI 4.82984341

AMFK 3.30302872

AMFL 4.94890798

AMFM 6.03238155

AMFN 3.20715910

AMFP 2.83807243

AMFQ 3.68648090

AMFR 3.29489757

AMFS 3.52020944

AMFT 3.85489259

AMFV 4.44433771

AMFW 3.74346457

AMFY 3.83375396

AMGA 4.77679478

AMGC 4.35064213

AMGD 3.19110515

AMGE 3.72786084

AMGF 5.34421326

AMGG 3.40697614

AMGH 3.99218945

AMGI 5.83398015

AMGK 3.95935911

AMGL 6.06952878

AMGM 7.59124379

AMGN 3.78937434

AMGP 3.43708636

AMGQ 4.54832686

AMGR 3.98802954

AMGS 4.19141968

AMGT 4.56789014

AMGV 5.31000701

AMGW 4.40583608

AMGY 4.64624700

AMHA 3.90714309

AMHC 3.29555944

AMHD 2.73434219

AMHE 3.13848459

AMHF 4.38744599

AMHG 2.79387570

AMHH 3.15144721

AMHI 4.85030287

AMHK 3.31851248

AMHL 5.05066735

AMHM 6.14444785

AMHN 3.11832517

AMHP 2.84112887

AMHQ 3.69714417

AMHR 3.37397452

AMHS 3.38770750

AMHT 3.71833697

AMHV 4.40080923

AMHW 3.68496112

AMHY 3.74486545

AMIA 4.47154772

AMIC 4.38817811

AMID 3.12002877

AMIE 3.58198904

AMIF 5.00075963

AMIG 3.42744072

AMIH 3.79023626

AMII 5.32866572

AMIK 3.77913256

AMIL 5.53579771

AMIM 6.65456540

AMIN 3.66272315

AMIP 3.28171584

AMIQ 4.16306468

AMIR 3.78455693

AMIS 3.96524916

AMIT 4.28978077

AMIV 4.90972736

AMIW 4.28419638

AMIY 4.41269802

AMKA 4.14519661

AMKC 4.14443276

AMKD 3.06154582

AMKE 3.41758728

AMKF 4.70309877

AMKG 3.05657828

AMKH 3.60725780

AMKI 5.16906224

AMKK 3.63459290

AMKL 5.37876909

AMKM 6.55956434

AMKN 3.47921310

AMKP 3.08681221

AMKQ 3.90821891

AMKR 3.62495480

AMKS 3.63644042

AMKT 3.95480095

AMKV 4.65524965

AMKW 3.97715604

AMKY 4.14231887

AMLA 4.39555371

AMLC 4.25899565

AMLD 3.04424514

AMLE 3.42152616

AMLF 4.82734340

AMLG 3.34298713

AMLH 3.70636589

AMLI 5.17880857

AMLK 3.62815940

AMLL 5.33961949

AMLM 6.45591263

AMLN 3.51310879

AMLP 3.16747444

AMLQ 4.01647098

AMLR 3.67171653

AMLS 3.83370063

AMLT 4.18368444

AMLV 4.78977299

AMLW 4.14760338

AMLY 4.22379009

AMMA 4.56167597

AMMC 4.40705369

AMMD 3.20197122

AMME 3.59974400

AMMF 5.00502537

AMMG 3.53845271

AMMH 3.82793237

AMMI 5.38120489

AMMK 3.76945345

AMML 5.56897309

AMMM 6.72449962

AMMN 3.69511288

AMMP 3.38529238

AMMQ 4.21486784

AMMR 3.80521434

AMMS 3.98950948

AMMT 4.34716292

AMMV 4.96805368

AMMW 4.35122738

AMMY 4.43791817

AMNA 4.00882691

AMNC 3.90361458

AMND 2.92278756

AMNE 3.35147769

AMNF 4.58536915

AMNG 2.98980010

AMNH 3.49840586

AMNI 5.09810937

AMNK 3.53423785

AMNL 5.25816163

AMNM 6.50108007

AMNN 3.36896825

AMNP 3.03789475

AMNQ 3.85710689

AMNR 3.49352672

AMNS 3.58791247

AMNT 3.88935901

AMNV 4.59724618

AMNW 3.87693186

AMNY 4.05838570

AMPA 4.28645064

AMPC 3.96347834

AMPD 3.02949026

AMPE 3.42679341

AMPF 4.82106364

AMPG 3.09761618

AMPH 3.72672617

AMPI 5.28839304

AMPK 3.65950672

AMPL 5.54330660

AMPM 7.09519950

AMPN 3.56511695

AMPP 3.20049007

AMPQ 4.15384234

AMPR 3.70228234

AMPS 3.79633352

AMPT 4.20293500

AMPV 4.86255657

AMPW 4.07520627

AMPY 4.20859570

AMQA 4.25617276

AMQC 4.12356182

AMQD 3.12076309

AMQE 3.48130096

AMQF 4.80578344

AMQG 3.22420040

AMQH 3.71074168

AMQI 5.26889715

AMQK 3.67151500

AMQL 5.47055509

AMQM 6.63941268

AMQN 3.49299819

AMQP 3.21524421

AMQQ 4.04295358

AMQR 3.76551012

AMQS 3.72795182

AMQT 4.10694471

AMQV 4.78929431

AMQW 4.11612934

AMQY 4.21916945

AMRA 4.10682391

AMRC 3.99637514

AMRD 2.92931647

AMRE 3.33760387

AMRF 4.54628285

AMRG 2.98134598

AMRH 3.54317404

AMRI 4.97783515

AMRK 3.41679011

AMRL 5.21727279

AMRM 6.37409981

AMRN 3.29970920

AMRP 3.00171979

AMRQ 3.86129191

AMRR 3.53136574

AMRS 3.53470204

AMRT 3.88737772

AMRV 4.58265198

AMRW 3.89575359

AMRY 3.94634233

AMSA 4.88075617

AMSC 4.87927404

AMSD 3.54028755

AMSE 3.97743185

AMSF 5.58391261

AMSG 3.77980839

AMSH 4.26210086

AMSI 6.11073786

AMSK 4.23784484

AMSL 6.31435864

AMSM 7.86734690

AMSN 4.12065329

AMSP 3.73400162

AMSQ 4.70990709

AMSR 4.27123069

AMSS 4.35542892

AMST 4.77734637

AMSV 5.54004582

AMSW 4.72128989

AMSY 4.86216370

AMTA 4.57618236

AMTC 4.49507183

AMTD 3.26234106

AMTE 3.61515297

AMTF 5.21359738

AMTG 3.50254472

AMTH 3.95289989

AMTI 5.67348361

AMTK 3.89704432

AMTL 5.91302090

AMTM 7.26782514

AMTN 3.74551471

AMTP 3.47580267

AMTQ 4.26957649

AMTR 3.92670066

AMTS 4.02076746

AMTT 4.38000947

AMTV 5.16339226

AMTW 4.44053427

AMTY 4.51942582

AMVA 4.76070287

AMVC 4.66929201

AMVD 3.38852226

AMVE 3.84061075

AMVF 5.29987291

AMVG 3.64150083

AMVH 4.07090810

AMVI 5.71847112

AMVK 4.05829191

AMVL 5.95694099

AMVM 7.36112587

AMVN 3.91089206

AMVP 3.60099063

AMVQ 4.50873856

AMVR 4.09496054

AMVS 4.22293966

AMVT 4.59391893

AMVV 5.24848239

AMVW 4.52750743

AMVY 4.68635135

AMWA 3.72870856

AMWC 3.15149177

AMWD 2.39073561

AMWE 2.78349434

AMWF 3.92647066

AMWG 2.69182977

AMWH 3.03507684

AMWI 4.41139106

AMWK 2.97668188

AMWL 4.51483711

AMWM 5.62490872

AMWN 2.95361660

AMWP 2.45734490

AMWQ 3.50454785

AMWR 3.03816211

AMWS 3.17272844

AMWT 3.58297089

AMWV 3.98857807

AMWW 3.26942756

AMWY 3.34181186

AMYA 3.87555402

AMYC 3.62702411

AMYD 2.63203549

AMYE 3.03504910

AMYF 4.28582780

AMYG 2.86227227

AMYH 3.20988907

AMYI 4.74713301

AMYK 3.28004975

AMYL 4.86129667

AMYM 5.96712378

AMYN 3.09205305

AMYP 2.74508836

AMYQ 3.60919346

AMYR 3.22917951

AMYS 3.36414489

AMYT 3.69547299

AMYV 4.31985546

AMYW 3.59710472

AMYY 3.73102569

ANAA 5.83795099

ANAC 5.55778584

ANAD 7.25840908

ANAE 6.35370015

ANAF 4.71439425

ANAG 6.52944264

ANAH 6.79410879

ANAI 4.79399623

ANAK 6.54163189

ANAL 4.77165091

ANAM 5.26009440

ANAN 10.81843493

ANAP 5.60306019

ANAQ 6.48189181

ANAR 6.20886241

ANAS 6.81165982

ANAT 6.37582400

ANAV 4.89958419

ANAW 4.64513155

ANAY 5.48000674

ANCA 4.52349621

ANCC 1.74494237

ANCD 5.72389413

ANCE 5.12046943

ANCF 3.44865927

ANCG 4.71115435

ANCH 4.77673249

ANCI 3.57453049

ANCK 5.21505581

ANCL 3.59795995

ANCM 4.10045955

ANCN 7.55341638

ANCP 3.91359540

ANCQ 5.20561125

ANCR 4.87609246

ANCS 5.37173173

ANCT 5.06715969

ANCV 3.70559572

ANCW 3.43381920

ANCY 4.16559911

ANDA 4.21932477

ANDC 3.43593405

ANDD 5.06990988

ANDE 4.51431018

ANDF 3.06241669

ANDG 4.50104948

ANDH 4.64510416

ANDI 3.23479943

ANDK 4.62700663

ANDL 3.20117079

ANDM 3.57496983

ANDN 6.43478602

ANDP 3.87061476

ANDQ 4.62595288

ANDR 4.35423643

ANDS 4.78789727

ANDT 4.49475927

ANDV 3.32539388

ANDW 2.92986756

ANDY 3.60983146

ANEA 4.40188103

ANEC 3.73381231

ANED 5.34126054

ANEE 4.76229683

ANEF 3.27324532

ANEG 4.87335243

ANEH 4.91638673

ANEI 3.43613583

ANEK 4.84424452

ANEL 3.39245652

ANEM 3.78229753

ANEN 6.74187388

ANEP 4.10782690

ANEQ 4.79290203

ANER 4.56454164

ANES 5.00826528

ANET 4.69749317

ANEV 3.52727916

ANEW 3.07391605

ANEY 3.84608543

ANFA 3.52078625

ANFC 2.94200583

ANFD 4.65459277

ANFE 4.02602726

ANFF 2.52886672

ANFG 3.93832520

ANFH 4.13112100

ANFI 2.66222192

ANFK 4.18038027

ANFL 2.62955784

ANFM 3.04545019

ANFN 6.09889867

ANFP 3.41114649

ANFQ 4.16371308

ANFR 3.87166191

ANFS 4.31118922

ANFT 3.96171270

ANFV 2.78710362

ANFW 2.52955235

ANFY 3.13263058

ANGA 4.52006466

ANGC 3.87167961

ANGD 5.55691712

ANGE 4.88970227

ANGF 3.48781413

ANGG 4.72948965

ANGH 5.12894861

ANGI 3.58502984

ANGK 5.01517010

ANGL 3.57514165

ANGM 4.06330761

ANGN 7.53677697

ANGP 4.20401264

ANGQ 5.10837111

ANGR 4.73457300

ANGS 5.28984278

ANGT 4.94578664

ANGV 3.65866399

ANGW 3.33015502

ANGY 4.04789434

ANHA 3.84830394

ANHC 2.83392313

ANHD 4.82622106

ANHE 4.25246552

ANHF 2.81137299

ANHG 4.18775214

ANHH 4.24022518

ANHI 3.00524764

ANHK 4.38366099

ANHL 2.98084173

ANHM 3.26016248

ANHN 6.21706553

ANHP 3.64417111

ANHQ 4.37140334

ANHR 4.13065133

ANHS 4.51692891

ANHT 4.15222660

ANHV 3.05125291

ANHW 2.79590359

ANHY 3.38029656

ANIA 3.95478039

ANIC 3.51857982

ANID 5.21544713

ANIE 4.58248754

ANIF 3.04459515

ANIG 4.49997717

ANIH 4.76377311

ANII 3.04667762

ANIK 4.69944477

ANIL 3.07515700

ANIM 3.45628680

ANIN 6.73110421

ANIP 3.94527055

ANIQ 4.65089168

ANIR 4.38528457

ANIS 4.83559979

ANIT 4.46793958

ANIV 3.12009435

ANIW 3.06273100

ANIY 3.74323382

ANKA 4.29375917

ANKC 3.64976025

ANKD 5.33118295

ANKE 4.70792570

ANKF 3.26787532

ANKG 4.83201157

ANKH 4.84497949

ANKI 3.34903944

ANKK 4.76534656

ANKL 3.37703675

ANKM 3.67240172

ANKN 6.55902896

ANKP 4.05699545

ANKQ 4.68004789

ANKR 4.42363134

ANKS 4.92325008

ANKT 4.60067884

ANKV 3.42377683

ANKW 3.10326970

ANKY 3.87492525

ANLA 3.90620716

ANLC 3.34814473

ANLD 5.04572305

ANLE 4.41495238

ANLF 2.88659909

ANLG 4.41810189

ANLH 4.62057189

ANLI 2.92100633

ANLK 4.52805545

ANLL 2.91779765

ANLM 3.36875027

ANLN 6.57204440

ANLP 3.79289652

ANLQ 4.49868934

ANLR 4.25608567

ANLS 4.69099983

ANLT 4.33123724

ANLV 3.03492709

ANLW 2.85480505

ANLY 3.54643850

ANMA 4.01158644

ANMC 3.60367914

ANMD 5.21193044

ANME 4.55314519

ANMF 3.08102907

ANMG 4.53626396

ANMH 4.75432019

ANMI 3.11685655

ANMK 4.63059734

ANML 3.14139440

ANMM 3.48040661

ANMN 6.70944089

ANMP 3.92761409

ANMQ 4.61421461

ANMR 4.38927705

ANMS 4.79154523

ANMT 4.43535467

ANMV 3.21849724

ANMW 3.12206761

ANMY 3.74443851

ANNA 4.15818489

ANNC 3.57246869

ANND 5.17906765

ANNE 4.57391702

ANNF 3.17739714

ANNG 4.59711283

ANNH 4.71306310

ANNI 3.32560986

ANNK 4.70611380

ANNL 3.32798713

ANNM 3.62875927

ANNN 6.49545073

ANNP 3.94991557

ANNQ 4.67875906

ANNR 4.38864879

ANNS 4.87208701

ANNT 4.56123212

ANNV 3.40703701

ANNW 3.13283975

ANNY 3.77170067

ANPA 4.37773607

ANPC 3.61062287

ANPD 5.41225671

ANPE 4.69640264

ANPF 3.22295164

ANPG 4.72437303

ANPH 4.91575561

ANPI 3.35382694

ANPK 4.79541285

ANPL 3.34419529

ANPM 3.76388012

ANPN 7.12847214

ANPP 4.09976405

ANPQ 4.80360907

ANPR 4.52304378

ANPS 5.08003253

ANPT 4.80625720

ANPV 3.52167779

ANPW 3.06309034

ANPY 3.83365513

ANQA 4.37637952

ANQC 3.63095693

ANQD 5.32746478

ANQE 4.69441330

ANQF 3.34760499

ANQG 4.82073151

ANQH 4.88812629

ANQI 3.35932041

ANQK 4.76428333

ANQL 3.38690469

ANQM 3.72869030

ANQN 6.63345839

ANQP 4.13772070

ANQQ 4.78918829

ANQR 4.53615449

ANQS 4.96029041

ANQT 4.65602378

ANQV 3.49562462

ANQW 3.16239796

ANQY 3.85732211

ANRA 4.13457244

ANRC 3.41377586

ANRD 5.06464063

ANRE 4.42664424

ANRF 2.98906888

ANRG 4.54049677

ANRH 4.63479475

ANRI 3.05859768

ANRK 4.45307894

ANRL 3.10404065

ANRM 3.44184641

ANRN 6.40759157

ANRP 3.84486675

ANRQ 4.47028596

ANRR 4.27378560

ANRS 4.69591161

ANRT 4.37786050

ANRV 3.21630235

ANRW 2.90790527

ANRY 3.52367048

ANSA 4.85054509

ANSC 4.45472545

ANSD 5.94830817

ANSE 5.22545741

ANSF 3.89337342

ANSG 5.33790525

ANSH 5.53840018

ANSI 3.94658969

ANSK 5.38395557

ANSL 3.95449272

ANSM 4.33192379

ANSN 7.73590652

ANSP 4.60513780

ANSQ 5.33364333

ANSR 5.11106955

ANSS 5.60258525

ANST 5.30545871

ANSV 4.05648629

ANSW 3.77888294

ANSY 4.53326276

ANTA 4.48567784

ANTC 4.01618622

ANTD 5.58431150

ANTE 4.86340878

ANTF 3.44881930

ANTG 4.94461919

ANTH 5.10140058

ANTI 3.53513494

ANTK 4.99261709

ANTL 3.57374579

ANTM 3.91278273

ANTN 7.20575995

ANTP 4.28954513

ANTQ 4.97745058

ANTR 4.72135564

ANTS 5.26341964

ANTT 4.89722914

ANTV 3.64477850

ANTW 3.41675250

ANTY 4.06460818

ANVA 4.35818917

ANVC 3.91262243

ANVD 5.65279270

ANVE 4.91913748

ANVF 3.31238759

ANVG 4.91442311

ANVH 5.15112405

ANVI 3.31830579

ANVK 5.01975295

ANVL 3.36665520

ANVM 3.79094377

ANVN 7.41022728

ANVP 4.26055001

ANVQ 5.01063768

ANVR 4.74473890

ANVS 5.22714327

ANVT 4.84405988

ANVV 3.40934150

ANVW 3.29673704

ANVY 4.02960170

ANWA 3.29532318

ANWC 2.51410745

ANWD 4.29780266

ANWE 3.72223786

ANWF 2.23937641

ANWG 3.63144740

ANWH 3.78771814

ANWI 2.40541897

ANWK 3.76157388

ANWL 2.40180699

ANWM 2.83247829

ANWN 5.69117912

ANWP 3.08568659

ANWQ 3.81727334

ANWR 3.51554681

ANWS 3.99956428

ANWT 3.66487519

ANWV 2.52278713

ANWW 2.23828240

ANWY 2.76731990

ANYA 3.60315497

ANYC 3.03943650

ANYD 4.65816937

ANYE 4.08643129

ANYF 2.62222448

ANYG 4.04482253

ANYH 4.15734981

ANYI 2.78004325

ANYK 4.21740326

ANYL 2.75463097

ANYM 3.18027856

ANYN 6.04470013

ANYP 3.46325990

ANYQ 4.20135711

ANYR 3.87258734

ANYS 4.35374301

ANYT 4.00098521

ANYV 2.84190477

ANYW 2.58045913

ANYY 3.23545078

APAA 6.15811742

APAC 4.84700857

APAD 6.03666860

APAE 5.93441510

APAF 4.60609856

APAG 5.75686604

APAH 5.42651533

APAI 5.01013048

APAK 5.84033960

APAL 4.94481818

APAM 4.80780024

APAN 5.60306019

APAP 11.50019689

APAQ 5.63449344

APAR 5.57983725

APAS 6.25647373

APAT 5.86263418

APAV 5.37238513

APAW 4.61815422

APAY 4.71274486

APCA 4.90683947

APCC 1.30434961

APCD 4.76740475

APCE 4.82658696

APCF 3.40274493

APCG 4.14638760

APCH 3.67490147

APCI 3.92219256

APCK 4.71556936

APCL 3.82068533

APCM 3.77089811

APCN 4.37521641

APCP 8.02079014

APCQ 4.45857565

APCR 4.35275928

APCS 4.94906089

APCT 4.64095086

APCV 4.20131967

APCW 3.18914666

APCY 3.54899780

APDA 4.56580957

APDC 3.17207586

APDD 4.47451095

APDE 4.43493222

APDF 3.17015271

APDG 4.10318527

APDH 3.83795471

APDI 3.55448438

APDK 4.26226960

APDL 3.51770414

APDM 3.40868260

APDN 4.08187193

APDP 7.38451551

APDQ 4.11890267

APDR 4.02156461

APDS 4.52460905

APDT 4.26257385

APDV 3.82804635

APDW 3.14579057

APDY 3.23063926

APEA 4.66171708

APEC 3.36070316

APED 4.67896908

APEE 4.56420155

APEF 3.31807939

APEG 4.39362424

APEH 4.00801502

APEI 3.65549613

APEK 4.39146378

APEL 3.56886523

APEM 3.46285721

APEN 4.28194745

APEP 7.75684298

APEQ 4.18966875

APER 4.08303125

APES 4.70803996

APET 4.39146466

APEV 3.96538012

APEW 3.19974985

APEY 3.37511902

APFA 3.86559945

APFC 2.58976895

APFD 3.93493425

APFE 3.85984371

APFF 2.73937883

APFG 3.62254090

APFH 3.37422470

APFI 3.05759639

APFK 3.87491774

APFL 2.92500706

APFM 2.91942214

APFN 3.70091834

APFP 6.90624986

APFQ 3.64179745

APFR 3.51588242

APFS 4.01268983

APFT 3.68919141

APFV 3.20055175

APFW 2.67052352

APFY 2.80082545

APGA 4.95520618

APGC 3.39662738

APGD 4.69625518

APGE 4.75834771

APGF 3.58087179

APGG 4.27042648

APGH 4.16430773

APGI 3.96781743

APGK 4.66962675

APGL 3.94863317

APGM 3.86955723

APGN 4.46223325

APGP 8.20755197

APGQ 4.54714639

APGR 4.40110868

APGS 4.92717612

APGT 4.64047274

APGV 4.21283934

APGW 3.45621289

APGY 3.59532894

APHA 4.23238457

APHC 2.52070968

APHD 4.22791156

APHE 4.13517889

APHF 2.90944909

APHG 3.85819070

APHH 3.41667490

APHI 3.31416193

APHK 4.05030797

APHL 3.27972143

APHM 3.08260414

APHN 3.82678724

APHP 7.13426255

APHQ 3.87615389

APHR 3.82095264

APHS 4.26449190

APHT 3.97763982

APHV 3.55307266

APHW 2.86888871

APHY 2.95762571

APIA 4.24017168

APIC 3.11862905

APID 4.39864301

APIE 4.29267474

APIF 3.15442648

APIG 4.01849041

APIH 3.81194219

APII 3.36178776

APIK 4.26218388

APIL 3.29960359

APIM 3.20161953

APIN 4.11906249

APIP 7.60912781

APIQ 4.02237626

APIR 3.91126475

APIS 4.46432687

APIT 4.10573932

APIV 3.55227608

APIW 3.12147068

APIY 3.27849498

APKA 4.49038844

APKC 3.27580470

APKD 4.53337704

APKE 4.42111330

APKF 3.30040250

APKG 4.21921049

APKH 3.91162388

APKI 3.62918921

APKK 4.44494623

APKL 3.50036409

APKM 3.38521888

APKN 4.30834167

APKP 7.59861085

APKQ 4.07622479

APKR 3.96288568

APKS 4.60100862

APKT 4.26949731

APKV 3.85634700

APKW 3.11013592

APKY 3.37614446

APLA 4.18520235

APLC 3.02856738

APLD 4.30306875

APLE 4.15113242

APLF 2.99766686

APLG 3.99793915

APLH 3.70017138

APLI 3.26457274

APLK 4.09630050

APLL 3.18349906

APLM 3.11046065

APLN 3.97714429

APLP 7.42700549

APLQ 3.86902818

APLR 3.86057648

APLS 4.35390198

APLT 4.05816299

APLV 3.51970261

APLW 2.98669586

APLY 3.07937353

APMA 4.27895796

APMC 3.12913905

APMD 4.34552035

APME 4.25005596

APMF 3.12224283

APMG 4.08820576

APMH 3.75679687

APMI 3.37598069

APMK 4.12175284

APML 3.33597559

APMM 3.17021050

APMN 4.01583568

APMP 7.63298644

APMQ 3.92836935

APMR 3.87694855

APMS 4.40574495

APMT 4.11615703

APMV 3.60640522

APMW 3.12795540

APMY 3.20528192

APNA 4.39566405

APNC 3.20697739

APND 4.39805046

APNE 4.34471010

APNF 3.19930807

APNG 4.07273703

APNH 3.83542423

APNI 3.61102349

APNK 4.31960856

APNL 3.50643618

APNM 3.44437966

APNN 4.18878479

APNP 7.37886897

APNQ 4.10611133

APNR 3.90248988

APNS 4.51980914

APNT 4.22657281

APNV 3.78022545

APNW 3.19510842

APNY 3.31002432

APPA 5.02783489

APPC 3.26992869

APPD 4.72791272

APPE 4.73603720

APPF 3.46273358

APPG 4.33189093

APPH 4.14279301

APPI 3.91474013

APPK 4.62663361

APPL 3.87113657

APPM 3.79442997

APPN 4.37151940

APPP 7.90546015

APPQ 4.51329679

APPR 4.38515874

APPS 4.90060420

APPT 4.58426675

APPV 4.20872907

APPW 3.34947910

APPY 3.49841538

APQA 4.57088863

APQC 3.25018426

APQD 4.54910738

APQE 4.40851735

APQF 3.25173044

APQG 4.33831858

APQH 3.91970114

APQI 3.57373072

APQK 4.30844983

APQL 3.51032405

APQM 3.33464013

APQN 4.18295844

APQP 7.65927504

APQQ 4.13969005

APQR 4.04715089

APQS 4.61558810

APQT 4.31895220

APQV 3.87904042

APQW 3.27702037

APQY 3.29858507

APRA 4.46040747

APRC 3.12074029

APRD 4.41388454

APRE 4.24491264

APRF 3.07792758

APRG 4.17762631

APRH 3.77840698

APRI 3.38508184

APRK 4.08738665

APRL 3.35125594

APRM 3.22121935

APRN 3.96776532

APRP 7.41100706

APRQ 3.92473386

APRR 3.97668105

APRS 4.43958494

APRT 4.15260541

APRV 3.72148567

APRW 3.12166835

APRY 3.07117096

APSA 5.17594475

APSC 3.93836519

APSD 5.02918721

APSE 4.97573571

APSF 3.82344275

APSG 4.76809188

APSH 4.45444966

APSI 4.21350092

APSK 4.91004275

APSL 4.14516340

APSM 3.99405734

APSN 4.76027649

APSP 8.60496577

APSQ 4.68910486

APSR 4.59007288

APSS 5.19470774

APST 4.85187200

APSV 4.45362211

APSW 3.78773772

APSY 3.90740446

APTA 4.83650791

APTC 3.53996956

APTD 4.73215344

APTE 4.62968939

APTF 3.50359857

APTG 4.44904201

APTH 4.11911757

APTI 3.80549422

APTK 4.53636987

APTL 3.77689968

APTM 3.65069087

APTN 4.41354960

APTP 8.02105642

APTQ 4.35093998

APTR 4.28905375

APTS 4.84358863

APTT 4.51166575

APTV 4.07378491

APTW 3.47278478

APTY 3.53719966

APVA 4.72378475

APVC 3.44909287

APVD 4.74634159

APVE 4.67176774

APVF 3.40327090

APVG 4.38105168

APVH 4.11281109

APVI 3.63347623

APVK 4.58153593

APVL 3.65042801

APVM 3.56368952

APVN 4.38085551

APVP 8.18175300

APVQ 4.38848949

APVR 4.32149451

APVS 4.83082132

APVT 4.45272692

APVV 3.91114375

APVW 3.46400719

APVY 3.51398467

APWA 3.69228054

APWC 2.25166021

APWD 3.67586285

APWE 3.65206305

APWF 2.41272522

APWG 3.39001687

APWH 3.12473296

APWI 2.90105031

APWK 3.48447986

APWL 2.70278908

APWM 2.74238279

APWN 3.36962423

APWP 6.43600965

APWQ 3.37122616

APWR 3.31037623

APWS 3.73661444

APWT 3.49489006

APWV 3.04372023

APWW 2.35023067

APWY 2.46264963

APYA 3.89142224

APYC 2.62921739

APYD 3.93548250

APYE 3.92371343

APYF 2.73684689

APYG 3.65288772

APYH 3.36630327

APYI 3.12527858

APYK 3.93642998

APYL 2.99041183

APYM 2.95100518

APYN 3.76107348

APYP 6.87734958

APYQ 3.64765028

APYR 3.50887768

APYS 4.04569304

APYT 3.75883707

APYV 3.31435948

APYW 2.62529321

APYY 2.83915241

AQAA 6.12890081

AQAC 4.96033399

AQAD 6.44569811

AQAE 7.13135032

AQAF 4.70228934

AQAG 5.67199010

AQAH 6.72952393

AQAI 5.12258233

AQAK 7.00223314

AQAL 5.41436545

AQAM 5.96703938

AQAN 6.48189181

AQAP 5.63449344

AQAQ 10.15693286

AQAR 6.87802264

AQAS 6.23271881

AQAT 6.11746743

AQAV 5.21375777

AQAW 4.88575566

AQAY 5.33543450

AQCA 4.61646614

AQCC 1.21333389

AQCD 4.91402853

AQCE 5.61894976

AQCF 3.36191286

AQCG 3.82404230

AQCH 4.57141576

AQCI 3.80790199

AQCK 5.45778720

AQCL 4.04560364

AQCM 4.58886688

AQCN 4.93642838

AQCP 3.81973578

AQCQ 6.94472467

AQCR 5.29224842

AQCS 4.79134089

AQCT 4.70723253

AQCV 3.88153093

AQCW 3.39148988

AQCY 4.02210072

AQDA 4.63775477

AQDC 3.27290576

AQDD 4.69445862

AQDE 5.18480320

AQDF 3.18178236

AQDG 3.92365378

AQDH 4.61821217

AQDI 3.62645452

AQDK 5.14064445

AQDL 3.78524402

AQDM 4.17084613

AQDN 4.65726905

AQDP 3.89675683

AQDQ 6.09698343

AQDR 5.01609905

AQDS 4.56374205

AQDT 4.48003909

AQDV 3.73820882

AQDW 3.28807617

AQDY 3.61158810

AQEA 4.93350604

AQEC 3.60523882

AQED 5.03425369

AQEE 5.51306559

AQEF 3.45174790

AQEG 4.34896404

AQEH 4.93040379

AQEI 3.90479212

AQEK 5.46483488

AQEL 4.08770012

AQEM 4.50704512

AQEN 5.07249665

AQEP 4.22520584

AQEQ 6.43844779

AQER 5.32346535

AQES 4.89698860

AQET 4.77097230

AQEV 3.99942994

AQEW 3.50202667

AQEY 3.91293091

AQFA 3.60733327

AQFC 2.45221753

AQFD 4.02584068

AQFE 4.50584912

AQFF 2.49624140

AQFG 3.24693915

AQFH 4.02330818

AQFI 2.89723147

AQFK 4.43100468

AQFL 2.96840112

AQFM 3.42391764

AQFN 4.10752990

AQFP 3.23958438

AQFQ 5.56067142

AQFR 4.22545184

AQFS 3.86822678

AQFT 3.72638407

AQFV 2.88155084

AQFW 2.60765304

AQFY 3.08139571

AQGA 4.62433201

AQGC 3.20275208

AQGD 4.78849513

AQGE 5.45395067

AQGF 3.39397371

AQGG 3.96004326

AQGH 4.98162918

AQGI 3.77182843

AQGK 5.29067612

AQGL 4.00096116

AQGM 4.54489863

AQGN 4.88775489

AQGP 4.08512098

AQGQ 6.99140746

AQGR 5.21232930

AQGS 4.70599058

AQGT 4.62522453

AQGV 3.85929784

AQGW 3.44853661

AQGY 3.89663064

AQHA 4.17138432

AQHC 2.56969453

AQHD 4.35343918

AQHE 4.87124450

AQHF 2.82854468

AQHG 3.58522017

AQHH 4.16718070

AQHI 3.28056324

AQHK 4.74942299

AQHL 3.48555445

AQHM 3.78064252

AQHN 4.29588316

AQHP 3.62818432

AQHQ 5.83959517

AQHR 4.62953502

AQHS 4.20206052

AQHT 4.08311655

AQHV 3.37775266

AQHW 2.95909622

AQHY 3.30328979

AQIA 4.11893234

AQIC 3.12388129

AQID 4.64915915

AQIE 5.13130208

AQIF 3.07309831

AQIG 3.78127174

AQIH 4.66308118

AQII 3.28831066

AQIK 5.05068719

AQIL 3.50095150

AQIM 3.96542546

AQIN 4.71099985

AQIP 3.84668520

AQIQ 6.20721653

AQIR 4.85967071

AQIS 4.42461030

AQIT 4.25071632

AQIV 3.31234842

AQIW 3.20126158

AQIY 3.67505296

AQKA 4.61618795

AQKC 3.43172358

AQKD 4.92965465

AQKE 5.38108029

AQKF 3.37701871

AQKG 4.13816022

AQKH 4.75874968

AQKI 3.80238609

AQKK 5.21298156

AQKL 3.92080102

AQKM 4.30864689

AQKN 4.90639290

AQKP 4.04348560

AQKQ 6.12668051

AQKR 4.93814318

AQKS 4.68771627

AQKT 4.54994017

AQKV 3.80999981

AQKW 3.35254393

AQKY 3.86531100

AQLA 4.15581528

AQLC 3.00131438

AQLD 4.54829862

AQLE 5.01509121

AQLF 2.95440427

AQLG 3.78996260

AQLH 4.57098574

AQLI 3.23675085

AQLK 4.90520355

AQLL 3.37353133

AQLM 3.87108912

AQLN 4.55850994

AQLP 3.75808447

AQLQ 6.02095226

AQLR 4.78624696

AQLS 4.35252222

AQLT 4.20321730

AQLV 3.29436104

AQLW 3.09561771

AQLY 3.51697133

AQMA 4.21049116

AQMC 3.21744703

AQMD 4.61825290

AQME 5.14165142

AQMF 3.08747436

AQMG 3.87887571

AQMH 4.64585032

AQMI 3.35136317

AQMK 4.97547842

AQML 3.56089919

AQMM 4.02147928

AQMN 4.62242397

AQMP 3.85116500

AQMQ 6.22597657

AQMR 4.86461830

AQMS 4.37657171

AQMT 4.22774583

AQMV 3.43791075

AQMW 3.27945721

AQMY 3.70265350

AQNA 4.32689886

AQNC 3.20710956

AQND 4.63624459

AQNE 5.13223154

AQNF 3.23425853

AQNG 3.85211109

AQNH 4.61279774

AQNI 3.64429011

AQNK 5.07879704

AQNL 3.77164577

AQNM 4.12529604

AQNN 4.72931873

AQNP 3.88036602

AQNQ 6.10166300

AQNR 4.81549230

AQNS 4.51320122

AQNT 4.37539848

AQNV 3.66812315

AQNW 3.15885193

AQNY 3.69395673

AQPA 4.62110573

AQPC 3.15844887

AQPD 4.77762511

AQPE 5.30994221

AQPF 3.25692569

AQPG 3.94034458

AQPH 4.79376845

AQPI 3.64653481

AQPK 5.11041931

AQPL 3.83984810

AQPM 4.25657406

AQPN 4.69792561

AQPP 4.05025054

AQPQ 6.64453660

AQPR 5.05692637

AQPS 4.62932806

AQPT 4.55003447

AQPV 3.83049058

AQPW 3.26375767

AQPY 3.70205847

AQQA 4.80347758

AQQC 3.50584330

AQQD 5.00874103

AQQE 5.43855744

AQQF 3.39241989

AQQG 4.27716280

AQQH 4.89891401

AQQI 3.81885373

AQQK 5.29874257

AQQL 4.00351829

AQQM 4.38177070

AQQN 4.93824805

AQQP 4.19445182

AQQQ 6.37357990

AQQR 5.18159634

AQQS 4.78956133

AQQT 4.68554693

AQQV 3.89570196

AQQW 3.50084331

AQQY 3.85567707

AQRA 4.57424295

AQRC 3.26187596

AQRD 4.72460875

AQRE 5.17678657

AQRF 3.11081874

AQRG 4.01076599

AQRH 4.59261689

AQRI 3.50169519

AQRK 4.87862289

AQRL 3.72406745

AQRM 4.09341397

AQRN 4.56487606

AQRP 3.89874132

AQRQ 5.98644024

AQRR 4.88639831

AQRS 4.47860116

AQRT 4.36442227

AQRV 3.66580026

AQRW 3.25912744

AQRY 3.53055815

AQSA 5.01489766

AQSC 3.91718402

AQSD 5.27819239

AQSE 5.82543385

AQSF 3.83232579

AQSG 4.56193452

AQSH 5.45755467

AQSI 4.23085192

AQSK 5.73960004

AQSL 4.44063278

AQSM 4.87320770

AQSN 5.37001131

AQSP 4.54188264

AQSQ 7.21881934

AQSR 5.59927560

AQSS 5.15598869

AQST 5.02201438

AQSV 4.29115586

AQSW 3.89871919

AQSY 4.37572337

AQTA 4.67023837

AQTC 3.53341835

AQTD 4.93598714

AQTE 5.44917460

AQTF 3.45181523

AQTG 4.20034047

AQTH 5.07385844

AQTI 3.81720871

AQTK 5.35027347

AQTL 4.03792089

AQTM 4.41358040

AQTN 4.96995364

AQTP 4.22234953

AQTQ 6.70005230

AQTR 5.23721736

AQTS 4.79570821

AQTT 4.66447962

AQTV 3.88534907

AQTW 3.58522763

AQTY 3.94443324

AQVA 4.52906650

AQVC 3.38315406

AQVD 4.96159208

AQVE 5.49930382

AQVF 3.30552361

AQVG 4.13325263

AQVH 5.03171325

AQVI 3.57866707

AQVK 5.37726207

AQVL 3.80813886

AQVM 4.32184890

AQVN 4.97094783

AQVP 4.18593996

AQVQ 6.81126518

AQVR 5.25509135

AQVS 4.74219137

AQVT 4.60445188

AQVV 3.64705032

AQVW 3.43735421

AQVY 3.90962413

AQWA 3.45522498

AQWC 2.06496114

AQWD 3.69737030

AQWE 4.22191890

AQWF 2.17088460

AQWG 2.97825124

AQWH 3.65483117

AQWI 2.62742320

AQWK 4.08188589

AQWL 2.74065027

AQWM 3.23021527

AQWN 3.67070998

AQWP 2.89576946

AQWQ 5.22892121

AQWR 3.95571101

AQWS 3.57779441

AQWT 3.45322819

AQWV 2.66281557

AQWW 2.33345660

AQWY 2.59179133

AQYA 3.70302087

AQYC 2.61564576

AQYD 4.06976828

AQYE 4.60768191

AQYF 2.63874174

AQYG 3.34890592

AQYH 4.03533676

AQYI 3.04710397

AQYK 4.53825134

AQYL 3.16724545

AQYM 3.63666756

AQYN 4.17438500

AQYP 3.32813648

AQYQ 5.52969519

AQYR 4.24255940

AQYS 3.92755014

AQYT 3.79181007

AQYV 3.05046077

AQYW 2.73836909

AQYY 3.16945025

ARAA 5.98683420

ARAC 5.20248712

ARAD 5.94918696

ARAE 6.44549561

ARAF 4.65770970

ARAG 5.56154844

ARAH 6.52959944

ARAI 5.10133696

ARAK 7.70329077

ARAL 5.33412090

ARAM 5.48619107

ARAN 6.20886241

ARAP 5.57983725

ARAQ 6.87802264

ARAR 10.34305854

ARAS 6.06210593

ARAT 5.99111983

ARAV 5.18313158

ARAW 5.24412169

ARAY 5.39884956

ARCA 4.52120009

ARCC 1.22893371

ARCD 4.53620724

ARCE 5.09590742

ARCF 3.38084454

ARCG 3.75078980

ARCH 4.44526070

ARCI 3.85207096

ARCK 6.12563448

ARCL 3.99928867

ARCM 4.17840218

ARCN 4.75244846

ARCP 3.81623832

ARCQ 5.40757976

ARCR 7.19974195

ARCS 4.67401348

ARCT 4.63592429

ARCV 3.89330191

ARCW 3.55113607

ARCY 4.12246423

ARDA 4.51044041

ARDC 3.38661566

ARDD 4.28512357

ARDE 4.71984363

ARDF 3.16555750

ARDG 3.79730239

ARDH 4.56864949

ARDI 3.61444066

ARDK 5.43651824

ARDL 3.77154076

ARDM 3.88235090

ARDN 4.41283035

ARDP 3.91344995

ARDQ 5.00198685

ARDR 6.26543637

ARDS 4.37336941

ARDT 4.37572190

ARDV 3.71918397

ARDW 3.51299717

ARDY 3.70219902

AREA 4.78064839

AREC 3.69686658

ARED 4.59985675

AREE 5.00372236

AREF 3.45422298

AREG 4.21003029

AREH 4.87623620

AREI 3.92549073

AREK 5.77227246

AREL 4.07742130

AREM 4.20081317

AREN 4.82022376

AREP 4.18089686

AREQ 5.30340081

ARER 6.58808782

ARES 4.70023906

ARET 4.66101143

AREV 3.99253395

AREW 3.81517486

AREY 3.99913898

ARFA 3.56415002

ARFC 2.57937587

ARFD 3.73813811

ARFE 4.09916229

ARFF 2.49828489

ARFG 3.17708366

ARFH 3.93342918

ARFI 2.90315297

ARFK 4.98342752

ARFL 2.96927981

ARFM 3.13588376

ARFN 3.97744040

ARFP 3.26232197

ARFQ 4.41752607

ARFR 5.74896301

ARFS 3.78017387

ARFT 3.69525052

ARFV 2.89150410

ARFW 2.88886121

ARFY 3.20485956

ARGA 4.51299504

ARGC 3.28373225

ARGD 4.36406386

ARGE 4.90196161

ARGF 3.35303817

ARGG 3.82192524

ARGH 4.83805188

ARGI 3.73521669

ARGK 5.91523181

ARGL 3.95086147

ARGM 4.11810339

ARGN 4.64464191

ARGP 4.06461563

ARGQ 5.31736372

ARGR 7.14232052

ARGS 4.55903190

ARGT 4.52183631

ARGV 3.82836721

ARGW 3.79142629

ARGY 3.94550662

ARHA 4.15591859

ARHC 2.72128709

ARHD 4.06116626

ARHE 4.48129759

ARHF 2.92195717

ARHG 3.58597807

ARHH 4.15111643

ARHI 3.41432270

ARHK 5.26563851

ARHL 3.52736028

ARHM 3.56943066

ARHN 4.19848124

ARHP 3.71999783

ARHQ 4.80733348

ARHR 6.09110039

ARHS 4.14645636

ARHT 4.11119582

ARHV 3.45057809

ARHW 3.29643429

ARHY 3.47770905

ARIA 4.01567782

ARIC 3.29987547

ARID 4.29686845

ARIE 4.71314437

ARIF 3.06052128

ARIG 3.70600493

ARIH 4.57132767

ARII 3.32839267

ARIK 5.59243947

ARIL 3.47059853

ARIM 3.67137253

ARIN 4.54121617

ARIP 3.80885102

ARIQ 4.97584148

ARIR 6.44249077

ARIS 4.31027926

ARIT 4.17420381

ARIV 3.30086817

ARIW 3.49435681

ARIY 3.79782414

ARKA 4.47963668

ARKC 3.59270622

ARKD 4.49294359

ARKE 4.91175355

ARKF 3.43042589

ARKG 4.04700484

ARKH 4.73905617

ARKI 3.87126006

ARKK 5.64816218

ARKL 3.95428731

ARKM 4.11036234

ARKN 4.77550248

ARKP 4.02289836

ARKQ 5.13303671

ARKR 6.38186385

ARKS 4.57828005

ARKT 4.48369783

ARKV 3.82778549

ARKW 3.69007185

ARKY 4.02551757

ARLA 4.11790779

ARLC 3.13774186

ARLD 4.24409488

ARLE 4.62302994

ARLF 2.95652086

ARLG 3.75190448

ARLH 4.50939327

ARLI 3.24670410

ARLK 5.44148693

ARLL 3.37592065

ARLM 3.59228115

ARLN 4.39612108

ARLP 3.81816793

ARLQ 4.91090196

ARLR 6.25829402

ARLS 4.25635163

ARLT 4.16127686

ARLV 3.31986493

ARLW 3.42889615

ARLY 3.62990268

ARMA 4.07929604

ARMC 3.25927206

ARMD 4.26269162

ARME 4.64190358

ARMF 3.02168825

ARMG 3.76361738

ARMH 4.52061995

ARMI 3.33772961

ARMK 5.53181267

ARML 3.49829144

ARMM 3.62946494

ARMN 4.43136523

ARMP 3.83747541

ARMQ 4.90729233

ARMR 6.45015035

ARMS 4.22935753

ARMT 4.13559945

ARMV 3.38659941

ARMW 3.59793625

ARMY 3.74457586

ARNA 4.09787259

ARNC 3.26335136

ARND 4.16106588

ARNE 4.58690123

ARNF 3.16473722

ARNG 3.67570198

ARNH 4.50474421

ARNI 3.60503223

ARNK 5.49384486

ARNL 3.66962828

ARNM 3.78501583

ARNN 4.47987712

ARNP 3.74311070

ARNQ 4.87883800

ARNR 6.26127080

ARNS 4.29688547

ARNT 4.20509480

ARNV 3.57151975

ARNW 3.43183959

ARNY 3.76567003

ARPA 4.45190386

ARPC 3.26997310

ARPD 4.29187442

ARPE 4.69941379

ARPF 3.16503311

ARPG 3.84967038

ARPH 4.62923135

ARPI 3.61587311

ARPK 5.60427192

ARPL 3.77019496

ARPM 3.90322329

ARPN 4.42716378

ARPP 3.94461539

ARPQ 5.08623499

ARPR 6.80235665

ARPS 4.42376946

ARPT 4.41899668

ARPV 3.76852346

ARPW 3.54902367

ARPY 3.72734598

ARQA 4.60720285

ARQC 3.53645417

ARQD 4.56002985

ARQE 4.89807588

ARQF 3.35503649

ARQG 4.12503620

ARQH 4.79953886

ARQI 3.79696462

ARQK 5.68880463

ARQL 3.92584008

ARQM 4.01411173

ARQN 4.67003270

ARQP 4.09178994

ARQQ 5.21767573

ARQR 6.53524620

ARQS 4.56766576

ARQT 4.52719984

ARQV 3.82234610

ARQW 3.74231373

ARQY 3.91644055

ARRA 4.67847460

ARRC 3.48762995

ARRD 4.50770604

ARRE 4.89705441

ARRF 3.26776164

ARRG 4.10746591

ARRH 4.72698998

ARRI 3.68921093

ARRK 5.44122975

ARRL 3.88103702

ARRM 4.02120375

ARRN 4.51724064

ARRP 4.05438850

ARRQ 5.10190322

ARRR 6.31369554

ARRS 4.52191419

ARRT 4.49890784

ARRV 3.84611208

ARRW 3.68691683

ARRY 3.76474354

ARSA 4.78750200

ARSC 4.06396806

ARSD 4.77971116

ARSE 5.19837751

ARSF 3.74187908

ARSG 4.39560824

ARSH 5.23417658

ARSI 4.14569129

ARSK 6.31488291

ARSL 4.28853371

ARSM 4.43624184

ARSN 5.10536224

ARSP 4.44403261

ARSQ 5.59683350

ARSR 7.46656075

ARSS 4.92181948

ARST 4.84344342

ARSV 4.16871762

ARSW 4.17138079

ARSY 4.40308333

ARTA 4.50969134

ARTC 3.59671918

ARTD 4.50090008

ARTE 4.88733762

ARTF 3.35637467

ARTG 4.08182556

ARTH 4.87742129

ARTI 3.75802297

ARTK 5.87570103

ARTL 3.92781457

ARTM 4.03244353

ARTN 4.72832378

ARTP 4.15998858

ARTQ 5.25233704

ARTR 6.91335182

ARTS 4.59269403

ARTT 4.53762012

ARTV 3.81868350

ARTW 3.82276866

ARTY 3.98723538

ARVA 4.44911219

ARVC 3.52826142

ARVD 4.58443901

ARVE 5.02212639

ARVF 3.29889398

ARVG 4.07565059

ARVH 4.92875279

ARVI 3.57766724

ARVK 5.96315049

ARVL 3.77901687

ARVM 4.01564100

ARVN 4.76377222

ARVP 4.18230175

ARVQ 5.33812146

ARVR 6.98062369

ARVS 4.62644939

ARVT 4.54254178

ARVV 3.65071079

ARVW 3.80065264

ARVY 4.03134413

ARWA 3.50178055

ARWC 2.16802380

ARWD 3.46590483

ARWE 3.87788285

ARWF 2.27019513

ARWG 3.03477132

ARWH 3.70466889

ARWI 2.70163099

ARWK 4.64268364

ARWL 2.79211174

ARWM 3.00156881

ARWN 3.59561726

ARWP 3.06053573

ARWQ 4.20597599

ARWR 5.44455496

ARWS 3.54979331

ARWT 3.47276749

ARWV 2.75405280

ARWW 2.65064407

ARWY 2.79841018

ARYA 3.67762000

ARYC 2.73271528

ARYD 3.78941205

ARYE 4.20970269

ARYF 2.65068355

ARYG 3.27162854

ARYH 4.00646195

ARYI 3.10686980

ARYK 5.05768671

ARYL 3.17667558

ARYM 3.38904314

ARYN 4.09982170

ARYP 3.33577958

ARYQ 4.49875119

ARYR 5.69946275

ARYS 3.85076677

ARYT 3.77739732

ARYV 3.07563276

ARYW 2.96772982

ARYY 3.30454176

ASAA 7.01886809

ASAC 6.55443395

ASAD 6.47212978

ASAE 6.24197039

ASAF 4.98837329

ASAG 6.54861453

ASAH 6.01784609

ASAI 5.17979545

ASAK 6.23940732

ASAL 5.08722198

ASAM 5.55341440

ASAN 6.81165982

ASAP 6.25647373

ASAQ 6.23271881

ASAR 6.06210593

ASAS 9.80515580

ASAT 7.27565552

ASAV 5.52856520

ASAW 4.82235621

ASAY 5.29313413

ASCA 5.59830637

ASCC 2.63608508

ASCD 5.09949505

ASCE 5.03886711

ASCF 3.70874837

ASCG 4.77414026

ASCH 4.17467439

ASCI 4.04001860

ASCK 4.96262077

ASCL 3.94554302

ASCM 4.37657771

ASCN 5.31577094

ASCP 4.41718863

ASCQ 4.95264964

ASCR 4.76158155

ASCS 6.79063877

ASCT 5.85247169

ASCV 4.33593259

ASCW 3.30822124

ASCY 4.05753920

ASDA 4.85431853

ASDC 4.13069432

ASDD 4.66469489

ASDE 4.54504594

ASDF 3.25522921

ASDG 4.39370303

ASDH 4.18176090

ASDI 3.50405854

ASDK 4.49617677

ASDL 3.47081834

ASDM 3.76950172

ASDN 4.77009057

ASDP 4.32755256

ASDQ 4.53740476

ASDR 4.32780450

ASDS 5.78246094

ASDT 5.02527678

ASDV 3.76761696

ASDW 3.12648534

ASDY 3.54619226

ASEA 5.08065921

ASEC 4.44759734

ASED 4.90967509

ASEE 4.77390249

ASEF 3.47171459

ASEG 4.72565482

ASEH 4.42167585

ASEI 3.71697731

ASEK 4.72538617

ASEL 3.67197982

ASEM 3.95738451

ASEN 5.05237503

ASEP 4.57237789

ASEQ 4.71037279

ASER 4.53156130

ASES 6.05030616

ASET 5.26111909

ASEV 3.98148527

ASEW 3.29967659

ASEY 3.74271020

ASFA 4.33902787

ASFC 3.71743041

ASFD 4.16095409

ASFE 4.01695683

ASFF 2.88934402

ASFG 4.10393227

ASFH 3.66597343

ASFI 3.14485383

ASFK 4.01772066

ASFL 3.02908544

ASFM 3.36429065

ASFN 4.39881629

ASFP 3.79172909

ASFQ 4.01498182

ASFR 3.78447663

ASFS 5.41420696

ASFT 4.62632345

ASFV 3.29832470

ASFW 2.75884966

ASFY 3.14690803

ASGA 5.49575931

ASGC 4.64666833

ASGD 4.92369379

ASGE 4.83979240

ASGF 3.76623297

ASGG 4.87988854

ASGH 4.52887715

ASGI 4.01075006

ASGK 4.81315279

ASGL 3.93850221

ASGM 4.36960225

ASGN 5.26285857

ASGP 4.65092770

ASGQ 4.91687134

ASGR 4.64740066

ASGS 6.73093152

ASGT 5.68505038

ASGV 4.24589992

ASGW 3.54698156

ASGY 3.98888446

ASHA 4.58575644

ASHC 3.54977923

ASHD 4.39793733

ASHE 4.25390620

ASHF 3.00948864

ASHG 4.18642862

ASHH 3.76167359

ASHI 3.28777812

ASHK 4.22818691

ASHL 3.26207130

ASHM 3.46736270

ASHN 4.51568862

ASHP 4.04173429

ASHQ 4.25932019

ASHR 4.06480019

ASHS 5.57600568

ASHT 4.79077318

ASHV 3.52672117

ASHW 2.92576040

ASHY 3.31782928

ASIA 4.86771038

ASIC 4.40944084

ASID 4.70790228

ASIE 4.55416202

ASIF 3.40106333

ASIG 4.63279448

ASIH 4.26465426

ASII 3.52828989

ASIK 4.55353074

ASIL 3.47482771

ASIM 3.81358961

ASIN 4.95981799

ASIP 4.37986264

ASIQ 4.52781310

ASIR 4.30614538

ASIS 6.01124863

ASIT 5.13798771

ASIV 3.73445253

ASIW 3.29064492

ASIY 3.70424919

ASKA 4.92311212

ASKC 4.32747038

ASKD 4.83195571

ASKE 4.68368421

ASKF 3.42373385

ASKG 4.59712422

ASKH 4.30719449

ASKI 3.64463561

ASKK 4.65079379

ASKL 3.57106104

ASKM 3.85715341

ASKN 4.98846961

ASKP 4.46618088

ASKQ 4.57202881

ASKR 4.34061333

ASKS 5.91721033

ASKT 5.14067761

ASKV 3.84995168

ASKW 3.23361672

ASKY 3.73514139

ASLA 4.75627312

ASLC 4.21738084

ASLD 4.57285379

ASLE 4.40101530

ASLF 3.22668434

ASLG 4.51105689

ASLH 4.14394248

ASLI 3.40786518

ASLK 4.37456020

ASLL 3.33147039

ASLM 3.69002102

ASLN 4.76541899

ASLP 4.29678384

ASLQ 4.38680565

ASLR 4.19997566

ASLS 5.81055197

ASLT 5.00917531

ASLV 3.63719192

ASLW 3.12852635

ASLY 3.51758692

ASMA 4.89030189

ASMC 4.38378713

ASMD 4.66009075

ASME 4.49282425

ASMF 3.33581213

ASMG 4.65371530

ASMH 4.20073600

ASMI 3.52792385

ASMK 4.42837810

ASML 3.47168770

ASMM 3.75572881

ASMN 4.88009863

ASMP 4.38420261

ASMQ 4.44974181

ASMR 4.27519107

ASMS 5.97600956

ASMT 5.11284824

ASMV 3.76533579

ASMW 3.31868539

ASMY 3.66376831

ASNA 4.83409818

ASNC 4.23249973

ASND 4.67083810

ASNE 4.53020293

ASNF 3.38084998

ASNG 4.51205964

ASNH 4.19734373

ASNI 3.63611102

ASNK 4.54201196

ASNL 3.53003204

ASNM 3.80898773

ASNN 4.90695666

ASNP 4.33438191

ASNQ 4.54164712

ASNR 4.24761020

ASNS 5.86973772

ASNT 5.07051807

ASNV 3.82833351

ASNW 3.19966115

ASNY 3.68770930

ASPA 5.21393632

ASPC 4.35146566

ASPD 4.89702130

ASPE 4.74324760

ASPF 3.46701600

ASPG 4.68273406

ASPH 4.39045180

ASPI 3.69847728

ASPK 4.65340516

ASPL 3.63300138

ASPM 4.03491299

ASPN 5.06070421

ASPP 4.56448046

ASPQ 4.73256391

ASPR 4.49561376

ASPS 6.42088448

ASPT 5.46705768

ASPV 4.02559001

ASPW 3.27165913

ASPY 3.71511193

ASQA 5.02716471

ASQC 4.36801030

ASQD 4.86465071

ASQE 4.67535592

ASQF 3.48490411

ASQG 4.72377360

ASQH 4.38802443

ASQI 3.66663399

ASQK 4.60779136

ASQL 3.61424232

ASQM 3.89919216

ASQN 4.97057217

ASQP 4.57115765

ASQQ 4.68206975

ASQR 4.46699426

ASQS 5.96848908

ASQT 5.20039505

ASQV 3.92151774

ASQW 3.34095339

ASQY 3.73239574

ASRA 4.81515203

ASRC 4.12654598

ASRD 4.62326721

ASRE 4.46829415

ASRF 3.20877933

ASRG 4.44674722

ASRH 4.17634094

ASRI 3.40625183

ASRK 4.32597754

ASRL 3.38896788

ASRM 3.66953267

ASRN 4.68389227

ASRP 4.30093012

ASRQ 4.41188180

ASRR 4.27649499

ASRS 5.73864463

ASRT 4.96573115

ASRV 3.71823920

ASRW 3.11721399

ASRY 3.44233747

ASSA 5.82184791

ASSC 5.33011395

ASSD 5.36171128

ASSE 5.18531750

ASSF 4.14372959

ASSG 5.41441346

ASSH 4.95125770

ASSI 4.35358777

ASSK 5.20389865

ASSL 4.27585844

ASSM 4.61378867

ASSN 5.69258256

ASSP 5.13628595

ASSQ 5.20366212

ASSR 5.00468429

ASSS 6.99692945

ASST 5.98294660

ASSV 4.61642938

ASSW 3.93214435

ASSY 4.40212348

ASTA 5.37644302

ASTC 4.85142399

ASTD 5.04154433

ASTE 4.84508424

ASTF 3.76195913

ASTG 5.02244334

ASTH 4.58704264

ASTI 3.95219510

ASTK 4.83932759

ASTL 3.89529924

ASTM 4.20153215

ASTN 5.30794787

ASTP 4.76174746

ASTQ 4.85390336

ASTR 4.67068153

ASTS 6.49988458

ASTT 5.56003908

ASTV 4.20246702

ASTW 3.61872920

ASTY 3.99081495

ASVA 5.32958696

ASVC 4.76503935

ASVD 5.08369034

ASVE 4.91386812

ASVF 3.66710167

ASVG 5.01959211

ASVH 4.59954545

ASVI 3.80219352

ASVK 4.86800346

ASVL 3.75716700

ASVM 4.15003355

ASVN 5.31774569

ASVP 4.78221865

ASVQ 4.88121473

ASVR 4.68970767

ASVS 6.57667858

ASVT 5.57931723

ASVV 4.05127995

ASVW 3.55746315

ASVY 3.96831309

ASWA 4.02533531

ASWC 3.10692868

ASWD 3.78852933

ASWE 3.69107471

ASWF 2.48574723

ASWG 3.69970516

ASWH 3.30919619

ASWI 2.79988843

ASWK 3.62461606

ASWL 2.69548352

ASWM 3.07884911

ASWN 3.94995495

ASWP 3.46842985

ASWQ 3.71076314

ASWR 3.50434281

ASWS 4.98621343

ASWT 4.26323134

ASWV 3.01308353

ASWW 2.38081741

ASWY 2.74717574

ASYA 4.32889793

ASYC 3.66785640

ASYD 4.13682279

ASYE 4.04222959

ASYF 2.86859530

ASYG 4.05722141

ASYH 3.71167578

ASYI 3.15586532

ASYK 4.07382756

ASYL 3.04775988

ASYM 3.38019007

ASYN 4.43186030

ASYP 3.82547174

ASYQ 4.04446760

ASYR 3.79227653

ASYS 5.34356661

ASYT 4.58985498

ASYV 3.32035707

ASYW 2.71218738

ASYY 3.21108694

ATAA 6.53448807

ATAC 6.38901357

ATAD 6.03602028

ATAE 6.09705617

ATAF 5.24964991

ATAG 5.67917723

ATAH 5.81335981

ATAI 6.13485728

ATAK 6.14878164

ATAL 5.73155271

ATAM 6.05148335

ATAN 6.37582400

ATAP 5.86263418

ATAQ 6.11746743

ATAR 5.99111983

ATAS 7.27565552

ATAT 10.01029957

ATAV 6.49726583

ATAW 4.89377953

ATAY 5.33560946

ATCA 5.15768406

ATCC 2.53592951

ATCD 4.65098598

ATCE 4.82423666

ATCF 3.98770806

ATCG 3.97230379

ATCH 3.93051505

ATCI 4.80542980

ATCK 4.83199870

ATCL 4.50527686

ATCM 4.76978821

ATCN 4.92106987

ATCP 4.06237609

ATCQ 4.74038899

ATCR 4.64949185

ATCS 5.71931402

ATCT 7.05356414

ATCV 5.12184410

ATCW 3.38545107

ATCY 4.15354117

ATDA 4.58545519

ATDC 3.98143994

ATDD 4.33729688

ATDE 4.43482768

ATDF 3.48397800

ATDG 3.83500394

ATDH 4.04788759

ATDI 4.20001171

ATDK 4.43357761

ATDL 3.91749693

ATDM 4.07577784

ATDN 4.48161062

ATDP 4.02923037

ATDQ 4.42789410

ATDR 4.31146648

ATDS 4.99797230

ATDT 5.93896865

ATDV 4.46995162

ATDW 3.23052907

ATDY 3.62117135

ATEA 4.78269121

ATEC 4.29401925

ATED 4.56667846

ATEE 4.64501435

ATEF 3.72052581

ATEG 4.12540700

ATEH 4.26341978

ATEI 4.42490126

ATEK 4.64238016

ATEL 4.12571873

ATEM 4.31845899

ATEN 4.72354458

ATEP 4.25383731

ATEQ 4.59267509

ATER 4.48731866

ATES 5.22233952

ATET 6.21991168

ATEV 4.70577735

ATEW 3.42699732

ATEY 3.83415390

ATFA 4.07427515

ATFC 3.64168718

ATFD 3.81650547

ATFE 3.85848367

ATFF 3.19216715

ATFG 3.55146010

ATFH 3.50602483

ATFI 3.80077517

ATFK 3.91251735

ATFL 3.50143395

ATFM 3.68582747

ATFN 4.07141758

ATFP 3.50191230

ATFQ 3.89745627

ATFR 3.70651643

ATFS 4.62425192

ATFT 5.50857616

ATFV 3.95032955

ATFW 2.90918704

ATFY 3.24721170

ATGA 5.16217639

ATGC 4.49512608

ATGD 4.56779519

ATGE 4.72850987

ATGF 4.06506634

ATGG 4.19156754

ATGH 4.38132421

ATGI 4.78430148

ATGK 4.76597719

ATGL 4.51422048

ATGM 4.78606002

ATGN 4.93842349

ATGP 4.36904414

ATGQ 4.83452166

ATGR 4.62794471

ATGS 5.68883094

ATGT 6.88258457

ATGV 5.04241479

ATGW 3.64834073

ATGY 4.09620223

ATHA 4.27153843

ATHC 3.39413007

ATHD 4.04109434

ATHE 4.11319960

ATHF 3.25881199

ATHG 3.57687874

ATHH 3.56947768

ATHI 3.97839818

ATHK 4.08593764

ATHL 3.69596109

ATHM 3.81298989

ATHN 4.16143272

ATHP 3.73186133

ATHQ 4.11488994

ATHR 3.99214460

ATHS 4.77215862

ATHT 5.67017584

ATHV 4.21275771

ATHW 3.04392143

ATHY 3.32297313

ATIA 4.56632055

ATIC 4.29404353

ATID 4.32905337

ATIE 4.40689281

ATIF 3.71361557

ATIG 4.00046518

ATIH 4.11006162

ATII 4.22020267

ATIK 4.42917955

ATIL 3.98577396

ATIM 4.16765379

ATIN 4.60093543

ATIP 4.09043999

ATIQ 4.38552150

ATIR 4.20889755

ATIS 5.18294529

ATIT 6.12576848

ATIV 4.43212018

ATIW 3.41272023

ATIY 3.82993710

ATKA 4.59739123

ATKC 4.19868138

ATKD 4.45657577

ATKE 4.54286933

ATKF 3.65927088

ATKG 3.97134406

ATKH 4.13417111

ATKI 4.34825105

ATKK 4.55897310

ATKL 3.99438307

ATKM 4.16498142

ATKN 4.64228328

ATKP 4.12460815

ATKQ 4.44478723

ATKR 4.23086076

ATKS 5.09287925

ATKT 6.05887726

ATKV 4.53910825

ATKW 3.32506041

ATKY 3.80052278

ATLA 4.48024969

ATLC 4.15447015

ATLD 4.19481220

ATLE 4.23713004

ATLF 3.53467564

ATLG 3.92207106

ATLH 3.98206832

ATLI 4.06920751

ATLK 4.23865092

ATLL 3.82774185

ATLM 4.06148129

ATLN 4.41027202

ATLP 3.96473538

ATLQ 4.24937871

ATLR 4.11813383

ATLS 4.99860806

ATLT 5.93365074

ATLV 4.32534707

ATLW 3.24348364

ATLY 3.61376077

ATMA 4.57280828

ATMC 4.32258382

ATMD 4.26579633

ATME 4.31464130

ATMF 3.62669991

ATMG 4.00096609

ATMH 4.03152794

ATMI 4.21427285

ATMK 4.29033995

ATML 3.94545535

ATMM 4.10861365

ATMN 4.48978861

ATMP 4.06873669

ATMQ 4.29024487

ATMR 4.18456433

ATMS 5.08305555

ATMT 6.12291044

ATMV 4.46384771

ATMW 3.39741803

ATMY 3.73371093

ATNA 4.50389674

ATNC 4.07653255

ATND 4.31207968

ATNE 4.37694645

ATNF 3.58323007

ATNG 3.90625002

ATNH 3.99521344

ATNI 4.27414997

ATNK 4.45740631

ATNL 3.95729108

ATNM 4.12129638

ATNN 4.58475287

ATNP 4.01816763

ATNQ 4.40703305

ATNR 4.14609604

ATNS 5.04855384

ATNT 5.97467434

ATNV 4.47447227

ATNW 3.22894837

ATNY 3.73380400

ATPA 4.85817342

ATPC 4.18630121

ATPD 4.54196303

ATPE 4.58957880

ATPF 3.67162675

ATPG 4.02681321

ATPH 4.24429231

ATPI 4.44471416

ATPK 4.58878454

ATPL 4.14019519

ATPM 4.40358497

ATPN 4.74448994

ATPP 4.21498968

ATPQ 4.64857485

ATPR 4.49156862

ATPS 5.42623212

ATPT 6.58179487

ATPV 4.76230438

ATPW 3.39467290

ATPY 3.77573270

ATQA 4.70165589

ATQC 4.22829136

ATQD 4.49647885

ATQE 4.51547863

ATQF 3.70166886

ATQG 4.11075812

ATQH 4.19238824

ATQI 4.34771289

ATQK 4.50578152

ATQL 4.05456697

ATQM 4.20454575

ATQN 4.62045267

ATQP 4.24208391

ATQQ 4.53705000

ATQR 4.37732006

ATQS 5.14017257

ATQT 6.10755126

ATQV 4.59792394

ATQW 3.46293146

ATQY 3.77628751

ATRA 4.53512271

ATRC 4.02666797

ATRD 4.30268363

ATRE 4.36131813

ATRF 3.43452412

ATRG 3.87564702

ATRH 4.01183058

ATRI 4.08672769

ATRK 4.19647262

ATRL 3.86021033

ATRM 4.02311496

ATRN 4.31538275

ATRP 4.00137125

ATRQ 4.28441163

ATRR 4.23461738

ATRS 4.92972318

ATRT 5.86855753

ATRV 4.41796393

ATRW 3.26131663

ATRY 3.52756710

ATSA 5.39802327

ATSC 5.07593346

ATSD 4.95045590

ATSE 5.01217675

ATSF 4.33696244

ATSG 4.66368888

ATSH 4.71498738

ATSI 5.09572560

ATSK 5.08440116

ATSL 4.74068850

ATSM 4.98526390

ATSN 5.31199120

ATSP 4.75487997

ATSQ 5.04297563

ATSR 4.89410457

ATSS 5.92821080

ATST 7.14158558

ATSV 5.36243358

ATSW 3.99003549

ATSY 4.41010349

ATTA 5.05827629

ATTC 4.68294926

ATTD 4.70380172

ATTE 4.74485378

ATTF 3.99944887

ATTG 4.36876569

ATTH 4.43167355

ATTI 4.68912423

ATTK 4.77700692

ATTL 4.40089398

ATTM 4.57954576

ATTN 4.97700513

ATTP 4.47305927

ATTQ 4.76777730

ATTR 4.63792750

ATTS 5.57824921

ATTT 6.64333432

ATTV 4.95195472

ATTW 3.70112775

ATTY 4.05855881

ATVA 5.03825425

ATVC 4.67001837

ATVD 4.72723191

ATVE 4.80389526

ATVF 3.96762255

ATVG 4.35129544

ATVH 4.46233321

ATVI 4.58956911

ATVK 4.80903031

ATVL 4.33606330

ATVM 4.58195923

ATVN 4.96520051

ATVP 4.46577889

ATVQ 4.78893241

ATVR 4.68430790

ATVS 5.64985360

ATVT 6.74352514

ATVV 4.85876022

ATVW 3.67368994

ATVY 4.08952868

ATWA 3.86619704

ATWC 3.00804117

ATWD 3.49859769

ATWE 3.56409488

ATWF 2.81739462

ATWG 3.19003059

ATWH 3.23071489

ATWI 3.44666945

ATWK 3.56173161

ATWL 3.20080078

ATWM 3.48014807

ATWN 3.63947665

ATWP 3.25202063

ATWQ 3.66238386

ATWR 3.47655804

ATWS 4.26366911

ATWT 5.15564163

ATWV 3.66070683

ATWW 2.51469637

ATWY 2.84903605

ATYA 4.02538837

ATYC 3.60402187

ATYD 3.80264626

ATYE 3.89928469

ATYF 3.15268408

ATYG 3.49940411

ATYH 3.52951583

ATYI 3.78299061

ATYK 3.96427687

ATYL 3.50067780

ATYM 3.72149278

ATYN 4.10206955

ATYP 3.52854855

ATYQ 3.91677332

ATYR 3.69763544

ATYS 4.57732449

ATYT 5.44882472

ATYV 3.96223335

ATYW 2.79274276

ATYY 3.28923381

AVAA 6.62780828

AVAC 6.80852973

AVAD 4.49178135

AVAE 5.08698694

AVAF 6.36005365

AVAG 4.80559890

AVAH 5.17238907

AVAI 8.34552498

AVAK 5.16362579

AVAL 7.25503747

AVAM 6.83988051

AVAN 4.89958419

AVAP 5.37238513

AVAQ 5.21375777

AVAR 5.18313158

AVAS 5.52856520

AVAT 6.49726583

AVAV 10.09424632

AVAW 5.44854453

AVAY 5.75068379

AVCA 5.41350820

AVCC 2.90171595

AVCD 3.24765110

AVCE 3.91487611

AVCF 5.05504049

AVCG 3.28176067

AVCH 3.35895894

AVCI 6.78093484

AVCK 3.95279119

AVCL 5.93101918

AVCM 5.59465880

AVCN 3.61820399

AVCP 3.70703939

AVCQ 4.00685533

AVCR 3.93539832

AVCS 4.29643641

AVCT 5.17566158

AVCV 7.20487232

AVCW 3.89457273

AVCY 4.56236956

AVDA 4.38389951

AVDC 4.25486902

AVDD 2.84996092

AVDE 3.30584062

AVDF 4.26839427

AVDG 2.80533206

AVDH 3.32329442

AVDI 5.73011451

AVDK 3.35536368

AVDL 4.97573001

AVDM 4.55731357

AVDN 3.04084539

AVDP 3.38724020

AVDQ 3.41295369

AVDR 3.41354420

AVDS 3.51232862

AVDT 4.23733416

AVDV 6.08905249

AVDW 3.52231493

AVDY 3.81771827

AVEA 4.65339083

AVEC 4.62714504

AVED 3.08320477

AVEE 3.53060151

AVEF 4.53063294

AVEG 3.13804295

AVEH 3.56780567

AVEI 6.00711673

AVEK 3.57857524

AVEL 5.20221769

AVEM 4.80484271

AVEN 3.32136975

AVEP 3.65259316

AVEQ 3.61952700

AVER 3.61099588

AVES 3.77211526

AVET 4.49615781

AVEV 6.38144634

AVEW 3.75846275

AVEY 4.07625082

AVFA 4.31586917

AVFC 4.11238271

AVFD 2.54855647

AVFE 2.94829081

AVFF 4.19140580

AVFG 3.06259522

AVFH 3.03635581

AVFI 5.45331592

AVFK 3.08762022

AVFL 4.74486975

AVFM 4.42041314

AVFN 2.89848169

AVFP 3.13232202

AVFQ 3.15039145

AVFR 3.04029874

AVFS 3.43669015

AVFT 4.08291366

AVFV 5.67612179

AVFW 3.41341351

AVFY 3.71562199

AVGA 5.21262623

AVGC 4.79583163

AVGD 3.11583439

AVGE 3.71071630

AVGF 5.05891689

AVGG 3.36242976

AVGH 3.75314933

AVGI 6.58539031

AVGK 3.76248633

AVGL 5.78426789

AVGM 5.43731191

AVGN 3.57997901

AVGP 3.88316622

AVGQ 3.95919548

AVGR 3.78927108

AVGS 4.16891266

AVGT 4.94729311

AVGV 6.96334832

AVGW 4.09202566

AVGY 4.43798810

AVHA 4.22419413

AVHC 3.75296374

AVHD 2.66334568

AVHE 3.12785041

AVHF 4.10167549

AVHG 2.71397776

AVHH 2.96858223

AVHI 5.53041378

AVHK 3.10756604

AVHL 4.80856399

AVHM 4.38041019

AVHN 2.85745767

AVHP 3.19261357

AVHQ 3.24228665

AVHR 3.18385372

AVHS 3.39267696

AVHT 4.05597333

AVHV 5.84196305

AVHW 3.35885725

AVHY 3.62834683

AVIA 4.87869663

AVIC 4.82441261

AVID 3.01334790

AVIE 3.51196990

AVIF 4.80674071

AVIG 3.43740011

AVIH 3.59265703

AVII 6.05391637

AVIK 3.61610077

AVIL 5.36312209

AVIM 4.96865939

AVIN 3.40675441

AVIP 3.68134172

AVIQ 3.63521777

AVIR 3.53961039

AVIS 3.96595660

AVIT 4.61675463

AVIV 6.34418685

AVIW 3.97710647

AVIY 4.26890903

AVKA 4.47616412

AVKC 4.51734778

AVKD 2.93690713

AVKE 3.40379976

AVKF 4.48564973

AVKG 2.99119087

AVKH 3.41471026

AVKI 5.92058320

AVKK 3.48496334

AVKL 5.08050510

AVKM 4.65828231

AVKN 3.24786113

AVKP 3.53608520

AVKQ 3.45010008

AVKR 3.34899125

AVKS 3.63003053

AVKT 4.31886259

AVKV 6.21265015

AVKW 3.59346701

AVKY 4.02972118

AVLA 4.72706444

AVLC 4.63516280

AVLD 2.91928114

AVLE 3.32080730

AVLF 4.60918245

AVLG 3.35505872

AVLH 3.48372777

AVLI 5.85421373

AVLK 3.41747581

AVLL 5.16999901

AVLM 4.80789304

AVLN 3.23027237

AVLP 3.56645150

AVLQ 3.50005581

AVLR 3.44423520

AVLS 3.78290202

AVLT 4.47742235

AVLV 6.15310338

AVLW 3.85681175

AVLY 4.07462908

AVMA 4.74066429

AVMC 4.73370089

AVMD 2.94230949

AVME 3.37510161

AVMF 4.63987373

AVMG 3.37969349

AVMH 3.48505727

AVMI 5.91640154

AVMK 3.43006452

AVML 5.22363341

AVMM 4.80736512

AVMN 3.28160091

AVMP 3.62965835

AVMQ 3.51944942

AVMR 3.45483487

AVMS 3.80327966

AVMT 4.50683883

AVMV 6.26431310

AVMW 3.92601908

AVMY 4.13849872

AVNA 4.34089969

AVNC 4.29914889

AVND 2.81451357

AVNE 3.27099878

AVNF 4.32688915

AVNG 2.89215183

AVNH 3.29780605

AVNI 5.75979069

AVNK 3.37622307

AVNL 4.96143064

AVNM 4.58579298

AVNN 3.17642071

AVNP 3.36548369

AVNQ 3.39488916

AVNR 3.25142335

AVNS 3.58497241

AVNT 4.24407451

AVNV 6.02887696

AVNW 3.47217040

AVNY 3.87726874

AVPA 4.72453283

AVPC 4.39279929

AVPD 3.02466837

AVPE 3.50969954

AVPF 4.55670049

AVPG 3.05459500

AVPH 3.52994164

AVPI 6.10281474

AVPK 3.54913370

AVPL 5.30204608

AVPM 4.88393647

AVPN 3.27210923

AVPP 3.62566193

AVPQ 3.70194102

AVPR 3.63642681

AVPS 3.82639575

AVPT 4.61017553

AVPV 6.54392532

AVPW 3.73239598

AVPY 4.02690540

AVQA 4.55922972

AVQC 4.49885559

AVQD 2.99976881

AVQE 3.41304753

AVQF 4.50493873

AVQG 3.13919480

AVQH 3.48557719

AVQI 5.91688156

AVQK 3.45363168

AVQL 5.13735002

AVQM 4.71720824

AVQN 3.22275045

AVQP 3.62215874

AVQQ 3.53591071

AVQR 3.50317338

AVQS 3.68417817

AVQT 4.40174536

AVQV 6.25055463

AVQW 3.81486670

AVQY 3.99901925

AVRA 4.46304729

AVRC 4.39588950

AVRD 2.85230584

AVRE 3.33187906

AVRF 4.30221173

AVRG 2.94854504

AVRH 3.38001031

AVRI 5.70333696

AVRK 3.17984846

AVRL 4.99237406

AVRM 4.55882641

AVRN 3.00488663

AVRP 3.45801112

AVRQ 3.37552077

AVRR 3.36951275

AVRS 3.52146817

AVRT 4.25526491

AVRV 6.10599256

AVRW 3.61150622

AVRY 3.79750145

AVSA 5.29649419

AVSC 5.31931828

AVSD 3.43288491

AVSE 3.92269352

AVSF 5.20727124

AVSG 3.71279174

AVSH 4.01356406

AVSI 6.80302808

AVSK 4.00892561

AVSL 5.90820461

AVSM 5.53871386

AVSN 3.85285067

AVSP 4.15602304

AVSQ 4.08340666

AVSR 3.99603197

AVSS 4.34385816

AVST 5.14926974

AVSV 7.21844960

AVSW 4.30389502

AVSY 4.64895038

AVTA 5.01913134

AVTC 4.96759122

AVTD 3.18462782

AVTE 3.64667527

AVTF 4.89846141

AVTG 3.48127247

AVTH 3.74690682

AVTI 6.37901906

AVTK 3.71534594

AVTL 5.61843484

AVTM 5.19517515

AVTN 3.55194483

AVTP 3.89654336

AVTQ 3.81976721

AVTR 3.76562102

AVTS 4.06609643

AVTT 4.79346114

AVTV 6.75433148

AVTW 4.07978641

AVTY 4.33398122

AVVA 5.32295637

AVVC 5.23147182

AVVD 3.33350314

AVVE 3.86220580

AVVF 5.09606386

AVVG 3.70376719

AVVH 3.91945434

AVVI 6.53498495

AVVK 3.90881310

AVVL 5.80089611

AVVM 5.39736964

AVVN 3.66679061

AVVP 4.04242754

AVVQ 3.99857840

AVVR 3.96144847

AVVS 4.26644231

AVVT 5.02857037

AVVV 6.95812304

AVVW 4.26028835

AVVY 4.55437925

AVWA 4.00702043

AVWC 3.45998425

AVWD 2.25999336

AVWE 2.70409396

AVWF 3.76400722

AVWG 2.66693859

AVWH 2.75233150

AVWI 5.04664425

AVWK 2.74481121

AVWL 4.41807311

AVWM 4.11853489

AVWN 2.51702647

AVWP 2.78399210

AVWQ 2.97186763

AVWR 2.84434067

AVWS 3.12006708

AVWT 3.78161411

AVWV 5.30335366

AVWW 3.06105505

AVWY 3.26905883

AVYA 4.17333211

AVYC 4.00846796

AVYD 2.51243188

AVYE 2.97667136

AVYF 4.09638484

AVYG 2.88354215

AVYH 2.99132594

AVYI 5.39699771

AVYK 3.09636673

AVYL 4.66240947

AVYM 4.34303402

AVYN 2.89705958

AVYP 3.07293132

AVYQ 3.11543380

AVYR 2.99151051

AVYS 3.32089081

AVYT 3.98129402

AVYV 5.64462358

AVYW 3.17374994

AVYY 3.61943600

AWAA 5.07983881

AWAC 5.49386626

AWAD 4.29079217

AWAE 4.50490903

AWAF 7.52241693

AWAG 4.47633529

AWAH 6.00907935

AWAI 5.68706190

AWAK 4.66443464

AWAL 6.07021950

AWAM 5.92215299

AWAN 4.64513155

AWAP 4.61815422

AWAQ 4.88575566

AWAR 5.24412169

AWAS 4.82235621

AWAT 4.89377953

AWAV 5.44854453

AWAW 12.82203422

AWAY 7.67330472

AWCA 4.07925688

AWCC 1.80543617

AWCD 3.10556227

AWCE 3.50806704

AWCF 6.04807677

AWCG 3.03957432

AWCH 4.21667711

AWCI 4.69987544

AWCK 3.67626147

AWCL 4.95140076

AWCM 4.91252050

AWCN 3.55233747

AWCP 3.04185783

AWCQ 3.81078342

AWCR 4.09093214

AWCS 3.75588296

AWCT 3.97055695

AWCV 4.43345137

AWCW 9.17098860

AWCY 6.32017556

AWDA 3.36173989

AWDC 3.40569157

AWDD 2.62368347

AWDE 2.83853889

AWDF 5.30798469

AWDG 2.64077835

AWDH 3.87337769

AWDI 3.84948708

AWDK 2.85638139

AWDL 4.13520711

AWDM 4.02719567

AWDN 2.81221874

AWDP 2.85602944

AWDQ 3.07260679

AWDR 3.36151264

AWDS 3.00871727

AWDT 3.20165630

AWDV 3.67463587

AWDW 8.46243317

AWDY 5.38007850

AWEA 3.49616111

AWEC 3.63832704

AWED 2.75979112

AWEE 2.93698267

AWEF 5.49782597

AWEG 2.87953135

AWEH 4.07756379

AWEI 4.03658186

AWEK 2.96340249

AWEL 4.29087526

AWEM 4.19297414

AWEN 3.00360822

AWEP 3.03280573

AWEQ 3.16930874

AWER 3.51167362

AWES 3.17485851

AWET 3.29769657

AWEV 3.80660757

AWEW 8.77326139

AWEY 5.62248206

AWFA 3.47230999

AWFC 3.41819156

AWFD 2.62317682

AWFE 2.95264891

AWFF 5.07846770

AWFG 2.89391875

AWFH 3.80276682

AWFI 4.00679635

AWFK 2.98926612

AWFL 4.12433706

AWFM 4.04874527

AWFN 2.97310204

AWFP 2.94858924

AWFQ 3.23759730

AWFR 3.33493243

AWFS 3.16159805

AWFT 3.32772424

AWFV 3.74752908

AWFW 7.93839006

AWFY 5.29975690

AWGA 4.03087863

AWGC 3.82907992

AWGD 3.03254464

AWGE 3.37367191

AWGF 6.02697273

AWGG 3.18115830

AWGH 4.53279937

AWGI 4.61918222

AWGK 3.48097273

AWGL 4.89442372

AWGM 4.78889224

AWGN 3.42234471

AWGP 3.39700755

AWGQ 3.76217270

AWGR 3.94902452

AWGS 3.63875361

AWGT 3.75389824

AWGV 4.34701157

AWGW 9.36366473

AWGY 6.14802762

AWHA 3.33507442

AWHC 2.93776701

AWHD 2.60798271

AWHE 2.83741424

AWHF 5.17369076

AWHG 2.61390053

AWHH 3.60410097

AWHI 3.80139329

AWHK 2.84155557

AWHL 4.05913727

AWHM 3.88052780

AWHN 2.71536884

AWHP 2.85073075

AWHQ 3.04869573

AWHR 3.27508878

AWHS 3.00356040

AWHT 3.13809885

AWHV 3.58195280

AWHW 8.09499525

AWHY 5.22779222

AWIA 3.65823417

AWIC 3.77554041

AWID 2.92174068

AWIE 3.17731777

AWIF 5.58307452

AWIG 3.12596833

AWIH 4.21577817

AWII 4.21797556

AWIK 3.28381307

AWIL 4.44586392

AWIM 4.27886268

AWIN 3.26472104

AWIP 3.17299776

AWIQ 3.43132853

AWIR 3.67403532

AWIS 3.42959826

AWIT 3.51381016

AWIV 3.93178881

AWIW 8.73654875

AWIY 5.78327642

AWKA 3.30154659

AWKC 3.59251379

AWKD 2.63284408

AWKE 2.81243030

AWKF 5.41413843

AWKG 2.76920665

AWKH 3.88060112

AWKI 3.96269355

AWKK 2.90154026

AWKL 4.13524914

AWKM 4.08816696

AWKN 2.95078408

AWKP 2.90203605

AWKQ 3.01853837

AWKR 3.31405818

AWKS 3.05366666

AWKT 3.13015534

AWKV 3.65696925

AWKW 8.60630147

AWKY 5.55139395

AWLA 3.74601684

AWLC 3.69786246

AWLD 2.85306490

AWLE 3.01844004

AWLF 5.47657992

AWLG 3.13694932

AWLH 4.17848951

AWLI 4.17605445

AWLK 3.13427919

AWLL 4.42892228

AWLM 4.28354440

AWLN 3.14973533

AWLP 3.17444538

AWLQ 3.36603125

AWLR 3.62148249

AWLS 3.34591895

AWLT 3.50802122

AWLV 3.99343708

AWLW 8.44098081

AWLY 5.66218987

AWMA 3.72244046

AWMC 3.81663873

AWMD 2.90701214

AWME 3.13403623

AWMF 5.58780313

AWMG 3.14037200

AWMH 4.23024501

AWMI 4.14091347

AWMK 3.18506983

AWML 4.43319216

AWMM 4.24516185

AWMN 3.23022880

AWMP 3.20051622

AWMQ 3.38106831

AWMR 3.65272771

AWMS 3.39796343

AWMT 3.50058764

AWMV 3.94730578

AWMW 8.69301289

AWMY 5.75089964

AWNA 3.28220017

AWNC 3.47372592

AWND 2.64920213

AWNE 2.80859110

AWNF 5.28580524

AWNG 2.72878836

AWNH 3.90999697

AWNI 3.90797997

AWNK 2.89955794

AWNL 4.08178791

AWNM 4.02314605

AWNN 2.94263741

AWNP 2.84634005

AWNQ 3.04764781

AWNR 3.26810857

AWNS 3.04980839

AWNT 3.18159744

AWNV 3.62574873

AWNW 8.48511319

AWNY 5.41160442

AWPA 3.68062005

AWPC 3.54362184

AWPD 2.90233613

AWPE 3.12628016

AWPF 5.63236178

AWPG 3.02936380

AWPH 4.24477435

AWPI 4.11229043

AWPK 3.13219006

AWPL 4.40997820

AWPM 4.32409659

AWPN 3.15498014

AWPP 3.03339619

AWPQ 3.41214672

AWPR 3.68122270

AWPS 3.33270414

AWPT 3.40957033

AWPV 3.95901193

AWPW 8.99038160

AWPY 5.80194376

AWQA 3.50197496

AWQC 3.57069857

AWQD 2.79143614

AWQE 2.88395767

AWQF 5.47025382

AWQG 2.89425787

AWQH 4.04643929

AWQI 3.99387692

AWQK 2.90942991

AWQL 4.23194951

AWQM 4.05456129

AWQN 2.94415461

AWQP 3.03247040

AWQQ 3.09362600

AWQR 3.43677837

AWQS 3.11468636

AWQT 3.23185305

AWQV 3.78342454

AWQW 8.68748278

AWQY 5.60805191

AWRA 3.51122250

AWRC 3.53253916

AWRD 2.74347283

AWRE 2.92050933

AWRF 5.34823809

AWRG 2.83453767

AWRH 4.04184020

AWRI 3.92463625

AWRK 2.82186281

AWRL 4.20513602

AWRM 4.04197095

AWRN 2.87281928

AWRP 3.00684820

AWRQ 3.12346843

AWRR 3.41383750

AWRS 3.10239690

AWRT 3.23572513

AWRV 3.78067047

AWRW 8.32402422

AWRY 5.40845642

AWSA 4.01940235

AWSC 4.31445283

AWSD 3.22998051

AWSE 3.41053281

AWSF 6.19919580

AWSG 3.46378770

AWSH 4.65813555

AWSI 4.65570846

AWSK 3.56155075

AWSL 4.90408763

AWSM 4.77829564

AWSN 3.60725438

AWSP 3.53186430

AWSQ 3.72648536

AWSR 4.04902210

AWSS 3.72795892

AWST 3.80814137

AWSV 4.36217528

AWSW 9.81922755

AWSY 6.33454387

AWTA 3.77127239

AWTC 4.03482416

AWTD 3.01111662

AWTE 3.17024070

AWTF 5.83090602

AWTG 3.21609714

AWTH 4.41472530

AWTI 4.33985240

AWTK 3.25137097

AWTL 4.63691980

AWTM 4.48396169

AWTN 3.28717347

AWTP 3.26632607

AWTQ 3.49427830

AWTR 3.82952014

AWTS 3.49026690

AWTT 3.57701237

AWTV 4.09071155

AWTW 9.20212576

AWTY 5.96050562

AWVA 3.96362750

AWVC 4.05086267

AWVD 3.17330836

AWVE 3.39544491

AWVF 5.96090141

AWVG 3.35154243

AWVH 4.60112284

AWVI 4.43154733

AWVK 3.49279833

AWVL 4.74170423

AWVM 4.60583837

AWVN 3.45419805

AWVP 3.43893455

AWVQ 3.69231117

AWVR 4.01058628

AWVS 3.68058915

AWVT 3.80459990

AWVV 4.20260690

AWVW 9.32427619

AWVY 6.13764343

AWWA 3.61205155

AWWC 3.08817912

AWWD 2.58035497

AWWE 2.76452707

AWWF 4.84980349

AWWG 2.88864463

AWWH 3.77313282

AWWI 3.89932029

AWWK 2.84183094

AWWL 4.00383272

AWWM 3.98189738

AWWN 2.90300567

AWWP 2.89935142

AWWQ 3.14361474

AWWR 3.34927978

AWWS 3.19584514

AWWT 3.20554965

AWWV 3.71517990

AWWW 7.37485460

AWWY 5.03150482

AWYA 3.41567062

AWYC 3.33706572

AWYD 2.60530134

AWYE 2.98215307

AWYF 5.05767209

AWYG 2.74086501

AWYH 3.76945804

AWYI 3.89356276

AWYK 2.97418177

AWYL 4.06108566

AWYM 4.04321419

AWYN 2.95357054

AWYP 2.83333069

AWYQ 3.16465802

AWYR 3.26388418

AWYS 3.08111603

AWYT 3.25224970

AWYV 3.64545249

AWYW 7.83469070

AWYY 5.18689616

AYAA 5.40201196

AYAC 6.00005135

AYAD 4.79122089

AYAE 4.93679347

AYAF 8.28493555

AYAG 4.66100374

AYAH 7.17961060

AYAI 5.93711951

AYAK 5.11723863

AYAL 6.19787081

AYAM 6.05103853

AYAN 5.48000674

AYAP 4.71274486

AYAQ 5.33543450

AYAR 5.39884956

AYAS 5.29313413

AYAT 5.33560946

AYAV 5.75068379

AYAW 7.67330472

AYAY 11.46414199

AYCA 4.35465308

AYCC 2.15942132

AYCD 3.58232327

AYCE 3.90996898

AYCF 6.51542375

AYCG 3.12248027

AYCH 5.12874147

AYCI 4.79460176

AYCK 4.06059588

AYCL 4.94338776

AYCM 4.90725593

AYCN 4.16856128

AYCP 3.19828930

AYCQ 4.21539464

AYCR 4.21661870

AYCS 4.15419878

AYCT 4.26046889

AYCV 4.60676204

AYCW 5.88379902

AYCY 8.14415062

AYDA 3.56484347

AYDC 3.74998208

AYDD 2.94391124

AYDE 3.09541058

AYDF 5.72184004

AYDG 2.80774048

AYDH 4.71014494

AYDI 4.04135916

AYDK 3.23170343

AYDL 4.16322902

AYDM 4.06635142

AYDN 3.39929620

AYDP 2.92009689

AYDQ 3.37915148

AYDR 3.46162839

AYDS 3.36123236

AYDT 3.46687168

AYDV 3.91012711

AYDW 5.39437250

AYDY 7.13637480

AYEA 3.73922151

AYEC 4.07124364

AYED 3.14578667

AYEE 3.23969508

AYEF 6.02397796

AYEG 3.05577130

AYEH 4.96455597

AYEI 4.24067976

AYEK 3.37927668

AYEL 4.35661961

AYEM 4.26441169

AYEN 3.65825959

AYEP 3.09346255

AYEQ 3.53242406

AYER 3.64583296

AYES 3.57429243

AYET 3.66257313

AYEV 4.09849678

AYEW 5.57599673

AYEY 7.40799763

AYFA 3.53637744

AYFC 3.49159688

AYFD 2.92023181

AYFE 3.05818298

AYFF 5.24859981

AYFG 2.88241042

AYFH 4.42400462

AYFI 3.93336028

AYFK 3.19349375

AYFL 3.97225403

AYFM 3.97530919

AYFN 3.43167958

AYFP 2.80445017

AYFQ 3.40733521

AYFR 3.37306459

AYFS 3.34745398

AYFT 3.43419725

AYFV 3.76627663

AYFW 5.09996218

AYFY 6.61341878

AYGA 4.20898133

AYGC 4.14696527

AYGD 3.38396293

AYGE 3.67021288

AYGF 6.50345988

AYGG 3.24162351

AYGH 5.40277977

AYGI 4.70811073

AYGK 3.77901778

AYGL 4.88740260

AYGM 4.82583372

AYGN 4.07200205

AYGP 3.36955768

AYGQ 4.11837853

AYGR 4.03152081

AYGS 4.00414129

AYGT 4.06812322

AYGV 4.51005837

AYGW 6.07672404

AYGY 8.07917798

AYHA 3.47022497

AYHC 3.23984007

AYHD 2.88818359

AYHE 3.01581437

AYHF 5.48678576

AYHG 2.69916220

AYHH 4.32074183

AYHI 3.90318041

AYHK 3.10124580

AYHL 4.04543586

AYHM 3.88109231

AYHN 3.32643094

AYHP 2.83957896

AYHQ 3.29333543

AYHR 3.38254883

AYHS 3.29194515

AYHT 3.35355060

AYHV 3.75298868

AYHW 5.15802142

AYHY 6.81906279

AYIA 3.87011901

AYIC 4.12276639

AYID 3.27517195

AYIE 3.46916616

AYIF 5.89372302

AYIG 3.21559054

AYIH 5.02319483

AYII 4.26699047

AYIK 3.63795974

AYIL 4.40464725

AYIM 4.31454833

AYIN 3.85424997

AYIP 3.20849606

AYIQ 3.76609021

AYIR 3.80286233

AYIS 3.74156934

AYIT 3.77587813

AYIV 4.09076391

AYIW 5.67014890

AYIY 7.37211097

AYKA 3.66522755

AYKC 3.97475720

AYKD 3.01883962

AYKE 3.14463303

AYKF 5.90961245

AYKG 2.96720386

AYKH 4.83221642

AYKI 4.16724222

AYKK 3.29467199

AYKL 4.25582369

AYKM 4.12560410

AYKN 3.59225667

AYKP 3.02686960

AYKQ 3.39440969

AYKR 3.52872080

AYKS 3.47457021

AYKT 3.55216484

AYKV 3.97744739

AYKW 5.48354908

AYKY 7.21891986

AYLA 3.81957114

AYLC 3.94635448

AYLD 3.13803288

AYLE 3.29041490

AYLF 5.69003992

AYLG 3.11907122

AYLH 4.90075800

AYLI 4.17152296

AYLK 3.41558983

AYLL 4.25606211

AYLM 4.20419967

AYLN 3.70169662

AYLP 3.07136384

AYLQ 3.58327234

AYLR 3.65628443

AYLS 3.59441097

AYLT 3.70562899

AYLV 4.04060579

AYLW 5.49816641

AYLY 7.13360042

AYMA 3.89278424

AYMC 4.07818691

AYMD 3.22589349

AYME 3.38532688

AYMF 5.87150888

AYMG 3.26736302

AYMH 5.02042047

AYMI 4.25309748

AYMK 3.48185741

AYML 4.40593995

AYMM 4.23529090

AYMN 3.79422265

AYMP 3.19948864

AYMQ 3.64955180

AYMR 3.73788355

AYMS 3.67162603

AYMT 3.71774169

AYMV 4.11691263

AYMW 5.66319383

AYMY 7.33049918

AYNA 3.55796960

AYNC 3.81532735

AYND 3.03033192

AYNE 3.14643061

AYNF 5.79209501

AYNG 2.94790578

AYNH 4.75476680

AYNI 4.12502531

AYNK 3.32978426

AYNL 4.22822866

AYNM 4.11571993

AYNN 3.58864917

AYNP 2.99981896

AYNQ 3.45608074

AYNR 3.48492837

AYNS 3.47594332

AYNT 3.60020193

AYNV 3.90571109

AYNW 5.41592659

AYNY 7.17015242

AYPA 3.84212877

AYPC 3.85530768

AYPD 3.23186472

AYPE 3.34127527

AYPF 6.12675230

AYPG 3.07534290

AYPH 5.06762692

AYPI 4.32606624

AYPK 3.46851654

AYPL 4.46030651

AYPM 4.37419955

AYPN 3.73962449

AYPP 3.13855742

AYPQ 3.72663630

AYPR 3.77952519

AYPS 3.68139499

AYPT 3.81325180

AYPV 4.19407608

AYPW 5.67340978

AYPY 7.73193107

AYQA 3.71947928

AYQC 3.96120960

AYQD 3.13155863

AYQE 3.18836854

AYQF 5.97610193

AYQG 3.11550866

AYQH 4.92409362

AYQI 4.19897635

AYQK 3.34906342

AYQL 4.33029195

AYQM 4.17577265

AYQN 3.60903181

AYQP 3.09908229

AYQQ 3.50770408

AYQR 3.63592048

AYQS 3.51707230

AYQT 3.62082630

AYQV 4.03663897

AYQW 5.61982906

AYQY 7.34781608

AYRA 3.65343333

AYRC 3.82661221

AYRD 3.00535768

AYRE 3.09753898

AYRF 5.73944428

AYRG 2.91637982

AYRH 4.78772405

AYRI 4.03643960

AYRK 3.14520882

AYRL 4.19230932

AYRM 4.07545007

AYRN 3.44993405

AYRP 2.96004725

AYRQ 3.38901561

AYRR 3.46462136

AYRS 3.41650343

AYRT 3.52811358

AYRV 3.95666969

AYRW 5.38551773

AYRY 7.02265822

AYSA 4.30423205

AYSC 4.71977985

AYSD 3.68925070

AYSE 3.80798747

AYSF 6.81220700

AYSG 3.62160040

AYSH 5.70719557

AYSI 4.84387909

AYSK 3.97448372

AYSL 5.03862346

AYSM 4.89916323

AYSN 4.31375394

AYSP 3.64724753

AYSQ 4.18657040

AYSR 4.23018007

AYSS 4.16956133

AYST 4.21327845

AYSV 4.66276151

AYSW 6.31842930

AYSY 8.46589628

AYTA 4.00388137

AYTC 4.38459952

AYTD 3.38977019

AYTE 3.48252656

AYTF 6.34945501

AYTG 3.34893383

AYTH 5.32750980

AYTI 4.52074853

AYTK 3.66111977

AYTL 4.70110994

AYTM 4.55235831

AYTN 3.99826439

AYTP 3.37159588

AYTQ 3.87672478

AYTR 3.93260426

AYTS 3.86012078

AYTT 3.91808485

AYTV 4.35156077

AYTW 5.95606856

AYTY 7.84933090

AYVA 4.18903591

AYVC 4.44195909

AYVD 3.56711283

AYVE 3.73456705

AYVF 6.38362813

AYVG 3.45171640

AYVH 5.49553624

AYVI 4.57074070

AYVK 3.88376583

AYVL 4.74429895

AYVM 4.66647037

AYVN 4.11833108

AYVP 3.49220295

AYVQ 4.08505145

AYVR 4.13508958

AYVS 4.04011249

AYVT 4.09999290

AYVV 4.40242177

AYVW 6.06757653

AYVY 8.00595743

AYWA 3.39542084

AYWC 3.01474305

AYWD 2.70855275

AYWE 2.86741377

AYWF 4.96852764

AYWG 2.70331568

AYWH 4.15912837

AYWI 3.70320170

AYWK 2.94593305

AYWL 3.73467217

AYWM 3.82960588

AYWN 3.15927504

AYWP 2.54351562

AYWQ 3.22545689

AYWR 3.16759082

AYWS 3.14410413

AYWT 3.25665867

AYWV 3.59715367

AYWW 4.60934228

AYWY 6.15148210

AYYA 3.53752920

AYYC 3.51006754

AYYD 2.91620597

AYYE 3.09811495

AYYF 5.36061190

AYYG 2.83343889

AYYH 4.42782585

AYYI 3.95819059

AYYK 3.21801242

AYYL 4.02145229

AYYM 3.98863714

AYYN 3.42921993

AYYP 2.84051308

AYYQ 3.36566112

AYYR 3.33587854

AYYS 3.32960804

AYYT 3.44584996

AYYV 3.79019658

AYYW 5.06763757

AYYY 6.60037795

CAAA 6.90403691

CAAC 6.01767543

CAAD 4.44293464

CAAE 4.66896649

CAAF 4.46565392

CAAG 5.37046872

CAAH 4.50044847

CAAI 4.98917051

CAAK 4.56375451

CAAL 4.85741026

CAAM 4.92380413

CAAN 4.52349621

CAAP 4.90683947

CAAQ 4.61646614

CAAR 4.52120009

CAAS 5.59830637

CAAT 5.15768406

CAAV 5.41350820

CAAW 4.07925688

CAAY 4.35465308

CACA 12.60214257

CACC 6.70179922

CACD 7.94995348

CACE 8.15564820

CACF 7.50988886

CACG 9.12089630

CACH 7.36154796

CACI 8.16996393

CACK 8.17938778

CACL 7.94258303

CACM 8.22014583

CACN 8.08784394

CACP 8.70612494

CACQ 8.18931033

CACR 7.95255536

CACS 9.50555642

CACT 8.88758649

CACV 9.03492451

CACW 6.78857920

CACY 7.57846005

CADA 4.51450984

CADC 3.80988189

CADD 2.71892355

CADE 2.91261436

CADF 2.60188527

CADG 3.12304155

CADH 2.71549883

CADI 3.01142756

CADK 2.80394430

CADL 2.90335739

CADM 3.06510257

CADN 2.79875256

CADP 3.22352246

CADQ 2.86017947

CADR 2.74753212

CADS 3.55231493

CADT 3.22391694

CADV 3.35864002

CADW 2.40972016

CADY 2.57280642

CAEA 4.48193521

CAEC 3.78193015

CAED 2.77441274

CAEE 2.90947968

CAEF 2.57404122

CAEG 3.23002848

CAEH 2.71337225

CAEI 3.03084429

CAEK 2.82257521

CAEL 2.87022323

CAEM 2.99644809

CAEN 2.79451427

CAEP 3.09966401

CAEQ 2.81203661

CAER 2.72555820

CAES 3.48641650

CAET 3.15436430

CAEV 3.32616296

CAEW 2.33868053

CAEY 2.55110608

CAFA 6.00812266

CAFC 4.95400424

CAFD 3.86063898

CAFE 4.15285855

CAFF 3.73083607

CAFG 4.61022530

CAFH 3.82119131

CAFI 4.32246467

CAFK 4.06885293

CAFL 4.10371329

CAFM 4.25794154

CAFN 3.98128539

CAFP 4.14085377

CAFQ 4.10762444

CAFR 3.90207235

CAFS 4.87626939

CAFT 4.49977991

CAFV 4.59153256

CAFW 3.30208482

CAFY 3.69900317

CAGA 5.69187061

CAGC 4.46684723

CAGD 3.28281551

CAGE 3.62183664

CAGF 3.60308813

CAGG 4.05939250

CAGH 3.38177563

CAGI 4.01562356

CAGK 3.49910388

CAGL 3.92264410

CAGM 4.03878014

CAGN 3.46843714

CAGP 3.75333377

CAGQ 3.64708542

CAGR 3.40586284

CAGS 4.45388577

CAGT 4.05124373

CAGV 4.28943255

CAGW 3.02022533

CAGY 3.34266337

CAHA 5.49113050

CAHC 4.16340373

CAHD 3.44018839

CAHE 3.63652223

CAHF 3.31421172

CAHG 3.99023499

CAHH 3.22290301

CAHI 3.77716970

CAHK 3.54037383

CAHL 3.64520192

CAHM 3.72784037

CAHN 3.44619749

CAHP 3.80102250

CAHQ 3.58754247

CAHR 3.46618888

CAHS 4.29397143

CAHT 3.96485009

CAHV 4.10040431

CAHW 2.91339513

CAHY 3.19964989

CAIA 6.33737944

CAIC 5.45574299

CAID 4.16075348

CAIE 4.43430892

CAIF 4.09335210

CAIG 4.94143074

CAIH 4.17843261

CAII 4.52665478

CAIK 4.38219970

CAIL 4.41612408

CAIM 4.49274726

CAIN 4.29639599

CAIP 4.47028162

CAIQ 4.38750458

CAIR 4.19632542

CAIS 5.20789365

CAIT 4.78188684

CAIV 4.86094741

CAIW 3.77704886

CAIY 4.04201524

CAKA 4.77205182

CAKC 4.03420428

CAKD 2.93958826

CAKE 3.12201053

CAKF 2.75299366

CAKG 3.37072588

CAKH 2.85114545

CAKI 3.23850587

CAKK 3.01632053

CAKL 3.04904235

CAKM 3.08630835

CAKN 3.03604007

CAKP 3.16720113

CAKQ 3.04754475

CAKR 2.83973287

CAKS 3.71841559

CAKT 3.32070238

CAKV 3.47060282

CAKW 2.45492379

CAKY 2.78213520

CALA 6.14052848

CALC 5.24766840

CALD 4.06570093

CALE 4.27618765

CALF 3.87910132

CALG 4.77431874

CALH 4.06161465

CALI 4.40929792

CALK 4.19070002

CALL 4.24210036

CALM 4.34296401

CALN 4.13159361

CALP 4.35051653

CALQ 4.20594502

CALR 4.09485409

CALS 5.00279449

CALT 4.63159421

CALV 4.72898826

CALW 3.55176174

CALY 3.78780360

CAMA 6.12378367

CAMC 5.22063152

CAMD 3.94085168

CAME 4.17213240

CAMF 3.86894671

CAMG 4.73974351

CAMH 3.97349977

CAMI 4.31612185

CAMK 4.04764916

CAML 4.21516900

CAMM 4.23248659

CAMN 4.05036164

CAMP 4.31681792

CAMQ 4.06127089

CAMR 3.96454289

CAMS 4.97036273

CAMT 4.52871352

CAMV 4.67524097

CAMW 3.55885149

CAMY 3.81511282

CANA 5.44611819

CANC 4.51451822

CAND 3.32047159

CANE 3.50209050

CANF 3.29488867

CANG 3.95745943

CANH 3.26447987

CANI 3.71966025

CANK 3.38993596

CANL 3.53829423

CANM 3.63530325

CANN 3.47451724

CANP 3.66260418

CANQ 3.44477823

CANR 3.25198754

CANS 4.28706747

CANT 3.81898694

CANV 4.02191982

CANW 2.85328371

CANY 3.19574343

CAPA 4.86151621

CAPC 3.87655348

CAPD 2.86327512

CAPE 3.03673728

CAPF 2.77782122

CAPG 3.37291679

CAPH 2.92775401

CAPI 3.28606391

CAPK 2.92381392

CAPL 3.15440323

CAPM 3.33067905

CAPN 2.95776932

CAPP 3.21544110

CAPQ 2.99719207

CAPR 2.84555530

CAPS 3.77941659

CAPT 3.36803168

CAPV 3.58989819

CAPW 2.48014216

CAPY 2.68878469

CAQA 4.91191156

CAQC 4.12988575

CAQD 3.13584797

CAQE 3.20282377

CAQF 2.95108762

CAQG 3.60526650

CAQH 3.02138514

CAQI 3.33422950

CAQK 3.09213284

CAQL 3.18968607

CAQM 3.26281033

CAQN 3.05281520

CAQP 3.42989464

CAQQ 3.10869573

CAQR 3.03376759

CAQS 3.82865293

CAQT 3.49701814

CAQV 3.67039331

CAQW 2.86711505

CAQY 2.90615089

CARA 5.07602965

CARC 4.21866657

CARD 3.17699321

CARE 3.37055180

CARF 2.97233557

CARG 3.58304627

CARH 3.06410083

CARI 3.39147457

CARK 3.16727514

CARL 3.22585415

CARM 3.30184173

CARN 3.15650445

CARP 3.47161112

CARQ 3.22648586

CARR 3.09796578

CARS 3.92988403

CART 3.57827527

CARV 3.76334974

CARW 2.65937612

CARY 2.91873400

CASA 6.50117350

CASC 5.57236036

CASD 4.03919454

CASE 4.24020522

CASF 4.06715918

CASG 4.89580364

CASH 4.03568297

CASI 4.59989647

CASK 4.12896156

CASL 4.40060330

CASM 4.53043211

CASN 4.14247832

CASP 4.51924839

CASQ 4.15631311

CASR 4.02082824

CASS 5.18490700

CAST 4.74452468

CASV 4.96392876

CASW 3.60532569

CASY 3.96583685

CATA 6.31489259

CATC 5.41479372

CATD 3.94782925

CATE 4.14446538

CATF 3.92482555

CATG 4.79102768

CATH 3.92225750

CATI 4.44398888

CATK 4.05570985

CATL 4.29764763

CATM 4.37458405

CATN 4.03952584

CATP 4.38515297

CATQ 4.10344155

CATR 3.98305886

CATS 5.00162071

CATT 4.54402674

CATV 4.77779231

CATW 3.55428179

CATY 3.81460735

CAVA 6.74883570

CAVC 5.74968093

CAVD 4.39228381

CAVE 4.70227917

CAVF 4.21589279

CAVG 5.20862277

CAVH 4.40685631

CAVI 4.71426338

CAVK 4.54400092

CAVL 4.62592070

CAVM 4.76666679

CAVN 4.44298719

CAVP 4.82188701

CAVQ 4.60267591

CAVR 4.46544674

CAVS 5.42045466

CAVT 4.97619087

CAVV 5.13002718

CAVW 3.93914091

CAVY 4.14873751

CAWA 5.37681843

CAWC 4.01740925

CAWD 3.39806107

CAWE 3.55576096

CAWF 3.10632448

CAWG 3.96218964

CAWH 3.29721620

CAWI 3.71892593

CAWK 3.47549094

CAWL 3.55654557

CAWM 3.73200197

CAWN 3.40350223

CAWP 3.52510622

CAWQ 3.53431070

CAWR 3.35488265

CAWS 4.26905891

CAWT 3.95501489

CAWV 4.02021237

CAWW 2.80282183

CAWY 2.95271550

CAYA 5.91415551

CAYC 4.77110771

CAYD 3.73059866

CAYE 4.03526767

CAYF 3.53047497

CAYG 4.39343647

CAYH 3.68669981

CAYI 4.12966538

CAYK 3.98559026

CAYL 3.96368066

CAYM 4.11291917

CAYN 3.86265931

CAYP 4.04786970

CAYQ 3.96350510

CAYR 3.73426903

CAYS 4.72232447

CAYT 4.34267276

CAYV 4.45510632

CAYW 3.14499856

CAYY 3.46024929

CCAA 2.96546567

CCAC 6.61099990

CCAD 1.10332104

CCAE 0.86266302

CCAF 2.29710836

CCAG 1.88526858

CCAH 1.79694616

CCAI 2.59221223

CCAK 1.07378983

CCAL 2.39474151

CCAM 2.32487641

CCAN 1.74494237

CCAP 1.30434961

CCAQ 1.21333389

CCAR 1.22893371

CCAS 2.63608508

CCAT 2.53592951

CCAV 2.90171595

CCAW 1.80543617

CCAY 2.15942132

CCCA 6.70179922

CCCC 14.78281871

CCCD 6.58392599

CCCE 4.96213620

CCCF 5.94506531

CCCG 5.86867144

CCCH 6.92721752

CCCI 5.87097180

CCCK 5.20063726

CCCL 5.67414395

CCCM 5.74054444

CCCN 5.98499095

CCCP 5.11310770

CCCQ 5.33045412

CCCR 5.47239176

CCCS 7.25877282

CCCT 6.68617296

CCCV 6.37117058

CCCW 5.62152722

CCCY 6.34541189

CCDA 1.19434502

CCDC 5.59507474

CCDD 0.88887206

CCDE -0.11758032

CCDF 0.65240559

CCDG 0.73463171

CCDH 0.80326351

CCDI 0.95837148

CCDK 0.09014667

CCDL 0.67153516

CCDM 0.71820094

CCDN 0.72270484

CCDP 0.06447194

CCDQ 0.45495888

CCDR -0.01355912

CCDS 2.26943622

CCDT 2.00240441

CCDV 1.03600000

CCDW 0.59984655

CCDY 0.67636626

CCEA 0.87199917

CCEC 4.75629588

CCED -0.03338952

CCEE -0.50781128

CCEF 0.55501345

CCEG 0.19124915

CCEH 0.59864406

CCEI 0.83450233

CCEK -0.04119141

CCEL 0.47676098

CCEM 0.58598133

CCEN 0.70013472

CCEP -0.21729530

CCEQ -0.15830167

CCER -0.10182213

CCES 0.98378694

CCET 0.84699103

CCEV 0.97656146

CCEW 0.80891484

CCEY 0.65504722

CCFA 2.32071835

CCFC 5.74654625

CCFD 0.66102189

CCFE 0.62730511

CCFF 1.61610788

CCFG 1.56314107

CCFH 1.49109796

CCFI 1.95078237

CCFK 0.85786420

CCFL 1.72419024

CCFM 1.69118164

CCFN 1.40949648

CCFP 0.79785659

CCFQ 1.03669137

CCFR 1.02533343

CCFS 2.10184166

CCFT 1.96108110

CCFV 2.09043476

CCFW 1.13221508

CCFY 1.59147048

CCGA 1.95520769

CCGC 5.83147703

CCGD 0.21099591

CCGE 0.18440283

CCGF 1.50965471

CCGG 0.82539242

CCGH 1.02076439

CCGI 1.66129988

CCGK 0.58969460

CCGL 1.56879640

CCGM 1.71546533

CCGN 1.01855223

CCGP 0.52190935

CCGQ 0.85169521

CCGR 0.49985214

CCGS 1.74928628

CCGT 1.48648261

CCGV 1.83616500

CCGW 1.25828620

CCGY 1.38209286

CCHA 1.84540726

CCHC 6.26604988

CCHD 0.61824195

CCHE 0.41768563

CCHF 1.28599252

CCHG 1.08402705

CCHH 0.71389588

CCHI 1.45937982

CCHK 0.47116030

CCHL 1.21935518

CCHM 1.17535461

CCHN 0.92469942

CCHP 0.45802532

CCHQ 0.65213797

CCHR 0.52138569

CCHS 1.74344718

CCHT 1.39771755

CCHV 1.74327801

CCHW 1.19098436

CCHY 1.30215241

CCIA 2.53673110

CCIC 5.75075930

CCID 0.81775630

CCIE 0.75496687

CCIF 1.83941809

CCIG 1.57094743

CCIH 1.49408989

CCII 2.12568214

CCIK 0.94672161

CCIL 1.95729847

CCIM 1.91472640

CCIN 1.36453185

CCIP 0.98887895

CCIQ 1.02766389

CCIR 0.97261008

CCIS 2.24221173

CCIT 2.05947607

CCIV 2.35078830

CCIW 1.43314972

CCIY 1.75800596

CCKA 1.12183488

CCKC 5.26232484

CCKD -0.03280076

CCKE -0.24022467

CCKF 0.81267786

CCKG 0.49127140

CCKH 0.54377218

CCKI 0.99341688

CCKK 0.04759871

CCKL 0.72671874

CCKM 0.64507933

CCKN 0.75841414

CCKP 0.11136663

CCKQ 0.01285606

CCKR -0.04801563

CCKS 1.02674835

CCKT 0.77770086

CCKV 1.14269262

CCKW 0.68188695

CCKY 1.15828827

CCLA 2.31303010

CCLC 5.59966432

CCLD 0.62082414

CCLE 0.48437876

CCLF 1.75143407

CCLG 1.37360724

CCLH 1.33021510

CCLI 1.93428849

CCLK 0.73132366

CCLL 1.75491446

CCLM 1.73665031

CCLN 1.29269710

CCLP 0.85049819

CCLQ 0.78948664

CCLR 0.89195009

CCLS 2.03220057

CCLT 1.87783550

CCLV 2.15058779

CCLW 1.19192106

CCLY 1.57353973

CCMA 2.23277330

CCMC 5.60040115

CCMD 0.63347841

CCME 0.52080473

CCMF 1.57335061

CCMG 1.33632355

CCMH 1.17308649

CCMI 1.96567986

CCMK 0.70375001

CCML 1.87723827

CCMM 1.73046589

CCMN 1.13357283

CCMP 0.87820012

CCMQ 0.71995029

CCMR 0.73379090

CCMS 1.93429148

CCMT 1.81253471

CCMV 2.18130739

CCMW 1.49248632

CCMY 1.69842311

CCNA 1.69633645

CCNC 5.81276692

CCND 0.82173280

CCNE 0.40381283

CCNF 1.43275314

CCNG 1.13478453

CCNH 0.99458131

CCNI 1.53127502

CCNK 0.54199431

CCNL 1.30651858

CCNM 1.20834836

CCNN 1.47985354

CCNP 0.51872965

CCNQ 0.46938672

CCNR 0.58204420

CCNS 1.89855744

CCNT 1.67573305

CCNV 1.60351524

CCNW 0.93822787

CCNY 1.28645453

CCPA 1.17128585

CCPC 5.37392392

CCPD 0.24274397

CCPE 0.15333464

CCPF 0.65547984

CCPG 0.27199439

CCPH 0.50490353

CCPI 1.05316886

CCPK -0.13358109

CCPL 0.78068746

CCPM 0.78044926

CCPN 0.80401924

CCPP -0.08143979

CCPQ 0.10485630

CCPR 0.00420346

CCPS 1.12766038

CCPT 0.90066253

CCPV 1.15205499

CCPW 0.34269389

CCPY 0.61490155

CCQA 1.13783867

CCQC 5.10235518

CCQD 0.62584554

CCQE -0.08252319

CCQF 0.83821737

CCQG 0.57441087

CCQH 0.51237801

CCQI 0.99033035

CCQK -0.02968478

CCQL 0.75630491

CCQM 0.67250310

CCQN 0.48240753

CCQP 0.11111661

CCQQ 0.01188945

CCQR 0.07689157

CCQS 1.29374393

CCQT 0.90013825

CCQV 1.18899064

CCQW 0.55974178

CCQY 0.88828423

CCRA 1.21420042

CCRC 5.49920912

CCRD 0.04740706

CCRE -0.13426753

CCRF 0.90290573

CCRG 0.55529029

CCRH 0.56796592

CCRI 1.11526223

CCRK 0.07921490

CCRL 0.79516055

CCRM 0.95139974

CCRN 0.48234886

CCRP 0.29668815

CCRQ 0.17436964

CCRR 0.31142468

CCRS 1.23902799

CCRT 0.95144216

CCRV 1.39622173

CCRW 0.81143447

CCRY 1.18414852

CCSA 2.76913403

CCSC 6.86751215

CCSD 1.58560053

CCSE 0.99486623

CCSF 2.07742664

CCSG 2.03029814

CCSH 1.85895955

CCSI 2.26128962

CCSK 1.01977155

CCSL 2.12062380

CCSM 2.15994880

CCSN 1.87574703

CCSP 1.17179768

CCSQ 1.18014575

CCSR 1.28895714

CCSS 2.99124539

CCST 2.56839595

CCSV 2.71477011

CCSW 1.60280700

CCSY 2.14625743

CCTA 2.53792272

CCTC 6.33537757

CCTD 1.10174411

CCTE 0.76309153

CCTF 1.91701257

CCTG 1.83403895

CCTH 1.60661662

CCTI 2.10372373

CCTK 0.86617307

CCTL 1.87251548

CCTM 1.91520769

CCTN 1.47413624

CCTP 0.88624825

CCTQ 1.07269175

CCTR 1.04538011

CCTS 2.41759343

CCTT 2.15826557

CCTV 2.37284255

CCTW 1.46103332

CCTY 1.80043046

CCVA 2.92552372

CCVC 6.32055512

CCVD 1.00361044

CCVE 0.88983620

CCVF 2.04131028

CCVG 1.82070608

CCVH 1.75552864

CCVI 2.39962211

CCVK 1.11643574

CCVL 2.16478173

CCVM 2.18175955

CCVN 1.64040608

CCVP 1.18680329

CCVQ 1.30652990

CCVR 1.23846725

CCVS 2.50173212

CCVT 2.34316642

CCVV 2.71048617

CCVW 1.82021274

CCVY 1.97768571

CCWA 1.80355110

CCWC 5.37207471

CCWD 0.53814678

CCWE 0.30085512

CCWF 1.20703595

CCWG 1.14958106

CCWH 1.14596214

CCWI 1.50347515

CCWK 0.62969775

CCWL 1.31057284

CCWM 1.38510034

CCWN 0.87373654

CCWP 0.48076736

CCWQ 0.64197493

CCWR 0.93203216

CCWS 1.80968255

CCWT 1.40252913

CCWV 1.56444726

CCWW 1.08041106

CCWY 1.33721993

CCYA 2.19746292

CCYC 6.11428529

CCYD 0.65620256

CCYE 0.48396882

CCYF 1.60453080

CCYG 1.51102982

CCYH 1.37932655

CCYI 1.85545897

CCYK 0.94740363

CCYL 1.70521700

CCYM 1.75307849

CCYN 1.18280900

CCYP 0.89249953

CCYQ 1.18079382

CCYR 1.02527878

CCYS 1.93937216

CCYT 1.78857774

CCYV 2.03584017

CCYW 1.28508427

CCYY 1.77152165

CDAA 4.30289031

CDAC 3.95505297

CDAD 7.69587462

CDAE 5.91105446

CDAF 2.98820066

CDAG 4.86598674

CDAH 4.86194385

CDAI 2.99821426

CDAK 4.83441779

CDAL 3.08506194

CDAM 3.40354904

CDAN 5.72389413

CDAP 4.76740475

CDAQ 4.91402853

CDAR 4.53620724

CDAS 5.09949505

CDAT 4.65098598

CDAV 3.24765110

CDAW 3.10556227

CDAY 3.58232327

CDCA 7.94995348

CDCC 6.58392599

CDCD 13.81060910

CDCE 9.94388191

CDCF 6.25708508

CDCG 8.73352161

CDCH 8.26650830

CDCI 6.49686286

CDCK 8.52232696

CDCL 6.53245028

CDCM 6.76227909

CDCN 10.00076283

CDCP 8.25203669

CDCQ 8.72926243

CDCR 7.96767041

CDCS 9.00716274

CDCT 8.52235196

CDCV 6.93744087

CDCW 6.10015421

CDCY 7.17231307

CDDA 3.13372601

CDDC 2.97007445

CDDD 5.50561887

CDDE 4.20204933

CDDF 1.90127815

CDDG 3.41880761

CDDH 3.34384023

CDDI 1.93715487

CDDK 3.36146437

CDDL 1.99253998

CDDM 2.34197160

CDDN 4.08144980

CDDP 3.29871010

CDDQ 3.55778354

CDDR 3.10825185

CDDS 3.72554139

CDDT 3.38172573

CDDV 2.30789675

CDDW 2.03806156

CDDY 2.35642605

CDEA 3.00236127

CDEC 2.70983183

CDED 5.41719153

CDEE 4.13203759

CDEF 1.80846842

CDEG 3.47818622

CDEH 3.32443566

CDEI 1.94606684

CDEK 3.35138094

CDEL 1.96119159

CDEM 2.22624012

CDEN 4.16636411

CDEP 3.34758167

CDEQ 3.43085524

CDER 3.06016839

CDES 3.65646720

CDET 3.25359644

CDEV 2.14483558

CDEW 2.00766391

CDEY 2.33442214

CDFA 3.77320040

CDFC 3.14177305

CDFD 6.96173337

CDFE 5.36052692

CDFF 2.36540943

CDFG 4.15384403

CDFH 4.31989743

CDFI 2.56045283

CDFK 4.48872429

CDFL 2.52134408

CDFM 2.83042970

CDFN 5.29028494

CDFP 4.10280507

CDFQ 4.47687862

CDFR 4.01434633

CDFS 4.63257250

CDFT 4.13876856

CDFV 2.71569412

CDFW 2.48296725

CDFY 3.06595802

CDGA 3.45431254

CDGC 2.93599004

CDGD 6.52664428

CDGE 4.91314305

CDGF 2.24743001

CDGG 3.61042818

CDGH 3.91208494

CDGI 2.40489380

CDGK 3.95964033

CDGL 2.33136217

CDGM 2.71391575

CDGN 4.73777508

CDGP 3.68001644

CDGQ 4.06698396

CDGR 3.69860121

CDGS 4.16916281

CDGT 3.75609802

CDGV 2.53967039

CDGW 2.39330968

CDGY 2.82248224

CDHA 3.61119692

CDHC 3.03685763

CDHD 6.34403355

CDHE 5.00547700

CDHF 2.27417461

CDHG 3.97730645

CDHH 3.76508777

CDHI 2.45649504

CDHK 4.01259603

CDHL 2.48993700

CDHM 2.74849920

CDHN 4.77114675

CDHP 3.87916682

CDHQ 4.12842103

CDHR 3.77260218

CDHS 4.25246144

CDHT 3.84800539

CDHV 2.62746238

CDHW 2.54173653

CDHY 2.74310239

CDIA 3.93147128

CDIC 3.49912189

CDID 7.15643563

CDIE 5.63112470

CDIF 2.69421277

CDIG 4.44270947

CDIH 4.54528177

CDII 2.68079281

CDIK 4.65200654

CDIL 2.78242228

CDIM 3.12108866

CDIN 5.45224962

CDIP 4.43360727

CDIQ 4.73205301

CDIR 4.25576417

CDIS 4.82687700

CDIT 4.33867498

CDIV 2.85240714

CDIW 2.89081709

CDIY 3.40614404

CDKA 3.06212228

CDKC 2.83066844

CDKD 5.62616743

CDKE 4.36021977

CDKF 1.99742008

CDKG 3.58187167

CDKH 3.47948685

CDKI 2.12515927

CDKK 3.53026256

CDKL 2.10721825

CDKM 2.76842101

CDKN 4.35243746

CDKP 3.46211880

CDKQ 3.60646475

CDKR 3.18468077

CDKS 3.83535110

CDKT 3.37392906

CDKV 2.23968820

CDKW 2.03263912

CDKY 2.48155970

CDLA 3.86681976

CDLC 3.23134106

CDLD 6.93017481

CDLE 5.43389923

CDLF 2.41629483

CDLG 4.37143426

CDLH 4.41973511

CDLI 2.52734362

CDLK 4.48338634

CDLL 2.62346370

CDLM 2.96322306

CDLN 5.22841177

CDLP 4.29708206

CDLQ 4.55216370

CDLR 4.14108135

CDLS 4.67642826

CDLT 4.24360024

CDLV 2.75835058

CDLW 2.60540898

CDLY 3.09421690

CDMA 3.77318669

CDMC 3.32871611

CDMD 6.94044889

CDME 5.37568833

CDMF 2.52074708

CDMG 4.32338510

CDMH 4.33119358

CDMI 2.55744013

CDMK 4.36528972

CDML 2.64963067

CDMM 2.87476718

CDMN 5.22682878

CDMP 4.24602580

CDMQ 4.39263055

CDMR 4.04674104

CDMS 4.54807805

CDMT 4.15079593

CDMV 2.76068393

CDMW 2.84323849

CDMY 3.16781978

CDNA 3.44162047

CDNC 3.19730741

CDND 6.36608896

CDNE 4.87004222

CDNF 2.25841427

CDNG 3.80746480

CDNH 3.87371245

CDNI 2.41808009

CDNK 3.88452598

CDNL 2.40201370

CDNM 2.71110362

CDNN 4.70305566

CDNP 3.70598308

CDNQ 4.04253131

CDNR 3.50626323

CDNS 4.09863070

CDNT 3.75411295

CDNV 2.57719776

CDNW 2.38851571

CDNY 2.81053830

CDPA 3.13501490

CDPC 3.10800472

CDPD 5.86198497

CDPE 4.43744962

CDPF 2.00623201

CDPG 3.37882098

CDPH 3.46446494

CDPI 1.99792867

CDPK 3.53909917

CDPL 2.06189870

CDPM 2.39423584

CDPN 4.21890175

CDPP 3.26931238

CDPQ 3.61088669

CDPR 3.22532035

CDPS 3.81721446

CDPT 3.45072350

CDPV 2.20735127

CDPW 2.01537416

CDPY 2.42960082

CDQA 3.17975275

CDQC 2.89573068

CDQD 5.81069248

CDQE 4.49529789

CDQF 2.02391030

CDQG 3.68872284

CDQH 3.60729127

CDQI 2.03296023

CDQK 3.55054924

CDQL 2.08977909

CDQM 2.40176689

CDQN 4.30997339

CDQP 3.60871017

CDQQ 3.65214823

CDQR 3.29437720

CDQS 3.80638378

CDQT 3.46178720

CDQV 2.27670840

CDQW 2.35795293

CDQY 2.51657038

CDRA 3.32607204

CDRC 2.87046694

CDRD 6.01670433

CDRE 4.56415029

CDRF 1.94432984

CDRG 3.73095575

CDRH 3.57923162

CDRI 2.08750277

CDRK 3.69145931

CDRL 2.07609126

CDRM 2.38704080

CDRN 4.46169046

CDRP 3.59918515

CDRQ 3.69070788

CDRR 3.29726283

CDRS 3.93373456

CDRT 3.47622373

CDRV 2.35331005

CDRW 2.16218664

CDRY 2.48514765

CDSA 4.10369417

CDSC 3.76190991

CDSD 7.33415566

CDSE 5.67481449

CDSF 2.88385830

CDSG 4.60240668

CDSH 4.57954980

CDSI 2.93688147

CDSK 4.56782516

CDSL 3.01194435

CDSM 3.32307397

CDSN 5.48702675

CDSP 4.43349866

CDSQ 4.65488664

CDSR 4.23308405

CDSS 4.80234179

CDST 4.38805249

CDSV 3.16015915

CDSW 3.07220367

CDSY 3.56627226

CDTA 3.94756858

CDTC 3.59175577

CDTD 7.12387353

CDTE 5.45837323

CDTF 2.66986318

CDTG 4.45157766

CDTH 4.48309208

CDTI 2.75630279

CDTK 4.40558680

CDTL 2.82201710

CDTM 3.09603485

CDTN 5.28432003

CDTP 4.34629906

CDTQ 4.52673173

CDTR 4.10293590

CDTS 4.68069947

CDTT 4.19530495

CDTV 2.91899041

CDTW 2.81178549

CDTY 3.21603377

CDVA 4.28561375

CDVC 3.71711098

CDVD 7.61584437

CDVE 5.92864436

CDVF 2.83775016

CDVG 4.70489045

CDVH 4.79242214

CDVI 2.86585150

CDVK 4.82548840

CDVL 2.97912822

CDVM 3.34609859

CDVN 5.65412326

CDVP 4.72475845

CDVQ 4.95553117

CDVR 4.50779182

CDVS 5.05186889

CDVT 4.56227458

CDVV 3.06123834

CDVW 3.07981702

CDVY 3.50622500

CDWA 3.41303754

CDWC 2.61012674

CDWD 6.27662910

CDWE 4.81003665

CDWF 1.94111676

CDWG 3.67541994

CDWH 3.70316518

CDWI 2.26926482

CDWK 4.03107600

CDWL 2.07636251

CDWM 2.73585251

CDWN 4.66506130

CDWP 3.62293290

CDWQ 3.93132656

CDWR 3.53071185

CDWS 4.16043472

CDWT 3.77975351

CDWV 2.40062625

CDWW 2.14165661

CDWY 2.46557968

CDYA 3.82186317

CDYC 3.19557678

CDYD 6.84649006

CDYE 5.29759991

CDYF 2.49585135

CDYG 4.25664161

CDYH 4.20924655

CDYI 2.72405945

CDYK 4.35827679

CDYL 2.65460660

CDYM 3.02136850

CDYN 5.26716775

CDYP 4.11396868

CDYQ 4.42192150

CDYR 3.96099345

CDYS 4.66334166

CDYT 4.30160134

CDYV 2.92424913

CDYW 2.48383334

CDYY 3.00679660

CEAA 4.70857561

CEAC 3.86748166

CEAD 6.07568646

CEAE 7.18457674

CEAF 3.33189674

CEAG 4.50679033

CEAH 4.94252053

CEAI 3.66855916

CEAK 5.37478148

CEAL 3.73783817

CEAM 4.09779188

CEAN 5.12046943

CEAP 4.82658696

CEAQ 5.61894976

CEAR 5.09590742

CEAS 5.03886711

CEAT 4.82423666

CEAV 3.91487611

CEAW 3.50806704

CEAY 3.90996898

CECA 8.15564820

CECC 4.96213620

CECD 9.94388191

CECE 13.00107356

CECF 6.47957604

CECG 8.12688276

CECH 7.89857953

CECI 7.10559188

CECK 8.99002905

CECL 7.20582656

CECM 7.54170790

CECN 8.73245105

CECP 8.30399285

CECQ 9.53874010

CECR 8.38928734

CECS 8.60807215

CECT 8.44208758

CECV 7.46673205

CECW 6.36427551

CECY 7.21726585

CEDA 3.37520914

CEDC 2.72284648

CEDD 4.34306424

CEDE 5.10296697

CEDF 2.03989003

CEDG 3.12672540

CEDH 3.39864079

CEDI 2.45688723

CEDK 3.78963235

CEDL 2.40963229

CEDM 2.85986026

CEDN 3.60383661

CEDP 3.48636640

CEDQ 4.03214305

CEDR 3.50514258

CEDS 3.63755533

CEDT 3.42785669

CEDV 2.66175093

CEDW 2.26233803

CEDY 2.50091450

CEEA 3.36784078

CEEC 2.66167520

CEED 4.33568030

CEEE 5.05806911

CEEF 2.14140195

CEEG 3.21743125

CEEH 3.44556011

CEEI 2.54006254

CEEK 3.88176054

CEEL 2.45275194

CEEM 2.81551341

CEEN 3.73061513

CEEP 3.45043283

CEEQ 4.00476013

CEER 3.56807413

CEES 3.66890939

CEET 3.46745930

CEEV 2.71123440

CEEW 2.33390279

CEEY 2.54036679

CEFA 4.04361190

CEFC 3.15019994

CEFD 5.42978969

CEFE 6.49874349

CEFF 2.66351687

CEFG 3.81350664

CEFH 4.24435491

CEFI 3.14145851

CEFK 4.85135568

CEFL 3.11989518

CEFM 3.36554722

CEFN 4.56193027

CEFP 4.12193186

CEFQ 5.10810722

CEFR 4.41374834

CEFS 4.43965094

CEFT 4.25528885

CEFV 3.31918127

CEFW 2.78273392

CEFY 3.32835400

CEGA 3.66291089

CEGC 2.75587625

CEGD 4.83255713

CEGE 6.04432599

CEGF 2.48800829

CEGG 3.17567824

CEGH 3.80878676

CEGI 2.78513305

CEGK 4.36910383

CEGL 2.77828253

CEGM 3.12015905

CEGN 4.00512027

CEGP 3.69695484

CEGQ 4.60760539

CEGR 4.02484010

CEGS 3.96994020

CEGT 3.75563167

CEGV 2.94837143

CEGW 2.50087443

CEGY 2.89966335

CEHA 3.92690514

CEHC 2.82776478

CEHD 5.03075518

CEHE 5.99578493

CEHF 2.60452960

CEHG 3.60901500

CEHH 3.76495265

CEHI 2.99685872

CEHK 4.49938636

CEHL 2.98114600

CEHM 3.41794283

CEHN 4.17040362

CEHP 3.96851371

CEHQ 4.71769431

CEHR 4.15740496

CEHS 4.15991068

CEHT 3.99729482

CEHV 3.22197231

CEHW 2.78106734

CEHY 2.97728072

CEIA 4.28398386

CEIC 3.74374701

CEID 5.71197807

CEIE 6.70266274

CEIF 2.98903870

CEIG 4.11226356

CEIH 4.57161887

CEII 3.32087989

CEIK 5.17784539

CEIL 3.36534007

CEIM 3.64830098

CEIN 4.92514372

CEIP 4.45150103

CEIQ 5.32132630

CEIR 4.72636254

CEIS 4.76129909

CEIT 4.50604778

CEIV 3.45390673

CEIW 3.14522323

CEIY 3.65211558

CEKA 3.41800415

CEKC 2.84647487

CEKD 4.57919124

CEKE 5.26958576

CEKF 2.22009623

CEKG 3.27929377

CEKH 3.51942155

CEKI 2.70494111

CEKK 4.03146008

CEKL 2.61160264

CEKM 3.07053162

CEKN 3.96147401

CEKP 3.50346032

CEKQ 4.22788856

CEKR 3.65511783

CEKS 3.82631233

CEKT 3.60399324

CEKV 2.82896149

CEKW 2.32551940

CEKY 2.74195213

CELA 4.26057241

CELC 3.34329257

CELD 5.60717522

CELE 6.54014818

CELF 2.74504057

CELG 4.10436240

CELH 4.46401230

CELI 3.18157235

CELK 4.98802149

CELL 3.16227193

CELM 3.50972233

CELN 4.73953681

CELP 4.30398663

CELQ 5.16798851

CELR 4.60373687

CELS 4.61899382

CELT 4.38215238

CELV 3.34641609

CELW 2.88040775

CELY 3.43481108

CEMA 4.13167977

CEMC 3.39252791

CEMD 5.51264866

CEME 6.54698684

CEMF 2.77641047

CEMG 3.99310138

CEMH 4.35596847

CEMI 3.16873972

CEMK 4.83572678

CEML 3.20693157

CEMM 3.38418716

CEMN 4.61347290

CEMP 4.25879816

CEMQ 5.03848737

CEMR 4.52181327

CEMS 4.45101182

CEMT 4.25564566

CEMV 3.30959948

CEMW 3.16503147

CEMY 3.47263968

CENA 3.70675124

CENC 3.10208252

CEND 4.93780936

CENE 5.94077343

CENF 2.48109041

CENG 3.48327484

CENH 3.85475346

CENI 3.01231963

CENK 4.32454155

CENL 2.89507167

CENM 3.35696044

CENN 4.11175670

CENP 3.77241558

CENQ 4.57291091

CENR 3.95502906

CENS 3.99010899

CENT 3.83148720

CENV 3.12850320

CENW 2.58186032

CENY 2.95729335

CEPA 3.36725629

CEPC 2.44573925

CEPD 4.37868963

CEPE 5.29499396

CEPF 2.01054280

CEPG 2.97188585

CEPH 3.40840311

CEPI 2.43519886

CEPK 3.81950052

CEPL 2.39453535

CEPM 2.70597515

CEPN 3.56259783

CEPP 3.32597857

CEPQ 4.06765306

CEPR 3.49266554

CEPS 3.62696169

CEPT 3.42565048

CEPV 2.67347633

CEPW 2.13071626

CEPY 2.48354237

CEQA 3.51748727

CEQC 2.87356926

CEQD 4.66540667

CEQE 5.41376446

CEQF 2.29125549

CEQG 3.39560010

CEQH 3.86924321

CEQI 2.70240387

CEQK 4.07028159

CEQL 2.62232979

CEQM 2.87556296

CEQN 3.89751787

CEQP 3.64977950

CEQQ 4.20825466

CEQR 3.79981890

CEQS 3.76733274

CEQT 3.66119357

CEQV 2.91400321

CEQW 2.47329503

CEQY 2.77487043

CERA 3.65826838

CERC 2.76857833

CERD 4.76582396

CERE 5.62215283

CERF 2.22727189

CERG 3.41696969

CERH 3.66231952

CERI 2.66227649

CERK 4.08433539

CERL 2.63889234

CERM 3.06426179

CERN 3.92129698

CERP 3.61814271

CERQ 4.31772326

CERR 3.77387117

CERS 3.87607446

CERT 3.66999970

CERV 2.92550983

CERW 2.46195331

CERY 2.63384835

CESA 4.43630200

CESC 3.70168044

CESD 5.80586969

CESE 6.92941656

CESF 3.19559761

CESG 4.24778507

CESH 4.69005064

CESI 3.60977992

CESK 5.05368352

CESL 3.57991824

CESM 3.87582138

CESN 4.85841683

CESP 4.51512927

CESQ 5.32180507

CESR 4.71169504

CESS 4.70014903

CEST 4.47671254

CESV 3.78561478

CESW 3.26154635

CESY 3.69470436

CETA 4.28255249

CETC 3.50177897

CETD 5.58150282

CETE 6.61228373

CETF 2.99921804

CETG 4.06389888

CETH 4.49085430

CETI 3.35145264

CETK 4.89465943

CETL 3.36993136

CETM 3.58393112

CETN 4.64988876

CETP 4.37333960

CETQ 5.14919573

CETR 4.60315800

CETS 4.54541753

CETT 4.32423612

CETV 3.51402297

CETW 3.19842962

CETY 3.46743742

CEVA 4.63493625

CEVC 3.89912412

CEVD 6.01514490

CEVE 7.08787799

CEVF 3.16242853

CEVG 4.39322241

CEVH 4.81273763

CEVI 3.55828786

CEVK 5.34610734

CEVL 3.56840150

CEVM 3.87440386

CEVN 5.10363501

CEVP 4.71179125

CEVQ 5.61665474

CEVR 5.01274021

CEVS 4.99344392

CEVT 4.72482359

CEVV 3.72002367

CEVW 3.38584268

CEVY 3.80459709

CEWA 3.62552080

CEWC 2.46243444

CEWD 4.83431605

CEWE 5.96334660

CEWF 2.13446137

CEWG 3.32918195

CEWH 3.73604512

CEWI 2.65208723

CEWK 4.36525270

CEWL 2.57252639

CEWM 3.09066054

CEWN 3.99736194

CEWP 3.53797635

CEWQ 4.61762430

CEWR 3.94244695

CEWS 3.91716475

CEWT 3.70868248

CEWV 2.80313103

CEWW 2.40900181

CEWY 2.67152433

CEYA 4.03337997

CEYC 3.14742015

CEYD 5.42511610

CEYE 6.50922212

CEYF 2.74047647

CEYG 3.88203432

CEYH 4.25223781

CEYI 3.22844384

CEYK 4.88589349

CEYL 3.21362207

CEYM 3.48665307

CEYN 4.58896073

CEYP 4.24240078

CEYQ 5.10845072

CEYR 4.39231438

CEYS 4.44499480

CEYT 4.26465610

CEYV 3.33998616

CEYW 2.81007223

CEYY 3.25599445

CFAA 4.02991366

CFAC 5.05079788

CFAD 2.83587276

CFAE 3.01243282

CFAF 7.90753037

CFAG 3.35166674

CFAH 4.77826883

CFAI 5.43998787

CFAK 3.12962468

CFAL 5.69424889

CFAM 5.40125046

CFAN 3.44865927

CFAP 3.40274493

CFAQ 3.36191286

CFAR 3.38084454

CFAS 3.70874837

CFAT 3.98770806

CFAV 5.05504049

CFAW 6.04807677

CFAY 6.51542375

CFCA 7.50988886

CFCC 5.94506531

CFCD 6.25708508

CFCE 6.47957604

CFCF 14.05990254

CFCG 6.68583512

CFCH 7.93193916

CFCI 8.87014943

CFCK 6.88990933

CFCL 9.36778646

CFCM 8.90405655

CFCN 7.04034114

CFCP 6.77124757

CFCQ 7.05173059

CFCR 6.90116153

CFCS 7.46840631

CFCT 7.49447292

CFCV 8.52352721

CFCW 9.92833604

CFCY 11.16277654

CFDA 2.23379862

CFDC 3.22809079

CFDD 1.33495438

CFDE 1.49620626

CFDF 5.69829112

CFDG 1.53604529

CFDH 2.94452709

CFDI 3.47027844

CFDK 1.60776728

CFDL 3.68160613

CFDM 3.45035186

CFDN 1.90263004

CFDP 1.87826924

CFDQ 1.76333245

CFDR 1.73189806

CFDS 2.04882349

CFDT 2.32988680

CFDV 3.14391163

CFDW 4.15614763

CFDY 4.61023013

CFEA 2.14590052

CFEC 3.18602833

CFED 1.45486618

CFEE 1.43927976

CFEF 5.62239872

CFEG 1.63207301

CFEH 2.89191073

CFEI 3.48558164

CFEK 1.50564935

CFEL 3.64424209

CFEM 3.30493275

CFEN 1.90593855

CFEP 1.84715268

CFEQ 1.71992751

CFER 1.74645805

CFES 2.01652606

CFET 2.36047903

CFEV 3.11411699

CFEW 4.12129033

CFEY 4.41605845

CFFA 3.82105061

CFFC 4.44043136

CFFD 2.63236047

CFFE 2.77401078

CFFF 7.05282734

CFFG 2.94951714

CFFH 4.22662361

CFFI 5.00595254

CFFK 2.93331118

CFFL 5.12829249

CFFM 4.86605361

CFFN 3.18581908

CFFP 3.21985658

CFFQ 3.10532715

CFFR 3.10136939

CFFS 3.42637849

CFFT 3.62090820

CFFV 4.64385333

CFFW 5.19871089

CFFY 5.78212209

CFGA 3.32059874

CFGC 3.74654739

CFGD 2.04667939

CFGE 2.28580195

CFGF 6.70338571

CFGG 2.48272982

CFGH 3.75506378

CFGI 4.56114742

CFGK 2.35854031

CFGL 4.72713350

CFGM 4.50783377

CFGN 2.60347398

CFGP 2.49324793

CFGQ 2.71960420

CFGR 2.53830491

CFGS 2.89545866

CFGT 3.14898310

CFGV 4.14246749

CFGW 4.90936550

CFGY 5.35343144

CFHA 3.02869147

CFHC 3.39440963

CFHD 2.05524410

CFHE 2.20616016

CFHF 6.50244360

CFHG 2.26876926

CFHH 3.36330637

CFHI 4.33342120

CFHK 2.33397442

CFHL 4.53070422

CFHM 4.09224540

CFHN 2.52520030

CFHP 2.56686742

CFHQ 2.53327875

CFHR 2.49272427

CFHS 2.77618631

CFHT 3.00227380

CFHV 4.02127792

CFHW 4.83111703

CFHY 5.29657461

CFIA 3.89149757

CFIC 4.78267332

CFID 2.74018528

CFIE 2.93652316

CFIF 7.25420259

CFIG 3.08089820

CFIH 4.35078590

CFII 5.12804762

CFIK 3.08645772

CFIL 5.35054127

CFIM 5.01730951

CFIN 3.32244992

CFIP 3.28952516

CFIQ 3.27386231

CFIR 3.24266204

CFIS 3.59884047

CFIT 3.82077943

CFIV 4.74000154

CFIW 5.66598054

CFIY 6.05243096

CFKA 2.31752740

CFKC 3.35171918

CFKD 1.50167396

CFKE 1.55277666

CFKF 5.88981111

CFKG 1.75735325

CFKH 3.01761110

CFKI 3.69765139

CFKK 1.76202899

CFKL 3.89881972

CFKM 3.49088703

CFKN 1.98951200

CFKP 1.94134936

CFKQ 1.81426964

CFKR 1.88150277

CFKS 2.16736801

CFKT 2.29734347

CFKV 3.31183799

CFKW 4.28327592

CFKY 4.66849293

CFLA 3.76002146

CFLC 4.59295128

CFLD 2.55628568

CFLE 2.71720958

CFLF 7.11273627

CFLG 2.97713528

CFLH 4.20201801

CFLI 4.95709171

CFLK 2.85466533

CFLL 5.12587785

CFLM 4.85403042

CFLN 3.10716683

CFLP 3.04196258

CFLQ 3.02753997

CFLR 3.04261237

CFLS 3.38913288

CFLT 3.64411554

CFLV 4.61762153

CFLW 5.38565206

CFLY 5.89802514

CFMA 3.70028614

CFMC 4.57584412

CFMD 2.54068665

CFME 2.66617271

CFMF 7.12769549

CFMG 2.95402518

CFMH 4.11745802

CFMI 4.88509065

CFMK 2.81595113

CFML 5.11013987

CFMM 4.74911018

CFMN 3.06743670

CFMP 3.12018629

CFMQ 2.99941116

CFMR 2.99010644

CFMS 3.40083791

CFMT 3.55038316

CFMV 4.53823969

CFMW 5.48220343

CFMY 5.86240677

CFNA 2.82895594

CFNC 3.76344603

CFND 1.90144754

CFNE 2.07029683

CFNF 6.55119601

CFNG 2.23165400

CFNH 3.53849867

CFNI 4.17315183

CFNK 2.22382628

CFNL 4.38631883

CFNM 4.16176503

CFNN 2.48771800

CFNP 2.34567832

CFNQ 2.37513696

CFNR 2.33099035

CFNS 2.63285682

CFNT 2.77087783

CFNV 3.78066336

CFNW 4.99298245

CFNY 5.26798888

CFPA 2.54344807

CFPC 3.09471903

CFPD 1.58472593

CFPE 1.65518794

CFPF 5.82029815

CFPG 1.72110623

CFPH 2.96166893

CFPI 3.74504748

CFPK 1.88082621

CFPL 3.95734495

CFPM 3.67778726

CFPN 2.05289180

CFPP 1.95129933

CFPQ 2.07619488

CFPR 1.97554204

CFPS 2.33346421

CFPT 2.41479415

CFPV 3.34832818

CFPW 4.29385459

CFPY 4.65899647

CFQA 2.47841106

CFQC 3.51631709

CFQD 1.52679479

CFQE 1.58138686

CFQF 6.05131370

CFQG 1.92683962

CFQH 3.10038795

CFQI 3.80554973

CFQK 1.74663782

CFQL 3.97436524

CFQM 3.64384168

CFQN 2.13181801

CFQP 2.10517526

CFQQ 1.92878024

CFQR 2.04823015

CFQS 2.26508251

CFQT 2.57602095

CFQV 3.42951585

CFQW 4.39357684

CFQY 4.78485505

CFRA 2.60057650

CFRC 3.50648340

CFRD 1.66510869

CFRE 1.71677681

CFRF 6.25729169

CFRG 1.85677747

CFRH 3.12942870

CFRI 3.87444757

CFRK 1.85261411

CFRL 4.13536644

CFRM 3.71964645

CFRN 2.11265052

CFRP 2.26802673

CFRQ 1.99514855

CFRR 1.97533473

CFRS 2.33435529

CFRT 2.56021066

CFRV 3.57448962

CFRW 4.36773555

CFRY 4.92628628

CFSA 3.66556780

CFSC 4.75173013

CFSD 2.52975428

CFSE 2.65877628

CFSF 7.75500632

CFSG 3.03743744

CFSH 4.37942853

CFSI 5.10045812

CFSK 2.82118513

CFSL 5.36451295

CFSM 5.00895184

CFSN 3.13659223

CFSP 3.05228283

CFSQ 3.07553548

CFSR 3.05189657

CFSS 3.35399786

CFST 3.58095719

CFSV 4.66144663

CFSW 5.86552461

CFSY 6.26010765

CFTA 3.60237070

CFTC 4.61258268

CFTD 2.45205094

CFTE 2.55975158

CFTF 7.33916964

CFTG 2.87576686

CFTH 4.23304378

CFTI 4.96185170

CFTK 2.71967516

CFTL 5.20752317

CFTM 4.90173267

CFTN 2.98152772

CFTP 2.99347025

CFTQ 2.96686247

CFTR 2.95459698

CFTS 3.26233444

CFTT 3.41444293

CFTV 4.51695852

CFTW 5.56685724

CFTY 6.03618467

CFVA 4.09768130

CFVC 4.90960374

CFVD 2.91081869

CFVE 3.13067144

CFVF 7.72911242

CFVG 3.24119001

CFVH 4.68305098

CFVI 5.32468684

CFVK 3.21500951

CFVL 5.57129894

CFVM 5.26969588

CFVN 3.38935223

CFVP 3.37804903

CFVQ 3.49545991

CFVR 3.44388006

CFVS 3.73975552

CFVT 3.94231692

CFVV 4.95166433

CFVW 6.03067082

CFVY 6.36258286

CFWA 3.44866145

CFWC 3.64575065

CFWD 2.26555978

CFWE 2.40343823

CFWF 6.36447082

CFWG 2.75168583

CFWH 3.83189850

CFWI 4.38289124

CFWK 2.66815053

CFWL 4.52137736

CFWM 4.43444143

CFWN 2.70068521

CFWP 2.81055991

CFWQ 2.76531661

CFWR 2.73344574

CFWS 3.08058141

CFWT 3.28194522

CFWV 4.10462867

CFWW 4.62206536

CFWY 5.12569446

CFYA 3.43992959

CFYC 4.20520548

CFYD 2.43850732

CFYE 2.62241739

CFYF 6.99227242

CFYG 2.72748090

CFYH 3.98809505

CFYI 4.78240343

CFYK 2.82358497

CFYL 4.95666349

CFYM 4.65603009

CFYN 2.95027525

CFYP 2.90448009

CFYQ 2.91005761

CFYR 2.86622072

CFYS 3.26971421

CFYT 3.36759890

CFYV 4.38688318

CFYW 5.04997197

CFYY 5.62738301

CGAA 4.80184431

CGAC 4.38884348

CGAD 4.44555234

CGAE 3.93285248

CGAF 3.06618270

CGAG 7.63355025

CGAH 3.90634330

CGAI 2.96883256

CGAK 3.96232040

CGAL 3.00393050

CGAM 3.35483908

CGAN 4.71115435

CGAP 4.14638760

CGAQ 3.82404230

CGAR 3.75078980

CGAS 4.77414026

CGAT 3.97230379

CGAV 3.28176067

CGAW 3.03957432

CGAY 3.12248027

CGCA 9.12089630

CGCC 5.86867144

CGCD 8.73352161

CGCE 8.12688276

CGCF 6.68583512

CGCG 14.25064233

CGCH 7.57262303

CGCI 6.46189891

CGCK 8.37826913

CGCL 6.74631862

CGCM 7.11072914

CGCN 9.18490396

CGCP 8.01307552

CGCQ 8.15295238

CGCR 8.02803635

CGCS 9.13293807

CGCT 8.16186800

CGCV 6.96570231

CGCW 6.56601743

CGCY 6.94701439

CGDA 2.92785028

CGDC 2.75143950

CGDD 2.79605465

CGDE 2.44161973

CGDF 1.58247903

CGDG 5.40155758

CGDH 2.42723381

CGDI 1.43841200

CGDK 2.45930307

CGDL 1.51537201

CGDM 1.97960117

CGDN 3.01402064

CGDP 2.70493037

CGDQ 2.35037583

CGDR 2.21338164

CGDS 3.05064627

CGDT 2.45644971

CGDV 1.82784748

CGDW 1.56485378

CGDY 1.66263260

CGEA 2.73203683

CGEC 2.55420667

CGED 2.79374374

CGEE 2.28821977

CGEF 1.44897092

CGEG 5.21911640

CGEH 2.24646110

CGEI 1.35893697

CGEK 2.22263148

CGEL 1.56262260

CGEM 1.67745656

CGEN 2.98319383

CGEP 2.52835356

CGEQ 2.17348789

CGER 2.03708425

CGES 2.90522515

CGET 2.28192751

CGEV 1.60528529

CGEW 1.66349807

CGEY 1.58971837

CGFA 4.22879528

CGFC 3.52856469

CGFD 3.99013234

CGFE 3.53702990

CGFF 2.50311259

CGFG 6.73902772

CGFH 3.27529187

CGFI 2.57885707

CGFK 3.56014691

CGFL 2.49571773

CGFM 2.87956707

CGFN 4.16341296

CGFP 3.54173636

CGFQ 3.42553841

CGFR 3.32737565

CGFS 4.28475888

CGFT 3.52615772

CGFV 2.76366018

CGFW 2.38986317

CGFY 2.60001301

CGGA 3.98480999

CGGC 3.55193147

CGGD 3.43220705

CGGE 3.16791552

CGGF 2.52629471

CGGG 6.29559217

CGGH 3.03329999

CGGI 2.43241943

CGGK 3.17889949

CGGL 2.43271750

CGGM 2.79244098

CGGN 3.72375784

CGGP 3.09313820

CGGQ 3.11697887

CGGR 2.93939787

CGGS 3.92820947

CGGT 3.22037061

CGGV 2.63231628

CGGW 2.29729400

CGGY 2.55523734

CGHA 3.74691154

CGHC 2.90568551

CGHD 3.60478233

CGHE 3.12182629

CGHF 2.32500033

CGHG 6.16628805

CGHH 2.86393500

CGHI 2.11232919

CGHK 3.18824001

CGHL 2.10202096

CGHM 2.42294903

CGHN 3.77928265

CGHP 3.21072894

CGHQ 3.07971107

CGHR 3.00400015

CGHS 3.76139337

CGHT 2.97582085

CGHV 2.29989305

CGHW 2.11451495

CGHY 2.23496081

CGIA 4.45175019

CGIC 3.99473245

CGID 4.17097723

CGIE 3.74652752

CGIF 2.78636535

CGIG 7.01395447

CGIH 3.63743435

CGII 2.65011678

CGIK 3.73419065

CGIL 2.69080373

CGIM 3.06391713

CGIN 4.37929211

CGIP 3.81930013

CGIQ 3.61190792

CGIR 3.51148623

CGIS 4.50948853

CGIT 3.70988474

CGIV 2.90573181

CGIW 2.94164281

CGIY 2.95802724

CGKA 2.94484804

CGKC 2.83854201

CGKD 2.96667868

CGKE 2.45146972

CGKF 1.67862096

CGKG 5.53890951

CGKH 2.45724911

CGKI 1.53802940

CGKK 2.60240699

CGKL 1.57308147

CGKM 1.82159066

CGKN 3.27863652

CGKP 2.55962958

CGKQ 2.36220398

CGKR 2.35996002

CGKS 3.08988781

CGKT 2.41094898

CGKV 1.74687828

CGKW 1.67907458

CGKY 1.87536798

CGLA 4.25749227

CGLC 3.84023890

CGLD 4.05576062

CGLE 3.58050353

CGLF 2.60955767

CGLG 6.82838469

CGLH 3.46683370

CGLI 2.50372465

CGLK 3.57059081

CGLL 2.52895006

CGLM 2.80801349

CGLN 4.23123211

CGLP 3.67449310

CGLQ 3.48988761

CGLR 3.34811545

CGLS 4.28650522

CGLT 3.58279165

CGLV 2.77455810

CGLW 2.62660104

CGLY 2.65694166

CGMA 4.20091477

CGMC 3.80466814

CGMD 3.95627918

CGME 3.52612941

CGMF 2.68776581

CGMG 6.84045409

CGMH 3.36409739

CGMI 2.52100850

CGMK 3.42082972

CGML 2.54037917

CGMM 2.82605722

CGMN 4.12749275

CGMP 3.62922716

CGMQ 3.31149912

CGMR 3.30713514

CGMS 4.22252789

CGMT 3.47898835

CGMV 2.74476391

CGMW 2.90144374

CGMY 2.71248456

CGNA 3.67353898

CGNC 3.36333630

CGND 3.37654108

CGNE 3.00469953

CGNF 2.22138611

CGNG 6.21367513

CGNH 2.96541761

CGNI 2.09635164

CGNK 3.00909577

CGNL 2.08716342

CGNM 2.49246867

CGNN 3.69812740

CGNP 3.11495496

CGNQ 2.89428568

CGNR 2.83754382

CGNS 3.66831747

CGNT 2.94111679

CGNV 2.34916411

CGNW 2.09588017

CGNY 2.28183540

CGPA 3.21029366

CGPC 2.73736667

CGPD 2.98151875

CGPE 2.58313240

CGPF 1.81734440

CGPG 5.56196377

CGPH 2.51476499

CGPI 1.71475346

CGPK 2.61592009

CGPL 1.70308608

CGPM 2.08249147

CGPN 3.23159234

CGPP 2.66666681

CGPQ 2.55232895

CGPR 2.40778370

CGPS 3.31186610

CGPT 2.65727060

CGPV 2.09914031

CGPW 1.72273862

CGPY 1.93343188

CGQA 3.15869913

CGQC 2.84702072

CGQD 3.04863991

CGQE 2.59016240

CGQF 1.80527534

CGQG 5.67579746

CGQH 2.55138581

CGQI 1.62168019

CGQK 2.58430144

CGQL 1.65092281

CGQM 1.92563571

CGQN 3.25838093

CGQP 2.84526983

CGQQ 2.43351327

CGQR 2.44797542

CGQS 3.18874144

CGQT 2.53475580

CGQV 1.88745467

CGQW 2.06531739

CGQY 2.05282292

CGRA 3.20752541

CGRC 2.86756068

CGRD 3.11744176

CGRE 2.63244295

CGRF 1.69398602

CGRG 5.80753427

CGRH 2.61625154

CGRI 1.61302891

CGRK 2.71056474

CGRL 1.68342691

CGRM 2.01640275

CGRN 3.32654048

CGRP 2.85330319

CGRQ 2.56463904

CGRR 2.49981011

CGRS 3.32713035

CGRT 2.62152439

CGRV 1.89270230

CGRW 1.82397007

CGRY 1.90122823

CGSA 4.52277987

CGSC 4.15060526

CGSD 4.09511917

CGSE 3.66001815

CGSF 3.01395149

CGSG 7.32355356

CGSH 3.54765746

CGSI 2.89590717

CGSK 3.69904923

CGSL 2.81491165

CGSM 3.19190185

CGSN 4.40018167

CGSP 3.78352439

CGSQ 3.50056649

CGSR 3.46316569

CGSS 4.45463288

CGST 3.70442821

CGSV 3.06753208

CGSW 2.91852415

CGSY 3.02006264

CGTA 4.39250595

CGTC 3.97273009

CGTD 3.99072647

CGTE 3.52226258

CGTF 2.84910517

CGTG 7.06760665

CGTH 3.48808315

CGTI 2.62686156

CGTK 3.55648587

CGTL 2.66478269

CGTM 3.01255948

CGTN 4.23997526

CGTP 3.64285632

CGTQ 3.43362765

CGTR 3.35843531

CGTS 4.31886311

CGTT 3.50595890

CGTV 2.90824830

CGTW 2.77956366

CGTY 2.85592639

CGVA 4.74650429

CGVC 4.22271331

CGVD 4.41871532

CGVE 3.99436758

CGVF 2.88539527

CGVG 7.41538916

CGVH 3.88764585

CGVI 2.77966684

CGVK 3.95135195

CGVL 2.90634412

CGVM 3.23526692

CGVN 4.58281948

CGVP 4.08343242

CGVQ 3.81651338

CGVR 3.74774208

CGVS 4.71160449

CGVT 3.89450893

CGVV 3.08632431

CGVW 3.05186562

CGVY 3.05864883

CGWA 3.69055581

CGWC 2.97645428

CGWD 3.43579874

CGWE 3.04346992

CGWF 2.10099342

CGWG 6.21624521

CGWH 2.86304185

CGWI 2.10952355

CGWK 3.15413239

CGWL 2.11511539

CGWM 2.60468039

CGWN 3.61635135

CGWP 3.05199620

CGWQ 2.98354551

CGWR 2.89703939

CGWS 3.81121565

CGWT 3.06516105

CGWV 2.35191630

CGWW 2.14541407

CGWY 2.18554418

CGYA 4.06608910

CGYC 3.48246985

CGYD 3.90671447

CGYE 3.51536344

CGYF 2.40649941

CGYG 6.62212225

CGYH 3.24183670

CGYI 2.43503516

CGYK 3.56932610

CGYL 2.34829613

CGYM 2.75966481

CGYN 4.13183544

CGYP 3.61984757

CGYQ 3.32168124

CGYR 3.17729554

CGYS 4.13696321

CGYT 3.40796078

CGYV 2.66908829

CGYW 2.26519839

CGYY 2.48860136

CHAA 3.65568257

CHAC 4.01492771

CHAD 4.23836116

CHAE 4.10100565

CHAF 4.17109807

CHAG 3.64664102

CHAH 7.67969976

CHAI 3.29763123

CHAK 4.37292735

CHAL 3.49353127

CHAM 3.67816262

CHAN 4.77673249

CHAP 3.67490147

CHAQ 4.57141576

CHAR 4.44526070

CHAS 4.17467439

CHAT 3.93051505

CHAV 3.35895894

CHAW 4.21667711

CHAY 5.12874147

CHCA 7.36154796

CHCC 6.92721752

CHCD 8.26650830

CHCE 7.89857953

CHCF 7.93193916

CHCG 7.57262303

CHCH 15.30318341

CHCI 6.69807050

CHCK 8.21616584

CHCL 7.10289973

CHCM 7.28444609

CHCN 8.93841089

CHCP 7.43860227

CHCQ 8.64403633

CHCR 8.37482533

CHCS 8.04017804

CHCT 7.71687971

CHCV 6.91065175

CHCW 7.43777759

CHCY 9.21967831

CHDA 2.17860519

CHDC 2.58572495

CHDD 2.49377370

CHDE 2.37905212

CHDF 2.81833972

CHDG 2.23970323

CHDH 6.02108629

CHDI 1.96560787

CHDK 2.78262661

CHDL 2.15792449

CHDM 2.21803581

CHDN 3.19606749

CHDP 2.25812145

CHDQ 2.94092157

CHDR 2.80249020

CHDS 2.67483629

CHDT 2.42131928

CHDV 1.98941995

CHDW 2.81017480

CHDY 3.58618540

CHEA 2.09826896

CHEC 2.56940791

CHED 2.53820498

CHEE 2.33478519

CHEF 2.53946029

CHEG 2.11617518

CHEH 5.22963436

CHEI 1.90465293

CHEK 2.76973000

CHEL 1.92342085

CHEM 2.21134708

CHEN 2.95685588

CHEP 2.25606735

CHEQ 2.82461609

CHER 2.62851318

CHES 2.56132416

CHET 2.39435427

CHEV 1.92099565

CHEW 2.57178411

CHEY 3.28041297

CHFA 3.13708132

CHFC 3.37456645

CHFD 3.76947052

CHFE 3.62664571

CHFF 3.42589820

CHFG 2.96611439

CHFH 6.67543101

CHFI 2.81430785

CHFK 3.91291423

CHFL 2.90785054

CHFM 3.10504328

CHFN 4.16556592

CHFP 3.12366206

CHFQ 4.16652970

CHFR 3.97262728

CHFS 3.69661909

CHFT 3.44139652

CHFV 2.75836097

CHFW 3.46807421

CHFY 4.47267513

CHGA 2.69564996

CHGC 3.04067862

CHGD 3.16934494

CHGE 3.15866372

CHGF 3.24193567

CHGG 2.33924986

CHGH 6.47210074

CHGI 2.55702338

CHGK 3.38498535

CHGL 2.62521197

CHGM 2.86020524

CHGN 3.73554845

CHGP 2.63665994

CHGQ 3.77150832

CHGR 3.48778797

CHGS 3.17567099

CHGT 2.94473838

CHGV 2.49500545

CHGW 3.07127895

CHGY 4.02583714

CHHA 2.88581051

CHHC 3.37036490

CHHD 3.43625277

CHHE 3.21297639

CHHF 3.29602212

CHHG 2.82968703

CHHH 6.06718209

CHHI 2.60557773

CHHK 3.38031902

CHHL 2.70135676

CHHM 2.95272345

CHHN 3.78885203

CHHP 2.80585709

CHHQ 3.65096212

CHHR 3.59741285

CHHS 3.36881293

CHHT 3.10881317

CHHV 2.61256934

CHHW 3.20337323

CHHY 4.06078469

CHIA 3.31052227

CHIC 3.58109040

CHID 3.98744256

CHIE 3.84745863

CHIF 3.67210882

CHIG 3.20799499

CHIH 6.86541001

CHII 2.94173145

CHIK 4.02776684

CHIL 3.08062268

CHIM 3.29073268

CHIN 4.37456145

CHIP 3.52851483

CHIQ 4.31599465

CHIR 4.07758533

CHIS 3.86757748

CHIT 3.62788593

CHIV 2.91820670

CHIW 3.60600326

CHIY 4.58576111

CHKA 2.28088263

CHKC 2.78335393

CHKD 2.74066363

CHKE 2.65931604

CHKF 2.69584137

CHKG 2.47278096

CHKH 5.26365553

CHKI 2.08374536

CHKK 2.88655993

CHKL 2.12467399

CHKM 2.44037008

CHKN 3.22850167

CHKP 2.34080370

CHKQ 2.90125621

CHKR 2.92380053

CHKS 2.78486930

CHKT 2.59861742

CHKV 2.06178958

CHKW 2.66982278

CHKY 3.46512450

CHLA 3.23425044

CHLC 3.48549054

CHLD 3.81588564

CHLE 3.67139963

CHLF 3.53890532

CHLG 3.18427609

CHLH 6.63927731

CHLI 2.82081623

CHLK 3.85661581

CHLL 2.92487734

CHLM 3.34706572

CHLN 4.21885218

CHLP 3.18723782

CHLQ 4.05937173

CHLR 3.92570802

CHLS 3.69117472

CHLT 3.44782449

CHLV 2.80452269

CHLW 3.45471673

CHLY 4.50724451

CHMA 3.16809450

CHMC 3.94036468

CHMD 3.90520720

CHME 3.65518413

CHMF 3.56908481

CHMG 3.13856707

CHMH 6.63975449

CHMI 2.79457900

CHMK 3.79860104

CHML 2.96323838

CHMM 3.49333516

CHMN 4.26795223

CHMP 3.32070181

CHMQ 3.99739273

CHMR 3.91056659

CHMS 3.63982758

CHMT 3.41280948

CHMV 2.84974186

CHMW 3.73934045

CHMY 4.56954528

CHNA 2.96741874

CHNC 3.24059544

CHND 3.26754912

CHNE 3.01190059

CHNF 3.32522296

CHNG 2.86550328

CHNH 6.22784637

CHNI 2.64206760

CHNK 3.36613993

CHNL 2.62932156

CHNM 2.93680761

CHNN 3.69756132

CHNP 2.76300523

CHNQ 3.52771188

CHNR 3.33865448

CHNS 3.24891614

CHNT 3.04734221

CHNV 2.51576214

CHNW 3.14855612

CHNY 4.04373195

CHPA 2.27397338

CHPC 2.79765581

CHPD 2.68169659

CHPE 2.53951273

CHPF 2.69588309

CHPG 2.04936324

CHPH 5.63488967

CHPI 2.12053916

CHPK 2.76027349

CHPL 2.12594529

CHPM 2.33181443

CHPN 3.19234580

CHPP 2.19028900

CHPQ 3.07814535

CHPR 2.85838790

CHPS 2.70995741

CHPT 2.52602828

CHPV 2.08142693

CHPW 2.63102466

CHPY 3.44837549

CHQA 2.44502846

CHQC 2.95884015

CHQD 2.94635670

CHQE 2.74637595

CHQF 2.86883960

CHQG 2.44593721

CHQH 5.56400851

CHQI 2.16318390

CHQK 3.02310219

CHQL 2.21817193

CHQM 2.36825818

CHQN 3.26697187

CHQP 2.62956715

CHQQ 3.07219225

CHQR 3.07422609

CHQS 2.86396813

CHQT 2.72481281

CHQV 2.17281036

CHQW 2.89560091

CHQY 3.65908042

CHRA 2.70206264

CHRC 2.94577172

CHRD 3.00719185

CHRE 2.91333122

CHRF 2.90483483

CHRG 2.54322612

CHRH 5.76269732

CHRI 2.31320676

CHRK 3.03388828

CHRL 2.28982260

CHRM 2.48656768

CHRN 3.48914371

CHRP 2.62832270

CHRQ 3.23413822

CHRR 3.17779853

CHRS 2.98413299

CHRT 2.76713912

CHRV 2.31305030

CHRW 2.73225611

CHRY 3.64111673

CHSA 3.43263491

CHSC 4.01380341

CHSD 4.00673323

CHSE 3.83636541

CHSF 4.16596641

CHSG 3.35888927

CHSH 7.37993009

CHSI 3.15211651

CHSK 3.98355352

CHSL 3.40012049

CHSM 3.49385174

CHSN 4.59422219

CHSP 3.41560063

CHSQ 4.38429543

CHSR 4.17946496

CHSS 3.88743489

CHST 3.61661870

CHSV 3.14865471

CHSW 3.98153279

CHSY 5.14466659

CHTA 3.27876569

CHTC 3.83662201

CHTD 3.82188809

CHTE 3.62434555

CHTF 3.81183899

CHTG 3.22493945

CHTH 6.94954062

CHTI 2.98606213

CHTK 3.79639340

CHTL 3.16552577

CHTM 3.37064844

CHTN 4.30113585

CHTP 3.31131535

CHTQ 4.15748912

CHTR 4.00258942

CHTS 3.75865616

CHTT 3.46536025

CHTV 3.02006773

CHTW 3.84312792

CHTY 4.67633967

CHVA 3.57872367

CHVC 3.84836038

CHVD 4.27032116

CHVE 4.09041647

CHVF 4.02530701

CHVG 3.48690555

CHVH 7.31102517

CHVI 3.16050283

CHVK 4.25330183

CHVL 3.33335897

CHVM 3.55280811

CHVN 4.60680874

CHVP 3.63680196

CHVQ 4.60891870

CHVR 4.41717353

CHVS 4.11766846

CHVT 3.83994796

CHVV 3.13881507

CHVW 4.00079364

CHVY 4.93440897

CHWA 2.86948944

CHWC 2.88474032

CHWD 3.30101605

CHWE 3.16326006

CHWF 2.95269594

CHWG 2.67873150

CHWH 6.17941263

CHWI 2.54387840

CHWK 3.53517142

CHWL 2.43843893

CHWM 2.88493520

CHWN 3.90120074

CHWP 2.72762148

CHWQ 3.81166120

CHWR 3.67287513

CHWS 3.35909944

CHWT 3.08036230

CHWV 2.39144687

CHWW 3.41627019

CHWY 3.74019326

CHYA 3.04260988

CHYC 3.56972603

CHYD 3.66299742

CHYE 3.51961854

CHYF 3.54305444

CHYG 3.00599893

CHYH 6.58235000

CHYI 2.82874803

CHYK 3.79954024

CHYL 2.96948405

CHYM 3.25291335

CHYN 4.10983045

CHYP 3.11650271

CHYQ 4.00319642

CHYR 3.83827153

CHYS 3.59195938

CHYT 3.37917807

CHYV 2.77309880

CHYW 3.28896164

CHYY 4.37451959

CIAA 4.74785863

CIAC 5.42362059

CIAD 3.01989247

CIAE 3.55232089

CIAF 5.60643967

CIAG 3.30529434

CIAH 4.01885171

CIAI 7.66965186

CIAK 3.81362330

CIAL 6.41226582

CIAM 5.97889297

CIAN 3.57453049

CIAP 3.92219256

CIAQ 3.80790199

CIAR 3.85207096

CIAS 4.04001860

CIAT 4.80542980

CIAV 6.78093484

CIAW 4.69987544

CIAY 4.79460176

CICA 8.16996393

CICC 5.87097180

CICD 6.49686286

CICE 7.10559188

CICF 8.87014943

CICG 6.46189891

CICH 6.69807050

CICI 13.07016129

CICK 7.45754121

CICL 10.07436035

CICM 9.56824545

CICN 7.16688081

CICP 7.26381546

CICQ 7.40486385

CICR 7.22843210

CICS 7.52806049

CICT 8.48741281

CICV 10.76764137

CICW 7.26348834

CICY 8.08435465

CIDA 2.78058707

CIDC 3.41648893

CIDD 1.50731252

CIDE 1.89877632

CIDF 3.56688261

CIDG 1.50532614

CIDH 2.25734250

CIDI 5.24341137

CIDK 2.07173788

CIDL 4.21434027

CIDM 3.86971716

CIDN 1.93490898

CIDP 2.20600407

CIDQ 2.09551318

CIDR 2.09982197

CIDS 2.25974465

CIDT 2.86370139

CIDV 4.49090836

CIDW 2.84447217

CIDY 2.92099188

CIEA 2.78716302

CIEC 3.46877570

CIED 1.58160456

CIEE 1.89061968

CIEF 3.59800521

CIEG 1.68963136

CIEH 2.30270130

CIEI 5.26675496

CIEK 2.07016769

CIEL 4.24920520

CIEM 3.83060696

CIEN 1.97277472

CIEP 2.24145512

CIEQ 2.08632396

CIER 2.12912856

CIES 2.28981311

CIET 2.88808326

CIEV 4.52875988

CIEW 2.90153737

CIEY 2.96526119

CIFA 4.33358873

CIFC 4.66125099

CIFD 2.73161811

CIFE 3.13986394

CIFF 4.92486384

CIFG 3.00041177

CIFH 3.45193062

CIFI 6.72057741

CIFK 3.41851732

CIFL 5.68139955

CIFM 5.29655461

CIFN 3.22240586

CIFP 3.39297946

CIFQ 3.45496508

CIFR 3.32699000

CIFS 3.63780706

CIFT 4.28502918

CIFV 5.98477909

CIFW 3.87160539

CIFY 4.24574034

CIGA 3.84431436

CIGC 3.93792324

CIGD 2.04058403

CIGE 2.59592576

CIGF 4.58131763

CIGG 2.17352262

CIGH 2.99578296

CIGI 6.37930594

CIGK 2.79514962

CIGL 5.32686765

CIGM 4.94801812

CIGN 2.72475794

CIGP 2.84745497

CIGQ 3.04082572

CIGR 2.81486709

CIGS 3.11278930

CIGT 3.79719743

CIGV 5.59756341

CIGW 3.65491492

CIGY 3.82513204

CIHA 3.56920072

CIHC 3.63117080

CIHD 2.26496601

CIHE 2.68784635

CIHF 4.24333619

CIHG 2.19740814

CIHH 2.76587643

CIHI 6.11118083

CIHK 2.83608016

CIHL 5.03588715

CIHM 4.62509466

CIHN 2.61293169

CIHP 2.84015447

CIHQ 2.86359673

CIHR 2.83640064

CIHS 3.00883137

CIHT 3.64234317

CIHV 5.30221854

CIHW 3.43560998

CIHY 3.68004457

CIIA 4.55970854

CIIC 5.09343507

CIID 2.91037883

CIIE 3.43569161

CIIF 5.30268400

CIIG 3.21290855

CIIH 3.76846285

CIII 6.98309615

CIIK 3.71311590

CIIL 6.00691717

CIIM 5.57867688

CIIN 3.45045973

CIIP 3.65200593

CIIQ 3.76532952

CIIR 3.65245109

CIIS 3.88335477

CIIT 4.51179306

CIIV 6.25867785

CIIW 4.31718563

CIIY 4.55832949

CIKA 3.03591928

CIKC 3.72454190

CIKD 1.70281122

CIKE 2.11729501

CIKF 3.87359153

CIKG 1.81996129

CIKH 2.48883753

CIKI 5.59609003

CIKK 2.27679429

CIKL 4.52080818

CIKM 4.04620944

CIKN 2.11625038

CIKP 2.35599225

CIKQ 2.23323414

CIKR 2.27678034

CIKS 2.43549335

CIKT 3.06813017

CIKV 4.79360327

CIKW 2.92651257

CIKY 3.18278808

CILA 4.39845713

CILC 4.88835673

CILD 2.77940993

CILE 3.17831178

CILF 5.04021748

CILG 3.04057516

CILH 3.59276263

CILI 6.77227254

CILK 3.41268185

CILL 5.76668768

CILM 5.36435666

CILN 3.20717412

CILP 3.45151532

CILQ 3.47381182

CILR 3.43713016

CILS 3.73846411

CILT 4.34829879

CILV 6.08227410

CILW 4.10456492

CILY 4.30600539

CIMA 4.32008692

CIMC 4.83264305

CIMD 2.67647017

CIME 3.29380933

CIMF 4.98398618

CIMG 2.96427782

CIMH 3.49571462

CIMI 6.74711195

CIMK 3.33539876

CIML 5.72654463

CIMM 5.22807225

CIMN 3.13719035

CIMP 3.48539582

CIMQ 3.35361820

CIMR 3.49547496

CIMS 3.58879290

CIMT 4.26526853

CIMV 6.00114753

CIMW 4.20659723

CIMY 4.28949915

CINA 3.45437132

CINC 4.09118086

CIND 2.06635842

CINE 2.52290758

CINF 4.30253036

CING 2.14237096

CINH 2.89631922

CINI 6.11218403

CINK 2.70222775

CINL 5.00702676

CINM 4.67536641

CINN 2.56009235

CINP 2.71088785

CINQ 2.73785908

CINR 2.68227991

CINS 2.85367645

CINT 3.50014094

CINV 5.24320367

CINW 3.41989269

CINY 3.56145380

CIPA 3.05636909

CIPC 3.34291510

CIPD 1.72837769

CIPE 2.03539018

CIPF 3.75254828

CIPG 1.72520664

CIPH 2.42760106

CIPI 5.53854082

CIPK 2.29546910

CIPL 4.51073991

CIPM 4.14609029

CIPN 2.13353376

CIPP 2.36754433

CIPQ 2.38190340

CIPR 2.39857620

CIPS 2.49427653

CIPT 3.03425684

CIPV 4.77107613

CIPW 2.95655333

CIPY 3.17147118

CIQA 3.10128255

CIQC 3.72248994

CIQD 1.83570574

CIQE 2.14007613

CIQF 3.91778375

CIQG 1.97773424

CIQH 2.64452890

CIQI 5.62197910

CIQK 2.35695985

CIQL 4.59160669

CIQM 4.16505624

CIQN 2.24640731

CIQP 2.54838731

CIQQ 2.28298728

CIQR 2.44350771

CIQS 2.53836956

CIQT 3.21005533

CIQV 4.89640689

CIQW 3.20291678

CIQY 3.31733443

CIRA 3.19766405

CIRC 3.71703468

CIRD 1.89775375

CIRE 2.31199195

CIRF 3.95126596

CIRG 1.89945159

CIRH 2.66575016

CIRI 5.82770238

CIRK 2.41347774

CIRL 4.68772610

CIRM 4.31150258

CIRN 2.23393447

CIRP 2.50283963

CIRQ 2.49494412

CIRR 2.38202090

CIRS 2.62870395

CIRT 3.26821757

CIRV 5.06661126

CIRW 3.18159098

CIRY 3.23263162

CISA 4.31754040

CISC 4.92256990

CISD 2.64580159

CISE 3.15702969

CISF 5.22343639

CISG 2.91442354

CISH 3.60498296

CISI 7.25105476

CISK 3.42878399

CISL 5.97339125

CISM 5.61307646

CISN 3.25787618

CISP 3.57045693

CISQ 3.48809731

CISR 3.45558026

CISS 3.62898520

CIST 4.37692246

CISV 6.27396938

CISW 4.25730842

CISY 4.43742563

CITA 4.23613908

CITC 4.88214664

CITD 2.57637534

CITE 3.04519186

CITF 5.08220372

CITG 2.85476857

CITH 3.54785010

CITI 6.98675111

CITK 3.36318585

CITL 5.85944015

CITM 5.45401642

CITN 3.15172127

CITP 3.42038049

CITQ 3.35074038

CITR 3.41691785

CITS 3.52705947

CITT 4.19446731

CITV 6.10662670

CITW 4.16960605

CITY 4.25316428

CIVA 4.80120825

CIVC 5.32634357

CIVD 3.08222612

CIVE 3.62922652

CIVF 5.48217218

CIVG 3.29461051

CIVH 3.98153858

CIVI 7.42759327

CIVK 3.84539151

CIVL 6.26872570

CIVM 5.87384415

CIVN 3.53710840

CIVP 3.82457381

CIVQ 3.96409456

CIVR 3.89813038

CIVS 4.02729472

CIVT 4.73643612

CIVV 6.61872289

CIVW 4.55865297

CIVY 4.70520192

CIWA 3.82578430

CIWC 3.88251182

CIWD 2.46084431

CIWE 2.73812582

CIWF 4.21350184

CIWG 2.48440450

CIWH 3.08246547

CIWI 6.13276462

CIWK 3.02116477

CIWL 5.02008451

CIWM 4.68861965

CIWN 2.77043886

CIWP 3.02495326

CIWQ 3.10899298

CIWR 3.01796004

CIWS 3.16122337

CIWT 4.08209181

CIWV 5.38196255

CIWW 3.32503668

CIWY 3.61579289

CIYA 4.07494573

CIYC 4.36955815

CIYD 2.58363801

CIYE 3.08057978

CIYF 4.71765164

CIYG 2.71958619

CIYH 3.30747851

CIYI 6.59193769

CIYK 3.35168592

CIYL 5.46981578

CIYM 5.12239986

CIYN 3.08863271

CIYP 3.21729550

CIYQ 3.29668613

CIYR 3.21545662

CIYS 3.48097953

CIYT 4.06715070

CIYV 5.81008526

CIYW 3.66721342

CIYY 3.94797577

CKAA 4.54299171

CKAC 4.10409412

CKAD 4.92879189

CKAE 5.33685072

CKAF 3.39689308

CKAG 4.53111840

CKAH 5.04215067

CKAI 3.87626415

CKAK 7.21345341

CKAL 3.93192482

CKAM 4.24175506

CKAN 5.21505581

CKAP 4.71556936

CKAQ 5.45778720

CKAR 6.12563448

CKAS 4.96262077

CKAT 4.83199870

CKAV 3.95279119

CKAW 3.67626147

CKAY 4.06059588

CKCA 8.17938778

CKCC 5.20063726

CKCD 8.52232696

CKCE 8.99002905

CKCF 6.88990933

CKCG 8.37826913

CKCH 8.21616584

CKCI 7.45754121

CKCK 13.07609863

CKCL 7.47014333

CKCM 7.90592617

CKCN 9.05420751

CKCP 8.36099190

CKCQ 9.41157002

CKCR 10.30102657

CKCS 8.76319119

CKCT 8.72666887

CKCV 7.64867781

CKCW 7.04655277

CKCY 7.56452001

CKDA 3.08579345

CKDC 2.72241974

CKDD 3.31389760

CKDE 3.67781368

CKDF 2.17258837

CKDG 2.90811849

CKDH 3.53206980

CKDI 2.69655492

CKDK 4.98083579

CKDL 2.50421867

CKDM 2.88019208

CKDN 3.57474053

CKDP 3.21647899

CKDQ 3.81196601

CKDR 4.24105130

CKDS 3.44880944

CKDT 3.31755234

CKDV 2.59293722

CKDW 2.27279961

CKDY 2.62334451

CKEA 3.12484374

CKEC 2.83473128

CKED 3.48529514

CKEE 3.69857398

CKEF 2.18439750

CKEG 3.09574407

CKEH 3.49940304

CKEI 2.58145598

CKEK 4.86875574

CKEL 2.52816896

CKEM 2.81070823

CKEN 3.68155362

CKEP 3.27811957

CKEQ 3.79028584

CKER 4.20999281

CKES 3.52910672

CKET 3.40779336

CKEV 2.65026612

CKEW 2.37144391

CKEY 2.69820214

CKFA 3.93095877

CKFC 3.33469092

CKFD 4.41451838

CKFE 4.79517282

CKFF 2.75162681

CKFG 3.78962093

CKFH 4.45454343

CKFI 3.29985906

CKFK 6.48516353

CKFL 3.27077777

CKFM 3.63405549

CKFN 4.63475252

CKFP 4.03023706

CKFQ 4.96618950

CKFR 5.45616461

CKFS 4.42227831

CKFT 4.30216783

CKFV 3.34120938

CKFW 3.00469960

CKFY 3.47396880

CKGA 3.48565959

CKGC 2.84033842

CKGD 3.77478553

CKGE 4.24783014

CKGF 2.44369391

CKGG 3.22029798

CKGH 3.94055463

CKGI 2.95979302

CKGK 6.01954253

CKGL 3.00024822

CKGM 3.34212474

CKGN 4.19232054

CKGP 3.54111593

CKGQ 4.42531725

CKGR 5.00016582

CKGS 3.92361576

CKGT 3.79415121

CKGV 2.98591255

CKGW 2.68331175

CKGY 3.07630504

CKHA 3.79082330

CKHC 2.98560012

CKHD 4.06880259

CKHE 4.42767368

CKHF 2.62855591

CKHG 3.61484726

CKHH 3.88308253

CKHI 3.11573092

CKHK 6.02725992

CKHL 3.20311169

CKHM 3.47484927

CKHN 4.36408026

CKHP 3.91867478

CKHQ 4.64009972

CKHR 5.14804510

CKHS 4.22987328

CKHT 4.04092327

CKHV 3.22415679

CKHW 2.91557019

CKHY 3.16529907

CKIA 4.14600255

CKIC 3.63228896

CKID 4.71526962

CKIE 5.11212031

CKIF 3.12694012

CKIG 4.07026676

CKIH 4.70047515

CKII 3.46177396

CKIK 6.65609207

CKIL 3.53872558

CKIM 3.88603398

CKIN 4.93704501

CKIP 4.39294488

CKIQ 5.20559657

CKIR 5.71141289

CKIS 4.70357913

CKIT 4.51671624

CKIV 3.50822835

CKIW 3.40566306

CKIY 3.84141508

CKKA 3.49082639

CKKC 3.21528194

CKKD 3.81212804

CKKE 4.09392648

CKKF 2.49550117

CKKG 3.45234985

CKKH 3.79322617

CKKI 2.95519584

CKKK 5.26454914

CKKL 2.93310400

CKKM 3.29249730

CKKN 4.03633540

CKKP 3.55074747

CKKQ 4.16034763

CKKR 4.61404911

CKKS 3.90962448

CKKT 3.77991156

CKKV 2.97107387

CKKW 2.62148567

CKKY 3.04521792

CKLA 4.07071015

CKLC 3.47779541

CKLD 4.54477236

CKLE 4.91632372

CKLF 2.91186470

CKLG 4.01263132

CKLH 4.56773888

CKLI 3.33096941

CKLK 6.50713217

CKLL 3.34159327

CKLM 3.76143536

CKLN 4.79123132

CKLP 4.22716072

CKLQ 5.06389032

CKLR 5.55594228

CKLS 4.57158193

CKLT 4.36777852

CKLV 3.37884038

CKLW 3.12509351

CKLY 3.68506581

CKMA 4.00572215

CKMC 3.61449359

CKMD 4.53494200

CKME 4.95274970

CKMF 2.88875166

CKMG 3.92428767

CKMH 4.47258115

CKMI 3.39070541

CKMK 6.42268928

CKML 3.37956954

CKMM 3.57052894

CKMN 4.67833762

CKMP 4.15883576

CKMQ 4.91252975

CKMR 5.53445513

CKMS 4.42970636

CKMT 4.28646986

CKMV 3.43640793

CKMW 3.30453499

CKMY 3.69460536

CKNA 3.61837680

CKNC 3.32404820

CKND 3.94955734

CKNE 4.29450196

CKNF 2.58984549

CKNG 3.46961227

CKNH 4.12855808

CKNI 3.09380409

CKNK 5.81592727

CKNL 3.10705327

CKNM 3.40204837

CKNN 4.19539423

CKNP 3.72136278

CKNQ 4.43046517

CKNR 4.98107454

CKNS 4.02677157

CKNT 3.85622054

CKNV 3.13957210

CKNW 2.72582349

CKNY 3.15689123

CKPA 3.11371062

CKPC 2.59731560

CKPD 3.37826290

CKPE 3.72131814

CKPF 2.08050539

CKPG 2.90560656

CKPH 3.41535590

CKPI 2.53163367

CKPK 5.27080469

CKPL 2.49676179

CKPM 2.97100956

CKPN 3.54220268

CKPP 3.14195189

CKPQ 3.91594028

CKPR 4.35404151

CKPS 3.50961721

CKPT 3.33898490

CKPV 2.57708054

CKPW 2.18275694

CKPY 2.50387420

CKQA 3.36160601

CKQC 3.03413440

CKQD 3.76707812

CKQE 4.00546737

CKQF 2.37571765

CKQG 3.32805917

CKQH 3.76522258

CKQI 2.78100538

CKQK 5.38284983

CKQL 2.80024030

CKQM 3.06100277

CKQN 3.89536024

CKQP 3.57008949

CKQQ 4.08290693

CKQR 4.64807395

CKQS 3.75439339

CKQT 3.68921737

CKQV 2.83640861

CKQW 2.69526071

CKQY 2.88362551

CKRA 3.55234852

CKRC 3.03493813

CKRD 3.76904962

CKRE 4.14674771

CKRF 2.37768431

CKRG 3.39792728

CKRH 3.87168517

CKRI 2.86959540

CKRK 5.67826985

CKRL 2.86812603

CKRM 3.21467422

CKRN 4.07729941

CKRP 3.65369527

CKRQ 4.34413814

CKRR 4.68923559

CKRS 3.92939811

CKRT 3.79150521

CKRV 2.98397678

CKRW 2.68391900

CKRY 2.89645602

CKSA 4.25408340

CKSC 3.96546631

CKSD 4.59558858

CKSE 4.97292951

CKSF 3.26798694

CKSG 4.21530384

CKSH 4.77874980

CKSI 3.75739826

CKSK 6.93037198

CKSL 3.72808263

CKSM 4.06031236

CKSN 4.94847084

CKSP 4.48049911

CKSQ 5.15897287

CKSR 5.88671723

CKSS 4.67315550

CKST 4.53953307

CKSV 3.78593227

CKSW 3.48351204

CKSY 3.79029276

CKTA 4.10045179

CKTC 3.72374465

CKTD 4.46769043

CKTE 4.83197327

CKTF 3.02653629

CKTG 4.04566087

CKTH 4.56597859

CKTI 3.50126854

CKTK 6.64978518

CKTL 3.55760387

CKTM 3.82238493

CKTN 4.73205180

CKTP 4.27063181

CKTQ 4.95304452

CKTR 5.65499605

CKTS 4.52215629

CKTT 4.35784408

CKTV 3.57180002

CKTW 3.23753124

CKTY 3.65287723

CKVA 4.44351228

CKVC 3.79138436

CKVD 4.91909451

CKVE 5.31486175

CKVF 3.21563976

CKVG 4.33927240

CKVH 4.95584790

CKVI 3.65061826

CKVK 7.07035177

CKVL 3.74357298

CKVM 4.07546056

CKVN 5.08250514

CKVP 4.66724196

CKVQ 5.44228180

CKVR 6.04403306

CKVS 4.89588400

CKVT 4.72563151

CKVV 3.73066999

CKVW 3.58472475

CKVY 3.90660215

CKWA 3.46561585

CKWC 2.82190365

CKWD 3.83764145

CKWE 4.24045384

CKWF 2.27842455

CKWG 3.32085002

CKWH 3.77743856

CKWI 2.87405292

CKWK 6.01555271

CKWL 2.79449208

CKWM 3.31262623

CKWN 4.07636967

CKWP 3.49075541

CKWQ 4.65196298

CKWR 4.90137823

CKWS 3.92658580

CKWT 3.80058763

CKWV 2.91547223

CKWW 2.50543661

CKWY 2.82935968

CKYA 3.95533234

CKYC 3.46249524

CKYD 4.36468206

CKYE 4.77480902

CKYF 2.79251716

CKYG 3.87232977

CKYH 4.40381417

CKYI 3.42096575

CKYK 6.48139843

CKYL 3.41881204

CKYM 3.76306654

CKYN 4.64917535

CKYP 3.96960177

CKYQ 4.95562650

CKYR 5.47349395

CKYS 4.42493926

CKYT 4.30510165

CKYV 3.44918392

CKYW 2.91174368

CKYY 3.36400395

CLAA 4.61725137

CLAC 5.28979506

CLAD 3.12853648

CLAE 3.60789220

CLAF 5.90018304

CLAG 3.36672630

CLAH 4.23072625

CLAI 6.44539982

CLAK 3.86423940

CLAL 7.21503937

CLAM 6.18217979

CLAN 3.59795995

CLAP 3.82068533

CLAQ 4.04560364

CLAR 3.99928867

CLAS 3.94554302

CLAT 4.50527686

CLAV 5.93101918

CLAW 4.95140076

CLAY 4.94338776

CLCA 7.94258303

CLCC 5.67414395

CLCD 6.53245028

CLCE 7.20582656

CLCF 9.36778646

CLCG 6.74631862

CLCH 7.10289973

CLCI 10.07436035

CLCK 7.47014333

CLCL 12.74707960

CLCM 9.96480622

CLCN 7.09267965

CLCP 7.43075064

CLCQ 7.78703739

CLCR 7.52497686

CLCS 7.41861403

CLCT 7.96705748

CLCV 9.49033890

CLCW 7.88717649

CLCY 8.27300660

CLDA 2.69423778

CLDC 3.24574746

CLDD 1.55254862

CLDE 1.91758511

CLDF 3.80157786

CLDG 1.52455561

CLDH 2.39712081

CLDI 4.28090612

CLDK 2.19989407

CLDL 4.92846361

CLDM 4.22377171

CLDN 1.92028671

CLDP 2.14968250

CLDQ 2.24042073

CLDR 2.26779111

CLDS 2.21403735

CLDT 2.62056196

CLDV 3.81025640

CLDW 3.02624711

CLDY 2.98482134

CLEA 2.66376207

CLEC 3.36514842

CLED 1.65694742

CLEE 1.97517281

CLEF 3.84062570

CLEG 1.64972968

CLEH 2.39499423

CLEI 4.34007311

CLEK 2.15802155

CLEL 4.90251493

CLEM 4.02954245

CLEN 2.01670463

CLEP 2.18673744

CLEQ 2.24573108

CLER 2.31575715

CLES 2.22180973

CLET 2.65414796

CLEV 3.83612489

CLEW 3.11983818

CLEY 3.06744344

CLFA 4.20970730

CLFC 4.50039884

CLFD 2.74517437

CLFE 3.17817925

CLFF 5.17357751

CLFG 2.96028743

CLFH 3.64236464

CLFI 5.73474881

CLFK 3.47373559

CLFL 6.35004909

CLFM 5.44956396

CLFN 3.19395297

CLFP 3.38775589

CLFQ 3.65108398

CLFR 3.49753769

CLFS 3.56517159

CLFT 3.97905542

CLFV 5.24390543

CLFW 4.08804346

CLFY 4.31743132

CLGA 3.62270880

CLGC 3.76420406

CLGD 2.11426128

CLGE 2.61540263

CLGF 4.72500040

CLGG 2.23537444

CLGH 3.07388998

CLGI 5.31731185

CLGK 2.81572904

CLGL 5.96960525

CLGM 5.12549682

CLGN 2.58987972

CLGP 2.75688384

CLGQ 3.10523633

CLGR 2.92941998

CLGS 2.91833304

CLGT 3.40621520

CLGV 4.78509871

CLGW 3.74032146

CLGY 3.85827540

CLHA 3.47806438

CLHC 3.50517570

CLHD 2.36466689

CLHE 2.67257494

CLHF 4.55901926

CLHG 2.24990006

CLHH 2.84039465

CLHI 5.14075051

CLHK 2.84348419

CLHL 5.84543217

CLHM 4.84409058

CLHN 2.61561331

CLHP 2.84391072

CLHQ 3.08859220

CLHR 3.00721253

CLHS 3.03895547

CLHT 3.38671280

CLHV 4.60674237

CLHW 3.56700273

CLHY 3.72926877

CLIA 4.39490714

CLIC 4.92163628

CLID 3.02225758

CLIE 3.46814156

CLIF 5.51872884

CLIG 3.20739136

CLIH 3.92786998

CLII 5.96325498

CLIK 3.73717118

CLIL 6.64220822

CLIM 5.74662069

CLIN 3.43784339

CLIP 3.58728974

CLIQ 3.83668047

CLIR 3.75045767

CLIS 3.78307870

CLIT 4.21529041

CLIV 5.45526077

CLIW 4.57137098

CLIY 4.68181704

CLKA 2.89377115

CLKC 3.59764484

CLKD 1.77648847

CLKE 2.15539584

CLKF 4.10677658

CLKG 1.79060677

CLKH 2.53276743

CLKI 4.58532441

CLKK 2.33167084

CLKL 5.23228951

CLKM 4.27793077

CLKN 2.17709367

CLKP 2.28093412

CLKQ 2.45251272

CLKR 2.42640644

CLKS 2.39559166

CLKT 2.76669611

CLKV 4.01879382

CLKW 3.11145479

CLKY 3.27734406

CLLA 4.33045376

CLLC 4.78233294

CLLD 2.88126604

CLLE 3.27808421

CLLF 5.35794808

CLLG 3.09516738

CLLH 3.82594768

CLLI 5.82805208

CLLK 3.53113935

CLLL 6.46806999

CLLM 5.62291925

CLLN 3.28169235

CLLP 3.42269333

CLLQ 3.67835385

CLLR 3.62214792

CLLS 3.67312531

CLLT 4.13421819

CLLV 5.36197546

CLLW 4.41243974

CLLY 4.51673129

CLMA 4.19367624

CLMC 4.67322799

CLMD 2.81772995

CLME 3.17859408

CLMF 5.29931145

CLMG 3.01121021

CLMH 3.67398077

CLMI 5.74317100

CLMK 3.46262548

CLML 6.43789919

CLMM 5.44677656

CLMN 3.26343061

CLMP 3.60416096

CLMQ 3.55184458

CLMR 3.50866135

CLMS 3.51282196

CLMT 3.99521108

CLMV 5.24830515

CLMW 4.38590391

CLMY 4.46502594

CLNA 3.35074404

CLNC 3.87073991

CLND 2.09744145

CLNE 2.48161735

CLNF 4.54659988

CLNG 2.15263465

CLNH 2.96496088

CLNI 5.03333985

CLNK 2.70576396

CLNL 5.82602855

CLNM 4.82903716

CLNN 2.53394382

CLNP 2.66999633

CLNQ 2.90904338

CLNR 2.80321289

CLNS 2.75876638

CLNT 3.15531683

CLNV 4.46960031

CLNW 3.53490569

CLNY 3.70575403

CLPA 2.89724670

CLPC 3.18513205

CLPD 1.75212972

CLPE 2.03129858

CLPF 3.95567678

CLPG 1.73876290

CLPH 2.54120449

CLPI 4.50077062

CLPK 2.25396353

CLPL 5.19423601

CLPM 4.25487134

CLPN 2.07054847

CLPP 2.33818981

CLPQ 2.45884836

CLPR 2.31512680

CLPS 2.38674479

CLPT 2.73963233

CLPV 4.01389926

CLPW 2.99465415

CLPY 3.19437630

CLQA 3.00675747

CLQC 3.67940420

CLQD 1.92296308

CLQE 2.23131175

CLQF 4.18761487

CLQG 1.94449629

CLQH 2.76440766

CLQI 4.64811969

CLQK 2.42325757

CLQL 5.35016222

CLQM 4.41994367

CLQN 2.26495190

CLQP 2.49787137

CLQQ 2.54367774

CLQR 2.57337056

CLQS 2.51539994

CLQT 2.97554825

CLQV 4.16870808

CLQW 3.43508025

CLQY 3.43774742

CLRA 3.12293610

CLRC 3.62776270

CLRD 2.07762497

CLRE 2.35382948

CLRF 4.19514765

CLRG 1.93856448

CLRH 2.77268986

CLRI 4.76527422

CLRK 2.48324765

CLRL 5.52395855

CLRM 4.53504004

CLRN 2.33889381

CLRP 2.50807582

CLRQ 2.63573557

CLRR 2.56588107

CLRS 2.74997594

CLRT 3.01843992

CLRV 4.29449812

CLRW 3.29519441

CLRY 3.34939440

CLSA 4.16671176

CLSC 4.77519242

CLSD 2.75176945

CLSE 3.18739487

CLSF 5.52710206

CLSG 2.98753082

CLSH 3.84169581

CLSI 6.03455617

CLSK 3.50049706

CLSL 6.92599085

CLSM 5.86106557

CLSN 3.29770169

CLSP 3.45398930

CLSQ 3.70816722

CLSR 3.58119907

CLSS 3.54460088

CLST 4.04629859

CLSV 5.45774693

CLSW 4.49226658

CLSY 4.57955477

CLTA 4.08491342

CLTC 4.75142570

CLTD 2.64974264

CLTE 3.04847978

CLTF 5.35446832

CLTG 2.84688095

CLTH 3.67623033

CLTI 5.85843163

CLTK 3.33249010

CLTL 6.67366382

CLTM 5.64802220

CLTN 3.14120339

CLTP 3.38088354

CLTQ 3.50463049

CLTR 3.49832246

CLTS 3.39481151

CLTT 3.86802446

CLTV 5.28358034

CLTW 4.41647601

CLTY 4.43394505

CLVA 4.63072600

CLVC 5.07269084

CLVD 3.17410008

CLVE 3.65035426

CLVF 5.71316372

CLVG 3.32473462

CLVH 4.10297786

CLVI 6.20375803

CLVK 3.83932375

CLVL 6.99532531

CLVM 6.02521395

CLVN 3.52495016

CLVP 3.80825365

CLVQ 4.04916948

CLVR 3.97843496

CLVS 3.89869859

CLVT 4.37982810

CLVV 5.70815241

CLVW 4.81898901

CLVY 4.83468541

CLWA 3.69484968

CLWC 3.69903123

CLWD 2.35721703

CLWE 2.70488786

CLWF 4.47982417

CLWG 2.48206055

CLWH 3.27581992

CLWI 5.08925834

CLWK 3.03373050

CLWL 5.80206843

CLWM 4.91841610

CLWN 2.75581659

CLWP 2.88480010

CLWQ 3.24240489

CLWR 3.16049335

CLWS 3.09823808

CLWT 3.46106531

CLWV 4.62954549

CLWW 3.63972203

CLWY 3.65271601

CLYA 3.87891550

CLYC 4.22286215

CLYD 2.59366751

CLYE 3.09950568

CLYF 5.05692657

CLYG 2.67324465

CLYH 3.52032489

CLYI 5.56161133

CLYK 3.40417784

CLYL 6.28887453

CLYM 5.33148214

CLYN 3.06746759

CLYP 3.26528134

CLYQ 3.48466241

CLYR 3.33975994

CLYS 3.37062638

CLYT 3.77183662

CLYV 5.06875938

CLYW 3.85904202

CLYY 4.13046651

CMAA 4.76349171

CMAC 5.32110852

CMAD 3.49207456

CMAE 3.95599420

CMAF 5.62725589

CMAG 3.78795594

CMAH 4.39277768

CMAI 6.10430678

CMAK 4.21171558

CMAL 6.24390243

CMAM 7.63647930

CMAN 4.10045955

CMAP 3.77089811

CMAQ 4.58886688

CMAR 4.17840218

CMAS 4.37657771

CMAT 4.76978821

CMAV 5.59465880

CMAW 4.91252050

CMAY 4.90725593

CMCA 8.22014583

CMCC 5.74054444

CMCD 6.76227909

CMCE 7.54170790

CMCF 8.90405655

CMCG 7.11072914

CMCH 7.28444609

CMCI 9.56824545

CMCK 7.90592617

CMCL 9.96480622

CMCM 13.59573871

CMCN 7.39687699

CMCP 7.25399080

CMCQ 8.34132314

CMCR 7.77250662

CMCS 7.85736547

CMCT 8.38885394

CMCV 9.07331560

CMCW 7.66257439

CMCY 8.30289981

CMDA 2.87223075

CMDC 3.30826321

CMDD 1.91991895

CMDE 2.23617641

CMDF 3.66327461

CMDG 1.93171953

CMDH 2.68860164

CMDI 3.92484638

CMDK 2.51605318

CMDL 4.13378352

CMDM 5.55914495

CMDN 2.41164576

CMDP 2.33283718

CMDQ 2.80286288

CMDR 2.53264163

CMDS 2.65094861

CMDT 2.98085194

CMDV 3.53667428

CMDW 3.02575307

CMDY 3.13879865

CMEA 2.86674852

CMEC 3.36994868

CMED 2.03496499

CMEE 2.21087699

CMEF 3.56588246

CMEG 2.12616931

CMEH 2.72499029

CMEI 3.97312689

CMEK 2.49364948

CMEL 4.06791601

CMEM 5.06435527

CMEN 2.45361589

CMEP 2.20861121

CMEQ 2.69386960

CMER 2.59616393

CMES 2.61405195

CMET 2.88295103

CMEV 3.55161141

CMEW 3.00035610

CMEY 3.14654208

CMFA 4.32938981

CMFC 4.54707599

CMFD 3.05376154

CMFE 3.48289590

CMFF 4.86798499

CMFG 3.31919618

CMFH 3.77261457

CMFI 5.30722406

CMFK 3.81880799

CMFL 5.41708917

CMFM 6.61765847

CMFN 3.53201085

CMFP 3.29415244

CMFQ 4.05870094

CMFR 3.72586233

CMFS 3.87567148

CMFT 4.26941802

CMFV 4.86734660

CMFW 4.18615281

CMFY 4.29113781

CMGA 3.82196089

CMGC 3.84121938

CMGD 2.60888805

CMGE 2.94847808

CMGF 4.57497151

CMGG 2.61783792

CMGH 3.51080124

CMGI 4.97264663

CMGK 3.23061400

CMGL 5.15601479

CMGM 6.51525202

CMGN 3.15757759

CMGP 2.86208599

CMGQ 3.71960627

CMGR 3.20586657

CMGS 3.47032083

CMGT 3.71974405

CMGV 4.52171533

CMGW 3.76219522

CMGY 3.91491458

CMHA 3.70804634

CMHC 3.61451231

CMHD 2.62911096

CMHE 2.99043354

CMHF 4.34932896

CMHG 2.68681132

CMHH 3.21019172

CMHI 4.80466787

CMHK 3.28039546

CMHL 4.97496714

CMHM 6.14558164

CMHN 3.09906716

CMHP 2.88393183

CMHQ 3.61861287

CMHR 3.24985497

CMHS 3.38275733

CMHT 3.63517897

CMHV 4.38068863

CMHW 3.61689087

CMHY 3.72805893

CMIA 4.52811407

CMIC 4.94351005

CMID 3.33677222

CMIE 3.77991107

CMIF 5.23215774

CMIG 3.57790140

CMIH 4.08992140

CMII 5.56718551

CMIK 4.06352407

CMIL 5.76763450

CMIM 6.89862837

CMIN 3.84933206

CMIP 3.52170067

CMIQ 4.37110824

CMIR 3.99023646

CMIS 4.14087124

CMIT 4.45718569

CMIV 5.10542274

CMIW 4.48054461

CMIY 4.60328891

CMKA 3.00342088

CMKC 3.58389469

CMKD 2.22804651

CMKE 2.56677201

CMKF 3.78111161

CMKG 2.18021232

CMKH 2.77703362

CMKI 4.18843951

CMKK 2.67380388

CMKL 4.36207862

CMKM 5.39291394

CMKN 2.56961081

CMKP 2.35284857

CMKQ 2.95813673

CMKR 2.70803948

CMKS 2.75004077

CMKT 3.00429856

CMKV 3.68765181

CMKW 3.30043854

CMKY 3.24859175

CMLA 4.37328714

CMLC 4.75666113

CMLD 3.14930039

CMLE 3.61072499

CMLF 4.98134948

CMLG 3.39663045

CMLH 3.95775548

CMLI 5.37693827

CMLK 3.80399796

CMLL 5.54033963

CMLM 6.73006212

CMLN 3.65148942

CMLP 3.44103540

CMLQ 4.13877187

CMLR 3.76177522

CMLS 3.96488716

CMLT 4.27939215

CMLV 4.96054411

CMLW 4.23449893

CMLY 4.37448568

CMMA 4.41224192

CMMC 4.85638266

CMMD 3.23626268

CMME 3.63858895

CMMF 5.05109605

CMMG 3.48659563

CMMH 4.01403050

CMMI 5.42400785

CMMK 3.78676881

CMML 5.60104965

CMMM 6.70891342

CMMN 3.66639455

CMMP 3.49675171

CMMQ 4.07546747

CMMR 3.79356952

CMMS 3.98580248

CMMT 4.31119756

CMMV 4.97785728

CMMW 4.53988121

CMMY 4.45447823

CMNA 3.54308609

CMNC 4.10253675

CMND 2.59994105

CMNE 2.92925502

CMNF 4.35105604

CMNG 2.68622193

CMNH 3.24361006

CMNI 4.72271628

CMNK 3.12317905

CMNL 4.96037704

CMNM 6.19225033

CMNN 3.01267526

CMNP 2.78113743

CMNQ 3.48025573

CMNR 3.17022214

CMNS 3.23849073

CMNT 3.73240575

CMNV 4.41898857

CMNW 3.64424230

CMNY 3.71236104

CMPA 3.13422039

CMPC 3.36373132

CMPD 2.14781832

CMPE 2.53859917

CMPF 3.81835195

CMPG 2.20604781

CMPH 2.85680946

CMPI 4.16054585

CMPK 2.69087480

CMPL 4.37463187

CMPM 5.61234814

CMPN 2.57784906

CMPP 2.28033638

CMPQ 2.97822179

CMPR 2.63956334

CMPS 2.82365606

CMPT 3.03111316

CMPV 3.71384493

CMPW 3.18363791

CMPY 3.31211008

CMQA 3.16860725

CMQC 3.59891625

CMQD 2.30329082

CMQE 2.54691672

CMQF 3.96156112

CMQG 2.48094322

CMQH 3.00867385

CMQI 4.30075965

CMQK 2.82364296

CMQL 4.49033372

CMQM 5.54466584

CMQN 2.75631095

CMQP 2.53702312

CMQQ 3.00483655

CMQR 2.84159999

CMQS 2.88957545

CMQT 3.24358260

CMQV 3.89935054

CMQW 3.52621667

CMQY 3.43972163

CMRA 3.26813816

CMRC 3.63528117

CMRD 2.27167971

CMRE 2.64778279

CMRF 3.93886572

CMRG 2.34376688

CMRH 2.94656672

CMRI 4.38504954

CMRK 2.83442904

CMRL 4.53616312

CMRM 5.82476523

CMRN 2.74779070

CMRP 2.56212999

CMRQ 3.06324813

CMRR 2.73733119

CMRS 2.96449479

CMRT 3.30139982

CMRV 3.98485775

CMRW 3.30147132

CMRY 3.31237448

CMSA 4.40449391

CMSC 4.85943207

CMSD 3.13318560

CMSE 3.51933658

CMSF 5.29291338

CMSG 3.49200537

CMSH 3.99535945

CMSI 5.75132647

CMSK 3.81008949

CMSL 5.93992414

CMSM 7.47860173

CMSN 3.73461296

CMSP 3.51404626

CMSQ 4.33187730

CMSR 3.83616995

CMSS 3.98753594

CMST 4.34520962

CMSV 5.18877091

CMSW 4.54328669

CMSY 4.52406965

CMTA 4.33616228

CMTC 4.78012073

CMTD 3.09469122

CMTE 3.40050215

CMTF 5.12052666

CMTG 3.38096087

CMTH 3.98189706

CMTI 5.54766416

CMTK 3.69646984

CMTL 5.72233765

CMTM 7.12538551

CMTN 3.60048247

CMTP 3.41524825

CMTQ 4.09851111

CMTR 3.75626358

CMTS 3.88967012

CMTT 4.20052501

CMTV 5.01325358

CMTW 4.42127627

CMTY 4.42724967

CMVA 4.77114422

CMVC 5.24102344

CMVD 3.54981119

CMVE 4.00831419

CMVF 5.45387899

CMVG 3.73892581

CMVH 4.35546455

CMVI 5.80350232

CMVK 4.21657210

CMVL 6.03885252

CMVM 7.37277214

CMVN 3.98142369

CMVP 3.72952347

CMVQ 4.66372366

CMVR 4.22965390

CMVS 4.29490041

CMVT 4.70621291

CMVV 5.34733858

CMVW 4.69749860

CMVY 4.73964143

CMWA 3.81442011

CMWC 3.82278461

CMWD 2.64212520

CMWE 3.08933171

CMWF 4.23866353

CMWG 2.79121627

CMWH 3.45639143

CMWI 4.87493388

CMWK 3.49617686

CMWL 4.82394220

CMWM 6.13293495

CMWN 3.39917873

CMWP 2.79119665

CMWQ 3.73084646

CMWR 3.29653813

CMWS 3.65062656

CMWT 4.24347314

CMWV 4.36886539

CMWW 3.63184846

CMWY 3.61873654

CMYA 4.10094840

CMYC 4.27073112

CMYD 2.92164440

CMYE 3.38986828

CMYF 4.68648292

CMYG 3.15167518

CMYH 3.76083894

CMYI 5.28136549

CMYK 3.74945863

CMYL 5.31017952

CMYM 6.64568462

CMYN 3.46785298

CMYP 3.27658586

CMYQ 3.97706879

CMYR 3.57894084

CMYS 3.73849600

CMYT 4.05098552

CMYV 4.76493822

CMYW 3.84849431

CMYY 4.00795037

CNAA 4.29629492

CNAC 4.54588799

CNAD 5.69950046

CNAE 4.87119668

CNAF 3.51946002

CNAG 5.06499165

CNAH 5.44009848

CNAI 3.52885577

CNAK 5.00458374

CNAL 3.51519448

CNAM 3.96424709

CNAN 7.55341638

CNAP 4.37521641

CNAQ 4.93642838

CNAR 4.75244846

CNAS 5.31577094

CNAT 4.92106987

CNAV 3.61820399

CNAW 3.55233747

CNAY 4.16856128

CNCA 8.08784394

CNCC 5.98499095

CNCD 10.00076283

CNCE 8.73245105

CNCF 7.04034114

CNCG 9.18490396

CNCH 8.93841089

CNCI 7.16688081

CNCK 9.05420751

CNCL 7.09267965

CNCM 7.39687699

CNCN 13.91081082

CNCP 8.28542161

CNCQ 8.85349229

CNCR 8.61320890

CNCS 9.41615967

CNCT 9.00714417

CNCV 7.37032118

CNCW 6.85012095

CNCY 7.93469734

CNDA 2.86689044

CNDC 3.06355740

CNDD 3.88166402

CNDE 3.29449474

CNDF 2.23597738

CNDG 3.31637034

CNDH 3.62387450

CNDI 2.17937320

CNDK 3.40379767

CNDL 2.08255073

CNDM 2.48436415

CNDN 5.40509969

CNDP 3.04700149

CNDQ 3.38127931

CNDR 3.14698792

CNDS 3.70015652

CNDT 3.37846802

CNDV 2.24062159

CNDW 2.25053254

CNDY 2.53350312

CNEA 2.77566590

CNEC 2.95729823

CNED 3.91795595

CNEE 3.26389011

CNEF 2.07445490

CNEG 3.45437837

CNEH 3.53473226

CNEI 2.07015082

CNEK 3.32938666

CNEL 2.07030306

CNEM 2.48177982

CNEN 5.09624788

CNEP 3.09331524

CNEQ 3.27374403

CNER 3.12472415

CNES 3.64229243

CNET 3.29414363

CNEV 2.17848933

CNEW 2.04456333

CNEY 2.60738891

CNFA 3.73577609

CNFC 3.73868860

CNFD 5.13304703

CNFE 4.37752678

CNFF 2.87345144

CNFG 4.26776586

CNFH 4.73815122

CNFI 2.97903115

CNFK 4.60119948

CNFL 2.92236402

CNFM 3.27475343

CNFN 6.65135012

CNFP 3.84086000

CNFQ 4.48028112

CNFR 4.23752295

CNFS 4.72193933

CNFT 4.35873407

CNFV 3.05785617

CNFW 2.96732564

CNFY 3.51591728

CNGA 3.43466602

CNGC 3.57696077

CNGD 4.58750693

CNGE 3.93371685

CNGF 2.74530320

CNGG 3.72714017

CNGH 4.37189010

CNGI 2.80212482

CNGK 4.11250576

CNGL 2.70674822

CNGM 3.24062043

CNGN 6.40205898

CNGP 3.44943555

CNGQ 4.14078296

CNGR 3.82861003

CNGS 4.33201488

CNGT 3.95094979

CNGV 2.82895696

CNGW 2.64593778

CNGY 3.31723224

CNHA 3.53916197

CNHC 3.21126056

CNHD 4.69039170

CNHE 3.98893716

CNHF 2.67364410

CNHG 4.05664113

CNHH 4.27147247

CNHI 2.75642885

CNHK 4.15287319

CNHL 2.70498192

CNHM 3.11554721

CNHN 6.21693143

CNHP 3.54167071

CNHQ 4.01331735

CNHR 3.89453770

CNHS 4.42516007

CNHT 4.00354930

CNHV 2.81846182

CNHW 2.72619313

CNHY 3.24656960

CNIA 3.97096781

CNIC 4.09673626

CNID 5.28606554

CNIE 4.69756839

CNIF 3.16817599

CNIG 4.55524332

CNIH 4.93485281

CNII 3.09829623

CNIK 4.74424898

CNIL 3.12145592

CNIM 3.51886980

CNIN 6.82543242

CNIP 4.11903431

CNIQ 4.69824943

CNIR 4.45654253

CNIS 4.97515540

CNIT 4.51085325

CNIV 3.14207262

CNIW 3.19075091

CNIY 3.81501223

CNKA 3.01247166

CNKC 3.29410099

CNKD 4.16998310

CNKE 3.50775852

CNKF 2.39923351

CNKG 3.71726972

CNKH 3.81894904

CNKI 2.35233675

CNKK 3.61942928

CNKL 2.33160104

CNKM 2.61769342

CNKN 5.33742211

CNKP 3.14056424

CNKQ 3.48457190

CNKR 3.39225057

CNKS 3.87982373

CNKT 3.47419054

CNKV 2.44404436

CNKW 2.52521802

CNKY 2.86241352

CNLA 3.82243940

CNLC 3.90508971

CNLD 5.10253396

CNLE 4.42614687

CNLF 2.96877068

CNLG 4.43566441

CNLH 4.78309431

CNLI 2.97166735

CNLK 4.60440207

CNLL 2.90980846

CNLM 3.41678007

CNLN 6.67969276

CNLP 3.89425634

CNLQ 4.53250451

CNLR 4.33475648

CNLS 4.79049554

CNLT 4.40901439

CNLV 3.01539859

CNLW 2.99461014

CNLY 3.57295387

CNMA 3.81008610

CNMC 3.96568492

CNMD 5.05522319

CNME 4.40178834

CNMF 2.99576761

CNMG 4.46344759

CNMH 4.71993666

CNMI 3.06382483

CNMK 4.48688630

CNML 2.98855862

CNMM 3.38115188

CNMN 6.65367471

CNMP 4.24509314

CNMQ 4.42126235

CNMR 4.28138340

CNMS 4.72248099

CNMT 4.38659941

CNMV 3.10952735

CNMW 3.25008751

CNMY 3.67925770

CNNA 3.42624913

CNNC 3.69655003

CNND 4.63207545

CNNE 3.88134992

CNNF 2.75538493

CNNG 3.99846425

CNNH 4.28467758

CNNI 2.79077025

CNNK 4.03702943

CNNL 2.71737756

CNNM 3.19160969

CNNN 6.17694999

CNNP 3.47058517

CNNQ 3.98136644

CNNR 3.77115010

CNNS 4.31688072

CNNT 3.99671359

CNNV 2.81979841

CNNW 2.68844953

CNNY 3.28944226

CNPA 3.06840974

CNPC 3.50104795

CNPD 4.13443805

CNPE 3.46698564

CNPF 2.22370367

CNPG 3.40810721

CNPH 3.74055202

CNPI 2.26133661

CNPK 3.58123523

CNPL 2.32576326

CNPM 2.69416966

CNPN 5.57521804

CNPP 3.11177343

CNPQ 3.48593523

CNPR 3.51287044

CNPS 3.91970222

CNPT 3.49690073

CNPV 2.34553402

CNPW 2.21577230

CNPY 2.86703816

CNQA 3.11045276

CNQC 3.43667260

CNQD 4.27653152

CNQE 3.56816280

CNQF 2.40873301

CNQG 3.77611203

CNQH 3.88553002

CNQI 2.33289405

CNQK 3.69375755

CNQL 2.30007570

CNQM 2.80861592

CNQN 5.49766595

CNQP 3.27637577

CNQQ 3.55154412

CNQR 3.48560476

CNQS 3.97122998

CNQT 3.61611570

CNQV 2.46116501

CNQW 2.55835107

CNQY 2.95400772

CNRA 3.20543018

CNRC 3.26059857

CNRD 4.34445609

CNRE 3.61402925

CNRF 2.33541155

CNRG 3.74202918

CNRH 3.95095612

CNRI 2.33852699

CNRK 3.88744217

CNRL 2.31514284

CNRM 2.72761661

CNRN 5.83639205

CNRP 3.29935947

CNRQ 3.63797008

CNRR 3.49514537

CNRS 4.02847805

CNRT 3.64764281

CNRV 2.48861317

CNRW 2.39500626

CNRY 2.88292879

CNSA 4.13602705

CNSC 4.40169373

CNSD 5.39978525

CNSE 4.63090544

CNSF 3.46919433

CNSG 4.77567853

CNSH 5.12550205

CNSI 3.44249032

CNSK 4.79356808

CNSL 3.41389911

CNSM 3.84540021

CNSN 7.19980099

CNSP 4.17258243

CNSQ 4.65917169

CNSR 4.50763255

CNSS 5.07526266

CNST 4.65745194

CNSV 3.49776339

CNSW 3.33366334

CNSY 4.08537460

CNTA 3.95414345

CNTC 4.15962280

CNTD 5.18406957

CNTE 4.45586885

CNTF 3.16973457

CNTG 4.60574034

CNTH 4.88313739

CNTI 3.19641426

CNTK 4.57914334

CNTL 3.22774559

CNTM 3.58861375

CNTN 6.86494027

CNTP 4.02997425

CNTQ 4.49816480

CNTR 4.31921179

CNTS 4.88731614

CNTT 4.43007373

CNTV 3.23803305

CNTW 3.20793463

CNTY 3.73609341

CNVA 4.21931625

CNVC 4.26702305

CNVD 5.60849274

CNVE 4.88558016

CNVF 3.34326811

CNVG 4.85149578

CNVH 5.27563051

CNVI 3.28203839

CNVK 4.95062514

CNVL 3.32933524

CNVM 3.77294452

CNVN 7.34916112

CNVP 4.35119310

CNVQ 4.93609260

CNVR 4.72471587

CNVS 5.22347861

CNVT 4.76070228

CNVV 3.38552202

CNVW 3.41077773

CNVY 4.01051731

CNWA 3.31930751

CNWC 3.04756409

CNWD 4.60387027

CNWE 3.78804237

CNWF 2.58208750

CNWG 3.77680808

CNWH 4.18367125

CNWI 2.72120173

CNWK 4.17346854

CNWL 2.42069045

CNWM 3.15126354

CNWN 6.28692322

CNWP 3.28899462

CNWQ 3.99310064

CNWR 3.67018099

CNWS 4.26242916

CNWT 3.94218380

CNWV 2.64809265

CNWW 2.44159043

CNWY 2.98790592

CNYA 3.75382153

CNYC 3.68815568

CNYD 5.08132885

CNYE 4.37858039

CNYF 2.82553252

CNYG 4.40682831

CNYH 4.71353887

CNYI 3.00090965

CNYK 4.59808962

CNYL 2.91312049

CNYM 3.40376447

CNYN 6.60935406

CNYP 3.78894193

CNYQ 4.41644231

CNYR 4.16900478

CNYS 4.74617735

CNYT 4.32307071

CNYV 3.05425794

CNYW 2.86176968

CNYY 3.52912284

CPAA 4.16648200

CPAC 3.55257946

CPAD 4.25482012

CPAE 4.09466939

CPAF 3.22567127

CPAG 4.00174288

CPAH 3.80115101

CPAI 3.48007312

CPAK 4.06490468

CPAL 3.35316114

CPAM 3.39280686

CPAN 3.91359540

CPAP 8.02079014

CPAQ 3.81973578

CPAR 3.81623832

CPAS 4.41718863

CPAT 4.06237609

CPAV 3.70703939

CPAW 3.04185783

CPAY 3.19828930

CPCA 8.70612494

CPCC 5.11310770

CPCD 8.25203669

CPCE 8.30399285

CPCF 6.77124757

CPCG 8.01307552

CPCH 7.43860227

CPCI 7.26381546

CPCK 8.36099190

CPCL 7.43075064

CPCM 7.25399080

CPCN 8.28542161

CPCP 14.78891715

CPCQ 8.27576528

CPCR 7.96962980

CPCS 8.92023868

CPCT 8.42010892

CPCV 7.68799847

CPCW 6.40483719

CPCY 6.98407741

CPDA 2.85766925

CPDC 2.41779206

CPDD 2.76641340

CPDE 2.73272839

CPDF 1.89833434

CPDG 2.54677642

CPDH 2.47385845

CPDI 2.27141443

CPDK 2.68329753

CPDL 2.08411377

CPDM 2.20116889

CPDN 2.63135753

CPDP 6.28937173

CPDQ 2.52007431

CPDR 2.36088437

CPDS 3.04058539

CPDT 2.70133851

CPDV 2.40916394

CPDW 1.91288950

CPDY 1.91346035

CPEA 2.59103447

CPEC 2.32419930

CPED 2.76759147

CPEE 2.54939276

CPEF 1.80392606

CPEG 2.53177283

CPEH 2.30400442

CPEI 1.97762028

CPEK 2.48206925

CPEL 1.85819090

CPEM 2.00211777

CPEN 2.49049944

CPEP 5.85915921

CPEQ 2.28402429

CPER 2.21122081

CPES 2.94319293

CPET 2.54146308

CPEV 2.21635542

CPEW 1.70692028

CPEY 1.78975293

CPFA 3.77783747

CPFC 2.92897712

CPFD 3.85448322

CPFE 3.77684933

CPFF 2.55718205

CPFG 3.49285537

CPFH 3.36601980

CPFI 2.96331620

CPFK 3.78501599

CPFL 2.88699055

CPFM 2.86672106

CPFN 3.64591814

CPFP 7.22464170

CPFQ 3.56732575

CPFR 3.46099567

CPFS 4.04291583

CPFT 3.64780251

CPFV 3.14905643

CPFW 2.50938836

CPFY 2.69640523

CPGA 3.46284750

CPGC 2.72835581

CPGD 3.32344502

CPGE 3.36198236

CPGF 2.59799137

CPGG 2.89199747

CPGH 3.00365874

CPGI 2.77380984

CPGK 3.33378736

CPGL 2.68181474

CPGM 2.70658018

CPGN 3.23374785

CPGP 6.79093744

CPGQ 3.17828468

CPGR 3.05865150

CPGS 3.68514462

CPGT 3.33090702

CPGV 2.90635142

CPGW 2.35560045

CPGY 2.53901660

CPHA 3.43598418

CPHC 2.58411090

CPHD 3.64749192

CPHE 3.39910709

CPHF 2.38330678

CPHG 3.19543613

CPHH 2.87493573

CPHI 2.67512556

CPHK 3.34810413

CPHL 2.66279476

CPHM 2.56640006

CPHN 3.23774236

CPHP 6.75747920

CPHQ 3.26063680

CPHR 3.17546555

CPHS 3.72932538

CPHT 3.30550401

CPHV 2.83076125

CPHW 2.50402730

CPHY 2.46835397

CPIA 3.88942312

CPIC 3.36830327

CPID 4.07565769

CPIE 3.99177423

CPIF 2.84683476

CPIG 3.69636337

CPIH 3.61448775

CPII 3.10498769

CPIK 3.95066050

CPIL 3.01170547

CPIM 2.97840988

CPIN 3.84749980

CPIP 7.46644209

CPIQ 3.72537068

CPIR 3.64370055

CPIS 4.26247293

CPIT 3.84458746

CPIV 3.29719838

CPIW 2.74619266

CPIY 2.92482817

CPKA 2.76502643

CPKC 2.47103112

CPKD 2.87814374

CPKE 2.75507797

CPKF 1.99447628

CPKG 2.66825280

CPKH 2.59378072

CPKI 2.20110683

CPKK 2.79473749

CPKL 2.06950903

CPKM 2.01701597

CPKN 2.84906467

CPKP 6.06132416

CPKQ 2.52753286

CPKR 2.51078373

CPKS 3.10430321

CPKT 2.75125734

CPKV 2.37600837

CPKW 1.88801469

CPKY 2.06533885

CPLA 3.70209091

CPLC 2.99808102

CPLD 3.89148848

CPLE 3.76940733

CPLF 2.65203663

CPLG 3.54437863

CPLH 3.38741914

CPLI 2.87833151

CPLK 3.72334901

CPLL 2.80298520

CPLM 2.82481984

CPLN 3.61228289

CPLP 7.23175837

CPLQ 3.41447359

CPLR 3.41215094

CPLS 4.04381374

CPLT 3.67371593

CPLV 3.11898257

CPLW 2.50496400

CPLY 2.71947269

CPMA 3.79687801

CPMC 3.21300437

CPMD 3.88239102

CPME 3.76276458

CPMF 2.69876655

CPMG 3.53519548

CPMH 3.44548745

CPMI 2.93476840

CPMK 3.67390524

CPML 2.88152851

CPMM 2.78047443

CPMN 3.57906607

CPMP 7.32052608

CPMQ 3.39556331

CPMR 3.43678037

CPMS 4.02494200

CPMT 3.75944975

CPMV 3.15039593

CPMW 2.80552926

CPMY 2.83637935

CPNA 3.35896384

CPNC 3.21206560

CPND 3.25261223

CPNE 3.25527569

CPNF 2.47786288

CPNG 3.10387308

CPNH 3.03344929

CPNI 2.71221443

CPNK 3.24109569

CPNL 2.64532462

CPNM 2.62448480

CPNN 3.16284610

CPNP 6.71964596

CPNQ 3.04546407

CPNR 2.96220134

CPNS 3.57813937

CPNT 3.17753764

CPNV 2.84255761

CPNW 2.44391589

CPNY 2.47117338

CPPA 3.05983097

CPPC 2.55572233

CPPD 3.03495475

CPPE 2.89636542

CPPF 2.04652530

CPPG 2.66303985

CPPH 2.66594338

CPPI 2.41965106

CPPK 2.90985479

CPPL 2.35676481

CPPM 2.41542030

CPPN 2.97955866

CPPP 5.96306976

CPPQ 2.76582255

CPPR 2.62102715

CPPS 3.26013108

CPPT 2.86820890

CPPV 2.56819143

CPPW 2.46309176

CPPY 2.11435762

CPQA 2.92120155

CPQC 2.56851484

CPQD 3.02230449

CPQE 2.88656535

CPQF 1.98620107

CPQG 2.85350648

CPQH 2.71294622

CPQI 2.27535892

CPQK 2.76522717

CPQL 2.20059239

CPQM 2.12304957

CPQN 2.81929721

CPQP 6.20676781

CPQQ 2.57004489

CPQR 2.56925821

CPQS 3.20372202

CPQT 2.96608833

CPQV 2.48341791

CPQW 1.99831561

CPQY 2.06382366

CPRA 3.00192381

CPRC 2.52241760

CPRD 3.06570674

CPRE 2.85684732

CPRF 2.08349838

CPRG 2.82651306

CPRH 2.65473301

CPRI 2.23036580

CPRK 2.87503996

CPRL 2.17480124

CPRM 2.07802956

CPRN 2.83286651

CPRP 6.39109081

CPRQ 2.65299865

CPRR 2.60244131

CPRS 3.36733106

CPRT 2.92159739

CPRV 2.46335445

CPRW 1.91297331

CPRY 2.04081317

CPSA 4.11402584

CPSC 3.53155561

CPSD 3.93848374

CPSE 3.88312370

CPSF 3.04254628

CPSG 3.82045863

CPSH 3.68657567

CPSI 3.41258149

CPSK 3.89549555

CPSL 3.27107224

CPSM 3.18152893

CPSN 3.83648824

CPSP 7.80829189

CPSQ 3.69963957

CPSR 3.62709107

CPSS 4.28745389

CPST 3.90219745

CPSV 3.54889219

CPSW 2.99602030

CPSY 3.12847587

CPTA 3.95530232

CPTC 3.52429921

CPTD 3.90782707

CPTE 3.79975052

CPTF 2.93049523

CPTG 3.68313480

CPTH 3.48794332

CPTI 3.19134656

CPTK 3.79911230

CPTL 3.10623598

CPTM 3.03233042

CPTN 3.67470446

CPTP 7.39787265

CPTQ 3.52528605

CPTR 3.58790490

CPTS 4.10553448

CPTT 3.72577364

CPTV 3.38038295

CPTW 2.77407627

CPTY 2.87998327

CPVA 4.27556087

CPVC 3.51434251

CPVD 4.33225024

CPVE 4.23885844

CPVF 3.03904807

CPVG 3.91824299

CPVH 3.82756348

CPVI 3.32648242

CPVK 4.18777279

CPVL 3.31362029

CPVM 3.29171063

CPVN 4.00075473

CPVP 7.86289907

CPVQ 3.93477507

CPVR 3.89901454

CPVS 4.46257330

CPVT 4.02868640

CPVV 3.54361695

CPVW 2.97797746

CPVY 3.10145830

CPWA 3.39670196

CPWC 2.57241752

CPWD 3.33661655

CPWE 3.26597476

CPWF 2.08397978

CPWG 2.93469245

CPWH 2.84602820

CPWI 2.52106221

CPWK 3.17977990

CPWL 2.36058138

CPWM 2.57261240

CPWN 3.18677949

CPWP 6.56097613

CPWQ 3.07049510

CPWR 2.90804012

CPWS 3.59221078

CPWT 3.24608679

CPWV 2.72548711

CPWW 2.10394739

CPWY 2.16483605

CPYA 3.75361958

CPYC 2.93547513

CPYD 3.71000268

CPYE 3.68863561

CPYF 2.51703582

CPYG 3.35458748

CPYH 3.29183156

CPYI 2.91852366

CPYK 3.73928072

CPYL 2.88897692

CPYM 2.80308703

CPYN 3.57738183

CPYP 7.19649733

CPYQ 3.47613477

CPYR 3.29767974

CPYS 4.00577514

CPYT 3.57966798

CPYV 3.10893232

CPYW 2.44612412

CPYY 2.52162840

CQAA 4.58748428

CQAC 4.21002194

CQAD 5.09938146

CQAE 5.57687184

CQAF 3.68082178

CQAG 4.37481069

CQAH 5.38065712

CQAI 3.91842265

CQAK 5.47945194

CQAL 4.14392005

CQAM 4.66625196

CQAN 5.20561125

CQAP 4.45857565

CQAQ 6.94472467

CQAR 5.40757976

CQAS 4.95264964

CQAT 4.74038899

CQAV 4.00685533

CQAW 3.81078342

CQAY 4.21539464

CQCA 8.18931033

CQCC 5.33045412

CQCD 8.72926243

CQCE 9.53874010

CQCF 7.05173059

CQCG 8.15295238

CQCH 8.64403633

CQCI 7.40486385

CQCK 9.41157002

CQCL 7.78703739

CQCM 8.34132314

CQCN 8.85349229

CQCP 8.27576528

CQCQ 13.32704890

CQCR 9.23483741

CQCS 8.68462172

CQCT 8.54827790

CQCV 7.65004156

CQCW 6.88554572

CQCY 7.74533962

CQDA 3.10442061

CQDC 2.83879434

CQDD 3.34353489

CQDE 3.78247486

CQDF 2.24894730

CQDG 2.77901706

CQDH 3.64583319

CQDI 2.53066564

CQDK 3.76279135

CQDL 2.64885154

CQDM 3.07673703

CQDN 3.47269161

CQDP 2.96290399

CQDQ 4.76125556

CQDR 3.65635578

CQDS 3.38535209

CQDT 3.26251845

CQDV 2.62484731

CQDW 2.42135462

CQDY 2.64857122

CQEA 3.10854474

CQEC 2.80933192

CQED 3.47238673

CQEE 3.79912362

CQEF 2.28905868

CQEG 2.92529438

CQEH 3.56150798

CQEI 2.54836968

CQEK 3.80833104

CQEL 2.65358870

CQEM 3.15010156

CQEN 3.55617048

CQEP 3.02264085

CQEQ 4.74065049

CQER 3.70520254

CQES 3.49805096

CQET 3.29889303

CQEV 2.60698406

CQEW 2.39095697

CQEY 2.63525304

CQFA 4.00107571

CQFC 3.56570809

CQFD 4.54296581

CQFE 5.10858427

CQFF 3.10017486

CQFG 3.60530859

CQFH 4.71546882

CQFI 3.38431849

CQFK 4.97719959

CQFL 3.51268325

CQFM 3.96579525

CQFN 4.60748881

CQFP 3.80988657

CQFQ 6.38339083

CQFR 4.75937866

CQFS 4.39711474

CQFT 4.21595849

CQFV 3.39497201

CQFW 3.31371928

CQFY 3.61697852

CQGA 3.56419544

CQGC 3.11283222

CQGD 3.86665324

CQGE 4.50341056

CQGF 2.75271359

CQGG 3.09178553

CQGH 4.27265793

CQGI 3.03434745

CQGK 4.44712224

CQGL 3.22340716

CQGM 3.71254496

CQGN 4.08864666

CQGP 3.33687458

CQGQ 5.92390917

CQGR 4.27453935

CQGS 3.88949086

CQGT 3.72832248

CQGV 3.05466913

CQGW 2.90986927

CQGY 3.20577530

CQHA 3.80807686

CQHC 3.00511319

CQHD 4.11478786

CQHE 4.62977816

CQHF 2.85011275

CQHG 3.42379334

CQHH 4.08020933

CQHI 3.12929471

CQHK 4.70401139

CQHL 3.35865689

CQHM 3.86875785

CQHN 4.22434182

CQHP 3.70303247

CQHQ 5.79711631

CQHR 4.47782334

CQHS 4.09688841

CQHT 3.94331769

CQHV 3.22939572

CQHW 3.03194479

CQHY 3.21128435

CQIA 4.18805141

CQIC 3.82101441

CQID 4.85923005

CQIE 5.32041250

CQIF 3.36195922

CQIG 3.95493519

CQIH 5.00949483

CQII 3.53583776

CQIK 5.22744085

CQIL 3.72400989

CQIM 4.19505366

CQIN 4.92865208

CQIP 4.13100892

CQIQ 6.45597026

CQIR 5.04876129

CQIS 4.68797661

CQIT 4.46576412

CQIV 3.61184868

CQIW 3.46675523

CQIY 3.91142763

CQKA 3.29138171

CQKC 3.09366727

CQKD 3.79557185

CQKE 4.17084361

CQKF 2.44942589

CQKG 3.08925508

CQKH 3.80137937

CQKI 2.76753955

CQKK 4.01495765

CQKL 2.88702872

CQKM 3.22042682

CQKN 3.82026403

CQKP 3.17883559

CQKQ 4.99482880

CQKR 3.92306879

CQKS 3.67115636

CQKT 3.54623447

CQKV 2.78052254

CQKW 2.60857726

CQKY 2.86485276

CQLA 4.15591182

CQLC 3.59417002

CQLD 4.70089314

CQLE 5.14186576

CQLF 3.13400693

CQLG 3.93268387

CQLH 4.89715035

CQLI 3.44406888

CQLK 5.06723345

CQLL 3.55519339

CQLM 4.06060725

CQLN 4.72111605

CQLP 4.03675071

CQLQ 6.22779330

CQLR 4.90266713

CQLS 4.55755123

CQLT 4.35487011

CQLV 3.46477851

CQLW 3.21547290

CQLY 3.70846440

CQMA 4.05918255

CQMC 3.63400666

CQMD 4.63837755

CQME 5.12426585

CQMF 3.15929720

CQMG 3.83220905

CQMH 4.78160083

CQMI 3.46268589

CQMK 4.93702787

CQML 3.59798667

CQMM 4.23004586

CQMN 4.67497298

CQMP 3.97481543

CQMQ 6.25150265

CQMR 4.88597954

CQMS 4.39703469

CQMT 4.28851179

CQMV 3.51481469

CQMW 3.61355467

CQMY 3.85777418

CQNA 3.60546839

CQNC 3.34927492

CQND 3.95538548

CQNE 4.46663983

CQNF 2.80771728

CQNG 3.39429125

CQNH 4.31358904

CQNI 3.17221085

CQNK 4.40157043

CQNL 3.24614795

CQNM 3.67935919

CQNN 4.14572727

CQNP 3.53288280

CQNQ 5.70593229

CQNR 4.29881143

CQNS 4.00836018

CQNT 3.78948271

CQNV 3.08602171

CQNW 2.86491817

CQNY 3.19383136

CQPA 3.16364308

CQPC 2.75433219

CQPD 3.48698393

CQPE 3.92690823

CQPF 2.30706291

CQPG 2.76211404

CQPH 3.67190816

CQPI 2.55114673

CQPK 3.84147223

CQPL 2.65993061

CQPM 3.09945699

CQPN 3.60219481

CQPP 3.08323979

CQPQ 5.16737247

CQPR 3.78264281

CQPS 3.47468249

CQPT 3.30608501

CQPV 2.63371306

CQPW 2.39866722

CQPY 2.66089078

CQQA 3.43666852

CQQC 3.14152022

CQQD 3.83793806

CQQE 4.27246384

CQQF 2.61073675

CQQG 3.29158228

CQQH 4.09442014

CQQI 2.85682588

CQQK 4.13178167

CQQL 2.98067577

CQQM 3.29407360

CQQN 3.83312412

CQQP 3.35973501

CQQQ 5.11516849

CQQR 4.06332396

CQQS 3.73928407

CQQT 3.62076296

CQQV 2.94141440

CQQW 2.83435539

CQQY 3.00420010

CQRA 3.58232592

CQRC 3.10691862

CQRD 3.87260018

CQRE 4.33096094

CQRF 2.50450066

CQRG 3.25808458

CQRH 4.00602632

CQRI 2.79272393

CQRK 4.28274542

CQRL 2.94140052

CQRM 3.36897130

CQRN 3.94574650

CQRP 3.39751571

CQRQ 5.48686407

CQRR 4.13550211

CQRS 3.90925812

CQRT 3.70386592

CQRV 2.99491511

CQRW 2.67101058

CQRY 2.91819375

CQSA 4.30315465

CQSC 3.97809298

CQSD 4.77171399

CQSE 5.26947984

CQSF 3.55255358

CQSG 4.11640343

CQSH 5.18419924

CQSI 3.84979400

CQSK 5.18431740

CQSL 3.99668804

CQSM 4.51372195

CQSN 4.92207019

CQSP 4.34855283

CQSQ 6.77472091

CQSR 5.12380301

CQSS 4.64617190

CQST 4.47468749

CQSV 3.86995304

CQSW 3.63383397

CQSY 3.99395944

CQTA 4.16381915

CQTC 3.85219209

CQTD 4.62522236

CQTE 5.05978088

CQTF 3.36726483

CQTG 4.00104359

CQTH 4.88356029

CQTI 3.61234884

CQTK 4.98036101

CQTL 3.77450535

CQTM 4.21113180

CQTN 4.67415594

CQTP 4.01515309

CQTQ 6.44878867

CQTR 4.91768243

CQTS 4.49914300

CQTT 4.30368199

CQTV 3.62805363

CQTW 3.46638058

CQTY 3.77803272

CQVA 4.49081449

CQVC 3.95703986

CQVD 5.06896210

CQVE 5.53368362

CQVF 3.47102147

CQVG 4.22243161

CQVH 5.25945408

CQVI 3.76169857

CQVK 5.44309705

CQVL 3.95757955

CQVM 4.50561668

CQVN 5.06959672

CQVP 4.37869911

CQVQ 6.83710528

CQVR 5.30146101

CQVS 4.88357559

CQVT 4.68436990

CQVV 3.79683314

CQVW 3.72090784

CQVY 4.01939834

CQWA 3.61288446

CQWC 2.99341980

CQWD 3.98001126

CQWE 4.45636411

CQWF 2.50498207

CQWG 3.28698199

CQWH 4.08645824

CQWI 2.94206450

CQWK 4.53366799

CQWL 3.13208091

CQWM 3.62164591

CQWN 4.00988022

CQWP 3.32172779

CQWQ 5.75684195

CQWR 4.34790143

CQWS 3.98624601

CQWT 3.73923886

CQWV 3.06402723

CQWW 2.81445629

CQWY 3.00087584

CQYA 3.99404962

CQYC 3.52640242

CQYD 4.44610103

CQYE 5.05223558

CQYF 3.02210237

CQYG 3.63314550

CQYH 4.75547818

CQYI 3.39685912

CQYK 4.98484100

CQYL 3.53879790

CQYM 3.93190856

CQYN 4.54587191

CQYP 3.95669336

CQYQ 6.26809348

CQYR 4.76598774

CQYS 4.37702439

CQYT 4.17469967

CQYV 3.45954528

CQYW 3.15663302

CQYY 3.50556688

CRAA 4.41994591

CRAC 4.20468455

CRAD 4.57079818

CRAE 4.96092123

CRAF 3.55836575

CRAG 4.26244701

CRAH 5.14789956

CRAI 3.87836658

CRAK 6.10898963

CRAL 4.00323212

CRAM 4.19605605

CRAN 4.87609246

CRAP 4.35275928

CRAQ 5.29224842

CRAR 7.19974195

CRAS 4.76158155

CRAT 4.64949185

CRAV 3.93539832

CRAW 4.09093214

CRAY 4.21661870

CRCA 7.95255536

CRCC 5.47239176

CRCD 7.96767041

CRCE 8.38928734

CRCF 6.90116153

CRCG 8.02803635

CRCH 8.37482533

CRCI 7.22843210

CRCK 10.30102657

CRCL 7.52497686

CRCM 7.77250662

CRCN 8.61320890

CRCP 7.96962980

CRCQ 9.23483741

CRCR 13.52936926

CRCS 8.45883331

CRCT 8.35224780

CRCV 7.42768993

CRCW 7.60355558

CRCY 7.67024425

CRDA 2.89320355

CRDC 2.74810536

CRDD 2.93918543

CRDE 3.20240624

CRDF 2.25330970

CRDG 2.66078562

CRDH 3.48875082

CRDI 2.44922205

CRDK 4.08009307

CRDL 2.53829874

CRDM 2.73719587

CRDN 3.26652541

CRDP 2.84361524

CRDQ 3.58439639

CRDR 4.98042161

CRDS 3.19281171

CRDT 3.17044692

CRDV 2.51708482

CRDW 2.47797895

CRDY 2.65611956

CREA 2.88426824

CREC 2.77381154

CRED 2.98642350

CREE 3.24882011

CREF 2.20914417

CREG 2.74531770

CREH 3.42019292

CREI 2.44218285

CREK 4.04527651

CREL 2.42738475

CREM 2.68121185

CREN 3.27149125

CREP 2.85569999

CREQ 3.55854982

CRER 4.82570836

CRES 3.23783050

CRET 3.11660620

CREV 2.55132509

CREW 2.57157002

CREY 2.67326169

CRFA 3.82691177

CRFC 3.48328673

CRFD 4.12074993

CRFE 4.46641235

CRFF 2.91933144

CRFG 3.58883065

CRFH 4.60739010

CRFI 3.31339276

CRFK 5.58323836

CRFL 3.38277296

CRFM 3.59926726

CRFN 4.40868826

CRFP 3.82354618

CRFQ 4.82703828

CRFR 6.49912822

CRFS 4.26432125

CRFT 4.15126472

CRFV 3.29381408

CRFW 3.30990776

CRFY 3.65727028

CRGA 3.35900820

CRGC 2.94350279

CRGD 3.39560800

CRGE 3.91029946

CRGF 2.68734739

CRGG 2.95458658

CRGH 4.01514986

CRGI 2.96544089

CRGK 4.97405282

CRGL 3.03189130

CRGM 3.27166963

CRGN 3.79688903

CRGP 3.20861568

CRGQ 4.29015212

CRGR 6.08353718

CRGS 3.68677526

CRGT 3.58303853

CRGV 2.98650719

CRGW 3.02094138

CRGY 3.25166324

CRHA 3.72466019

CRHC 3.18572623

CRHD 3.66100504

CRHE 4.03595090

CRHF 2.78561330

CRHG 3.38201396

CRHH 3.97905140

CRHI 3.15790466

CRHK 5.07948733

CRHL 3.22101750

CRHM 3.43104980

CRHN 3.96633055

CRHP 3.61757065

CRHQ 4.42568147

CRHR 5.92147079

CRHS 3.97248674

CRHT 3.83106510

CRHV 3.22763202

CRHW 3.31209351

CRHY 3.35853879

CRIA 3.99741893

CRIC 3.71837983

CRID 4.35534492

CRIE 4.75208731

CRIF 3.29103349

CRIG 3.86140804

CRIH 4.82152968

CRII 3.45223821

CRIK 5.77320840

CRIL 3.56917370

CRIM 3.80163968

CRIN 4.65770175

CRIP 4.08084176

CRIQ 5.06517891

CRIR 6.67474791

CRIS 4.48115160

CRIT 4.29196122

CRIV 3.44668689

CRIW 3.69176239

CRIY 4.01187032

CRKA 3.10098188

CRKC 3.10121560

CRKD 3.26635543

CRKE 3.57729244

CRKF 2.46680859

CRKG 2.96762240

CRKH 3.83694208

CRKI 2.81577831

CRKK 4.44219201

CRKL 2.81130201

CRKM 3.01916922

CRKN 3.59752138

CRKP 3.12093655

CRKQ 3.85351375

CRKR 5.27651843

CRKS 3.52288855

CRKT 3.36751944

CRKV 2.75850641

CRKW 2.80711221

CRKY 2.98309992

CRLA 4.02268609

CRLC 3.60897417

CRLD 4.25380077

CRLE 4.59280877

CRLF 3.11884448

CRLG 3.82406586

CRLH 4.65358523

CRLI 3.35428413

CRLK 5.59594125

CRLL 3.43908890

CRLM 3.70674757

CRLN 4.50920903

CRLP 3.97572345

CRLQ 4.90144635

CRLR 6.45080397

CRLS 4.38359142

CRLT 4.22343965

CRLV 3.40210070

CRLW 3.49247048

CRLY 3.69959964

CRMA 3.84915475

CRMC 3.71508403

CRMD 4.17025218

CRME 4.60538801

CRMF 3.12141173

CRMG 3.73192126

CRMH 4.52070417

CRMI 3.32779711

CRMK 5.46640519

CRML 3.40568769

CRMM 3.55585682

CRMN 4.39218550

CRMP 3.94897759

CRMQ 4.68523463

CRMR 6.48395514

CRMS 4.21151983

CRMT 4.09803068

CRMV 3.34435329

CRMW 3.67017900

CRMY 3.70102909

CRNA 3.35446105

CRNC 3.31375453

CRND 3.47825962

CRNE 3.81123991

CRNF 2.68199908

CRNG 3.18694633

CRNH 4.11569046

CRNI 3.03089579

CRNK 4.87864088

CRNL 3.07349969

CRNM 3.27500973

CRNN 3.81508750

CRNP 3.33201090

CRNQ 4.18313230

CRNR 5.92200830

CRNS 3.80124307

CRNT 3.60888767

CRNV 3.01999478

CRNW 3.00900534

CRNY 3.26711036

CRPA 2.92448070

CRPC 2.67191083

CRPD 3.01989872

CRPE 3.34101141

CRPF 2.24821002

CRPG 2.72601830

CRPH 3.42675730

CRPI 2.50294108

CRPK 4.27816692

CRPL 2.54898920

CRPM 2.76549686

CRPN 3.22272002

CRPP 2.80150962

CRPQ 3.74198059

CRPR 5.29042060

CRPS 3.22911747

CRPT 3.11788357

CRPV 2.54448652

CRPW 2.57928026

CRPY 2.66253369

CRQA 3.17351235

CRQC 3.07059449

CRQD 3.35373530

CRQE 3.55857001

CRQF 2.45031288

CRQG 3.08072629

CRQH 3.82757421

CRQI 2.69640201

CRQK 4.54269234

CRQL 2.76084869

CRQM 2.98769930

CRQN 3.54762548

CRQP 3.18189408

CRQQ 3.86932285

CRQR 5.38386726

CRQS 3.48042539

CRQT 3.37822586

CRQV 2.76778163

CRQW 2.86296534

CRQY 3.04395061

CRRA 3.50014599

CRRC 3.32817364

CRRD 3.50889271

CRRE 3.80895989

CRRF 2.55148914

CRRG 3.23305657

CRRH 3.92074530

CRRI 2.86553368

CRRK 4.72639287

CRRL 2.92668304

CRRM 3.21398131

CRRN 3.76966603

CRRP 3.37620043

CRRQ 4.13696239

CRRR 5.58171435

CRRS 3.74970395

CRRT 3.60528385

CRRV 2.94245345

CRRW 3.08428438

CRRY 3.03378700

CRSA 4.07316244

CRSC 4.18876326

CRSD 4.15945270

CRSE 4.50898657

CRSF 3.40196071

CRSG 3.94205360

CRSH 4.83056162

CRSI 3.69226872

CRSK 5.83267154

CRSL 3.80745236

CRSM 3.95736940

CRSN 4.55169058

CRSP 4.10061005

CRSQ 4.98232929

CRSR 6.98966145

CRSS 4.41457349

CRST 4.30644915

CRSV 3.72863798

CRSW 3.78049968

CRSY 3.97817527

CRTA 3.96859614

CRTC 3.84838056

CRTD 4.07401311

CRTE 4.41053312

CRTF 3.21808766

CRTG 3.80874461

CRTH 4.61981526

CRTI 3.49991491

CRTK 5.59664784

CRTL 3.60112909

CRTM 3.87050794

CRTN 4.39059676

CRTP 3.90896627

CRTQ 4.84284147

CRTR 6.68626829

CRTS 4.31454970

CRTT 4.16511754

CRTV 3.53948004

CRTW 3.61363502

CRTY 3.75434816

CRVA 4.31621012

CRVC 3.96395474

CRVD 4.56162301

CRVE 4.95189985

CRVF 3.44013041

CRVG 4.11440687

CRVH 5.04869853

CRVI 3.63620848

CRVK 6.04446124

CRVL 3.78545845

CRVM 4.05202370

CRVN 4.78684782

CRVP 4.32037342

CRVQ 5.29636256

CRVR 7.05625788

CRVS 4.70273217

CRVT 4.52644013

CRVV 3.70165987

CRVW 3.85892900

CRVY 4.10115800

CRWA 3.42181637

CRWC 2.92249408

CRWD 3.59030858

CRWE 3.93797941

CRWF 2.56160639

CRWG 3.18043235

CRWH 4.37810260

CRWI 3.05556334

CRWK 5.15990685

CRWL 3.09312758

CRWM 3.29975861

CRWN 3.94451402

CRWP 3.27994841

CRWQ 4.46352380

CRWR 5.95939057

CRWS 3.85172013

CRWT 3.73222490

CRWV 2.97480069

CRWW 3.12060022

CRWY 3.02948578

CRYA 3.79247404

CRYC 3.50056459

CRYD 3.97828515

CRYE 4.39672756

CRYF 2.88757648

CRYG 3.61565904

CRYH 4.52385236

CRYI 3.34190436

CRYK 5.56486089

CRYL 3.40467835

CRYM 3.64348921

CRYN 4.36886515

CRYP 3.72226890

CRYQ 4.80828942

CRYR 6.44667438

CRYS 4.21017425

CRYT 4.08965284

CRYV 3.36786100

CRYW 3.42625107

CRYY 3.52675936

CSAA 5.36657387

CSAC 5.43855832

CSAD 5.10531635

CSAE 4.82971231

CSAF 3.86681921

CSAG 5.18542296

CSAH 4.82387246

CSAI 4.01101762

CSAK 4.84093108

CSAL 3.90039283

CSAM 4.32424698

CSAN 5.37173173

CSAP 4.94906089

CSAQ 4.79134089

CSAR 4.67401348

CSAS 6.79063877

CSAT 5.71931402

CSAV 4.29643641

CSAW 3.75588296

CSAY 4.15419878

CSCA 9.50555642

CSCC 7.25877282

CSCD 9.00716274

CSCE 8.60807215

CSCF 7.46840631

CSCG 9.13293807

CSCH 8.04017804

CSCI 7.52806049

CSCK 8.76319119

CSCL 7.41861403

CSCM 7.85736547

CSCN 9.41615967

CSCP 8.92023868

CSCQ 8.68462172

CSCR 8.45883331

CSCS 12.76076465

CSCT 9.99743049

CSCV 7.96587204

CSCW 7.03378171

CSCY 7.79515304

CSDA 3.60337312

CSDC 3.74713533

CSDD 3.53387779

CSDE 3.26358649

CSDF 2.38200576

CSDG 3.36768007

CSDH 3.20569893

CSDI 2.60919454

CSDK 3.26215235

CSDL 2.41345705

CSDM 2.82845562

CSDN 3.65120908

CSDP 3.40593935

CSDQ 3.23005321

CSDR 3.08254507

CSDS 4.73181419

CSDT 4.00384017

CSDV 2.83804289

CSDW 2.32421007

CSDY 2.60075439

CSEA 3.44649534

CSEC 3.60951200

CSED 3.52876960

CSEE 3.26199522

CSEF 2.39736105

CSEG 3.37867169

CSEH 3.15488482

CSEI 2.49997217

CSEK 3.24340753

CSEL 2.35458357

CSEM 2.63952162

CSEN 3.65511117

CSEP 3.40918040

CSEQ 3.16061041

CSER 3.03212446

CSES 4.58239147

CSET 3.81912220

CSEV 2.68425953

CSEW 2.26793379

CSEY 2.61307055

CSFA 4.72710096

CSFC 4.62488824

CSFD 4.64404138

CSFE 4.39688335

CSFF 3.27060660

CSFG 4.44510272

CSFH 4.28781233

CSFI 3.50077498

CSFK 4.47847980

CSFL 3.37905197

CSFM 3.72164829

CSFN 4.90814722

CSFP 4.35085170

CSFQ 4.34914543

CSFR 4.24967654

CSFS 6.05314480

CSFT 5.07874143

CSFV 3.69412840

CSFW 3.24514389

CSFY 3.57167291

CSGA 4.41582831

CSGC 4.29283318

CSGD 4.05049514

CSGE 3.92500700

CSGF 3.11854102

CSGG 3.95282080

CSGH 3.81176511

CSGI 3.27823982

CSGK 4.01259413

CSGL 3.15936143

CSGM 3.53293826

CSGN 4.40194190

CSGP 3.87352052

CSGQ 3.99218465

CSGR 3.75028447

CSGS 5.69416968

CSGT 4.74938430

CSGV 3.46273348

CSGW 2.89678899

CSGY 3.30648099

CSHA 4.35184041

CSHC 4.15893021

CSHD 4.18601511

CSHE 4.03956620

CSHF 2.92449531

CSHG 4.06723718

CSHH 3.66229968

CSHI 3.16607232

CSHK 4.10492128

CSHL 3.06768907

CSHM 3.37133916

CSHN 4.49063358

CSHP 4.08696928

CSHQ 4.02810119

CSHR 3.84474955

CSHS 5.53879730

CSHT 4.69640505

CSHV 3.40874784

CSHW 2.89298007

CSHY 3.26135345

CSIA 4.95427342

CSIC 4.99264271

CSID 4.82742339

CSIE 4.66090070

CSIF 3.54795665

CSIG 4.80169740

CSIH 4.54422357

CSII 3.67698096

CSIK 4.65949716

CSIL 3.58181163

CSIM 3.96046832

CSIN 5.08889538

CSIP 4.69087466

CSIQ 4.60594915

CSIR 4.41907014

CSIS 6.26547292

CSIT 5.31094222

CSIV 3.87723918

CSIW 3.45707352

CSIY 3.85579129

CSKA 3.75488324

CSKC 3.91656742

CSKD 3.69000225

CSKE 3.44744091

CSKF 2.67340399

CSKG 3.62836226

CSKH 3.43163444

CSKI 2.70553681

CSKK 3.48640653

CSKL 2.60596787

CSKM 2.89551167

CSKN 3.94531082

CSKP 3.60330021

CSKQ 3.39592616

CSKR 3.31259962

CSKS 4.89963439

CSKT 4.09876874

CSKV 2.89349451

CSKW 2.47860132

CSKY 2.86842715

CSLA 4.80216819

CSLC 4.84477211

CSLD 4.69562536

CSLE 4.45877098

CSLF 3.35584238

CSLG 4.61506096

CSLH 4.36491875

CSLI 3.52880753

CSLK 4.47249322

CSLL 3.40633021

CSLM 3.78710041

CSLN 4.90245750

CSLP 4.48120261

CSLQ 4.40719005

CSLR 4.25131680

CSLS 6.04940533

CSLT 5.17511913

CSLV 3.71394341

CSLW 3.22351879

CSLY 3.61404900

CSMA 4.77379576

CSMC 4.89800660

CSMD 4.61668854

CSME 4.40071824

CSMF 3.42558895

CSMG 4.59370865

CSMH 4.27349338

CSMI 3.49364617

CSMK 4.35994203

CSML 3.45634782

CSMM 3.68544234

CSMN 4.84455732

CSMP 4.47328062

CSMQ 4.26612119

CSMR 4.22310378

CSMS 6.00445687

CSMT 5.10652341

CSMV 3.75139917

CSMW 3.39611589

CSMY 3.64964743

CSNA 4.33044212

CSNC 4.49410316

CSND 4.08236090

CSNE 3.87990530

CSNF 3.04000836

CSNG 4.08976099

CSNH 3.86650202

CSNI 3.20871750

CSNK 3.91126288

CSNL 3.07642583

CSNM 3.44085879

CSNN 4.38974210

CSNP 3.99155440

CSNQ 3.89093097

CSNR 3.71317760

CSNS 5.51552876

CSNT 4.58340379

CSNV 3.39771839

CSNW 2.91182000

CSNY 3.25531651

CSPA 3.81777258

CSPC 3.83775989

CSPD 3.60977406

CSPE 3.42146898

CSPF 2.47295277

CSPG 3.44561113

CSPH 3.39276578

CSPI 2.72768383

CSPK 3.45327891

CSPL 2.58108688

CSPM 2.96529326

CSPN 3.92759530

CSPP 3.47550558

CSPQ 3.39835736

CSPR 3.23445791

CSPS 5.06907210

CSPT 4.11502561

CSPV 2.92613532

CSPW 2.36180068

CSPY 2.74431848

CSQA 3.86220220

CSQC 3.99894424

CSQD 3.78976888

CSQE 3.54604706

CSQF 2.64000868

CSQG 3.76781226

CSQH 3.57473800

CSQI 2.81334147

CSQK 3.55039356

CSQL 2.66366335

CSQM 2.95464430

CSQN 3.97795236

CSQP 3.76509080

CSQQ 3.41432488

CSQR 3.41866421

CSQS 5.00069040

CSQT 4.20303801

CSQV 3.02848987

CSQW 2.64024911

CSQY 2.94676526

CSRA 3.97920593

CSRC 4.02106523

CSRD 3.83317113

CSRE 3.58315438

CSRF 2.68968186

CSRG 3.74013428

CSRH 3.56464166

CSRI 2.79301039

CSRK 3.62920752

CSRL 2.64853443

CSRM 2.98751295

CSRN 4.02228781

CSRP 3.75862459

CSRQ 3.54526950

CSRR 3.42435349

CSRS 5.29328788

CSRT 4.29729406

CSRV 3.03999650

CSRW 2.60243519

CSRY 2.90557476

CSSA 5.24563445

CSSC 5.41647758

CSSD 4.79835567

CSSE 4.57013825

CSSF 3.83600588

CSSG 4.92539196

CSSH 4.55532340

CSSI 3.94125902

CSSK 4.62600218

CSSL 3.79660786

CSSM 4.19578709

CSSN 5.16570773

CSSP 4.75360958

CSSQ 4.57516371

CSSR 4.48785918

CSSS 6.50384404

CSST 5.48435874

CSSV 4.17629483

CSSW 3.64149416

CSSY 4.03749441

CSTA 4.99231023

CSTC 5.15801221

CSTD 4.66576868

CSTE 4.41903859

CSTF 3.53001498

CSTG 4.74490304

CSTH 4.40657178

CSTI 3.70423812

CSTK 4.46284391

CSTL 3.59806592

CSTM 3.93729833

CSTN 4.95039190

CSTP 4.56501490

CSTQ 4.39637307

CSTR 4.30499577

CSTS 6.24994414

CSTT 5.22405140

CSTV 3.93956314

CSTW 3.41148012

CSTY 3.84738523

CSVA 5.26867506

CSVC 5.25978059

CSVD 5.10745028

CSVE 4.88401873

CSVF 3.71560396

CSVG 5.07037123

CSVH 4.78425343

CSVI 3.84512626

CSVK 4.86394135

CSVL 3.76162484

CSVM 4.17980472

CSVN 5.30816409

CSVP 4.92017719

CSVQ 4.83728942

CSVR 4.68707286

CSVS 6.64432516

CSVT 5.58612363

CSVV 4.06929467

CSVW 3.64356223

CSVY 4.02227347

CSWA 4.25661260

CSWC 3.92484441

CSWD 4.15470701

CSWE 3.85940975

CSWF 2.80837545

CSWG 3.89060193

CSWH 3.65531277

CSWI 3.03854835

CSWK 3.99031301

CSWL 2.85051179

CSWM 3.34007679

CSWN 4.39481647

CSWP 3.71881597

CSWQ 3.86544022

CSWR 3.69436354

CSWS 5.58778124

CSWT 4.56103897

CSWV 3.22991709

CSWW 2.78894962

CSWY 2.97960615

CSYA 4.64661485

CSYC 4.57740864

CSYD 4.53317650

CSYE 4.32217319

CSYF 3.22560652

CSYG 4.40143427

CSYH 4.26092981

CSYI 3.38342528

CSYK 4.40405945

CSYL 3.29306593

CSYM 3.66366082

CSYN 4.83705158

CSYP 4.34356973

CSYQ 4.30462859

CSYR 4.13225832

CSYS 5.99912094

CSYT 5.03518837

CSYV 3.61336231

CSYW 3.17294653

CSYY 3.53010089

CTAA 5.04300688

CTAC 5.41421919

CTAD 4.70193748

CTAE 4.70105128

CTAF 4.23184749

CTAG 4.45207752

CTAH 4.70340302

CTAI 4.88259046

CTAK 4.76223475

CTAL 4.54772471

CTAM 4.77000211

CTAN 5.06715969

CTAP 4.64095086

CTAQ 4.70723253

CTAR 4.63592429

CTAS 5.85247169

CTAT 7.05356414

CTAV 5.17566158

CTAW 3.97055695

CTAY 4.26046889

CTCA 8.88758649

CTCC 6.68617296

CTCD 8.52235196

CTCE 8.44208758

CTCF 7.49447292

CTCG 8.16186800

CTCH 7.71687971

CTCI 8.48741281

CTCK 8.72666887

CTCL 7.96705748

CTCM 8.38885394

CTCN 9.00714417

CTCP 8.42010892

CTCQ 8.54827790

CTCR 8.35224780

CTCS 9.99743049

CTCT 12.82558582

CTCV 8.86274063

CTCW 6.80221032

CTCY 7.73868559

CTDA 3.26570759

CTDC 3.57259489

CTDD 3.13100862

CTDE 3.10939644

CTDF 2.65885823

CTDG 2.76127750

CTDH 3.02250780

CTDI 3.16944185

CTDK 3.20435549

CTDL 2.88527965

CTDM 3.19776014

CTDN 3.37480791

CTDP 3.12805469

CTDQ 3.12859511

CTDR 3.06626671

CTDS 4.04814321

CTDT 4.95880990

CTDV 3.43272224

CTDW 2.58030399

CTDY 2.68176159

CTEA 3.17207454

CTEC 3.44291484

CTED 3.12556881

CTEE 3.06145072

CTEF 2.65345496

CTEG 2.73430127

CTEH 3.04376976

CTEI 3.16104991

CTEK 3.12283038

CTEL 2.80634180

CTEM 3.00058509

CTEN 3.34159055

CTEP 3.04530536

CTEQ 3.04866430

CTER 2.99067444

CTES 3.85752216

CTET 4.70384909

CTEV 3.36231035

CTEW 2.34714994

CTEY 2.67390817

CTFA 4.47124717

CTFC 4.58330161

CTFD 4.29974275

CTFE 4.29171102

CTFF 3.59130959

CTFG 3.85792213

CTFH 4.17684877

CTFI 4.29966194

CTFK 4.40630409

CTFL 3.92803627

CTFM 4.18306111

CTFN 4.56759683

CTFP 4.06991759

CTFQ 4.33934528

CTFR 4.16928960

CTFS 5.20338407

CTFT 6.22037662

CTFV 4.44847317

CTFW 3.25731222

CTFY 3.66933746

CTGA 4.17524625

CTGC 4.18487352

CTGD 3.67370592

CTGE 3.82098962

CTGF 3.44043699

CTGG 3.34116251

CTGH 3.73082403

CTGI 4.01976056

CTGK 3.90030063

CTGL 3.70906752

CTGM 3.97806599

CTGN 4.07546783

CTGP 3.60928199

CTGQ 3.92798155

CTGR 3.69016394

CTGS 4.82727179

CTGT 5.87836630

CTGV 4.18289046

CTGW 3.06881863

CTGY 3.47768601

CTHA 4.02838531

CTHC 3.79529621

CTHD 3.87540872

CTHE 3.90112827

CTHF 3.20755627

CTHG 3.45165519

CTHH 3.56145905

CTHI 3.81157792

CTHK 3.99844511

CTHL 3.52641215

CTHM 3.76829560

CTHN 4.09317790

CTHP 3.68215183

CTHQ 3.92162502

CTHR 3.79658639

CTHS 4.75288368

CTHT 5.68566299

CTHV 4.10807614

CTHW 2.99707089

CTHY 3.35698269

CTIA 4.69276010

CTIC 4.88869316

CTID 4.51892998

CTIE 4.53548674

CTIF 3.92604109

CTIG 4.10458552

CTIH 4.38527998

CTII 4.43856732

CTIK 4.57610460

CTIL 4.16736731

CTIM 4.38257218

CTIN 4.79799464

CTIP 4.36650746

CTIQ 4.57516191

CTIR 4.36403535

CTIS 5.45395109

CTIT 6.43056569

CTIV 4.64998957

CTIW 3.68402109

CTIY 4.05941778

CTKA 3.38804680

CTKC 3.69622329

CTKD 3.34227463

CTKE 3.29946844

CTKF 2.79564215

CTKG 2.95220078

CTKH 3.27269085

CTKI 3.39943953

CTKK 3.39946671

CTKL 3.03294634

CTKM 3.25469907

CTKN 3.65023054

CTKP 3.24705518

CTKQ 3.26274567

CTKR 3.28858561

CTKS 4.11243147

CTKT 5.02738453

CTKV 3.54004790

CTKW 2.60345070

CTKY 2.89625208

CTLA 4.51870721

CTLC 4.66735586

CTLD 4.35623657

CTLE 4.29795982

CTLF 3.68077517

CTLG 3.97385303

CTLH 4.18409204

CTLI 4.27369381

CTLK 4.37225894

CTLL 3.94559617

CTLM 4.22822621

CTLN 4.62929834

CTLP 4.17915068

CTLQ 4.28677073

CTLR 4.17175563

CTLS 5.24459512

CTLT 6.22795507

CTLV 4.48718904

CTLW 3.35859730

CTLY 3.74934476

CTMA 4.47069027

CTMC 4.73906301

CTMD 4.26669666

CTME 4.25499834

CTMF 3.70214697

CTMG 3.95671777

CTMH 4.12112244

CTMI 4.28025480

CTMK 4.25877478

CTML 3.96135503

CTMM 4.16017654

CTMN 4.56415022

CTMP 4.19752337

CTMQ 4.18779607

CTMR 4.16660746

CTMS 5.20769890

CTMT 6.23659879

CTMV 4.54676349

CTMW 3.45057594

CTMY 3.76372234

CTNA 3.94910611

CTNC 4.18315648

CTND 3.76361653

CTNE 3.72313609

CTNF 3.36244477

CTNG 3.43821892

CTNH 3.63074284

CTNI 3.86787626

CTNK 3.80286823

CTNL 3.57662442

CTNM 3.79006211

CTNN 4.04201449

CTNP 3.61783158

CTNQ 3.74458742

CTNR 3.59241721

CTNS 4.62463448

CTNT 5.65805083

CTNV 4.07762001

CTNW 2.92690581

CTNY 3.25829148

CTPA 3.51494186

CTPC 3.61322796

CTPD 3.30313542

CTPE 3.28579517

CTPF 2.75289342

CTPG 2.87163005

CTPH 3.16343287

CTPI 3.40331664

CTPK 3.35829838

CTPL 3.10021519

CTPM 3.31868437

CTPN 3.63538518

CTPP 3.08915150

CTPQ 3.30395402

CTPR 3.19878570

CTPS 4.22228883

CTPT 5.18281599

CTPV 3.60001734

CTPW 2.55376426

CTPY 2.82144225

CTQA 3.53333361

CTQC 3.81446556

CTQD 3.41561941

CTQE 3.34923529

CTQF 2.89610259

CTQG 3.11410398

CTQH 3.38503215

CTQI 3.42090775

CTQK 3.43762016

CTQL 3.13521696

CTQM 3.33103244

CTQN 3.62636370

CTQP 3.39525043

CTQQ 3.30784871

CTQR 3.34839778

CTQS 4.22130642

CTQT 5.10756084

CTQV 3.69140078

CTQW 2.75883950

CTQY 3.01338137

CTRA 3.67203241

CTRC 3.80901031

CTRD 3.53287169

CTRE 3.45329529

CTRF 2.84956045

CTRG 3.14631281

CTRH 3.39331435

CTRI 3.44258566

CTRK 3.50545336

CTRL 3.11462066

CTRM 3.39335398

CTRN 3.71057394

CTRP 3.37404657

CTRQ 3.39954961

CTRR 3.35182465

CTRS 4.35092295

CTRT 5.32456928

CTRV 3.72742972

CTRW 2.68860410

CTRY 2.92350893

CTSA 4.83559875

CTSC 5.14863842

CTSD 4.41174471

CTSE 4.39210882

CTSF 4.04629610

CTSG 4.20194997

CTSH 4.38820588

CTSI 4.70324386

CTSK 4.52075961

CTSL 4.32220679

CTSM 4.56526280

CTSN 4.80131271

CTSP 4.40304592

CTSQ 4.42610163

CTSR 4.36631556

CTSS 5.48769456

CTST 6.71160518

CTSV 4.92361581

CTSW 3.76432154

CTSY 4.06782117

CTTA 4.68416706

CTTC 4.97412226

CTTD 4.29816770

CTTE 4.27383003

CTTF 3.82996318

CTTG 4.05816547

CTTH 4.26514596

CTTI 4.48938579

CTTK 4.39143588

CTTL 4.17304747

CTTM 4.38305347

CTTN 4.64362750

CTTP 4.23614631

CTTQ 4.40364643

CTTR 4.27442821

CTTS 5.35471199

CTTT 6.43791708

CTTV 4.71493978

CTTW 3.58350976

CTTY 3.91325673

CTVA 5.02462565

CTVC 5.20775221

CTVD 4.78243402

CTVE 4.79920465

CTVF 4.08446580

CTVG 4.36283753

CTVH 4.70759447

CTVI 4.69182924

CTVK 4.87572806

CTVL 4.41226573

CTVM 4.65718850

CTVN 5.02763339

CTVP 4.62814537

CTVQ 4.81677923

CTVR 4.69864318

CTVS 5.77468356

CTVT 6.88770893

CTVV 4.93212170

CTVW 3.89965614

CTVY 4.21009810

CTWA 3.96215404

CTWC 3.66636515

CTWD 3.72274493

CTWE 3.76083878

CTWF 3.02007524

CTWG 3.33934092

CTWH 3.58947859

CTWI 3.75761424

CTWK 3.88686453

CTWL 3.50515508

CTWM 4.26602210

CTWN 3.95757172

CTWP 3.57636050

CTWQ 3.85304530

CTWR 3.61440825

CTWS 4.69377159

CTWT 5.53883172

CTWV 3.97143783

CTWW 2.76493561

CTWY 3.13948475

CTYA 4.35956643

CTYC 4.37407093

CTYD 4.17950452

CTYE 4.20199917

CTYF 3.45132678

CTYG 3.77456760

CTYH 4.01138368

CTYI 4.18383971

CTYK 4.30684485

CTYL 3.78905217

CTYM 4.10957929

CTYN 4.48546291

CTYP 4.01868099

CTYQ 4.24730590

CTYR 4.04162075

CTYS 5.08920490

CTYT 6.17373073

CTYV 4.37244142

CTYW 3.10711234

CTYY 3.57760818

CVAA 5.18225893

CVAC 5.77614112

CVAD 3.28414215

CVAE 3.78916237

CVAF 5.24423717

CVAG 3.63773420

CVAH 4.06552475

CVAI 6.80420938

CVAK 3.87146897

CVAL 5.88586525

CVAM 5.48987859

CVAN 3.70559572

CVAP 4.20131967

CVAQ 3.88153093

CVAR 3.89330191

CVAS 4.33593259

CVAT 5.12184410

CVAV 7.20487232

CVAW 4.43345137

CVAY 4.60676204

CVCA 9.03492451

CVCC 6.37117058

CVCD 6.93744087

CVCE 7.46673205

CVCF 8.52352721

CVCG 6.96570231

CVCH 6.91065175

CVCI 10.76764137

CVCK 7.64867781

CVCL 9.49033890

CVCM 9.07331560

CVCN 7.37032118

CVCP 7.68799847

CVCQ 7.65004156

CVCR 7.42768993

CVCS 7.96587204

CVCT 8.86274063

CVCV 12.83432596

CVCW 7.20627368

CVCY 8.01559496

CVDA 3.13127677

CVDC 3.69343369

CVDD 1.74118855

CVDE 2.10490736

CVDF 3.30568165

CVDG 1.79166960

CVDH 2.39724238

CVDI 4.56906775

CVDK 2.16122400

CVDL 3.78956825

CVDM 3.53457223

CVDN 2.10167082

CVDP 2.54297359

CVDQ 2.25031764

CVDR 2.18930810

CVDS 2.54484601

CVDT 3.16697076

CVDV 4.93528314

CVDW 2.62266872

CVDY 2.89921304

CVEA 3.12074257

CVEC 3.74904464

CVED 1.82452573

CVEE 2.11908222

CVEF 3.32587008

CVEG 1.84378020

CVEH 2.49218321

CVEI 4.57770721

CVEK 2.15720233

CVEL 3.84114655

CVEM 3.49281731

CVEN 2.07099565

CVEP 2.45865241

CVEQ 2.19466910

CVER 2.18273764

CVES 2.51363547

CVET 3.13645886

CVEV 4.90013944

CVEW 2.73807790

CVEY 2.92967654

CVFA 4.64404158

CVFC 4.87720511

CVFD 2.90248434

CVFE 3.34907792

CVFF 4.58937423

CVFG 3.26599666

CVFH 3.51588165

CVFI 5.99076509

CVFK 3.49424552

CVFL 5.18808556

CVFM 4.87348202

CVFN 3.29054469

CVFP 3.60787239

CVFQ 3.57005496

CVFR 3.44849206

CVFS 3.85787729

CVFT 4.53269216

CVFV 6.30935343

CVFW 3.65378545

CVFY 4.04097226

CVGA 4.20667989

CVGC 4.28154467

CVGD 2.29891311

CVGE 2.88140299

CVGF 4.35021258

CVGG 2.47954844

CVGH 3.05478477

CVGI 5.68128737

CVGK 2.93326667

CVGL 4.91707548

CVGM 4.58475693

CVGN 2.78870897

CVGP 3.06373694

CVGQ 3.09412950

CVGR 2.92944419

CVGS 3.36018647

CVGT 4.05950187

CVGV 5.99076168

CVGW 3.44133197

CVGY 3.68371675

CVHA 4.01045660

CVHC 3.92541945

CVHD 2.51178110

CVHE 2.96330149

CVHF 4.09706952

CVHG 2.70193176

CVHH 2.87046944

CVHI 5.41807165

CVHK 2.99088462

CVHL 4.68151810

CVHM 4.29128618

CVHN 2.78909223

CVHP 3.15891939

CVHQ 3.01698285

CVHR 2.98376768

CVHS 3.36135189

CVHT 4.00746374

CVHV 5.75957705

CVHW 3.33828010

CVHY 3.61653469

CVIA 4.93408369

CVIC 5.37580962

CVID 3.14425486

CVIE 3.64319223

CVIF 4.93743947

CVIG 3.45861002

CVIH 3.81389380

CVII 6.25887171

CVIK 3.75537186

CVIL 5.50828563

CVIM 5.12560638

CVIN 3.57196178

CVIP 3.88241810

CVIQ 3.78836736

CVIR 3.68794567

CVIS 4.13206584

CVIT 4.75103228

CVIV 6.58263394

CVIW 4.10198258

CVIY 4.41605704

CVKA 3.31235577

CVKC 3.93905260

CVKD 1.91807157

CVKE 2.33557418

CVKF 3.61728329

CVKG 2.04473511

CVKH 2.52477418

CVKI 4.93442561

CVKK 2.38654901

CVKL 4.06320139

CVKM 3.70854349

CVKN 2.24669682

CVKP 2.60897711

CVKQ 2.37454405

CVKR 2.31615335

CVKS 2.69499519

CVKT 3.28689931

CVKV 5.22499342

CVKW 2.72226749

CVKY 3.05182742

CVLA 4.69602784

CVLC 5.11222096

CVLD 2.97241368

CVLE 3.38483732

CVLF 4.73381159

CVLG 3.33159510

CVLH 3.64329315

CVLI 6.03894895

CVLK 3.46718141

CVLL 5.27529777

CVLM 4.92509438

CVLN 3.29361979

CVLP 3.68332593

CVLQ 3.51620670

CVLR 3.48266125

CVLS 3.94264179

CVLT 4.58303485

CVLV 6.37001701

CVLW 3.83201185

CVLY 4.15357941

CVMA 4.62951398

CVMC 5.11542868

CVMD 2.88609866

CVME 3.38632951

CVMF 4.72404760

CVMG 3.27615636

CVMH 3.54055683

CVMI 5.97217183

CVMK 3.37258288

CVML 5.21933895

CVMM 4.78320364

CVMN 3.25740960

CVMP 3.68320260

CVMQ 3.39109702

CVMR 3.49726952

CVMS 3.80707207

CVMT 4.52051821

CVMV 6.35087692

CVMW 3.89503912

CVMY 4.12089073

CVNA 3.85968097

CVNC 4.30713498

CVND 2.31043022

CVNE 2.67321889

CVNF 4.01350485

CVNG 2.39332051

CVNH 2.91560120

CVNI 5.35081359

CVNK 2.80899186

CVNL 4.53492489

CVNM 4.22964306

CVNN 2.67295298

CVNP 3.04785738

CVNQ 2.89897877

CVNR 2.75569127

CVNS 3.14001990

CVNT 3.80490045

CVNV 5.70182115

CVNW 3.34634019

CVNY 3.48338583

CVPA 3.39802421

CVPC 3.62654416

CVPD 1.92477902

CVPE 2.26926621

CVPF 3.50837701

CVPG 1.95908267

CVPH 2.46883201

CVPI 4.86983925

CVPK 2.42980946

CVPL 4.10403947

CVPM 3.74437774

CVPN 2.26787412

CVPP 2.55532394

CVPQ 2.46970117

CVPR 2.37347605

CVPS 2.71509641

CVPT 3.32471640

CVPV 5.16517576

CVPW 2.73873339

CVPY 2.93586193

CVQA 3.45203988

CVQC 4.02348016

CVQD 2.03395786

CVQE 2.35158434

CVQF 3.65158618

CVQG 2.19143239

CVQH 2.69869891

CVQI 4.93497385

CVQK 2.41526432

CVQL 4.16741973

CVQM 3.79870301

CVQN 2.32703708

CVQP 2.76208546

CVQQ 2.41732764

CVQR 2.46793038

CVQS 2.79609233

CVQT 3.44818084

CVQV 5.26880279

CVQW 3.08131216

CVQY 3.21316270

CVRA 3.59249709

CVRC 3.99821643

CVRD 2.11782398

CVRE 2.53326795

CVRF 3.71174772

CVRG 2.17752742

CVRH 2.73612745

CVRI 5.08314872

CVRK 2.49470676

CVRL 4.28497751

CVRM 3.87736037

CVRN 2.35677919

CVRP 2.76247875

CVRQ 2.54495596

CVRR 2.47345267

CVRS 2.88333854

CVRT 3.50209360

CVRV 5.50212115

CVRW 2.97346246

CVRY 3.18372931

CVSA 4.78900892

CVSC 5.24721140

CVSD 2.96456651

CVSE 3.43397444

CVSF 4.87234992

CVSG 3.23557990

CVSH 3.66562126

CVSI 6.40198910

CVSK 3.53645640

CVSL 5.51213364

CVSM 5.15126317

CVSN 3.40929005

CVSP 3.82847905

CVSQ 3.58269855

CVSR 3.53448163

CVSS 3.93414038

CVST 4.68656599

CVSV 6.77233552

CVSW 3.99641976

CVSY 4.28398492

CVTA 4.68107576

CVTC 5.19225826

CVTD 2.85510561

CVTE 3.33351568

CVTF 4.74128906

CVTG 3.14711700

CVTH 3.59637109

CVTI 6.18820566

CVTK 3.42044223

CVTL 5.37943107

CVTM 4.98628503

CVTN 3.28415204

CVTP 3.70438000

CVTQ 3.48193074

CVTR 3.47896934

CVTS 3.81570130

CVTT 4.53566142

CVTV 6.55770181

CVTW 3.92285624

CVTY 4.15189292

CVVA 5.25184878

CVVC 5.70865608

CVVD 3.37921219

CVVE 3.91224195

CVVF 5.12139665

CVVG 3.62893792

CVVH 4.08476476

CVVI 6.63462955

CVVK 3.93516631

CVVL 5.80705504

CVVM 5.46553281

CVVN 3.71451747

CVVP 4.10618245

CVVQ 4.03589914

CVVR 3.99362292

CVVS 4.31557078

CVVT 5.04884556

CVVV 7.04492099

CVVW 4.41192500

CVVY 4.58073246

CVWA 4.03948245

CVWC 4.00091063

CVWD 2.52479534

CVWE 2.87889244

CVWF 3.91686315

CVWG 2.67388641

CVWH 3.03950129

CVWI 5.41031113

CVWK 3.00128420

CVWL 4.59069305

CVWM 4.39411671

CVWN 2.81790177

CVWP 3.03141880

CVWQ 3.16000495

CVWR 2.94104853

CVWS 3.30046253

CVWT 3.93349820

CVWV 5.69064717

CVWW 3.09948110

CVWY 3.33029476

CVYA 4.48766104

CVYC 4.67247583

CVYD 2.80960884

CVYE 3.29950546

CVYF 4.44802928

CVYG 2.95953674

CVYH 3.37821870

CVYI 5.87076709

CVYK 3.43610105

CVYL 5.04122578

CVYM 4.78957559

CVYN 3.12581683

CVYP 3.47693462

CVYQ 3.41722069

CVYR 3.28287150

CVYS 3.72599611

CVYT 4.30660776

CVYV 6.27782194

CVYW 3.38025728

CVYY 3.85214636

CWAA 3.39221233

CWAC 4.13090621

CWAD 2.86480844

CWAE 2.99899883

CWAF 5.71292909

CWAG 3.06802507

CWAH 4.30947973

CWAI 4.14747946

CWAK 3.15123195

CWAL 4.31954653

CWAM 4.30419105

CWAN 3.43381920

CWAP 3.18914666

CWAQ 3.39148988

CWAR 3.55113607

CWAS 3.30822124

CWAT 3.38545107

CWAV 3.89457273

CWAW 9.17098860

CWAY 5.88379902

CWCA 6.78857920

CWCC 5.62152722

CWCD 6.10015421

CWCE 6.36427551

CWCF 9.92833604

CWCG 6.56601743

CWCH 7.43777759

CWCI 7.26348834

CWCK 7.04655277

CWCL 7.88717649

CWCM 7.66257439

CWCN 6.85012095

CWCP 6.40483719

CWCQ 6.88554572

CWCR 7.60355558

CWCS 7.03378171

CWCT 6.80221032

CWCV 7.20627368

CWCW 16.10514842

CWCY 10.12575185

CWDA 1.82707877

CWDC 2.65612242

CWDD 1.53081257

CWDE 1.53894774

CWDF 3.94401962

CWDG 1.51989308

CWDH 3.17991364

CWDI 2.48993631

CWDK 1.71190930

CWDL 2.58547854

CWDM 2.56235600

CWDN 1.75950497

CWDP 1.73514418

CWDQ 2.07672152

CWDR 2.03883787

CWDS 1.84680474

CWDT 1.85477997

CWDV 2.23245680

CWDW 7.06709924

CWDY 3.91961727

CWEA 1.71615422

CWEC 2.63291899

CWED 1.55274921

CWEE 1.37698576

CWEF 3.86266749

CWEG 1.43450016

CWEH 2.39033169

CWEI 2.53426748

CWEK 1.43170784

CWEL 2.58194809

CWEM 2.62278147

CWEN 1.55636261

CWEP 1.66750174

CWEQ 1.55092381

CWER 1.87948289

CWES 1.72584381

CWET 1.74438811

CWEV 2.22551483

CWEW 6.96673037

CWEY 3.89493922

CWFA 3.27621838

CWFC 3.80706245

CWFD 2.52379262

CWFE 2.72182075

CWFF 5.11828890

CWFG 2.79258628

CWFH 3.80800602

CWFI 3.96896682

CWFK 2.90931111

CWFL 3.97770959

CWFM 4.06296602

CWFN 2.92816562

CWFP 3.09769113

CWFQ 3.13907984

CWFR 3.47425947

CWFS 3.14869546

CWFT 3.19678530

CWFV 3.63227385

CWFW 8.25744210

CWFY 5.23460677

CWGA 2.74863038

CWGC 3.10746483

CWGD 1.93967375

CWGE 2.57009811

CWGF 4.81468192

CWGG 2.11325432

CWGH 3.43102292

CWGI 3.49756703

CWGK 2.49156515

CWGL 3.64059196

CWGM 3.67826488

CWGN 2.56755852

CWGP 2.30121327

CWGQ 2.74553739

CWGR 2.72025221

CWGS 2.60728326

CWGT 2.78067059

CWGV 3.14948277

CWGW 7.98391943

CWGY 4.97584112

CWHA 2.64516819

CWHC 2.89283059

CWHD 2.13308938

CWHE 2.23209335

CWHF 4.71511008

CWHG 2.08911832

CWHH 3.18365542

CWHI 3.25802022

CWHK 2.37671590

CWHL 3.41302417

CWHM 3.43454716

CWHN 2.36203748

CWHP 2.56521478

CWHQ 2.51490871

CWHR 2.75981969

CWHS 2.50461287

CWHT 2.57469534

CWHV 3.02400372

CWHW 7.79880780

CWHY 4.61357493

CWIA 3.24936814

CWIC 4.13936617

CWID 2.66191135

CWIE 2.84385552

CWIF 5.46772358

CWIG 2.83515806

CWIH 4.14559987

CWII 3.97877032

CWIK 3.05391107

CWIL 4.16034795

CWIM 4.01864073

CWIN 3.10177171

CWIP 2.95459599

CWIQ 3.14246653

CWIR 3.46699341

CWIS 3.20346084

CWIT 3.21246922

CWIV 3.56882875

CWIW 8.56879176

CWIY 5.59718620

CWKA 1.88733299

CWKC 2.77975081

CWKD 1.37950851

CWKE 1.65571293

CWKF 4.19628076

CWKG 1.63910285

CWKH 2.75049732

CWKI 2.67488576

CWKK 1.75862868

CWKL 2.69425621

CWKM 2.68187947

CWKN 1.76546693

CWKP 1.89662800

CWKQ 1.81519094

CWKR 2.19935149

CWKS 1.91732774

CWKT 1.90469880

CWKV 2.39522145

CWKW 7.39716147

CWKY 4.09652457

CWLA 3.22625555

CWLC 3.84445750

CWLD 2.53071216

CWLE 2.72762977

CWLF 5.22983253

CWLG 2.62124557

CWLH 3.95197774

CWLI 3.75987994

CWLK 2.87347679

CWLL 4.06439917

CWLM 3.84596294

CWLN 2.94045494

CWLP 2.79735620

CWLQ 2.96830578

CWLR 3.28423088

CWLS 3.03955693

CWLT 3.12229328

CWLV 3.53788517

CWLW 8.33724565

CWLY 5.43847635

CWMA 3.10559770

CWMC 3.93676156

CWMD 2.53903401

CWME 2.76405575

CWMF 5.19511324

CWMG 2.75645233

CWMH 3.87285163

CWMI 3.71297338

CWMK 2.87300044

CWML 3.94026994

CWMM 3.83765535

CWMN 2.86225067

CWMP 2.91500025

CWMQ 3.19732301

CWMR 3.25246005

CWMS 2.95033305

CWMT 3.09987830

CWMV 3.49564150

CWMW 8.37959352

CWMY 5.29942719

CWNA 2.40829690

CWNC 3.29839287

CWND 1.88890658

CWNE 1.95518614

CWNF 4.54691216

CWNG 2.17158467

CWNH 4.08582923

CWNI 3.04056293

CWNK 2.09664275

CWNL 3.26314837

CWNM 3.24514850

CWNN 2.32717588

CWNP 2.03357708

CWNQ 2.27172160

CWNR 2.45534560

CWNS 2.40881176

CWNT 2.40219306

CWNV 2.75134669

CWNW 7.89729382

CWNY 4.67555641

CWPA 2.20808599

CWPC 2.79405269

CWPD 1.62746741

CWPE 1.85309979

CWPF 4.25706460

CWPG 1.64983145

CWPH 2.97466307

CWPI 2.70189854

CWPK 2.11621276

CWPL 2.92379650

CWPM 2.98717440

CWPN 2.08182748

CWPP 1.92365148

CWPQ 2.17407791

CWPR 2.21092860

CWPS 2.12139179

CWPT 2.08946575

CWPV 2.47836174

CWPW 7.22498408

CWPY 4.13357069

CWQA 2.08152940

CWQC 3.02923762

CWQD 1.76218134

CWQE 1.66477033

CWQF 4.22780657

CWQG 1.76990875

CWQH 2.89967539

CWQI 2.71900819

CWQK 1.82155970

CWQL 2.89264072

CWQM 2.87922824

CWQN 1.89771929

CWQP 1.92552432

CWQQ 1.96622742

CWQR 2.51808196

CWQS 1.91550657

CWQT 2.08894148

CWQV 2.52535106

CWQW 7.40155290

CWQY 4.19357321

CWRA 2.18388636

CWRC 3.71084306

CWRD 1.66916970

CWRE 1.93110175

CWRF 4.27674085

CWRG 1.80265741

CWRH 2.99041975

CWRI 2.87788740

CWRK 2.05943151

CWRL 2.96739731

CWRM 2.92108568

CWRN 1.94364682

CWRP 2.25548577

CWRQ 2.11024087

CWRR 2.44338204

CWRS 2.24781375

CWRT 2.24199889

CWRV 2.65233490

CWRW 7.59215099

CWRY 4.36621160

CWSA 3.07539484

CWSC 3.98711669

CWSD 2.37086190

CWSE 2.60198209

CWSF 5.71456450

CWSG 2.63014599

CWSH 3.85779184

CWSI 3.79285444

CWSK 2.78355319

CWSL 3.97186829

CWSM 3.91601253

CWSN 2.94817108

CWSP 2.85326656

CWSQ 3.00835926

CWSR 3.16529261

CWSS 2.94892681

CWST 2.96427718

CWSV 3.51278338

CWSW 9.07399708

CWSY 5.60546705

CWTA 3.04287169

CWTC 3.89191259

CWTD 2.33785306

CWTE 2.45885475

CWTF 5.45934074

CWTG 2.65743545

CWTH 3.89396021

CWTI 3.71638416

CWTK 2.72240096

CWTL 3.96178304

CWTM 3.91724240

CWTN 2.77397078

CWTP 2.79751750

CWTQ 2.86437939

CWTR 3.20147917

CWTS 2.90419648

CWTT 2.93933610

CWTV 3.42061380

CWTW 8.66775846

CWTY 5.42219309

CWVA 3.46040890

CWVC 3.98812144

CWVD 2.85669862

CWVE 3.09169558

CWVF 5.71522420

CWVG 3.00189612

CWVH 4.35867559

CWVI 4.04307982

CWVK 3.16088945

CWVL 4.35376131

CWVM 4.28269002

CWVN 3.10251204

CWVP 3.13396621

CWVQ 3.30938270

CWVR 3.72507031

CWVS 3.30434341

CWVT 3.39664529

CWVV 3.75035261

CWVW 9.01585437

CWVY 5.80313733

CWWA 3.07739043

CWWC 3.72913412

CWWD 2.42294382

CWWE 2.81570255

CWWF 4.79708973

CWWG 2.51758711

CWWH 3.97417565

CWWI 3.69227838

CWWK 2.90353709

CWWL 3.66930107

CWWM 3.88133209

CWWN 2.85806926

CWWP 2.72401837

CWWQ 3.00070316

CWWR 3.42826392

CWWS 3.05293356

CWWT 2.96984398

CWWV 3.38784375

CWWW 7.58259208

CWWY 4.92421715

CWYA 2.97487643

CWYC 3.82915733

CWYD 2.42235511

CWYE 2.61269145

CWYF 5.01478933

CWYG 2.65092345

CWYH 3.76404998

CWYI 3.60275249

CWYK 2.76946491

CWYL 3.73685543

CWYM 3.78987862

CWYN 2.70023498

CWYP 2.70690724

CWYQ 2.96856641

CWYR 3.17755613

CWYS 2.89128340

CWYT 3.01802295

CWYV 3.40384622

CWYW 8.36955366

CWYY 4.92550533

CYAA 4.02512765

CYAC 4.89796159

CYAD 3.55286021

CYAE 3.74648943

CYAF 6.53309182

CYAG 3.45643950

CYAH 5.73961141

CYAI 4.69739289

CYAK 3.85415700

CYAL 4.82610291

CYAM 4.73702694

CYAN 4.16559911

CYAP 3.54899780

CYAQ 4.02210072

CYAR 4.12246423

CYAS 4.05753920

CYAT 4.15354117

CYAV 4.56236956

CYAW 6.32017556

CYAY 8.14415062

CYCA 7.57846005

CYCC 6.34541189

CYCD 7.17231307

CYCE 7.21726585

CYCF 11.16277654

CYCG 6.94701439

CYCH 9.21967831

CYCI 8.08435465

CYCK 7.56452001

CYCL 8.27300660

CYCM 8.30289981

CYCN 7.93469734

CYCP 6.98407741

CYCQ 7.74533962

CYCR 7.67024425

CYCS 7.79515304

CYCT 7.73868559

CYCV 8.01559496

CYCW 10.12575185

CYCY 14.41214265

CYDA 2.36007882

CYDC 3.40635319

CYDD 2.03343897

CYDE 2.12257386

CYDF 4.61217926

CYDG 1.80012706

CYDH 3.82839091

CYDI 3.01125455

CYDK 2.21891414

CYDL 3.08195272

CYDM 3.04288863

CYDN 2.55553943

CYDP 2.19414364

CYDQ 2.40618817

CYDR 2.39242867

CYDS 2.46726763

CYDT 2.62183536

CYDV 2.92157605

CYDW 4.31685166

CYDY 5.94767285

CYEA 2.28979626

CYEC 3.46137493

CYED 1.96349351

CYEE 2.03926883

CYEF 4.57207658

CYEG 1.81549712

CYEH 3.64214999

CYEI 3.00421536

CYEK 2.15539146

CYEL 2.96461387

CYEM 2.92837102

CYEN 2.46787428

CYEP 2.04849869

CYEQ 2.25384886

CYER 2.49494510

CYES 2.46673671

CYET 2.48853582

CYEV 2.82625949

CYEW 4.33523638

CYEY 5.75967054

CYFA 3.58943722

CYFC 4.20881797

CYFD 3.22671767

CYFE 3.33728296

CYFF 5.68103629

CYFG 2.87402295

CYFH 4.99837706

CYFI 4.12392358

CYFK 3.52203835

CYFL 4.11599212

CYFM 4.10075822

CYFN 3.75067229

CYFP 3.09829673

CYFQ 3.67207989

CYFR 3.65635236

CYFS 3.58858611

CYFT 3.59678322

CYFV 3.96215549

CYFW 5.41250864

CYFY 7.14468973

CYGA 3.16615321

CYGC 3.64046488

CYGD 2.60783338

CYGE 2.81091741

CYGF 5.47375269

CYGG 2.40114187

CYGH 4.46092930

CYGI 3.93745767

CYGK 2.98149648

CYGL 3.96469104

CYGM 3.90585192

CYGN 3.21985750

CYGP 2.69047784

CYGQ 3.23853937

CYGR 3.15468843

CYGS 3.14028331

CYGT 3.24857561

CYGV 3.66772905

CYGW 5.22040381

CYGY 6.84764308

CYHA 3.10552294

CYHC 3.69884914

CYHD 2.72053721

CYHE 2.80948752

CYHF 5.46312302

CYHG 2.50964364

CYHH 4.30161797

CYHI 3.74163224

CYHK 2.97272575

CYHL 3.81571619

CYHM 3.74515479

CYHN 3.26849593

CYHP 2.78271301

CYHQ 3.10235654

CYHR 3.26111087

CYHS 3.18200283

CYHT 3.27762039

CYHV 3.57492568

CYHW 5.03762475

CYHY 6.75301721

CYIA 3.75484712

CYIC 4.49295697

CYID 3.37851134

CYIE 3.52617029

CYIF 5.97769266

CYIG 3.17551303

CYIH 5.17241453

CYII 4.36421319

CYIK 3.67610485

CYIL 4.41848816

CYIM 4.33538315

CYIN 3.91161196

CYIP 3.36801442

CYIQ 3.76738907

CYIR 3.80099289

CYIS 3.79349183

CYIT 3.83595980

CYIV 4.14622292

CYIW 5.74855959

CYIY 7.39532150

CYKA 2.48716455

CYKC 3.48267587

CYKD 2.11218091

CYKE 2.20251877

CYKF 4.84902006

CYKG 2.04657202

CYKH 3.86845987

CYKI 3.16835745

CYKK 2.36201806

CYKL 3.18526217

CYKM 3.08524424

CYKN 2.71631950

CYKP 2.16143194

CYKQ 2.43065315

CYKR 2.53970646

CYKS 2.67683632

CYKT 2.71297685

CYKV 2.98711519

CYKW 4.49690026

CYKY 6.07698360

CYLA 3.65909144

CYLC 4.42476327

CYLD 3.13054372

CYLE 3.29845128

CYLF 5.81308735

CYLG 3.04586945

CYLH 5.08340255

CYLI 4.14006434

CYLK 3.46374404

CYLL 4.23160524

CYLM 4.17108340

CYLN 3.80565142

CYLP 3.09365599

CYLQ 3.64851075

CYLR 3.64750024

CYLS 3.65036378

CYLT 3.68247816

CYLV 3.99800085

CYLW 5.57421947

CYLY 7.26382081

CYMA 3.59279406

CYMC 4.42395792

CYMD 3.13504386

CYME 3.28801066

CYMF 5.89216173

CYMG 3.04433988

CYMH 5.00967321

CYMI 4.16880557

CYMK 3.41546082

CYML 4.25817006

CYMM 4.08686243

CYMN 3.66471139

CYMP 3.13249847

CYMQ 3.55921115

CYMR 3.63790291

CYMS 3.56710146

CYMT 3.62501624

CYMV 3.96410129

CYMW 5.83555254

CYMY 7.28900486

CYNA 2.98965997

CYNC 3.83139292

CYND 2.59183163

CYNE 2.68195793

CYNF 5.37198432

CYNG 2.44482544

CYNH 4.47681027

CYNI 3.59292830

CYNK 2.79956780

CYNL 3.78320936

CYNM 3.61945080

CYNN 3.10410151

CYNP 2.58038293

CYNQ 2.97464665

CYNR 3.01691480

CYNS 3.05926939

CYNT 3.18008017

CYNV 3.42730469

CYNW 5.17437888

CYNY 6.78900348

CYPA 2.66485045

CYPC 3.23394334

CYPD 2.18600255

CYPE 2.27772368

CYPF 4.75666149

CYPG 1.97929810

CYPH 3.87689693

CYPI 3.23489859

CYPK 2.46864057

CYPL 3.30084627

CYPM 3.19824635

CYPN 2.66927531

CYPP 2.22218628

CYPQ 2.63307738

CYPR 2.62657170

CYPS 2.73411904

CYPT 2.79239080

CYPV 3.05972481

CYPW 4.45165289

CYPY 6.09283549

CYQA 2.61452945

CYQC 3.58015958

CYQD 2.25865552

CYQE 2.25666407

CYQF 4.99784581

CYQG 2.20979940

CYQH 4.01763794

CYQI 3.23906919

CYQK 2.46676926

CYQL 3.34211742

CYQM 3.29975356

CYQN 2.80709521

CYQP 2.31650537

CYQQ 2.48858023

CYQR 2.68335022

CYQS 2.61843162

CYQT 2.74295693

CYQV 3.15110825

CYQW 4.64739027

CYQY 6.24539340

CYRA 2.77460190

CYRC 3.59064587

CYRD 2.32965948

CYRE 2.40454496

CYRF 5.02890942

CYRG 2.23182165

CYRH 4.18638481

CYRI 3.27002491

CYRK 2.53353787

CYRL 3.35355597

CYRM 3.29058699

CYRN 2.85678958

CYRP 2.46091116

CYRQ 2.68192139

CYRR 2.70719546

CYRS 2.80187542

CYRT 2.81515606

CYRV 3.20940908

CYRW 4.70643788

CYRY 6.41191806

CYSA 3.77831989

CYSC 4.70547178

CYSD 3.19243145

CYSE 3.34363953

CYSF 6.34608029

CYSG 3.16682170

CYSH 5.37081719

CYSI 4.37165818

CYSK 3.47642458

CYSL 4.53728981

CYSM 4.46098522

CYSN 3.84813298

CYSP 3.26957039

CYSQ 3.76375173

CYSR 3.74268678

CYSS 3.74761084

CYST 3.82164850

CYSV 4.18656209

CYSW 5.94116963

CYSY 7.86138427

CYTA 3.60597377

CYTC 4.49530196

CYTD 3.11632914

CYTE 3.20908552

CYTF 6.10677067

CYTG 3.07201501

CYTH 5.12498543

CYTI 4.27875881

CYTK 3.35132543

CYTL 4.40510459

CYTM 4.32150914

CYTN 3.69928825

CYTP 3.11901344

CYTQ 3.52666245

CYTR 3.65504476

CYTS 3.63265662

CYTT 3.64226115

CYTV 4.06782942

CYTW 5.74365695

CYTY 7.48630567

CYVA 3.99091940

CYVC 4.69104649

CYVD 3.60347797

CYVE 3.76838909

CYVF 6.34369930

CYVG 3.36086677

CYVH 5.58053901

CYVI 4.56255873

CYVK 3.85616093

CYVL 4.69411629

CYVM 4.66716754

CYVN 4.04092517

CYVP 3.52876897

CYVQ 3.99502976

CYVR 4.08007205

CYVS 4.01828145

CYVT 4.02003158

CYVV 4.36159697

CYVW 6.12015376

CYVY 7.85208969

CYWA 3.32088386

CYWC 3.76463451

CYWD 2.89454333

CYWE 2.98811288

CYWF 5.24337814

CYWG 2.74246486

CYWH 4.54279961

CYWI 3.88063026

CYWK 3.26250774

CYWL 3.84037496

CYWM 3.93218044

CYWN 3.32966876

CYWP 2.76798034

CYWQ 3.44059381

CYWR 3.33472236

CYWS 3.39328853

CYWT 3.43176093

CYWV 3.65466969

CYWW 4.82013625

CYWY 6.65041198

CYYA 3.55227060

CYYC 4.33178373

CYYD 3.12528016

CYYE 3.24255304

CYYF 5.75107298

CYYG 2.93327282

CYYH 4.96947538

CYYI 4.08994885

CYYK 3.41794218

CYYL 4.16114922

CYYM 4.15576869

CYYN 3.58373227

CYYP 2.99479479

CYYQ 3.56296700

CYYR 3.54927527

CYYS 3.53470744

CYYT 3.65954746

CYYV 3.92989350

CYYW 5.25747021

CYYY 7.15243713

DAAA 5.94671264

DAAC 4.44109627

DAAD 4.71908056

DAAE 4.91111468

DAAF 3.45304089

DAAG 4.53912645

DAAH 4.08466692

DAAI 3.91765754

DAAK 4.45874787

DAAL 3.99598354

DAAM 4.01100399

DAAN 4.21932477

DAAP 4.56580957

DAAQ 4.63775477

DAAR 4.51044041

DAAS 4.85431853

DAAT 4.58545519

DAAV 4.38389951

DAAW 3.36173989

DAAY 3.56484347

DACA 4.51450984

DACC 1.19434502

DACD 3.13372601

DACE 3.37520914

DACF 2.23379862

DACG 2.92785028

DACH 2.17860519

DACI 2.78058707

DACK 3.08579345

DACL 2.69423778

DACM 2.87223075

DACN 2.86689044

DACP 2.85766925

DACQ 3.10442061

DACR 2.89320355

DACS 3.60337312

DACT 3.26570759

DACV 3.13127677

DACW 1.82707877

DACY 2.36007882

DADA 10.73181738

DADC 7.72705859

DADD 6.80270827

DADE 7.11799039

DADF 6.20728864

DADG 7.50569120

DADH 6.45768223

DADI 6.86477191

DADK 6.84053444

DADL 6.72787901

DADM 6.92988905

DADN 6.75448385

DADP 7.20558038

DADQ 6.96392159

DADR 6.70239373

DADS 7.87524681

DADT 7.35989757

DADV 7.45535147

DADW 5.82310811

DADY 6.17044602

DAEA 7.57677124

DAEC 6.07623414

DAED 5.65609992

DAEE 5.90173241

DAEF 4.84219491

DAEG 5.91429459

DAEH 5.24098995

DAEI 5.47316157

DAEK 5.62960400

DAEL 5.35349208

DAEM 5.48069554

DAEN 5.54694705

DAEP 5.68727653

DAEQ 5.74168032

DAER 5.46313618

DAES 6.27796352

DAET 5.91518117

DAEV 5.90445175

DAEW 4.55644296

DAEY 4.90625936

DAFA 4.01227766

DAFC 2.72154391

DAFD 2.88143159

DAFE 3.07240132

DAFF 1.94568102

DAFG 2.85444000

DAFH 2.37404630

DAFI 2.51351221

DAFK 2.89436352

DAFL 2.35165388

DAFM 2.54812098

DAFN 2.73644892

DAFP 2.77189325

DAFQ 2.91458851

DAFR 2.61567823

DAFS 3.20677414

DAFT 2.90751329

DAFV 2.71979550

DAFW 1.78020753

DAFY 2.16691872

DAGA 6.18927827

DAGC 4.36656420

DAGD 4.61622266

DAGE 4.92510969

DAGF 3.65547707

DAGG 4.46783850

DAGH 4.05660633

DAGI 4.22840259

DAGK 4.57353310

DAGL 4.20013151

DAGM 4.26943209

DAGN 4.31862137

DAGP 4.59948099

DAGQ 4.68208871

DAGR 4.46977269

DAGS 5.02132527

DAGT 4.72104682

DAGV 4.69739780

DAGW 3.43679337

DAGY 3.75750713

DAHA 6.12143093

DAHC 4.24556123

DAHD 4.51416191

DAHE 4.75588991

DAHF 3.73478218

DAHG 4.59831931

DAHH 3.91910584

DAHI 4.30588595

DAHK 4.43569430

DAHL 4.22449200

DAHM 4.23838456

DAHN 4.24818523

DAHP 4.51976657

DAHQ 4.55690584

DAHR 4.33471587

DAHS 4.94825109

DAHT 4.63067250

DAHV 4.66578436

DAHW 3.53085451

DAHY 3.77355613

DAIA 4.14198350

DAIC 2.98900725

DAID 3.04841944

DAIE 3.28128077

DAIF 2.14662342

DAIG 2.99654746

DAIH 2.58896870

DAII 2.53551486

DAIK 3.10101962

DAIL 2.44223264

DAIM 2.56598657

DAIN 2.96302234

DAIP 2.97142711

DAIQ 3.03227715

DAIR 2.79278351

DAIS 3.37202126

DAIT 3.02486863

DAIV 2.77828039

DAIW 1.96566495

DAIY 2.36826230

DAKA 6.23744397

DAKC 4.95514707

DAKD 4.77642573

DAKE 4.98271013

DAKF 4.01086740

DAKG 4.79984048

DAKH 4.30128440

DAKI 4.56005664

DAKK 4.75308094

DAKL 4.39971889

DAKM 4.44957627

DAKN 4.65234036

DAKP 4.66899505

DAKQ 4.71230348

DAKR 4.39720160

DAKS 5.14339813

DAKT 4.82212053

DAKV 4.81649230

DAKW 3.69244948

DAKY 4.13364270

DALA 4.29794539

DALC 3.07132519

DALD 3.17469442

DALE 3.37695721

DALF 2.19768125

DALG 3.10986233

DALH 2.71928926

DALI 2.66260300

DALK 3.16177309

DALL 2.53961382

DALM 2.73226250

DALN 3.02793195

DALP 3.11111904

DALQ 3.16302864

DALR 2.94662097

DALS 3.48268219

DALT 3.16964965

DALV 2.93514665

DALW 2.00013711

DALY 2.38572082

DAMA 4.58813303

DAMC 3.37802888

DAMD 3.37681874

DAME 3.60644638

DAMF 2.46582707

DAMG 3.36764465

DAMH 2.89908145

DAMI 2.91674138

DAMK 3.31562304

DAML 2.84450924

DAMM 2.99512970

DAMN 3.16858840

DAMP 3.30951380

DAMQ 3.32814173

DAMR 3.12583837

DAMS 3.63716784

DAMT 3.33822306

DAMV 3.22231103

DAMW 2.33312385

DAMY 2.64818237

DANA 7.25044365

DANC 5.70379473

DAND 5.51717241

DANE 5.78656098

DANF 4.66221065

DANG 5.59769579

DANH 4.97398291

DANI 5.27760040

DANK 5.49779375

DANL 5.12505482

DANM 5.21891089

DANN 5.33635639

DANP 5.48274959

DANQ 5.50904216

DANR 5.11981478

DANS 5.99650488

DANT 5.64032508

DANV 5.65087413

DANW 4.35088884

DANY 4.76726006

DAPA 6.03820271

DAPC 4.34921097

DAPD 4.65093432

DAPE 4.93061011

DAPF 3.66381427

DAPG 4.50647520

DAPH 4.12690077

DAPI 4.24270153

DAPK 4.53187057

DAPL 4.22455859

DAPM 4.23873791

DAPN 4.28325724

DAPP 4.51408524

DAPQ 4.68081906

DAPR 4.45678793

DAPS 4.94220626

DAPT 4.63836116

DAPV 4.62940664

DAPW 3.44385897

DAPY 3.73710943

DAQA 6.45657167

DAQC 5.02684676

DAQD 5.01006733

DAQE 5.13525913

DAQF 4.08778704

DAQG 5.03590756

DAQH 4.47610229

DAQI 4.66385790

DAQK 4.83561480

DAQL 4.55832323

DAQM 4.59422456

DAQN 4.71307067

DAQP 4.85175835

DAQQ 4.95950181

DAQR 4.68726973

DAQS 5.30029340

DAQT 5.00617378

DAQV 5.01218304

DAQW 3.92963238

DAQY 4.14190852

DARA 5.89002708

DARC 4.54826305

DARD 4.49440957

DARE 4.67353860

DARF 3.63516710

DARG 4.50981878

DARH 4.02805433

DARI 4.14724374

DARK 4.23807656

DARL 4.11216956

DARM 4.15703408

DARN 4.14920033

DARP 4.38625194

DARQ 4.43582093

DARR 4.28819889

DARS 4.77100641

DART 4.51440558

DARV 4.53380970

DARW 3.45473523

DARY 3.65784283

DASA 6.46174364

DASC 5.03151462

DASD 5.08707543

DASE 5.31728281

DASF 4.11745608

DASG 4.98501803

DASH 4.43843509

DASI 4.64124264

DASK 4.91681322

DASL 4.57125633

DASM 4.58994060

DASN 4.72835721

DASP 4.95478450

DASQ 4.97139525

DASR 4.70450351

DASS 5.32321523

DAST 5.00484499

DASV 5.01298561

DASW 3.84163852

DASY 4.18672818

DATA 6.05631904

DATC 4.61413622

DATD 4.74973656

DATE 4.98743336

DATF 3.71018336

DATG 4.64564514

DATH 4.11087359

DATI 4.23417717

DATK 4.56259515

DATL 4.18548032

DATM 4.19219322

DATN 4.39052031

DATP 4.66024858

DATQ 4.67155249

DATR 4.38778620

DATS 4.94188177

DATT 4.64188023

DATV 4.57826634

DATW 3.50438579

DATY 3.79803733

DAVA 4.46263206

DAVC 3.15236108

DAVD 3.34582558

DAVE 3.55129736

DAVF 2.33735067

DAVG 3.25890615

DAVH 2.83040020

DAVI 2.72334253

DAVK 3.26512178

DAVL 2.68811343

DAVM 2.81089297

DAVN 3.10218844

DAVP 3.26526989

DAVQ 3.30961731

DAVR 3.11672986

DAVS 3.59498886

DAVT 3.27192450

DAVV 3.02222812

DAVW 2.16508149

DAVY 2.47254840

DAWA 4.26728626

DAWC 2.77808401

DAWD 2.96374681

DAWE 3.20450244

DAWF 2.22455124

DAWG 3.03602228

DAWH 2.58994070

DAWI 2.70816625

DAWK 2.94838258

DAWL 2.59415387

DAWM 2.77005838

DAWN 2.78966220

DAWP 2.91558592

DAWQ 3.04739708

DAWR 2.86013253

DAWS 3.32491979

DAWT 3.08819200

DAWV 2.97189994

DAWW 1.98517893

DAWY 2.27739314

DAYA 4.76567630

DAYC 3.50746384

DAYD 3.34430960

DAYE 3.60415585

DAYF 2.64870173

DAYG 3.48145916

DAYH 2.92376281

DAYI 3.19666682

DAYK 3.42820916

DAYL 3.02759631

DAYM 3.18534225

DAYN 3.33189140

DAYP 3.33177449

DAYQ 3.42543058

DAYR 3.10820829

DAYS 3.83394850

DAYT 3.50368439

DAYV 3.44191764

DAYW 2.32735566

DAYY 2.82819611

DCAA 4.36917432

DCAC 7.96933616

DCAD 3.14420665

DCAE 3.11487996

DCAF 3.69585829

DCAG 3.43325365

DCAH 3.69159371

DCAI 3.94927698

DCAK 3.10924352

DCAL 3.95201649

DCAM 3.81505570

DCAN 3.43593405

DCAP 3.17207586

DCAQ 3.27290576

DCAR 3.38661566

DCAS 4.13069432

DCAT 3.98143994

DCAV 4.25486902

DCAW 3.40569157

DCAY 3.74998208

DCCA 3.80988189

DCCC 5.59507474

DCCD 2.97007445

DCCE 2.72284648

DCCF 3.22809079

DCCG 2.75143950

DCCH 2.58572495

DCCI 3.41648893

DCCK 2.72241974

DCCL 3.24574746

DCCM 3.30826321

DCCN 3.06355740

DCCP 2.41779206

DCCQ 2.83879434

DCCR 2.74810536

DCCS 3.74713533

DCCT 3.57259489

DCCV 3.69343369

DCCW 2.65612242

DCCY 3.40635319

DCDA 7.72705859

DCDC 13.96051799

DCDD 5.65287258

DCDE 5.46583679

DCDF 6.87629187

DCDG 6.50990716

DCDH 6.30393517

DCDI 7.14296393

DCDK 5.62525182

DCDL 6.96664190

DCDM 7.00111907

DCDN 6.32517163

DCDP 5.83465234

DCDQ 5.83039975

DCDR 5.97006978

DCDS 7.38040167

DCDT 7.11701763

DCDV 7.54554162

DCDW 6.34514058

DCDY 6.80825080

DCEA 6.04638018

DCEC 9.99643999

DCED 4.33659604

DCEE 4.23836486

DCEF 5.42359870

DCEG 5.01223965

DCEH 5.04063981

DCEI 5.64356432

DCEK 4.40304431

DCEL 5.50095047

DCEM 5.50401180

DCEN 4.95513196

DCEP 4.40136043

DCEQ 4.57814144

DCER 4.57642509

DCES 5.82965211

DCET 5.56674997

DCEV 5.95367116

DCEW 4.84491823

DCEY 5.38911400

DCFA 3.06736151

DCFC 6.30181258

DCFD 2.02000223

DCFE 1.89659557

DCFF 2.35617839

DCFG 2.21832267

DCFH 2.34890874

DCFI 2.81241485

DCFK 1.99285356

DCFL 2.55105731

DCFM 2.57809353

DCFN 2.40309431

DCFP 1.82227201

DCFQ 2.09003666

DCFR 2.10714052

DCFS 2.91908003

DCFT 2.91712919

DCFV 2.91905663

DCFW 2.06493066

DCFY 2.47211734

DCGA 4.85749422

DCGC 8.65858443

DCGD 3.48013222

DCGE 3.49968127

DCGF 4.10222901

DCGG 3.62060448

DCGH 3.97584779

DCGI 4.45465231

DCGK 3.60075163

DCGL 4.30587352

DCGM 4.27753753

DCGN 3.83751410

DCGP 3.39352593

DCGQ 3.71559430

DCGR 3.69935426

DCGS 4.56419480

DCGT 4.39038247

DCGV 4.77437335

DCGW 3.85554783

DCGY 4.18232480

DCHA 4.92108079

DCHC 8.38507386

DCHD 3.46982077

DCHE 3.41186885

DCHF 4.24968171

DCHG 3.89512680

DCHH 3.87157783

DCHI 4.65888195

DCHK 3.52957011

DCHL 4.45733146

DCHM 4.37485673

DCHN 3.86281499

DCHP 3.45898176

DCHQ 3.67231640

DCHR 3.66485378

DCHS 4.69240585

DCHT 4.53270691

DCHV 4.90430598

DCHW 3.78958244

DCHY 4.23195640

DCIA 3.07730373

DCIC 6.52160847

DCID 2.28029283

DCIE 2.01384754

DCIF 2.55873004

DCIG 2.23003349

DCIH 2.51072795

DCII 2.76245171

DCIK 2.18686572

DCIL 2.60142489

DCIM 2.61499590

DCIN 2.41964407

DCIP 2.07290386

DCIQ 2.12435521

DCIR 2.12190076

DCIS 2.99692902

DCIT 2.77541171

DCIV 2.95392316

DCIW 2.21232004

DCIY 2.65775678

DCKA 4.93159353

DCKC 8.56917005

DCKD 3.42601748

DCKE 3.40513787

DCKF 4.47567898

DCKG 3.98775526

DCKH 4.17113585

DCKI 4.73964270

DCKK 3.58559678

DCKL 4.50831803

DCKM 4.42954396

DCKN 3.97879500

DCKP 3.57390316

DCKQ 3.58557551

DCKR 3.68245410

DCKS 4.62505670

DCKT 4.44447044

DCKV 4.89352647

DCKW 3.99459969

DCKY 4.48072650

DCLA 3.12290389

DCLC 6.48273853

DCLD 1.99770459

DCLE 2.03406724

DCLF 2.55471432

DCLG 2.33352718

DCLH 2.60988757

DCLI 2.85219260

DCLK 2.13797043

DCLL 2.64213866

DCLM 2.69880967

DCLN 2.48844709

DCLP 2.07470075

DCLQ 2.11372978

DCLR 2.17391437

DCLS 3.00444452

DCLT 2.82854751

DCLV 3.02300012

DCLW 2.13660928

DCLY 2.66612665

DCMA 3.43397772

DCMC 6.80166373

DCMD 2.25695116

DCME 2.29997506

DCMF 2.85587332

DCMG 2.81961864

DCMH 2.85474031

DCMI 3.19607599

DCMK 2.39545750

DCML 2.99305424

DCMM 2.91616221

DCMN 2.68326131

DCMP 2.33746151

DCMQ 2.36363626

DCMR 2.43788211

DCMS 3.26324486

DCMT 3.17959093

DCMV 3.35348890

DCMW 2.64362542

DCMY 3.00568142

DCNA 5.73791369

DCNC 10.05129574

DCND 4.22153357

DCNE 4.22382158

DCNF 5.19492273

DCNG 4.70170614

DCNH 4.80522987

DCNI 5.45324317

DCNK 4.35735295

DCNL 5.26180192

DCNM 5.26346058

DCNN 4.75049974

DCNP 4.23280495

DCNQ 4.44649642

DCNR 4.47061722

DCNS 5.53674833

DCNT 5.29690577

DCNV 5.69831999

DCNW 4.67276727

DCNY 5.14638069

DCPA 4.68866924

DCPC 8.23827631

DCPD 3.41067601

DCPE 3.34134113

DCPF 4.02744916

DCPG 3.62349961

DCPH 3.82305015

DCPI 4.39687571

DCPK 3.35372334

DCPL 4.24725951

DCPM 4.20822037

DCPN 3.75168517

DCPP 3.35700106

DCPQ 3.56113384

DCPR 3.53590202

DCPS 4.42669818

DCPT 4.26584268

DCPV 4.61374313

DCPW 3.54219602

DCPY 4.03171081

DCQA 5.09730822

DCQC 8.82973752

DCQD 3.57987163

DCQE 3.51315741

DCQF 4.49664464

DCQG 4.12525582

DCQH 4.18250996

DCQI 4.80097891

DCQK 3.67440643

DCQL 4.60475763

DCQM 4.52408192

DCQN 4.03375333

DCQP 3.68080200

DCQQ 3.71523834

DCQR 3.86539171

DCQS 4.70381304

DCQT 4.58724650

DCQV 5.02705466

DCQW 4.10716721

DCQY 4.51216172

DCRA 4.62033185

DCRC 8.04910105

DCRD 3.09590140

DCRE 3.10587658

DCRF 4.04712542

DCRG 3.62550940

DCRH 3.76080431

DCRI 4.34612852

DCRK 3.19718714

DCRL 4.15572510

DCRM 4.09356869

DCRN 3.55730588

DCRP 3.22226755

DCRQ 3.28780082

DCRR 3.34567829

DCRS 4.24361843

DCRT 4.15094428

DCRV 4.59879669

DCRW 3.60213105

DCRY 3.92247136

DCSA 5.07331513

DCSC 8.93580467

DCSD 3.76685095

DCSE 3.70602404

DCSF 4.50519776

DCSG 4.13454887

DCSH 4.22272496

DCSI 4.83465183

DCSK 3.80186527

DCSL 4.68921384

DCSM 4.56086621

DCSN 4.10919649

DCSP 3.73612586

DCSQ 3.82604955

DCSR 3.90891026

DCSS 4.77395559

DCST 4.64968022

DCSV 5.03805545

DCSW 4.13245773

DCSY 4.53840463

DCTA 4.63050964

DCTC 8.50828892

DCTD 3.35371366

DCTE 3.29962865

DCTF 4.02536680

DCTG 3.71161298

DCTH 3.86870328

DCTI 4.36289707

DCTK 3.39002595

DCTL 4.23769564

DCTM 4.13742989

DCTN 3.68390670

DCTP 3.36899800

DCTQ 3.43062324

DCTR 3.52596528

DCTS 4.34700639

DCTT 4.15717965

DCTV 4.57391710

DCTW 3.63905572

DCTY 4.06251712

DCVA 3.24564123

DCVC 6.77546484

DCVD 2.19412378

DCVE 2.12836021

DCVF 2.71408595

DCVG 2.41238475

DCVH 2.64363333

DCVI 2.91730020

DCVK 2.22078064

DCVL 2.84097383

DCVM 2.82719318

DCVN 2.48823276

DCVP 2.18119099

DCVQ 2.29478016

DCVR 2.33195614

DCVS 3.23911479

DCVT 2.93353973

DCVV 3.15092893

DCVW 2.35253760

DCVY 2.83141300

DCWA 3.36562330

DCWC 6.26033102

DCWD 2.19332839

DCWE 2.20757549

DCWF 2.66058370

DCWG 2.39488688

DCWH 2.64502454

DCWI 3.04762545

DCWK 2.53641813

DCWL 2.84567800

DCWM 2.99963996

DCWN 2.45852882

DCWP 1.87291456

DCWQ 2.53662248

DCWR 2.19864848

DCWS 3.00389037

DCWT 2.94667942

DCWV 3.30709171

DCWW 2.18305165

DCWY 2.58497723

DCYA 3.65215159

DCYC 7.33048370

DCYD 2.44427844

DCYE 2.40954823

DCYF 3.03974673

DCYG 2.81125453

DCYH 2.93653300

DCYI 3.46059990

DCYK 2.51288243

DCYL 3.21600869

DCYM 3.22322820

DCYN 3.07942879

DCYP 2.40329122

DCYQ 2.64165378

DCYR 2.44389819

DCYS 3.57147016

DCYT 3.48884724

DCYV 3.58231552

DCYW 2.67723147

DCYY 3.09549735

DDAA 4.30199027

DDAC 2.72594024

DDAD 6.72200017

DDAE 5.52974999

DDAF 2.40309170

DDAG 4.49641122

DDAH 4.24737296

DDAI 2.43902924

DDAK 4.45786486

DDAL 2.67671217

DDAM 2.86265793

DDAN 5.06990988

DDAP 4.47451095

DDAQ 4.69445862

DDAR 4.28512357

DDAS 4.66469489

DDAT 4.33729688

DDAV 2.84996092

DDAW 2.62368347

DDAY 2.94391124

DDCA 2.71892355

DDCC 0.88887206

DDCD 5.50561887

DDCE 4.34306424

DDCF 1.33495438

DDCG 2.79605465

DDCH 2.49377370

DDCI 1.50731252

DDCK 3.31389760

DDCL 1.55254862

DDCM 1.91991895

DDCN 3.88166402

DDCP 2.76641340

DDCQ 3.34353489

DDCR 2.93918543

DDCS 3.53387779

DDCT 3.13100862

DDCV 1.74118855

DDCW 1.53081257

DDCY 2.03343897

DDDA 6.80270827

DDDC 5.65287258

DDDD 11.91045057

DDDE 8.38885233

DDDF 5.00305801

DDDG 7.12417246

DDDH 6.89541303

DDDI 5.12642112

DDDK 6.86423332

DDDL 5.20017961

DDDM 5.58687785

DDDN 8.16846827

DDDP 6.79055246

DDDQ 7.18141959

DDDR 6.47511050

DDDS 7.32526739

DDDT 6.88242414

DDDV 5.46344787

DDDW 5.12982267

DDDY 5.63873431

DDEA 5.51580598

DDEC 4.26058403

DDED 8.36182295

DDEE 6.93270321

DDEF 3.85543341

DDEG 5.85619068

DDEH 5.55470291

DDEI 3.99480977

DDEK 5.68964216

DDEL 4.07715176

DDEM 4.40221912

DDEN 6.61082827

DDEP 5.59866355

DDEQ 5.93119503

DDER 5.30591733

DDES 6.01605654

DDET 5.61688694

DDEV 4.30324446

DDEW 3.88310711

DDEY 4.40917415

DDFA 2.36991320

DDFC 1.32850582

DDFD 4.97247414

DDFE 3.88676294

DDFF 1.05125014

DDFG 2.72716762

DDFH 2.71729333

DDFI 1.30661302

DDFK 3.09662303

DDFL 1.24256636

DDFM 1.50857146

DDFN 3.68803236

DDFP 2.74025900

DDFQ 3.13779152

DDFR 2.62952893

DDFS 3.11569828

DDFT 2.74388486

DDFV 1.38295876

DDFW 1.17908675

DDFY 1.71240606

DDGA 4.42017731

DDGC 2.83415332

DDGD 7.11612993

DDGE 5.83821883

DDGF 2.66268367

DDGG 4.43911180

DDGH 4.43344386

DDGI 2.87644006

DDGK 4.82231624

DDGL 2.98441295

DDGM 3.33070299

DDGN 5.43665340

DDGP 4.53263172

DDGQ 4.97097538

DDGR 4.47415819

DDGS 4.95703017

DDGT 4.61440688

DDGV 3.21378278

DDGW 2.77398819

DDGY 3.26036493

DDHA 4.25327659

DDHC 2.85983341

DDHD 6.88615234

DDHE 5.59807732

DDHF 2.72473253

DDHG 4.46714557

DDHH 4.26201935

DDHI 2.89381712

DDHK 4.62618206

DDHL 2.98716298

DDHM 3.19245438

DDHN 5.34173537

DDHP 4.45580448

DDHQ 4.75620505

DDHR 4.31178500

DDHS 4.76800869

DDHT 4.38175724

DDHV 3.12364431

DDHW 2.88666501

DDHY 3.27459119

DDIA 2.44036016

DDIC 1.56721017

DDID 5.06864283

DDIE 4.04054079

DDIF 1.24508315

DDIG 2.91455211

DDIH 2.88255669

DDII 1.31558138

DDIK 3.26418504

DDIL 1.35394317

DDIM 1.62307347

DDIN 3.83985949

DDIP 2.92644820

DDIQ 3.26147244

DDIR 2.77308487

DDIS 3.25091348

DDIT 2.84604830

DDIV 1.43915555

DDIW 1.39947796

DDIY 1.89983906

DDKA 4.46848150

DDKC 3.38418355

DDKD 6.87224962

DDKE 5.70581998

DDKF 3.07864376

DDKG 4.88280450

DDKH 4.64443667

DDKI 3.27373747

DDKK 4.98398291

DDKL 3.28169588

DDKM 3.50686328

DDKN 5.65398013

DDKP 4.69531506

DDKQ 4.94371264

DDKR 4.44855612

DDKS 5.04639733

DDKT 4.70075161

DDKV 3.43176520

DDKW 3.06155680

DDKY 3.70407665

DDLA 2.66154851

DDLC 1.57594697

DDLD 5.14179225

DDLE 4.08771314

DDLF 1.27551768

DDLG 3.01274621

DDLH 2.93888011

DDLI 1.33618908

DDLK 3.28899181

DDLL 1.36680337

DDLM 1.69512927

DDLN 3.85350532

DDLP 3.05386563

DDLQ 3.31805269

DDLR 2.87796372

DDLS 3.34393898

DDLT 2.95231519

DDLV 1.52919840

DDLW 1.42268916

DDLY 1.85036118

DDMA 2.79883970

DDMC 1.84931659

DDMD 5.46686623

DDME 4.39655240

DDMF 1.49262005

DDMG 3.23722660

DDMH 3.14992239

DDMI 1.58805370

DDMK 3.45005674

DDML 1.65717741

DDMM 1.97033620

DDMN 4.05164590

DDMP 3.24457315

DDMQ 3.46757250

DDMR 3.06943742

DDMS 3.49997332

DDMT 3.12158701

DDMV 1.75215093

DDMW 1.79584955

DDMY 2.13772767

DDNA 5.13036711

DDNC 4.04966699

DDND 8.18956171

DDNE 6.66035630

DDNF 3.68662696

DDNG 5.49720539

DDNH 5.34597724

DDNI 3.87848579

DDNK 5.65495518

DDNL 3.90248672

DDNM 4.15307208

DDNN 6.45940894

DDNP 5.29637826

DDNQ 5.72111718

DDNR 5.09439653

DDNS 5.77791302

DDNT 5.40247203

DDNV 4.08067219

DDNW 3.72962248

DDNY 4.30299097

DDPA 4.58303688

DDPC 2.94162775

DDPD 6.80763232

DDPE 5.74931774

DDPF 2.78703834

DDPG 4.59237220

DDPH 4.50870648

DDPI 3.01555188

DDPK 4.75212373

DDPL 3.15427163

DDPM 3.33679323

DDPN 5.34211525

DDPP 4.52119783

DDPQ 4.95452941

DDPR 4.45921752

DDPS 4.94184594

DDPT 4.58836983

DDPV 3.32907752

DDPW 2.89081736

DDPY 3.33563234

DDQA 4.64325769

DDQC 3.42325058

DDQD 7.09116042

DDQE 5.89183598

DDQF 3.03628914

DDQG 4.97319157

DDQH 4.71781482

DDQI 3.24011255

DDQK 4.92670881

DDQL 3.28863949

DDQM 3.51088244

DDQN 5.62553444

DDQP 4.82763761

DDQQ 5.08735338

DDQR 4.60916778

DDQS 5.11942714

DDQT 4.76743920

DDQV 3.46708711

DDQW 3.21959033

DDQY 3.61145872

DDRA 4.26938300

DDRC 3.05044810

DDRD 6.43879033

DDRE 5.29624745

DDRF 2.61813918

DDRG 4.51373270

DDRH 4.28053757

DDRI 2.79944724

DDRK 4.43424026

DDRL 2.88352779

DDRM 3.10720578

DDRN 5.08266853

DDRP 4.39833842

DDRQ 4.59077105

DDRR 4.25002050

DDRS 4.66737410

DDRT 4.31129258

DDRV 3.07540248

DDRW 2.77084330

DDRY 3.14241068

DDSA 4.72491547

DDSC 3.44470803

DDSD 7.29427545

DDSE 6.10712323

DDSF 3.13516840

DDSG 4.95653483

DDSH 4.76088289

DDSI 3.31051671

DDSK 5.09764418

DDSL 3.39697732

DDSM 3.60494154

DDSN 5.76436495

DDSP 4.88072189

DDSQ 5.18240654

DDSR 4.68306735

DDSS 5.25938389

DDST 4.88784073

DDSV 3.55474826

DDSW 3.24109259

DDSY 3.70850200

DDTA 4.38749448

DDTC 3.02241413

DDTD 6.85293888

DDTE 5.70094741

DDTF 2.70840718

DDTG 4.61218972

DDTH 4.41065005

DDTI 2.87856621

DDTK 4.71915112

DDTL 2.95713048

DDTM 3.16412313

DDTN 5.38322590

DDTP 4.56979192

DDTQ 4.84357562

DDTR 4.32961609

DDTS 4.84738833

DDTT 4.50580099

DDTV 3.15486478

DDTW 2.90453583

DDTY 3.26818051

DDVA 2.86548018

DDVC 1.74567089

DDVD 5.41360299

DDVE 4.31685058

DDVF 1.43335067

DDVG 3.24299383

DDVH 3.10813539

DDVI 1.48831396

DDVK 3.44011896

DDVL 1.54791008

DDVM 2.00466638

DDVN 4.02095804

DDVP 3.21644625

DDVQ 3.49139841

DDVR 3.06352135

DDVS 3.48661421

DDVT 3.10171879

DDVV 1.69575317

DDVW 1.57853965

DDVY 2.02037767

DDWA 2.56478375

DDWC 1.35628693

DDWD 4.98967585

DDWE 3.88388420

DDWF 1.10231444

DDWG 2.82367857

DDWH 2.79510021

DDWI 1.37688146

DDWK 3.13600104

DDWL 1.37326948

DDWM 1.80394079

DDWN 3.63496919

DDWP 2.73832690

DDWQ 3.17629260

DDWR 2.79933311

DDWS 3.16228930

DDWT 2.83466640

DDWV 1.59209695

DDWW 1.36174799

DDWY 1.64914518

DDYA 2.95637881

DDYC 2.07101998

DDYD 5.62882386

DDYE 4.43951660

DDYF 1.70689759

DDYG 3.39737519

DDYH 3.28515434

DDYI 1.91289989

DDYK 3.63502246

DDYL 1.88959219

DDYM 2.21144730

DDYN 4.34029162

DDYP 3.28008904

DDYQ 3.70151389

DDYR 3.10285965

DDYS 3.72078269

DDYT 3.31494666

DDYV 2.06272673

DDYW 1.73954863

DDYY 2.39681298

DEAA 4.58570808

DEAC 2.76999498

DEAD 5.62830324

DEAE 6.39129436

DEAF 2.54086214

DEAG 4.14368992

DEAH 4.23355048

DEAI 2.88298346

DEAK 4.88379962

DEAL 3.07639648

DEAM 3.27429344

DEAN 4.51431018

DEAP 4.43493222

DEAQ 5.18480320

DEAR 4.71984363

DEAS 4.54504594

DEAT 4.43482768

DEAV 3.30584062

DEAW 2.83853889

DEAY 3.09541058

DECA 2.91261436

DECC -0.11758032

DECD 4.20204933

DECE 5.10296697

DECF 1.49620626

DECG 2.44161973

DECH 2.37905212

DECI 1.89877632

DECK 3.67781368

DECL 1.91758511

DECM 2.23617641

DECN 3.29449474

DECP 2.73272839

DECQ 3.78247486

DECR 3.20240624

DECS 3.26358649

DECT 3.10939644

DECV 2.10490736

DECW 1.53894774

DECY 2.12257386

DEDA 7.11799039

DEDC 5.46583679

DEDD 8.38885233

DEDE 11.19832942

DEDF 5.16987174

DEDG 6.58537756

DEDH 6.71908346

DEDI 5.73004650

DEDK 7.37763921

DEDL 5.72391077

DEDM 6.08936569

DEDN 7.15952239

DEDP 6.87944362

DEDQ 7.86127909

DEDR 6.93424866

DEDS 7.10028154

DEDT 6.95114869

DEDV 6.09697561

DEDW 5.23697164

DEDY 5.73701578

DEEA 5.90037417

DEEC 4.29546674

DEED 7.09484727

DEEE 8.00761187

DEEF 4.12020520

DEEG 5.52946857

DEEH 5.59845521

DEEI 4.61819190

DEEK 6.24355616

DEEL 4.62283343

DEEM 4.93049478

DEEN 6.08482208

DEEP 5.66761224

DEEQ 6.56640907

DEER 5.81622863

DEES 5.98419805

DEET 5.81102444

DEEV 4.89742931

DEEW 4.15917548

DEEY 4.65857310

DEFA 2.52530154

DEFC 1.25863847

DEFD 3.85118830

DEFE 4.57582966

DEFF 1.20674950

DEFG 2.34809949

DEFH 2.64321373

DEFI 1.64255284

DEFK 3.39902226

DEFL 1.55026180

DEFM 1.81137264

DEFN 3.12678608

DEFP 2.65205524

DEFQ 3.51891337

DEFR 2.91596525

DEFS 2.91473784

DEFT 2.70387853

DEFV 1.74420367

DEFW 1.23491865

DEFY 1.86951583

DEGA 4.60733666

DEGC 2.79781385

DEGD 5.68020214

DEGE 6.73437034

DEGF 2.83716543

DEGG 3.98916329

DEGH 4.35463060

DEGI 3.31021834

DEGK 5.18598169

DEGL 3.34431953

DEGM 3.63886676

DEGN 4.74257833

DEGP 4.50785226

DEGQ 5.40383709

DEGR 4.82722635

DEGS 4.73892572

DEGT 4.65685009

DEGV 3.68433342

DEGW 2.93680058

DEGY 3.39403097

DEHA 4.51734898

DEHC 2.76515776

DEHD 5.57771264

DEHE 6.49527343

DEHF 2.92056355

DEHG 4.09596516

DEHH 4.21126082

DEHI 3.38357504

DEHK 5.03787791

DEHL 3.41415735

DEHM 3.64464102

DEHN 4.69974554

DEHP 4.48533866

DEHQ 5.27227089

DEHR 4.66299933

DEHS 4.64686084

DEHT 4.50131849

DEHV 3.59475145

DEHW 3.03497783

DEHY 3.40428833

DEIA 2.65147664

DEIC 1.49591087

DEID 4.04913955

DEIE 4.75549421

DEIF 1.42251291

DEIG 2.56093657

DEIH 2.86859808

DEII 1.70757018

DEIK 3.62935209

DEIL 1.70588749

DEIM 1.97491968

DEIN 3.40200506

DEIP 2.90241336

DEIQ 3.65726867

DEIR 3.10959519

DEIS 3.13129310

DEIT 2.90679809

DEIV 1.80986079

DEIW 1.52225569

DEIY 2.06328315

DEKA 4.80631379

DEKC 3.44152271

DEKD 5.88119230

DEKE 6.53262399

DEKF 3.34856748

DEKG 4.52367362

DEKH 4.68302913

DEKI 3.81478186

DEKK 5.41997368

DEKL 3.75360719

DEKM 3.97205915

DEKN 5.19534483

DEKP 4.72194218

DEKQ 5.46410068

DEKR 4.87825269

DEKS 5.01363904

DEKT 4.85145498

DEKV 3.96546394

DEKW 3.30793391

DEKY 3.88287852

DELA 2.88719436

DELC 1.55013480

DELD 4.11896368

DELE 4.80110067

DELF 1.40762938

DELG 2.66004218

DELH 2.94079065

DELI 1.76987175

DELK 3.66079961

DELL 1.73468257

DELM 2.22396370

DELN 3.39891516

DELP 3.02302017

DELQ 3.74834303

DELR 3.21608476

DELS 3.23313599

DELT 3.02390222

DELV 1.91832911

DELW 1.54850734

DELY 2.01652766

DEMA 3.06538771

DEMC 1.84151021

DEMD 4.45660670

DEME 5.15680517

DEMF 1.64920048

DEMG 2.93023412

DEMH 3.16194099

DEMI 2.03275953

DEMK 3.85894517

DEML 2.08182146

DEMM 2.33193923

DEMN 3.58023081

DEMP 3.21402784

DEMQ 3.92882084

DEMR 3.46841038

DEMS 3.39760894

DEMT 3.21441934

DEMV 2.21596557

DEMW 1.90352039

DEMY 2.28151793

DENA 5.38395523

DENC 3.98973788

DEND 6.68361543

DENE 7.71629807

DENF 3.84745042

DENG 5.03644059

DENH 5.30607970

DENI 4.40545196

DENK 6.08885424

DENL 4.33323475

DENM 4.60932877

DENN 5.77320459

DENP 5.35162818

DENQ 6.25491935

DENR 5.49365777

DENS 5.63032680

DENT 5.48581567

DENV 4.60303410

DENW 3.91288496

DENY 4.45411826

DEPA 4.77490606

DEPC 2.84936102

DEPD 5.65026722

DEPE 6.45858017

DEPF 2.89959140

DEPG 4.16214403

DEPH 4.42921777

DEPI 3.39711033

DEPK 5.09336991

DEPL 3.44932383

DEPM 3.63464791

DEPN 4.73404616

DEPP 4.46686018

DEPQ 5.34686165

DEPR 4.77198292

DEPS 4.77518707

DEPT 4.61480081

DEPV 3.72242056

DEPW 3.06034667

DEPY 3.44128401

DEQA 4.97299236

DEQC 3.45000142

DEQD 6.06837843

DEQE 6.78804185

DEQF 3.27629069

DEQG 4.62652490

DEQH 4.78305039

DEQI 3.79825457

DEQK 5.39440077

DEQL 3.77558500

DEQM 4.01458981

DEQN 5.15405278

DEQP 4.82122783

DEQQ 5.62963040

DEQR 5.06360576

DEQS 5.04984314

DEQT 4.92631828

DEQV 4.00903842

DEQW 3.54329809

DEQY 3.82655044

DERA 4.55394319

DERC 2.97955502

DERD 5.40922733

DERE 6.08578647

DERF 2.84065294

DERG 4.14825985

DERH 4.28391920

DERI 3.25118906

DERK 4.81857484

DERL 3.28601952

DERM 3.52521674

DERN 4.55404818

DERP 4.34610097

DERQ 5.04908272

DERR 4.64404126

DERS 4.56414816

DERT 4.42712918

DERV 3.54873620

DERW 2.97443856

DERY 3.29072350

DESA 4.94813181

DESC 3.43591989

DESD 6.15419578

DESE 6.94879407

DESF 3.31259815

DESG 4.56476216

DESH 4.74512344

DESI 3.74039803

DESK 5.48011591

DESL 3.78833415

DESM 3.96987599

DESN 5.18815756

DESP 4.87768544

DESQ 5.64477390

DESR 5.05189391

DESS 5.11006455

DEST 4.94232323

DESV 4.01684983

DESW 3.40602763

DESY 3.86718650

DETA 4.59993404

DETC 3.04033032

DETD 5.73217779

DETE 6.50948461

DETF 2.89544142

DETG 4.20352907

DETH 4.36472980

DETI 3.34636314

DETK 5.09768647

DETL 3.34087502

DETM 3.54339039

DETN 4.79558319

DETP 4.57315390

DETQ 5.30102929

DETR 4.71014848

DETS 4.71514856

DETT 4.58863730

DETV 3.59385129

DETW 3.02680404

DETY 3.44165340

DEVA 3.06789151

DEVC 1.66526938

DEVD 4.34462757

DEVE 5.04955557

DEVF 1.56959066

DEVG 2.86675205

DEVH 3.06702703

DEVI 1.85901920

DEVK 3.80534907

DEVL 1.89308023

DEVM 2.19161434

DEVN 3.50143115

DEVP 3.17873838

DEVQ 3.92247627

DEVR 3.42088879

DEVS 3.39696171

DEVT 3.17676729

DEVV 2.06092023

DEVW 1.71386683

DEVY 2.13417461

DEWA 2.73993818

DEWC 1.22949251

DEWD 3.81311930

DEWE 4.63177404

DEWF 1.28037461

DEWG 2.42233576

DEWH 2.82003061

DEWI 1.65361056

DEWK 3.36677603

DEWL 1.67166052

DEWM 2.00472103

DEWN 3.02585231

DEWP 2.65733617

DEWQ 3.55621637

DEWR 3.07521481

DEWS 2.98625136

DEWT 2.81800910

DEWV 1.97066431

DEWW 1.34912459

DEWY 1.79334189

DEYA 3.13992451

DEYC 1.91447822

DEYD 4.39585645

DEYE 5.21293907

DEYF 1.84153547

DEYG 2.96822005

DEYH 3.18337181

DEYI 2.30105026

DEYK 3.96153262

DEYL 2.21514540

DEYM 2.54262418

DEYN 3.73920457

DEYP 3.21572721

DEYQ 4.07221913

DEYR 3.40866512

DEYS 3.49040577

DEYT 3.30820264

DEYV 2.42065337

DEYW 1.86817908

DEYY 2.45358292

DFAA 3.58206873

DFAC 3.84126467

DFAD 2.88778334

DFAE 3.01546043

DFAF 6.87835972

DFAG 2.84057259

DFAH 4.12084676

DFAI 4.49491429

DFAK 2.89401189

DFAL 4.85112520

DFAM 4.53295625

DFAN 3.06241669

DFAP 3.17015271

DFAQ 3.18178236

DFAR 3.16555750

DFAS 3.25522921

DFAT 3.48397800

DFAV 4.26839427

DFAW 5.30798469

DFAY 5.72184004

DFCA 2.60188527

DFCC 0.65240559

DFCD 1.90127815

DFCE 2.03989003

DFCF 5.69829112

DFCG 1.58247903

DFCH 2.81833972

DFCI 3.56688261

DFCK 2.17258837

DFCL 3.80157786

DFCM 3.66327461

DFCN 2.23597738

DFCP 1.89833434

DFCQ 2.24894730

DFCR 2.25330970

DFCS 2.38200576

DFCT 2.65885823

DFCV 3.30568165

DFCW 3.94401962

DFCY 4.61217926

DFDA 6.20728864

DFDC 6.87629187

DFDD 5.00305801

DFDE 5.16987174

DFDF 12.24883358

DFDG 5.29885536

DFDH 6.91674432

DFDI 7.66816323

DFDK 5.24382780

DFDL 8.08092780

DFDM 7.73469877

DFDN 5.62121061

DFDP 5.59684982

DFDQ 5.56152028

DFDR 5.47063636

DFDS 5.83919857

DFDT 6.09490777

DFDV 7.23922500

DFDW 8.49525061

DFDY 9.25482103

DFEA 4.83144412

DFEC 5.47698227

DFED 3.91220052

DFEE 4.01736470

DFEF 8.88240455

DFEG 4.07258273

DFEH 5.38122663

DFEI 6.10807125

DFEK 4.02325888

DFEL 6.41721114

DFEM 6.09610074

DFEN 4.36800441

DFEP 4.27598059

DFEQ 4.30497487

DFER 4.20659658

DFES 4.53135951

DFET 4.75368303

DFEV 5.75041655

DFEW 6.81294686

DFEY 7.38427081

DFFA 2.34343328

DFFC 2.44131794

DFFD 1.85563947

DFFE 1.89651201

DFFF 4.99659201

DFFG 1.73281229

DFFH 2.69939048

DFFI 3.31505091

DFFK 1.91025971

DFFL 3.34647293

DFFM 3.13786251

DFFN 2.09663031

DFFP 2.00682947

DFFQ 2.08302894

DFFR 2.03128945

DFFS 2.15450981

DFFT 2.31919741

DFFV 2.95445938

DFFW 3.67475596

DFFY 4.11916996

DFGA 3.93453334

DFGC 3.90858876

DFGD 3.09405211

DFGE 3.38986779

DFGF 7.31080563

DFGG 2.90075219

DFGH 4.36771291

DFGI 5.03389610

DFGK 3.37270558

DFGL 5.27798715

DFGM 4.96203499

DFGN 3.40758760

DFGP 3.38764608

DFGQ 3.61484974

DFGR 3.48166335

DFGS 3.61976134

DFGT 3.84902006

DFGV 4.73735723

DFGW 5.58858457

DFGY 6.14555328

DFHA 3.84696334

DFHC 3.78118736

DFHD 2.99645946

DFHE 3.15544554

DFHF 7.33748516

DFHG 3.01058340

DFHH 4.05907314

DFHI 5.03491954

DFHK 3.21260525

DFHL 5.28240326

DFHM 4.90602509

DFHN 3.32345771

DFHP 3.33970359

DFHQ 3.41412400

DFHR 3.36370153

DFHS 3.53551821

DFHT 3.76422165

DFHV 4.67637901

DFHW 5.56290647

DFHY 6.03121722

DFIA 2.29803191

DFIC 2.64555007

DFID 2.03148270

DFIE 1.94107295

DFIF 5.19636291

DFIG 1.72029093

DFIH 2.79304413

DFII 3.24629914

DFIK 1.93805559

DFIL 3.41861677

DFIM 3.17645818

DFIN 2.10071934

DFIP 2.05594648

DFIQ 2.04028363

DFIR 2.00843540

DFIS 2.22811183

DFIT 2.34254167

DFIV 2.91075013

DFIW 4.13678094

DFIY 4.29731517

DFKA 3.94383695

DFKC 4.42984876

DFKD 3.12228846

DFKE 3.23686719

DFKF 7.40050867

DFKG 3.22789934

DFKH 4.43882948

DFKI 5.20218856

DFKK 3.31406658

DFKL 5.35485222

DFKM 5.02820334

DFKN 3.52814644

DFKP 3.51512561

DFKQ 3.39228288

DFKR 3.39929441

DFKS 3.66138951

DFKT 3.87610312

DFKV 4.79702385

DFKW 5.71588831

DFKY 6.20844430

DFLA 2.39575480

DFLC 2.68034685

DFLD 1.86029596

DFLE 1.92809155

DFLF 5.26829165

DFLG 1.78411624

DFLH 2.89778478

DFLI 3.35017533

DFLK 1.92302376

DFLL 3.45737633

DFLM 3.28555135

DFLN 2.19138179

DFLP 2.11021080

DFLQ 2.05212264

DFLR 1.99458935

DFLS 2.23746540

DFLT 2.40807889

DFLV 3.04653263

DFLW 4.01154930

DFLY 4.37696655

DFMA 2.61286430

DFMC 2.99504334

DFMD 2.04869513

DFME 2.13599800

DFMF 5.60812434

DFMG 1.98923183

DFMH 3.06567553

DFMI 3.60133015

DFMK 2.12392729

DFML 3.80192239

DFMM 3.50361970

DFMN 2.35021672

DFMP 2.35857218

DFMQ 2.24471647

DFMR 2.22423282

DFMS 2.43258545

DFMT 2.59542752

DFMV 3.33752959

DFMW 4.24969859

DFMY 4.65614870

DFNA 4.64273563

DFNC 5.11410434

DFND 3.83680095

DFNE 4.04909182

DFNF 8.56283047

DFNG 3.88194314

DFNH 5.20849023

DFNI 5.98591826

DFNK 4.06116069

DFNL 6.24199567

DFNM 5.89220438

DFNN 4.22802067

DFNP 4.19139452

DFNQ 4.22634850

DFNR 4.13655772

DFNS 4.42792294

DFNT 4.61103451

DFNV 5.56366757

DFNW 6.70919482

DFNY 7.18198399

DFPA 3.89118625

DFPC 3.87279856

DFPD 3.03104127

DFPE 3.26549006

DFPF 7.04080007

DFPG 2.99712669

DFPH 4.25533726

DFPI 4.94613074

DFPK 3.22804400

DFPL 5.19162295

DFPM 4.89726469

DFPN 3.31881514

DFPP 3.27891499

DFPQ 3.47811610

DFPR 3.37673883

DFPS 3.54951164

DFPT 3.74297836

DFPV 4.64344719

DFPW 5.48216992

DFPY 5.92308169

DFQA 4.08020169

DFQC 4.54319888

DFQD 3.20424968

DFQE 3.31941936

DFQF 7.61226302

DFQG 3.38277937

DFQH 4.54695518

DFQI 5.29443708

DFQK 3.33817054

DFQL 5.50463406

DFQM 5.11808225

DFQN 3.57748943

DFQP 3.59711322

DFQQ 3.51339572

DFQR 3.52389631

DFQS 3.73020170

DFQT 3.95163089

DFQV 4.92348283

DFQW 5.94454320

DFQY 6.36133719

DFRA 3.74045622

DFRC 4.06756457

DFRD 2.83116282

DFRE 2.95254676

DFRF 6.90776796

DFRG 2.94615518

DFRH 4.17295962

DFRI 4.77802864

DFRK 2.92859702

DFRL 4.99714317

DFRM 4.74259111

DFRN 3.19457367

DFRP 3.23433308

DFRQ 3.15953386

DFRR 3.15931077

DFRS 3.39427133

DFRT 3.60750770

DFRV 4.51683873

DFRW 5.31215518

DFRY 5.71141335

DFSA 4.13939026

DFSC 4.52225196

DFSD 3.40838511

DFSE 3.53281984

DFSF 7.66161104

DFSG 3.39306747

DFSH 4.58639602

DFSI 5.32639646

DFSK 3.54754962

DFSL 5.58654341

DFSM 5.20256329

DFSN 3.68389627

DFSP 3.67609976

DFSQ 3.70112367

DFSR 3.65068471

DFSS 3.83860897

DFST 4.03819336

DFSV 4.97394322

DFSW 5.98283809

DFSY 6.43859631

DFTA 3.79066435

DFTC 4.12103507

DFTD 3.11436386

DFTE 3.22705729

DFTF 7.17031772

DFTG 3.05368533

DFTH 4.26518130

DFTI 4.93382558

DFTK 3.20598856

DFTL 5.17133165

DFTM 4.81343226

DFTN 3.35581451

DFTP 3.37037244

DFTQ 3.38417309

DFTR 3.30801611

DFTS 3.50258652

DFTT 3.69199159

DFTV 4.57405281

DFTW 5.62294674

DFTY 6.00413982

DFVA 2.53142666

DFVC 2.88757592

DFVD 1.98209459

DFVE 2.09895335

DFVF 5.53537750

DFVG 1.88690324

DFVH 3.02229600

DFVI 3.43248862

DFVK 2.07116100

DFVL 3.66415310

DFVM 3.44190070

DFVN 2.20824098

DFVP 2.23134781

DFVQ 2.23677041

DFVR 2.17986949

DFVS 2.34594644

DFVT 2.50383667

DFVV 3.13082500

DFVW 4.26306772

DFVY 4.53066265

DFWA 2.66330153

DFWC 2.45399215

DFWD 1.82587376

DFWE 2.01004587

DFWF 5.14112598

DFWG 1.87542916

DFWH 2.87280076

DFWI 3.44145610

DFWK 1.98043454

DFWL 3.58129340

DFWM 3.45418703

DFWN 2.14852447

DFWP 1.97494522

DFWQ 2.17762943

DFWR 2.10240220

DFWS 2.27961286

DFWT 2.54269892

DFWV 3.18129784

DFWW 3.66359393

DFWY 4.13952009

DFYA 2.86965947

DFYC 3.08008418

DFYD 2.21170187

DFYE 2.33754949

DFYF 5.86641161

DFYG 2.24354165

DFYH 3.18490184

DFYI 3.93760182

DFYK 2.38444533

DFYL 4.05039425

DFYM 3.88210772

DFYN 2.57174824

DFYP 2.43032186

DFYQ 2.50328622

DFYR 2.41920517

DFYS 2.70514645

DFYT 2.86886265

DFYV 3.58732854

DFYW 4.27047389

DFYY 4.78171466

DGAA 4.26608869

DGAC 3.25204877

DGAD 4.66536129

DGAE 4.17782091

DGAF 2.51858358

DGAG 6.77018153

DGAH 3.70357388

DGAI 2.38135597

DGAK 3.89821967

DGAL 2.56241152

DGAM 2.80961296

DGAN 4.50104948

DGAP 4.10318527

DGAQ 3.92365378

DGAR 3.79730239

DGAS 4.39370303

DGAT 3.83500394

DGAV 2.80533206

DGAW 2.64077835

DGAY 2.80774048

DGCA 3.12304155

DGCC 0.73463171

DGCD 3.41880761

DGCE 3.12672540

DGCF 1.53604529

DGCG 5.40155758

DGCH 2.23970323

DGCI 1.50532614

DGCK 2.90811849

DGCL 1.52455561

DGCM 1.93171953

DGCN 3.31637034

DGCP 2.54677642

DGCQ 2.77901706

DGCR 2.66078562

DGCS 3.36768007

DGCT 2.76127750

DGCV 1.79166960

DGCW 1.51989308

DGCY 1.80012706

DGDA 7.50569120

DGDC 6.50990716

DGDD 7.12417246

DGDE 6.58537756

DGDF 5.29885536

DGDG 12.26574290

DGDH 6.33253296

DGDI 5.09809632

DGDK 6.45416221

DGDL 5.24094427

DGDM 5.71751066

DGDN 7.40364428

DGDP 6.47948232

DGDQ 6.45088992

DGDR 6.24506313

DGDS 7.38725408

DGDT 6.48194095

DGDV 5.53344589

DGDW 5.24275596

DGDY 5.41569636

DGEA 5.63671791

DGEC 4.74006829

DGED 5.73526766

DGEE 5.22884871

DGEF 3.85903172

DGEG 8.52354215

DGEH 4.88614826

DGEI 3.75248098

DGEK 5.03653765

DGEL 3.81892490

DGEM 4.18152929

DGEN 5.89051710

DGEP 5.12284884

DGEQ 5.01449088

DGER 4.76835579

DGES 5.73718890

DGET 5.04256911

DGEV 4.12226401

DGEW 3.83238661

DGEY 4.06536269

DGFA 2.73708437

DGFC 1.77800373

DGFD 3.08060576

DGFE 2.71776005

DGFF 1.27294213

DGFG 4.97620532

DGFH 2.20317231

DGFI 1.29936918

DGFK 2.69546943

DGFL 1.23481414

DGFM 1.60710311

DGFN 3.16749657

DGFP 2.50066082

DGFQ 2.51705433

DGFR 2.30651421

DGFS 2.96038887

DGFT 2.37793518

DGFV 1.48471603

DGFW 1.35016509

DGFY 1.58127454

DGGA 4.75363798

DGGC 3.47367016

DGGD 5.03035801

DGGE 4.70270684

DGGF 2.99474353

DGGG 7.04934117

DGGH 4.00424915

DGGI 2.96569025

DGGK 4.46572004

DGGL 3.05156876

DGGM 3.39070804

DGGN 4.95721529

DGGP 4.22374402

DGGQ 4.43600776

DGGR 4.18363154

DGGS 4.87091075

DGGT 4.30954170

DGGV 3.34090979

DGGW 2.95336983

DGGY 3.27090386

DGHA 4.58769341

DGHC 3.26395141

DGHD 4.65105158

DGHE 4.26753446

DGHF 2.93822067

DGHG 7.07298480

DGHH 3.79181544

DGHI 2.82870553

DGHK 4.11896881

DGHL 2.93352261

DGHM 3.20218539

DGHN 4.72375960

DGHP 4.13860938

DGHQ 4.10274874

DGHR 3.94213068

DGHS 4.60835382

DGHT 4.07967191

DGHV 3.14031064

DGHW 2.90489186

DGHY 3.07264344

DGIA 2.73116519

DGIC 1.93876031

DGID 3.21013640

DGIE 2.85303256

DGIF 1.35211137

DGIG 5.04818014

DGIH 2.40753887

DGII 1.31121067

DGIK 2.72748097

DGIL 1.25406224

DGIM 1.56591851

DGIN 3.30494789

DGIP 2.63887469

DGIQ 2.58242623

DGIR 2.35711965

DGIS 3.03944261

DGIT 2.48063285

DGIV 1.47836948

DGIW 1.56209472

DGIY 1.76816741

DGKA 4.57824827

DGKC 3.80294833

DGKD 4.89965056

DGKE 4.38554532

DGKF 3.12928047

DGKG 7.19155343

DGKH 4.08097615

DGKI 3.04042555

DGKK 4.36065516

DGKL 3.04838396

DGKM 3.32779441

DGKN 5.08912101

DGKP 4.30049740

DGKQ 4.16185871

DGKR 3.95659711

DGKS 4.75311682

DGKT 4.15369132

DGKV 3.29089953

DGKW 3.01425687

DGKY 3.39397347

DGLA 2.83548147

DGLC 2.00383314

DGLD 3.24352433

DGLE 2.84291168

DGLF 1.35394838

DGLG 5.12752597

DGLH 2.46993994

DGLI 1.29906781

DGLK 2.74282279

DGLL 1.30873948

DGLM 1.67099415

DGLN 3.32439980

DGLP 2.75223375

DGLQ 2.61953452

DGLR 2.42880763

DGLS 3.11975395

DGLT 2.55910200

DGLV 1.55334704

DGLW 1.40915845

DGLY 1.69570620

DGMA 3.08063918

DGMC 2.37499658

DGMD 3.48526954

DGME 3.08804259

DGMF 1.63014582

DGMG 5.47807039

DGMH 2.64812164

DGMI 1.58254770

DGMK 2.89619796

DGML 1.56136901

DGMM 1.86708482

DGMN 3.48284779

DGMP 2.97519197

DGMQ 2.77054924

DGMR 2.62358086

DGMS 3.31696648

DGMT 2.70993785

DGMV 1.80356849

DGMW 1.73246264

DGMY 1.98348732

DGNA 5.56066387

DGNC 4.66090056

DGND 5.75130788

DGNE 5.29899961

DGNF 3.87262974

DGNG 8.56716161

DGNH 4.83281695

DGNI 3.78504441

DGNK 5.18993216

DGNL 3.86504939

DGNM 4.17426477

DGNN 5.91447729

DGNP 4.99756560

DGNQ 5.07107941

DGNR 4.79521548

DGNS 5.66057813

DGNT 4.96325137

DGNV 4.07000009

DGNW 3.86329594

DGNY 4.14139588

DGPA 4.60250689

DGPC 3.33051124

DGPD 4.81223855

DGPE 4.45318392

DGPF 2.91901089

DGPG 6.76899375

DGPH 3.97952876

DGPI 2.86392376

DGPK 4.17183176

DGPL 3.01420594

DGPM 3.24396254

DGPN 4.73631654

DGPP 4.07837862

DGPQ 4.18630431

DGPR 3.95626090

DGPS 4.65826797

DGPT 4.10609061

DGPV 3.20230466

DGPW 2.88577443

DGPY 3.14702023

DGQA 4.74753349

DGQC 3.81023423

DGQD 4.99648547

DGQE 4.47646374

DGQF 3.15414254

DGQG 7.36916110

DGQH 4.16388100

DGQI 3.07904551

DGQK 4.35178864

DGQL 3.12694485

DGQM 3.39408453

DGQN 5.02548806

DGQP 4.45507450

DGQQ 4.32058139

DGQR 4.10125325

DGQS 4.84033895

DGQT 4.22559704

DGQV 3.37913580

DGQW 3.19272929

DGQY 3.40010605

DGRA 4.34056688

DGRC 3.40209869

DGRD 4.51431131

DGRE 4.04312293

DGRF 2.78302125

DGRG 6.69268632

DGRH 3.76219415

DGRI 2.69486864

DGRK 3.86045518

DGRL 2.75355562

DGRM 3.04090138

DGRN 4.52716695

DGRP 4.02870503

DGRQ 3.86297181

DGRR 3.76544204

DGRS 4.40359691

DGRT 3.85015890

DGRV 3.01550696

DGRW 2.79491923

DGRY 2.94236122

DGSA 4.89172105

DGSC 3.98039056

DGSD 5.14185134

DGSE 4.74517938

DGSF 3.26295517

DGSG 7.44019199

DGSH 4.20815410

DGSI 3.20547881

DGSK 4.52556895

DGSL 3.27298547

DGSM 3.51930679

DGSN 5.13238034

DGSP 4.53400858

DGSQ 4.44221584

DGSR 4.26089862

DGSS 4.99413601

DGST 4.37161818

DGSV 3.50012745

DGSW 3.28110434

DGSY 3.51739258

DGTA 4.52046423

DGTC 3.58000662

DGTD 4.83464326

DGTE 4.36957754

DGTF 2.89881252

DGTG 6.98664961

DGTH 3.88544887

DGTI 2.79335293

DGTK 4.18219646

DGTL 2.88310094

DGTM 3.13468355

DGTN 4.77236490

DGTP 4.24614768

DGTQ 4.10840712

DGTR 3.89910082

DGTS 4.61698361

DGTT 4.02552303

DGTV 3.11553513

DGTW 2.89710532

DGTY 3.13920525

DGVA 3.05250006

DGVC 2.18792403

DGVD 3.51888657

DGVE 3.11666436

DGVF 1.55759075

DGVG 5.35212260

DGVH 2.61565688

DGVI 1.48912376

DGVK 2.92230111

DGVL 1.50724324

DGVM 1.81474706

DGVN 3.48237650

DGVP 2.91913333

DGVQ 2.86020954

DGVR 2.65640696

DGVS 3.28935195

DGVT 2.72448201

DGVV 1.70249234

DGVW 1.61985011

DGVY 1.89068839

DGWA 2.98907963

DGWC 1.87786251

DGWD 3.17433672

DGWE 2.75584432

DGWF 1.48371237

DGWG 5.07091126

DGWH 2.38593845

DGWI 1.45767814

DGWK 2.65172755

DGWL 1.46572772

DGWM 1.98566637

DGWN 3.15880075

DGWP 2.70732316

DGWQ 2.65193190

DGWR 2.49838248

DGWS 3.11068830

DGWT 2.61452987

DGWV 1.76789770

DGWW 1.50694769

DGWY 1.80285638

DGYA 3.38982349

DGYC 2.52677897

DGYD 3.61894314

DGYE 3.24174880

DGYF 1.91559022

DGYG 5.72739576

DGYH 2.80586318

DGYI 1.93910789

DGYK 3.19305315

DGYL 1.83292487

DGYM 2.29224397

DGYN 3.75264441

DGYP 3.05726950

DGYQ 3.08558595

DGYR 2.78234929

DGYS 3.60541203

DGYT 2.98693031

DGYV 2.13753711

DGYW 1.79254090

DGYY 2.22008459

DHAA 3.92674708

DHAC 3.50997089

DHAD 4.51101193

DHAE 4.40937699

DHAF 3.81925797

DHAG 3.80303436

DHAH 6.80283360

DHAI 3.08813031

DHAK 4.30808973

DHAL 3.36929188

DHAM 3.42256527

DHAN 4.64510416

DHAP 3.83795471

DHAQ 4.61821217

DHAR 4.56864949

DHAS 4.18176090

DHAT 4.04788759

DHAV 3.32329442

DHAW 3.87337769

DHAY 4.71014494

DHCA 2.71549883

DHCC 0.80326351

DHCD 3.34384023

DHCE 3.39864079

DHCF 2.94452709

DHCG 2.42723381

DHCH 6.02108629

DHCI 2.25734250

DHCK 3.53206980

DHCL 2.39712081

DHCM 2.68860164

DHCN 3.62387450

DHCP 2.47385845

DHCQ 3.64583319

DHCR 3.48875082

DHCS 3.20569893

DHCT 3.02250780

DHCV 2.39724238

DHCW 3.17991364

DHCY 3.82839091

DHDA 6.45768223

DHDC 6.30393517

DHDD 6.89541303

DHDE 6.71908346

DHDF 6.91674432

DHDG 6.33253296

DHDH 12.60443224

DHDI 5.92541049

DHDK 6.94319030

DHDL 6.16596965

DHDM 6.30970442

DHDN 7.65157238

DHDP 6.28659139

DHDQ 7.53785074

DHDR 7.24763991

DHDS 6.83601544

DHDT 6.66754600

DHDV 6.03786232

DHDW 6.70121107

DHDY 7.96273129

DHEA 5.16918678

DHEC 5.02905719

DHED 5.61167375

DHEE 5.50029699

DHEF 5.43180259

DHEG 5.10307146

DHEH 8.56569070

DHEI 4.67111831

DHEK 5.61249818

DHEL 4.83643575

DHEM 4.95431779

DHEN 6.16174009

DHEP 4.97717862

DHEQ 5.98467324

DHER 5.78380700

DHES 5.55053431

DHET 5.36049199

DHEV 4.77143488

DHEW 5.25536798

DHEY 6.34020703

DHFA 2.25772900

DHFC 1.95207180

DHFD 2.94195713

DHFE 2.96317984

DHFF 2.25436512

DHFG 2.28607088

DHFH 5.02884278

DHFI 1.92094867

DHFK 3.04806147

DHFL 1.91854314

DHFM 2.32943888

DHFN 3.28242202

DHFP 2.29875752

DHFQ 3.11577344

DHFR 2.95499558

DHFS 2.76479935

DHFT 2.57584999

DHFV 1.95789381

DHFW 2.28504549

DHFY 3.21781367

DHGA 4.11560743

DHGC 3.57636379

DHGD 4.64996007

DHGE 4.69601772

DHGF 4.11874250

DHGG 3.73380411

DHGH 7.34809254

DHGI 3.57846145

DHGK 4.70618786

DHGL 3.71202605

DHGM 3.84614260

DHGN 4.91952206

DHGP 3.94830484

DHGQ 5.00763321

DHGR 4.84790158

DHGS 4.48901666

DHGT 4.37247366

DHGV 3.74430573

DHGW 4.00596844

DHGY 5.03294030

DHHA 4.15846127

DHHC 3.58412197

DHHD 4.73265790

DHHE 4.71901857

DHHF 4.27147188

DHHG 3.97418145

DHHH 7.19348962

DHHI 3.70419910

DHHK 4.68796521

DHHL 3.79666158

DHHM 3.96414205

DHHN 4.98782392

DHHP 3.98539846

DHHQ 4.91409011

DHHR 4.79791383

DHHS 4.50591903

DHHT 4.36927453

DHHV 3.79850847

DHHW 4.24031020

DHHY 5.12555587

DHIA 2.40541895

DHIC 2.29431376

DHID 3.08034525

DHIE 3.07529555

DHIF 2.43585516

DHIG 2.34023064

DHIH 5.17674125

DHII 1.99198980

DHIK 3.14590766

DHIL 1.96144334

DHIM 2.20178521

DHIN 3.38494500

DHIP 2.44384359

DHIQ 3.23957118

DHIR 3.18848503

DHIS 2.92426210

DHIT 2.68418320

DHIV 1.92189823

DHIW 2.50109124

DHIY 3.42053440

DHKA 4.18977640

DHKC 4.15192912

DHKD 4.75445249

DHKE 4.67757650

DHKF 4.46558188

DHKG 4.21303728

DHKH 7.01354134

DHKI 3.87354324

DHKK 4.74086047

DHKL 3.93761821

DHKM 4.01070140

DHKN 5.14828376

DHKP 4.13536480

DHKQ 4.88356523

DHKR 4.72774513

DHKS 4.61244620

DHKT 4.47139049

DHKV 3.88092870

DHKW 4.28824838

DHKY 5.30780953

DHLA 2.51782249

DHLC 2.42327415

DHLD 3.15965639

DHLE 3.14036217

DHLF 2.49744750

DHLG 2.46815411

DHLH 5.21065485

DHLI 2.04826758

DHLK 3.16237489

DHLL 2.02995221

DHLM 2.30621701

DHLN 3.45374802

DHLP 2.55360302

DHLQ 3.29304653

DHLR 3.18531172

DHLS 2.97328581

DHLT 2.78187580

DHLV 2.05579981

DHLW 2.44983352

DHLY 3.41846252

DHMA 2.71575232

DHMC 2.60533287

DHMD 3.40025039

DHME 3.38015963

DHMF 2.84852474

DHMG 2.73868411

DHMH 5.58417259

DHMI 2.23715477

DHMK 3.34427031

DHML 2.37339267

DHMM 2.51600768

DHMN 3.63035765

DHMP 2.87575231

DHMQ 3.49711241

DHMR 3.40318305

DHMS 3.15835220

DHMT 2.94532737

DHMV 2.30171633

DHMW 2.91888181

DHMY 3.68147574

DHNA 4.88867727

DHNC 4.83203460

DHND 5.56230027

DHNE 5.53202138

DHNF 5.24010343

DHNG 4.81177417

DHNH 8.48749658

DHNI 4.58106919

DHNK 5.58652165

DHNL 4.66219328

DHNM 4.78192993

DHNN 5.95587980

DHNP 4.82371036

DHNQ 5.83174477

DHNR 5.59751829

DHNS 5.36504834

DHNT 5.21760576

DHNV 4.60502940

DHNW 5.04848019

DHNY 6.14558203

DHPA 4.16919160

DHPC 3.54279434

DHPD 4.59421820

DHPE 4.56705100

DHPF 4.03999622

DHPG 3.83676750

DHPH 6.94672522

DHPI 3.54293704

DHPK 4.53382249

DHPL 3.74909331

DHPM 3.81186490

DHPN 4.79335093

DHPP 3.87797727

DHPQ 4.78303973

DHPR 4.67205782

DHPS 4.42102201

DHPT 4.27621247

DHPV 3.69178879

DHPW 4.03512628

DHPY 4.89811105

DHQA 4.38900202

DHQC 4.18323575

DHQD 4.89641622

DHQE 4.77812648

DHQF 4.56707025

DHQG 4.36583475

DHQH 7.23376177

DHQI 3.92261677

DHQK 4.83371798

DHQL 4.03679107

DHQM 4.10967843

DHQN 5.19548706

DHQP 4.25706064

DHQQ 5.03918898

DHQR 4.94001329

DHQS 4.71923746

DHQT 4.55896710

DHQV 3.96776802

DHQW 4.45365390

DHQY 5.34923695

DHRA 4.07244459

DHRC 3.85585241

DHRD 4.49667208

DHRE 4.42424412

DHRF 4.05284062

DHRG 3.93965343

DHRH 6.59460046

DHRI 3.48776471

DHRK 4.31021471

DHRL 3.61812980

DHRM 3.72983464

DHRN 4.70892221

DHRP 3.85816914

DHRQ 4.56163746

DHRR 4.50489708

DHRS 4.30465565

DHRT 4.18244196

DHRV 3.65546190

DHRW 4.04443524

DHRY 4.81790749

DHSA 4.43060006

DHSC 4.17026116

DHSD 4.99761303

DHSE 4.95308738

DHSF 4.55524396

DHSG 4.29871730

DHSH 7.38462839

DHSI 3.91581191

DHSK 4.96970016

DHSL 4.06097727

DHSM 4.11826583

DHSN 5.25111708

DHSP 4.31238450

DHSQ 5.15250961

DHSR 5.03970849

DHSS 4.77524840

DHST 4.61880754

DHSV 4.02016242

DHSW 4.44031878

DHSY 5.42641956

DHTA 4.02690048

DHTC 3.78169812

DHTD 4.67323330

DHTE 4.60916784

DHTF 4.11227292

DHTG 3.93172782

DHTH 6.94843410

DHTI 3.52174730

DHTK 4.60820298

DHTL 3.62789869

DHTM 3.73378974

DHTN 4.87597850

DHTP 3.97189177

DHTQ 4.80122347

DHTR 4.65337171

DHTS 4.40100144

DHTT 4.29065580

DHTV 3.62006009

DHTW 4.01676869

DHTY 4.96974900

DHVA 2.68087500

DHVC 2.36780492

DHVD 3.34240587

DHVE 3.30951264

DHVF 2.69126098

DHVG 2.59632597

DHVH 5.46880527

DHVI 2.09559485

DHVK 3.33944666

DHVL 2.17637486

DHVM 2.41042138

DHVN 3.58406526

DHVP 2.73768638

DHVQ 3.51299128

DHVR 3.41917670

DHVS 3.08893851

DHVT 2.94934450

DHVV 2.16869728

DHVW 2.73287603

DHVY 3.57977594

DHWA 2.60845821

DHWC 2.00538800

DHWD 3.15862932

DHWE 3.10576223

DHWF 2.55877044

DHWG 2.43325127

DHWH 5.32938087

DHWI 2.08954766

DHWK 3.13821186

DHWL 2.13932670

DHWM 2.53104437

DHWN 3.33072035

DHWP 2.43323166

DHWQ 3.34734637

DHWR 3.18760069

DHWS 3.05165347

DHWT 2.83106036

DHWV 2.15176294

DHWW 2.42588656

DHWY 3.33477212

DHYA 2.86851429

DHYC 2.69037371

DHYD 3.52787869

DHYE 3.47390783

DHYF 3.07653870

DHYG 2.79371019

DHYH 5.77501349

DHYI 2.60692683

DHYK 3.55527389

DHYL 2.66624331

DHYM 2.99686984

DHYN 3.85423311

DHYP 2.83115803

DHYQ 3.75743195

DHYR 3.51492153

DHYS 3.34473029

DHYT 3.15536374

DHYV 2.57585014

DHYW 2.90556681

DHYY 3.89034434

DIAA 4.14908730

DIAC 4.24331767

DIAD 3.12270990

DIAE 3.51573444

DIAF 4.60643268

DIAG 2.82590487

DIAH 3.53019039

DIAI 6.41778213

DIAK 3.51357002

DIAL 5.44371104

DIAM 4.99137182

DIAN 3.23479943

DIAP 3.55448438

DIAQ 3.62645452

DIAR 3.61444066

DIAS 3.50405854

DIAT 4.20001171

DIAV 5.73011451

DIAW 3.84948708

DIAY 4.04135916

DICA 3.01142756

DICC 0.95837148

DICD 1.93715487

DICE 2.45688723

DICF 3.47027844

DICG 1.43841200

DICH 1.96560787

DICI 5.24341137

DICK 2.69655492

DICL 4.28090612

DICM 3.92484638

DICN 2.17937320

DICP 2.27141443

DICQ 2.53066564

DICR 2.44922205

DICS 2.60919454

DICT 3.16944185

DICV 4.56906775

DICW 2.48993631

DICY 3.01125455

DIDA 6.86477191

DIDC 7.14296393

DIDD 5.12642112

DIDE 5.73004650

DIDF 7.66816323

DIDG 5.09809632

DIDH 5.92541049

DIDI 11.48776518

DIDK 5.92995803

DIDL 8.78986968

DIDM 8.29464977

DIDN 5.70871514

DIDP 6.08904989

DIDQ 6.00590657

DIDR 5.93921485

DIDS 6.01922494

DIDT 6.99959226

DIDV 9.28368133

DIDW 6.46032597

DIDY 6.76478653

DIEA 5.54286382

DIEC 5.79287604

DIED 4.14965763

DIEE 4.61335860

DIEF 6.18356508

DIEG 4.05748209

DIEH 4.72761231

DIEI 8.35251288

DIEK 4.74348809

DIEL 7.08677534

DIEM 6.70613722

DIEN 4.55951158

DIEP 4.72689482

DIEQ 4.81260174

DIER 4.71231752

DIES 4.85319948

DIET 5.62006924

DIEV 7.45144403

DIEW 5.14945790

DIEY 5.45909996

DIFA 2.73953700

DIFC 2.76322538

DIFD 1.85056558

DIFE 2.18371697

DIFF 3.14268656

DIFG 1.61251343

DIFH 2.13541691

DIFI 4.74691306

DIFK 2.32066301

DIFL 3.78736840

DIFM 3.44749471

DIFN 2.11099265

DIFP 2.18856245

DIFQ 2.24851361

DIFR 2.17262464

DIFS 2.26358366

DIFT 2.81780838

DIFV 4.07521040

DIFW 2.35393918

DIFY 2.71898701

DIGA 4.47750178

DIGC 4.21063960

DIGD 3.19436149

DIGE 3.83317346

DIGF 4.96037157

DIGG 2.82889699

DIGH 3.75493849

DIGI 6.91788531

DIGK 3.88976015

DIGL 5.87378751

DIGM 5.37954753

DIGN 3.46941032

DIGP 3.76389067

DIGQ 3.95108731

DIGR 3.86663711

DIGS 3.83345479

DIGT 4.55692169

DIGV 6.22847799

DIGW 4.14148569

DIGY 4.42170255

DIHA 4.39282628

DIHC 4.09571527

DIHD 3.12567909

DIHE 3.60490302

DIHF 4.96478897

DIHG 2.96793916

DIHH 3.49345530

DIHI 6.92665055

DIHK 3.70116256

DIHL 5.81060901

DIHM 5.35066654

DIHN 3.35835188

DIHP 3.66052744

DIHQ 3.77508392

DIHR 3.67986318

DIHS 3.74825185

DIHT 4.43739651

DIHV 6.13616728

DIHW 4.03843383

DIHY 4.32966626

DIIA 2.86649599

DIIC 2.96445502

DIID 1.98029201

DIIE 2.44607225

DIIF 3.37285045

DIIG 1.69785923

DIIH 2.34831031

DIII 4.81878001

DIIK 2.49859431

DIIL 3.95115378

DIIM 3.59722855

DIIN 2.26403316

DIIP 2.37398521

DIIQ 2.41336372

DIIR 2.29919889

DIIS 2.45001346

DIIT 2.95447337

DIIV 4.16403085

DIIW 2.64833097

DIIY 2.93666134

DIKA 4.59280458

DIKC 4.80044037

DIKD 3.36824704

DIKE 3.79138959

DIKF 5.30066130

DIKG 3.26447533

DIKH 3.92754199

DIKI 7.05391148

DIKK 3.91959275

DIKL 5.99077281

DIKM 5.54255296

DIKN 3.72039075

DIKP 3.89409333

DIKQ 3.89687503

DIKR 3.84265898

DIKS 3.94584151

DIKT 4.61318472

DIKV 6.26935929

DIKW 4.28200610

DIKY 4.68761879

DILA 2.95176946

DILC 3.01802160

DILD 2.01970084

DILE 2.34052792

DILF 3.35096389

DILG 1.75887622

DILH 2.35686738

DILI 4.91735533

DILK 2.47701963

DILL 3.97074107

DILM 3.69621463

DILN 2.23945658

DILP 2.40400197

DILQ 2.42405180

DILR 2.34946484

DILS 2.44224147

DILT 2.99062175

DILV 4.27410161

DILW 2.57916305

DILY 2.89174646

DIMA 3.16026837

DIMC 3.30100923

DIMD 2.19546242

DIME 2.61373024

DIMF 3.60390734

DIMG 1.99141697

DIMH 2.57413428

DIMI 5.28811782

DIMK 2.61937098

DIML 4.34118273

DIMM 3.91711405

DIMN 2.46976948

DIMP 2.67948841

DIMQ 2.63557126

DIMR 2.57802724

DIMS 2.59837368

DIMT 3.19210278

DIMV 4.62137707

DIMW 2.94032833

DIMY 3.17406023

DINA 5.22381515

DINC 5.43019445

DIND 3.99011984

DINE 4.53000079

DINF 5.96955680

DING 3.79521896

DINH 4.52445909

DINI 8.07158180

DINK 4.66919391

DINL 6.80926664

DINM 6.36682586

DINN 4.35038662

DINP 4.59968835

DINQ 4.63596926

DINR 4.49077878

DINS 4.61504623

DINT 5.37231881

DINV 7.16783173

DINW 4.91932267

DINY 5.27684327

DIPA 4.38257223

DIPC 4.15141397

DIPD 3.19718419

DIPE 3.66985965

DIPF 4.85896707

DIPG 2.95503476

DIPH 3.65931136

DIPI 6.66881071

DIPK 3.72860062

DIPL 5.71527767

DIPM 5.23212313

DIPN 3.38674100

DIPP 3.66817123

DIPQ 3.82031814

DIPR 3.75776767

DIPS 3.72400283

DIPT 4.37609252

DIPV 5.98851031

DIPW 3.98078089

DIPY 4.25449527

DIQA 4.72871103

DIQC 4.86411511

DIQD 3.45657761

DIQE 3.87191703

DIQF 5.34399040

DIQG 3.40717491

DIQH 4.02731677

DIQI 7.22649155

DIQK 3.99142932

DIQL 6.14596012

DIQM 5.68404849

DIQN 3.73906542

DIQP 3.98137705

DIQQ 4.02562050

DIQR 4.01148177

DIQS 4.01877935

DIQT 4.74877224

DIQV 6.45981586

DIQW 4.51418200

DIQY 4.68788141

DIRA 4.31507012

DIRC 4.42599788

DIRD 3.00018103

DIRE 3.45581409

DIRF 4.80964074

DIRG 2.93856338

DIRH 3.59116678

DIRI 6.56629816

DIRK 3.46164702

DIRL 5.57665358

DIRM 5.16958348

DIRN 3.29978943

DIRP 3.56726602

DIRQ 3.56423370

DIRR 3.53491570

DIRS 3.59784756

DIRT 4.27096007

DIRV 5.91711766

DIRW 3.97360173

DIRY 4.17596911

DISA 4.71345044

DISC 4.83457335

DISD 3.58854856

DISE 4.01584020

DISF 5.35561769

DISG 3.36023145

DISH 4.02191624

DISI 7.19141016

DISK 4.10568598

DISL 6.14362831

DISM 5.66170472

DISN 3.80572330

DISP 4.06282666

DISQ 4.09206275

DISR 4.05958862

DISS 4.07299273

DIST 4.77722531

DISV 6.42581993

DISW 4.42476887

DISY 4.70856989

DITA 4.35915132

DITC 4.46316062

DITD 3.28039949

DITE 3.71248324

DITF 4.95955921

DITG 3.03061624

DITH 3.69105665

DITI 6.72159799

DITK 3.75826550

DITL 5.75023985

DITM 5.25954109

DITN 3.47722005

DITP 3.75847662

DITQ 3.78012679

DITR 3.70392413

DITS 3.73054990

DITT 4.37427046

DITV 5.99785519

DITW 4.07938826

DITY 4.37114002

DIVA 3.05978497

DIVC 3.11692053

DIVD 2.13983055

DIVE 2.55807603

DIVF 3.56239940

DIVG 1.86185793

DIVH 2.61071375

DIVI 5.09294174

DIVK 2.65936551

DIVL 4.21244450

DIVM 3.85345494

DIVN 2.34003240

DIVP 2.58038242

DIVQ 2.63382936

DIVR 2.54069732

DIVS 2.60151448

DIVT 3.13264636

DIVV 4.43679089

DIVW 2.86528496

DIVY 3.06690600

DIWA 2.99076849

DIWC 2.66042237

DIWD 1.80991156

DIWE 2.35397961

DIWF 3.27716687

DIWG 1.81144544

DIWH 2.32461751

DIWI 4.88143934

DIWK 2.34677305

DIWL 3.96676164

DIWM 3.73368071

DIWN 2.08633925

DIWP 2.23200151

DIWQ 2.45806023

DIWR 2.28061254

DIWS 2.40337542

DIWT 2.96925496

DIWV 4.26247679

DIWW 2.34395534

DIWY 2.80538193

DIYA 3.32972539

DIYC 3.31766309

DIYD 2.35069608

DIYE 2.64351538

DIYF 3.84731652

DIYG 2.13119491

DIYH 2.62906502

DIYI 5.54814094

DIYK 2.82571144

DIYL 4.53157063

DIYM 4.21026936

DIYN 2.59816464

DIYP 2.78317414

DIYQ 2.76566285

DIYR 2.59879377

DIYS 2.79036308

DIYT 3.41201796

DIYV 4.82126888

DIYW 2.89669905

DIYY 3.30863100

DKAA 4.41063332

DKAC 2.98949913

DKAD 4.76689486

DKAE 5.11687948

DKAF 2.62071430

DKAG 4.06073371

DKAH 4.35771274

DKAI 3.09817326

DKAK 6.24933571

DKAL 3.26745985

DKAM 3.51940479

DKAN 4.62700663

DKAP 4.26226960

DKAQ 5.14064445

DKAR 5.43651824

DKAS 4.49617677

DKAT 4.43357761

DKAV 3.35536368

DKAW 2.85638139

DKAY 3.23170343

DKCA 2.80394430

DKCC 0.09014667

DKCD 3.36146437

DKCE 3.78963235

DKCF 1.60776728

DKCG 2.45930307

DKCH 2.78262661

DKCI 2.07173788

DKCK 4.98083579

DKCL 2.19989407

DKCM 2.51605318

DKCN 3.40379767

DKCP 2.68329753

DKCQ 3.76279135

DKCR 4.08009307

DKCS 3.26215235

DKCT 3.20435549

DKCV 2.16122400

DKCW 1.71190930

DKCY 2.21891414

DKDA 6.84053444

DKDC 5.62525182

DKDD 6.86423332

DKDE 7.37763921

DKDF 5.24382780

DKDG 6.45416221

DKDH 6.94319030

DKDI 5.92995803

DKDK 11.09847858

DKDL 5.97885159

DKDM 6.37496934

DKDN 7.27262154

DKDP 6.63694333

DKDQ 7.68622357

DKDR 8.32474311

DKDS 7.02184482

DKDT 7.00034007

DKDV 6.07418553

DKDW 5.33714087

DKDY 5.83055493

DKEA 5.68445010

DKEC 4.59104759

DKED 5.89010855

DKEE 6.26698027

DKEF 4.18707338

DKEG 5.41524864

DKEH 5.74106040

DKEI 4.81773237

DKEK 7.90769704

DKEL 4.84640535

DKEM 5.18161837

DKEN 6.15554240

DKEP 5.44046345

DKEQ 6.45376117

DKER 6.87969881

DKES 5.90520410

DKET 5.80156701

DKEV 4.91566828

DKEW 4.24823579

DKEY 4.72786074

DKFA 2.44311229

DKFC 1.36121071

DKFD 3.05081024

DKFE 3.39576458

DKFF 1.20664636

DKFG 2.33496476

DKFH 2.69549382

DKFI 1.76174315

DKFK 4.46775646

DKFL 1.72836667

DKFM 2.10114787

DKFN 3.18172567

DKFP 2.56771877

DKFQ 3.39736230

DKFR 3.61610003

DKFS 2.87026187

DKFT 2.74580124

DKFV 1.80537441

DKFW 1.40788022

DKFY 1.92529386

DKGA 4.44662622

DKGC 2.97077541

DKGD 4.80024180

DKGE 5.30671797

DKGF 2.90785894

DKGG 3.90429377

DKGH 4.50111995

DKGI 3.53289864

DKGK 6.61546852

DKGL 3.54519124

DKGM 3.90379591

DKGN 4.87262741

DKGP 4.36392132

DKGQ 5.35540869

DKGR 5.65462872

DKGS 4.72061738

DKGT 4.71265031

DKGV 3.72692765

DKGW 3.03576156

DKGY 3.52680345

DKHA 4.36177972

DKHC 2.87100513

DKHD 4.74302063

DKHE 5.13762259

DKHF 3.02499374

DKHG 4.02812226

DKHH 4.36942538

DKHI 3.66650945

DKHK 6.41723952

DKHL 3.70937467

DKHM 3.90653632

DKHN 4.79537910

DKHP 4.30154454

DKHQ 5.21590954

DKHR 5.51951686

DKHS 4.62717733

DKHT 4.55286988

DKHV 3.72151819

DKHW 3.13487594

DKHY 3.58348185

DKIA 2.57040735

DKIC 1.72231169

DKID 3.29403501

DKIE 3.66159763

DKIF 1.49088557

DKIG 2.51773034

DKIH 3.01459260

DKII 1.91040228

DKIK 4.65850772

DKIL 1.90133307

DKIM 2.19763428

DKIN 3.44866855

DKIP 2.79526701

DKIQ 3.61032307

DKIR 3.87106397

DKIS 3.09250949

DKIT 2.94553126

DKIV 1.88939549

DKIW 1.63381671

DKIY 2.18677604

DKKA 4.88053114

DKKC 3.82831468

DKKD 5.32819985

DKKE 5.66597862

DKKF 3.63820422

DKKG 4.71981555

DKKH 4.98919941

DKKI 4.16958175

DKKK 6.56295217

DKKL 4.17572586

DKKM 4.42634627

DKKN 5.41379187

DKKP 4.74592200

DKKQ 5.58745365

DKKR 5.79317026

DKKS 5.16432162

DKKT 5.06832804

DKKV 4.22544240

DKKW 3.60962879

DKKY 4.20879201

DKLA 2.74472354

DKLC 1.75224272

DKLD 3.33239513

DKLE 3.68798303

DKLF 1.44934641

DKLG 2.61089960

DKLH 3.02968795

DKLI 1.93191754

DKLK 4.74863847

DKLL 1.90575702

DKLM 2.26247507

DKLN 3.45873775

DKLP 2.88263963

DKLQ 3.69706768

DKLR 3.90483953

DKLS 3.19401664

DKLT 3.04101397

DKLV 1.97967338

DKLW 1.56946581

DKLY 2.14584134

DKMA 2.97685704

DKMC 2.10758118

DKMD 3.61674422

DKME 3.98907266

DKMF 1.76326836

DKMG 2.90455187

DKMH 3.25107096

DKMI 2.22956783

DKMK 5.04974003

DKML 2.24814993

DKMM 2.59343747

DKMN 3.66865886

DKMP 3.13021399

DKMQ 3.92434614

DKMR 4.21913895

DKMS 3.35247822

DKMT 3.25096171

DKMV 2.31319981

DKMW 1.97694628

DKMY 2.47024681

DKNA 5.35808964

DKNC 4.27011239

DKND 5.81398739

DKNE 6.26785745

DKNF 4.05177616

DKNG 5.07895997

DKNH 5.54205107

DKNI 4.72388279

DKNK 7.62501438

DKNL 4.72806355

DKNM 4.99087695

DKNN 5.97202160

DKNP 5.27007174

DKNQ 6.29263611

DKNR 6.57640032

DKNS 5.71148834

DKNT 5.69153853

DKNV 4.80786268

DKNW 4.05531216

DKNY 4.64274017

DKPA 4.56465558

DKPC 3.06079673

DKPD 4.80471329

DKPE 5.19463514

DKPF 2.99752592

DKPG 4.07057539

DKPH 4.52630447

DKPI 3.58237641

DKPK 6.34820500

DKPL 3.62995524

DKPM 3.82475061

DKPN 4.82016446

DKPP 4.34239861

DKPQ 5.27445185

DKPR 5.51937981

DKPS 4.70314451

DKPT 4.62003493

DKPV 3.74433859

DKPW 3.04216674

DKPY 3.56491786

DKQA 4.89620521

DKQC 3.71101916

DKQD 5.27640879

DKQE 5.56464415

DKQF 3.46163598

DKQG 4.65239534

DKQH 4.96673836

DKQI 4.07470316

DKQK 6.72319906

DKQL 4.07692260

DKQM 4.32430952

DKQN 5.28733497

DKQP 4.72933914

DKQQ 5.62109985

DKQR 5.92539419

DKQS 5.07898134

DKQT 5.00963052

DKQV 4.13132001

DKQW 3.56531776

DKQY 4.00606972

DKRA 4.43217571

DKRC 3.28291322

DKRD 4.72634860

DKRE 5.07414098

DKRF 3.00598118

DKRG 4.16439911

DKRH 4.41127454

DKRI 3.51118551

DKRK 5.97266163

DKRL 3.56231547

DKRM 3.85060441

DKRN 4.69810232

DKRP 4.23043641

DKRQ 5.04636461

DKRR 5.29562700

DKRS 4.57969113

DKRT 4.47821292

DKRV 3.68352527

DKRW 3.17249110

DKRY 3.49603385

DKSA 4.83517889

DKSC 3.66521944

DKSD 5.28910729

DKSE 5.64241129

DKSF 3.44462945

DKSG 4.55993660

DKSH 4.91954448

DKSI 4.01510230

DKSK 6.79888244

DKSL 4.03406550

DKSM 4.29368372

DKSN 5.30132449

DKSP 4.75934487

DKSQ 5.62156952

DKSR 5.92339337

DKSS 5.11409986

DKST 5.02291117

DKSV 4.09740015

DKSW 3.50212043

DKSY 4.00880661

DKTA 4.44806150

DKTC 3.28924073

DKTD 4.90845019

DKTE 5.27316984

DKTF 2.96698266

DKTG 4.18312726

DKTH 4.53557131

DKTI 3.54992848

DKTK 6.36814328

DKTL 3.58263558

DKTM 3.85463133

DKTN 4.91237693

DKTP 4.43223299

DKTQ 5.26080814

DKTR 5.50207244

DKTS 4.70046403

DKTT 4.67614055

DKTV 3.68850786

DKTW 3.10988010

DKTY 3.61461497

DKVA 2.91586518

DKVC 1.89200220

DKVD 3.52824949

DKVE 3.89541218

DKVF 1.59368883

DKVG 2.79009972

DKVH 3.18306153

DKVI 2.11647704

DKVK 4.98105080

DKVL 2.06358614

DKVM 2.39755323

DKVN 3.59372416

DKVP 3.04417700

DKVQ 3.89805484

DKVR 4.14517272

DKVS 3.31935472

DKVT 3.20133702

DKVV 2.11238079

DKVW 1.77287221

DKVY 2.25738314

DKWA 2.62166845

DKWC 1.31499123

DKWD 3.03116875

DKWE 3.40242641

DKWF 1.23094375

DKWG 2.34111383

DKWH 2.72995777

DKWI 1.79855775

DKWK 4.47135062

DKWL 1.78965562

DKWM 2.22207671

DKWN 3.10213786

DKWP 2.53291182

DKWQ 3.45692500

DKWR 3.69615797

DKWS 2.90182508

DKWT 2.80654609

DKWV 1.86799143

DKWW 1.39084162

DKWY 1.90740977

DKYA 3.04930406

DKYC 2.11545416

DKYD 3.61236829

DKYE 3.97113807

DKYF 1.92636613

DKYG 2.93242887

DKYH 3.37068867

DKYI 2.50149256

DKYK 5.13120662

DKYL 2.47704069

DKYM 2.94938293

DKYN 3.81356910

DKYP 3.15756955

DKYQ 4.00814150

DKYR 4.19096785

DKYS 3.50655325

DKYT 3.40316169

DKYV 2.51447105

DKYW 2.01312627

DKYY 2.62654448

DLAA 4.11486587

DLAC 4.09686556

DLAD 3.21813835

DLAE 3.56158955

DLAF 4.85230464

DLAG 2.92829566

DLAH 3.69167588

DLAI 5.33172852

DLAK 3.53487699

DLAL 6.19134188

DLAM 5.19391697

DLAN 3.20117079

DLAP 3.51770414

DLAQ 3.78524402

DLAR 3.77154076

DLAS 3.47081834

DLAT 3.91749693

DLAV 4.97573001

DLAW 4.13520711

DLAY 4.16322902

DLCA 2.90335739

DLCC 0.67153516

DLCD 1.99253998

DLCE 2.40963229

DLCF 3.68160613

DLCG 1.51537201

DLCH 2.15792449

DLCI 4.21434027

DLCK 2.50421867

DLCL 4.92846361

DLCM 4.13378352

DLCN 2.08255073

DLCP 2.08411377

DLCQ 2.64885154

DLCR 2.53829874

DLCS 2.41345705

DLCT 2.88527965

DLCV 3.78956825

DLCW 2.58547854

DLCY 3.08195272

DLDA 6.72787901

DLDC 6.96664190

DLDD 5.20017961

DLDE 5.72391077

DLDF 8.08092780

DLDG 5.24094427

DLDH 6.16596965

DLDI 8.78986968

DLDK 5.97885159

DLDL 11.17556796

DLDM 8.66348417

DLDN 5.68034939

DLDP 6.03578891

DLDQ 6.28536896

DLDR 6.11735120

DLDS 5.93862429

DLDT 6.59327341

DLDV 8.16700352

DLDW 6.86386438

DLDY 6.99014222

DLEA 5.40360557

DLEC 5.67304931

DLED 4.20124647

DLEE 4.60072394

DLEF 6.49973561

DLEG 4.11380895

DLEH 4.88689314

DLEI 7.09144358

DLEK 4.75890312

DLEL 8.02661603

DLEM 6.94240247

DLEN 4.54150552

DLEP 4.64191824

DLEQ 5.00397958

DLER 4.82603403

DLES 4.75948523

DLET 5.25781706

DLEV 6.56429469

DLEW 5.46551072

DLEY 5.62040674

DLFA 2.64660677

DLFC 2.54802580

DLFD 1.85699433

DLFE 2.19207959

DLFF 3.30472807

DLFG 1.59106092

DLFH 2.18404952

DLFI 3.85861762

DLFK 2.30162328

DLFL 4.35168011

DLFM 3.59764746

DLFN 2.02276593

DLFP 2.10728709

DLFQ 2.34132087

DLFR 2.19569523

DLFS 2.18630579

DLFT 2.54909940

DLFV 3.38431890

DLFW 2.54887564

DLFY 2.80495393

DLGA 4.29568277

DLGC 3.93058793

DLGD 3.24709702

DLGE 3.73315124

DLGF 5.08733648

DLGG 2.83289457

DLGH 3.75935305

DLGI 5.71286924

DLGK 3.80889935

DLGL 6.55453535

DLGM 5.55494417

DLGN 3.38659814

DLGP 3.67695153

DLGQ 3.98184910

DLGR 3.88928914

DLGS 3.64885160

DLGT 4.12615212

DLGV 5.32658954

DLGW 4.25535457

DLGY 4.38493973

DLHA 4.27226358

DLHC 3.89296622

DLHD 3.19847954

DLHE 3.61131414

DLHF 5.21345543

DLHG 3.00090949

DLHH 3.62036171

DLHI 5.81765402

DLHK 3.72031278

DLHL 6.56102511

DLHM 5.55575162

DLHN 3.38807146

DLHP 3.62752555

DLHQ 3.92076903

DLHR 3.78357782

DLHS 3.66608430

DLHT 4.12317487

DLHV 5.31270885

DLHW 4.34602654

DLHY 4.42657159

DLIA 2.67597383

DLIC 2.75937967

DLID 2.03542381

DLIE 2.32885736

DLIF 3.49330387

DLIG 1.68238636

DLIH 2.37358866

DLII 3.89899385

DLIK 2.42742353

DLIL 4.49764999

DLIM 3.72926974

DLIN 2.19793422

DLIP 2.24414858

DLIQ 2.46464443

DLIR 2.36353481

DLIS 2.32114475

DLIT 2.61264963

DLIV 3.43403479

DLIW 2.83557304

DLIY 2.99602430

DLKA 4.42432098

DLKC 4.59219164

DLKD 3.40117730

DLKE 3.72200001

DLKF 5.46652590

DLKG 3.24751414

DLKH 3.98306289

DLKI 5.99832673

DLKK 3.88149929

DLKL 6.64792832

DLKM 5.68692193

DLKN 3.70996786

DLKP 3.73932664

DLKQ 3.95754994

DLKR 3.85845019

DLKS 3.82808062

DLKT 4.24829568

DLKV 5.44040845

DLKW 4.51495470

DLKY 4.74716721

DLLA 2.86229902

DLLC 2.88944736

DLLD 2.06595882

DLLE 2.37846086

DLLF 3.56761823

DLLG 1.78730426

DLLH 2.48966167

DLLI 4.03534724

DLLK 2.49135284

DLLL 4.56958519

DLLM 3.87468648

DLLN 2.25069323

DLLP 2.34368697

DLLQ 2.56488340

DLLR 2.48207568

DLLS 2.40617132

DLLT 2.74555524

DLLV 3.59752181

DLLW 2.81598715

DLLY 3.04139959

DLMA 3.05326590

DLMC 3.15307778

DLMD 2.21277949

DLME 2.55125928

DLMF 3.75969435

DLMG 1.94161985

DLMH 2.63452007

DLMI 4.32138467

DLMK 2.60403726

DLML 4.95673044

DLMM 4.05196921

DLMN 2.36934628

DLMP 2.56227352

DLMQ 2.69353031

DLMR 2.63471855

DLMS 2.52454286

DLMT 2.87100363

DLMV 3.86403084

DLMW 3.14329727

DLMY 3.25404815

DLNA 5.05185475

DLNC 5.22481334

DLND 4.06977396

DLNE 4.49958030

DLNF 6.23136298

DLNG 3.83675986

DLNH 4.63059526

DLNI 6.83482121

DLNK 4.64167435

DLNL 7.69314083

DLNM 6.58767632

DLNN 4.30776872

DLNP 4.53006459

DLNQ 4.75127819

DLNR 4.57552377

DLNS 4.49841422

DLNT 4.98359837

DLNV 6.26058757

DLNW 5.20503567

DLNY 5.40062125

DLPA 4.27774472

DLPC 3.97492007

DLPD 3.26804602

DLPE 3.65364112

DLPF 5.02945059

DLPG 2.93664230

DLPH 3.74606474

DLPI 5.60240164

DLPK 3.67880349

DLPL 6.36065059

DLPM 5.38735481

DLPN 3.36344469

DLPP 3.55149925

DLPQ 3.88777706

DLPR 3.81354682

DLPS 3.63471936

DLPT 4.04973399

DLPV 5.20024603

DLPW 4.21878607

DLPY 4.32951173

DLQA 4.61078143

DLQC 4.72295452

DLQD 3.55503144

DLQE 3.88341911

DLQF 5.59412352

DLQG 3.42578355

DLQH 4.15099241

DLQI 6.15249058

DLQK 3.98970482

DLQL 6.88250268

DLQM 5.87583416

DLQN 3.73622405

DLQP 3.88788191

DLQQ 4.16096092

DLQR 4.08571911

DLQS 3.92873499

DLQT 4.40633329

DLQV 5.64785736

DLQW 4.75273036

DLQY 4.80804688

DLRA 4.24428754

DLRC 4.26206122

DLRD 3.11681146

DLRE 3.46720226

DLRF 5.01722788

DLRG 3.00646915

DLRH 3.71881690

DLRI 5.55615011

DLRK 3.45664950

DLRL 6.23235953

DLRM 5.35349182

DLRN 3.27474291

DLRP 3.50640065

DLRQ 3.69581054

DLRR 3.64110193

DLRS 3.53839830

DLRT 3.99129540

DLRV 5.16404713

DLRW 4.20748475

DLRY 4.27286372

DLSA 4.57790318

DLSC 4.64355359

DLSD 3.66924003

DLSE 4.02612062

DLSF 5.59570186

DLSG 3.37814185

DLSH 4.10514687

DLSI 6.11750520

DLSK 4.08897916

DLSL 6.88203507

DLSM 5.84613843

DLSN 3.77953873

DLSP 3.98500358

DLSQ 4.20965705

DLSR 4.11964512

DLSS 3.95852276

DLST 4.40890870

DLSV 5.62417629

DLSW 4.67563122

DLSY 4.81702083

DLTA 4.24306634

DLTC 4.28467552

DLTD 3.30739911

DLTE 3.67271770

DLTF 5.16134803

DLTG 3.05278271

DLTH 3.76362590

DLTI 5.67108367

DLTK 3.70631027

DLTL 6.41694122

DLTM 5.41761998

DLTN 3.41034941

DLTP 3.65580413

DLTQ 3.88471172

DLTR 3.76733761

DLTS 3.60501020

DLTT 4.01169126

DLTV 5.18644368

DLTW 4.27917237

DLTY 4.42045727

DLVA 2.91785953

DLVC 2.94872870

DLVD 2.21376879

DLVE 2.47881984

DLVF 3.70408342

DLVG 1.91058370

DLVH 2.53758412

DLVI 4.12931577

DLVK 2.54868613

DLVL 4.80161365

DLVM 3.99111644

DLVN 2.25889114

DLVP 2.46418406

DLVQ 2.66665452

DLVR 2.57359019

DLVS 2.43970247

DLVT 2.80318961

DLVV 3.69398485

DLVW 2.96994971

DLVY 3.10991868

DLWA 2.94219037

DLWC 2.48134223

DLWD 1.92238586

DLWE 2.31750386

DLWF 3.44238263

DLWG 1.75210454

DLWH 2.35198824

DLWI 3.98497436

DLWK 2.32706798

DLWL 4.57979427

DLWM 3.85860676

DLWN 2.12485054

DLWP 2.14622508

DLWQ 2.52147371

DLWR 2.38739124

DLWS 2.28318897

DLWT 2.72403245

DLWV 3.60841661

DLWW 2.52744527

DLWY 2.81072635

DLYA 3.15965355

DLYC 3.14904994

DLYD 2.25855804

DLYE 2.61643832

DLYF 4.01606633

DLYG 2.08264632

DLYH 2.72252494

DLYI 4.57654772

DLYK 2.77681403

DLYL 5.14655076

DLYM 4.34760031

DLYN 2.54836751

DLYP 2.59251447

DLYQ 2.83124567

DLYR 2.64355672

DLYS 2.68618132

DLYT 3.11864308

DLYV 4.06966710

DLYW 3.08340129

DLYY 3.41050717

DMAA 4.12598754

DMAC 3.99194892

DMAD 3.45148446

DMAE 3.78642376

DMAF 4.53170254

DMAG 3.18286550

DMAH 3.75285807

DMAI 4.91300843

DMAK 3.81995669

DMAL 5.20579259

DMAM 6.44616939

DMAN 3.57496983

DMAP 3.40868260

DMAQ 4.17084613

DMAR 3.88235090

DMAS 3.76950172

DMAT 4.07577784

DMAV 4.55731357

DMAW 4.02719567

DMAY 4.06635142

DMCA 3.06510257

DMCC 0.71820094

DMCD 2.34197160

DMCE 2.85986026

DMCF 3.45035186

DMCG 1.97960117

DMCH 2.21803581

DMCI 3.86971716

DMCK 2.88019208

DMCL 4.22377171

DMCM 5.55914495

DMCN 2.48436415

DMCP 2.20116889

DMCQ 3.07673703

DMCR 2.73719587

DMCS 2.82845562

DMCT 3.19776014

DMCV 3.53457223

DMCW 2.56235600

DMCY 3.04288863

DMDA 6.92988905

DMDC 7.00111907

DMDD 5.58687785

DMDE 6.08936569

DMDF 7.73469877

DMDG 5.71751066

DMDH 6.30970442

DMDI 8.29464977

DMDK 6.37496934

DMDL 8.66348417

DMDM 11.91988976

DMDN 6.15754786

DMDP 5.91577730

DMDQ 6.90262837

DMDR 6.32636179

DMDS 6.44455540

DMDT 6.96645949

DMDV 7.73497477

DMDW 6.73922999

DMDY 6.88339809

DMEA 5.53461885

DMEC 5.61260620

DMED 4.56667067

DMEE 4.94755545

DMEF 6.15955501

DMEG 4.52293190

DMEH 5.00778776

DMEI 6.70334122

DMEK 5.12309156

DMEL 6.95580964

DMEM 8.46940413

DMEN 4.98277039

DMEP 4.54799221

DMEQ 5.46522392

DMER 5.05601390

DMES 5.18905831

DMET 5.57943562

DMEV 6.22999402

DMEW 5.36602988

DMEY 5.48467992

DMFA 2.71643724

DMFC 2.56215465

DMFD 2.18446922

DMFE 2.42546206

DMFF 3.01402433

DMFG 1.90977701

DMFH 2.37116611

DMFI 3.48582864

DMFK 2.56390295

DMFL 3.56073101

DMFM 4.65941382

DMFN 2.38683725

DMFP 2.04376995

DMFQ 2.76323057

DMFR 2.40765303

DMFS 2.50981178

DMFT 2.72296087

DMFV 3.08192684

DMFW 2.48573741

DMFY 2.78127874

DMGA 4.38233993

DMGC 3.93976173

DMGD 3.56191488

DMGE 4.06059922

DMGF 4.82918327

DMGG 3.19926340

DMGH 3.92118119

DMGI 5.33867629

DMGK 4.12667509

DMGL 5.57580363

DMGM 6.89648916

DMGN 3.77113356

DMGP 3.55923609

DMGQ 4.46811017

DMGR 4.09341616

DMGS 4.04006056

DMGT 4.38689941

DMGV 4.94645506

DMGW 4.26763736

DMGY 4.33170936

DMHA 4.37773274

DMHC 3.89137683

DMHD 3.46933103

DMHE 3.89901810

DMHF 4.96244447

DMHG 3.35069897

DMHH 3.74408055

DMHI 5.40340785

DMHK 4.07343525

DMHL 5.61727164

DMHM 6.83797428

DMHN 3.78029771

DMHP 3.56376102

DMHQ 4.34551031

DMHR 3.99730465

DMHS 4.06258652

DMHT 4.33067128

DMHV 4.96651593

DMHW 4.23423593

DMHY 4.38007857

DMIA 2.73539389

DMIC 2.73391318

DMID 2.24738161

DMIE 2.55727358

DMIF 3.18389529

DMIG 1.98054569

DMIH 2.52943626

DMII 3.51310622

DMIK 2.79026388

DMIL 3.69393146

DMIM 4.70459674

DMIN 2.53112276

DMIP 2.20991764

DMIQ 2.84061784

DMIR 2.51845996

DMIS 2.58000719

DMIT 2.77621470

DMIV 3.11757207

DMIW 2.75511732

DMIY 2.91016600

DMKA 4.54021543

DMKC 4.55590463

DMKD 3.73057116

DMKE 4.03824823

DMKF 5.15735230

DMKG 3.61456547

DMKH 4.14680307

DMKI 5.59913822

DMKK 4.20078158

DMKL 5.75722656

DMKM 6.86883794

DMKN 4.04941539

DMKP 3.70672915

DMKQ 4.40273519

DMKR 4.13948104

DMKS 4.19258332

DMKT 4.51297545

DMKV 5.12212015

DMKW 4.45567030

DMKY 4.65271270

DMLA 2.88326210

DMLC 2.79172324

DMLD 2.25623991

DMLE 2.57417291

DMLF 3.23757771

DMLG 2.04024280

DMLH 2.60260067

DMLI 3.62120114

DMLK 2.70282355

DMLL 3.72373932

DMLM 4.84753687

DMLN 2.53374470

DMLP 2.26418196

DMLQ 2.92104833

DMLR 2.62306575

DMLS 2.63645996

DMLT 2.88795473

DMLV 3.23854879

DMLW 2.74184060

DMLY 2.91005564

DMMA 3.24580509

DMMC 3.18684657

DMMD 2.59529575

DMME 2.93709972

DMMF 3.55231451

DMMG 2.38060305

DMMH 2.94539845

DMMI 4.01964191

DMMK 3.00608880

DMML 4.13632702

DMMM 5.20638485

DMMN 2.86079691

DMMP 2.58423886

DMMQ 3.20667601

DMMR 2.91758255

DMMS 2.92174316

DMMT 3.21045116

DMMV 3.61283223

DMMW 3.24885674

DMMY 3.24007504

DMNA 5.23808868

DMNC 5.21750464

DMND 4.41994790

DMNE 4.87157328

DMNF 5.93291965

DMNG 4.24319437

DMNH 4.83501600

DMNI 6.44226105

DMNK 4.98032503

DMNL 6.65204784

DMNM 8.04284784

DMNN 4.72165079

DMNP 4.45506602

DMNQ 5.27933192

DMNR 4.85754911

DMNS 4.91418167

DMNT 5.28044507

DMNV 5.95748835

DMNW 5.17264559

DMNY 5.33598252

DMPA 4.33972046

DMPC 3.96709666

DMPD 3.51909844

DMPE 3.93078991

DMPF 4.77018401

DMPG 3.25130673

DMPH 3.92666672

DMPI 5.24672681

DMPK 4.00130095

DMPL 5.48917215

DMPM 6.65733752

DMPN 3.75248817

DMPP 3.49335442

DMPQ 4.33489558

DMPR 3.99706650

DMPS 4.02807717

DMPT 4.29411933

DMPV 4.86595027

DMPW 4.17059819

DMPY 4.24935460

DMQA 4.69225961

DMQC 4.67986056

DMQD 3.89197212

DMQE 4.21351365

DMQF 5.25870158

DMQG 3.78127903

DMQH 4.27952827

DMQI 5.77880162

DMQK 4.31760560

DMQL 5.94181303

DMQM 7.07492095

DMQN 4.10957007

DMQP 3.84463609

DMQQ 4.58959215

DMQR 4.34927714

DMQS 4.29652244

DMQT 4.66210827

DMQV 5.30338216

DMQW 4.64118457

DMQY 4.67921188

DMRA 4.25622286

DMRC 4.18582734

DMRD 3.37022206

DMRE 3.72831719

DMRF 4.72217345

DMRG 3.27238422

DMRH 3.81904098

DMRI 5.15456662

DMRK 3.80152349

DMRL 5.35329253

DMRM 6.39767921

DMRN 3.63233780

DMRP 3.38527654

DMRQ 4.08620926

DMRR 3.81807677

DMRS 3.85536796

DMRT 4.16899186

DMRV 4.80486459

DMRW 4.08382004

DMRY 4.16974185

DMSA 4.68462615

DMSC 4.61642911

DMSD 4.00641739

DMSE 4.32057062

DMSF 5.27087258

DMSG 3.71838012

DMSH 4.28164391

DMSI 5.70698179

DMSK 4.39821063

DMSL 5.95043843

DMSM 7.14868048

DMSN 4.14375417

DMSP 3.92246735

DMSQ 4.65021199

DMSR 4.33779936

DMSS 4.31944182

DMST 4.65003597

DMSV 5.27807621

DMSW 4.57188016

DMSY 4.72196100

DMTA 4.29156548

DMTC 4.23366358

DMTD 3.61924218

DMTE 3.94282782

DMTF 4.84147483

DMTG 3.36945893

DMTH 3.91914158

DMTI 5.25843075

DMTK 4.01372895

DMTL 5.49705886

DMTM 6.65174852

DMTN 3.79805211

DMTP 3.58093932

DMTQ 4.29078717

DMTR 3.94677203

DMTS 3.93188906

DMTT 4.21644645

DMTV 4.81236939

DMTW 4.21042215

DMTY 4.34036592

DMVA 2.95550690

DMVC 2.82784039

DMVD 2.44274956

DMVE 2.79453272

DMVF 3.38205302

DMVG 2.14546942

DMVH 2.69247131

DMVI 3.71892873

DMVK 2.82601196

DMVL 3.94202358

DMVM 5.05884384

DMVN 2.62175756

DMVP 2.36618480

DMVQ 3.03306910

DMVR 2.75276289

DMVS 2.75303094

DMVT 2.95867431

DMVV 3.31225909

DMVW 2.90109989

DMVY 3.03981141

DMWA 2.97907513

DMWC 2.55775535

DMWD 2.19909764

DMWE 2.51590751

DMWF 3.16885329

DMWG 2.13762172

DMWH 2.58040447

DMWI 3.64900444

DMWK 2.67969090

DMWL 3.75773281

DMWM 4.95294179

DMWN 2.46429807

DMWP 2.11684355

DMWQ 2.95742923

DMWR 2.55234104

DMWS 2.62263650

DMWT 2.98415753

DMWV 3.34087533

DMWW 2.68186091

DMWY 2.80225058

DMYA 3.26887855

DMYC 3.24274106

DMYD 2.57878101

DMYE 2.86917422

DMYF 3.74813007

DMYG 2.55398938

DMYH 2.90277388

DMYI 4.22578959

DMYK 3.06374972

DMYL 4.30775117

DMYM 5.50984960

DMYN 2.92705020

DMYP 2.55345038

DMYQ 3.28005196

DMYR 2.86470494

DMYS 3.02676529

DMYT 3.35896139

DMYV 3.78941558

DMYW 3.02403764

DMYY 3.34346750

DNAA 4.12657639

DNAC 3.35259663

DNAD 5.39334009

DNAE 4.77474492

DNAF 2.78821944

DNAG 4.59138790

DNAH 4.66944538

DNAI 2.79228610

DNAK 4.64188821

DNAL 2.93602214

DNAM 3.28529222

DNAN 6.43478602

DNAP 4.08187193

DNAQ 4.65726905

DNAR 4.41283035

DNAS 4.77009057

DNAT 4.48161062

DNAV 3.04084539

DNAW 2.81221874

DNAY 3.39929620

DNCA 2.79875256

DNCC 0.72270484

DNCD 4.08144980

DNCE 3.60383661

DNCF 1.90263004

DNCG 3.01402064

DNCH 3.19606749

DNCI 1.93490898

DNCK 3.57474053

DNCL 1.92028671

DNCM 2.41164576

DNCN 5.40509969

DNCP 2.63135753

DNCQ 3.47269161

DNCR 3.26652541

DNCS 3.65120908

DNCT 3.37480791

DNCV 2.10167082

DNCW 1.75950497

DNCY 2.55553943

DNDA 6.75448385

DNDC 6.32517163

DNDD 8.16846827

DNDE 7.15952239

DNDF 5.62121061

DNDG 7.40364428

DNDH 7.65157238

DNDI 5.70871514

DNDK 7.27262154

DNDL 5.68034939

DNDM 6.15754786

DNDN 11.81836776

DNDP 6.41683860

DNDQ 7.30340324

DNDR 6.93783388

DNDS 7.68635614

DNDT 7.33744592

DNDV 5.83906163

DNDW 5.42629318

DNDY 6.28452487

DNEA 5.42546049

DNEC 4.86317143

DNED 6.62520691

DNEE 5.94656115

DNEF 4.30834096

DNEG 5.98492961

DNEH 6.08852725

DNEI 4.40486732

DNEK 5.94472391

DNEL 4.40300040

DNEM 4.81043936

DNEN 8.21469187

DNEP 5.24170857

DNEQ 5.95744791

DNER 5.59996946

DNES 6.21941518

DNET 5.87047786

DNEV 4.53155615

DNEW 4.09799371

DNEY 4.90918757

DNFA 2.41069485

DNFC 1.82023263

DNFD 3.76679355

DNFE 3.24509062

DNFF 1.54297695

DNFG 2.93937189

DNFH 3.13863081

DNFI 1.63484109

DNFK 3.26224607

DNFL 1.55322761

DNFM 1.94967220

DNFN 4.77949535

DNFP 2.55642075

DNFQ 3.15761849

DNFR 2.85206910

DNFS 3.27460235

DNFT 2.95689564

DNFV 1.67258016

DNFW 1.53754702

DNFY 2.16386900

DNGA 4.33323336

DNGC 3.42180454

DNGD 5.63544257

DNGE 5.07181787

DNGF 3.14184702

DNGG 4.57114881

DNGH 4.83164959

DNGI 3.30331572

DNGK 5.00578989

DNGL 3.30863335

DNGM 3.71349542

DNGN 6.89471455

DNGP 4.26341835

DNGQ 5.01448159

DNGR 4.65726177

DNGS 5.11606305

DNGT 4.81279164

DNGV 3.45081448

DNGW 3.07574405

DNGY 3.75545075

DNHA 4.24036006

DNHC 3.25963772

DNHD 5.46292126

DNHE 4.86297263

DNHF 3.23608814

DNHG 4.64345985

DNHH 4.84556686

DNHI 3.26423763

DNHK 4.85251904

DNHL 3.30848636

DNHM 3.65746384

DNHN 6.76600401

DNHP 4.16040664

DNHQ 4.81408004

DNHR 4.51658419

DNHS 4.98220152

DNHT 4.65067700

DNHV 3.41714500

DNHW 3.23400191

DNHY 3.82076578

DNIA 2.46505483

DNIC 2.02856333

DNID 3.92734146

DNIE 3.43605228

DNIF 1.66906535

DNIG 3.08589464

DNIH 3.23754715

DNII 1.64197046

DNIK 3.41450637

DNIL 1.67993277

DNIM 1.97729675

DNIN 4.85168843

DNIP 2.73038795

DNIQ 3.29708581

DNIR 3.00955462

DNIS 3.40534788

DNIT 3.05586269

DNIV 1.70729910

DNIW 1.68767137

DNIY 2.32695679

DNKA 4.50921583

DNKC 3.98503208

DNKD 5.75908718

DNKE 5.16070375

DNKF 3.56162694

DNKG 5.10892165

DNKH 5.10299661

DNKI 3.66112761

DNKK 5.12210999

DNKL 3.65223634

DNKM 3.99305190

DNKN 6.72934647

DNKP 4.46554054

DNKQ 4.99952629

DNKR 4.72762204

DNKS 5.24965185

DNKT 4.93986505

DNKV 3.74051326

DNKW 3.42895547

DNKY 4.18642512

DNLA 2.60459139

DNLC 2.11714245

DNLD 3.94452936

DNLE 3.44269914

DNLF 1.63046575

DNLG 3.16991731

DNLH 3.31973370

DNLI 1.70341734

DNLK 3.42192523

DNLL 1.63284016

DNLM 2.17658774

DNLN 4.93167964

DNLP 2.78854618

DNLQ 3.32857276

DNLR 3.05044486

DNLS 3.48166546

DNLT 3.14492379

DNLV 1.77924263

DNLW 1.65502933

DNLY 2.30428595

DNMA 2.88268880

DNMC 2.44795860

DNMD 4.29194673

DNME 3.75883827

DNMF 2.03325645

DNMG 3.45803847

DNMH 3.57194022

DNMI 1.98160012

DNMK 3.62812306

DNML 2.03084845

DNMM 2.43458226

DNMN 5.18919042

DNMP 3.08295047

DNMQ 3.59575212

DNMR 3.33327162

DNMS 3.68136000

DNMT 3.35697406

DNMV 2.09187464

DNMW 2.16204547

DNMY 2.66953191

DNNA 5.25540626

DNNC 4.73015544

DNND 6.69882084

DNNE 6.01151315

DNNF 4.26480837

DNNG 5.84644772

DNNH 5.92067084

DNNI 4.35831892

DNNK 5.96179674

DNNL 4.36811381

DNNM 4.67236714

DNNN 7.98506121

DNNP 5.12775353

DNNQ 5.89512814

DNNR 5.49475672

DNNS 6.10336568

DNNT 5.79113435

DNNV 4.49410780

DNNW 4.12890304

DNNY 4.89982077

DNPA 4.35925448

DNPC 3.41914070

DNPD 5.49370085

DNPE 4.94925933

DNPF 3.16430642

DNPG 4.66081444

DNPH 4.81464188

DNPI 3.30190485

DNPK 4.87406060

DNPL 3.34525886

DNPM 3.66370917

DNPN 6.55512603

DNPP 4.20038327

DNPQ 4.85290814

DNPR 4.56042915

DNPS 5.01415987

DNPT 4.72167819

DNPV 3.45069269

DNPW 3.06061607

DNPY 3.71733636

DNQA 4.64583778

DNQC 4.00734140

DNQD 5.85919938

DNQE 5.21723539

DNQF 3.57825025

DNQG 5.18928393

DNQH 5.21995824

DNQI 3.68023815

DNQK 5.16500854

DNQL 3.68606573

DNQM 3.97538778

DNQN 6.89175752

DNQP 4.56305710

DNQQ 5.16474868

DNQR 4.87727590

DNQS 5.33771031

DNQT 5.01581993

DNQV 3.78569923

DNQW 3.55003623

DNQY 4.18908560

DNRA 4.16972945

DNRC 3.56354856

DNRD 5.27754827

DNRE 4.68319032

DNRF 3.07861505

DNRG 4.64945748

DNRH 4.64587038

DNRI 3.14981820

DNRK 4.57474466

DNRL 3.16412634

DNRM 3.53181839

DNRN 6.30422925

DNRP 4.05208503

DNRQ 4.58972905

DNRR 4.38094581

DNRS 4.81000261

DNRT 4.49547043

DNRV 3.30947807

DNRW 3.03075443

DNRY 3.60439014

DNSA 4.69650957

DNSC 4.03597051

DNSD 5.99441866

DNSE 5.38786460

DNSF 3.67888984

DNSG 5.18458958

DNSH 5.24757411

DNSI 3.69731836

DNSK 5.30996614

DNSL 3.74857741

DNSM 4.01674480

DNSN 7.06677577

DNSP 4.65029424

DNSQ 5.22947707

DNSR 4.96840517

DNSS 5.44833115

DNST 5.14440211

DNSV 3.84947533

DNSW 3.51337426

DNSY 4.23199585

DNTA 4.31088626

DNTC 3.63489310

DNTD 5.60836305

DNTE 5.01350879

DNTF 3.22479604

DNTG 4.79880350

DNTH 4.83412856

DNTI 3.26365045

DNTK 4.91929792

DNTL 3.29289846

DNTM 3.59668787

DNTN 6.59122951

DNTP 4.28510198

DNTQ 4.86590205

DNTR 4.57099559

DNTS 5.03580039

DNTT 4.75398182

DNTV 3.41740495

DNTW 3.15747577

DNTY 3.79553122

DNVA 2.80052561

DNVC 2.17599715

DNVD 4.19635961

DNVE 3.67288402

DNVF 1.80282172

DNVG 3.35706955

DNVH 3.47872575

DNVI 1.82038410

DNVK 3.60427111

DNVL 1.81724446

DNVM 2.23115451

DNVN 5.16764469

DNVP 2.97971896

DNVQ 3.56232187

DNVR 3.26180244

DNVS 3.65005042

DNVT 3.33790344

DNVV 1.89428360

DNVW 1.87095765

DNVY 2.44936872

DNWA 2.64823042

DNWC 1.84801373

DNWD 3.80860998

DNWE 3.29888521

DNWF 1.59404125

DNWG 3.01284260

DNWH 3.13744939

DNWI 1.78843792

DNWK 3.25647204

DNWL 1.78592471

DNWM 2.23426705

DNWN 4.83492987

DNWP 2.49824981

DNWQ 3.23436623

DNWR 3.01252442

DNWS 3.35371082

DNWT 3.08177084

DNWV 1.98597643

DNWW 1.61900954

DNWY 2.10339728

DNYA 3.08037383

DNYC 2.59189313

DNYD 4.38223738

DNYE 3.80838666

DNYF 2.27805886

DNYG 3.59349973

DNYH 3.81535529

DNYI 2.35098873

DNYK 3.84641385

DNYL 2.25468556

DNYM 2.64346786

DNYN 5.49365999

DNYP 3.12499487

DNYQ 3.73380907

DNYR 3.38063087

DNYS 3.92685679

DNYT 3.61971053

DNYV 2.39309055

DNYW 2.08317679

DNYY 2.91710894

DPAA 4.21415008

DPAC 2.86665575

DPAD 4.47757185

DPAE 4.36085515

DPAF 2.78581789

DPAG 4.02026843

DPAH 3.65277897

DPAI 2.99688578

DPAK 4.03202040

DPAL 3.09444741

DPAM 2.95254986

DPAN 3.87061476

DPAP 7.38451551

DPAQ 3.89675683

DPAR 3.91344995

DPAS 4.32755256

DPAT 4.02923037

DPAV 3.38724020

DPAW 2.85602944

DPAY 2.92009689

DPCA 3.22352246

DPCC 0.06447194

DPCD 3.29871010

DPCE 3.48636640

DPCF 1.87826924

DPCG 2.70493037

DPCH 2.25812145

DPCI 2.20600407

DPCK 3.21647899

DPCL 2.14968250

DPCM 2.33283718

DPCN 3.04700149

DPCP 6.28937173

DPCQ 2.96290399

DPCR 2.84361524

DPCS 3.40593935

DPCT 3.12805469

DPCV 2.54297359

DPCW 1.73514418

DPCY 2.19414364

DPDA 7.20558038

DPDC 5.83465234

DPDD 6.79055246

DPDE 6.87944362

DPDF 5.59684982

DPDG 6.47948232

DPDH 6.28659139

DPDI 6.08904989

DPDK 6.63694333

DPDL 6.03578891

DPDM 5.91577730

DPDN 6.41683860

DPDP 12.57298272

DPDQ 6.52369037

DPDR 6.31631164

DPDS 7.16675696

DPDT 6.74426841

DPDV 6.39102545

DPDW 5.49728026

DPDY 5.65438103

DPEA 5.52329830

DPEC 4.32667444

DPED 5.55866002

DPEE 5.44985754

DPEF 4.17268652

DPEG 5.24274916

DPEH 4.84679429

DPEI 4.51078572

DPEK 5.19980459

DPEL 4.44299582

DPEM 4.37907839

DPEN 5.18070238

DPEP 9.07634889

DPEQ 5.07663294

DPER 4.86301349

DPES 5.68572474

DPET 5.30065327

DPEV 4.82660825

DPEW 4.04561854

DPEY 4.21827673

DPFA 2.73134576

DPFC 1.54794432

DPFD 3.03616396

DPFE 3.02292304

DPFF 1.50231434

DPFG 2.52951787

DPFH 2.28876775

DPFI 1.87351470

DPFK 2.91072053

DPFL 1.77502740

DPFM 1.81839619

DPFN 2.77757195

DPFP 5.57036931

DPFQ 2.68645763

DPFR 2.48090554

DPFS 2.98299489

DPFT 2.70223722

DPFV 2.04414803

DPFW 1.55898992

DPFY 1.80390517

DPGA 4.70495190

DPGC 3.05640683

DPGD 4.77071530

DPGE 4.84949227

DPGF 3.26147549

DPGG 4.01069334

DPGH 4.02873981

DPGI 3.65619039

DPGK 4.65723082

DPGL 3.63916978

DPGM 3.59564800

DPGN 4.35772564

DPGP 7.69298439

DPGQ 4.45499606

DPGR 4.33892462

DPGS 4.79554974

DPGT 4.50454603

DPGV 3.95055784

DPGW 3.18402102

DPGY 3.38377654

DPHA 4.50313653

DPHC 2.84533041

DPHD 4.60097533

DPHE 4.58134556

DPHF 3.20194632

DPHG 4.15747798

DPHH 3.77002734

DPHI 3.58357555

DPHK 4.40745199

DPHL 3.52546489

DPHM 3.43079487

DPHN 4.16140312

DPHP 7.74639114

DPHQ 4.21333913

DPHR 4.13732617

DPHS 4.64783366

DPHT 4.28759966

DPHV 3.80782171

DPHW 3.28364169

DPHY 3.28944499

DPIA 2.62670602

DPIC 1.80716569

DPID 3.15901593

DPIE 3.09674231

DPIF 1.68573182

DPIG 2.61267147

DPIH 2.49577356

DPII 1.86336608

DPIK 2.97510808

DPIL 1.77586621

DPIM 1.77784925

DPIN 2.92001710

DPIP 5.72827498

DPIQ 2.69814165

DPIR 2.52924838

DPIS 3.06060281

DPIT 2.69746412

DPIV 1.97623053

DPIW 1.61891646

DPIY 1.91302918

DPKA 4.56292250

DPKC 3.50362626

DPKD 4.72343712

DPKE 4.58494588

DPKF 3.47453038

DPKG 4.34833151

DPKH 4.12114055

DPKI 3.77153537

DPKK 4.56780516

DPKL 3.65367154

DPKM 3.56103313

DPKN 4.52176385

DPKP 7.72603785

DPKQ 4.22579160

DPKR 4.10551259

DPKS 4.76673406

DPKT 4.43251854

DPKV 3.95938209

DPKW 3.40459467

DPKY 3.63398688

DPLA 2.78599234

DPLC 1.78697323

DPLD 3.22949662

DPLE 3.10059805

DPLF 1.65315353

DPLG 2.72257026

DPLH 2.46434907

DPLI 1.89168600

DPLK 2.98252693

DPLL 1.82851174

DPLM 1.84312288

DPLN 2.89339887

DPLP 5.79115243

DPLQ 2.72860463

DPLR 2.59758430

DPLS 3.14495708

DPLT 2.79981309

DPLV 2.08293603

DPLW 1.58627442

DPLY 1.88601094

DPMA 2.99453239

DPMC 2.07567450

DPMD 3.40551385

DPME 3.34319609

DPMF 1.90935998

DPMG 2.94689557

DPMH 2.72225796

DPMI 2.16504420

DPMK 3.13744426

DPML 2.08272324

DPMM 2.06733375

DPMN 3.00584010

DPMP 6.15508736

DPMQ 2.87205081

DPMR 2.80641048

DPMS 3.26107053

DPMT 2.97004319

DPMV 2.39235353

DPMW 2.07057048

DPMY 2.18326701

DPNA 5.48477213

DPNC 4.20669288

DPND 5.49320040

DPNE 5.54245946

DPNF 4.17102617

DPNG 4.99618760

DPNH 4.88425162

DPNI 4.61911534

DPNK 5.38345439

DPNL 4.54041623

DPNM 4.38406144

DPNN 5.16705943

DPNP 8.96785991

DPNQ 5.16809140

DPNR 4.90882324

DPNS 5.61995303

DPNT 5.25640037

DPNV 4.81309177

DPNW 4.07236184

DPNY 4.29402917

DPPA 4.83215019

DPPC 3.12888584

DPPD 4.77105683

DPPE 4.81652237

DPPF 3.33176323

DPPG 4.23049725

DPPH 4.09708789

DPPI 3.73501682

DPPK 4.60834331

DPPL 3.74131876

DPPM 3.60211547

DPPN 4.34973515

DPPP 7.36368644

DPPQ 4.44751052

DPPR 4.28915361

DPPS 4.76245435

DPPT 4.48730870

DPPV 4.05376254

DPPW 3.33043838

DPPY 3.39801451

DPQA 4.67748737

DPQC 3.47954799

DPQD 4.82709370

DPQE 4.66768177

DPQF 3.42186106

DPQG 4.51008600

DPQH 4.12650492

DPQI 3.74974428

DPQK 4.52406149

DPQL 3.69934259

DPQM 3.54056221

DPQN 4.45607683

DPQP 7.88809400

DPQQ 4.31563591

DPQR 4.22214892

DPQS 4.81076354

DPQT 4.49702361

DPQV 4.01861357

DPQW 3.51007857

DPQY 3.51799913

DPRA 4.31844323

DPRC 3.13822545

DPRD 4.39447002

DPRE 4.23478721

DPRF 3.08550519

DPRG 4.11311363

DPRH 3.77143909

DPRI 3.35269109

DPRK 4.05939751

DPRL 3.29816183

DPRM 3.26081762

DPRN 4.00632417

DPRP 7.25153148

DPRQ 3.87902873

DPRR 3.89807085

DPRS 4.38769074

DPRT 4.10683525

DPRV 3.62996693

DPRW 3.07109933

DPRY 3.09460252

DPSA 4.91138065

DPSC 3.58276642

DPSD 5.05002116

DPSE 5.00993928

DPSF 3.60583955

DPSG 4.52899342

DPSH 4.25973918

DPSI 3.94230574

DPSK 4.78946400

DPSL 3.94798368

DPSM 3.79712771

DPSN 4.64459654

DPSP 7.98122698

DPSQ 4.55243946

DPSR 4.41507612

DPSS 4.99704297

DPST 4.65896090

DPSV 4.19946021

DPSW 3.51960178

DPSY 3.70905686

DPTA 4.52099072

DPTC 3.24042067

DPTD 4.68839029

DPTE 4.61690348

DPTF 3.16578910

DPTG 4.17310438

DPTH 3.90630511

DPTI 3.49082842

DPTK 4.39962631

DPTL 3.49390533

DPTM 3.35551568

DPTN 4.26963578

DPTP 7.50766596

DPTQ 4.17806673

DPTR 4.06607190

DPTS 4.60207671

DPTT 4.28505757

DPTV 3.75310301

DPTW 3.17575931

DPTY 3.27711642

DPVA 3.04876937

DPVC 1.95000250

DPVD 3.43503322

DPVE 3.37790722

DPVF 1.85491299

DPVG 2.87913665

DPVH 2.66921583

DPVI 2.01622151

DPVK 3.17256720

DPVL 2.02966718

DPVM 2.02357189

DPVN 3.02182038

DPVP 6.03000687

DPVQ 2.97592497

DPVR 2.84749512

DPVS 3.28066184

DPVT 2.95961261

DPVV 2.26320750

DPVW 1.84659685

DPVY 2.05209590

DPWA 2.90477045

DPWC 1.63100786

DPWD 3.07205149

DPWE 3.05213199

DPWF 1.73960545

DPWG 2.66655371

DPWH 2.37612300

DPWI 1.99234611

DPWK 2.89837385

DPWL 1.89721902

DPWM 2.07866172

DPWN 2.70747558

DPWP 5.59134660

DPWQ 2.80119988

DPWR 2.65954087

DPWS 3.05434298

DPWT 2.78177560

DPWV 2.19481481

DPWW 1.59464874

DPWY 1.91857181

DPYA 3.27174173

DPYC 2.28122815

DPYD 3.50880063

DPYE 3.51456519

DPYF 2.17426360

DPYG 3.09630526

DPYH 2.85049550

DPYI 2.53962165

DPYK 3.42876835

DPYL 2.41633675

DPYM 2.42355625

DPYN 3.34319042

DPYP 6.29867914

DPYQ 3.15239177

DPYR 2.93138479

DPYS 3.52918562

DPYT 3.25974670

DPYV 2.68209584

DPYW 2.09729014

DPYY 2.37682728

DQAA 4.48715292

DQAC 3.09633017

DQAD 4.98624215

DQAE 5.37570178

DQAF 2.87099813

DQAG 4.05840193

DQAH 4.58857850

DQAI 3.15214879

DQAK 5.04314199

DQAL 3.45225831

DQAM 3.80781305

DQAN 4.62595288

DQAP 4.11890267

DQAQ 6.09698343

DQAR 5.00198685

DQAS 4.53740476

DQAT 4.42789410

DQAV 3.41295369

DQAW 3.07260679

DQAY 3.37915148

DQCA 2.86017947

DQCC 0.45495888

DQCD 3.55778354

DQCE 4.03214305

DQCF 1.76333245

DQCG 2.35037583

DQCH 2.94092157

DQCI 2.09551318

DQCK 3.81196601

DQCL 2.24042073

DQCM 2.80286288

DQCN 3.38127931

DQCP 2.52007431

DQCQ 4.76125556

DQCR 3.58439639

DQCS 3.23005321

DQCT 3.12859511

DQCV 2.25031764

DQCW 2.07672152

DQCY 2.40618817

DQDA 6.96392159

DQDC 5.83039975

DQDD 7.18141959

DQDE 7.86127909

DQDF 5.56152028

DQDG 6.45088992

DQDH 7.53785074

DQDI 6.00590657

DQDK 7.68622357

DQDL 6.28536896

DQDM 6.90262837

DQDN 7.30340324

DQDP 6.52369037

DQDQ 11.14872166

DQDR 7.51233183

DQDS 7.08117550

DQDT 6.97915782

DQDV 6.18474173

DQDW 5.67394550

DQDY 6.06805941

DQEA 5.75234494

DQEC 4.63814396

DQED 6.11281359

DQEE 6.58790149

DQEF 4.42003556

DQEG 5.35483623

DQEH 6.12382685

DQEI 4.82045089

DQEK 6.41451750

DQEL 5.04714981

DQEM 5.47772889

DQEN 6.13483459

DQEP 5.29589071

DQEQ 7.81690221

DQER 6.23815655

DQES 5.91872450

DQET 5.78959115

DQEV 4.95831360

DQEW 4.42901606

DQEY 4.91481097

DQFA 2.52543531

DQFC 1.63594758

DQFD 3.21762016

DQFE 3.60670128

DQFF 1.45281408

DQFG 2.31307125

DQFH 2.93972373

DQFI 1.84980758

DQFK 3.50058864

DQFL 1.86469371

DQFM 2.40389912

DQFN 3.16618282

DQFP 2.47119983

DQFQ 4.29158936

DQFR 3.21997885

DQFS 2.93724166

DQFT 2.77052225

DQFV 1.87887956

DQFW 1.60392446

DQFY 2.04148479

DQGA 4.52587393

DQGC 3.09907272

DQGD 4.99256055

DQGE 5.55511755

DQGF 3.14304510

DQGG 3.89656387

DQGH 4.73348187

DQGI 3.53699157

DQGK 5.31564526

DQGL 3.71158344

DQGM 4.15884598

DQGN 4.82318471

DQGP 4.20407154

DQGQ 6.50337625

DQGR 5.13460013

DQGS 4.73231985

DQGT 4.65978726

DQGV 3.77936515

DQGW 3.24189462

DQGY 3.67692448

DQHA 4.44264752

DQHC 2.94208615

DQHD 4.90708727

DQHE 5.41444967

DQHF 3.26960094

DQHG 4.01326601

DQHH 4.61856467

DQHI 3.65545476

DQHK 5.18749205

DQHL 3.81551193

DQHM 4.14010400

DQHN 4.77492042

DQHP 4.14696541

DQHQ 6.32975165

DQHR 5.01101698

DQHS 4.62233194

DQHT 4.49312182

DQHV 3.76147574

DQHW 3.39965933

DQHY 3.71851876

DQIA 2.61556463

DQIC 1.80770611

DQID 3.42332142

DQIE 3.83965313

DQIF 1.67272571

DQIG 2.46937612

DQIH 3.17091370

DQII 1.91513182

DQIK 3.69287016

DQIL 2.02007571

DQIM 2.43890320

DQIN 3.42120373

DQIP 2.65900189

DQIQ 4.45414401

DQIR 3.39960781

DQIS 3.07649119

DQIT 2.90371822

DQIV 1.93535102

DQIW 1.82891024

DQIY 2.26971341

DQKA 4.71534910

DQKC 3.71149165

DQKD 5.26654457

DQKE 5.62699393

DQKF 3.63119001

DQKG 4.43460181

DQKH 5.00542790

DQKI 3.99015990

DQKK 5.43707162

DQKL 4.07971206

DQKM 4.44271874

DQKN 5.18019830

DQKP 4.39865144

DQKQ 6.25421965

DQKR 5.14407646

DQKS 4.95630831

DQKT 4.82789453

DQKV 4.02943950

DQKW 3.55569477

DQKY 4.14048193

DQLA 2.84621479

DQLC 1.86551616

DQLD 3.51891599

DQLE 3.89348082

DQLF 1.66904192

DQLG 2.63548178

DQLH 3.25697766

DQLI 1.98533582

DQLK 3.75358551

DQLL 2.05911744

DQLM 2.56264315

DQLN 3.45065232

DQLP 2.75301238

DQLQ 4.53935171

DQLR 3.50019830

DQLS 3.21242297

DQLT 3.05106764

DQLV 2.04811451

DQLW 1.79626820

DQLY 2.27406780

DQMA 3.05863794

DQMC 2.20174581

DQMD 3.79107920

DQME 4.19495193

DQMF 2.00943608

DQMG 2.86943348

DQMH 3.51926498

DQMI 2.26746424

DQMK 3.95614636

DQML 2.42206558

DQMM 2.81884663

DQMN 3.65079687

DQMP 3.00198619

DQMQ 4.84008859

DQMR 3.76818134

DQMS 3.39219506

DQMT 3.22500488

DQMV 2.34558824

DQMW 2.20235544

DQMY 2.56441143

DQNA 5.37203842

DQNC 4.34473683

DQND 5.95398181

DQNE 6.50695914

DQNF 4.23775687

DQNG 4.99115776

DQNH 5.80294029

DQNI 4.68105630

DQNK 6.26966213

DQNL 4.80505460

DQNM 5.25010082

DQNN 5.87073185

DQNP 5.06776342

DQNQ 7.50418642

DQNR 5.94991776

DQNS 5.66329188

DQNT 5.56151005

DQNV 4.78451526

DQNW 4.20744438

DQNY 4.78100388

DQPA 4.62042482

DQPC 3.12942626

DQPD 4.95083000

DQPE 5.37695995

DQPF 3.21150551

DQPG 4.03539464

DQPH 4.72793379

DQPI 3.58268269

DQPK 5.18703570

DQPL 3.77374324

DQPM 4.11345237

DQPN 4.74965981

DQPP 4.15826208

DQPQ 6.21954724

DQPR 5.03178011

DQPS 4.69347333

DQPT 4.56585163

DQPV 3.78906428

DQPW 3.28833446

DQPY 3.72048303

DQQA 4.94939806

DQQC 3.77964869

DQQD 5.47231842

DQQE 5.82120076

DQQF 3.69043657

DQQG 4.60474860

DQQH 5.23454373

DQQI 4.06233389

DQQK 5.57839704

DQQL 4.21796063

DQQM 4.58161779

DQQN 5.28403779

DQQP 4.57421286

DQQQ 6.53981838

DQQR 5.41824015

DQQS 5.09577268

DQQT 5.02776478

DQQV 4.17187825

DQQW 3.78147291

DQQY 4.16108955

DQRA 4.47530935

DQRC 3.31024634

DQRD 4.85664139

DQRE 5.22950900

DQRF 3.18076427

DQRG 4.09575236

DQRH 4.59591351

DQRI 3.50124173

DQRK 4.91657376

DQRL 3.65900702

DQRM 4.02737217

DQRN 4.63292087

DQRP 4.02561993

DQRQ 5.85236743

DQRR 4.87231031

DQRS 4.54686867

DQRT 4.44153007

DQRV 3.69261285

DQRW 3.27217273

DQRY 3.57626963

DQSA 4.90313031

DQSC 3.80569926

DQSD 5.47594965

DQSE 5.91581459

DQSF 3.66778052

DQSG 4.51404007

DQSH 5.16366851

DQSI 4.00651991

DQSK 5.63099308

DQSL 4.19775271

DQSM 4.52203369

DQSN 5.25327795

DQSP 4.59704775

DQSQ 6.64645033

DQSR 5.41628616

DQSS 5.10301233

DQST 4.98142942

DQSV 4.14932649

DQSW 3.76115030

DQSY 4.16296290

DQTA 4.51871795

DQTC 3.37102163

DQTD 5.08431125

DQTE 5.51825486

DQTF 3.21557304

DQTG 4.14705059

DQTH 4.74390696

DQTI 3.56023070

DQTK 5.22769953

DQTL 3.73078226

DQTM 4.08206249

DQTN 4.88130537

DQTP 4.27549547

DQTQ 6.25921903

DQTR 5.02153942

DQTS 4.69944770

DQTT 4.61031271

DQTV 3.69987218

DQTW 3.29715413

DQTY 3.73207388

DQVA 2.99256728

DQVC 1.97438966

DQVD 3.69547029

DQVE 4.09851117

DQVF 1.83671684

DQVG 2.75163788

DQVH 3.39029630

DQVI 2.05575606

DQVK 3.90392099

DQVL 2.20846159

DQVM 2.63882215

DQVN 3.55774127

DQVP 2.94168472

DQVQ 4.77544064

DQVR 3.70822044

DQVS 3.33583752

DQVT 3.17578808

DQVV 2.17192721

DQVW 2.09224977

DQVY 2.40130080

DQWA 2.77776082

DQWC 1.63154828

DQWD 3.23224858

DQWE 3.72897523

DQWF 1.57022087

DQWG 2.43968364

DQWH 3.00469464

DQWI 1.88326204

DQWK 3.45055670

DQWL 2.08809065

DQWM 2.59377531

DQWN 3.12305350

DQWP 2.42196202

DQWQ 4.38670281

DQWR 3.34234100

DQWS 2.99598971

DQWT 2.90942794

DQWV 2.06000338

DQWW 1.67765132

DQWY 2.11705161

DQYA 3.14237282

DQYC 2.28176857

DQYD 3.74210515

DQYE 4.18618327

DQYF 2.14732328

DQYG 2.94056975

DQYH 3.57923131

DQYI 2.54016208

DQYK 4.09013329

DQYL 2.60733910

DQYM 3.06981321

DQYN 3.79303825

DQYP 3.04228081

DQYQ 4.96028711

DQYR 3.74585279

DQYS 3.49557782

DQYT 3.39674936

DQYV 2.59044256

DQYW 2.17183115

DQYY 2.74442517

DRAA 4.35686014

DRAC 3.21467421

DRAD 4.51745764

DRAE 4.85527354

DRAF 2.82886541

DRAG 3.89056519

DRAH 4.55670193

DRAI 3.16760561

DRAK 5.37712010

DRAL 3.46320192

DRAM 3.56022799

DRAN 4.35423643

DRAP 4.02156461

DRAQ 5.01609905

DRAR 6.26543637

DRAS 4.32780450

DRAT 4.31146648

DRAV 3.41354420

DRAW 3.36151264

DRAY 3.46162839

DRCA 2.74753212

DRCC -0.01355912

DRCD 3.10825185

DRCE 3.50514258

DRCF 1.73189806

DRCG 2.21338164

DRCH 2.80249020

DRCI 2.09982197

DRCK 4.24105130

DRCL 2.26779111

DRCM 2.53264163

DRCN 3.14698792

DRCP 2.36088437

DRCQ 3.65635578

DRCR 4.98042161

DRCS 3.08254507

DRCT 3.06626671

DRCV 2.18930810

DRCW 2.03883787

DRCY 2.39242867

DRDA 6.70239373

DRDC 5.97006978

DRDD 6.47511050

DRDE 6.93424866

DRDF 5.47063636

DRDG 6.24506313

DRDH 7.24763991

DRDI 5.93921485

DRDK 8.32474311

DRDL 6.11735120

DRDM 6.32636179

DRDN 6.93783388

DRDP 6.31631164

DRDQ 7.51233183

DRDR 11.38849717

DRDS 6.82518843

DRDT 6.79518897

DRDV 6.04528149

DRDW 5.95918685

DRDY 6.09627791

DREA 5.50055705

DREC 4.68016112

DRED 5.45252608

DREE 5.80395657

DREF 4.32759797

DREG 5.08975829

DREH 5.84387039

DREI 4.76427381

DREK 6.84339868

DREL 4.88886798

DREM 5.08351893

DREN 5.74584307

DREP 5.08799257

DREQ 6.21281087

DRER 7.89222673

DRES 5.62761281

DRET 5.55824167

DREV 4.83978113

DREW 4.65194900

DREY 4.89983102

DRFA 2.41303958

DRFC 1.78161222

DRFD 2.79951193

DRFE 3.16081426

DRFF 1.43882206

DRFG 2.17139908

DRFH 2.88523117

DRFI 1.85236871

DRFK 3.86350706

DRFL 1.83877030

DRFM 2.12923196

DRFN 2.95620477

DRFP 2.34644339

DRFQ 3.35293389

DRFR 4.41707957

DRFS 2.74277625

DRFT 2.63756386

DRFV 1.88824390

DRFW 1.76957421

DRFY 2.14370916

DRGA 4.36560292

DRGC 3.18434644

DRGD 4.45728165

DRGE 4.96921930

DRGF 3.07851391

DRGG 3.71534763

DRGH 4.63909227

DRGI 3.54525838

DRGK 5.66133605

DRGL 3.66807814

DRGM 3.87003241

DRGN 4.54442603

DRGP 4.06866283

DRGQ 5.19562760

DRGR 6.60327598

DRGS 4.50170528

DRGT 4.52540946

DRGV 3.73990485

DRGW 3.49305048

DRGY 3.72712512

DRHA 4.37406013

DRHC 3.11410212

DRHD 4.49844590

DRHE 4.88478211

DRHF 3.23594189

DRHG 3.91980539

DRHH 4.51755580

DRHI 3.70776893

DRHK 5.59302459

DRHL 3.84598322

DRHM 3.92938376

DRHN 4.56042878

DRHP 4.07635319

DRHQ 5.14426497

DRHR 6.46358555

DRHS 4.51898415

DRHT 4.47634893

DRHV 3.77493605

DRHW 3.66656208

DRHY 3.82146742

DRIA 2.47321821

DRIC 1.81452612

DRID 2.97530108

DRIE 3.38185112

DRIF 1.63528105

DRIG 2.32177415

DRIH 3.07408964

DRII 1.98788861

DRIK 4.01395891

DRIL 1.97336050

DRIM 2.19898199

DRIN 3.14455622

DRIP 2.53432148

DRIQ 3.48951229

DRIR 4.54005093

DRIS 2.89937935

DRIT 2.78703301

DRIV 1.92676282

DRIW 2.00458746

DRIY 2.37731001

DRKA 4.49635904

DRKC 3.77955443

DRKD 4.73402107

DRKE 5.10455055

DRKF 3.61906242

DRKG 4.23318364

DRKH 4.88112348

DRKI 3.99888874

DRKK 5.67999842

DRKL 4.04007283

DRKM 4.19989075

DRKN 4.94896712

DRKP 4.23763877

DRKQ 5.22869948

DRKR 6.39679339

DRKS 4.74849967

DRKT 4.67604437

DRKV 3.97734943

DRKW 3.79848618

DRKY 4.20958688

DRLA 2.74047453

DRLC 1.93952540

DRLD 3.09291990

DRLE 3.46472241

DRLF 1.65266590

DRLG 2.47157869

DRLH 3.17324926

DRLI 2.01670222

DRLK 4.08691182

DRLL 2.08064189

DRLM 2.31825090

DRLN 3.17553227

DRLP 2.67017333

DRLQ 3.61067110

DRLR 4.62347958

DRLS 3.04262494

DRLT 2.94452187

DRLV 2.05233807

DRLW 2.00253374

DRLY 2.34912937

DRMA 2.88276926

DRMC 2.23018287

DRMD 3.30779333

DRME 3.68747331

DRMF 1.94335554

DRMG 2.71690727

DRMH 3.34542827

DRMI 2.28304870

DRMK 4.31833224

DRML 2.37576970

DRMM 2.53602534

DRMN 3.40227604

DRMP 2.84764565

DRMQ 3.79665679

DRMR 4.95945196

DRMS 3.16677142

DRMT 3.09150159

DRMV 2.30925505

DRMW 2.32695847

DRMY 2.71173454

DRNA 5.11895326

DRNC 4.41913752

DRND 5.34490343

DRNE 5.79026129

DRNF 4.18655998

DRNG 4.75654681

DRNH 5.63518107

DRNI 4.62340439

DRNK 6.66394899

DRNL 4.71065739

DRNM 4.91120486

DRNN 5.58734418

DRNP 4.87494911

DRNQ 6.06867781

DRNR 7.65579825

DRNS 5.41173122

DRNT 5.36928110

DRNV 4.66449592

DRNW 4.48778651

DRNY 4.81256944

DRPA 4.45202659

DRPC 3.15282774

DRPD 4.44160663

DRPE 4.83508789

DRPF 3.12799517

DRPG 3.86378335

DRPH 4.59936547

DRPI 3.53930521

DRPK 5.45346357

DRPL 3.71846211

DRPM 3.81056897

DRPN 4.45872562

DRPP 4.00315910

DRPQ 5.07342134

DRPR 6.35990212

DRPS 4.42834469

DRPT 4.42407875

DRPV 3.73123246

DRPW 3.48528833

DRPY 3.72923773

DRQA 4.74123028

DRQC 3.86476595

DRQD 4.90945229

DRQE 5.20055247

DRQF 3.60424846

DRQG 4.38349770

DRQH 5.04040349

DRQI 4.05087058

DRQK 5.88928597

DRQL 4.13593855

DRQM 4.29730047

DRQN 4.97195886

DRQP 4.40442545

DRQQ 5.45012529

DRQR 6.69498104

DRQS 4.84742560

DRQT 4.81727574

DRQV 4.07547009

DRQW 4.00445583

DRQY 4.18370125

DRRA 4.47479207

DRRC 3.42921209

DRRD 4.54908260

DRRE 4.90508313

DRRF 3.24327729

DRRG 4.05241749

DRRH 4.61123243

DRRI 3.61631064

DRRK 5.25664357

DRRL 3.75077866

DRRM 3.92611701

DRRN 4.49465888

DRRP 4.04622346

DRRQ 4.98556163

DRRR 6.02180238

DRRS 4.48692659

DRRT 4.45878859

DRRV 3.80364386

DRRW 3.64941540

DRRY 3.73411875

DRSA 4.65848270

DRSC 3.78257087

DRSD 4.88070292

DRSE 5.24788466

DRSF 3.58058603

DRSG 4.29532389

DRSH 5.01089186

DRSI 3.95443653

DRSK 5.95004343

DRSL 4.08343723

DRSM 4.22576201

DRSN 4.95772479

DRSP 4.41494342

DRSQ 5.42212172

DRSR 6.78446619

DRSS 4.82886218

DRST 4.78113078

DRSV 4.04797048

DRSW 3.95150878

DRSY 4.17349782

DRTA 4.30110357

DRTC 3.37621852

DRTD 4.52470838

DRTE 4.89974161

DRTF 3.12876903

DRTG 3.93082971

DRTH 4.63438146

DRTI 3.53706156

DRTK 5.53140673

DRTL 3.66015303

DRTM 3.77748973

DRTN 4.55454585

DRTP 4.08452682

DRTQ 5.06625434

DRTR 6.31417463

DRTS 4.44422185

DRTT 4.45359525

DRTV 3.65569113

DRTW 3.54654667

DRTY 3.74800892

DRVA 2.86202755

DRVC 2.02742034

DRVD 3.23551143

DRVE 3.59783088

DRVF 1.82386125

DRVG 2.59801606

DRVH 3.28867789

DRVI 2.06387647

DRVK 4.23094057

DRVL 2.21450190

DRVM 2.43646435

DRVN 3.28064329

DRVP 2.78090679

DRVQ 3.75926152

DRVR 4.87525873

DRVS 3.16359143

DRVT 3.06885821

DRVV 2.17132052

DRVW 2.23074038

DRVY 2.46100584

DRWA 2.76814724

DRWC 1.60360288

DRWD 2.94377563

DRWE 3.32958159

DRWF 1.60742815

DRWG 2.38237331

DRWH 2.99750780

DRWI 2.04092921

DRWK 3.87182787

DRWL 2.07765815

DRWM 2.46629423

DRWN 2.99033578

DRWP 2.47751093

DRWQ 3.56592909

DRWR 4.57181315

DRWS 2.92426261

DRWT 2.82365907

DRWV 2.09935912

DRWW 1.85103672

DRWY 2.15358614

DRYA 3.01641619

DRYC 2.34998913

DRYD 3.34853102

DRYE 3.75367518

DRYF 2.16216274

DRYG 2.77729863

DRYH 3.51644586

DRYI 2.60471099

DRYK 4.47204716

DRYL 2.58889314

DRYM 2.88561926

DRYN 3.58913666

DRYP 2.94472994

DRYQ 3.97426488

DRYR 5.02953456

DRYS 3.35071466

DRYT 3.25995395

DRYV 2.59056052

DRYW 2.49806804

DRYY 2.85175021

DSAA 4.71760589

DSAC 3.99886369

DSAD 4.96306354

DSAE 4.76388028

DSAF 2.99846626

DSAG 4.48638115

DSAH 4.20243502

DSAI 3.12508601

DSAK 4.48669435

DSAL 3.18978567

DSAM 3.47756154

DSAN 4.78789727

DSAP 4.52460905

DSAQ 4.56374205

DSAR 4.37336941

DSAS 5.78246094

DSAT 4.99797230

DSAV 3.51232862

DSAW 3.00871727

DSAY 3.36123236

DSCA 3.55231493

DSCC 2.26943622

DSCD 3.72554139

DSCE 3.63755533

DSCF 2.04882349

DSCG 3.05064627

DSCH 2.67483629

DSCI 2.25974465

DSCK 3.44880944

DSCL 2.21403735

DSCM 2.65094861

DSCN 3.70015652

DSCP 3.04058539

DSCQ 3.38535209

DSCR 3.19281171

DSCS 4.73181419

DSCT 4.04814321

DSCV 2.54484601

DSCW 1.84680474

DSCY 2.46726763

DSDA 7.87524681

DSDC 7.38040167

DSDD 7.32526739

DSDE 7.10028154

DSDF 5.83919857

DSDG 7.38725408

DSDH 6.83601544

DSDI 6.01922494

DSDK 7.02184482

DSDL 5.93862429

DSDM 6.44455540

DSDN 7.68635614

DSDP 7.16675696

DSDQ 7.08117550

DSDR 6.82518843

DSDS 10.87934329

DSDT 8.28227870

DSDV 6.38287762

DSDW 5.64074085

DSDY 6.11855800

DSEA 6.20674462

DSEC 5.81549977

DSED 6.06968336

DSEE 5.88129933

DSEF 4.52617229

DSEG 5.91716748

DSEH 5.52761791

DSEI 4.74509239

DSEK 5.76131282

DSEL 4.66320484

DSEM 5.06527408

DSEN 6.26450354

DSEP 5.75670619

DSEQ 5.79357986

DSER 5.50267707

DSES 7.47416030

DSET 6.47344748

DSEV 5.05599760

DSEW 4.36520257

DSEY 4.81770426

DSFA 3.03565140

DSFC 2.40779773

DSFD 3.36687543

DSFE 3.22417409

DSFF 1.72439889

DSFG 2.94347772

DSFH 2.71319640

DSFI 1.96245518

DSFK 3.14415005

DSFL 1.80765374

DSFM 2.22397047

DSFN 3.43736464

DSFP 2.90424807

DSFQ 3.07996884

DSFR 2.81299700

DSFS 4.18509292

DSFT 3.43538616

DSFV 2.11928272

DSFW 1.72049903

DSFY 2.13979715

DSGA 5.04657570

DSGC 4.13769374

DSGD 5.14436801

DSGE 5.07605789

DSGF 3.38639795

DSGG 4.55836085

DSGH 4.45098350

DSGI 3.61655698

DSGK 4.90384315

DSGL 3.57609218

DSGM 3.94535347

DSGN 5.13808014

DSGP 4.68332929

DSGQ 4.91321168

DSGR 4.65614852

DSGS 6.26217586

DSGT 5.41129418

DSGV 3.95652587

DSGW 3.35067081

DSGY 3.76078166

DSHA 4.95580593

DSHC 3.99836690

DSHD 4.97773948

DSHE 4.85047868

DSHF 3.39005894

DSHG 4.66161992

DSHH 4.29296348

DSHI 3.63931727

DSHK 4.70618531

DSHL 3.60115996

DSHM 3.87981357

DSHN 5.03526657

DSHP 4.62831984

DSHQ 4.70971816

DSHR 4.48887906

DSHS 6.16964403

DSHT 5.25888589

DSHV 3.92105411

DSHW 3.33390171

DSHY 3.73384041

DSIA 3.10421863

DSIC 2.66456205

DSID 3.52528156

DSIE 3.41565932

DSIF 1.86970659

DSIG 3.05675250

DSIH 2.88713398

DSII 1.97719773

DSIK 3.33199056

DSIL 1.88752677

DSIM 2.26846885

DSIN 3.60451438

DSIP 3.10408937

DSIQ 3.19990181

DSIR 2.96602532

DSIS 4.28268736

DSIT 3.56667115

DSIV 2.12898195

DSIW 1.80739261

DSIY 2.34661783

DSKA 5.10513727

DSKC 4.64369523

DSKD 5.26489306

DSKE 5.08793391

DSKF 3.74590213

DSKG 4.96568213

DSKH 4.68587256

DSKI 3.93660408

DSKK 4.97456672

DSKL 3.83876188

DSKM 4.12137893

DSKN 5.33309325

DSKP 4.85742650

DSKQ 4.87697169

DSKR 4.62328666

DSKS 6.15234559

DSKT 5.42000568

DSKV 4.13643314

DSKW 3.52524402

DSKY 4.09445883

DSLA 3.24004510

DSLC 2.71597280

DSLD 3.59114197

DSLE 3.42340627

DSLF 1.91922609

DSLG 3.13743972

DSLH 2.95379096

DSLI 2.03962194

DSLK 3.32262896

DSLL 1.91443474

DSLM 2.33917563

DSLN 3.63848742

DSLP 3.17029330

DSLQ 3.25505289

DSLR 3.01302561

DSLS 4.34591093

DSLT 3.62768068

DSLV 2.25488689

DSLW 1.84004203

DSLY 2.29847631

DSMA 3.49479208

DSMC 3.11592073

DSMD 3.84333949

DSME 3.70240255

DSMF 2.13550656

DSMG 3.40401974

DSMH 3.22984719

DSMI 2.31789645

DSMK 3.50301450

DSML 2.24263025

DSMM 2.58631390

DSMN 3.85621603

DSMP 3.44230243

DSMQ 3.46120917

DSMR 3.25088746

DSMS 4.59036998

DSMT 3.82509561

DSMV 2.50837050

DSMW 2.19930456

DSMY 2.56136055

DSNA 6.04813284

DSNC 5.47755204

DSND 6.15346884

DSNE 5.96428572

DSNF 4.46981805

DSNG 5.72590119

DSNH 5.40020633

DSNI 4.68901337

DSNK 5.79037509

DSNL 4.60008215

DSNM 4.92554082

DSNN 6.17388562

DSNP 5.61894735

DSNQ 5.75496612

DSNR 5.41265056

DSNS 7.32704980

DSNT 6.40217894

DSNV 4.94833915

DSNW 4.33331111

DSNY 4.80487754

DSPA 4.99295717

DSPC 4.10679501

DSPD 5.07656567

DSPE 4.97386100

DSPF 3.35993881

DSPG 4.58475930

DSPH 4.44821438

DSPI 3.61371031

DSPK 4.76707212

DSPL 3.60547677

DSPM 3.86046532

DSPN 5.03652228

DSPP 4.58669860

DSPQ 4.80442358

DSPR 4.53953157

DSPS 6.01820413

DSPT 5.25347269

DSPV 3.92479039

DSPW 3.25743559

DSPY 3.67290542

DSQA 5.28206222

DSQC 4.70455002

DSQD 5.38937358

DSQE 5.15721867

DSQF 3.76873808

DSQG 5.10908982

DSQH 4.76054344

DSQI 3.98037316

DSQK 4.99818338

DSQL 3.90899004

DSQM 4.16622514

DSQN 5.36901779

DSQP 4.97670362

DSQQ 5.02284646

DSQR 4.80007471

DSQS 6.29407439

DSQT 5.51489441

DSQV 4.23181216

DSQW 3.66803279

DSQY 4.07657456

DSRA 4.76605820

DSRC 4.18909433

DSRD 4.86808734

DSRE 4.66902093

DSRF 3.28765628

DSRG 4.54912630

DSRH 4.27118662

DSRI 3.46706774

DSRK 4.43891325

DSRL 3.41744167

DSRM 3.73062647

DSRN 4.79858029

DSRP 4.44269721

DSRQ 4.51112358

DSRR 4.34087804

DSRS 5.71869444

DSRT 4.99286232

DSRV 3.78306987

DSRW 3.30115459

DSRY 3.58708486

DSSA 5.40343147

DSSC 4.79704204

DSSD 5.61556839

DSSE 5.44019222

DSSF 3.87341826

DSSG 5.13605906

DSSH 4.85887440

DSSI 4.06677176

DSSK 5.20950612

DSSL 4.01630783

DSSM 4.27953885

DSSN 5.52884661

DSSP 5.09087705

DSSQ 5.18392517

DSSR 4.93320918

DSSS 6.43101670

DSST 5.67524189

DSSV 4.34421499

DSSW 3.74168633

DSSY 4.21830978

DSTA 4.96577758

DSTC 4.36815364

DSTD 5.19933232

DSTE 5.05654625

DSTF 3.41244633

DSTG 4.73879657

DSTH 4.43911814

DSTI 3.61247307

DSTK 4.81446733

DSTL 3.56326703

DSTM 3.86505319

DSTN 5.12036084

DSTP 4.70736455

DSTQ 4.83023893

DSTR 4.54245155

DSTS 5.99761008

DSTT 5.25848804

DSTV 3.88923510

DSTW 3.29788687

DSTY 3.74874444

DSVA 3.43796391

DSVC 2.97654644

DSVD 3.79984360

DSVE 3.68356250

DSVF 2.04028323

DSVG 3.33249898

DSVH 3.12083841

DSVI 2.16286314

DSVK 3.52007792

DSVL 2.08706927

DSVM 2.47952835

DSVN 3.80054452

DSVP 3.37235031

DSVQ 3.46918694

DSVR 3.25779955

DSVS 4.58802916

DSVT 3.83784852

DSVV 2.37293286

DSVW 2.04885996

DSVY 2.43283267

DSWA 3.23485453

DSWC 2.34470443

DSWD 3.35193073

DSWE 3.25623880

DSWF 1.78462882

DSWG 3.03087970

DSWH 2.78768534

DSWI 2.02257907

DSWK 3.13082263

DSWL 1.94022322

DSWM 2.44186104

DSWN 3.37486142

DSWP 2.92030171

DSWQ 3.14499187

DSWR 2.91479229

DSWS 4.24068022

DSWT 3.50659555

DSWV 2.24881480

DSWW 1.69566206

DSWY 2.17158822

DSYA 3.71531447

DSYC 3.16461972

DSYD 3.88794150

DSYE 3.76040757

DSYF 2.41131326

DSYG 3.60211060

DSYH 3.30577923

DSYI 2.61668927

DSYK 3.69845183

DSYL 2.51043894

DSYM 2.86873288

DSYN 4.03053567

DSYP 3.50507639

DSYQ 3.64288282

DSYR 3.31128558

DSYS 4.86233437

DSYT 4.09543380

DSYV 2.80231841

DSYW 2.18984188

DSYY 2.83050018

DTAA 4.46165271

DTAC 3.88239155

DTAD 4.64480412

DTAE 4.64273361

DTAF 3.24901180

DTAG 3.96587482

DTAH 4.07679588

DTAI 3.83981223

DTAK 4.43398165

DTAL 3.67875148

DTAM 3.82530155

DTAN 4.49475927

DTAP 4.26257385

DTAQ 4.48003909

DTAR 4.37572190

DTAS 5.02527678

DTAT 5.93896865

DTAV 4.23733416

DTAW 3.20165630

DTAY 3.46687168

DTCA 3.22391694

DTCC 2.00240441

DTCD 3.38172573

DTCE 3.42785669

DTCF 2.32988680

DTCG 2.45644971

DTCH 2.42131928

DTCI 2.86370139

DTCK 3.31755234

DTCL 2.62056196

DTCM 2.98085194

DTCN 3.37846802

DTCP 2.70133851

DTCQ 3.26251845

DTCR 3.17044692

DTCS 4.00384017

DTCT 4.95880990

DTCV 3.16697076

DTCW 1.85477997

DTCY 2.62183536

DTDA 7.35989757

DTDC 7.11701763

DTDD 6.88242414

DTDE 6.95114869

DTDF 6.09490777

DTDG 6.48194095

DTDH 6.66754600

DTDI 6.99959226

DTDK 7.00034007

DTDL 6.59327341

DTDM 6.96645949

DTDN 7.33744592

DTDP 6.74426841

DTDQ 6.97915782

DTDR 6.79518897

DTDS 8.28227870

DTDT 11.03167341

DTDV 7.36529560

DTDW 5.70060621

DTDY 6.15430995

DTEA 5.83705305

DTEC 5.55769507

DTED 5.67982717

DTEE 5.71499724

DTEF 4.74096141

DTEG 5.23930561

DTEH 5.36369360

DTEI 5.52195570

DTEK 5.67632242

DTEL 5.14669036

DTEM 5.45932074

DTEN 5.90779950

DTEP 5.40191786

DTEQ 5.65749137

DTER 5.45248847

DTES 6.46763536

DTET 7.58836906

DTEV 5.82591634

DTEW 4.42410375

DTEY 4.86695904

DTFA 2.80658744

DTFC 2.32449246

DTFD 3.08115625

DTFE 3.09525312

DTFF 1.89537971

DTFG 2.49695363

DTFH 2.58550477

DTFI 2.55052716

DTFK 3.04075149

DTFL 2.18539667

DTFM 2.47946712

DTFN 3.14947496

DTFP 2.64347525

DTFQ 2.96459465

DTFR 2.75325704

DTFS 3.48007994

DTFT 4.22352831

DTFV 2.69530707

DTFW 1.76994088

DTFY 2.20750121

DTGA 4.80404692

DTGC 4.00611762

DTGD 4.82881734

DTGE 5.02287459

DTGF 3.63538129

DTGG 3.98371190

DTGH 4.38095622

DTGI 4.38495886

DTGK 4.92825641

DTGL 4.09355999

DTGM 4.33065424

DTGN 4.85513282

DTGP 4.43464220

DTGQ 4.89109269

DTGR 4.72699903

DTGS 5.46303809

DTGT 6.45819112

DTGV 4.71714794

DTGW 3.44855302

DTGY 3.89428778

DTHA 4.62500177

DTHC 3.94676161

DTHD 4.61070199

DTHE 4.69480900

DTHF 3.63545794

DTHG 4.01658133

DTHH 4.11673204

DTHI 4.36925867

DTHK 4.62731177

DTHL 4.05191768

DTHM 4.24663339

DTHN 4.66783511

DTHP 4.27589898

DTHQ 4.58264552

DTHR 4.43411854

DTHS 5.26937621

DTHT 6.25659443

DTHV 4.61576327

DTHW 3.45164051

DTHY 3.81806562

DTIA 2.85185867

DTIC 2.57129731

DTID 3.20651728

DTIE 3.29573094

DTIF 2.11127245

DTIG 2.54933971

DTIH 2.76330805

DTII 2.57505767

DTIK 3.24515441

DTIL 2.29006603

DTIM 2.48877979

DTIN 3.33440861

DTIP 2.84502827

DTIQ 3.11409490

DTIR 2.91278881

DTIS 3.60043937

DTIT 4.32013222

DTIV 2.73128422

DTIW 1.91393168

DTIY 2.47289238

DTKA 4.78866855

DTKC 4.48411817

DTKD 4.89842279

DTKE 4.93528838

DTKF 3.95187050

DTKG 4.34237559

DTKH 4.49691930

DTKI 4.61664311

DTKK 4.89467832

DTKL 4.24251590

DTKM 4.41428487

DTKN 5.00639646

DTKP 4.52999041

DTKQ 4.75893904

DTKR 4.55096305

DTKS 5.39560503

DTKT 6.28172846

DTKV 4.80250936

DTKW 3.64071621

DTKY 4.17599068

DTLA 2.96702868

DTLC 2.63535411

DTLD 3.24573675

DTLE 3.26790740

DTLF 2.08698177

DTLG 2.63112140

DTLH 2.80588414

DTLI 2.60837736

DTLK 3.22769361

DTLL 2.29738620

DTLM 2.58439213

DTLN 3.33096083

DTLP 2.90974694

DTLQ 3.14414028

DTLR 2.96514761

DTLS 3.67312881

DTLT 4.39171961

DTLV 2.83347694

DTLW 1.92155351

DTLY 2.38878473

DTMA 3.22253851

DTMC 2.93822516

DTMD 3.49395502

DTME 3.54512927

DTMF 2.35560036

DTMG 2.85851986

DTMH 3.05637556

DTMI 2.89814893

DTMK 3.41661345

DTML 2.64450421

DTMM 2.87678936

DTMN 3.53748603

DTMP 3.14863729

DTMQ 3.35836292

DTMR 3.18086018

DTMS 3.86564001

DTMT 4.67464148

DTMV 3.12419307

DTMW 2.28974195

DTMY 2.68065281

DTNA 5.65794365

DTNC 5.30652020

DTND 5.72957396

DTNE 5.81959699

DTNF 4.67583666

DTNG 5.01129719

DTNH 5.22520109

DTNI 5.44564636

DTNK 5.74896814

DTNL 5.08952526

DTNM 5.29046638

DTNN 5.84004537

DTNP 5.24815528

DTNQ 5.67222222

DTNR 5.33651496

DTNS 6.35892070

DTNT 7.50201632

DTNV 5.69373105

DTNW 4.35896123

DTNY 4.86991553

DTPA 4.71454378

DTPC 3.93301981

DTPD 4.73246861

DTPE 4.82902829

DTPF 3.58271226

DTPG 4.06949288

DTPH 4.31575676

DTPI 4.29818589

DTPK 4.68206250

DTPL 4.05341645

DTPM 4.21103041

DTPN 4.74235691

DTPP 4.27456423

DTPQ 4.73172111

DTPR 4.53298384

DTPS 5.25919926

DTPT 6.14162451

DTPV 4.61975560

DTPW 3.40085718

DTPY 3.77246421

DTQA 4.95381434

DTQC 4.53320156

DTQD 5.00650146

DTQE 5.00057619

DTQF 3.97688300

DTQG 4.48256130

DTQH 4.60953543

DTQI 4.67096983

DTQK 4.90993136

DTQL 4.32904137

DTQM 4.48335633

DTQN 5.01608217

DTQP 4.65188068

DTQQ 4.89641741

DTQR 4.74879164

DTQS 5.50593459

DTQT 6.40389281

DTQV 4.91531036

DTQW 3.81099923

DTQY 4.13008136

DTRA 4.51770878

DTRC 4.09028206

DTRD 4.54700194

DTRE 4.54939044

DTRF 3.52894149

DTRG 3.99824957

DTRH 4.19119215

DTRI 4.13958677

DTRK 4.33192230

DTRL 3.86895583

DTRM 4.02208494

DTRN 4.50470619

DTRP 4.16383996

DTRQ 4.38976106

DTRR 4.31652154

DTRS 4.99961762

DTRT 5.85066020

DTRV 4.47255818

DTRW 3.34446289

DTRY 3.68849735

DTSA 5.07863844

DTSC 4.62268218

DTSD 5.24679461

DTSE 5.29808587

DTSF 4.08268327

DTSG 4.51585973

DTSH 4.71258579

DTSI 4.74701232

DTSK 5.12684444

DTSL 4.46779237

DTSM 4.61325003

DTSN 5.20515588

DTSP 4.74096684

DTSQ 5.08021219

DTSR 4.87154139

DTSS 5.65155527

DTST 6.58890928

DTSV 5.03808769

DTSW 3.82769202

DTSY 4.28168981

DTTA 4.70943334

DTTC 4.19333716

DTTD 4.91909891

DTTE 5.01422491

DTTF 3.66362121

DTTG 4.19423078

DTTH 4.35675496

DTTI 4.33160484

DTTK 4.81885125

DTTL 4.02762586

DTTM 4.20180063

DTTN 4.88395883

DTTP 4.44419676

DTTQ 4.81485905

DTTR 4.57537410

DTTS 5.30667501

DTTT 6.19439175

DTTV 4.60088055

DTTW 3.44005785

DTTY 3.88492965

DTVA 3.20347379

DTVC 2.77007801

DTVD 3.53140222

DTVE 3.58722492

DTVF 2.27000466

DTVG 2.87726096

DTVH 3.01089049

DTVI 2.79152280

DTVK 3.48275367

DTVL 2.54203877

DTVM 2.76725539

DTVN 3.52920891

DTVP 3.13542981

DTVQ 3.41305116

DTVR 3.26889023

DTVS 3.92175719

DTVT 4.67778321

DTVV 3.02198007

DTVW 2.13453755

DTVY 2.56094553

DTWA 3.01935302

DTWC 2.47821055

DTWD 3.11798041

DTWE 3.13662598

DTWF 2.13677523

DTWG 2.50666698

DTWH 2.65371115

DTWI 2.64483237

DTWK 3.09582302

DTWL 2.42683908

DTWM 2.77700102

DTWN 3.08953846

DTWP 2.64301980

DTWQ 3.08016927

DTWR 2.91931196

DTWS 3.57749440

DTWT 4.35360829

DTWV 2.90527376

DTWW 1.82916817

DTWY 2.37548366

DTYA 3.47368787

DTYC 3.06062653

DTYD 3.55506324

DTYE 3.62598048

DTYF 2.62814776

DTYG 3.05074580

DTYH 3.16327309

DTYI 3.25871952

DTYK 3.62747572

DTYL 2.90575215

DTYM 3.19632605

DTYN 3.79104606

DTYP 3.22214229

DTYQ 3.50536174

DTYR 3.25896788

DTYS 4.11096978

DTYT 4.95022389

DTYV 3.44183569

DTYW 2.37065371

DTYY 2.86066655

DVAA 4.48519554

DVAC 4.40805531

DVAD 3.39023327

DVAE 3.76740783

DVAF 4.26747952

DVAG 3.15213449

DVAH 3.61151506

DVAI 5.60007162

DVAK 3.60015576

DVAL 4.95913110

DVAM 4.53857112

DVAN 3.32539388

DVAP 3.82804635

DVAQ 3.73820882

DVAR 3.71918397

DVAS 3.76761696

DVAT 4.46995162

DVAV 6.08905249

DVAW 3.67463587

DVAY 3.91012711

DVCA 3.35864002

DVCC 1.03600000

DVCD 2.30789675

DVCE 2.66175093

DVCF 3.14391163

DVCG 1.82784748

DVCH 1.98941995

DVCI 4.49090836

DVCK 2.59293722

DVCL 3.81025640

DVCM 3.53667428

DVCN 2.24062159

DVCP 2.40916394

DVCQ 2.62484731

DVCR 2.51708482

DVCS 2.83804289

DVCT 3.43272224

DVCV 4.93528314

DVCW 2.23245680

DVCY 2.92157605

DVDA 7.45535147

DVDC 7.54554162

DVDD 5.46344787

DVDE 6.09697561

DVDF 7.23922500

DVDG 5.53344589

DVDH 6.03786232

DVDI 9.28368133

DVDK 6.07418553

DVDL 8.16700352

DVDM 7.73497477

DVDN 5.83906163

DVDP 6.39102545

DVDQ 6.18474173

DVDR 6.04528149

DVDS 6.38287762

DVDT 7.36529560

DVDV 11.09882805

DVDW 6.20908952

DVDY 6.62623216

DVEA 5.92612392

DVEC 6.01320396

DVED 4.42661000

DVEE 4.86617650

DVEF 5.78176112

DVEG 4.36532260

DVEH 4.79456968

DVEI 7.40147957

DVEK 4.81164457

DVEL 6.51267506

DVEM 6.17337275

DVEN 4.62866692

DVEP 4.99171869

DVEQ 4.88240824

DVER 4.76079082

DVES 5.09353476

DVET 5.87099971

DVEV 7.90259255

DVEW 4.91957643

DVEY 5.28775458

DVFA 2.98085355

DVFC 2.78864881

DVFD 1.97742677

DVFE 2.34864761

DVFF 2.79758319

DVFG 1.82380340

DVFH 2.15472496

DVFI 4.09485386

DVFK 2.42108931

DVFL 3.33607497

DVFM 3.07937721

DVFN 2.11352158

DVFP 2.32355569

DVFQ 2.34140888

DVFR 2.22094847

DVFS 2.40687773

DVFT 2.97715743

DVFV 4.32728874

DVFW 2.17376012

DVFY 2.60537119

DVGA 4.90797919

DVGC 4.51425470

DVGD 3.51088664

DVGE 4.17698613

DVGF 4.64453392

DVGG 3.18009535

DVGH 3.88736636

DVGI 6.16458605

DVGK 4.09321346

DVGL 5.41742372

DVGM 5.00174712

DVGN 3.64554633

DVGP 4.06873252

DVGQ 4.15372397

DVGR 4.03750734

DVGS 4.14288537

DVGT 4.85523374

DVGV 6.65328841

DVGW 4.13518423

DVGY 4.31309974

DVHA 4.77163999

DVHC 4.37601178

DVHD 3.36861847

DVHE 3.87474518

DVHF 4.64788159

DVHG 3.24906876

DVHH 3.54744788

DVHI 6.13586351

DVHK 3.81262858

DVHL 5.32711155

DVHM 4.92477948

DVHN 3.50266582

DVHP 3.88593852

DVHQ 3.90842187

DVHR 3.80076132

DVHS 4.01763651

DVHT 4.71397221

DVHV 6.53261035

DVHW 3.84149586

DVHY 4.23359662

DVIA 3.03312366

DVIC 3.07326204

DVID 2.13268829

DVIE 2.55655115

DVIF 3.01360091

DVIG 1.90850066

DVIH 2.32764189

DVII 4.12847570

DVIK 2.51384063

DVIL 3.44694590

DVIM 3.17003550

DVIN 2.32727707

DVIP 2.48298890

DVIQ 2.46732605

DVIR 2.32828553

DVIS 2.61199579

DVIT 3.07633528

DVIV 4.38354133

DVIW 2.40163130

DVIY 2.78249793

DVKA 4.89064412

DVKC 4.98018032

DVKD 3.58775752

DVKE 4.01230662

DVKF 4.93204200

DVKG 3.53601888

DVKH 3.97480473

DVKI 6.29193161

DVKK 4.02482393

DVKL 5.45801736

DVKM 5.07455305

DVKN 3.80666814

DVKP 4.10031232

DVKQ 3.96579679

DVKR 3.86799853

DVKS 4.17258513

DVKT 4.83012126

DVKV 6.58762956

DVKW 4.01293861

DVKY 4.52665952

DVLA 3.13513713

DVLC 3.12078712

DVLD 2.13690770

DVLE 2.49388761

DVLF 3.00506076

DVLG 1.94610508

DVLH 2.36908601

DVLI 4.22614094

DVLK 2.48226222

DVLL 3.48314223

DVLM 3.28085770

DVLN 2.29817289

DVLP 2.54548936

DVLQ 2.49919165

DVLR 2.38346642

DVLS 2.60836473

DVLT 3.15768608

DVLV 4.49400948

DVLW 2.38475658

DVLY 2.76257239

DVMA 3.37288218

DVMC 3.43032190

DVMD 2.32632555

DVME 2.75627874

DVMF 3.25344221

DVMG 2.22181577

DVMH 2.62371976

DVMI 4.54184402

DVMK 2.66428284

DVML 3.83051344

DVMM 3.53929080

DVMN 2.47690282

DVMP 2.80688069

DVMQ 2.68843187

DVMR 2.59152542

DVMS 2.77349005

DVMT 3.36360789

DVMV 4.87042436

DVMW 2.70806076

DVMY 3.07011676

DVNA 5.65187898

DVNC 5.66642232

DVND 4.31549122

DVNE 4.85649394

DVNF 5.61511860

DVNG 4.09072086

DVNH 4.60247318

DVNI 7.20869267

DVNK 4.82677212

DVNL 6.29619632

DVNM 5.89535829

DVNN 4.48897711

DVNP 4.84834935

DVNQ 4.79374373

DVNR 4.59759403

DVNS 4.90237210

DVNT 5.66074053

DVNV 7.65521019

DVNW 4.73908283

DVNY 5.17260292

DVPA 4.74213621

DVPC 4.42511655

DVPD 3.47582047

DVPE 3.97742631

DVPF 4.54149927

DVPG 3.24732965

DVPH 3.78289395

DVPI 5.97019084

DVPK 3.87189470

DVPL 5.26543851

DVPM 4.84123495

DVPN 3.53913729

DVPP 3.89819958

DVPQ 3.98894569

DVPR 3.90511073

DVPS 4.00867912

DVPT 4.66774877

DVPV 6.35838514

DVPW 3.83174739

DVPY 4.17543506

DVQA 5.03480338

DVQC 5.07004079

DVQD 3.70980434

DVQE 4.09625549

DVQF 4.98341603

DVQG 3.70801647

DVQH 4.06582209

DVQI 6.41752275

DVQK 4.06625118

DVQL 5.60759588

DVQM 5.19469625

DVQN 3.83467568

DVQP 4.22865400

DVQQ 4.09584825

DVQR 4.06941862

DVQS 4.23097458

DVQT 4.96726468

DVQV 6.80765603

DVQW 4.29485982

DVQY 4.53639824

DVRA 4.61782071

DVRC 4.63052697

DVRD 3.24147782

DVRE 3.68335550

DVRF 4.47669508

DVRG 3.21284277

DVRH 3.67877812

DVRI 5.82624759

DVRK 3.53802211

DVRL 5.09978090

DVRM 4.73524382

DVRN 3.34391523

DVRP 3.77784337

DVRQ 3.66131832

DVRR 3.64974434

DVRS 3.82512841

DVRT 4.49516514

DVRV 6.22151704

DVRW 3.80314836

DVRY 4.03438477

DVSA 5.08326978

DVSC 5.05408383

DVSD 3.87370005

DVSE 4.30799598

DVSF 5.00408805

DVSG 3.67114414

DVSH 4.10202783

DVSI 6.42673607

DVSK 4.22780268

DVSL 5.66239133

DVSM 5.23031000

DVSN 3.93249947

DVSP 4.29039430

DVSQ 4.22328834

DVSR 4.14954972

DVSS 4.33008640

DVST 5.03303867

DVSV 6.79886326

DVSW 4.24567544

DVSY 4.58731809

DVTA 4.72928735

DVTC 4.66393737

DVTD 3.55419783

DVTE 4.00496561

DVTF 4.63272808

DVTG 3.36672448

DVTH 3.81965537

DVTI 5.99704449

DVTK 3.91138044

DVTL 5.28718326

DVTM 4.85394193

DVTN 3.63300693

DVTP 3.99984654

DVTQ 3.94978040

DVTR 3.86291109

DVTS 4.00252780

DVTT 4.66168128

DVTV 6.35521983

DVTW 3.89346201

DVTY 4.26344746

DVVA 3.35124530

DVVC 3.24876944

DVVD 2.41266462

DVVE 2.80746864

DVVF 3.26309972

DVVG 2.16321686

DVVH 2.60853300

DVVI 4.39746862

DVVK 2.71165824

DVVL 3.74995658

DVVM 3.46507365

DVVN 2.47530450

DVVP 2.76405341

DVVQ 2.75906823

DVVR 2.65183626

DVVS 2.79736872

DVVT 3.34637538

DVVV 4.70288638

DVVW 2.60503429

DVVY 2.96962626

DVWA 3.24478568

DVWC 2.85346064

DVWD 2.00183621

DVWE 2.43598657

DVWF 2.99434778

DVWG 1.94147391

DVWH 2.36153658

DVWI 4.21349506

DVWK 2.40973425

DVWL 3.52336162

DVWM 3.31881650

DVWN 2.08548928

DVWP 2.35084523

DVWQ 2.52191984

DVWR 2.40301409

DVWS 2.54338798

DVWT 3.12165124

DVWV 4.51217028

DVWW 2.17442353

DVWY 2.73538579

DVYA 3.59977327

DVYC 3.49321710

DVYD 2.39595020

DVYE 2.84011147

DVYF 3.50589819

DVYG 2.32099117

DVYH 2.67596818

DVYI 4.83792479

DVYK 2.89379144

DVYL 4.03053229

DVYM 3.78867427

DVYN 2.66586376

DVYP 2.82520998

DVYQ 2.84855462

DVYR 2.65068646

DVYS 2.96368214

DVYT 3.59356059

DVYV 5.11775187

DVYW 2.67887169

DVYY 3.19568272

DWAA 3.33813714

DWAC 3.35164183

DWAD 2.99209315

DWAE 3.09289737

DWAF 5.26175673

DWAG 2.85383211

DWAH 4.08400276

DWAI 3.60092039

DWAK 2.97895638

DWAL 3.99775217

DWAM 3.82360700

DWAN 2.92986756

DWAP 3.14579057

DWAQ 3.28807617

DWAR 3.51299717

DWAS 3.12648534

DWAT 3.23052907

DWAV 3.52231493

DWAW 8.46243317

DWAY 5.39437250

DWCA 2.40972016

DWCC 0.59984655

DWCD 2.03806156

DWCE 2.26233803

DWCF 4.15614763

DWCG 1.56485378

DWCH 2.81017480

DWCI 2.84447217

DWCK 2.27279961

DWCL 3.02624711

DWCM 3.02575307

DWCN 2.25053254

DWCP 1.91288950

DWCQ 2.42135462

DWCR 2.47797895

DWCS 2.32421007

DWCT 2.58030399

DWCV 2.62266872

DWCW 7.06709924

DWCY 4.31685166

DWDA 5.82310811

DWDC 6.34514058

DWDD 5.12982267

DWDE 5.23697164

DWDF 8.49525061

DWDG 5.24275596

DWDH 6.70121107

DWDI 6.46032597

DWDK 5.33714087

DWDL 6.86386438

DWDM 6.73922999

DWDN 5.42629318

DWDP 5.49728026

DWDQ 5.67394550

DWDR 5.95918685

DWDS 5.64074085

DWDT 5.70060621

DWDV 6.20908952

DWDW 14.03042515

DWDY 8.55774672

DWEA 4.49022334

DWEC 4.77380131

DWED 3.87730523

DWEE 4.01206512

DWEF 6.70440401

DWEG 3.97004384

DWEH 5.18198687

DWEI 4.99126331

DWEK 3.99221007

DWEL 5.33777960

DWEM 5.21918035

DWEN 4.10817134

DWEP 4.11189753

DWEQ 4.27607851

DWER 4.51859776

DWES 4.26334900

DWET 4.35435901

DWEV 4.81708056

DWEW 10.44382800

DWEY 6.77161373

DWFA 2.18304536

DWFC 2.11122493

DWFD 1.87346976

DWFE 2.09951226

DWFF 3.81127776

DWFG 1.77231380

DWFH 2.66374482

DWFI 2.54980427

DWFK 2.04627838

DWFL 2.62524068

DWFM 2.64059510

DWFN 2.04053092

DWFP 2.00341808

DWFQ 2.13791619

DWFR 2.28143653

DWFS 2.07951338

DWFT 2.15004062

DWFV 2.39568347

DWFW 6.59980395

DWFY 3.98485244

DWGA 3.71721734

DWGC 3.43903603

DWGD 3.16515769

DWGE 3.54982563

DWGF 5.58263251

DWGG 2.87259178

DWGH 4.29210595

DWGI 4.07736394

DWGK 3.43694117

DWGL 4.40732576

DWGM 4.34552464

DWGN 3.41177107

DWGP 3.35841954

DWGQ 3.66072466

DWGR 3.84980353

DWGS 3.53186923

DWGT 3.65478793

DWGV 3.94325987

DWGW 9.00046414

DWGY 5.81712187

DWHA 3.60413716

DWHC 3.38070502

DWHD 3.09044909

DWHE 3.33283627

DWHF 5.59038644

DWHG 2.96931017

DWHH 4.01137985

DWHI 4.09802298

DWHK 3.33568487

DWHL 4.37161292

DWHM 4.30735948

DWHN 3.23283063

DWHP 3.32254988

DWHQ 3.55973126

DWHR 3.71423612

DWHS 3.45364360

DWHT 3.50551326

DWHV 3.89385363

DWHW 8.77432717

DWHY 5.59906172

DWIA 1.98869745

DWIC 2.23528671

DWID 1.86285311

DWIE 1.94706610

DWIF 3.82947354

DWIG 1.68935470

DWIH 2.64812107

DWII 2.42084106

DWIK 1.97964370

DWIL 2.60431697

DWIM 2.57456055

DWIN 1.99773087

DWIP 1.99426605

DWIQ 2.07590041

DWIR 2.21094948

DWIS 2.05681107

DWIT 2.04019365

DWIV 2.21152129

DWIW 6.89185531

DWIY 4.11373971

DWKA 3.58196919

DWKC 3.94851216

DWKD 3.07073094

DWKE 3.23512446

DWKF 5.69021149

DWKG 3.14934509

DWKH 4.19256806

DWKI 4.22126391

DWKK 3.33481435

DWKL 4.37551461

DWKM 4.24656949

DWKN 3.36578087

DWKP 3.34698535

DWKQ 3.41895053

DWKR 3.61005805

DWKS 3.43906562

DWKT 3.52933137

DWKV 3.91100187

DWKW 8.97984792

DWKY 5.77870816

DWLA 2.20598308

DWLC 2.40272126

DWLD 1.90038621

DWLE 2.00601561

DWLF 3.92167944

DWLG 1.78600498

DWLH 2.74728069

DWLI 2.55695416

DWLK 2.01088572

DWLL 2.72219860

DWLM 2.70170175

DWLN 2.05768599

DWLP 2.06520911

DWLQ 2.13389985

DWLR 2.31286800

DWLS 2.11981133

DWLT 2.16342135

DWLV 2.40834549

DWLW 6.82040795

DWLY 4.09627578

DWMA 2.40082705

DWMC 2.59456099

DWMD 2.08991823

DWME 2.26985208

DWMF 4.16778601

DWMG 1.95030466

DWMH 2.98316327

DWMI 2.72440976

DWMK 2.37033520

DWML 2.94320486

DWMM 2.91049228

DWMN 2.29765768

DWMP 2.35040726

DWMQ 2.36208243

DWMR 2.54768939

DWMS 2.25449553

DWMT 2.36339879

DWMV 2.61287254

DWMW 7.29561034

DWMY 4.34055526

DWNA 4.31172105

DWNC 4.69315789

DWND 3.85958896

DWNE 4.03252446

DWNF 6.62246245

DWNG 3.86979837

DWNH 5.08940349

DWNI 4.96868925

DWNK 4.15654063

DWNL 5.20105572

DWNM 5.10806236

DWNN 4.07689780

DWNP 4.07839963

DWNQ 4.25991070

DWNR 4.45797352

DWNS 4.22504055

DWNT 4.26788534

DWNV 4.69454834

DWNW 10.37043694

DWNY 6.77961207

DWPA 3.66716582

DWPC 3.48747603

DWPD 3.16441244

DWPE 3.36100378

DWPF 5.49303736

DWPG 3.00221787

DWPH 4.41673381

DWPI 4.09666356

DWPK 3.27151185

DWPL 4.37195778

DWPM 4.22993633

DWPN 3.26800588

DWPP 3.21371913

DWPQ 3.56884293

DWPR 3.71003028

DWPS 3.40722244

DWPT 3.50215268

DWPV 3.93725710

DWPW 8.57062319

DWPY 5.60253666

DWQA 3.79822252

DWQC 3.97981880

DWQD 3.26797768

DWQE 3.33255384

DWQF 5.79706932

DWQG 3.28568917

DWQH 4.36482974

DWQI 4.26989242

DWQK 3.38058098

DWQL 4.54139501

DWQM 4.38502457

DWQN 3.37864095

DWQP 3.47409468

DWQQ 3.56998701

DWQR 3.80814960

DWQS 3.55515865

DWQT 3.63426618

DWQV 4.05351521

DWQW 9.16614185

DWQY 5.90808703

DWRA 3.55340543

DWRC 3.72772358

DWRD 2.94295852

DWRE 3.10135118

DWRF 5.33257135

DWRG 2.90524168

DWRH 4.06325667

DWRI 3.94231209

DWRK 2.99223799

DWRL 4.19456238

DWRM 4.09345828

DWRN 3.12869325

DWRP 3.18630370

DWRQ 3.28887246

DWRR 3.47732996

DWRS 3.26936145

DWRT 3.35499294

DWRV 3.83369628

DWRW 8.31280333

DWRY 5.39484872

DWSA 3.86705002

DWSC 4.04827181

DWSD 3.46162074

DWSE 3.54593848

DWSF 5.92814541

DWSG 3.36672254

DWSH 4.45669758

DWSI 4.32934757

DWSK 3.57063564

DWSL 4.61999165

DWSM 4.48283830

DWSN 3.56130146

DWSP 3.58701484

DWSQ 3.75926810

DWSR 3.93038605

DWSS 3.66392164

DWST 3.75624184

DWSV 4.15972182

DWSW 9.29853323

DWSY 6.12079099

DWTA 3.51050703

DWTC 3.76383682

DWTD 3.13948826

DWTE 3.27045951
[truncated: 2,320,509 more chars]
