# Supplementary material for: New alignment method for remote protein sequences by the direct use of pairwise sequence correlations and substitutions
Source: Front Bioinform. 2023 Oct 12;3:1227193. doi: 10.3389/fbinf.2023.1227193 (PMC10602800; doi:10.3389/fbinf.2023.1227193)
Supplement: Supplementary file 2 [file DataSheet1.PDF]

Supporting information for

New Alignment Method for Protein Sequences by the Direct Use of Pairwise Sequence Correlations

Kejue Jia,[1] Mesih Kilinc,[1,2] Robert Jernigan[1,2]

[1]Roy J. Carver Department of Biochemistry, Biophysics, and Molecular Biology

[1]Bioinformatics and Computational Biology Program

Iowa State University

Ames, IA 50011

S1. ProSub400 matrix

The ProtSub400 matrix can be found in supporting information file "PS400.txt". This matrix comprises 400 × 400 elements, where each element represents a log-odds score indicating the likelihood of substitutions between specific pairs of amino acids. For improved readability, we have formatted the matrix into "key-value" tuples.

S2. CAO contact matrix

The CAO substitution matrix (Kleinjung, J., et al., *Contact-based sequence alignment.* Nucleic Acids Res, 2004. **32**(8): p. 2464-73) utilizes a Markov model to analyze the evolution of protein side-chain contacts. The matrix can be found in the file "CAO120.txt" in the same "key-value" tuple format. It consists of a 400 × 400 contact substitution scores. Each row and column represent the 20 × 20 potential combinations of residue contacts. Within each matrix cell, there is a score indicating the evolutionary transition (mutation) from a contact specified by the row to a contact specified by the column axis.

S3. The list of CATHS20 H-level PDB structures

A set of homologous proteins that have 20% sequence identities and high structural similarities is used to test the level of agreement between sequence alignment and structure alignment. See file "supplemental.tables.xlsx".

S4. Pfam domains

The list of Pfam MSA used for derive ProtSub400 matrix are provided in "supplemetal.tables.xlsx".
