# Supplementary material for: New alignment method for remote protein sequences by the direct use of pairwise sequence correlations and substitutions
Source: Front Bioinform. 2023 Oct 12;3:1227193. doi: 10.3389/fbinf.2023.1227193 (PMC10602800; doi:10.3389/fbinf.2023.1227193)
Supplement: Supplementary file 4 [file DataSheet2.docx]

AAAA 4.22072

AAAC -0.212266

AAAD -1.00676

AAAE -0.880938

AAAF -0.532094

AAAG -1.15225

AAAH -0.472935

AAAI -0.803677

AAAK -0.874805

AAAL -0.942532

AAAM -0.243723

AAAN -0.890528

AAAP -1.06848

AAAQ -0.624698

AAAR -0.622609

AAAS -0.680397

AAAT -0.650535

AAAV -0.61685

AAAW -0.149566

AAAY -0.478342

AACA -0.16687

AACC 1.7592

AACD -0.352855

AACE -0.412763

AACF 0.376933

AACG -0.483069

AACH 0.346661

AACI 0.115304

AACK -0.217052

AACL -0.0179198

AACM 0.34357

AACN -0.126877

AACP -0.24152

AACQ 0.0438828

AACR -0.00794101

AACS -0.139704

AACT -0.16036

AACV 0.0579772

AACW 0.732473

AACY 0.424178

AADA -0.889008

AADC -0.337345

AADD -0.828214

AADE -0.836291

AADF -0.542312

AADG -1.16292

AADH -0.271209

AADI -0.795282

AADK -0.658705

AADL -1.06999

AADM -0.460238

AADN -0.57038

AADP -0.897744

AADQ -0.551732

AADR -0.507912

AADS -0.781749

AADT -0.709517

AADV -0.914042

AADW -0.0738223

AADY -0.346802

AAEA -0.796861

AAEC -0.401477

AAED -0.943019

AAEE -0.905453

AAEF -0.579361

AAEG -1.39105

AAEH -0.363428

AAEI -0.806574

AAEK -0.656876

AAEL -1.08405

AAEM -0.448519

AAEN -0.739424

AAEP -1.02482

AAEQ -0.589448

AAER -0.575164

AAES -0.957378

AAET -0.87204

AAEV -0.974062

AAEW -0.0643733

AAEY -0.425833

AAFA -0.47859

AAFC 0.396584

AAFD -0.48367

AAFE -0.572838

AAFF 0.138583

AAFG -0.867558

AAFH 0.138096

AAFI -0.195908

AAFK -0.504147

AAFL -0.477439

AAFM 0.316006

AAFN -0.349738

AAFP -0.443525

AAFQ -0.220381

AAFR -0.313609

AAFS -0.496679

AAFT -0.371008

AAFV -0.35499

AAFW 0.564811

AAFY 0.135064

AAGA -1.22074

AAGC -0.578291

AAGD -1.16388

AAGE -1.26713

AAGF -0.790466

AAGG -2.19932

AAGH -0.805883

AAGI -1.07696

AAGK -1.15145

AAGL -1.33136

AAGM -0.719325

AAGN -0.993079

AAGP -1.32483

AAGQ -0.965319

AAGR -0.948541

AAGS -1.13967

AAGT -1.05263

AAGV -1.17073

AAGW -0.466184

AAGY -0.697817

AAHA -0.548662

AAHC 0.29443

AAHD -0.228461

AAHE -0.353575

AAHF 0.150675

AAHG -0.807238

AAHH 0.292022

AAHI -0.20491

AAHK -0.485165

AAHL -0.35092

AAHM 0.134428

AAHN -0.250619

AAHP -0.416814

AAHQ -0.208765

AAHR -0.188302

AAHS -0.320305

AAHT -0.260257

AAHV -0.315032

AAHW 0.516111

AAHY 0.191433

AAIA -0.676135

AAIC 0.183799

AAID -0.79109

AAIE -0.843951

AAIF -0.107656

AAIG -1.16643

AAIH -0.176176

AAII -0.4403

AAIK -0.754553

AAIL -0.776457

AAIM 0.077106

AAIN -0.61316

AAIP -0.733422

AAIQ -0.490379

AAIR -0.551501

AAIS -0.732196

AAIT -0.625521

AAIV -0.606007

AAIW 0.286211

AAIY -0.0594208

AAKA -0.588679

AAKC -0.271122

AAKD -0.644981

AAKE -0.720736

AAKF -0.519829

AAKG -1.23384

AAKH -0.472817

AAKI -0.756288

AAKK -0.822601

AAKL -0.944915

AAKM -0.452695

AAKN -0.701854

AAKP -0.94062

AAKQ -0.542662

AAKR -0.781995

AAKS -0.877884

AAKT -0.794258

AAKV -0.90204

AAKW -0.147908

AAKY -0.390481

AALA -0.959292

AALC 0.0726507

AALD -1.07023

AALE -1.08695

AALF -0.489418

AALG -1.3702

AALH -0.332703

AALI -0.783533

AALK -1.03142

AALL -1.09204

AALM -0.0968115

AALN -0.880962

AALP -0.946908

AALQ -0.648476

AALR -0.827562

AALS -1.0097

AALT -0.950039

AALV -1.00522

AALW 0.123279

AALY -0.41457

AAMA -0.325966

AAMC 0.415756

AAMD -0.402728

AAME -0.41376

AAMF 0.316132

AAMG -0.888207

AAMH 0.0345864

AAMI 0.0839968

AAMK -0.370143

AAML -0.145097

AAMM 0.438416

AAMN -0.345652

AAMP -0.503861

AAMQ -0.182281

AAMR -0.19651

AAMS -0.416335

AAMT -0.274385

AAMV -0.0720577

AAMW 0.447289

AAMY 0.27919

AANA -0.778641

AANC -0.127422

AAND -0.60727

AANE -0.720107

AANF -0.333976

AANG -1.04102

AANH -0.236963

AANI -0.657819

AANK -0.595677

AANL -0.896288

AANM -0.331982

AANN -0.404628

AANP -0.704217

AANQ -0.358326

AANR -0.505858

AANS -0.514176

AANT -0.592742

AANV -0.770701

AANW 0.121821

AANY -0.137896

AAPA -0.673488

AAPC -0.176214

AAPD -0.702233

AAPE -0.650957

AAPF -0.309484

AAPG -1.05925

AAPH -0.264591

AAPI -0.641543

AAPK -0.73711

AAPL -0.848444

AAPM -0.276191

AAPN -0.563997

AAPP -0.706295

AAPQ -0.453467

AAPR -0.522608

AAPS -0.686402

AAPT -0.627811

AAPV -0.685706

AAPW 0.168665

AAPY -0.209363

AAQA -0.743943

AAQC 0.0322852

AAQD -0.611212

AAQE -0.614025

AAQF -0.192511

AAQG -0.996764

AAQH -0.236951

AAQI -0.520904

AAQK -0.536306

AAQL -0.688518

AAQM -0.220046

AAQN -0.403476

AAQP -0.699127

AAQQ -0.177742

AAQR -0.352458

AAQS -0.580247

AAQT -0.500458

AAQV -0.604403

AAQW 0.164451

AAQY -0.0982254

AARA -0.576094

AARC -0.0880692

AARD -0.54842

AARE -0.528393

AARF -0.305914

AARG -1.13177

AARH -0.203707

AARI -0.563529

AARK -0.780102

AARL -0.636668

AARM -0.173083

AARN -0.565828

AARP -0.709874

AARQ -0.384888

AARR -0.462226

AARS -0.644184

AART -0.653307

AARV -0.689677

AARW 0.100779

AARY -0.176062

AASA -0.402257

AASC -0.102082

AASD -0.799665

AASE -0.811194

AASF -0.467749

AASG -1.09104

AASH -0.301468

AASI -0.7607

AASK -0.847677

AASL -1.04154

AASM -0.373892

AASN -0.625547

AASP -0.880299

AASQ -0.501455

AASR -0.670335

AASS -0.770707

AAST -0.679396

AASV -0.847908

AASW -0.0515587

AASY -0.342936

AATA -0.474482

AATC -0.0231197

AATD -0.765797

AATE -0.75832

AATF -0.366212

AATG -1.08467

AATH -0.262642

AATI -0.629604

AATK -0.757629

AATL -0.959208

AATM -0.250754

AATN -0.57578

AATP -0.786618

AATQ -0.445019

AATR -0.556713

AATS -0.700603

AATT -0.648662

AATV -0.740787

AATW 0.0629752

AATY -0.267981

AAVA -0.390896

AAVC 0.0929356

AAVD -0.953829

AAVE -0.919291

AAVF -0.364279

AAVG -1.25256

AAVH -0.303047

AAVI -0.590314

AAVK -0.91272

AAVL -0.996994

AAVM -0.0763597

AAVN -0.769019

AAVP -0.831938

AAVQ -0.626096

AAVR -0.726092

AAVS -0.848444

AAVT -0.775156

AAVV -0.84095

AAVW 0.157887

AAVY -0.317663

AAWA -0.176419

AAWC 0.736398

AAWD -0.12175

AAWE -0.150316

AAWF 0.586614

AAWG -0.416707

AAWH 0.340544

AAWI 0.323311

AAWK -0.111275

AAWL 0.130019

AAWM 0.534608

AAWN 0.0596566

AAWP -0.0745013

AAWQ 0.181162

AAWR 0.151765

AAWS -0.11422

AAWT -0.0202

AAWV 0.189321

AAWW 0.881752

AAWY 0.679598

AAYA -0.470535

AAYC 0.592217

AAYD -0.370172

AAYE -0.497177

AAYF 0.145469

AAYG -0.735187

AAYH 0.147838

AAYI -0.154333

AAYK -0.393818

AAYL -0.453364

AAYM 0.253152

AAYN -0.231311

AAYP -0.311371

AAYQ -0.0818179

AAYR -0.173791

AAYS -0.421132

AAYT -0.294268

AAYV -0.321983

AAYW 0.63142

AAYY 0.223587

ACAA -0.465176

ACAC 3.5905

ACAD -0.472004

ACAE -0.474126

ACAF 0.0653925

ACAG -0.911341

ACAH 0.0435688

ACAI -0.168967

ACAK -0.394661

ACAL -0.443826

ACAM 0.220539

ACAN -0.320858

ACAP -0.608855

ACAQ -0.184113

ACAR -0.237703

ACAS -0.429914

ACAT -0.335516

ACAV -0.286483

ACAW 0.392286

ACAY 0.104393

ACCA 0.291967

ACCC 2.34378

ACCD 0.0935543

ACCE 0.036052

ACCF 0.896289

ACCG -0.0219104

ACCH 0.763971

ACCI 0.623071

ACCK 0.253712

ACCL 0.575345

ACCM 0.753091

ACCN 0.308451

ACCP 0.185658

ACCQ 0.486812

ACCR 0.466885

ACCS 0.329013

ACCT 0.312156

ACCV 0.614047

ACCW 1.14022

ACCY 0.922227

ACDA -0.412893

ACDC 0.122658

ACDD -0.213976

ACDE -0.255307

ACDF 0.0946631

ACDG -0.580803

ACDH 0.256575

ACDI -0.184841

ACDK -0.00503945

ACDL -0.405576

ACDM 0.0274684

ACDN 0.0506375

ACDP -0.342452

ACDQ 0.00108933

ACDR 0.143548

ACDS -0.135499

ACDT -0.085279

ACDV -0.283758

ACDW 0.406699

ACDY 0.276827

ACEA -0.482341

ACEC 0.0657647

ACED -0.348965

ACEE -0.28663

ACEF 0.0247426

ACEG -0.836164

ACEH 0.166895

ACEI -0.218528

ACEK -0.0377429

ACEL -0.428205

ACEM 0.0607383

ACEN -0.130421

ACEP -0.477845

ACEQ -0.0120156

ACER 0.0914097

ACES -0.343511

ACET -0.232508

ACEV -0.34113

ACEW 0.426821

ACEY 0.147418

ACFA 0.0124445

ACFC 1.13668

ACFD 0.12167

ACFE 0.0299296

ACFF 0.777847

ACFG -0.308013

ACFH 0.671066

ACFI 0.477002

ACFK 0.110756

ACFL 0.188426

ACFM 0.877196

ACFN 0.218026

ACFP 0.118615

ACFQ 0.345433

ACFR 0.289375

ACFS 0.149212

ACFT 0.233692

ACFV 0.299469

ACFW 1.09283

ACFY 0.761313

ACGA -0.910948

ACGC -0.118646

ACGD -0.555896

ACGE -0.691122

ACGF -0.224926

ACGG -1.75282

ACGH -0.322469

ACGI -0.514674

ACGK -0.542305

ACGL -0.705932

ACGM -0.259859

ACGN -0.431914

ACGP -0.798194

ACGQ -0.413363

ACGR -0.384571

ACGS -0.560607

ACGT -0.437178

ACGV -0.590993

ACGW -0.0108778

ACGY -0.116973

ACHA -0.0391448

ACHC 0.693101

ACHD 0.311212

ACHE 0.182428

ACHF 0.67023

ACHG -0.33093

ACHH 0.763303

ACHI 0.317523

ACHK 0.0211158

ACHL 0.256498

ACHM 0.571937

ACHN 0.243231

ACHP 0.0488331

ACHQ 0.231652

ACHR 0.321302

ACHS 0.201564

ACHT 0.270775

ACHV 0.259376

ACHW 0.951908

ACHY 0.72775

ACIA -0.188946

ACIC 0.72046

ACID -0.18561

ACIE -0.205559

ACIF 0.489793

ACIG -0.568701

ACIH 0.37148

ACII 0.281781

ACIK -0.133032

ACIL -0.077615

ACIM 0.693778

ACIN -0.0256152

ACIP -0.144681

ACIQ 0.102468

ACIR 0.0549061

ACIS -0.125835

ACIT 0.0231435

ACIV 0.0782695

ACIW 0.836124

ACIY 0.53564

ACKA -0.456054

ACKC 0.203963

ACKD -0.0546181

ACKE -0.0507333

ACKF 0.0920689

ACKG -0.73691

ACKH 0.0026679

ACKI -0.149739

ACKK -0.186168

ACKL -0.376914

ACKM 0.0402665

ACKN -0.0951772

ACKP -0.400293

ACKQ 0.0129504

ACKR -0.183244

ACKS -0.258048

ACKT -0.163941

ACKV -0.269265

ACKW 0.319759

ACKY 0.233169

ACLA -0.498522

ACLC 0.727525

ACLD -0.426042

ACLE -0.425866

ACLF 0.200686

ACLG -0.770244

ACLH 0.25966

ACLI -0.0796616

ACLK -0.352824

ACLL -0.412009

ACLM 0.468755

ACLN -0.259268

ACLP -0.32592

ACLQ -0.027745

ACLR -0.148462

ACLS -0.383109

ACLT -0.262575

ACLV -0.277413

ACLW 0.73253

ACLY 0.24186

ACMA 0.191841

ACMC 0.816795

ACMD 0.0884154

ACME 0.103943

ACMF 0.880018

ACMG -0.433279

ACMH 0.46245

ACMI 0.647207

ACMK 0.128756

ACML 0.466021

ACMM 0.904171

ACMN 0.128137

ACMP -0.058501

ACMQ 0.283559

ACMR 0.324726

ACMS 0.0767586

ACMT 0.250141

ACMV 0.504843

ACMW 0.865326

ACMY 0.821684

ACNA -0.32742

ACNC 0.325465

ACND -0.00770259

ACNE -0.122954

ACNF 0.227771

ACNG -0.469301

ACNH 0.263034

ACNI -0.0533624

ACNK -0.0152717

ACNL -0.246075

ACNM 0.13881

ACNN 0.208785

ACNP -0.163948

ACNQ 0.171102

ACNR 0.08726

ACNS -0.0305445

ACNT 0.0209596

ACNV -0.154028

ACNW 0.601563

ACNY 0.404572

ACPA -0.310393

ACPC 0.287218

ACPD -0.0923829

ACPE -0.0763125

ACPF 0.276816

ACPG -0.49582

ACPH 0.225848

ACPI -0.0428212

ACPK -0.154527

ACPL -0.203031

ACPM 0.207062

ACPN -0.0149839

ACPP -0.18462

ACPQ 0.0975993

ACPR 0.0621371

ACPS -0.0854931

ACPT -0.030345

ACPV -0.0611427

ACPW 0.657031

ACPY 0.373271

ACQA -0.15756

ACQC 0.469015

ACQD -0.045377

ACQE -0.02439

ACQF 0.384332

ACQG -0.439327

ACQH 0.218632

ACQI 0.0701575

ACQK 0.0449638

ACQL -0.052326

ACQM 0.235635

ACQN 0.138633

ACQP -0.190148

ACQQ 0.355967

ACQR 0.183185

ACQS 0.00940585

ACQT 0.0921943

ACQV -0.0148125

ACQW 0.619462

ACQY 0.489235

ACRA -0.281157

ACRC 0.393828

ACRD 0.108729

ACRE 0.0945859

ACRF 0.32855

ACRG -0.569331

ACRH 0.299906

ACRI 0.0562847

ACRK -0.18105

ACRL -0.161273

ACRM 0.350055

ACRN 0.0220501

ACRP -0.146904

ACRQ 0.192794

ACRR 0.137041

ACRS -0.0708985

ACRT -0.0369549

ACRV -0.0722139

ACRW 0.589949

ACRY 0.428181

ACSA -0.450632

ACSC 0.468043

ACSD -0.172842

ACSE -0.220499

ACSF 0.16909

ACSG -0.518396

ACSH 0.216582

ACSI -0.162484

ACSK -0.18777

ACSL -0.343466

ACSM 0.125723

ACSN -0.0164258

ACSP -0.340534

ACSQ 0.0880792

ACSR -0.0182843

ACSS -0.0725455

ACST -0.0533142

ACSV -0.25454

ACSW 0.446223

ACSY 0.257054

ACTA -0.349545

ACTC 0.483731

ACTD -0.123325

ACTE -0.143461

ACTF 0.263451

ACTG -0.5126

ACTH 0.273004

ACTI 0.00360274

ACTK -0.138165

ACTL -0.255964

ACTM 0.282216

ACTN 0.0344408

ACTP -0.207595

ACTQ 0.148853

ACTR 0.0365176

ACTS -0.0804453

ACTT 0.021091

ACTV -0.1121

ACTW 0.563888

ACTY 0.356929

ACVA -0.339074

ACVC 0.805517

ACVD -0.308894

ACVE -0.327309

ACVF 0.328207

ACVG -0.680453

ACVH 0.266311

ACVI 0.070457

ACVK -0.255628

ACVL -0.269384

ACVM 0.522601

ACVN -0.162493

ACVP -0.255127

ACVQ -0.011611

ACVR -0.0898583

ACVS -0.261144

ACVT -0.127506

ACVV -0.0863094

ACVW 0.727199

ACVY 0.336729

ACWA 0.330944

ACWC 1.14461

ACWD 0.358663

ACWE 0.340074

ACWF 1.12573

ACWG 0.0549018

ACWH 0.747977

ACWI 0.879327

ACWK 0.370275

ACWL 0.740105

ACWM 0.960859

ACWN 0.533814

ACWP 0.369152

ACWQ 0.635159

ACWR 0.649452

ACWS 0.37547

ACWT 0.461581

ACWV 0.762817

ACWW 1.30451

ACWY 1.21543

ACYA 0.0369811

ACYC 1.12661

ACYD 0.248063

ACYE 0.123267

ACYF 0.788454

ACYG -0.146077

ACYH 0.681515

ACYI 0.472082

ACYK 0.22214

ACYL 0.210596

ACYM 0.795744

ACYN 0.380033

ACYP 0.262148

ACYQ 0.473746

ACYR 0.428513

ACYS 0.20177

ACYT 0.30688

ACYV 0.319078

ACYW 1.16637

ACYY 0.855512

ADAA -0.75691

ADAC 0.0307553

ADAD 3.97618

ADAE -0.333537

ADAF -0.240806

ADAG -1.12838

ADAH -0.1705

ADAI -0.491172

ADAK -0.613309

ADAL -0.810074

ADAM -0.00848603

ADAN -0.449075

ADAP -0.825277

ADAQ -0.382395

ADAR -0.470735

ADAS -0.664716

ADAT -0.471107

ADAV -0.560041

ADAW 0.179791

ADAY -0.168328

ADCA 0.0764616

ADCC 2.08795

ADCD -0.0337641

ADCE -0.127796

ADCF 0.692433

ADCG -0.193888

ADCH 0.624329

ADCI 0.413113

ADCK 0.0821495

ADCL 0.315067

ADCM 0.61773

ADCN 0.153048

ADCP 0.0373585

ADCQ 0.331515

ADCR 0.289473

ADCS 0.145343

ADCT 0.13419

ADCV 0.380258

ADCW 1.00634

ADCY 0.729098

ADDA -0.712108

ADDC -0.0504053

ADDD -0.368515

ADDE -0.490535

ADDF -0.174365

ADDG -0.850235

ADDH 0.0384429

ADDI -0.460081

ADDK -0.305837

ADDL -0.713133

ADDM -0.165637

ADDN -0.226409

ADDP -0.553308

ADDQ -0.247658

ADDR -0.149125

ADDS -0.414784

ADDT -0.383673

ADDV -0.573675

ADDW 0.217357

ADDY 0.0233989

ADEA -0.755648

ADEC -0.113834

ADED -0.442801

ADEE -0.556277

ADEF -0.233109

ADEG -1.07483

ADEH -0.0572393

ADEI -0.505695

ADEK -0.326554

ADEL -0.73363

ADEM -0.145488

ADEN -0.383378

ADEP -0.69494

ADEQ -0.28021

ADER -0.205505

ADES -0.566699

ADET -0.50656

ADEV -0.641046

ADEW 0.225724

ADEY -0.118951

ADFA -0.283237

ADFC 0.708097

ADFD -0.13678

ADFE -0.254123

ADFF 0.472289

ADFG -0.558235

ADFH 0.44618

ADFI 0.167913

ADFK -0.162163

ADFL -0.137595

ADFM 0.634182

ADFN -0.0232708

ADFP -0.126657

ADFQ 0.109513

ADFR 0.0222149

ADFS -0.0846329

ADFT -0.0573211

ADFV -0.0105417

ADFW 0.872242

ADFY 0.478903

ADGA -1.18145

ADGC -0.305525

ADGD -0.637599

ADGE -0.945021

ADGF -0.478904

ADGG -1.91238

ADGH -0.495289

ADGI -0.763067

ADGK -0.806885

ADGL -1.00538

ADGM -0.444392

ADGN -0.680539

ADGP -1.00383

ADGQ -0.62771

ADGR -0.636934

ADGS -0.825902

ADGT -0.714526

ADGV -0.858367

ADGW -0.174778

ADGY -0.377833

ADHA -0.266841

ADHC 0.556445

ADHD 0.0902107

ADHE -0.0394719

ADHF 0.443465

ADHG -0.512452

ADHH 0.587857

ADHI 0.0999832

ADHK -0.169694

ADHL -0.0158679

ADHM 0.41922

ADHN 0.0546079

ADHP -0.12046

ADHQ 0.0569744

ADHR 0.109561

ADHS 0.00865293

ADHT 0.0542319

ADHV 0.0131807

ADHW 0.80031

ADHY 0.512348

ADIA -0.525872

ADIC 0.494653

ADID -0.462232

ADIE -0.487584

ADIF 0.182996

ADIG -0.827913

ADIH 0.13306

ADII -0.0452304

ADIK -0.426232

ADIL -0.414537

ADIM 0.418704

ADIN -0.297578

ADIP -0.389267

ADIQ -0.155393

ADIR -0.237444

ADIS -0.4138

ADIT -0.269939

ADIV -0.266021

ADIW 0.603435

ADIY 0.230248

ADKA -0.738438

ADKC 0.0153198

ADKD -0.200979

ADKE -0.299412

ADKF -0.174221

ADKG -0.989298

ADKH -0.188127

ADKI -0.421

ADKK -0.452715

ADKL -0.680938

ADKM -0.154596

ADKN -0.353725

ADKP -0.634921

ADKQ -0.257104

ADKR -0.45046

ADKS -0.512034

ADKT -0.462975

ADKV -0.569962

ADKW 0.139153

ADKY -0.0409963

ADLA -0.841281

ADLC 0.404546

ADLD -0.552793

ADLE -0.695822

ADLF -0.136528

ADLG -1.06369

ADLH 0.00547338

ADLI -0.413598

ADLK -0.674736

ADLL -0.781701

ADLM 0.183815

ADLN -0.55317

ADLP -0.594554

ADLQ -0.337568

ADLR -0.469686

ADLS -0.695642

ADLT -0.593457

ADLV -0.620343

ADLW 0.458408

ADLY -0.0769043

ADMA -0.0438974

ADMC 0.691678

ADMD -0.0919836

ADME -0.100405

ADMF 0.641661

ADMG -0.604739

ADMH 0.314885

ADMI 0.39355

ADMK -0.0714693

ADML 0.1769

ADMM 0.732514

ADMN -0.0496726

ADMP -0.217119

ADMQ 0.11202

ADMR 0.115915

ADMS -0.128604

ADMT 0.041311

ADMV 0.238352

ADMW 0.724652

ADMY 0.593719

ADNA -0.596051

ADNC 0.161623

ADND -0.243977

ADNE -0.326959

ADNF -0.0251787

ADNG -0.714118

ADNH 0.0684104

ADNI -0.320475

ADNK -0.254584

ADNL -0.532877

ADNM -0.0363855

ADNN -0.0631418

ADNP -0.338437

ADNQ -0.029952

ADNR -0.169885

ADNS -0.232264

ADNT -0.216118

ADNV -0.43792

ADNW 0.420062

ADNY 0.135998

ADPA -0.584977

ADPC 0.114907

ADPD -0.159652

ADPE -0.340601

ADPF 0.0135803

ADPG -0.746377

ADPH 0.0344064

ADPI -0.311912

ADPK -0.412776

ADPL -0.504897

ADPM 0.0227158

ADPN -0.257167

ADPP -0.410995

ADPQ -0.133628

ADPR -0.199822

ADPS -0.338358

ADPT -0.297651

ADPV -0.35946

ADPW 0.467858

ADPY 0.109204

ADQA -0.422507

ADQC 0.315473

ADQD -0.243652

ADQE -0.255176

ADQF 0.128092

ADQG -0.668249

ADQH 0.0509071

ADQI -0.181234

ADQK -0.217755

ADQL -0.341214

ADQM 0.0669599

ADQN -0.0942621

ADQP -0.388321

ADQQ 0.107643

ADQR -0.0560143

ADQS -0.237704

ADQT -0.149878

ADQV -0.278731

ADQW 0.457333

ADQY 0.244256

ADRA -0.582253

ADRC 0.208504

ADRD -0.0193172

ADRE -0.238366

ADRF 0.0577526

ADRG -0.802089

ADRH 0.095156

ADRI -0.229733

ADRK -0.440075

ADRL -0.467726

ADRM 0.144006

ADRN -0.220077

ADRP -0.374443

ADRQ -0.0498865

ADRR -0.143725

ADRS -0.343709

ADRT -0.308863

ADRV -0.370046

ADRW 0.404521

ADRY 0.151925

ADSA -0.730198

ADSC 0.189937

ADSD -0.0595827

ADSE -0.496135

ADSF -0.116051

ADSG -0.785872

ADSH -0.00139832

ADSI -0.432515

ADSK -0.476028

ADSL -0.666415

ADSM -0.073674

ADSN -0.265174

ADSP -0.56833

ADSQ -0.183951

ADSR -0.29771

ADSS -0.379519

ADST -0.370024

ADSV -0.570214

ADSW 0.254422

ADSY -0.00794744

ADTA -0.65204

ADTC 0.285746

ADTD -0.253993

ADTE -0.417374

ADTF -0.0327749

ADTG -0.793233

ADTH 0.0521653

ADTI -0.294512

ADTK -0.421939

ADTL -0.568859

ADTM 0.0715692

ADTN -0.230445

ADTP -0.452174

ADTQ -0.13153

ADTR -0.248888

ADTS -0.381187

ADTT -0.259205

ADTV -0.424155

ADTW 0.363973

ADTY 0.0724998

ADVA -0.531759

ADVC 0.414222

ADVD 0.0707095

ADVE -0.516121

ADVF 0.00254226

ADVG -0.860459

ADVH 0.0336843

ADVI -0.266466

ADVK -0.567977

ADVL -0.631237

ADVM 0.264179

ADVN -0.406544

ADVP -0.527038

ADVQ -0.179161

ADVR -0.370402

ADVS -0.566411

ADVT -0.44126

ADVV -0.437332

ADVW 0.482417

ADVY 0.0182865

ADWA 0.12046

ADWC 1.01024

ADWD 0.171205

ADWE 0.148968

ADWF 0.899324

ADWG -0.121352

ADWH 0.610713

ADWI 0.641453

ADWK 0.183004

ADWL 0.472291

ADWM 0.812357

ADWN 0.360983

ADWP 0.207978

ADWQ 0.47008

ADWR 0.456079

ADWS 0.187541

ADWT 0.275272

ADWV 0.51272

ADWW 1.1607

ADWY 0.991156

ADYA -0.256642

ADYC 0.903394

ADYD 0.113002

ADYE -0.154134

ADYF 0.504769

ADYG -0.403733

ADYH 0.476194

ADYI 0.179912

ADYK -0.0569823

ADYL -0.108882

ADYM 0.574525

ADYN 0.113464

ADYP 0.0188928

ADYQ 0.2105

ADYR 0.154258

ADYS -0.0796189

ADYT 0.0281701

ADYV 0.00922942

ADYW 0.94326

ADYY 0.563619

AEAA -0.723608

AEAC -0.0638869

AEAD -0.426057

AEAE 4.0981

AEAF -0.346383

AEAG -1.26284

AEAH -0.232019

AEAI -0.593263

AEAK -0.533461

AEAL -0.816739

AEAM -0.1231

AEAN -0.599875

AEAP -0.916741

AEAQ -0.115593

AEAR -0.580877

AEAS -0.785822

AEAT -0.679967

AEAV -0.694141

AEAW 0.0888517

AEAY -0.264185

AECA -0.0122883

AECC 1.99312

AECD -0.141754

AECE -0.158504

AECF 0.611751

AECG -0.272449

AECH 0.55152

AECI 0.334766

AECK 0.00099134

AECL 0.22198

AECM 0.545683

AECN 0.0761399

AECP -0.0377843

AECQ 0.255759

AECR 0.208058

AECS 0.0608275

AECT 0.0539854

AECV 0.288115

AECW 0.933864

AECY 0.646166

AEDA -0.79531

AEDC -0.124936

AEDD -0.547202

AEDE -0.545439

AEDF -0.274551

AEDG -0.932767

AEDH -0.0523479

AEDI -0.554441

AEDK -0.358938

AEDL -0.81252

AEDM -0.247143

AEDN -0.316779

AEDP -0.659962

AEDQ -0.336237

AEDR -0.251328

AEDS -0.513558

AEDT -0.476574

AEDV -0.675559

AEDW 0.135071

AEDY -0.0980206

AEEA -0.859452

AEEC -0.185834

AEED -0.71387

AEEE -0.340717

AEEF -0.341014

AEEG -1.15739

AEEH -0.117487

AEEI -0.589022

AEEK -0.405648

AEEL -0.829386

AEEM -0.231926

AEEN -0.501078

AEEP -0.79732

AEEQ -0.331175

AEER -0.315468

AEES -0.719751

AEET -0.601008

AEEV -0.742189

AEEW 0.136677

AEEY -0.215339

AEFA -0.371119

AEFC 0.623914

AEFD -0.245749

AEFE -0.193731

AEFF 0.37855

AEFG -0.644233

AEFH 0.359906

AEFI 0.0692427

AEFK -0.263334

AEFL -0.242967

AEFM 0.557579

AEFN -0.123633

AEFP -0.215797

AEFQ 0.00379777

AEFR -0.0782032

AEFS -0.18888

AEFT -0.147179

AEFV -0.114709

AEFW 0.781382

AEFY 0.377

AEGA -1.27291

AEGC -0.38046

AEGD -0.881718

AEGE -0.868634

AEGF -0.570278

AEGG -1.98731

AEGH -0.591391

AEGI -0.880855

AEGK -0.878482

AEGL -1.08333

AEGM -0.536679

AEGN -0.766684

AEGP -1.08992

AEGQ -0.718505

AEGR -0.737779

AEGS -0.926064

AEGT -0.806859

AEGV -0.967704

AEGW -0.25095

AEGY -0.476795

AEHA -0.35197

AEHC 0.484806

AEHD -0.00780272

AEHE -0.0760086

AEHF 0.354591

AEHG -0.597573

AEHH 0.509102

AEHI 0.00886822

AEHK -0.246218

AEHL -0.104763

AEHM 0.34702

AEHN -0.0328655

AEHP -0.201659

AEHQ -0.0277023

AEHR 0.0540836

AEHS -0.0980546

AEHT -0.0288517

AEHV -0.0818696

AEHW 0.727496

AEHY 0.415992

AEIA -0.6133

AEIC 0.406682

AEID -0.560504

AEIE -0.519737

AEIF 0.0989316

AEIG -0.905324

AEIH 0.0381885

AEII -0.144399

AEIK -0.484071

AEIL -0.514217

AEIM 0.371429

AEIN -0.384902

AEIP -0.494431

AEIQ -0.252417

AEIR -0.3251

AEIS -0.509365

AEIT -0.373527

AEIV -0.377477

AEIW 0.518204

AEIY 0.157228

AEKA -0.843849

AEKC -0.0605474

AEKD -0.452585

AEKE -0.38584

AEKF -0.258171

AEKG -1.09496

AEKH -0.279967

AEKI -0.538573

AEKK -0.376353

AEKL -0.784425

AEKM -0.241466

AEKN -0.449012

AEKP -0.723539

AEKQ -0.324131

AEKR -0.498457

AEKS -0.626466

AEKT -0.534467

AEKV -0.649717

AEKW 0.0592718

AEKY -0.143872

AELA -0.942322

AELC 0.310976

AELD -0.85386

AELE -0.736236

AELF -0.237415

AELG -1.16536

AELH -0.0953181

AELI -0.518751

AELK -0.762256

AELL -0.907156

AELM 0.0837586

AELN -0.648721

AELP -0.706433

AELQ -0.426462

AELR -0.570978

AELS -0.791142

AELT -0.615943

AELV -0.730549

AELW 0.360986

AELY -0.187524

AEMA -0.147183

AEMC 0.6177

AEMD -0.189603

AEME -0.122307

AEMF 0.546065

AEMG -0.672878

AEMH 0.252368

AEMI 0.2967

AEMK -0.167955

AEML 0.078913

AEMM 0.645351

AEMN -0.132097

AEMP -0.292056

AEMQ 0.035068

AEMR 0.0334003

AEMS -0.209532

AEMT -0.0454605

AEMV 0.14584

AEMW 0.650699

AEMY 0.512804

AENA -0.692632

AENC 0.0828261

AEND -0.364409

AENE -0.306585

AENF -0.119576

AENG -0.807101

AENH -0.0031867

AENI -0.421847

AENK -0.363294

AENL -0.630775

AENM -0.100603

AENN -0.148242

AENP -0.467581

AENQ -0.159632

AENR -0.268044

AENS -0.407398

AENT -0.331029

AENV -0.525641

AENW 0.337859

AENY 0.0431883

AEPA -0.698314

AEPC 0.0368493

AEPD -0.409557

AEPE 0.0323024

AEPF -0.0677538

AEPG -0.837739

AEPH -0.0478489

AEPI -0.396737

AEPK -0.516914

AEPL -0.595112

AEPM -0.0581348

AEPN -0.336289

AEPP -0.480861

AEPQ -0.261785

AEPR -0.276739

AEPS -0.435659

AEPT -0.411864

AEPV -0.457484

AEPW 0.385938

AEPY 0.0149083

AEQA -0.517505

AEQC 0.238194

AEQD -0.385961

AEQE -0.290691

AEQF 0.0490515

AEQG -0.758847

AEQH -0.0346913

AEQI -0.272519

AEQK -0.304293

AEQL -0.450361

AEQM -0.00226307

AEQN -0.185776

AEQP -0.477101

AEQQ 0.0238161

AEQR -0.169774

AEQS -0.329745

AEQT -0.264021

AEQV -0.384136

AEQW 0.376786

AEQY 0.147488

AERA -0.674862

AERC 0.131546

AERD -0.291508

AERE -0.131999

AERF -0.0423017

AERG -0.898525

AERH 0.0159733

AERI -0.324061

AERK -0.545857

AERL -0.592305

AERM 0.0602772

AERN -0.323097

AERP -0.468528

AERQ -0.13365

AERR -0.237561

AERS -0.445938

AERT -0.409274

AERV -0.461737

AERW 0.320974

AERY 0.0602493

AESA -0.777519

AESC 0.112326

AESD -0.563272

AESE 0.158552

AESF -0.207556

AESG -0.88747

AESH -0.0957339

AESI -0.537816

AESK -0.574354

AESL -0.576734

AESM -0.1488

AESN -0.364424

AESP -0.6717

AESQ -0.274649

AESR -0.420282

AESS -0.492153

AEST -0.417171

AESV -0.661196

AESW 0.167238

AESY -0.111193

AETA -0.737105

AETC 0.19891

AETD -0.434875

AETE -0.00975275

AETF -0.121623

AETG -0.882802

AETH -0.0346868

AETI -0.394918

AETK -0.538196

AETL -0.654598

AETM -0.0136316

AETN -0.355307

AETP -0.549697

AETQ -0.237109

AETR -0.354997

AETS -0.50174

AETT -0.289871

AETV -0.496953

AETW 0.276137

AETY -0.0253797

AEVA -0.693916

AEVC 0.328663

AEVD -0.650334

AEVE -0.404969

AEVF -0.0979798

AEVG -0.98742

AEVH -0.0727329

AEVI -0.381124

AEVK -0.661551

AEVL -0.718018

AEVM 0.161137

AEVN -0.511774

AEVP -0.611245

AEVQ -0.313638

AEVR -0.463166

AEVS -0.651203

AEVT -0.553329

AEVV -0.574589

AEVW 0.39585

AEVY -0.0811102

AEWA 0.0235689

AEWC 0.937736

AEWD 0.089391

AEWE 0.0738757

AEWF 0.813032

AEWG -0.200079

AEWH 0.544796

AEWI 0.556459

AEWK 0.103959

AEWL 0.374175

AEWM 0.737083

AEWN 0.295569

AEWP 0.13037

AEWQ 0.3872

AEWR 0.379207

AEWS 0.112365

AEWT 0.190701

AEWV 0.419502

AEWW 1.08577

AEWY 0.904401

AEYA -0.309402

AEYC 0.817575

AEYD -0.115684

AEYE -0.194117

AEYF 0.386805

AEYG -0.496436

AEYH 0.370693

AEYI 0.0812814

AEYK -0.132186

AEYL -0.221929

AEYM 0.486251

AEYN 0.0433881

AEYP -0.0758691

AEYQ 0.16548

AEYR 0.0506134

AEYS -0.168897

AEYT -0.0650697

AEYV -0.0885494

AEYW 0.863232

AEYY 0.466364

AFAA -0.956763

AFAC -0.106368

AFAD -0.915326

AFAE -0.928383

AFAF 4.6952

AFAG -1.29307

AFAH -0.30323

AFAI -0.389527

AFAK -0.86722

AFAL -0.466021

AFAM 0.00848365

AFAN -0.726731

AFAP -0.983208

AFAQ -0.577233

AFAR -0.658238

AFAS -0.8474

AFAT -0.821723

AFAV -0.643726

AFAW 0.210089

AFAY 0.298762

AFCA -0.0659821

AFCC 1.93782

AFCD -0.188075

AFCE -0.255423

AFCF 0.596722

AFCG -0.321318

AFCH 0.505919

AFCI 0.277661

AFCK -0.0492311

AFCL 0.166295

AFCM 0.49891

AFCN 0.0300574

AFCP -0.0849097

AFCQ 0.207928

AFCR 0.15769

AFCS 0.0055654

AFCT 0.00693059

AFCV 0.240335

AFCW 0.88764

AFCY 0.593536

AFDA -0.851433

AFDC -0.160653

AFDD -0.653092

AFDE -0.698651

AFDF -0.215212

AFDG -0.995776

AFDH -0.100093

AFDI -0.612569

AFDK -0.471815

AFDL -0.869839

AFDM -0.296675

AFDN -0.380816

AFDP -0.719926

AFDQ -0.397391

AFDR -0.318181

AFDS -0.58836

AFDT -0.543936

AFDV -0.718391

AFDW 0.0956852

AFDY -0.165876

AFEA -0.936745

AFEC -0.240276

AFED -0.774818

AFEE -0.740903

AFEF -0.255713

AFEG -1.22274

AFEH -0.197539

AFEI -0.656699

AFEK -0.508162

AFEL -0.884763

AFEM -0.273639

AFEN -0.544839

AFEP -0.840656

AFEQ -0.422261

AFER -0.374196

AFES -0.773821

AFET -0.678987

AFEV -0.804779

AFEW 0.0853641

AFEY -0.251969

AFFA -0.443587

AFFC 0.573141

AFFD -0.297147

AFFE -0.391755

AFFF 0.416903

AFFG -0.700934

AFFH 0.307116

AFFI 0.068917

AFFK -0.314909

AFFL -0.317382

AFFM 0.490924

AFFN -0.177356

AFFP -0.271163

AFFQ -0.0475523

AFFR -0.125501

AFFS -0.278571

AFFT -0.198664

AFFV -0.131831

AFFW 0.729889

AFFY 0.326186

AFGA -1.31186

AFGC -0.431384

AFGD -0.97357

AFGE -1.09914

AFGF -0.379246

AFGG -2.0386

AFGH -0.64288

AFGI -0.844422

AFGK -0.951701

AFGL -1.13887

AFGM -0.57188

AFGN -0.828793

AFGP -1.13888

AFGQ -0.78585

AFGR -0.797437

AFGS -0.975389

AFGT -0.873978

AFGV -0.984064

AFGW -0.310555

AFGY -0.564873

AFHA -0.40057

AFHC 0.437442

AFHD -0.0586455

AFHE -0.184221

AFHF 0.361578

AFHG -0.64032

AFHH 0.458077

AFHI -0.0418587

AFHK -0.311827

AFHL -0.155784

AFHM 0.298781

AFHN -0.0806561

AFHP -0.252532

AFHQ -0.0688391

AFHR -0.0261595

AFHS -0.152061

AFHT -0.0858972

AFHV -0.126533

AFHW 0.674469

AFHY 0.357863

AFIA -0.674497

AFIC 0.35344

AFID -0.615811

AFIE -0.640594

AFIF 0.506449

AFIG -0.975677

AFIH 0.0198808

AFII -0.202587

AFIK -0.555179

AFIL -0.627175

AFIM 0.260937

AFIN -0.448404

AFIP -0.543494

AFIQ -0.306344

AFIR -0.401312

AFIS -0.555371

AFIT -0.437672

AFIV -0.36261

AFIW 0.463683

AFIY 0.0787756

AFKA -0.901808

AFKC -0.110485

AFKD -0.517514

AFKE -0.519983

AFKF -0.258781

AFKG -1.1293

AFKH -0.327204

AFKI -0.592115

AFKK -0.632182

AFKL -0.838469

AFKM -0.286344

AFKN -0.498483

AFKP -0.77152

AFKQ -0.390659

AFKR -0.59043

AFKS -0.679367

AFKT -0.596668

AFKV -0.719537

AFKW 0.0129976

AFKY -0.193737

AFLA -0.962432

AFLC 0.253562

AFLD -0.889813

AFLE -0.864059

AFLF -0.250735

AFLG -1.21142

AFLH -0.171242

AFLI -0.586519

AFLK -0.809035

AFLL -0.982759

AFLM 0.0170591

AFLN -0.702615

AFLP -0.763719

AFLQ -0.478642

AFLR -0.603287

AFLS -0.820784

AFLT -0.755582

AFLV -0.812775

AFLW 0.294244

AFLY -0.25445

AFMA -0.198089

AFMC 0.574324

AFMD -0.242715

AFME -0.241662

AFMF 0.553929

AFMG -0.729529

AFMH 0.212362

AFMI 0.245061

AFMK -0.210588

AFML 0.0734618

AFMM 0.590644

AFMN -0.17595

AFMP -0.34223

AFMQ -0.018218

AFMR -0.0148089

AFMS -0.250235

AFMT -0.0987403

AFMV 0.081593

AFMW 0.606514

AFMY 0.451371

AFNA -0.757381

AFNC 0.0329556

AFND -0.416515

AFNE -0.515825

AFNF -0.114791

AFNG -0.860249

AFNH -0.0571804

AFNI -0.452091

AFNK -0.439746

AFNL -0.64463

AFNM -0.163984

AFNN -0.207646

AFNP -0.527848

AFNQ -0.222289

AFNR -0.326661

AFNS -0.422458

AFNT -0.388168

AFNV -0.596445

AFNW 0.289412

AFNY -0.0232713

AFPA -0.740849

AFPC -0.0147893

AFPD -0.510919

AFPE -0.504398

AFPF -0.0586171

AFPG -0.885571

AFPH -0.0969694

AFPI -0.457328

AFPK -0.563154

AFPL -0.655392

AFPM -0.109697

AFPN -0.389473

AFPP -0.549944

AFPQ -0.275039

AFPR -0.34668

AFPS -0.503036

AFPT -0.449712

AFPV -0.49142

AFPW 0.347713

AFPY -0.044106

AFQA -0.577368

AFQC 0.192181

AFQD -0.436403

AFQE -0.425398

AFQF 0.0884459

AFQG -0.805853

AFQH -0.0803471

AFQI -0.33075

AFQK -0.359565

AFQL -0.490602

AFQM -0.0602913

AFQN -0.240703

AFQP -0.527711

AFQQ -0.0302043

AFQR -0.209875

AFQS -0.398779

AFQT -0.324593

AFQV -0.442587

AFQW 0.319213

AFQY 0.075578

AFRA -0.722029

AFRC 0.0801151

AFRD -0.346106

AFRE -0.373276

AFRF -0.0664325

AFRG -0.942219

AFRH -0.0432403

AFRI -0.366807

AFRK -0.594312

AFRL -0.638281

AFRM 0.0139806

AFRN -0.364383

AFRP -0.524091

AFRQ -0.203236

AFRR -0.293743

AFRS -0.505429

AFRT -0.463131

AFRV -0.528391

AFRW 0.267192

AFRY -0.0126972

AFSA -0.923892

AFSC 0.0600731

AFSD -0.597619

AFSE -0.67606

AFSF 0.28639

AFSG -0.91669

AFSH -0.113947

AFSI -0.574093

AFSK -0.63128

AFSL -0.836308

AFSM -0.18701

AFSN -0.426623

AFSP -0.722747

AFSQ -0.332598

AFSR -0.449952

AFSS -0.544204

AFST -0.512185

AFSV -0.695428

AFSW 0.113917

AFSY -0.214547

AFTA -0.788202

AFTC 0.15291

AFTD -0.557134

AFTE -0.598664

AFTF 0.331355

AFTG -0.919335

AFTH -0.0921075

AFTI -0.400667

AFTK -0.591668

AFTL -0.742211

AFTM -0.0251868

AFTN -0.392756

AFTP -0.597924

AFTQ -0.261772

AFTR -0.41186

AFTS -0.542653

AFTT -0.435756

AFTV -0.586314

AFTW 0.238703

AFTY 0.00385523

AFVA -0.818208

AFVC 0.265019

AFVD -0.752773

AFVE -0.781476

AFVF 0.276345

AFVG -1.10878

AFVH -0.119572

AFVI -0.294019

AFVK -0.713024

AFVL -0.712009

AFVM 0.0996709

AFVN -0.586617

AFVP -0.672692

AFVQ -0.433585

AFVR -0.534774

AFVS -0.69757

AFVT -0.600816

AFVV -0.627631

AFVW 0.336047

AFVY -0.147884

AFWA -0.0266335

AFWC 0.891499

AFWD 0.042928

AFWE 0.0226901

AFWF 0.760725

AFWG -0.250366

AFWH 0.493058

AFWI 0.497237

AFWK 0.0540171

AFWL 0.317061

AFWM 0.693009

AFWN 0.232461

AFWP 0.0826838

AFWQ 0.340977

AFWR 0.32065

AFWS 0.0569561

AFWT 0.140498

AFWV 0.366884

AFWW 1.04179

AFWY 0.85082

AFYA -0.406254

AFYC 0.764762

AFYD -0.184621

AFYE -0.285412

AFYF 0.369975

AFYG -0.55014

AFYH 0.329054

AFYI 0.0143225

AFYK -0.198178

AFYL -0.245574

AFYM 0.426896

AFYN -0.0246007

AFYP -0.127377

AFYQ 0.0797126

AFYR 0.00236845

AFYS -0.19198

AFYT -0.121953

AFYV -0.152501

AFYW 0.768796

AFYY 0.419514

AGAA -0.35825

AGAC 0.135569

AGAD -0.584231

AGAE -0.626165

AGAF -0.0743976

AGAG 3.28746

AGAH -0.0736258

AGAI -0.338291

AGAK -0.541974

AGAL -0.641513

AGAM 0.160156

AGAN -0.383948

AGAP -0.716318

AGAQ -0.289568

AGAR -0.348069

AGAS -0.583551

AGAT -0.495475

AGAV -0.536528

AGAW 0.285153

AGAY -0.0568967

AGCA 0.188296

AGCC 2.21161

AGCD 0.0312726

AGCE -0.0275166

AGCF 0.802107

AGCG -0.0769088

AGCH 0.714767

AGCI 0.532065

AGCK 0.169432

AGCL 0.444601

AGCM 0.706089

AGCN 0.250826

AGCP 0.135445

AGCQ 0.426794

AGCR 0.393369

AGCS 0.251352

AGCT 0.237998

AGCV 0.497986

AGCW 1.09474

AGCY 0.840251

AGDA -0.52717

AGDC 0.0464995

AGDD -0.359238

AGDE -0.403724

AGDF -0.0465956

AGDG -0.657591

AGDH 0.152153

AGDI -0.328452

AGDK -0.15566

AGDL -0.547273

AGDM -0.058934

AGDN -0.0941198

AGDP -0.454086

AGDQ -0.116026

AGDR -0.0162244

AGDS -0.301663

AGDT -0.239154

AGDV -0.440649

AGDW 0.327895

AGDY 0.132669

AGEA -0.639508

AGEC -0.015095

AGED -0.488792

AGEE -0.435536

AGEF -0.113835

AGEG -0.968809

AGEH 0.0678589

AGEI -0.353189

AGEK -0.206651

AGEL -0.584901

AGEM -0.0331733

AGEN -0.2715

AGEP -0.590104

AGEQ -0.165761

AGER -0.0712271

AGES -0.472958

AGET -0.379108

AGEV -0.495622

AGEW 0.334846

AGEY 0.00484705

AGFA -0.12647

AGFC 0.819879

AGFD -0.0189247

AGFE -0.111892

AGFF 0.616781

AGFG -0.415885

AGFH 0.565089

AGFI 0.313607

AGFK -0.0291116

AGFL 0.00282383

AGFM 0.758474

AGFN 0.10226

AGFP -0.00104094

AGFQ 0.224028

AGFR 0.147787

AGFS 0.0116072

AGFT 0.0870442

AGFV 0.14338

AGFW 0.988627

AGFY 0.610105

AGGA -1.01487

AGGC -0.202598

AGGD -0.688778

AGGE -0.808751

AGGF -0.355228

AGGG -1.74225

AGGH -0.403407

AGGI -0.649365

AGGK -0.672297

AGGL -0.866311

AGGM -0.337989

AGGN -0.545175

AGGP -0.894443

AGGQ -0.515909

AGGR -0.510214

AGGS -0.691775

AGGT -0.582031

AGGV -0.74429

AGGW -0.0744469

AGGY -0.232361

AGHA -0.150416

AGHC 0.645189

AGHD 0.200287

AGHE 0.0905626

AGHF 0.562999

AGHG -0.389022

AGHH 0.690992

AGHI 0.210652

AGHK -0.06394

AGHL 0.117487

AGHM 0.513686

AGHN 0.161964

AGHP -0.0232928

AGHQ 0.159495

AGHR 0.230034

AGHS 0.0976274

AGHT 0.167765

AGHV 0.139258

AGHW 0.895481

AGHY 0.622204

AGIA -0.346251

AGIC 0.611069

AGID -0.323443

AGIE -0.354313

AGIF 0.321558

AGIG -0.681146

AGIH 0.258525

AGII 0.113823

AGIK -0.279206

AGIL -0.262106

AGIM 0.549607

AGIN -0.166091

AGIP -0.270676

AGIQ -0.0394354

AGIR -0.093787

AGIS -0.274135

AGIT -0.135063

AGIV -0.111051

AGIW 0.724107

AGIY 0.381148

AGKA -0.587648

AGKC 0.114818

AGKD -0.202987

AGKE -0.223288

AGKF -0.0464218

AGKG -0.823491

AGKH -0.0788636

AGKI -0.298658

AGKK -0.338243

AGKL -0.528529

AGKM -0.0446558

AGKN -0.225539

AGKP -0.510545

AGKQ -0.114353

AGKR -0.316523

AGKS -0.407813

AGKT -0.307852

AGKV -0.418398

AGKW 0.242063

AGKY 0.088743

AGLA -0.668297

AGLC 0.536788

AGLD -0.583917

AGLE -0.584567

AGLF 0.0186677

AGLG -0.816366

AGLH 0.122889

AGLI -0.259315

AGLK -0.519078

AGLL -0.61898

AGLM 0.32461

AGLN -0.400986

AGLP -0.470681

AGLQ -0.18273

AGLR -0.307423

AGLS -0.555669

AGLT -0.440646

AGLV -0.468165

AGLW 0.590812

AGLY 0.0677862

AGMA 0.0679195

AGMC 0.782304

AGMD 0.00017095

AGME 0.00815845

AGMF 0.759573

AGMG -0.502082

AGMH 0.40904

AGMI 0.51893

AGMK 0.0371814

AGML 0.316185

AGMM 0.828229

AGMN 0.0580492

AGMP -0.120636

AGMQ 0.213431

AGMR 0.227807

AGMS -0.00745082

AGMT 0.156024

AGMV 0.363977

AGMW 0.815285

AGMY 0.712471

AGNA -0.4679

AGNC 0.262173

AGND -0.143293

AGNE -0.262518

AGNF 0.112983

AGNG -0.502046

AGNH 0.177753

AGNI -0.192189

AGNK -0.156385

AGNL -0.395621

AGNM 0.0666394

AGNN 0.0770826

AGNP -0.264857

AGNQ 0.0455737

AGNR -0.0435073

AGNS -0.179269

AGNT -0.11968

AGNV -0.302487

AGNW 0.523004

AGNY 0.266961

AGPA -0.469313

AGPC 0.206578

AGPD -0.246888

AGPE -0.234626

AGPF 0.14318

AGPG -0.434858

AGPH 0.145826

AGPI -0.17901

AGPK -0.310519

AGPL -0.360295

AGPM 0.127896

AGPN -0.134193

AGPP -0.292819

AGPQ -0.0118439

AGPR -0.06885

AGPS -0.19822

AGPT -0.166716

AGPV -0.215617

AGPW 0.574734

AGPY 0.237014

AGQA -0.307031

AGQC 0.410987

AGQD -0.167693

AGQE -0.160016

AGQF 0.259123

AGQG -0.409885

AGQH 0.153236

AGQI -0.0561333

AGQK -0.0870047

AGQL -0.207741

AGQM 0.167936

AGQN 0.0220919

AGQP -0.281286

AGQQ 0.245514

AGQR 0.0574629

AGQS -0.12538

AGQT -0.0403879

AGQV -0.154654

AGQW 0.553449

AGQY 0.365545

AGRA -0.431613

AGRC 0.308701

AGRD -0.0420618

AGRE -0.065608

AGRF 0.183567

AGRG -0.621587

AGRH 0.206334

AGRI -0.0813224

AGRK -0.309552

AGRL -0.333107

AGRM 0.255715

AGRN -0.101912

AGRP -0.261751

AGRQ 0.067487

AGRR -0.00396156

AGRS -0.211828

AGRT -0.181568

AGRV -0.231491

AGRW 0.509088

AGRY 0.295019

AGSA -0.568405

AGSC 0.297873

AGSD -0.3349

AGSE -0.378407

AGSF 0.00880194

AGSG -0.443428

AGSH 0.115775

AGSI -0.309891

AGSK -0.322415

AGSL -0.529795

AGSM 0.0426505

AGSN -0.147071

AGSP -0.460053

AGSQ -0.0683968

AGSR -0.169249

AGSS -0.245878

AGST -0.213902

AGSV -0.422478

AGSW 0.368571

AGSY 0.122524

AGTA -0.488742

AGTC 0.390925

AGTD -0.257584

AGTE -0.309284

AGTF 0.127871

AGTG -0.452744

AGTH 0.175134

AGTI -0.177809

AGTK -0.29949

AGTL -0.424956

AGTM 0.186522

AGTN -0.113774

AGTP -0.333986

AGTQ 0.018523

AGTR -0.101709

AGTS -0.242846

AGTT -0.119127

AGTV -0.278785

AGTW 0.475123

AGTY 0.208471

AGVA -0.501351

AGVC 0.53338

AGVD -0.467312

AGVE -0.484973

AGVF 0.150692

AGVG -0.617881

AGVH 0.145318

AGVI -0.105231

AGVK -0.414434

AGVL -0.463507

AGVM 0.383331

AGVN -0.309606

AGVP -0.3912

AGVQ -0.141497

AGVR -0.246749

AGVS -0.417012

AGVT -0.288194

AGVV -0.285481

AGVW 0.604892

AGVY 0.171076

AGWA 0.232079

AGWC 1.09849

AGWD 0.282588

AGWE 0.255026

AGWF 1.01811

AGWG -0.0193598

AGWH 0.700843

AGWI 0.765117

AGWK 0.289988

AGWL 0.600189

AGWM 0.905787

AGWN 0.46035

AGWP 0.305495

AGWQ 0.569921

AGWR 0.563075

AGWS 0.295304

AGWT 0.378079

AGWV 0.638314

AGWW 1.25299

AGWY 1.1097

AGYA -0.116874

AGYC 1.0184

AGYD 0.110473

AGYE -0.0106697

AGYF 0.629941

AGYG -0.2599

AGYH 0.584616

AGYI 0.318914

AGYK 0.0800073

AGYL 0.0433898

AGYM 0.688314

AGYN 0.257147

AGYP 0.142041

AGYQ 0.348626

AGYR 0.290005

AGYS 0.071712

AGYT 0.164853

AGYV 0.153315

AGYW 1.06915

AGYY 0.715082

AHAA -0.568706

AHAC 0.200709

AHAD -0.51612

AHAE -0.485118

AHAF 0.0256705

AHAG -0.963396

AHAH 3.87264

AHAI -0.238235

AHAK -0.414553

AHAL -0.500979

AHAM 0.18204

AHAN -0.238477

AHAP -0.644832

AHAQ -0.0441606

AHAR -0.181031

AHAS -0.495281

AHAT -0.420197

AHAV -0.409219

AHAW 0.358955

AHAY 0.139143

AHCA 0.257069

AHCC 2.29338

AHCD 0.0807436

AHCE 0.0217211

AHCF 0.865639

AHCG -0.0406725

AHCH 0.760344

AHCI 0.592547

AHCK 0.235942

AHCL 0.523227

AHCM 0.74737

AHCN 0.296989

AHCP 0.17474

AHCQ 0.475086

AHCR 0.44571

AHCS 0.303698

AHCT 0.292028

AHCV 0.571611

AHCW 1.134

AHCY 0.897583

AHDA -0.471999

AHDC 0.0961831

AHDD -0.265316

AHDE -0.303236

AHDF 0.0476351

AHDG -0.627575

AHDH 0.233616

AHDI -0.24091

AHDK -0.0720379

AHDL -0.467698

AHDM 0.00498796

AHDN -0.00965142

AHDP -0.383679

AHDQ -0.0464456

AHDR 0.0839243

AHDS -0.199271

AHDT -0.132715

AHDV -0.340418

AHDW 0.382262

AHDY 0.216131

AHEA -0.53934

AHEC 0.0342724

AHED -0.404149

AHEE -0.353255

AHEF -0.0236211

AHEG -0.876576

AHEH 0.16573

AHEI -0.276429

AHEK -0.100741

AHEL -0.493638

AHEM 0.0352023

AHEN -0.176375

AHEP -0.515767

AHEQ -0.0510776

AHER 0.0326414

AHES -0.396515

AHET -0.28805

AHEV -0.405369

AHEW 0.397629

AHEY 0.0968871

AHFA -0.0448465

AHFC 0.882968

AHFD 0.0678072

AHFE -0.0209374

AHFF 0.712754

AHFG -0.349662

AHFH 0.64397

AHFI 0.414712

AHFK 0.0607708

AHFL 0.113901

AHFM 0.834926

AHFN 0.169084

AHFP 0.0724826

AHFQ 0.302326

AHFR 0.236372

AHFS 0.103006

AHFT 0.182011

AHFV 0.242621

AHFW 1.06726

AHFY 0.701469

AHGA -0.95842

AHGC -0.159455

AHGD -0.607582

AHGE -0.739564

AHGF -0.273985

AHGG -1.76753

AHGH -0.262515

AHGI -0.565371

AHGK -0.593681

AHGL -0.76389

AHGM -0.286978

AHGN -0.472192

AHGP -0.829986

AHGQ -0.450905

AHGR -0.432994

AHGS -0.628369

AHGT -0.492244

AHGV -0.649972

AHGW -0.0253866

AHGY -0.145579

AHHA -0.0760467

AHHC 0.684401

AHHD 0.275647

AHHE 0.144301

AHHF 0.632291

AHHG -0.3507

AHHH 0.743849

AHHI 0.280659

AHHK -0.007689

AHHL 0.220517

AHHM 0.559249

AHHN 0.220574

AHHP 0.0295017

AHHQ 0.213089

AHHR 0.294075

AHHS 0.17619

AHHT 0.238264

AHHV 0.217614

AHHW 0.943565

AHHY 0.691754

AHIA -0.254821

AHIC 0.679434

AHID -0.229962

AHIE -0.269701

AHIF 0.421359

AHIG -0.614819

AHIH 0.355339

AHII 0.205524

AHIK -0.175292

AHIL -0.146047

AHIM 0.63276

AHIN -0.0770555

AHIP -0.191953

AHIQ 0.0490904

AHIR -0.0013001

AHIS -0.175097

AHIT -0.0406816

AHIV -0.0099864

AHIW 0.79866

AHIY 0.487823

AHKA -0.516742

AHKC 0.166691

AHKD -0.117124

AHKE -0.123266

AHKF 0.0394566

AHKG -0.781219

AHKH 0.00745583

AHKI -0.207391

AHKK -0.243621

AHKL -0.443921

AHKM 0.0150995

AHKN -0.137879

AHKP -0.439496

AHKQ -0.0358357

AHKR -0.229232

AHKS -0.319722

AHKT -0.222815

AHKV -0.330912

AHKW 0.297288

AHKY 0.175306

AHLA -0.567119

AHLC 0.622612

AHLD -0.490173

AHLE -0.497709

AHLF 0.139137

AHLG -0.830207

AHLH 0.265212

AHLI -0.134723

AHLK -0.432877

AHLL -0.509235

AHLM 0.415626

AHLN -0.324172

AHLP -0.383932

AHLQ -0.0826266

AHLR -0.202191

AHLS -0.447908

AHLT -0.339437

AHLV -0.345077

AHLW 0.676621

AHLY 0.16548

AHMA 0.145905

AHMC 0.823518

AHMD 0.0599439

AHME 0.0819657

AHMF 0.835568

AHMG -0.451493

AHMH 0.473042

AHMI 0.606347

AHMK 0.0974116

AHML 0.412963

AHMM 0.882612

AHMN 0.106858

AHMP -0.0725858

AHMQ 0.265845

AHMR 0.292525

AHMS 0.046706

AHMT 0.218256

AHMV 0.449594

AHMW 0.856435

AHMY 0.783637

AHNA -0.379311

AHNC 0.30011

AHND -0.0440562

AHNE -0.1791

AHNF 0.177702

AHNG -0.510781

AHNH 0.246822

AHNI -0.102666

AHNK -0.0548334

AHNL -0.306706

AHNM 0.12063

AHNN 0.161814

AHNP -0.199188

AHNQ 0.123746

AHNR 0.03842

AHNS -0.0745318

AHNT -0.033004

AHNV -0.208207

AHNW 0.577632

AHNY 0.380934

AHPA -0.361999

AHPC 0.253215

AHPD -0.15182

AHPE -0.132558

AHPF 0.225233

AHPG -0.539562

AHPH 0.244949

AHPI -0.0961359

AHPK -0.202417

AHPL -0.268986

AHPM 0.184302

AHPN -0.0595667

AHPP -0.221528

AHPQ 0.0554259

AHPR 0.0122247

AHPS -0.132136

AHPT -0.0816808

AHPV -0.124481

AHPW 0.638067

AHPY 0.31976

AHQA -0.216576

AHQC 0.457479

AHQD -0.0865424

AHQE -0.0706046

AHQF 0.337567

AHQG -0.477481

AHQH 0.218938

AHQI 0.0240946

AHQK -0.00320959

AHQL -0.112454

AHQM 0.21936

AHQN 0.116324

AHQP -0.218629

AHQQ 0.309318

AHQR 0.140092

AHQS -0.0331786

AHQT 0.0506661

AHQV -0.0725541

AHQW 0.60644

AHQY 0.444759

AHRA -0.340294

AHRC 0.363182

AHRD 0.0442023

AHRE 0.0205402

AHRF 0.278469

AHRG -0.60971

AHRH 0.27287

AHRI -2.599e-05

AHRK -0.231425

AHRL -0.230978

AHRM 0.320623

AHRN -0.0286047

AHRP -0.188024

AHRQ 0.145905

AHRR 0.086266

AHRS -0.114586

AHRT -0.0910296

AHRV -0.134648

AHRW 0.567916

AHRY 0.379505

AHSA -0.512639

AHSC 0.355357

AHSD -0.232392

AHSE -0.268236

AHSF 0.109435

AHSG -0.570181

AHSH 0.296945

AHSI -0.215688

AHSK -0.206773

AHSL -0.419693

AHSM 0.0961983

AHSN -0.0554001

AHSP -0.373781

AHSQ 0.0476549

AHSR -0.0829029

AHSS -0.149658

AHST -0.108324

AHSV -0.336928

AHSW 0.425222

AHSY 0.215232

AHTA -0.411485

AHTC 0.454864

AHTD -0.181829

AHTE -0.207958

AHTF 0.201319

AHTG -0.564899

AHTH 0.319163

AHTI -0.0548518

AHTK -0.198879

AHTL -0.318777

AHTM 0.256171

AHTN -0.0187519

AHTP -0.252279

AHTQ 0.100945

AHTR -0.01371

AHTS -0.137911

AHTT -0.0332484

AHTV -0.17995

AHTW 0.534437

AHTY 0.300957

AHVA -0.423217

AHVC 0.612945

AHVD -0.370741

AHVE -0.389231

AHVF 0.26221

AHVG -0.735959

AHVH 0.333467

AHVI -0.00884104

AHVK -0.310544

AHVL -0.359087

AHVM 0.470596

AHVN -0.212601

AHVP -0.307682

AHVQ -0.0565081

AHVR -0.132055

AHVS -0.332628

AHVT -0.193676

AHVV -0.156204

AHVW 0.683545

AHVY 0.284267

AHWA 0.294444

AHWC 1.13797

AHWD 0.337757

AHWE 0.319413

AHWF 1.08918

AHWG 0.0364888

AHWH 0.753479

AHWI 0.838517

AHWK 0.346709

AHWL 0.686282

AHWM 0.949985

AHWN 0.516534

AHWP 0.354375

AHWQ 0.618172

AHWR 0.623267

AHWS 0.351769

AHWT 0.448794

AHWV 0.716849

AHWW 1.2948

AHWY 1.17819

AHYA -0.0214548

AHYC 1.08555

AHYD 0.19451

AHYE 0.0794821

AHYF 0.72101

AHYG -0.190545

AHYH 0.677019

AHYI 0.409576

AHYK 0.179986

AHYL 0.148524

AHYM 0.758327

AHYN 0.342954

AHYP 0.220283

AHYQ 0.426785

AHYR 0.37298

AHYS 0.154041

AHYT 0.252848

AHYV 0.256053

AHYW 1.13137

AHYY 0.81619

AIAA -1.12372

AIAC -0.236097

AIAD -1.06106

AIAE -1.07063

AIAF -0.284896

AIAG -1.45233

AIAH -0.462505

AIAI 4.54838

AIAK -0.971328

AIAL -0.317495

AIAM 0.142116

AIAN -0.875679

AIAP -1.13795

AIAQ -0.722572

AIAR -0.793607

AIAS -1.01063

AIAT -0.605782

AIAV 0.111738

AIAW -0.124093

AIAY -0.466723

AICA -0.204912

AICC 1.78935

AICD -0.325065

AICE -0.383182

AICF 0.414214

AICG -0.454839

AICH 0.377739

AICI 0.248173

AICK -0.188213

AICL 0.0194523

AICM 0.361729

AICN -0.0990601

AICP -0.213635

AICQ 0.075469

AICR 0.0213053

AICS -0.119464

AICT -0.129189

AICV 0.140271

AICW 0.760181

AICY 0.459022

AIDA -1.02658

AIDC -0.30854

AIDD -0.801629

AIDE -0.849545

AIDF -0.480917

AIDG -1.14316

AIDH -0.248897

AIDI -0.708251

AIDK -0.628421

AIDL -1.00764

AIDM -0.442917

AIDN -0.53946

AIDP -0.86261

AIDQ -0.544595

AIDR -0.459939

AIDS -0.74629

AIDT -0.610135

AIDV -0.907443

AIDW -0.0324638

AIDY -0.307799

AIEA -1.0777

AIEC -0.374956

AIED -0.923146

AIEE -0.90004

AIEF -0.514391

AIEG -1.36999

AIEH -0.337175

AIEI -0.65193

AIEK -0.636136

AIEL -1.01866

AIEM -0.437585

AIEN -0.703853

AIEP -0.994359

AIEQ -0.56265

AIER -0.51533

AIES -0.929251

AIET -0.8815

AIEV -0.925374

AIEW -0.0500972

AIEY -0.429766

AIFA -0.548749

AIFC 0.431903

AIFD -0.452248

AIFE -0.536623

AIFF 0.165361

AIFG -0.85346

AIFH 0.161536

AIFI -0.0146644

AIFK -0.472365

AIFL -0.523248

AIFM 0.332812

AIFN -0.328928

AIFP -0.392107

AIFQ -0.197405

AIFR -0.281476

AIFS -0.392572

AIFT -0.340794

AIFV -0.304688

AIFW 0.58852

AIFY 0.163095

AIGA -1.41205

AIGC -0.556529

AIGD -1.11739

AIGE -1.25344

AIGF -0.746728

AIGG -2.17131

AIGH -0.774925

AIGI -0.603658

AIGK -1.11498

AIGL -1.20045

AIGM -0.680651

AIGN -0.970517

AIGP -1.27301

AIGQ -0.923773

AIGR -0.946117

AIGS -1.11066

AIGT -1.00709

AIGV -1.20925

AIGW -0.443068

AIGY -0.679792

AIHA -0.553297

AIHC 0.309612

AIHD -0.198587

AIHE -0.319448

AIHF 0.163836

AIHG -0.776405

AIHH 0.324697

AIHI -0.124653

AIHK -0.445403

AIHL -0.285238

AIHM 0.171261

AIHN -0.218079

AIHP -0.387936

AIHQ -0.204451

AIHR -0.166057

AIHS -0.291541

AIHT -0.222231

AIHV -0.28402

AIHW 0.543667

AIHY 0.227265

AIIA -0.816545

AIIC 0.211794

AIID -0.757222

AIIE -0.804371

AIIF -0.166007

AIIG -0.745394

AIIH -0.13456

AIII -0.327611

AIIK -0.716217

AIIL -0.769183

AIIM 0.0990973

AIIN -0.598217

AIIP -0.697667

AIIQ -0.456827

AIIR -0.548657

AIIS -0.725291

AIIT -0.579454

AIIV -0.548395

AIIW 0.315216

AIIY -0.0479267

AIKA -1.04602

AIKC -0.247127

AIKD -0.658948

AIKE -0.685857

AIKF -0.472601

AIKG -1.26593

AIKH -0.466302

AIKI -0.616782

AIKK -0.779632

AIKL -1.04619

AIKM -0.42374

AIKN -0.659359

AIKP -0.917205

AIKQ -0.538578

AIKR -0.735571

AIKS -0.839829

AIKT -0.714251

AIKV -0.891073

AIKW -0.128005

AIKY -0.350609

AILA -1.1328

AILC 0.107066

AILD -1.03823

AILE -1.06418

AILF -0.457569

AILG -1.37491

AILH -0.310017

AILI -0.527398

AILK -0.957767

AILL -0.934691

AILM 0.0553105

AILN -0.861311

AILP -0.904549

AILQ -0.641748

AILR -0.778109

AILS -0.983257

AILT -0.921339

AILV -1.00322

AILW 0.156294

AILY -0.399737

AIMA -0.341372

AIMC 0.442191

AIMD -0.377732

AIME -0.379469

AIMF 0.357522

AIMG -0.866208

AIMH 0.0666609

AIMI 0.120559

AIMK -0.346096

AIML -0.129885

AIMM 0.491538

AIMN -0.308949

AIMP -0.474241

AIMQ -0.156356

AIMR -0.153178

AIMS -0.402323

AIMT -0.231029

AIMV -0.0356197

AIMW 0.47491

AIMY 0.308199

AINA -0.908029

AINC -0.0950627

AIND -0.582154

AINE -0.684818

AINF -0.340551

AING -1.00415

AINH -0.2095

AINI -0.490441

AINK -0.596194

AINL -0.890579

AINM -0.302972

AINN -0.348712

AINP -0.66288

AINQ -0.368497

AINR -0.473124

AINS -0.608248

AINT -0.521753

AINV -0.705514

AINW 0.156599

AINY -0.168108

AIPA -0.891045

AIPC -0.147608

AIPD -0.660092

AIPE -0.660552

AIPF -0.293508

AIPG -1.02867

AIPH -0.228455

AIPI -0.390643

AIPK -0.705198

AIPL -0.804465

AIPM -0.249101

AIPN -0.538708

AIPP -0.687667

AIPQ -0.418837

AIPR -0.494568

AIPS -0.642323

AIPT -0.593502

AIPV -0.684465

AIPW 0.19768

AIPY -0.185827

AIQA -0.738135

AIQC 0.0616231

AIQD -0.588916

AIQE -0.578424

AIQF -0.164205

AIQG -0.948308

AIQH -0.212542

AIQI -0.420599

AIQK -0.512555

AIQL -0.62454

AIQM -0.185681

AIQN -0.390992

AIQP -0.670192

AIQQ -0.176004

AIQR -0.363383

AIQS -0.544445

AIQT -0.394082

AIQV -0.580542

AIQW 0.201702

AIQY -0.0576446

AIRA -0.886612

AIRC -0.0545847

AIRD -0.478104

AIRE -0.527113

AIRF -0.257538

AIRG -1.1061

AIRH -0.179485

AIRI -0.411484

AIRK -0.732157

AIRL -0.78137

AIRM -0.122798

AIRN -0.528351

AIRP -0.665735

AIRQ -0.354104

AIRR -0.452759

AIRS -0.64362

AIRT -0.630969

AIRV -0.633587

AIRW 0.132127

AIRY -0.098743

AISA -1.02958

AISC -0.0807488

AISD -0.765361

AISE -0.831179

AISF -0.443419

AISG -1.10703

AISH -0.281626

AISI -0.31155

AISK -0.79183

AISL -0.796078

AISM -0.362148

AISN -0.593622

AISP -0.875496

AISQ -0.470006

AISR -0.618052

AISS -0.637886

AIST -0.678669

AISV -0.589806

AISW -0.0204003

AISY -0.317742

AITA -0.978384

AITC 0.0178587

AITD -0.718031

AITE -0.749472

AITF -0.329102

AITG -1.10173

AITH -0.22656

AITI -0.297873

AITK -0.734419

AITL -0.98119

AITM -0.220437

AITN -0.548556

AITP -0.74354

AITQ -0.416505

AITR -0.55333

AITS -0.695492

AITT -0.59622

AITV -0.6146

AITW 0.0890481

AITY -0.244435

AIVA -1.00473

AIVC 0.127411

AIVD -0.912639

AIVE -0.938606

AIVF -0.282199

AIVG -1.29679

AIVH -0.268624

AIVI 0.449158

AIVK -0.833929

AIVL -0.711612

AIVM 0.0283046

AIVN -0.738499

AIVP -0.825885

AIVQ -0.59555

AIVR -0.673025

AIVS -0.882592

AIVT -0.715935

AIVV -0.647673

AIVW 0.209956

AIVY -0.303873

AIWA -0.167727

AIWC 0.765109

AIWD -0.0929983

AIWE -0.11213

AIWF 0.621557

AIWG -0.386534

AIWH 0.373589

AIWI 0.372273

AIWK -0.079536

AIWL 0.160299

AIWM 0.56809

AIWN 0.0960021

AIWP -0.0462658

AIWQ 0.209077

AIWR 0.18288

AIWS -0.0828035

AIWT 0.00362372

AIWV 0.21415

AIWW 0.909612

AIWY 0.709591

AIYA -0.565756

AIYC 0.623705

AIYD -0.332806

AIYE -0.458244

AIYF 0.182851

AIYG -0.696033

AIYH 0.202082

AIYI -0.0274732

AIYK -0.35434

AIYL -0.445811

AIYM 0.289283

AIYN -0.16206

AIYP -0.278987

AIYQ -0.0302737

AIYR -0.14316

AIYS -0.380781

AIYT -0.279445

AIYV -0.277136

AIYW 0.667777

AIYY 0.267687

AKAA -0.776295

AKAC -0.0432408

AKAD -0.764649

AKAE -0.592281

AKAF -0.344039

AKAG -1.23746

AKAH -0.220273

AKAI -0.552778

AKAK 4.16084

AKAL -0.803849

AKAM -0.0899742

AKAN -0.450015

AKAP -0.904127

AKAQ -0.239775

AKAR -0.1106

AKAS -0.75528

AKAT -0.561238

AKAV -0.703692

AKAW 0.0918214

AKAY -0.254255

AKCA -0.0066483

AKCC 2.0041

AKCD -0.131871

AKCE -0.193109

AKCF 0.618297

AKCG -0.267109

AKCH 0.562775

AKCI 0.339957

AKCK 0.0454206

AKCL 0.228611

AKCM 0.553501

AKCN 0.0904956

AKCP -0.0225356

AKCQ 0.262217

AKCR 0.221935

AKCS 0.0795314

AKCT 0.0657151

AKCV 0.314015

AKCW 0.942637

AKCY 0.655517

AKDA -0.785373

AKDC -0.119231

AKDD -0.570693

AKDE -0.613137

AKDF -0.234023

AKDG -0.924078

AKDH -0.038913

AKDI -0.537864

AKDK -0.159744

AKDL -0.769201

AKDM -0.228474

AKDN -0.311962

AKDP -0.654238

AKDQ -0.328088

AKDR -0.22691

AKDS -0.515502

AKDT -0.471698

AKDV -0.665967

AKDW 0.143854

AKDY -0.0838671

AKEA -0.780546

AKEC -0.18355

AKED -0.712556

AKEE -0.621614

AKEF -0.33566

AKEG -1.14956

AKEH -0.126883

AKEI -0.58754

AKEK -0.187528

AKEL -0.815629

AKEM -0.208805

AKEN -0.455882

AKEP -0.791872

AKEQ -0.335483

AKER -0.240755

AKES -0.739448

AKET -0.591794

AKEV -0.718221

AKEW 0.151489

AKEY -0.190917

AKFA -0.363856

AKFC 0.634228

AKFD -0.236762

AKFE -0.307097

AKFF 0.373975

AKFG -0.609738

AKFH 0.371651

AKFI 0.0732629

AKFK -0.242682

AKFL -0.227817

AKFM 0.56847

AKFN -0.122885

AKFP -0.206806

AKFQ 0.0207427

AKFR -0.0661783

AKFS -0.190142

AKFT -0.114724

AKFV -0.101267

AKFW 0.775279

AKFY 0.384364

AKGA -1.25155

AKGC -0.373374

AKGD -0.900469

AKGE -1.02713

AKGF -0.57139

AKGG -1.97686

AKGH -0.576143

AKGI -0.869685

AKGK -0.678562

AKGL -1.05071

AKGM -0.511691

AKGN -0.746339

AKGP -1.08321

AKGQ -0.723315

AKGR -0.709382

AKGS -0.909211

AKGT -0.786606

AKGV -0.960022

AKGW -0.250595

AKGY -0.455156

AKHA -0.350329

AKHC 0.49286

AKHD 0.0109646

AKHE -0.124147

AKHF 0.381682

AKHG -0.586383

AKHH 0.520579

AKHI 0.028728

AKHK -0.215155

AKHL -0.102185

AKHM 0.357044

AKHN -0.0230923

AKHP -0.194141

AKHQ -0.0141616

AKHR 0.0537012

AKHS -0.0976059

AKHT -0.0209835

AKHV -0.0473094

AKHW 0.725594

AKHY 0.446302

AKIA -0.597031

AKIC 0.41762

AKID -0.554317

AKIE -0.579001

AKIF 0.0906999

AKIG -0.911686

AKIH 0.0642877

AKII -0.118255

AKIK -0.439679

AKIL -0.509373

AKIM 0.338331

AKIN -0.363229

AKIP -0.478094

AKIQ -0.243081

AKIR -0.293287

AKIS -0.493637

AKIT -0.360874

AKIV -0.350605

AKIW 0.524722

AKIY 0.135352

AKKA -0.81292

AKKC -0.0550094

AKKD -0.420677

AKKE -0.425584

AKKF -0.261665

AKKG -1.06445

AKKH -0.264842

AKKI -0.509436

AKKK -0.312243

AKKL -0.757291

AKKM -0.221914

AKKN -0.414288

AKKP -0.707811

AKKQ -0.193428

AKKR -0.531931

AKKS -0.585003

AKKT -0.527593

AKKV -0.645377

AKKW 0.0728655

AKKY -0.132193

AKLA -0.901948

AKLC 0.319986

AKLD -0.820609

AKLE -0.862981

AKLF -0.211762

AKLG -1.1626

AKLH -0.100953

AKLI -0.512518

AKLK -0.601792

AKLL -0.913137

AKLM 0.111755

AKLN -0.650021

AKLP -0.693238

AKLQ -0.429757

AKLR -0.53081

AKLS -0.729838

AKLT -0.675901

AKLV -0.693523

AKLW 0.383415

AKLY -0.169968

AKMA -0.146126

AKMC 0.62753

AKMD -0.175399

AKME -0.179386

AKMF 0.555811

AKMG -0.693356

AKMH 0.254769

AKMI 0.308251

AKMK -0.127191

AKML 0.0875871

AKMM 0.651462

AKMN -0.114516

AKMP -0.280371

AKMQ 0.0463362

AKMR 0.0493505

AKMS -0.195251

AKMT -0.0386951

AKMV 0.152012

AKMW 0.659993

AKMY 0.516584

AKNA -0.677334

AKNC 0.0899119

AKND -0.367529

AKNE -0.472425

AKNF -0.108005

AKNG -0.789014

AKNH -0.00222397

AKNI -0.399143

AKNK -0.272879

AKNL -0.61992

AKNM -0.0924201

AKNN -0.107315

AKNP -0.466165

AKNQ -0.149782

AKNR -0.253425

AKNS -0.400869

AKNT -0.32968

AKNV -0.506619

AKNW 0.359542

AKNY 0.0595691

AKPA -0.661448

AKPC 0.0432079

AKPD -0.450451

AKPE -0.412952

AKPF -0.0703559

AKPG -0.819654

AKPH -0.0414264

AKPI -0.39269

AKPK -0.198067

AKPL -0.57891

AKPM -0.0448663

AKPN -0.339824

AKPP -0.485176

AKPQ -0.216455

AKPR -0.231992

AKPS -0.443177

AKPT -0.365546

AKPV -0.445008

AKPW 0.395503

AKPY 0.0377741

AKQA -0.511023

AKQC 0.249531

AKQD -0.359928

AKQE -0.369843

AKQF 0.0510757

AKQG -0.757269

AKQH -0.023767

AKQI -0.26884

AKQK -0.247813

AKQL -0.42782

AKQM 0.00140429

AKQN -0.157917

AKQP -0.464978

AKQQ 0.0367293

AKQR -0.139404

AKQS -0.331342

AKQT -0.252676

AKQV -0.380733

AKQW 0.383617

AKQY 0.155859

AKRA -0.675738

AKRC 0.136462

AKRD -0.275769

AKRE -0.261682

AKRF -0.033679

AKRG -0.889334

AKRH 0.0667522

AKRI -0.31549

AKRK -0.471157

AKRL -0.56696

AKRM 0.0683711

AKRN -0.283242

AKRP -0.466374

AKRQ -0.061043

AKRR -0.21032

AKRS -0.435125

AKRT -0.410885

AKRV -0.457701

AKRW 0.331975

AKRY 0.067944

AKSA -0.830753

AKSC 0.120685

AKSD -0.542068

AKSE -0.582452

AKSF -0.202669

AKSG -0.875484

AKSH -0.0320995

AKSI -0.514904

AKSK 0.0121911

AKSL -0.748478

AKSM -0.146428

AKSN -0.401164

AKSP -0.650227

AKSQ -0.266493

AKSR -0.427209

AKSS -0.418486

AKST -0.47219

AKSV -0.653115

AKSW 0.176211

AKSY -0.0998034

AKTA -0.717371

AKTC 0.211257

AKTD -0.503682

AKTE -0.540604

AKTF -0.137407

AKTG -0.859341

AKTH -0.0325243

AKTI -0.372733

AKTK -0.307368

AKTL -0.66586

AKTM -0.00838542

AKTN -0.312032

AKTP -0.531433

AKTQ -0.200741

AKTR -0.357983

AKTS -0.477591

AKTT -0.435345

AKTV -0.511291

AKTW 0.290211

AKTY 0.00791788

AKVA -0.763439

AKVC 0.33778

AKVD -0.673274

AKVE -0.716624

AKVF -0.0970852

AKVG -1.03037

AKVH -0.0527897

AKVI -0.351204

AKVK -0.0779471

AKVL -0.693845

AKVM 0.164595

AKVN -0.51823

AKVP -0.604157

AKVQ -0.387696

AKVR -0.444278

AKVS -0.652381

AKVT -0.549671

AKVV -0.537281

AKVW 0.398155

AKVY -0.051029

AKWA 0.0416563

AKWC 0.946587

AKWD 0.107348

AKWE 0.0781863

AKWF 0.823398

AKWG -0.192364

AKWH 0.546443

AKWI 0.563642

AKWK 0.12009

AKWL 0.382911

AKWM 0.746959

AKWN 0.28988

AKWP 0.140573

AKWQ 0.398581

AKWR 0.388843

AKWS 0.114418

AKWT 0.197639

AKWV 0.434723

AKWW 1.09451

AKWY 0.914073

AKYA -0.344685

AKYC 0.827861

AKYD -0.104533

AKYE -0.172906

AKYF 0.386793

AKYG -0.488323

AKYH 0.383504

AKYI 0.0896914

AKYK -0.102039

AKYL -0.194897

AKYM 0.498821

AKYN 0.047581

AKYP -0.0614257

AKYQ 0.133615

AKYR 0.0599432

AKYS -0.16936

AKYT -0.0526605

AKYV -0.0794647

AKYW 0.988695

AKYY 0.489869

ALAA -1.27395

ALAC -0.522336

ALAD -1.39134

ALAE -1.30549

ALAF -0.37277

ALAG -1.76693

ALAH -0.736629

ALAI -0.328875

ALAK -1.23378

ALAL 4.39181

ALAM 0.0842021

ALAN -1.13303

ALAP -1.43457

ALAQ -0.9611

ALAR -1.05732

ALAS -1.31676

ALAT -1.1949

ALAV -0.0137918

ALAW -0.402848

ALAY -0.741363

ALCA -0.512826

ALCC 1.47137

ALCD -0.619136

ALCE -0.683274

ALCF 0.116789

ALCG -0.752139

ALCH 0.0851326

ALCI -0.118006

ALCK -0.495003

ALCL -0.205176

ALCM 0.0794001

ALCN -0.394779

ALCP -0.495379

ALCQ -0.218446

ALCR -0.274255

ALCS -0.431005

ALCT -0.426295

ALCV -0.253004

ALCW 0.47124

ALCY 0.157538

ALDA -1.35136

ALDC -0.606688

ALDD -1.12575

ALDE -1.17251

ALDF -0.775205

ALDG -1.46163

ALDH -0.557521

ALDI -1.08445

ALDK -0.962346

ALDL -1.15213

ALDM -0.73222

ALDN -0.855343

ALDP -1.1666

ALDQ -0.86334

ALDR -0.774606

ALDS -1.06078

ALDT -1.03494

ALDV -1.18793

ALDW -0.357022

ALDY -0.636205

ALEA -1.40528

ALEC -0.671908

ALED -1.24328

ALEE -1.22866

ALEF -0.854574

ALEG -1.6842

ALEH -0.653068

ALEI -1.14868

ALEK -1.00235

ALEL -1.15481

ALEM -0.671097

ALEN -1.02035

ALEP -1.29936

ALEQ -0.896122

ALER -0.836683

ALES -1.22162

ALET -1.1452

ALEV -1.25967

ALEW -0.303866

ALEY -0.724763

ALFA -0.898872

ALFC 0.122221

ALFD -0.772436

ALFE -0.872027

ALFF -0.138497

ALFG -1.15996

ALFH -0.140792

ALFI 0.132966

ALFK -0.774197

ALFL -0.624556

ALFM 0.224259

ALFN -0.648127

ALFP -0.733315

ALFQ -0.486071

ALFR -0.600849

ALFS -0.759958

ALFT -0.697636

ALFV -0.67232

ALFW 0.281881

ALFY -0.165465

ALGA -1.79623

ALGC -0.85111

ALGD -1.43297

ALGE -1.57051

ALGF -1.08884

ALGG -2.46748

ALGH -1.05948

ALGI -1.34791

ALGK -1.43313

ALGL -0.944076

ALGM -1.0306

ALGN -1.28966

ALGP -1.59109

ALGQ -1.23573

ALGR -1.27341

ALGS -1.46723

ALGT -1.34577

ALGV -1.38005

ALGW -0.750304

ALGY -0.996771

ALHA -0.853506

ALHC 0.0195351

ALHD -0.515559

ALHE -0.633923

ALHF -0.157031

ALHG -1.08394

ALHH 0.0248981

ALHI -0.502989

ALHK -0.746926

ALHL -0.526801

ALHM -0.123877

ALHN -0.520239

ALHP -0.681592

ALHQ -0.50533

ALHR -0.472738

ALHS -0.607753

ALHT -0.522834

ALHV -0.594633

ALHW 0.289434

ALHY -0.0864446

ALIA -1.11864

ALIC -0.0890226

ALID -1.08054

ALIE -1.11563

ALIF -0.409523

ALIG -1.45363

ALIH -0.446528

ALII -0.703794

ALIK -1.06198

ALIL -0.604062

ALIM -0.213385

ALIN -0.904926

ALIP -0.981979

ALIQ -0.738352

ALIR -0.849023

ALIS -1.02263

ALIT -0.913476

ALIV -0.940182

ALIW -0.00043821

ALIY -0.415515

ALKA -1.36119

ALKC -0.547774

ALKD -0.994214

ALKE -0.985683

ALKF -0.765894

ALKG -1.57213

ALKH -0.7729

ALKI -1.07678

ALKK -1.09593

ALKL -1.15146

ALKM -0.732451

ALKN -0.974813

ALKP -1.23705

ALKQ -0.834876

ALKR -1.06752

ALKS -1.15811

ALKT -1.08318

ALKV -1.21598

ALKW -0.422931

ALKY -0.68731

ALLA -1.48172

ALLC -0.217022

ALLD -1.36409

ALLE -1.3887

ALLF -0.790195

ALLG -1.68833

ALLH -0.631941

ALLI -1.09095

ALLK -1.29478

ALLL -1.20217

ALLM -0.322444

ALLN -1.18789

ALLP -1.24821

ALLQ -0.942251

ALLR -1.10874

ALLS -1.31637

ALLT -1.24462

ALLV -1.16899

ALLW -0.171958

ALLY -0.727623

ALMA -0.673834

ALMC 0.152174

ALMD -0.685228

ALME -0.689792

ALMF 0.0526879

ALMG -1.15723

ALMH -0.219765

ALMI -0.224679

ALMK -0.646656

ALML -0.329053

ALMM 0.156137

ALMN -0.612159

ALMP -0.760418

ALMQ -0.455594

ALMR -0.453531

ALMS -0.724053

ALMT -0.556635

ALMV -0.392081

ALMW 0.187627

ALMY -0.00412154

ALNA -1.19065

ALNC -0.392841

ALND -0.910258

ALNE -1.01478

ALNF -0.654963

ALNG -1.3128

ALNH -0.466323

ALNI -0.940949

ALNK -0.918653

ALNL -1.01695

ALNM -0.596795

ALNN -0.673532

ALNP -0.981322

ALNQ -0.676266

ALNR -0.783934

ALNS -0.933988

ALNT -0.841614

ALNV -1.00019

ALNW -0.122499

ALNY -0.455353

ALPA -1.22631

ALPC -0.436604

ALPD -0.981335

ALPE -0.984014

ALPF -0.553113

ALPG -1.3439

ALPH -0.536642

ALPI -0.84337

ALPK -1.03403

ALPL -0.837898

ALPM -0.522044

ALPN -0.857758

ALPP -0.997803

ALPQ -0.743883

ALPR -0.817074

ALPS -0.962718

ALPT -0.882351

ALPV -1.01693

ALPW -0.106734

ALPY -0.509661

ALQA -1.05482

ALQC -0.23331

ALQD -0.888825

ALQE -0.902171

ALQF -0.491189

ALQG -1.25988

ALQH -0.502937

ALQI -0.783885

ALQK -0.83188

ALQL -0.84041

ALQM -0.487412

ALQN -0.69409

ALQP -0.98669

ALQQ -0.476368

ALQR -0.664517

ALQS -0.871791

ALQT -0.786631

ALQV -0.82179

ALQW -0.095037

ALQY -0.368858

ALRA -1.20729

ALRC -0.341536

ALRD -0.831621

ALRE -0.85774

ALRF -0.577374

ALRG -1.41716

ALRH -0.482569

ALRI -0.842035

ALRK -1.06249

ALRL -1.02158

ALRM -0.438854

ALRN -0.831733

ALRP -0.981296

ALRQ -0.668998

ALRR -0.744429

ALRS -0.981459

ALRT -0.919298

ALRV -1.05306

ALRW -0.171789

ALRY -0.482966

ALSA -1.41353

ALSC -0.382011

ALSD -1.08468

ALSE -1.13581

ALSF -0.593903

ALSG -1.41636

ALSH -0.579509

ALSI -1.06921

ALSK -1.04403

ALSL -0.644637

ALSM -0.657171

ALSN -0.926243

ALSP -1.19377

ALSQ -0.803673

ALSR -0.944583

ALSS -0.965986

ALST -0.946217

ALSV -1.117

ALSW -0.308154

ALSY -0.652364

ALTA -1.29819

ALTC -0.294654

ALTD -1.02863

ALTE -1.05784

ALTF -0.657785

ALTG -1.38632

ALTH -0.522966

ALTI -1.0036

ALTK -1.08046

ALTL -0.666308

ALTM -0.51627

ALTN -0.86386

ALTP -1.05749

ALTQ -0.748837

ALTR -0.880844

ALTS -1.03801

ALTT -0.867864

ALTV -0.958613

ALTW -0.215584

ALTY -0.578487

ALVA -1.34616

ALVC -0.187608

ALVD -1.22055

ALVE -1.27409

ALVF -0.621021

ALVG -1.57959

ALVH -0.583454

ALVI -0.645744

ALVK -1.20362

ALVL -0.159478

ALVM -0.370738

ALVN -1.03723

ALVP -1.14922

ALVQ -0.885227

ALVR -1.00094

ALVS -1.16248

ALVT -1.10922

ALVV -0.588884

ALVW -0.0976725

ALVY -0.607929

ALWA -0.475853

ALWC 0.475596

ALWD -0.389456

ALWE -0.423554

ALWF 0.309963

ALWG -0.684235

ALWH 0.0748682

ALWI 0.0375504

ALWK -0.387228

ALWL -0.15424

ALWM 0.262786

ALWN -0.200556

ALWP -0.344405

ALWQ -0.0845647

ALWR -0.112024

ALWS -0.375124

ALWT -0.297996

ALWV -0.0901008

ALWW 0.61632

ALWY 0.397863

ALYA -0.884525

ALYC 0.317974

ALYD -0.654022

ALYE -0.758028

ALYF -0.14669

ALYG -1.0139

ALYH -0.116909

ALYI -0.484591

ALYK -0.65999

ALYL -0.711842

ALYM -0.0398653

ALYN -0.492664

ALYP -0.627118

ALYQ -0.385258

ALYR -0.476336

ALYS -0.692726

ALYT -0.601992

ALYV -0.635861

ALYW 0.364218

ALYY -0.0605693

AMAA -0.557913

AMAC 0.159259

AMAD -0.572526

AMAE -0.594619

AMAF 0.118964

AMAG -0.948034

AMAH -0.0363796

AMAI 0.147967

AMAK -0.502674

AMAL 0.101432

AMAM 4.25506

AMAN -0.414352

AMAP -0.684895

AMAQ -0.254681

AMAR -0.322889

AMAS -0.562346

AMAT -0.294278

AMAV -0.240164

AMAW 0.319572

AMAY -0.0289307

AMCA 0.213619

AMCC 2.24411

AMCD 0.0485303

AMCE -0.0104845

AMCF 0.827712

AMCG -0.072583

AMCH 0.729447

AMCI 0.546258

AMCK 0.200314

AMCL 0.470042

AMCM 0.730318

AMCN 0.265569

AMCP 0.14654

AMCQ 0.445857

AMCR 0.416909

AMCS 0.269094

AMCT 0.258823

AMCV 0.533384

AMCW 1.10868

AMCY 0.861116

AMDA -0.533402

AMDC 0.0656331

AMDD -0.328688

AMDE -0.368943

AMDF -0.0108347

AMDG -0.679269

AMDH 0.182438

AMDI -0.296035

AMDK -0.126308

AMDL -0.517439

AMDM -0.0100663

AMDN -0.0477788

AMDP -0.427099

AMDQ -0.0851238

AMDR 0.033124

AMDS -0.256251

AMDT -0.202409

AMDV -0.402216

AMDW 0.346412

AMDY 0.170732

AMEA -0.604748

AMEC 0.00323844

AMED -0.45542

AMEE -0.401114

AMEF -0.0841832

AMEG -0.926136

AMEH 0.0896204

AMEI -0.326231

AMEK -0.17353

AMEL -0.538653

AMEM -0.00100684

AMEN -0.229542

AMEP -0.558754

AMEQ -0.112483

AMER -0.0338125

AMES -0.454359

AMET -0.34506

AMEV -0.470184

AMEW 0.359673

AMEY 0.0399752

AMFA -0.103939

AMFC 0.84436

AMFD 0.0153422

AMFE -0.0761886

AMFF 0.680989

AMFG -0.400763

AMFH 0.59607

AMFI 0.335093

AMFK 0.0147841

AMFL 0.605912

AMFM 0.788457

AMFN 0.118404

AMFP 0.028152

AMFQ 0.252107

AMFR 0.17963

AMFS 0.0487146

AMFT 0.116804

AMFV 0.201318

AMFW 1.01131

AMFY 0.650788

AMGA -1.00234

AMGC -0.189871

AMGD -0.661574

AMGE -0.792369

AMGF -0.285599

AMGG -1.79775

AMGH -0.380192

AMGI -0.590233

AMGK -0.639923

AMGL -0.83833

AMGM -0.233073

AMGN -0.510754

AMGP -0.869911

AMGQ -0.495317

AMGR -0.482221

AMGS -0.665289

AMGT -0.549595

AMGV -0.678959

AMGW -0.0618136

AMGY -0.214829

AMHA -0.120579

AMHC 0.661261

AMHD 0.230011

AMHE 0.102284

AMHF 0.588513

AMHG -0.387751

AMHH 0.710584

AMHI 0.236695

AMHK -0.0436072

AMHL 0.153238

AMHM 0.534201

AMHN 0.182556

AMHP -0.0026691

AMHQ 0.189586

AMHR 0.250665

AMHS 0.12658

AMHT 0.193825

AMHV 0.166934

AMHW 0.909982

AMHY 0.650645

AMIA -0.334415

AMIC 0.637457

AMID -0.295634

AMIE -0.31859

AMIF 0.348974

AMIG -0.680435

AMIH 0.286129

AMII 0.137467

AMIK -0.234977

AMIL -0.196658

AMIM 0.595786

AMIN -0.113705

AMIP -0.243831

AMIQ 0.00364161

AMIR -0.0602076

AMIS -0.236112

AMIT -0.0944245

AMIV 0.0167265

AMIW 0.743286

AMIY 0.399886

AMKA -0.571942

AMKC 0.134923

AMKD -0.17165

AMKE -0.177428

AMKF -0.0168417

AMKG -0.828167

AMKH -0.0600562

AMKI -0.253512

AMKK -0.295082

AMKL -0.507503

AMKM -0.0100884

AMKN -0.195488

AMKP -0.484429

AMKQ -0.0808387

AMKR -0.28352

AMKS -0.368792

AMKT -0.265253

AMKV -0.39059

AMKW 0.264122

AMKY 0.130759

AMLA -0.638266

AMLC 0.573693

AMLD -0.547146

AMLE -0.551239

AMLF 0.0652752

AMLG -0.884641

AMLH 0.153781

AMLI -0.212994

AMLK -0.486658

AMLL -0.56503

AMLM 0.369216

AMLN -0.361643

AMLP -0.436042

AMLQ -0.143391

AMLR -0.28118

AMLS -0.513693

AMLT -0.398469

AMLV -0.393052

AMLW 0.624091

AMLY 0.114686

AMMA 0.092582

AMMC 0.797351

AMMD 0.0296838

AMME 0.0323489

AMMF 0.788169

AMMG -0.481954

AMMH 0.42502

AMMI 0.54886

AMMK 0.0622358

AMML 0.369945

AMMM 0.862555

AMMN 0.0751772

AMMP -0.102345

AMMQ 0.231824

AMMR 0.255543

AMMS 0.0077827

AMMT 0.179644

AMMV 0.403218

AMMW 0.827963

AMMY 0.740828

AMNA -0.421866

AMNC 0.270724

AMND -0.109352

AMNE -0.224438

AMNF 0.132818

AMNG -0.561253

AMNH 0.19804

AMNI -0.162693

AMNK -0.122765

AMNL -0.375401

AMNM 0.10367

AMNN 0.108488

AMNP -0.236303

AMNQ 0.0897999

AMNR -0.0114081

AMNS -0.133324

AMNT -0.0810282

AMNV -0.254169

AMNW 0.544028

AMNY 0.305711

AMPA -0.421536

AMPC 0.221701

AMPD -0.199264

AMPE -0.18291

AMPF 0.174103

AMPG -0.585928

AMPH 0.164001

AMPI -0.146947

AMPK -0.253994

AMPL -0.31614

AMPM 0.180686

AMPN -0.0959928

AMPP -0.263266

AMPQ 0.022361

AMPR -0.0362515

AMPS -0.18401

AMPT -0.131302

AMPV -0.187419

AMPW 0.596936

AMPY 0.270555

AMQA -0.266719

AMQC 0.428668

AMQD -0.133048

AMQE -0.117955

AMQF 0.295353

AMQG -0.518113

AMQH 0.168703

AMQI -0.0263796

AMQK -0.0511227

AMQL -0.184222

AMQM 0.191268

AMQN 0.0478382

AMQP -0.256229

AMQQ 0.269431

AMQR 0.089987

AMQS -0.0825326

AMQT -0.0052197

AMQV -0.12541

AMQW 0.571121

AMQY 0.395695

AMRA -0.401566

AMRC 0.328511

AMRD -0.00400448

AMRE -0.0268331

AMRF 0.221713

AMRG -0.657986

AMRH 0.229893

AMRI -0.0595615

AMRK -0.283046

AMRL -0.291054

AMRM 0.288686

AMRN -0.076102

AMRP -0.231585

AMRQ 0.104123

AMRR 0.0315628

AMRS -0.165789

AMRT -0.145497

AMRV -0.183373

AMRW 0.530081

AMRY 0.323947

AMSA -0.577243

AMSC 0.319542

AMSD -0.283769

AMSE -0.336174

AMSF 0.0706968

AMSG -0.627135

AMSH 0.13734

AMSI -0.261075

AMSK -0.303174

AMSL -0.336127

AMSM 0.163874

AMSN -0.110718

AMSP -0.427396

AMSQ -0.0132568

AMSR -0.133193

AMSS -0.201901

AMST -0.180647

AMSV -0.378797

AMSW 0.389791

AMSY 0.153625

AMTA -0.470295

AMTC 0.416764

AMTD -0.231709

AMTE -0.255771

AMTF 0.175191

AMTG -0.613468

AMTH 0.197693

AMTI -0.132609

AMTK -0.256206

AMTL -0.453976

AMTM 0.463076

AMTN -0.0726659

AMTP -0.298147

AMTQ 0.0587308

AMTR -0.0788231

AMTS -0.200283

AMTT -0.0989189

AMTV -0.247117

AMTW 0.498382

AMTY 0.246423

AMVA -0.488188

AMVC 0.561399

AMVD -0.421732

AMVE -0.448815

AMVF 0.200406

AMVG -0.785415

AMVH 0.175514

AMVI -0.158623

AMVK -0.374554

AMVL -0.363141

AMVM 0.571803

AMVN -0.27321

AMVP -0.349609

AMVQ -0.114548

AMVR -0.208978

AMVS -0.387874

AMVT -0.238328

AMVV -0.0153275

AMVW 0.693294

AMVY 0.215355

AMWA 0.252475

AMWC 1.11273

AMWD 0.305631

AMWE 0.278047

AMWF 1.04715

AMWG 0.00404096

AMWH 0.714871

AMWI 0.797087

AMWK 0.311704

AMWL 0.624908

AMWM 0.935482

AMWN 0.480765

AMWP 0.324028

AMWQ 0.586399

AMWR 0.58599

AMWS 0.321362

AMWT 0.399955

AMWV 0.668334

AMWW 1.2719

AMWY 1.13606

AMYA -0.0876408

AMYC 1.04473

AMYD 0.155842

AMYE 0.0164771

AMYF 0.760983

AMYG -0.239992

AMYH 0.6075

AMYI 0.361348

AMYK 0.113397

AMYL 0.112805

AMYM 0.751565

AMYN 0.285295

AMYP 0.16865

AMYQ 0.378241

AMYR 0.320998

AMYS 0.0965981

AMYT 0.194932

AMYV 0.175717

AMYW 1.08547

AMYY 0.73007

ANAA -0.718478

ANAC 0.104101

ANAD -0.526875

ANAE -0.585155

ANAF -0.130011

ANAG -1.0059

ANAH 0.0293419

ANAI -0.383589

ANAK -0.376476

ANAL -0.629565

ANAM 0.0718873

ANAN 3.96672

ANAP -0.769543

ANAQ -0.285516

ANAR -0.280923

ANAS -0.343377

ANAT -0.345415

ANAV -0.569992

ANAW 0.254111

ANAY -0.0617847

ANCA 0.152697

ANCC 2.16868

ANCD 0.00398707

ANCE -0.0618575

ANCF 0.7662

ANCG -0.124669

ANCH 0.690966

ANCI 0.494841

ANCK 0.146183

ANCL 0.403114

ANCM 0.678708

ANCN 0.243107

ANCP 0.101228

ANCQ 0.394298

ANCR 0.358406

ANCS 0.212082

ANCT 0.204331

ANCV 0.457788

ANCW 1.06754

ANCY 0.809772

ANDA -0.595622

ANDC 0.0199368

ANDD -0.404292

ANDE -0.450361

ANDF -0.0897455

ANDG -0.732074

ANDH 0.116146

ANDI -0.375174

ANDK -0.228003

ANDL -0.606412

ANDM -0.0945375

ANDN -0.0746367

ANDP -0.494009

ANDQ -0.162297

ANDR -0.0514522

ANDS -0.342517

ANDT -0.287698

ANDV -0.484489

ANDW 0.290299

ANDY 0.0868855

ANEA -0.660172

ANEC -0.0474446

ANED -0.54125

ANEE -0.460718

ANEF -0.16319

ANEG -0.997146

ANEH 0.0360515

ANEI -0.404488

ANEK -0.26025

ANEL -0.650649

ANEM -0.0696795

ANEN -0.235656

ANEP -0.629377

ANEQ -0.198678

ANER -0.117664

ANES -0.53281

ANET -0.435216

ANEV -0.517034

ANEW 0.301133

ANEY -0.0423675

ANFA -0.195276

ANFC 0.782668

ANFD -0.0562611

ANFE -0.165555

ANFF 0.567782

ANFG -0.468918

ANFH 0.530503

ANFI 0.26165

ANFK -0.079623

ANFL -0.0410857

ANFM 0.715797

ANFN 0.0685031

ANFP 0.0400844

ANFQ 0.195095

ANFR 0.115304

ANFS -0.0324759

ANFT 0.0407219

ANFV 0.0871627

ANFW 0.949762

ANFY 0.561091

ANGA -1.08302

ANGC -0.234656

ANGD -0.706382

ANGE -0.869726

ANGF -0.396177

ANGG -1.84668

ANGH -0.431177

ANGI -0.690983

ANGK -0.721894

ANGL -0.911428

ANGM -0.380809

ANGN -0.398551

ANGP -0.93328

ANGQ -0.562924

ANGR -0.567786

ANGS -0.73456

ANGT -0.608635

ANGV -0.794005

ANGW -0.114204

ANGY -0.299986

ANHA -0.180546

ANHC 0.617459

ANHD 0.164634

ANHE 0.0327837

ANHF 0.528742

ANHG -0.442311

ANHH 0.65612

ANHI 0.171229

ANHK -0.104678

ANHL 0.0782239

ANHM 0.485367

ANHN 0.143419

ANHP -0.0551927

ANHQ 0.136815

ANHR 0.196482

ANHS 0.0623963

ANHT 0.127155

ANHV 0.0959582

ANHW 0.865036

ANHY 0.584939

ANIA -0.389112

ANIC 0.572833

ANID -0.375721

ANIE -0.372174

ANIF 0.294935

ANIG -0.747542

ANIH 0.2346

ANII 0.0518174

ANIK -0.332625

ANIL -0.330736

ANIM 0.49922

ANIN -0.18335

ANIP -0.312827

ANIQ -0.0547862

ANIR -0.139031

ANIS -0.334147

ANIT -0.188005

ANIV -0.125268

ANIW 0.683311

ANIY 0.312472

ANKA -0.672348

ANKC 0.0826387

ANKD -0.25574

ANKE -0.238771

ANKF -0.0841634

ANKG -0.894719

ANKH -0.12037

ANKI -0.335561

ANKK -0.353479

ANKL -0.593572

ANKM -0.0828085

ANKN -0.135262

ANKP -0.549273

ANKQ -0.166685

ANKR -0.3588

ANKS -0.33616

ANKT -0.289766

ANKV -0.476233

ANKW 0.210559

ANKY 0.0451891

ANLA -0.723479

ANLC 0.491849

ANLD -0.632591

ANLE -0.644779

ANLF -0.0171227

ANLG -0.970846

ANLH 0.10685

ANLI -0.323289

ANLK -0.553139

ANLL -0.652464

ANLM 0.325184

ANLN -0.378969

ANLP -0.524251

ANLQ -0.237119

ANLR -0.348859

ANLS -0.627868

ANLT -0.493033

ANLV -0.513472

ANLW 0.544298

ANLY 0.0168457

ANMA 0.0195773

ANMC 0.754674

ANMD -0.0332658

ANME -0.0332315

ANMF 0.722708

ANMG -0.529422

ANMH 0.379667

ANMI 0.469225

ANMK 0.0146337

ANML 0.254401

ANMM 0.875891

ANMN 0.0495286

ANMP -0.153095

ANMQ 0.182199

ANMR 0.187469

ANMS -0.0434339

ANMT 0.113027

ANMV 0.316074

ANMW 0.789227

ANMY 0.677455

ANNA -0.507779

ANNC 0.222475

ANND -0.177824

ANNE -0.297946

ANNF 0.0579555

ANNG -0.62552

ANNH 0.143744

ANNI -0.239524

ANNK -0.204933

ANNL -0.441973

ANNM 0.0473113

ANNN 0.0657382

ANNP -0.310678

ANNQ 0.00317764

ANNR -0.0924895

ANNS -0.228658

ANNT -0.146485

ANNV -0.335433

ANNW 0.490439

ANNY 0.221068

ANPA -0.501727

ANPC 0.173872

ANPD -0.275661

ANPE -0.270762

ANPF 0.10011

ANPG -0.654952

ANPH 0.106846

ANPI -0.214143

ANPK -0.324578

ANPL -0.412198

ANPM 0.0931957

ANPN -0.0698411

ANPP -0.330237

ANPQ -0.0457995

ANPR -0.116742

ANPS -0.26173

ANPT -0.210068

ANPV -0.268845

ANPW 0.540014

ANPY 0.198686

ANQA -0.340604

ANQC 0.381872

ANQD -0.196103

ANQE -0.20368

ANQF 0.21992

ANQG -0.589534

ANQH 0.118217

ANQI -0.0946183

ANQK -0.120027

ANQL -0.257995

ANQM 0.13532

ANQN 0.0265708

ANQP -0.316929

ANQQ 0.225314

ANQR 0.0166633

ANQS -0.182746

ANQT -0.0880601

ANQV -0.201776

ANQW 0.521399

ANQY 0.327952

ANRA -0.467788

ANRC 0.27652

ANRD -0.0735846

ANRE -0.105852

ANRF 0.137886

ANRG -0.727069

ANRH 0.163606

ANRI -0.139854

ANRK -0.366673

ANRL -0.369687

ANRM 0.218512

ANRN -0.111546

ANRP -0.295299

ANRQ 0.0250814

ANRR -0.0361362

ANRS -0.273694

ANRT -0.154515

ANRV -0.270181

ANRW 0.47468

ANRY 0.253232

ANSA -0.65351

ANSC 0.26964

ANSD -0.333663

ANSE -0.424695

ANSF -0.036509

ANSG -0.701396

ANSH 0.084348

ANSI -0.358064

ANSK -0.391563

ANSL -0.560686

ANSM 0.00356507

ANSN 0.0949085

ANSP -0.499485

ANSQ -0.0819814

ANSR -0.220438

ANSS -0.328059

ANST -0.225992

ANSV -0.474228

ANSW 0.323796

ANSY 0.0843463

ANTA -0.555784

ANTC 0.35525

ANTD -0.320927

ANTE -0.341624

ANTF 0.0741391

ANTG -0.684078

ANTH 0.137866

ANTI -0.206688

ANTK -0.335271

ANTL -0.4825

ANTM 0.151687

ANTN 0.178849

ANTP -0.374197

ANTQ -0.0411298

ANTR -0.108395

ANTS -0.226874

ANTT -0.211914

ANTV -0.329273

ANTW 0.436451

ANTY 0.17287

ANVA -0.589363

ANVC 0.492358

ANVD -0.510964

ANVE -0.506771

ANVF 0.100832

ANVG -0.865802

ANVH 0.119139

ANVI -0.185196

ANVK -0.491262

ANVL -0.474169

ANVM 0.368701

ANVN -0.0580802

ANVP -0.437505

ANVQ -0.204273

ANVR -0.285138

ANVS -0.395713

ANVT -0.36081

ANVV -0.360364

ANVW 0.566795

ANVY 0.115913

ANWA 0.191178

ANWC 1.07126

ANWD 0.24703

ANWE 0.221126

ANWF 0.977745

ANWG -0.0520818

ANWH 0.679027

ANWI 0.723769

ANWK 0.253842

ANWL 0.554243

ANWM 0.877388

ANWN 0.441082

ANWP 0.273817

ANWQ 0.536197

ANWR 0.526766

ANWS 0.273441

ANWT 0.348609

ANWV 0.597179

ANWW 1.22356

ANWY 1.07037

ANYA -0.162977

ANYC 0.980407

ANYD 0.0524664

ANYE -0.0644717

ANYF 0.585022

ANYG -0.321202

ANYH 0.556633

ANYI 0.265565

ANYK 0.0526392

ANYL -0.0046525

ANYM 0.649933

ANYN 0.231674

ANYP 0.0956821

ANYQ 0.299273

ANYR 0.244054

ANYS 0.055191

ANYT 0.126279

ANYV 0.100767

ANYW 1.0241

ANYY 0.664225

APAA -0.525794

APAC 0.186744

APAD -0.532437

APAE -0.531381

APAF -0.0158477

APAG -0.967628

APAH -0.00637174

APAI -0.275223

APAK -0.459947

APAL -0.560461

APAM 0.171985

APAN -0.398903

APAP 3.29491

APAQ -0.247943

APAR -0.308626

APAS -0.498959

APAT -0.421787

APAV -0.450112

APAW 0.342252

APAY 0.0336399

APCA 0.244322

APCC 2.27951

APCD 0.0724089

APCE 0.0143688

APCF 0.856377

APCG -0.0477808

APCH 0.750754

APCI 0.582301

APCK 0.225782

APCL 0.510996

APCM 0.741407

APCN 0.289064

APCP 0.196041

APCQ 0.467741

APCR 0.440412

APCS 0.296151

APCT 0.286143

APCV 0.559954

APCW 1.12907

APCY 0.887465

APDA -0.493148

APDC 0.0889399

APDD -0.287512

APDE -0.334634

APDF 0.0174685

APDG -0.638971

APDH 0.212959

APDI -0.0164716

APDK -0.0879066

APDL -0.502848

APDM -0.0123899

APDN -0.0133069

APDP -0.383404

APDQ -0.0514796

APDR 0.0679698

APDS -0.219722

APDT -0.166339

APDV -0.350343

APDW 0.372831

APDY 0.202358

APEA -0.572002

APEC 0.0262597

APED -0.420192

APEE -0.342899

APEF -0.0480475

APEG -0.890572

APEH 0.119687

APEI -0.286455

APEK -0.125965

APEL -0.456778

APEM 0.0111949

APEN -0.202314

APEP -0.494869

APEQ -0.0786111

APER 0.0438619

APES -0.403387

APET -0.304447

APEV -0.40255

APEW 0.384913

APEY 0.0789413

APFA -0.0611539

APFC 0.873949

APFD 0.0534391

APFE -0.0322762

APFF 0.701816

APFG -0.367365

APFH 0.627181

APFI 0.393236

APFK 0.0427487

APFL 0.0987821

APFM 0.822562

APFN 0.157385

APFP 0.123353

APFQ 0.287513

APFR 0.22426

APFS 0.0896106

APFT 0.159952

APFV 0.217325

APFW 1.04649

APFY 0.692569

APGA -0.971862

APGC -0.165765

APGD -0.62415

APGE -0.751408

APGF -0.288653

APGG -1.77458

APGH -0.348868

APGI -0.583175

APGK -0.60129

APGL -0.613012

APGM -0.304976

APGN -0.494437

APGP -0.73204

APGQ -0.465214

APGR -0.454515

APGS -0.627553

APGT -0.508092

APGV -0.67215

APGW -0.0368979

APGY -0.175917

APHA -0.0847819

APHC 0.67938

APHD 0.260477

APHE 0.135049

APHF 0.621259

APHG -0.362363

APHH 0.736505

APHI 0.270917

APHK -0.0147943

APHL 0.209109

APHM 0.553153

APHN 0.20877

APHP 0.0274708

APHQ 0.204713

APHR 0.278616

APHS 0.154649

APHT 0.230565

APHV 0.197744

APHW 0.937703

APHY 0.685899

APIA -0.278517

APIC 0.668355

APID -0.25297

APIE -0.279615

APIF 0.407437

APIG -0.631923

APIH 0.320916

APII 0.192239

APIK -0.202651

APIL -0.178283

APIM 0.619182

APIN -0.0917807

APIP -0.178058

APIQ 0.0401311

APIR -0.0180514

APIS -0.205009

APIT -0.056174

APIV -0.0245118

APIW 0.784371

APIY 0.442428

APKA -0.538211

APKC 0.159449

APKD -0.132576

APKE -0.141638

APKF 0.0177138

APKG -0.795478

APKH -0.0334029

APKI -0.22598

APKK -0.264927

APKL -0.420515

APKM 0.0044322

APKN -0.160846

APKP -0.211056

APKQ -0.0420718

APKR -0.248834

APKS -0.319845

APKT -0.243343

APKV -0.356682

APKW 0.288054

APKY 0.166984

APLA -0.601172

APLC 0.609198

APLD -0.507593

APLE -0.517845

APLF 0.108256

APLG -0.846761

APLH 0.19068

APLI -0.166888

APLK -0.442063

APLL -0.524372

APLM 0.392378

APLN -0.335488

APLP -0.328498

APLQ -0.0983441

APLR -0.236723

APLS -0.473924

APLT -0.349684

APLV -0.354148

APLW 0.665232

APLY 0.155025

APMA 0.128243

APMC 0.818115

APMD 0.0510228

APME 0.0608222

APMF 0.823562

APMG -0.45463

APMH 0.447043

APMI 0.585673

APMK 0.0912209

APML 0.39103

APMM 0.8761

APMN 0.102912

APMP -0.0619857

APMQ 0.259625

APMR 0.28535

APMS 0.0355937

APMT 0.211186

APMV 0.433097

APMW 0.85126

APMY 0.775424

APNA -0.389512

APNC 0.294045

APND -0.0729825

APNE -0.18439

APNF 0.173497

APNG -0.519647

APNH 0.227803

APNI -0.128976

APNK -0.0883217

APNL -0.317932

APNM 0.111943

APNN 0.14678

APNP -0.176747

APNQ 0.121901

APNR 0.0225251

APNS -0.0788634

APNT -0.0420039

APNV -0.226139

APNW 0.584293

APNY 0.336074

APPA -0.30738

APPC 0.243035

APPD -0.149487

APPE -0.151371

APPF 0.210722

APPG -0.547691

APPH 0.195042

APPI -0.10386

APPK -0.217497

APPL -0.291998

APPM 0.17419

APPN -0.0715249

APPP -0.0373998

APPQ 0.0485532

APPR -0.00565147

APPS -0.112598

APPT -0.0873046

APPV -0.145286

APPW 0.622411

APPY 0.298758

APQA -0.223326

APQC 0.452178

APQD -0.0993388

APQE -0.0804913

APQF 0.326391

APQG -0.487281

APQH 0.194515

APQI 0.030798

APQK -0.0217824

APQL -0.138685

APQM 0.209007

APQN 0.0854278

APQP -0.201448

APQQ 0.298805

APQR 0.128865

APQS -0.0459526

APQT 0.0350025

APQV -0.0716338

APQW 0.603283

APQY 0.435782

APRA -0.368187

APRC 0.35327

APRD 0.0341449

APRE 0.0143671

APRF 0.256779

APRG -0.626368

APRH 0.258925

APRI -0.0160778

APRK -0.247387

APRL -0.236938

APRM 0.30856

APRN -0.0350373

APRP -0.0700951

APRQ 0.133656

APRR 0.0597215

APRS -0.14732

APRT -0.108941

APRV -0.153352

APRW 0.55668

APRY 0.359245

APSA -0.532537

APSC 0.346347

APSD -0.242588

APSE -0.2995

APSF 0.0978966

APSG -0.610929

APSH 0.181978

APSI -0.248313

APSK -0.261216

APSL -0.457506

APSM 0.0867226

APSN -0.0791538

APSP -0.0718818

APSQ 0.0298545

APSR -0.098484

APSS -0.168787

APST -0.12684

APSV -0.14474

APSW 0.411776

APSY 0.1877

APTA -0.427222

APTC 0.445957

APTD -0.19138

APTE -0.219285

APTF 0.201763

APTG -0.578135

APTH 0.228567

APTI -0.0810611

APTK -0.207865

APTL -0.334107

APTM 0.240077

APTN -0.030206

APTP -0.0697727

APTQ 0.0788753

APTR -0.0347824

APTS -0.16679

APTT -0.0590496

APTV -0.222651

APTW 0.524562

APTY 0.289682

APVA -0.399909

APVC 0.599801

APVD -0.377177

APVE -0.408798

APVF 0.234771

APVG -0.750232

APVH 0.212595

APVI -0.0221601

APVK -0.32153

APVL -0.377411

APVM 0.456399

APVN -0.233523

APVP -0.130697

APVQ -0.0791879

APVR -0.168435

APVS -0.347796

APVT -0.200677

APVV -0.203623

APVW 0.670336

APVY 0.253264

APWA 0.283635

APWC 1.13316

APWD 0.32777

APWE 0.305359

APWF 1.07979

APWG 0.0294235

APWH 0.735752

APWI 0.825583

APWK 0.340863

APWL 0.671578

APWM 0.944457

APWN 0.506465

APWP 0.347992

APWQ 0.611011

APWR 0.613982

APWS 0.34235

APWT 0.428259

APWV 0.702652

APWW 1.28938

APWY 1.16937

APYA -0.0422912

APYC 1.07481

APYD 0.172668

APYE 0.0544648

APYF 0.710099

APYG -0.202762

APYH 0.634584

APYI 0.391865

APYK 0.159644

APYL 0.114161

APYM 0.747817

APYN 0.316621

APYP 0.247662

APYQ 0.414792

APYR 0.359243

APYS 0.129969

APYT 0.235945

APYV 0.228373

APYW 1.12101

APYY 0.777833

AQAA -0.592028

AQAC 0.101467

AQAD -0.599575

AQAE -0.240253

AQAF -0.119893

AQAG -1.0509

AQAH 0.0842793

AQAI -0.369862

AQAK -0.305616

AQAL -0.597011

AQAM 0.0921781

AQAN -0.424896

AQAP -0.757963

AQAQ 4.08074

AQAR 0.0653191

AQAS -0.503588

AQAT -0.471595

AQAV -0.54903

AQAW 0.249454

AQAY -0.0680299

AQCA 0.164848

AQCC 2.18281

AQCD 0.00999331

AQCE -0.0465119

AQCF 0.778827

AQCG -0.117387

AQCH 0.691671

AQCI 0.49891

AQCK 0.157431

AQCL 0.410998

AQCM 0.68573

AQCN 0.223238

AQCP 0.108031

AQCQ 0.412699

AQCR 0.364964

AQCS 0.220084

AQCT 0.210929

AQCV 0.463025

AQCW 1.07337

AQCY 0.812714

AQDA -0.579937

AQDC 0.0242426

AQDD -0.397342

AQDE -0.436632

AQDF -0.0528836

AQDG -0.751198

AQDH 0.119944

AQDI -0.358117

AQDK -0.203136

AQDL -0.60625

AQDM -0.0859611

AQDN -0.116394

AQDP -0.486055

AQDQ -0.124347

AQDR -0.0687914

AQDS -0.332551

AQDT -0.266745

AQDV -0.465463

AQDW 0.297659

AQDY 0.0993376

AQEA -0.657403

AQEC -0.0401504

AQED -0.520286

AQEE -0.471194

AQEF -0.125693

AQEG -0.989357

AQEH 0.0349715

AQEI -0.397871

AQEK -0.198196

AQEL -0.620212

AQEM -0.0605528

AQEN -0.284778

AQEP -0.619787

AQEQ -0.0599992

AQER -0.111831

AQES -0.52002

AQET -0.419178

AQEV -0.551709

AQEW 0.311955

AQEY -0.0329623

AQFA -0.170761

AQFC 0.793393

AQFD -0.0539951

AQFE -0.148497

AQFF 0.581111

AQFG -0.457019

AQFH 0.537763

AQFI 0.270391

AQFK -0.0657938

AQFL -0.0323653

AQFM 0.72671

AQFN 0.0670135

AQFP -0.03262

AQFQ 0.239284

AQFR 0.114028

AQFS -0.0255437

AQFT 0.0537448

AQFV 0.0901668

AQFW 0.960025

AQFY 0.578493

AQGA -1.08758

AQGC -0.230879

AQGD -0.739542

AQGE -0.850817

AQGF -0.383614

AQGG -1.8394

AQGH -0.434084

AQGI -0.686534

AQGK -0.708652

AQGL -0.896213

AQGM -0.368461

AQGN -0.581022

AQGP -0.912061

AQGQ -0.481591

AQGR -0.554308

AQGS -0.731445

AQGT -0.605087

AQGV -0.768913

AQGW -0.0973446

AQGY -0.275735

AQHA -0.111683

AQHC 0.623209

AQHD 0.16876

AQHE 0.0525329

AQHF 0.533605

AQHG -0.43493

AQHH 0.677748

AQHI 0.185819

AQHK -0.0990992

AQHL 0.0923259

AQHM 0.489422

AQHN 0.131309

AQHP -0.0390384

AQHQ 0.184304

AQHR 0.189586

AQHS 0.0944364

AQHT 0.152654

AQHV 0.111985

AQHW 0.871886

AQHY 0.591601

AQIA -0.419656

AQIC 0.581828

AQID -0.366577

AQIE -0.386912

AQIF 0.28171

AQIG -0.731945

AQIH 0.256699

AQII 0.0512137

AQIK -0.320375

AQIL -0.311042

AQIM 0.517959

AQIN -0.183594

AQIP -0.305184

AQIQ -0.0534315

AQIR -0.12246

AQIS -0.299772

AQIT -0.166748

AQIV -0.0734696

AQIW 0.695873

AQIY 0.341719

AQKA -0.619133

AQKC 0.0902805

AQKD -0.236911

AQKE -0.21114

AQKF -0.0827701

AQKG -0.911714

AQKH -0.113533

AQKI -0.329844

AQKK -0.37138

AQKL -0.578712

AQKM -0.0688066

AQKN -0.266225

AQKP -0.539523

AQKQ -0.108841

AQKR -0.357155

AQKS -0.423538

AQKT -0.366085

AQKV -0.459085

AQKW 0.216513

AQKY 0.107968

AQLA -0.624602

AQLC 0.503967

AQLD -0.617507

AQLE -0.617961

AQLF -0.0241327

AQLG -0.956621

AQLH 0.0917866

AQLI -0.323941

AQLK -0.538489

AQLL -0.661072

AQLM 0.286546

AQLN -0.445308

AQLP -0.510193

AQLQ -0.176381

AQLR -0.306525

AQLS -0.594622

AQLT -0.456923

AQLV -0.509856

AQLW 0.556973

AQLY 0.0252709

AQMA 0.0329216

AQMC 0.760746

AQMD -0.0287354

AQME -0.0146425

AQMF 0.729423

AQMG -0.524427

AQMH 0.386062

AQMI 0.489754

AQMK 0.0262427

AQML 0.277337

AQMM 0.802664

AQMN 0.024765

AQMP -0.144262

AQMQ 0.18058

AQMR 0.195894

AQMS -0.0493147

AQMT 0.117706

AQMV 0.346728

AQMW 0.793425

AQMY 0.684953

AQNA -0.415678

AQNC 0.231642

AQND -0.16596

AQNE -0.301468

AQNF 0.0677245

AQNG -0.622399

AQNH 0.146372

AQNI -0.229185

AQNK -0.189558

AQNL -0.416896

AQNM 0.0428982

AQNN 0.0242791

AQNP -0.302987

AQNQ 0.153149

AQNR -0.0666711

AQNS -0.211058

AQNT -0.12555

AQNV -0.342584

AQNW 0.503066

AQNY 0.23472

AQPA -0.481256

AQPC 0.181835

AQPD -0.252463

AQPE -0.267719

AQPF 0.109787

AQPG -0.646788

AQPH 0.118668

AQPI -0.198038

AQPK -0.312453

AQPL -0.397861

AQPM 0.102712

AQPN -0.161649

AQPP -0.31886

AQPQ 0.0510509

AQPR -0.100599

AQPS -0.254372

AQPT -0.190371

AQPV -0.252764

AQPW 0.547686

AQPY 0.208425

AQQA -0.326346

AQQC 0.388679

AQQD -0.199176

AQQE -0.175686

AQQF 0.231713

AQQG -0.570416

AQQH 0.133744

AQQI -0.0908799

AQQK -0.126015

AQQL -0.240336

AQQM 0.144559

AQQN -0.00659561

AQQP -0.308863

AQQQ 0.223336

AQQR 0.0392063

AQQS -0.151397

AQQT -0.0698588

AQQV -0.193995

AQQW 0.529

AQQY 0.333564

AQRA -0.474518

AQRC 0.288824

AQRD -0.0797215

AQRE -0.133985

AQRF 0.148804

AQRG -0.717971

AQRH 0.178301

AQRI -0.119211

AQRK -0.337192

AQRL -0.375177

AQRM 0.229899

AQRN -0.131509

AQRP -0.292134

AQRQ 0.0588562

AQRR -0.0444579

AQRS -0.242599

AQRT -0.244626

AQRV -0.264371

AQRW 0.483738

AQRY 0.256019

AQSA -0.620314

AQSC 0.270836

AQSD -0.324175

AQSE -0.452219

AQSF -0.0242114

AQSG -0.70812

AQSH 0.0999887

AQSI -0.349669

AQSK -0.36402

AQSL -0.548813

AQSM 0.00637031

AQSN -0.138963

AQSP -0.484562

AQSQ 0.32503

AQSR -0.18241

AQSS -0.310048

AQST -0.243757

AQSV -0.445303

AQSW 0.345044

AQSY 0.0728593

AQTA -0.542827

AQTC 0.365986

AQTD -0.311362

AQTE -0.351693

AQTF 0.0791397

AQTG -0.689241

AQTH 0.133563

AQTI -0.192556

AQTK -0.320975

AQTL -0.474644

AQTM 0.153307

AQTN 0.0816791

AQTP -0.360775

AQTQ 0.109529

AQTR -0.0932188

AQTS -0.280267

AQTT -0.200187

AQTV -0.321126

AQTW 0.443541

AQTY 0.176013

AQVA -0.565332

AQVC 0.502388

AQVD -0.49

AQVE -0.46904

AQVF 0.123698

AQVG -0.858704

AQVH 0.110132

AQVI -0.165576

AQVK -0.478466

AQVL -0.558968

AQVM 0.350531

AQVN -0.310044

AQVP -0.42744

AQVQ -0.0832977

AQVR -0.156719

AQVS -0.470889

AQVT -0.307641

AQVV 0.0458422

AQVW 0.572151

AQVY 0.14141

AQWA 0.197912

AQWC 1.07747

AQWD 0.261166

AQWE 0.232513

AQWF 0.990297

AQWG -0.0435107

AQWH 0.677875

AQWI 0.733292

AQWK 0.26273

AQWL 0.564412

AQWM 0.886147

AQWN 0.434874

AQWP 0.282255

AQWQ 0.547772

AQWR 0.534531

AQWS 0.268559

AQWT 0.350347

AQWV 0.606431

AQWW 1.23062

AQWY 1.08039

AQYA -0.1274

AQYC 0.989919

AQYD 0.0685925

AQYE -0.0492954

AQYF 0.598534

AQYG -0.307191

AQYH 0.549669

AQYI 0.273829

AQYK 0.0497596

AQYL 0.0194101

AQYM 0.677138

AQYN 0.225361

AQYP 0.0987968

AQYQ 0.32385

AQYR 0.257354

AQYS 0.0203648

AQYT 0.125437

AQYV 0.125041

AQYW 1.03148

AQYY 0.670458

ARAA -0.648049

ARAC -0.0102332

ARAD -0.746025

ARAE -0.763647

ARAF -0.259008

ARAG -1.16751

ARAH -0.110701

ARAI -0.499007

ARAK -0.23455

ARAL -0.751338

ARAM -0.0341389

ARAN -0.478413

ARAP -0.876756

ARAQ 0.00720906

ARAR 4.26976

ARAS -0.692811

ARAT -0.565876

ARAV -0.640324

ARAW 0.156528

ARAY -0.223459

ARCA 0.0365651

ARCC 2.04258

ARCD -0.0992653

ARCE -0.150548

ARCF 0.655322

ARCG -0.228747

ARCH 0.599819

ARCI 0.379297

ARCK 0.0462642

ARCL 0.271007

ARCM 0.585576

ARCN 0.119725

ARCP 0.00624776

ARCQ 0.299169

ARCR 0.298205

ARCS 0.0982144

ARCT 0.0959117

ARCV 0.346488

ARCW 0.974447

ARCY 0.694797

ARDA -0.76351

ARDC -0.0851591

ARDD -0.546145

ARDE -0.585751

ARDF -0.227235

ARDG -0.865046

ARDH -0.00753427

ARDI -0.516836

ARDK -0.32834

ARDL -0.744518

ARDM -0.200373

ARDN -0.261542

ARDP -0.619419

ARDQ -0.311849

ARDR 0.0189176

ARDS -0.471173

ARDT -0.428298

ARDV -0.632793

ARDW 0.181418

ARDY -0.0436773

AREA -0.793568

AREC -0.149221

ARED -0.669667

AREE -0.623483

AREF -0.285735

AREG -1.11738

AREH -0.0998313

AREI -0.556316

AREK -0.362735

AREL -0.789087

AREM -0.182175

AREN -0.440241

AREP -0.743487

AREQ -0.352201

ARER -0.0937266

ARES -0.667539

ARET -0.574578

AREV -0.597762

AREW 0.187162

AREY -0.177328

ARFA -0.332106

ARFC 0.670533

ARFD -0.193245

ARFE -0.284581

ARFF 0.421892

ARFG -0.600404

ARFH 0.404051

ARFI 0.126008

ARFK -0.217965

ARFL -0.2126

ARFM 0.592991

ARFN -0.0698416

ARFP -0.167172

ARFQ 0.1454

ARFR 0.0148807

ARFS -0.152908

ARFT -0.0981398

ARFV -0.0530374

ARFW 0.834581

ARFY 0.428515

ARGA -1.20133

ARGC -0.338368

ARGD -0.868024

ARGE -1.006

ARGF -0.517687

ARGG -1.94723

ARGH -0.548535

ARGI -0.823539

ARGK -0.781232

ARGL -1.04344

ARGM -0.490942

ARGN -0.720267

ARGP -1.04432

ARGQ -0.685045

ARGR -0.350114

ARGS -0.879791

ARGT -0.755757

ARGV -0.913293

ARGW -0.201032

ARGY -0.410868

ARHA -0.301488

ARHC 0.524493

ARHD 0.0469043

ARHE -0.0875609

ARHF 0.404146

ARHG -0.550886

ARHH 0.552502

ARHI 0.0588026

ARHK -0.199581

ARHL -0.0615599

ARHM 0.38488

ARHN 0.0128231

ARHP -0.160098

ARHQ 0.0588017

ARHR 0.0903633

ARHS -0.0566833

ARHT 0.0121014

ARHV -0.019731

ARHW 0.766509

ARHY 0.471064

ARIA -0.569985

ARIC 0.455004

ARID -0.500836

ARIE -0.505914

ARIF 0.135961

ARIG -0.867424

ARIH 0.0928106

ARII -0.104377

ARIK -0.439424

ARIL -0.46916

ARIM 0.391047

ARIN -0.321985

ARIP -0.433291

ARIQ -0.223237

ARIR -0.240678

ARIS -0.450513

ARIT -0.309215

ARIV -0.251959

ARIW 0.561558

ARIY 0.201523

ARKA -0.788741

ARKC -0.0204711

ARKD -0.398847

ARKE -0.409498

ARKF -0.230885

ARKG -1.03172

ARKH -0.232622

ARKI -0.470151

ARKK -0.529595

ARKL -0.73818

ARKM -0.185769

ARKN -0.407557

ARKP -0.668842

ARKQ -0.312583

ARKR -0.365418

ARKS -0.577873

ARKT -0.49914

ARKV -0.609724

ARKW 0.107044

ARKY -0.0871537

ARLA -0.854206

ARLC 0.365076

ARLD -0.776154

ARLE -0.796136

ARLF -0.179917

ARLG -1.10031

ARLH -0.0333436

ARLI -0.45874

ARLK -0.651741

ARLL -0.825522

ARLM 0.134967

ARLN -0.597603

ARLP -0.647509

ARLQ -0.322894

ARLR -0.37472

ARLS -0.715851

ARLT -0.629838

ARLV -0.68269

ARLW 0.416791

ARLY -0.126664

ARMA -0.0938981

ARMC 0.659287

ARMD -0.146198

ARME -0.13521

ARMF 0.598526

ARMG -0.63864

ARMH 0.281642

ARMI 0.346169

ARMK -0.0997448

ARML 0.127443

ARMM 0.694698

ARMN -0.0778885

ARMP -0.251879

ARMQ 0.0759964

ARMR 0.0941775

ARMS -0.163035

ARMT -0.00069594

ARMV 0.266327

ARMW 0.697165

ARMY 0.552662

ARNA -0.607098

ARNC 0.124242

ARND -0.324637

ARNE -0.425403

ARNF -0.0645902

ARNG -0.758684

ARNH 0.038197

ARNI -0.35497

ARNK -0.303397

ARNL -0.591485

ARNM -0.0667233

ARNN -0.110555

ARNP -0.426832

ARNQ -0.125792

ARNR -0.197007

ARNS -0.345992

ARNT -0.303844

ARNV -0.471195

ARNW 0.383565

ARNY 0.107411

ARPA -0.627221

ARPC 0.0761158

ARPD -0.401923

ARPE -0.408166

ARPF -0.0248537

ARPG -0.779028

ARPH -0.00265956

ARPI -0.354833

ARPK -0.459223

ARPL -0.54458

ARPM -0.0113361

ARPN -0.30296

ARPP -0.446296

ARPQ -0.168362

ARPR -0.154005

ARPS -0.390645

ARPT -0.342659

ARPV -0.404932

ARPW 0.431573

ARPY 0.0617456

ARQA -0.474657

ARQC 0.282537

ARQD -0.327848

ARQE -0.333381

ARQF 0.0961812

ARQG -0.710843

ARQH 0.0810089

ARQI -0.243203

ARQK -0.288061

ARQL -0.38249

ARQM 0.0289984

ARQN -0.0947118

ARQP -0.429552

ARQQ 0.248687

ARQR -0.0621765

ARQS -0.286352

ARQT -0.228236

ARQV -0.190114

ARQW 0.420612

ARQY 0.201101

ARRA -0.611022

ARRC 0.173881

ARRD -0.21208

ARRE -0.260309

ARRF 0.00948572

ARRG -0.849895

ARRH 0.0619595

ARRI -0.268742

ARRK -0.42402

ARRL -0.494347

ARRM 0.119035

ARRN -0.267199

ARRP -0.417346

ARRQ -0.12784

ARRR -0.128592

ARRS -0.389516

ARRT -0.366172

ARRV -0.419604

ARRW 0.36805

ARRY 0.112618

ARSA -0.792965

ARSC 0.155926

ARSD -0.506478

ARSE -0.577584

ARSF -0.166277

ARSG -0.847091

ARSH -0.0363472

ARSI -0.438095

ARSK -0.527192

ARSL -0.721014

ARSM -0.113962

ARSN -0.308098

ARSP -0.620571

ARSQ -0.0527837

ARSR 0.0689044

ARSS -0.430958

ARST -0.41285

ARSV -0.518199

ARSW 0.216907

ARSY -0.030313

ARTA -0.720561

ARTC 0.246353

ARTD -0.452837

ARTE -0.478547

ARTF -0.0604382

ARTG -0.79009

ARTH 0.0149763

ARTI -0.349976

ARTK -0.493265

ARTL -0.623752

ARTM 0.0325472

ARTN -0.297199

ARTP -0.485969

ARTQ -0.2121

ARTR 0.244097

ARTS -0.407673

ARTT -0.331907

ARTV -0.499067

ARTW 0.326251

ARTY 0.0348454

ARVA -0.740123

ARVC 0.369869

ARVD -0.644872

ARVE -0.657857

ARVF -0.0509708

ARVG -1.00651

ARVH -0.0227489

ARVI -0.285672

ARVK -0.559747

ARVL -0.677846

ARVM 0.203163

ARVN -0.484651

ARVP -0.556609

ARVQ -0.296036

ARVR -0.0425785

ARVS -0.595134

ARVT -0.481704

ARVV -0.459126

ARVW 0.43806

ARVY -0.00870013

ARWA 0.0714438

ARWC 0.97835

ARWD 0.139951

ARWE 0.115064

ARWF 0.862259

ARWG -0.156554

ARWH 0.578742

ARWI 0.602238

ARWK 0.152329

ARWL 0.424011

ARWM 0.779469

ARWN 0.327723

ARWP 0.177835

ARWQ 0.443221

ARWR 0.415169

ARWS 0.151115

ARWT 0.237445

ARWV 0.470179

ARWW 1.12764

ARWY 0.95304

ARYA -0.241727

ARYC 0.864935

ARYD -0.0786519

ARYE -0.179669

ARYF 0.456398

ARYG -0.445106

ARYH 0.423543

ARYI 0.122263

ARYK -0.0835731

ARYL -0.149486

ARYM 0.554428

ARYN 0.0819099

ARYP -0.0234313

ARYQ 0.161168

ARYR 0.144114

ARYS -0.119143

ARYT -0.0136676

ARYV -0.0160267

ARYW 0.904788

ARYY 0.52074

ASAA -0.482527

ASAC 0.0208662

ASAD -0.716697

ASAE -0.745282

ASAF -0.22486

ASAG -1.17968

ASAH -0.201641

ASAI -0.492718

ASAK -0.65592

ASAL -0.787472

ASAM -0.0502856

ASAN -0.317557

ASAP -0.843779

ASAQ -0.338388

ASAR -0.469501

ASAS 4.04101

ASAT -0.179628

ASAV -0.682936

ASAW 0.131884

ASAY -0.203769

ASCA 0.0437419

ASCC 2.05205

ASCD -0.0939157

ASCE -0.146222

ASCF 0.65302

ASCG -0.218907

ASCH 0.599346

ASCI 0.381565

ASCK 0.0436978

ASCL 0.283777

ASCM 0.592333

ASCN 0.134912

ASCP 0.0110404

ASCQ 0.300189

ASCR 0.261863

ASCS 0.159184

ASCT 0.106793

ASCV 0.353253

ASCW 0.981036

ASCY 0.697333

ASDA -0.724854

ASDC -0.0783041

ASDD -0.517558

ASDE -0.58442

ASDF -0.231076

ASDG -0.886665

ASDH -0.00779319

ASDI -0.492501

ASDK -0.347215

ASDL -0.72415

ASDM -0.190063

ASDN -0.246569

ASDP -0.57786

ASDQ -0.228782

ASDR -0.0830007

ASDS -0.387759

ASDT -0.43222

ASDV -0.615904

ASDW 0.191214

ASDY -0.0286989

ASEA -0.802528

ASEC -0.137011

ASED -0.656546

ASEE -0.596653

ASEF -0.285306

ASEG -1.11142

ASEH -0.0879095

ASEI -0.538981

ASEK -0.314336

ASEL -0.736965

ASEM -0.177035

ASEN -0.419805

ASEP -0.723493

ASEQ -0.318586

ASER -0.195721

ASES -0.560925

ASET -0.485263

ASEV -0.669822

ASEW 0.196718

ASEY -0.15912

ASFA -0.332151

ASFC 0.676539

ASFD -0.187625

ASFE -0.283898

ASFF 0.445717

ASFG -0.58617

ASFH 0.415319

ASFI 0.137864

ASFK -0.219631

ASFL -0.188842

ASFM 0.603017

ASFN -0.0666611

ASFP -0.162364

ASFQ 0.055846

ASFR -0.0151482

ASFS -0.104678

ASFT -0.0783162

ASFV -0.0751865

ASFW 0.840714

ASFY 0.439995

ASGA -1.15819

ASGC -0.329273

ASGD -0.838221

ASGE -0.963758

ASGF -0.500968

ASGG -1.93931

ASGH -0.533684

ASGI -0.796409

ASGK -0.847852

ASGL -1.01332

ASGM -0.483873

ASGN -0.680355

ASGP -1.02944

ASGQ -0.677732

ASGR -0.676239

ASGS -0.620809

ASGT -0.714084

ASGV -0.921266

ASGW -0.204067

ASGY -0.421588

ASHA -0.305112

ASHC 0.530812

ASHD 0.0537755

ASHE -0.0629227

ASHF 0.411967

ASHG -0.539788

ASHH 0.558038

ASHI 0.0637717

ASHK -0.190688

ASHL -0.0478313

ASHM 0.393322

ASHN 0.0229268

ASHP -0.154227

ASHQ 0.0301361

ASHR 0.0825107

ASHS -0.0340354

ASHT 0.0176594

ASHV -0.0135126

ASHW 0.773929

ASHY 0.479843

ASIA -0.447598

ASIC 0.463202

ASID -0.498746

ASIE -0.526202

ASIF 0.142845

ASIG -0.868457

ASIH 0.0961518

ASII -0.0899177

ASIK -0.499924

ASIL -0.468711

ASIM 0.384442

ASIN -0.351999

ASIP -0.434398

ASIQ -0.222615

ASIR -0.291039

ASIS -0.430362

ASIT -0.292312

ASIV -0.331355

ASIW 0.569954

ASIY 0.192446

ASKA -0.785631

ASKC -0.0133624

ASKD -0.38848

ASKE -0.379103

ASKF -0.208012

ASKG -1.01813

ASKH -0.223615

ASKI -0.470282

ASKK -0.477496

ASKL -0.735478

ASKM -0.174343

ASKN -0.366118

ASKP -0.659496

ASKQ -0.25562

ASKR -0.491812

ASKS -0.507428

ASKT -0.435345

ASKV -0.583499

ASKW 0.111609

ASKY -0.093292

ASLA -0.905721

ASLC 0.372263

ASLD -0.759155

ASLE -0.808298

ASLF -0.176198

ASLG -1.11016

ASLH -0.0608385

ASLI -0.478678

ASLK -0.657884

ASLL -0.779264

ASLM 0.147438

ASLN -0.400636

ASLP -0.628757

ASLQ -0.35495

ASLR -0.422156

ASLS -0.681333

ASLT -0.703903

ASLV -0.668395

ASLW 0.435426

ASLY -0.114005

ASMA -0.0883787

ASMC 0.666485

ASMD -0.143533

ASME -0.136173

ASMF 0.600099

ASMG -0.648477

ASMH 0.288118

ASMI 0.360055

ASMK -0.088728

ASML 0.134334

ASMM 0.702783

ASMN 0.00125694

ASMP -0.247522

ASMQ 0.0835876

ASMR 0.0893266

ASMS -0.11683

ASMT 0.00876498

ASMV 0.200847

ASMW 0.698115

ASMY 0.557173

ASNA -0.609647

ASNC 0.129433

ASND -0.309439

ASNE -0.410683

ASNF -0.0580723

ASNG -0.746983

ASNH 0.0479822

ASNI -0.352613

ASNK -0.269076

ASNL -0.580337

ASNM -0.0605817

ASNN -0.0824947

ASNP -0.397746

ASNQ -0.0674381

ASNR -0.207141

ASNS -0.299832

ASNT -0.277382

ASNV -0.47577

ASNW 0.395635

ASNY 0.122093

ASPA -0.630277

ASPC 0.0853193

ASPD -0.384801

ASPE -0.394796

ASPF -0.0179381

ASPG -0.760922

ASPH 0.0158222

ASPI -0.347272

ASPK -0.456065

ASPL -0.53535

ASPM -0.00113177

ASPN -0.285123

ASPP -0.438166

ASPQ -0.16491

ASPR -0.233415

ASPS -0.241527

ASPT -0.32654

ASPV -0.387709

ASPW 0.441809

ASPY 0.0764599

ASQA -0.468191

ASQC 0.28826

ASQD -0.317325

ASQE -0.325207

ASQF 0.104385

ASQG -0.698556

ASQH 0.0214958

ASQI -0.21552

ASQK -0.251019

ASQL -0.388757

ASQM 0.0396748

ASQN -0.13618

ASQP -0.402925

ASQQ 0.0919228

ASQR -0.0358188

ASQS -0.256359

ASQT -0.200451

ASQV -0.325842

ASQW 0.423864

ASQY 0.203529

ASRA -0.60919

ASRC 0.178096

ASRD -0.223761

ASRE -0.224855

ASRF 0.023747

ASRG -0.84762

ASRH 0.0664718

ASRI -0.24858

ASRK -0.467444

ASRL -0.520882

ASRM 0.114031

ASRN -0.272039

ASRP -0.415265

ASRQ -0.104189

ASRR -0.177204

ASRS -0.325304

ASRT -0.389822

ASRV -0.402769

ASRW 0.373413

ASRY 0.1334

ASSA -0.755263

ASSC 0.164485

ASSD -0.493893

ASSE -0.533991

ASSF -0.179656

ASSG -0.823442

ASSH -0.045543

ASSI -0.47282

ASSK -0.499442

ASSL -0.705653

ASSM -0.0845649

ASSN -0.381737

ASSP -0.601213

ASSQ -0.191794

ASSR -0.372122

ASSS 0.0127797

ASST -0.408603

ASSV -0.58519

ASSW 0.222829

ASSY -0.0899954

ASTA -0.688163

ASTC 0.25366

ASTD -0.456055

ASTE -0.470062

ASTF -0.0568571

ASTG -0.806504

ASTH 0.021517

ASTI -0.328744

ASTK -0.488199

ASTL -0.61778

ASTM 0.0378735

ASTN -0.260613

ASTP -0.488103

ASTQ -0.153337

ASTR -0.268488

ASTS -0.00633287

ASTT -0.411995

ASTV -0.470762

ASTW 0.335956

ASTY 0.0264134

ASVA -0.716372

ASVC 0.374178

ASVD -0.635311

ASVE -0.696425

ASVF -0.0270927

ASVG -1.00068

ASVH -0.0478187

ASVI -0.310703

ASVK -0.051887

ASVL -0.587077

ASVM 0.237242

ASVN -0.345803

ASVP -0.55951

ASVQ -0.226935

ASVR -0.386402

ASVS -0.0859549

ASVT -0.14878

ASVV -0.494883

ASVW 0.454218

ASVY -0.00095439

ASWA 0.0824878

ASWC 0.984925

ASWD 0.150192

ASWE 0.119194

ASWF 0.867769

ASWG -0.150053

ASWH 0.585668

ASWI 0.610032

ASWK 0.158697

ASWL 0.43958

ASWM 0.785949

ASWN 0.332432

ASWP 0.181623

ASWQ 0.440252

ASWR 0.434113

ASWS 0.153931

ASWT 0.243966

ASWV 0.484025

ASWW 1.13678

ASWY 0.963058

ASYA -0.304142

ASYC 0.872973

ASYD -0.033905

ASYE -0.107687

ASYF 0.45517

ASYG -0.418345

ASYH 0.437757

ASYI 0.140016

ASYK -0.0681937

ASYL -0.159026

ASYM 0.543103

ASYN 0.097059

ASYP -0.0162954

ASYQ 0.174412

ASYR 0.134372

ASYS 0.0287614

ASYT 0.0042901

ASYV -0.0263865

ASYW 0.915425

ASYY 0.530094

ATAA -0.593145

ATAC -0.0252168

ATAD -0.663567

ATAE -0.779907

ATAF -0.339663

ATAG -1.23208

ATAH -0.267037

ATAI -0.228352

ATAK -0.602359

ATAL -0.806094

ATAM 0.0773022

ATAN -0.460075

ATAP -0.907087

ATAQ -0.446875

ATAR -0.483047

ATAS -0.320108

ATAT 4.19642

ATAV -0.0214584

ATAW 0.0985587

ATAY -0.243426

ATCA -0.00543666

ATCC 2.00846

ATCD -0.131531

ATCE -0.192602

ATCF 0.62307

ATCG -0.256737

ATCH 0.564921

ATCI 0.339324

ATCK 0.00884247

ATCL 0.243418

ATCM 0.560968

ATCN 0.0903029

ATCP -0.020231

ATCQ 0.268564

ATCR 0.220874

ATCS 0.0928247

ATCT 0.102936

ATCV 0.311273

ATCW 0.947177

ATCY 0.660535

ATDA -0.786229

ATDC -0.114056

ATDD -0.56952

ATDE -0.617929

ATDF -0.265399

ATDG -0.918429

ATDH -0.0265615

ATDI -0.535128

ATDK -0.381754

ATDL -0.792986

ATDM -0.232993

ATDN -0.298829

ATDP -0.647055

ATDQ -0.324701

ATDR -0.251773

ATDS -0.508141

ATDT -0.445024

ATDV -0.652303

ATDW 0.150489

ATDY -0.0816722

ATEA -0.858502

ATEC -0.179287

ATED -0.704853

ATEE -0.659657

ATEF -0.321239

ATEG -1.15999

ATEH -0.118773

ATEI -0.568527

ATEK -0.403274

ATEL -0.823324

ATEM -0.214936

ATEN -0.470992

ATEP -0.765609

ATEQ -0.335249

ATER -0.300166

ATES -0.719385

ATET -0.524015

ATEV -0.729038

ATEW 0.166308

ATEY -0.215426

ATFA -0.352651

ATFC 0.636652

ATFD -0.234233

ATFE -0.301467

ATFF 0.403116

ATFG -0.636926

ATFH 0.374309

ATFI 0.0998933

ATFK -0.232875

ATFL -0.250603

ATFM 0.557036

ATFN -0.139843

ATFP -0.205756

ATFQ 0.0245769

ATFR -0.0636768

ATFS 0.109409

ATFT 0.104149

ATFV -0.123857

ATFW 0.803991

ATFY 0.390512

ATGA -1.20918

ATGC -0.367603

ATGD -0.881575

ATGE -1.02402

ATGF -0.558508

ATGG -1.97292

ATGH -0.56998

ATGI -0.75754

ATGK -0.890628

ATGL -1.10163

ATGM -0.477561

ATGN -0.741768

ATGP -1.05629

ATGQ -0.699362

ATGR -0.701385

ATGS -0.901818

ATGT -0.652059

ATGV -0.790876

ATGW -0.234843

ATGY -0.448333

ATHA -0.332756

ATHC 0.498125

ATHD 0.0115097

ATHE -0.10129

ATHF 0.382912

ATHG -0.581406

ATHH 0.526875

ATHI 0.0305262

ATHK -0.241656

ATHL -0.0822494

ATHM 0.356907

ATHN -0.00845289

ATHP -0.189082

ATHQ -0.00641155

ATHR 0.0509012

ATHS -0.10813

ATHT -0.0118811

ATHV -0.0673738

ATHW 0.734495

ATHY 0.437234

ATIA -0.60524

ATIC 0.422701

ATID -0.540128

ATIE -0.537751

ATIF 0.08025

ATIG -0.908933

ATIH 0.0716729

ATII -0.101707

ATIK -0.477896

ATIL -0.506581

ATIM 0.34392

ATIN -0.380506

ATIP -0.477106

ATIQ -0.228789

ATIR -0.309873

ATIS -0.462313

ATIT -0.341083

ATIV -0.345969

ATIW 0.531351

ATIY 0.220625

ATKA -0.76771

ATKC -0.0490255

ATKD -0.388427

ATKE -0.484446

ATKF -0.247905

ATKG -1.06087

ATKH -0.257917

ATKI -0.49239

ATKK -0.577441

ATKL -0.733055

ATKM -0.220904

ATKN -0.389883

ATKP -0.696659

ATKQ -0.289657

ATKR -0.521014

ATKS -0.637528

ATKT -0.335256

ATKV -0.632006

ATKW 0.0726857

ATKY -0.141494

ATLA -0.913155

ATLC 0.330661

ATLD -0.80874

ATLE -0.853763

ATLF -0.225459

ATLG -1.16534

ATLH -0.0882308

ATLI -0.50549

ATLK -0.729379

ATLL -0.852297

ATLM 0.107175

ATLN -0.646984

ATLP -0.693521

ATLQ -0.402059

ATLR -0.506311

ATLS -0.627703

ATLT -0.618788

ATLV -0.712839

ATLW 0.383638

ATLY -0.170086

ATMA -0.132739

ATMC 0.631847

ATMD -0.178145

ATME -0.151908

ATMF 0.560492

ATMG -0.666795

ATMH 0.260251

ATMI 0.317588

ATMK -0.138497

ATML 0.101865

ATMM 0.669421

ATMN -0.112841

ATMP -0.277787

ATMQ 0.0458069

ATMR 0.0549471

ATMS -0.201521

ATMT -0.0265582

ATMV 0.163409

ATMW 0.664322

ATMY 0.537618

ATNA -0.695028

ATNC 0.0944562

ATND -0.353071

ATNE -0.474458

ATNF -0.104532

ATNG -0.780302

ATNH 0.00394154

ATNI -0.396101

ATNK -0.359735

ATNL -0.544433

ATNM -0.0978794

ATNN -0.120392

ATNP -0.455436

ATNQ -0.145953

ATNR -0.25014

ATNS -0.330059

ATNT -0.272976

ATNV -0.477796

ATNW 0.360391

ATNY 0.0708511

ATPA -0.675202

ATPC 0.0465415

ATPD -0.431818

ATPE -0.437108

ATPF -0.0526495

ATPG -0.808878

ATPH -0.0321372

ATPI -0.386245

ATPK -0.493543

ATPL -0.581593

ATPM -0.0402062

ATPN -0.321019

ATPP -0.48074

ATPQ -0.203809

ATPR -0.273624

ATPS -0.40662

ATPT -0.208178

ATPV -0.462193

ATPW 0.401659

ATPY 0.0393481

ATQA -0.50811

ATQC 0.254796

ATQD -0.363664

ATQE -0.35767

ATQF 0.0499232

ATQG -0.727175

ATQH -0.0183673

ATQI -0.260448

ATQK -0.28838

ATQL -0.444622

ATQM 0.00597572

ATQN -0.145321

ATQP -0.447042

ATQQ 0.0269938

ATQR -0.154737

ATQS -0.317823

ATQT -0.110421

ATQV -0.386476

ATQW 0.396203

ATQY 0.175961

ATRA -0.63835

ATRC 0.143688

ATRD -0.206828

ATRE -0.29077

ATRF -0.0234942

ATRG -0.876406

ATRH 0.0326955

ATRI -0.308018

ATRK -0.503559

ATRL -0.55087

ATRM 0.0729783

ATRN -0.263553

ATRP -0.444333

ATRQ -0.138618

ATRR -0.222086

ATRS -0.384027

ATRT -0.327996

ATRV -0.441897

ATRW 0.333698

ATRY 0.0525227

ATSA -0.796383

ATSC 0.123736

ATSD -0.547804

ATSE -0.527164

ATSF -0.190001

ATSG -0.87443

ATSH -0.0770247

ATSI -0.502529

ATSK -0.498359

ATSL -0.760027

ATSM -0.141509

ATSN -0.364973

ATSP -0.641229

ATSQ -0.279136

ATSR -0.394243

ATSS -0.43894

ATST -0.0435324

ATSV -0.664857

ATSW 0.182633

ATSY -0.135758

ATTA -0.725713

ATTC 0.220037

ATTD -0.46098

ATTE -0.517829

ATTF -0.103023

ATTG -0.856522

ATTH -0.0200279

ATTI -0.360676

ATTK -0.497221

ATTL -0.676615

ATTM -0.0080955

ATTN -0.315062

ATTP -0.518872

ATTQ -0.168889

ATTR -0.330625

ATTS -0.480646

ATTT -0.153337

ATTV -0.491362

ATTW 0.300328

ATTY -0.0303421

ATVA -0.744435

ATVC 0.335878

ATVD -0.681783

ATVE -0.720362

ATVF -0.082175

ATVG -0.985872

ATVH -0.0436416

ATVI -0.363518

ATVK -0.520133

ATVL -0.765823

ATVM 0.207724

ATVN -0.466424

ATVP -0.600312

ATVQ -0.377232

ATVR -0.461636

ATVS -0.56442

ATVT -0.218282

ATVV -0.575444

ATVW 0.401389

ATVY -0.0669892

ATWA 0.045656

ATWC 0.951221

ATWD 0.107649

ATWE 0.0791271

ATWF 0.82873

ATWG -0.187113

ATWH 0.551294

ATWI 0.571381

ATWK 0.119892

ATWL 0.389113

ATWM 0.750618

ATWN 0.294674

ATWP 0.149261

ATWQ 0.404541

ATWR 0.386131

ATWS 0.118518

ATWT 0.211606

ATWV 0.440009

ATWW 1.1023

ATWY 0.921674

ATYA -0.352203

ATYC 0.833392

ATYD -0.110801

ATYE -0.226173

ATYF 0.439451

ATYG -0.475193

ATYH 0.393563

ATYI 0.0818303

ATYK -0.114311

ATYL -0.187594

ATYM 0.49788

ATYN 0.0443356

ATYP -0.0600638

ATYQ 0.15888

ATYR 0.0687046

ATYS -0.15699

ATYT -0.0492496

ATYV -0.0741055

ATYW 0.895568

ATYY 0.493369

AVAA -0.89649

AVAC -0.313214

AVAD -1.08953

AVAE -1.13111

AVAF -0.498695

AVAG -1.61017

AVAH -0.593088

AVAI 0.152138

AVAK -1.08184

AVAL 0.0379882

AVAM -0.205613

AVAN -1.02168

AVAP -1.27244

AVAQ -0.86134

AVAR -0.894524

AVAS -1.16045

AVAT -0.358488

AVAV 4.45621

AVAW -0.219677

AVAY -0.623502

AVCA -0.336165

AVCC 1.66785

AVCD -0.43781

AVCE -0.496052

AVCF 0.308418

AVCG -0.570838

AVCH 0.267601

AVCI 0.0261755

AVCK -0.301509

AVCL -0.122907

AVCM 0.257546

AVCN -0.210977

AVCP -0.329575

AVCQ -0.0449448

AVCR -0.093694

AVCS -0.244012

AVCT -0.239673

AVCV 0.0582552

AVCW 0.649158

AVCY 0.342712

AVDA -1.12948

AVDC -0.423521

AVDD -0.928493

AVDE -0.969914

AVDF -0.564813

AVDG -1.26566

AVDH -0.363852

AVDI -0.898834

AVDK -0.744389

AVDL -1.20575

AVDM -0.555426

AVDN -0.668198

AVDP -0.986871

AVDQ -0.672389

AVDR -0.609862

AVDS -0.872693

AVDT -0.7586

AVDV -0.926533

AVDW -0.171797

AVDY -0.423976

AVEA -1.21001

AVEC -0.48802

AVED -1.05289

AVEE -1.02791

AVEF -0.696973

AVEG -1.49293

AVEH -0.428721

AVEI -0.93993

AVEK -0.7929

AVEL -1.16617

AVEM -0.565788

AVEN -0.828707

AVEP -1.10872

AVEQ -0.703529

AVER -0.675768

AVES -1.07764

AVET -0.681484

AVEV -0.78349

AVEW -0.172186

AVEY -0.568666

AVFA -0.726929

AVFC 0.307274

AVFD -0.579404

AVFE -0.658297

AVFF 0.0345421

AVFG -0.970522

AVFH 0.041086

AVFI -0.338005

AVFK -0.587076

AVFL -0.659164

AVFM 0.199217

AVFN -0.441289

AVFP -0.539451

AVFQ -0.319105

AVFR -0.402546

AVFS -0.591036

AVFT -0.502371

AVFV -0.309794

AVFW 0.47789

AVFY 0.0390453

AVGA -1.59528

AVGC -0.666471

AVGD -1.24911

AVGE -1.37803

AVGF -0.768844

AVGG -2.27807

AVGH -0.89588

AVGI -1.12688

AVGK -1.25071

AVGL -1.21576

AVGM -0.779413

AVGN -1.09378

AVGP -1.40845

AVGQ -1.0475

AVGR -1.06044

AVGS -1.25786

AVGT -1.15753

AVGV -0.838824

AVGW -0.556118

AVGY -0.796877

AVHA -0.679471

AVHC 0.200224

AVHD -0.325412

AVHE -0.44869

AVHF 0.0384119

AVHG -0.899333

AVHH 0.209526

AVHI -0.300902

AVHK -0.568604

AVHL -0.446339

AVHM 0.0468826

AVHN -0.335827

AVHP -0.501946

AVHQ -0.308009

AVHR -0.284642

AVHS -0.417643

AVHT -0.347823

AVHV -0.321622

AVHW 0.427094

AVHY 0.0982969

AVIA -0.989546

AVIC 0.0943489

AVID -0.896303

AVIE -0.93005

AVIF -0.287922

AVIG -1.26454

AVIH -0.262031

AVII -0.450505

AVIK -0.834095

AVIL -1.0148

AVIM 0.00256872

AVIN -0.719097

AVIP -0.814957

AVIQ -0.588042

AVIR -0.653773

AVIS -0.828006

AVIT -0.689203

AVIV -0.424132

AVIW 0.199596

AVIY -0.24047

AVKA -1.19176

AVKC -0.36139

AVKD -0.797582

AVKE -0.80565

AVKF -0.652638

AVKG -1.39903

AVKH -0.583139

AVKI -0.86363

AVKK -0.915668

AVKL -1.1702

AVKM -0.54772

AVKN -0.791064

AVKP -1.04651

AVKQ -0.672127

AVKR -0.861438

AVKS -0.971521

AVKT -0.91301

AVKV -0.888927

AVKW -0.240049

AVKY -0.430826

AVLA -1.241

AVLC -0.0243251

AVLD -1.17244

AVLE -1.16632

AVLF -0.564305

AVLG -1.48541

AVLH -0.427841

AVLI -0.900299

AVLK -1.12152

AVLL -1.15027

AVLM -0.302917

AVLN -0.980232

AVLP -1.04449

AVLQ -0.787761

AVLR -0.927886

AVLS -1.16377

AVLT -1.04624

AVLV -0.829617

AVLW 0.0276215

AVLY -0.53776

AVMA -0.47142

AVMC 0.332582

AVMD -0.500897

AVME -0.505065

AVMF 0.223833

AVMG -0.978692

AVMH -0.0456853

AVMI -0.0145712

AVMK -0.465344

AVML -0.258095

AVMM 0.369932

AVMN -0.429111

AVMP -0.58855

AVMQ -0.274302

AVMR -0.281748

AVMS -0.533536

AVMT -0.36669

AVMV -0.12348

AVMW 0.362734

AVMY 0.187844

AVNA -1.03632

AVNC -0.210332

AVND -0.708396

AVNE -0.813517

AVNF -0.244418

AVNG -1.13068

AVNH -0.25074

AVNI -0.749856

AVNK -0.720281

AVNL -0.851931

AVNM -0.417439

AVNN -0.480965

AVNP -0.787643

AVNQ -0.496131

AVNR -0.595875

AVNS -0.676652

AVNT -0.626793

AVNV -0.756649

AVNW 0.0345621

AVNY -0.30209

AVPA -1.02986

AVPC -0.263802

AVPD -0.789513

AVPE -0.778034

AVPF -0.39552

AVPG -1.14978

AVPH -0.353172

AVPI -0.656297

AVPK -0.835678

AVPL -0.92422

AVPM -0.360922

AVPN -0.664521

AVPP -0.814841

AVPQ -0.546464

AVPR -0.622381

AVPS -0.775208

AVPT -0.733434

AVPV -0.431404

AVPW 0.0818651

AVPY -0.321065

AVQA -0.851354

AVQC -0.0517998

AVQD -0.709413

AVQE -0.708532

AVQF -0.240588

AVQG -1.0706

AVQH -0.332582

AVQI -0.603122

AVQK -0.636037

AVQL -0.804122

AVQM -0.326548

AVQN -0.518756

AVQP -0.778844

AVQQ -0.298117

AVQR -0.486029

AVQS -0.650641

AVQT -0.613289

AVQV -0.59038

AVQW 0.0781283

AVQY -0.173505

AVRA -1.0198

AVRC -0.166593

AVRD -0.625794

AVRE -0.655437

AVRF -0.374039

AVRG -1.22687

AVRH -0.306504

AVRI -0.65177

AVRK -0.863858

AVRL -0.910339

AVRM -0.257852

AVRN -0.655742

AVRP -0.794799

AVRQ -0.46664

AVRR -0.57381

AVRS -0.755115

AVRT -0.734321

AVRV -0.811361

AVRW 0.0143611

AVRY -0.24162

AVSA -1.114

AVSC -0.191993

AVSD -0.906941

AVSE -0.966537

AVSF -0.564961

AVSG -1.22666

AVSH -0.409562

AVSI -0.70446

AVSK -0.92787

AVSL -1.12035

AVSM -0.477552

AVSN -0.726167

AVSP -0.98743

AVSQ -0.599052

AVSR -0.745432

AVSS -0.851403

AVST -0.812066

AVSV -0.386636

AVSW -0.125118

AVSY -0.435978

AVTA -1.09252

AVTC -0.103173

AVTD -0.831235

AVTE -0.871318

AVTF -0.433053

AVTG -1.22011

AVTH -0.346218

AVTI -0.726905

AVTK -0.85921

AVTL -1.14067

AVTM -0.338609

AVTN -0.66707

AVTP -0.853634

AVTQ -0.537188

AVTR -0.679941

AVTS -0.819874

AVTT -0.717368

AVTV -0.579769

AVTW -0.0337636

AVTY -0.366118

AVVA -0.81023

AVVC 0.00109673

AVVD -1.03195

AVVE -1.05442

AVVF -0.422735

AVVG -1.38153

AVVH -0.384781

AVVI -0.869887

AVVK -0.998438

AVVL -0.538719

AVVM -0.123638

AVVN -0.873985

AVVP -0.935912

AVVQ -0.67262

AVVR -0.816439

AVVS -1.03365

AVVT -0.892163

AVVV -0.111204

AVVW 0.0755305

AVVY -0.394543

AVWA -0.276819

AVWC 0.652617

AVWD -0.201214

AVWE -0.234192

AVWF 0.495717

AVWG -0.50088

AVWH 0.251571

AVWI 0.237706

AVWK -0.200318

AVWL 0.0378816

AVWM 0.449385

AVWN -0.0196753

AVWP -0.163884

AVWQ 0.0934482

AVWR 0.0689123

AVWS -0.197246

AVWT -0.114602

AVWV 0.0990176

AVWW 0.797094

AVWY 0.587192

AVYA -0.674326

AVYC 0.503975

AVYD -0.458344

AVYE -0.569389

AVYF 0.0457954

AVYG -0.817892

AVYH 0.0627043

AVYI -0.233104

AVYK -0.477546

AVYL -0.568851

AVYM 0.207969

AVYN -0.276498

AVYP -0.413999

AVYQ -0.203806

AVYR -0.237274

AVYS -0.511414

AVYT -0.403839

AVYV -0.369938

AVYW 0.541618

AVYY 0.130287

AWAA -0.576997

AWAC 0.217765

AWAD -0.497489

AWAE -0.495908

AWAF 0.207329

AWAG -0.936277

AWAH 0.0272949

AWAI -0.231483

AWAK -0.434119

AWAL -0.498859

AWAM 0.206331

AWAN -0.345369

AWAP -0.627869

AWAQ -0.210646

AWAR -0.245462

AWAS -0.493416

AWAT -0.386261

AWAV -0.367467

AWAW 4.02081

AWAY 0.111471

AWCA 0.274466

AWCC 2.3188

AWCD 0.08938

AWCE 0.0308444

AWCF 0.885926

AWCG -0.0280988

AWCH 0.764648

AWCI 0.610568

AWCK 0.246294

AWCL 0.551816

AWCM 0.753286

AWCN 0.305893

AWCP 0.183463

AWCQ 0.484187

AWCR 0.459745

AWCS 0.317165

AWCT 0.304887

AWCV 0.598963

AWCW 1.1418

AWCY 0.912592

AWDA -0.441987

AWDC 0.107073

AWDD -0.245472

AWDE -0.281546

AWDF 0.0622482

AWDG -0.605657

AWDH 0.240988

AWDI -0.212848

AWDK -0.0361102

AWDL -0.432328

AWDM 0.0156376

AWDN 0.0222957

AWDP -0.360863

AWDQ -0.0198262

AWDR 0.111887

AWDS -0.170763

AWDT -0.11776

AWDV -0.314626

AWDW 0.431479

AWDY 0.252215

AWEA -0.5167

AWEC 0.0453274

AWED -0.375313

AWEE -0.326892

AWEF -0.0157485

AWEG -0.857563

AWEH 0.151734

AWEI -0.255076

AWEK -0.079541

AWEL -0.477707

AWEM 0.0463688

AWEN -0.155407

AWEP -0.500135

AWEQ -0.0380228

AWER 0.0620289

AWES -0.3801

AWET -0.238983

AWEV -0.289401

AWEW 0.4359

AWEY 0.114507

AWFA -0.011826

AWFC 0.900024

AWFD 0.0955477

AWFE 0.0103259

AWFF 0.821469

AWFG -0.328384

AWFH 0.654043

AWFI 0.443987

AWFK 0.083307

AWFL 0.150803

AWFM 0.861052

AWFN 0.196773

AWFP 0.100073

AWFQ 0.327228

AWFR 0.260897

AWFS 0.125715

AWFT 0.208437

AWFV 0.273344

AWFW 1.08742

AWFY 0.737822

AWGA -0.92922

AWGC -0.148722

AWGD -0.578686

AWGE -0.713821

AWGF -0.241142

AWGG -1.75696

AWGH -0.330788

AWGI -0.538143

AWGK -0.5664

AWGL -0.740498

AWGM -0.271914

AWGN -0.455977

AWGP -0.811114

AWGQ -0.430732

AWGR -0.403382

AWGS -0.591594

AWGT -0.465902

AWGV -0.615262

AWGW 0.0612466

AWGY -0.141023

AWHA -0.0564206

AWHC 0.691268

AWHD 0.293193

AWHE 0.164742

AWHF 0.67248

AWHG -0.340869

AWHH 0.757378

AWHI 0.309433

AWHK 0.00944853

AWHL 0.221388

AWHM 0.569001

AWHN 0.233127

AWHP 0.0414493

AWHQ 0.225458

AWHR 0.306606

AWHS 0.184171

AWHT 0.256249

AWHV 0.239864

AWHW 0.973403

AWHY 0.711449

AWIA -0.226354

AWIC 0.699446

AWID -0.209223

AWIE -0.236861

AWIF 0.454686

AWIG -0.591168

AWIH 0.350698

AWII 0.244357

AWIK -0.158958

AWIL -0.122045

AWIM 0.665805

AWIN -0.0415387

AWIP -0.1697

AWIQ 0.0876207

AWIR 0.0158103

AWIS -0.15424

AWIT -0.00729632

AWIV 0.0471759

AWIW 0.810769

AWIY 0.515147

AWKA -0.481612

AWKC 0.178693

AWKD -0.0876153

AWKE -0.0818193

AWKF 0.0560873

AWKG -0.75774

AWKH -0.00826836

AWKI -0.166721

AWKK -0.219092

AWKL -0.424065

AWKM 0.0318708

AWKN -0.118021

AWKP -0.416258

AWKQ -0.00823736

AWKR -0.2074

AWKS -0.285858

AWKT -0.193045

AWKV -0.304333

AWKW 0.351237

AWKY 0.209254

AWLA -0.52914

AWLC 0.650588

AWLD -0.458554

AWLE -0.461839

AWLF 0.220334

AWLG -0.799511

AWLH 0.25033

AWLI -0.114016

AWLK -0.387749

AWLL -0.47385

AWLM 0.441579

AWLN -0.2884

AWLP -0.347941

AWLQ -0.0540454

AWLR -0.17817

AWLS -0.419743

AWLT -0.296278

AWLV -0.317735

AWLW 0.724036

AWLY 0.209992

AWMA 0.165705

AWMC 0.831354

AWMD 0.0784547

AWME 0.096205

AWMF 0.863061

AWMG -0.439033

AWMH 0.461146

AWMI 0.623808

AWMK 0.116853

AWML 0.439196

AWMM 0.896885

AWMN 0.12209

AWMP -0.0621665

AWMQ 0.276987

AWMR 0.308975

AWMS 0.0621583

AWMT 0.236514

AWMV 0.475286

AWMW 0.87307

AWMY 0.803491

AWNA -0.351174

AWNC 0.310395

AWND -0.0398533

AWNE -0.149822

AWNF 0.203452

AWNG -0.490656

AWNH 0.252102

AWNI -0.0867691

AWNK -0.0333028

AWNL -0.285043

AWNM 0.132991

AWNN 0.184577

AWNP -0.180246

AWNQ 0.144874

AWNR 0.0619891

AWNS -0.0591934

AWNT -0.00693822

AWNV -0.185719

AWNW 0.687135

AWNY 0.374505

AWPA -0.333881

AWPC 0.260104

AWPD -0.122502

AWPE -0.10456

AWPF 0.25038

AWPG -0.51556

AWPH 0.215685

AWPI -0.0651524

AWPK -0.176851

AWPL -0.238961

AWPM 0.198149

AWPN -0.0332234

AWPP -0.199215

AWPQ 0.0796225

AWPR 0.0375695

AWPS -0.110754

AWPT -0.0543871

AWPV -0.0939767

AWPW 0.682032

AWPY 0.342214

AWQA -0.18777

AWQC 0.467449

AWQD -0.0675447

AWQE -0.00044179

AWQF 0.6232

AWQG -0.455185

AWQH 0.213562

AWQI 0.0522838

AWQK 0.0176167

AWQL -0.0815094

AWQM 0.229336

AWQN 0.12022

AWQP -0.205106

AWQQ 0.334921

AWQR 0.163

AWQS 0.0190594

AWQT 0.0646465

AWQV -0.0484099

AWQW 0.600511

AWQY 0.434224

AWRA -0.311217

AWRC 0.374601

AWRD 0.0803404

AWRE 0.0652752

AWRF 0.282855

AWRG -0.583723

AWRH 0.290209

AWRI 0.028404

AWRK -0.199147

AWRL -0.188017

AWRM 0.338192

AWRN -0.00141025

AWRP -0.164174

AWRQ 0.172783

AWRR 0.105595

AWRS -0.0959873

AWRT -0.06846

AWRV -0.101005

AWRW 0.604935

AWRY 0.406052

AWSA -0.494608

AWSC 0.371134

AWSD -0.21497

AWSE -0.216261

AWSF 0.195724

AWSG -0.545439

AWSH 0.196061

AWSI -0.183601

AWSK -0.216912

AWSL -0.400969

AWSM 0.118113

AWSN -0.0432494

AWSP -0.354247

AWSQ 0.0808504

AWSR -0.0470386

AWSS -0.119247

AWST -0.0665202

AWSV -0.286871

AWSW 0.555269

AWSY 0.233392

AWTA -0.391807

AWTC 0.468394

AWTD -0.150625

AWTE -0.183221

AWTF 0.209424

AWTG -0.535929

AWTH 0.26045

AWTI -0.0322354

AWTK -0.166942

AWTL -0.301147

AWTM 0.272531

AWTN 0.0125058

AWTP -0.228955

AWTQ 0.120861

AWTR 0.00302124

AWTS -0.109612

AWTT -0.0160599

AWTV -0.133075

AWTW 0.864262

AWTY 0.322262

AWVA -0.382867

AWVC 0.63149

AWVD -0.33626

AWVE -0.359114

AWVF 0.293817

AWVG -0.705705

AWVH 0.25366

AWVI 0.0415316

AWVK -0.282685

AWVL -0.30746

AWVM 0.498467

AWVN -0.187879

AWVP -0.28052

AWVQ -0.0363131

AWVR -0.117593

AWVS -0.290342

AWVT -0.153184

AWVV -0.129558

AWVW 0.849683

AWVY 0.297999

AWWA 0.314224

AWWC 1.14468

AWWD 0.349785

AWWE 0.329395

AWWF 1.11157

AWWG 0.04761

AWWH 0.747917

AWWI 0.860879

AWWK 0.362405

AWWL 0.71145

AWWM 0.961971

AWWN 0.527602

AWWP 0.365586

AWWQ 0.629453

AWWR 0.639256

AWWS 0.36564

AWWT 0.452172

AWWV 0.744373

AWWW 1.30557

AWWY 1.19863

AWYA 0.006742

AWYC 1.10446

AWYD 0.226615

AWYE 0.0986776

AWYF 0.734184

AWYG -0.165932

AWYH 0.665636

AWYI 0.44398

AWYK 0.194529

AWYL 0.195614

AWYM 0.784812

AWYN 0.36414

AWYP 0.240186

AWYQ 0.453152

AWYR 0.404914

AWYS 0.172062

AWYT 0.281546

AWYV 0.279361

AWYW 1.15376

AWYY 0.831219

AYAA -0.882771

AYAC -0.0471265

AYAD -0.822608

AYAE -0.825945

AYAF 0.319002

AYAG -1.25533

AYAH -0.169517

AYAI -0.551113

AYAK -0.757195

AYAL -0.814373

AYAM -0.11917

AYAN -0.638264

AYAP -0.91348

AYAQ -0.50513

AYAR -0.602449

AYAS -0.806068

AYAT -0.705246

AYAV -0.748292

AYAW 0.134472

AYAY 4.67865

AYCA -0.00092864

AYCC 2.00464

AYCD -0.133116

AYCE -0.195446

AYCF 0.620493

AYCG -0.261637

AYCH 0.561201

AYCI 0.338715

AYCK 0.00677013

AYCL 0.235274

AYCM 0.555589

AYCN 0.087503

AYCP -0.0276592

AYCQ 0.26473

AYCR 0.222991

AYCS 0.067682

AYCT 0.0687659

AYCV 0.299217

AYCW 0.943275

AYCY 0.675149

AYDA -0.803254

AYDC -0.118323

AYDD -0.578735

AYDE -0.625459

AYDF -0.278569

AYDG -0.92648

AYDH -0.0390899

AYDI -0.556569

AYDK -0.387529

AYDL -0.797578

AYDM -0.234599

AYDN -0.314415

AYDP -0.651694

AYDQ -0.332718

AYDR -0.242626

AYDS -0.512896

AYDT -0.471952

AYDV -0.660633

AYDW 0.148753

AYDY -0.0345235

AYEA -0.860624

AYEC -0.183438

AYED -0.707921

AYEE -0.657144

AYEF -0.326112

AYEG -1.15665

AYEH -0.130509

AYEI -0.60377

AYEK -0.444858

AYEL -0.855616

AYEM -0.219611

AYEN -0.479734

AYEP -0.785047

AYEQ -0.355757

AYER -0.304782

AYES -0.712604

AYET -0.615449

AYEV -0.55793

AYEW 0.154085

AYEY -0.16322

AYFA -0.36048

AYFC 0.634945

AYFD -0.232624

AYFE -0.321352

AYFF 0.416752

AYFG -0.631425

AYFH 0.392437

AYFI 0.0821412

AYFK -0.245908

AYFL -0.241442

AYFM 0.556936

AYFN -0.123488

AYFP -0.203197

AYFQ 0.0167382

AYFR -0.0604577

AYFS -0.198009

AYFT 0.0257759

AYFV -0.108349

AYFW 0.797423

AYFY 0.411667

AYGA -1.24929

AYGC -0.372484

AYGD -0.905444

AYGE -1.02114

AYGF -0.595144

AYGG -1.97831

AYGH -0.565806

AYGI -0.866131

AYGK -0.893254

AYGL -1.09256

AYGM -0.519255

AYGN -0.741469

AYGP -1.08134

AYGQ -0.720259

AYGR -0.7146

AYGS -0.916246

AYGT -0.798252

AYGV -0.961503

AYGW -0.240071

AYGY -0.176061

AYHA -0.335799

AYHC 0.493357

AYHD 0.00912881

AYHE -0.115231

AYHF 0.365452

AYHG -0.576946

AYHH 0.517653

AYHI 0.0181475

AYHK -0.239762

AYHL -0.099365

AYHM 0.352534

AYHN -0.0154214

AYHP -0.194046

AYHQ -0.00669241

AYHR 0.0564287

AYHS -0.0809138

AYHT -0.024143

AYHV -0.0239058

AYHW 0.737025

AYHY 0.497132

AYIA -0.606711

AYIC 0.420384

AYID -0.548221

AYIE -0.580616

AYIF 0.0518215

AYIG -0.910446

AYIH 0.0721436

AYII -0.128308

AYIK -0.517258

AYIL -0.327888

AYIM 0.338192

AYIN -0.333523

AYIP -0.480376

AYIQ -0.229874

AYIR -0.167458

AYIS -0.490274

AYIT -0.365457

AYIV -0.326212

AYIW 0.523157

AYIY 0.067302

AYKA -0.84184

AYKC -0.0541649

AYKD -0.442487

AYKE -0.453436

AYKF -0.255816

AYKG -1.06267

AYKH -0.258486

AYKI -0.513857

AYKK -0.560133

AYKL -0.782138

AYKM -0.226712

AYKN -0.435682

AYKP -0.707697

AYKQ -0.317994

AYKR -0.527038

AYKS -0.617297

AYKT -0.536858

AYKV -0.659892

AYKW 0.068799

AYKY -0.100755

AYLA -0.926155

AYLC 0.325663

AYLD -0.811728

AYLE -0.832375

AYLF -0.231162

AYLG -1.14296

AYLH -0.0920165

AYLI -0.503168

AYLK -0.76557

AYLL -0.878993

AYLM 0.09322

AYLN -0.628232

AYLP -0.696218

AYLQ -0.412299

AYLR -0.566949

AYLS -0.7273

AYLT -0.674243

AYLV -0.733337

AYLW 0.371287

AYLY 0.0204701

AYMA -0.14029

AYMC 0.626875

AYMD -0.181324

AYME -0.178252

AYMF 0.550843

AYMG -0.667852

AYMH 0.256449

AYMI 0.35602

AYMK -0.146488

AYML 0.0762341

AYMM 0.654634

AYMN -0.10874

AYMP -0.283875

AYMQ 0.0428791

AYMR 0.0463088

AYMS -0.185183

AYMT -0.0554669

AYMV 0.154786

AYMW 0.659705

AYMY 0.503661

AYNA -0.682208

AYNC 0.0911613

AYND -0.360917

AYNE -0.46989

AYNF 0.0117962

AYNG -0.786956

AYNH 0.00524187

AYNI -0.399817

AYNK -0.368257

AYNL -0.623132

AYNM -0.103629

AYNN -0.130811

AYNP -0.461705

AYNQ -0.153938

AYNR -0.253445

AYNS -0.38397

AYNT -0.341538

AYNV -0.481031

AYNW 0.350347

AYNY 0.258202

AYPA -0.670289

AYPC 0.0426614

AYPD -0.44543

AYPE -0.437103

AYPF -0.0556755

AYPG -0.815662

AYPH -0.03004

AYPI -0.399782

AYPK -0.499417

AYPL -0.582193

AYPM -0.0473759

AYPN -0.330508

AYPP -0.482308

AYPQ -0.21111

AYPR -0.279304

AYPS -0.429411

AYPT -0.384017

AYPV -0.452729

AYPW 0.398435

AYPY 0.0804644

AYQA -0.508976

AYQC 0.250115

AYQD -0.369922

AYQE -0.357026

AYQF 0.0318515

AYQG -0.741889

AYQH -0.0224614

AYQI -0.241737

AYQK -0.291392

AYQL -0.433485

AYQM -0.00443125

AYQN -0.177293

AYQP -0.464455

AYQQ 0.0373468

AYQR -0.141176

AYQS -0.330063

AYQT -0.241833

AYQV -0.214252

AYQW 0.388772

AYQY 0.180581

AYRA -0.6523

AYRC 0.139519

AYRD -0.277206

AYRE -0.304834

AYRF -0.0262008

AYRG -0.88774

AYRH 0.0267041

AYRI -0.313062

AYRK -0.522525

AYRL -0.569856

AYRM 0.0756447

AYRN -0.304633

AYRP -0.459952

AYRQ -0.139598

AYRR -0.224285

AYRS -0.431397

AYRT -0.396808

AYRV -0.46089

AYRW 0.330076

AYRY 0.154099

AYSA -0.839993

AYSC 0.117864

AYSD -0.550683

AYSE -0.592209

AYSF -0.0982099

AYSG -0.876636

AYSH -0.0832155

AYSI -0.511992

AYSK -0.526448

AYSL -0.746203

AYSM -0.149229

AYSN -0.364999

AYSP -0.654514

AYSQ -0.24299

AYSR -0.376122

AYSS -0.480491

AYST -0.436362

AYSV -0.61766

AYSW 0.17923

AYSY 0.0939326

AYTA -0.731187

AYTC 0.215742

AYTD -0.481253

AYTE -0.525504

AYTF -0.104768

AYTG -0.862121

AYTH -0.00966763

AYTI -0.384392

AYTK -0.506478

AYTL -0.660524

AYTM -0.00801587

AYTN -0.313957

AYTP -0.532794

AYTQ -0.199205

AYTR -0.335889

AYTS -0.469472

AYTT -0.374829

AYTV -0.519624

AYTW 0.288898

AYTY 0.217462

AYVA -0.773288

AYVC 0.334313

AYVD -0.672209

AYVE -0.698339

AYVF -0.00619817

AYVG -1.03752

AYVH -0.0626168

AYVI -0.321371

AYVK -0.628448

AYVL -0.734936

AYVM 0.170522

AYVN -0.523222

AYVP -0.598885

AYVQ -0.37108

AYVR -0.48674

AYVS -0.495455

AYVT -0.546987

AYVV -0.500382

AYVW 0.415696

AYVY 0.383013

AYWA 0.0386178

AYWC 0.946996

AYWD 0.104719

AYWE 0.0831788

AYWF 0.824141

AYWG -0.18994

AYWH 0.546531

AYWI 0.563665

AYWK 0.115169

AYWL 0.38482

AYWM 0.74682

AYWN 0.291286

AYWP 0.141256

AYWQ 0.401096

AYWR 0.382493

AYWS 0.114204

AYWT 0.19964

AYWV 0.435885

AYWW 1.09882

AYWY 0.914718

AYYA -0.350579

AYYC 0.828687

AYYD -0.113317

AYYE -0.231443

AYYF 0.397659

AYYG -0.480549

AYYH 0.394299

AYYI 0.11273

AYYK -0.140963

AYYL -0.191115

AYYM 0.500849

AYYN 0.0583832

AYYP -0.0559869

AYYQ 0.147971

AYYR 0.0757856

AYYS -0.052341

AYYT -0.0392084

AYYV -0.0702198

AYYW 0.881139

AYYY 0.521742

CAAA -0.488211

CAAC 0.223536

CAAD -0.494729

CAAE -0.490958

CAAF 0.0373478

CAAG -0.927044

CAAH 0.0314991

CAAI -0.206213

CAAK -0.426499

CAAL -0.502747

CAAM 0.206469

CAAN -0.340693

CAAP -0.619709

CAAQ -0.189162

CAAR -0.259335

CAAS -0.475468

CAAT -0.384167

CAAV -0.377866

CAAW 0.380556

CAAY 0.0821605

CACA 3.78145

CACC 2.33483

CACD 0.0991161

CACE 0.0825355

CACF 1.04466

CACG -0.0169508

CACH 0.76566

CACI 0.62622

CACK 0.298318

CACL 0.638449

CACM 0.749861

CACN 0.308529

CACP 0.192595

CACQ 0.606109

CACR 0.47904

CACS 0.526176

CACT 0.46865

CACV 0.84902

CACW 1.14268

CACY 0.958917

CADA -0.421108

CADC 0.11132

CADD -0.224539

CADE -0.266817

CADF 0.0820522

CADG -0.588494

CADH 0.25108

CADI -0.194398

CADK -0.0149028

CADL -0.41744

CADM 0.0233853

CADN 0.0411952

CADP -0.345974

CADQ -0.00563693

CADR 0.132518

CADS -0.152531

CADT -0.0970674

CADV -0.294626

CADW 0.404649

CADY 0.266448

CAEA -0.487408

CAEC 0.0498989

CAED -0.358987

CAEE -0.299709

CAEF 0.0151753

CAEG -0.841295

CAEH 0.162435

CAEI -0.227084

CAEK -0.0561817

CAEL -0.437031

CAEM 0.0566308

CAEN -0.142701

CAEP -0.483943

CAEQ -0.0208828

CAER 0.078681

CAES -0.354096

CAET -0.25056

CAEV -0.350315

CAEW 0.423126

CAEY 0.137978

CAFA 0.0127192

CAFC 0.909519

CAFD 0.1113

CAFE 0.0202894

CAFF 0.765945

CAFG -0.31506

CAFH 0.666097

CAFI 0.468248

CAFK 0.0983355

CAFL 0.175712

CAFM 0.871592

CAFN 0.209586

CAFP 0.111284

CAFQ 0.338326

CAFR 0.27869

CAFS 0.144234

CAFT 0.22566

CAFV 0.297394

CAFW 1.08783

CAFY 0.753977

CAGA -0.915362

CAGC -0.144673

CAGD -0.566074

CAGE -0.70087

CAGF -0.232729

CAGG -1.75324

CAGH -0.324964

CAGI -0.520779

CAGK -0.547717

CAGL -0.716616

CAGM -0.270016

CAGN -0.442979

CAGP -0.801361

CAGQ -0.419585

CAGR -0.392956

CAGS -0.574825

CAGT -0.4479

CAGV -0.601983

CAGW -0.0117099

CAGY -0.126157

CAHA -0.0459101

CAHC 0.692921

CAHD 0.305038

CAHE 0.177253

CAHF 0.66427

CAHG -0.33321

CAHH 0.761824

CAHI 0.312188

CAHK 0.0183482

CAHL 0.246844

CAHM 0.572537

CAHN 0.241343

CAHP 0.0476429

CAHQ 0.23015

CAHR 0.315915

CAHS 0.195741

CAHT 0.268579

CAHV 0.251733

CAHW 0.952105

CAHY 0.722244

CAIA -0.195925

CAIC 0.71003

CAID -0.192399

CAIE -0.218307

CAIF 0.477343

CAIG -0.576379

CAIH 0.363094

CAII 0.264285

CAIK -0.143324

CAIL -0.0872815

CAIM 0.673375

CAIN -0.033555

CAIP -0.155092

CAIQ 0.0921769

CAIR 0.0437057

CAIS -0.137545

CAIT 0.0135214

CAIV 0.0566659

CAIW 0.830183

CAIY 0.518448

CAKA -0.459788

CAKC 0.184141

CAKD -0.0667827

CAKE -0.0629847

CAKF 0.0820653

CAKG -0.743935

CAKH 0.00019264

CAKI -0.159303

CAKK -0.200535

CAKL -0.3863

CAKM 0.0378489

CAKN -0.102783

CAKP -0.406593

CAKQ 0.00477743

CAKR -0.187314

CAKS -0.268357

CAKT -0.175829

CAKV -0.278223

CAKW 0.318199

CAKY 0.222845

CALA -0.496427

CALC 0.662672

CALD -0.437755

CALE -0.437903

CALF 0.184393

CALG -0.783079

CALH 0.250742

CALI -0.104854

CALK -0.369466

CALL -0.446926

CALM 0.459449

CALN -0.269746

CALP -0.337735

CALQ -0.0408528

CALR -0.158931

CALS -0.392565

CALT -0.282213

CALV -0.290735

CALW 0.718568

CALY 0.230233

CAMA 0.190058

CAMC 0.833349

CAMD 0.0849254

CAME 0.100514

CAMF 0.871584

CAMG -0.434385

CAMH 0.463712

CAMI 0.638266

CAMK 0.125062

CAML 0.45734

CAMM 0.902736

CAMN 0.12758

CAMP -0.0584071

CAMQ 0.281784

CAMR 0.31918

CAMS 0.071615

CAMT 0.245097

CAMV 0.490931

CAMW 0.867493

CAMY 0.814039

CANA -0.330915

CANC 0.313913

CAND -0.0186574

CANE -0.132131

CANF 0.221978

CANG -0.477637

CANH 0.259816

CANI -0.0696135

CANK -0.024435

CANL -0.254854

CANM 0.138158

CANN 0.199488

CANP -0.169444

CANQ 0.156358

CANR 0.0780156

CANS -0.0445731

CANT 0.0152805

CANV -0.171762

CANW 0.599759

CANY 0.396084

CAPA -0.317011

CAPC 0.263766

CAPD -0.107775

CAPE -0.0858641

CAPF 0.26675

CAPG -0.501829

CAPH 0.223604

CAPI -0.0511458

CAPK -0.162072

CAPL -0.216844

CAPM 0.204948

CAPN -0.0218251

CAPP -0.187817

CAPQ 0.0910366

CAPR 0.0512915

CAPS -0.0947843

CAPT -0.0376158

CAPV -0.0707757

CAPW 0.65483

CAPY 0.359317

CAQA -0.170365

CAQC 0.471004

CAQD -0.0522573

CAQE -0.0336592

CAQF 0.376928

CAQG -0.445176

CAQH 0.217894

CAQI 0.0617962

CAQK 0.0363932

CAQL -0.0646451

CAQM 0.235597

CAQN 0.128828

CAQP -0.193265

CAQQ 0.349837

CAQR 0.175984

CAQS 0.00214601

CAQT 0.0827382

CAQV -0.0269632

CAQW 0.619112

CAQY 0.481364

CARA -0.292544

CARC 0.377465

CARD 0.0966635

CARE 0.0797024

CARF 0.316451

CARG -0.576964

CARH 0.296225

CARI 0.046284

CARK -0.190283

CARL -0.175747

CARM 0.345862

CARN 0.0133021

CARP -0.153089

CARQ 0.184826

CARR 0.120974

CARS -0.0792561

CART -0.0471473

CARV -0.0833108

CARW 0.587778

CARY 0.418965

CASA -0.458302

CASC 0.378819

CASD -0.180712

CASE -0.233199

CASF 0.147603

CASG -0.52754

CASH 0.211018

CASI -0.17166

CASK -0.19717

CASL -0.36824

CASM 0.122661

CASN -0.0226762

CASP -0.343503

CASQ 0.0774047

CASR -0.0287185

CASS -0.0949435

CAST -0.064404

CASV -0.272393

CASW 0.443901

CASY 0.246148

CATA -0.350566

CATC 0.475903

CATD -0.134714

CATE -0.156443

CATF 0.253676

CATG -0.52275

CATH 0.268694

CATI -0.014725

CATK -0.147898

CATL -0.275469

CATM 0.283542

CATN 0.02546

CATP -0.212797

CATQ 0.140557

CATR 0.0216818

CATS -0.0900397

CATT 0.00749111

CATV -0.116279

CATW 0.560994

CATY 0.347214

CAVA -0.292159

CAVC 0.650166

CAVD -0.318365

CAVE -0.341775

CAVF 0.310476

CAVG -0.687081

CAVH 0.255861

CAVI 0.0604401

CAVK -0.259312

CAVL -0.296217

CAVM 0.515421

CAVN -0.178856

CAVP -0.264839

CAVQ -0.0197182

CAVR -0.100536

CAVS -0.272001

CAVT -0.138782

CAVV -0.101251

CAVW 0.721177

CAVY 0.325258

CAWA 0.326922

CAWC 1.1463

CAWD 0.356767

CAWE 0.33708

CAWF 1.12003

CAWG 0.0532701

CAWH 0.749724

CAWI 0.872816

CAWK 0.368306

CAWL 0.730191

CAWM 0.961921

CAWN 0.532911

CAWP 0.369138

CAWQ 0.634261

CAWR 0.646413

CAWS 0.37271

CAWT 0.459068

CAWV 0.755369

CAWW 1.3057

CAWY 1.20931

CAYA 0.035862

CAYC 1.11387

CAYD 0.234914

CAYE 0.112264

CAYF 0.772887

CAYG -0.15386

CAYH 0.674934

CAYI 0.4603

CAYK 0.213894

CAYL 0.204067

CAYM 0.788535

CAYN 0.371662

CAYP 0.254755

CAYQ 0.465774

CAYR 0.418569

CAYS 0.188139

CAYT 0.29667

CAYV 0.29809

CAYW 1.16338

CAYY 0.846864

CCAA -0.688926

CCAC 0.14856

CCAD -0.610025

CCAE -0.612341

CCAF -0.085639

CCAG -1.03052

CCAH -0.058984

CCAI -0.338737

CCAK -0.542539

CCAL -0.64534

CCAM 0.110173

CCAN -0.451498

CCAP -0.711313

CCAQ -0.297986

CCAR -0.380108

CCAS -0.593955

CCAT -0.497063

CCAV -0.500642

CCAW 0.298103

CCAY -0.0390596

CCCA 0.20804

CCCC 6.51786

CCCD 0.0446908

CCCE -0.0157993

CCCF 0.820224

CCCG -0.0786674

CCCH 0.728672

CCCI 0.543563

CCCK 0.195713

CCCL 0.461401

CCCM 0.718273

CCCN 0.262403

CCCP 0.143601

CCCQ 0.439166

CCCR 0.405647

CCCS 0.266406

CCCT 0.254018

CCCV 0.522281

CCCW 1.10609

CCCY 0.85238

CCDA -0.547501

CCDC 0.0626609

CCDD -0.340699

CCDE -0.381317

CCDF -0.0253301

CCDG -0.695049

CCDH 0.168676

CCDI -0.305997

CCDK -0.145251

CCDL -0.544737

CCDM -0.0425146

CCDN -0.0717733

CCDP -0.436721

CCDQ -0.104515

CCDR 0.00748301

CCDS -0.269391

CCDT -0.214942

CCDV -0.415506

CCDW 0.339982

CCDY 0.153214

CCEA -0.618786

CCEC -0.00118518

CCED -0.46981

CCEE -0.418031

CCEF -0.0944295

CCEG -0.937251

CCEH 0.0794442

CCEI -0.344566

CCEK -0.18688

CCEL -0.571615

CCEM -0.0157969

CCEN -0.249031

CCEP -0.570182

CCEQ -0.124354

CCER -0.0474029

CCES -0.468434

CCET -0.365235

CCEV -0.47068

CCEW 0.35227

CCEY 0.0273623

CCFA -0.117514

CCFC 0.837387

CCFD 0.00223541

CCFE -0.0894313

CCFF 0.640527

CCFG -0.412452

CCFH 0.58227

CCFI 0.337703

CCFK -0.010236

CCFL 0.0399036

CCFM 0.777345

CCFN 0.11198

CCFP 0.0176415

CCFQ 0.243264

CCFR 0.171038

CCFS 0.0316801

CCFT 0.110433

CCFV 0.161623

CCFW 1.00787

CCFY 0.633384

CCGA -1.02234

CCGC -0.193424

CCGD -0.671834

CCGE -0.803403

CCGF -0.334547

CCGG -1.80216

CCGH -0.386754

CCGI -0.627463

CCGK -0.658854

CCGL -0.839649

CCGM -0.329508

CCGN -0.537836

CCGP -0.876227

CCGQ -0.50576

CCGR -0.497393

CCGS -0.68135

CCGT -0.557422

CCGV -0.72143

CCGW -0.0663364

CCGY -0.229509

CCHA -0.127938

CCHC 0.657323

CCHD 0.218821

CCHE 0.0930727

CCHF 0.580662

CCHG -0.394807

CCHH 0.704752

CCHI 0.228983

CCHK -0.050838

CCHL 0.139183

CCHM 0.526886

CCHN 0.174623

CCHP -0.00863385

CCHQ 0.175133

CCHR 0.242033

CCHS 0.115699

CCHT 0.185233

CCHV 0.158132

CCHW 0.907683

CCHY 0.641109

CCIA -0.336236

CCIC 0.627946

CCID -0.308025

CCIE -0.333592

CCIF 0.349438

CCIG -0.680746

CCIH 0.276066

CCII 0.123452

CCIK -0.260496

CCIL -0.229983

CCIM 0.569814

CCIN -0.139284

CCIP -0.254006

CCIQ -0.00815344

CCIR -0.0704234

CCIS -0.252458

CCIT -0.110754

CCIV -0.0780754

CCIW 0.741767

CCIY 0.39132

CCKA -0.58453

CCKC 0.132112

CCKD -0.191067

CCKE -0.194216

CCKF -0.0259297

CCKG -0.839372

CCKH -0.0684218

CCKI -0.275624

CCKK -0.317469

CCKL -0.519394

CCKM -0.0291638

CCKN -0.20709

CCKP -0.492367

CCKQ -0.0943923

CCKR -0.295784

CCKS -0.378998

CCKT -0.290094

CCKV -0.400973

CCKW 0.257925

CCKY 0.111436

CCLA -0.651585

CCLC 0.559645

CCLD -0.564538

CCLE -0.57117

CCLF 0.0440202

CCLG -0.899985

CCLH 0.141477

CCLI -0.242131

CCLK -0.499438

CCLL -0.594076

CCLM 0.342168

CCLN -0.388591

CCLP -0.450415

CCLQ -0.160739

CCLR -0.29256

CCLS -0.526194

CCLT -0.412204

CCLV -0.432902

CCLW 0.610791

CCLY 0.094975

CCMA 0.0835907

CCMC 0.79276

CCMD 0.0161021

CCME 0.0243008

CCMF 0.77836

CCMG -0.487736

CCMH 0.422291

CCMI 0.538149

CCMK 0.0537572

CCML 0.338349

CCMM 0.844121

CCMN 0.072052

CCMP -0.106918

CCMQ 0.226971

CCMR 0.244971

CCMS 0.00298762

CCMT 0.169452

CCMV 0.384962

CCMW 0.827229

CCMY 0.730321

CCNA -0.442747

CCNC 0.269743

CCND -0.126682

CCNE -0.239677

CCNF 0.122284

CCNG -0.571535

CCNH 0.193072

CCNI -0.172661

CCNK -0.134314

CCNL -0.37517

CCNM 0.0812984

CCNN 0.095274

CCNP -0.251824

CCNQ 0.0661607

CCNR -0.0240037

CCNS -0.151212

CCNT -0.096566

CCNV -0.281436

CCNW 0.539357

CCNY 0.292287

CCPA -0.434984

CCPC 0.218928

CCPD -0.212455

CCPE -0.19842

CCPF 0.163812

CCPG -0.596498

CCPH 0.157498

CCPI -0.158227

CCPK -0.264385

CCPL -0.339571

CCPM 0.143165

CCPN -0.113814

CCPP -0.272827

CCPQ 0.00286984

CCPR -0.0516186

CCPS -0.200407

CCPT -0.145933

CCPV -0.193073

CCPW 0.589952

CCPY 0.25698

CCQA -0.28057

CCQC 0.423879

CCQD -0.146764

CCQE -0.135876

CCQF 0.279326

CCQG -0.529823

CCQH 0.162896

CCQI -0.0382032

CCQK -0.0654249

CCQL -0.185332

CCQM 0.181716

CCQN 0.0396547

CCQP -0.26401

CCQQ 0.257065

CCQR 0.0782583

CCQS -0.0975373

CCQT -0.018585

CCQV -0.135554

CCQW 0.566947

CCQY 0.38319

CCRA -0.411417

CCRC 0.323509

CCRD -0.0259476

CCRE -0.0460987

CCRF 0.206123

CCRG -0.669606

CCRH 0.222316

CCRI -0.0698454

CCRK -0.293664

CCRL -0.306625

CCRM 0.27268

CCRN -0.0848086

CCRP -0.24235

CCRQ 0.085757

CCRR 0.0107684

CCRS -0.191915

CCRT -0.158784

CCRV -0.205459

CCRW 0.523262

CCRY 0.308034

CCSA -0.594266

CCSC 0.314071

CCSD -0.300934

CCSE -0.352477

CCSF 0.0360217

CCSG -0.637664

CCSH 0.127949

CCSI -0.284262

CCSK -0.314728

CCSL -0.49942

CCSM 0.0509975

CCSN -0.134444

CCSP -0.436395

CCSQ -0.0251353

CCSR -0.143748

CCSS -0.216797

CCST -0.184137

CCSV -0.397455

CCSW 0.37625

CCSY 0.133713

CCTA -0.488538

CCTC 0.408478

CCTD -0.250793

CCTE -0.275625

CCTF 0.138253

CCTG -0.626872

CCTH 0.188377

CCTI -0.132751

CCTK -0.265744

CCTL -0.404799

CCTM 0.20153

CCTN -0.0841157

CCTP -0.309575

CCTQ 0.0347264

CCTR -0.0906959

CCTS -0.211035

CCTT -0.113357

CCTV -0.253659

CCTW 0.489714

CCTY 0.2311

CCVA -0.496294

CCVC 0.570871

CCVD -0.438788

CCVE -0.465166

CCVF 0.17566

CCVG -0.801117

CCVH 0.163653

CCVI -0.0866885

CCVK -0.386287

CCVL -0.436093

CCVM 0.406521

CCVN -0.283179

CCVP -0.371192

CCVQ -0.128757

CCVR -0.223004

CCVS -0.398018

CCVT -0.263159

CCVV -0.253054

CCVW 0.625295

CCVY 0.197281

CCWA 0.244801

CCWC 1.11011

CCWD 0.295007

CCWE 0.271395

CCWF 1.03605

CCWG -0.00464749

CCWH 0.712447

CCWI 0.782204

CCWK 0.304869

CCWL 0.619808

CCWM 0.918608

CCWN 0.476016

CCWP 0.319254

CCWQ 0.581272

CCWR 0.579014

CCWS 0.309338

CCWT 0.393806

CCWV 0.657698

CCWW 1.26475

CCWY 1.12582

CCYA -0.0936098

CCYC 1.03448

CCYD 0.123789

CCYE 0.00516891

CCYF 0.655126

CCYG -0.255152

CCYH 0.596362

CCYI 0.338686

CCYK 0.101782

CCYL 0.0635896

CCYM 0.706794

CCYN 0.270571

CCYP 0.158732

CCYQ 0.368994

CCYR 0.308378

CCYS 0.0822825

CCYT 0.183413

CCYV 0.174058

CCYW 1.08039

CCYY 0.725405

CDAA -0.472875

CDAC 0.226444

CDAD -0.403634

CDAE -0.419103

CDAF 0.116575

CDAG -0.882747

CDAH 0.0564935

CDAI -0.125045

CDAK -0.350402

CDAL -0.407736

CDAM 0.2427

CDAN -0.288083

CDAP -0.590301

CDAQ -0.142697

CDAR -0.193845

CDAS -0.411806

CDAT -0.30894

CDAV -0.27819

CDAW 0.39679

CDAY 0.151294

CDCA 0.300436

CDCC 2.3728

CDCD 2.19836

CDCE 0.0427535

CDCF 0.889694

CDCG -0.0395029

CDCH 0.734977

CDCI 0.631155

CDCK 0.248104

CDCL 0.602882

CDCM 0.721712

CDCN 0.303908

CDCP 0.158905

CDCQ 0.462306

CDCR 0.45801

CDCS 0.329691

CDCT 0.302854

CDCV 0.634696

CDCW 1.10876

CDCY 0.918842

CDDA -0.360242

CDDC 0.0907185

CDDD -0.166104

CDDE -0.207358

CDDF 0.130403

CDDG -0.547436

CDDH 0.266939

CDDI -0.139989

CDDK 0.0602329

CDDL -0.34465

CDDM 0.0180538

CDDN 0.0991213

CDDP -0.325551

CDDQ 0.0289552

CDDR 0.201866

CDDS -0.0883288

CDDT -0.033515

CDDV -0.229926

CDDW 0.39864

CDDY 0.321132

CDEA -0.427068

CDEC 0.0306809

CDED -0.307105

CDEE -0.23634

CDEF 0.0649524

CDEG -0.813863

CDEH 0.178128

CDEI -0.169087

CDEK 0.0233748

CDEL -0.36097

CDEM 0.0611732

CDEN -0.0918109

CDEP -0.464543

CDEQ 0.0206225

CDER 0.170372

CDES -0.304246

CDET -0.193482

CDEV -0.282576

CDEW 0.425087

CDEY 0.189989

CDFA 0.0768323

CDFC 0.913152

CDFD 0.178909

CDFE 0.0702438

CDFF 0.841393

CDFG -0.282473

CDFH 0.684402

CDFI 0.546107

CDFK 0.151891

CDFL 0.268086

CDFM 0.90092

CDFN 0.243675

CDFP 0.140193

CDFQ 0.36915

CDFR 0.327089

CDFS 0.197863

CDFT 0.280774

CDFV 0.375032

CDFW 1.103

CDFY 0.816003

CDGA -0.875465

CDGC -0.166325

CDGD -0.516104

CDGE -0.655164

CDGF -0.192175

CDGG -1.77529

CDGH -0.332998

CDGI -0.476042

CDGK -0.501701

CDGL -0.649657

CDGM -0.278643

CDGN -0.409502

CDGP -0.79588

CDGQ -0.397784

CDGR -0.346902

CDGS -0.528623

CDGT -0.396284

CDGV -0.547304

CDGW -0.0283973

CDGY -0.0863357

CDHA -0.02685

CDHC 0.659752

CDHD 0.328241

CDHE 0.193619

CDHF 0.682021

CDHG -0.342427

CDHH 0.74806

CDHI 0.329625

CDHK 0.0179224

CDHL 0.296053

CDHM 0.547086

CDHN 0.236168

CDHP 0.0329235

CDHQ 0.215223

CDHR 0.32353

CDHS 0.208542

CDHT 0.279763

CDHV 0.282849

CDHW 0.926199

CDHY 0.736356

CDIA -0.122642

CDIC 0.724521

CDID -0.134634

CDIE -0.148333

CDIF 0.558645

CDIG -0.538266

CDIH 0.383885

CDII 0.353699

CDIK -0.0847182

CDIL 0.00950933

CDIM 0.715548

CDIN 0.0064044

CDIP -0.1204

CDIQ 0.133121

CDIR 0.102843

CDIS -0.0755222

CDIT 0.0807359

CDIV 0.151478

CDIW 0.851458

CDIY 0.59154

CDKA -0.402025

CDKC 0.167951

CDKD 0.0239322

CDKE 0.0141909

CDKF 0.133278

CDKG -0.711887

CDKH -0.00206995

CDKI -0.102128

CDKK -0.142137

CDKL -0.313315

CDKM 0.0348711

CDKN -0.0612411

CDKP -0.386707

CDKQ 0.0445466

CDKR -0.149314

CDKS -0.210013

CDKT -0.11855

CDKV -0.210847

CDKW 0.307676

CDKY 0.274677

CDLA -0.417193

CDLC 0.71225

CDLD -0.34955

CDLE -0.354963

CDLF 0.277148

CDLG -0.72072

CDLH 0.297493

CDLI -0.00223279

CDLK -0.290318

CDLL -0.334668

CDLM 0.522777

CDLN -0.206975

CDLP -0.276535

CDLQ 0.0262935

CDLR -0.0818982

CDLS -0.318589

CDLT -0.19827

CDLV -0.18993

CDLW 0.770905

CDLY 0.316955

CDMA 0.211669

CDMC 0.802934

CDMD 0.0856607

CDME 0.106432

CDMF 0.902103

CDMG -0.451853

CDMH 0.434743

CDMI 0.67902

CDMK 0.129556

CDML 0.519559

CDMM 0.890959

CDMN 0.112903

CDMP -0.0810664

CDMQ 0.266965

CDMR 0.319968

CDMS 0.0700691

CDMT 0.253814

CDMV 0.537823

CDMW 0.836263

CDMY 0.830988

CDNA -0.282233

CDNC 0.290027

CDND 0.0420272

CDNE -0.0830925

CDNF 0.257036

CDNG -0.448409

CDNH 0.256249

CDNI -0.0300913

CDNK 0.0244622

CDNL -0.192669

CDNM 0.123734

CDNN 0.252656

CDNP -0.153476

CDNQ 0.18505

CDNR 0.120477

CDNS 0.00589967

CDNT 0.0614059

CDNV -0.11933

CDNW 0.588944

CDNY 0.437959

CDPA -0.269168

CDPC 0.238966

CDPD -0.0593982

CDPE -0.0331492

CDPF 0.309245

CDPG -0.472859

CDPH 0.219249

CDPI -0.00839543

CDPK -0.122817

CDPL -0.146229

CDPM 0.196166

CDPN 0.00809503

CDPP -0.173382

CDPQ 0.114161

CDPR 0.0922656

CDPS -0.0477195

CDPT 0.00811481

CDPV -0.0116532

CDPW 0.649019

CDPY 0.400799

CDQA -0.122001

CDQC 0.446177

CDQD -0.0225976

CDQE 0.0130913

CDQF 0.411252

CDQG -0.426977

CDQH 0.201524

CDQI 0.0987

CDQK 0.0769796

CDQL 0.00013328

CDQM 0.216835

CDQN 0.153138

CDQP -0.192028

CDQQ 0.377201

CDQR 0.210287

CDQS 0.0393031

CDQT 0.122059

CDQV 0.022119

CDQW 0.600003

CDQY 0.515932

CDRA -0.228255

CDRC 0.360662

CDRD 0.161981

CDRE 0.156182

CDRF 0.369009

CDRG -0.54833

CDRH 0.300932

CDRI 0.104136

CDRK -0.147097

CDRL -0.0987377

CDRM 0.350843

CDRN 0.0551546

CDRP -0.129357

CDRQ 0.221308

CDRR 0.174425

CDRS -0.0292192

CDRT 0.00113916

CDRV -0.0174186

CDRW 0.581432

CDRY 0.470513

CDSA -0.409825

CDSC 0.373403

CDSD -0.107947

CDSE -0.172505

CDSF 0.204114

CDSG -0.478341

CDSH 0.222695

CDSI -0.112835

CDSK -0.138313

CDSL -0.289942

CDSM 0.126284

CDSN 0.0224688

CDSP -0.321777

CDSQ 0.124073

CDSR 0.0267

CDSS -0.0261254

CDST 0.00538063

CDSV -0.207695

CDSW 0.441702

CDSY 0.301021

CDTA -0.300403

CDTC 0.473467

CDTD -0.0561256

CDTE -0.0882149

CDTF 0.312135

CDTG -0.474567

CDTH 0.28254

CDTI 0.0545352

CDTK -0.0837471

CDTL -0.190038

CDTM 0.289264

CDTN 0.0729339

CDTP -0.18419

CDTQ 0.182927

CDTR 0.085187

CDTS -0.0264482

CDTT 0.0762296

CDTV -0.0481164

CDTW 0.563412

CDTY 0.405058

CDVA -0.266592

CDVC 0.671292

CDVD -0.247944

CDVE -0.269616

CDVF 0.393318

CDVG -0.642185

CDVH 0.288383

CDVI 0.145569

CDVK -0.19448

CDVL -0.189023

CDVM 0.560827

CDVN -0.122257

CDVP -0.216582

CDVQ 0.0314999

CDVR -0.0376709

CDVS -0.201677

CDVT -0.0611241

CDVV -0.0120802

CDVW 0.752291

CDVY 0.401462

CDWA 0.335836

CDWC 1.113

CDWD 0.349415

CDWE 0.33294

CDWF 1.13772

CDWG 0.0406282

CDWH 0.717338

CDWI 0.898183

CDWK 0.360587

CDWL 0.78102

CDWM 0.934314

CDWN 0.518115

CDWP 0.347601

CDWQ 0.616189

CDWR 0.644193

CDWS 0.36609

CDWT 0.453995

CDWV 0.78942

CDWW 1.27617

CDWY 1.22569

CDYA 0.0873008

CDYC 1.12518

CDYD 0.293189

CDYE 0.170061

CDYF 0.840854

CDYG -0.116457

CDYH 0.684746

CDYI 0.528344

CDYK 0.263427

CDYL 0.282562

CDYM 0.80364

CDYN 0.413054

CDYP 0.287069

CDYQ 0.499262

CDYR 0.470556

CDYS 0.24363

CDYT 0.349044

CDYV 0.375326

CDYW 1.17509

CDYY 0.906503

CEAA -0.473943

CEAC 0.227782

CEAD -0.438826

CEAE -0.377014

CEAF 0.108067

CEAG -0.882697

CEAH 0.0563109

CEAI -0.124322

CEAK -0.3528

CEAL -0.413034

CEAM 0.242525

CEAN -0.295088

CEAP -0.589501

CEAQ -0.140362

CEAR -0.186288

CEAS -0.405272

CEAT -0.311172

CEAV -0.277592

CEAW 0.397094

CEAY 0.147804

CECA 0.342695

CECC 2.37115

CECD 0.101593

CECE 2.2482

CECF 0.902529

CECG -0.0371425

CECH 0.736391

CECI 0.634152

CECK 0.261572

CECL 0.601937

CECM 0.726317

CECN 0.291224

CECP 0.162031

CECQ 0.49612

CECR 0.455887

CECS 0.321187

CECT 0.309557

CECV 0.620522

CECW 1.11158

CECY 0.920864

CEDA -0.363595

CEDC 0.0929954

CEDD -0.176762

CEDE -0.205543

CEDF 0.128741

CEDG -0.54873

CEDH 0.268038

CEDI -0.141546

CEDK 0.0530827

CEDL -0.346952

CEDM 0.0208251

CEDN 0.0931647

CEDP -0.323709

CEDQ 0.0275772

CEDR 0.198447

CEDS -0.0896473

CEDT -0.0349727

CEDV -0.232715

CEDW 0.400223

CEDY 0.319894

CEEA -0.430479

CEEC 0.0329239

CEED -0.310345

CEEE -0.223352

CEEF 0.0631547

CEEG -0.814198

CEEH 0.178386

CEEI -0.172625

CEEK 0.0319951

CEEL -0.361022

CEEM 0.0625985

CEEN -0.0978897

CEEP -0.464661

CEEQ 0.0235031

CEER 0.146849

CEES -0.303992

CEET -0.19596

CEEV -0.285906

CEEW 0.426119

CEEY 0.188013

CEFA 0.0749559

CEFC 0.913896

CEFD 0.162168

CEFE 0.085681

CEFF 0.8379

CEFG -0.28232

CEFH 0.683758

CEFI 0.541604

CEFK 0.148673

CEFL 0.263148

CEFM 0.903514

CEFN 0.248225

CEFP 0.139242

CEFQ 0.368566

CEFR 0.327341

CEFS 0.195383

CEFT 0.279793

CEFV 0.376579

CEFW 1.10345

CEFY 0.816039

CEGA -0.875285

CEGC -0.164031

CEGD -0.517328

CEGE -0.653558

CEGF -0.193375

CEGG -1.77295

CEGH -0.33162

CEGI -0.476642

CEGK -0.504968

CEGL -0.65296

CEGM -0.276561

CEGN -0.41033

CEGP -0.79499

CEGQ -0.398656

CEGR -0.349684

CEGS -0.529897

CEGT -0.398251

CEGV -0.549894

CEGW -0.0262959

CEGY -0.0868187

CEHA -0.0267203

CEHC 0.662523

CEHD 0.324485

CEHE 0.19561

CEHF 0.682412

CEHG -0.340662

CEHH 0.750073

CEHI 0.329815

CEHK 0.0191169

CEHL 0.293735

CEHM 0.549565

CEHN 0.236385

CEHP 0.0349205

CEHQ 0.216972

CEHR 0.324015

CEHS 0.208961

CEHT 0.280038

CEHV 0.282336

CEHW 0.92871

CEHY 0.736756

CEIA -0.126418

CEIC 0.724991

CEID -0.144878

CEIE -0.136364

CEIF 0.554848

CEIG -0.539848

CEIH 0.382905

CEII 0.348998

CEIK -0.0886865

CEIL 0.00625443

CEIM 0.714031

CEIN 0.0109539

CEIP -0.121411

CEIQ 0.131526

CEIR 0.0999587

CEIS -0.0774791

CEIT 0.0804513

CEIV 0.146617

CEIW 0.851436

CEIY 0.588101

CEKA -0.404246

CEKC 0.16974

CEKD 0.00171018

CEKE 0.0124395

CEKF 0.129326

CEKG -0.713715

CEKH -0.00015116

CEKI -0.104837

CEKK -0.143924

CEKL -0.316605

CEKM 0.036046

CEKN -0.0612679

CEKP -0.386687

CEKQ 0.0413027

CEKR -0.150962

CEKS -0.217234

CEKT -0.121641

CEKV -0.213354

CEKW 0.309507

CEKY 0.273047

CELA -0.417795

CELC 0.710806

CELD -0.368771

CELE -0.347249

CELF 0.274089

CELG -0.723496

CELH 0.295212

CELI -0.00356126

CELK -0.288193

CELL -0.344234

CELM 0.519255

CELN -0.21446

CELP -0.280681

CELQ 0.0353858

CELR -0.0879383

CELS -0.323596

CELT -0.201939

CELV -0.196162

CELW 0.769374

CELY 0.315124

CEMA 0.210305

CEMC 0.80563

CEMD 0.0854223

CEME 0.123168

CEMF 0.901532

CEMG -0.449876

CEMH 0.437196

CEMI 0.677717

CEMK 0.128859

CEML 0.517094

CEMM 0.895103

CEMN 0.117742

CEMP -0.0785882

CEMQ 0.268301

CEMR 0.320543

CEMS 0.0722892

CEMT 0.254308

CEMV 0.536052

CEMW 0.838948

CEMY 0.831114

CENA -0.288121

CENC 0.292763

CEND 0.0275185

CENE -0.0825164

CENF 0.25602

CENG -0.448099

CENH 0.257403

CENI -0.0312657

CENK 0.0230498

CENL -0.195853

CENM 0.125699

CENN 0.243282

CENP -0.153082

CENQ 0.187222

CENR 0.12273

CENS 0.0069468

CENT 0.0571482

CENV -0.120943

CENW 0.590864

CENY 0.437631

CEPA -0.274646

CEPC 0.241476

CEPD -0.0661159

CEPE -0.0329466

CEPF 0.308099

CEPG -0.473417

CEPH 0.22076

CEPI -0.00989127

CEPK -0.124585

CEPL -0.149521

CEPM 0.197869

CEPN 0.00565648

CEPP -0.173053

CEPQ 0.11374

CEPR 0.0909991

CEPS -0.0486255

CEPT 0.0047617

CEPV -0.0133789

CEPW 0.650615

CEPY 0.39964

CEQA -0.123936

CEQC 0.448655

CEQD -0.0159051

CEQE 0.0070169

CEQF 0.410799

CEQG -0.426808

CEQH 0.203559

CEQI 0.0980706

CEQK 0.0768261

CEQL -0.00306439

CEQM 0.219571

CEQN 0.152152

CEQP -0.190917

CEQQ 0.377284

CEQR 0.20953

CEQS 0.0382373

CEQT 0.12096

CEQV 0.0200686

CEQW 0.602247

CEQY 0.51527

CERA -0.225312

CERC 0.362774

CERD 0.162225

CERE 0.158152

CERF 0.36696

CERG -0.548295

CERH 0.301919

CERI 0.107243

CERK -0.14853

CERL -0.0952005

CERM 0.351453

CERN 0.0480049

CERP -0.129594

CERQ 0.218345

CERR 0.17186

CERS -0.0281134

CERT 0.00382137

CERV -0.0192053

CERW 0.583033

CERY 0.46872

CESA -0.4163

CESC 0.375582

CESD -0.122787

CESE -0.138311

CESF 0.202015

CESG -0.479485

CESH 0.222488

CESI -0.114371

CESK -0.136125

CESL -0.295897

CESM 0.129544

CESN 0.0217388

CESP -0.322292

CESQ 0.124175

CESR 0.0255351

CESS -0.0225072

CEST -0.00056267

CESV -0.210697

CESW 0.443084

CESY 0.299129

CETA -0.304437

CETC 0.474495

CETD -0.0767436

CETE -0.0662003

CETF 0.31426

CETG -0.475406

CETH 0.282479

CETI 0.0510581

CETK -0.0937812

CETL -0.194296

CETM 0.289591

CETN 0.071233

CETP -0.18174

CETQ 0.180698

CETR 0.0800605

CETS -0.0296836

CETT 0.0762148

CETV -0.048044

CETW 0.563919

CETY 0.402284

CEVA -0.281183

CEVC 0.670814

CEVD -0.260134

CEVE -0.248378

CEVF 0.38994

CEVG -0.641359

CEVH 0.298092

CEVI 0.144468

CEVK -0.1912

CEVL -0.191593

CEVM 0.558561

CEVN -0.120533

CEVP -0.218199

CEVQ 0.0257006

CEVR -0.03809

CEVS -0.206661

CEVT -0.0697219

CEVV -0.0172911

CEVW 0.751674

CEVY 0.397413

CEWA 0.336788

CEWC 1.11579

CEWD 0.349615

CEWE 0.339831

CEWF 1.13801

CEWG 0.042412

CEWH 0.720045

CEWI 0.89797

CEWK 0.363447

CEWL 0.779248

CEWM 0.936864

CEWN 0.520589

CEWP 0.349907

CEWQ 0.618009

CEWR 0.645308

CEWS 0.367978

CEWT 0.455453

CEWV 0.788607

CEWW 1.27881

CEWY 1.22604

CEYA 0.0850821

CEYC 1.12586

CEYD 0.288076

CEYE 0.167839

CEYF 0.838759

CEYG -0.112422

CEYH 0.685241

CEYI 0.524932

CEYK 0.261805

CEYL 0.277731

CEYM 0.803797

CEYN 0.411656

CEYP 0.286573

CEYQ 0.499925

CEYR 0.468318

CEYS 0.235862

CEYT 0.348022

CEYV 0.378164

CEYW 1.1745

CEYY 0.905546

CFAA -0.542627

CFAC 0.229639

CFAD -0.476977

CFAE -0.465139

CFAF 0.101832

CFAG -0.911453

CFAH 0.0418494

CFAI -0.185306

CFAK -0.399773

CFAL -0.471351

CFAM 0.222342

CFAN -0.32541

CFAP -0.605873

CFAQ -0.173403

CFAR -0.238798

CFAS -0.46441

CFAT -0.35388

CFAV -0.331502

CFAW 0.393797

CFAY 0.105363

CFCA 0.446442

CFCC 2.34879

CFCD 0.0901539

CFCE 0.0441487

CFCF 4.13476

CFCG -0.0195811

CFCH 0.763759

CFCI 0.687676

CFCK 0.231757

CFCL 0.790481

CFCM 0.760776

CFCN 0.302527

CFCP 0.183934

CFCQ 0.476169

CFCR 0.468549

CFCS 0.620211

CFCT 0.255918

CFCV 0.666183

CFCW 1.13987

CFCY 1.0901

CFDA -0.404433

CFDC 0.111741

CFDD -0.209661

CFDE -0.247035

CFDF 0.105318

CFDG -0.574393

CFDH 0.261928

CFDI -0.172827

CFDK 0.00683713

CFDL -0.395517

CFDM 0.0274293

CFDN 0.0603092

CFDP -0.337481

CFDQ 0.00860906

CFDR 0.155293

CFDS -0.132183

CFDT -0.074316

CFDV -0.272157

CFDW 0.409593

CFDY 0.285807

CFEA -0.473521

CFEC 0.050884

CFED -0.338289

CFEE -0.278106

CFEF 0.0335593

CFEG -0.83035

CFEH 0.172304

CFEI -0.209096

CFEK -0.0299957

CFEL -0.413842

CFEM 0.0631721

CFEN -0.125592

CFEP -0.468306

CFEQ -0.00455117

CFER 0.105536

CFES -0.338582

CFET -0.230903

CFEV -0.32905

CFEW 0.433873

CFEY 0.160422

CFFA 0.0290227

CFFC 0.918072

CFFD 0.129545

CFFE 0.0396762

CFFF 0.81628

CFFG -0.302519

CFFH 0.677279

CFFI 0.491884

CFFK 0.117824

CFFL 0.206002

CFFM 0.883778

CFFN 0.22387

CFFP 0.123744

CFFQ 0.354079

CFFR 0.295646

CFFS 0.162333

CFFT 0.243358

CFFV 0.317693

CFFW 1.09761

CFFY 0.770916

CFGA -0.902255

CFGC -0.144423

CFGD -0.548124

CFGE -0.683872

CFGF -0.21295

CFGG -1.75288

CFGH -0.32132

CFGI -0.505519

CFGK -0.53409

CFGL -0.694349

CFGM -0.266682

CFGN -0.428653

CFGP -0.794018

CFGQ -0.408138

CFGR -0.375725

CFGS -0.555435

CFGT -0.430625

CFGV -0.586782

CFGW -0.00974059

CFGY -0.112328

CFHA -0.0336621

CFHC 0.689662

CFHD 0.31585

CFHE 0.187172

CFHF 0.681706

CFHG -0.330099

CFHH 0.764352

CFHI 0.32263

CFHK 0.0242524

CFHL 0.264003

CFHM 0.571442

CFHN 0.244395

CFHP 0.049813

CFHQ 0.232729

CFHR 0.326007

CFHS 0.205293

CFHT 0.27547

CFHV 0.26514

CFHW 0.950864

CFHY 0.732943

CFIA -0.177297

CFIC 0.719851

CFID -0.177437

CFIE -0.19649

CFIF 0.52897

CFIG -0.562896

CFIH 0.376514

CFII 0.285676

CFIK -0.12519

CFIL -0.0586088

CFIM 0.688451

CFIN -0.0199838

CFIP -0.134882

CFIQ 0.109456

CFIR 0.063812

CFIS -0.117752

CFIT 0.0340083

CFIV 0.0869946

CFIW 0.841262

CFIY 0.539158

CFKA -0.442266

CFKC 0.186103

CFKD -0.043545

CFKE -0.0345695

CFKF 0.100744

CFKG -0.730283

CFKH 0.00514936

CFKI -0.139422

CFKK -0.180314

CFKL -0.366499

CFKM 0.0467587

CFKN -0.0865698

CFKP -0.395562

CFKQ 0.0222383

CFKR -0.17569

CFKS -0.248397

CFKT -0.155719

CFKV -0.257053

CFKW 0.320947

CFKY 0.241207

CFLA -0.481111

CFLC 0.680876

CFLD -0.416471

CFLE -0.415342

CFLF 0.247481

CFLG -0.762849

CFLH 0.266053

CFLI -0.0643823

CFLK -0.339983

CFLL -0.418985

CFLM 0.488189

CFLN -0.248312

CFLP -0.318207

CFLQ -0.0170906

CFLR -0.135209

CFLS -0.377435

CFLT -0.256907

CFLV -0.258527

CFLW 0.737325

CFLY 0.260835

CFMA 0.191755

CFMC 0.830985

CFMD 0.0911829

CFME 0.107667

CFMF 0.891337

CFMG -0.43288

CFMH 0.461688

CFMI 0.661019

CFMK 0.132357

CFML 0.482022

CFMM 0.905072

CFMN 0.129405

CFMP -0.0585067

CFMQ 0.284552

CFMR 0.323101

CFMS 0.0765493

CFMT 0.253507

CFMV 0.510631

CFMW 0.864567

CFMY 0.822614

CFNA -0.317488

CFNC 0.313522

CFND 0.00069213

CFNE -0.11492

CFNF 0.247192

CFNG -0.465331

CFNH 0.264535

CFNI -0.0542216

CFNK -0.00678396

CFNL -0.234687

CFNM 0.140545

CFNN 0.217812

CFNP -0.155383

CFNQ 0.170689

CFNR 0.0962856

CFNS -0.0261662

CFNT 0.0289419

CFNV -0.153456

CFNW 0.602318

CFNY 0.409612

CFPA -0.307171

CFPC 0.263144

CFPD -0.0908408

CFPE -0.0666704

CFPF 0.285893

CFPG -0.489647

CFPH 0.228236

CFPI -0.0331628

CFPK -0.146351

CFPL -0.188128

CFPM 0.21006

CFPN -0.00938439

CFPP -0.180052

CFPQ 0.102952

CFPR 0.0674429

CFPS -0.0762525

CFPT -0.0227647

CFPV -0.051635

CFPW 0.659393

CFPY 0.375723

CFQA -0.15393

CFQC 0.470171

CFQD -0.0385988

CFQE -0.0183413

CFQF 0.390501

CFQG -0.434445

CFQH 0.219573

CFQI 0.0759854

CFQK 0.0523429

CFQL -0.0437706

CFQM 0.236673

CFQN 0.141556

CFQP -0.187172

CFQQ 0.362088

CFQR 0.190417

CFQS 0.0161455

CFQT 0.0979764

CFQV -0.00846052

CFQW 0.619819

CFQY 0.495323

CFRA -0.273252

CFRC 0.379041

CFRD 0.118614

CFRE 0.103176

CFRF 0.340392

CFRG -0.564126

CFRH 0.303302

CFRI 0.0649998

CFRK -0.173517

CFRL -0.150433

CFRM 0.353285

CFRN 0.0284398

CFRP -0.141354

CFRQ 0.198885

CFRR 0.141954

CFRS -0.0607624

CFRT -0.0307016

CFRV -0.0626051

CFRW 0.591753

CFRY 0.436131

CFSA -0.458091

CFSC 0.382832

CFSD -0.162554

CFSE -0.21397

CFSF 0.188218

CFSG -0.509974

CFSH 0.217392

CFSI -0.148628

CFSK -0.176601

CFSL -0.342742

CFSM 0.131058

CFSN -0.00706029

CFSP -0.335091

CFSQ 0.0980484

CFSR -0.0102634

CFSS -0.0751228

CFST -0.0407186

CFSV -0.255193

CFSW 0.451321

CFSY 0.26763

CFTA -0.348523

CFTC 0.48109

CFTD -0.114302

CFTE -0.134598

CFTF 0.279253

CFTG -0.504914

CFTH 0.279841

CFTI 0.0137393

CFTK -0.128403

CFTL -0.248339

CFTM 0.287254

CFTN 0.0420682

CFTP -0.200308

CFTQ 0.156842

CFTR 0.0433369

CFTS -0.0691781

CFTT 0.028398

CFTV -0.102282

CFTW 0.566971

CFTY 0.36916

CFVA -0.34443

CFVC 0.659138

CFVD -0.298785

CFVE -0.318123

CFVF 0.37516

CFVG -0.673311

CFVH 0.272548

CFVI 0.0873637

CFVK -0.239359

CFVL -0.24528

CFVM 0.526085

CFVN -0.151593

CFVP -0.249118

CFVQ -0.00220728

CFVR -0.079561

CFVS -0.222831

CFVT -0.109049

CFVV -0.0836968

CFVW 0.733199

CFVY 0.348806

CFWA 0.332898

CFWC 1.14301

CFWD 0.3604

CFWE 0.342353

CFWF 1.13102

CFWG 0.0559976

CFWH 0.746617

CFWI 0.884788

CFWK 0.37217

CFWL 0.747652

CFWM 0.960519

CFWN 0.535008

CFWP 0.369387

CFWQ 0.635745

CFWR 0.650368

CFWS 0.377084

CFWT 0.463681

CFWV 0.769482

CFWW 1.30407

CFWY 1.21909

CFYA 0.041018

CFYC 1.1224

CFYD 0.254223

CFYE 0.13185

CFYF 0.812398

CFYG -0.139128

CFYH 0.682689

CFYI 0.481123

CFYK 0.230108

CFYL 0.225445

CFYM 0.797064

CFYN 0.387535

CFYP 0.268191

CFYQ 0.480078

CFYR 0.437716

CFYS 0.2047

CFYT 0.314243

CFYV 0.325115

CFYW 1.16908

CFYY 0.860256

CGAA -0.483159

CGAC 0.23091

CGAD -0.443828

CGAE -0.429868

CGAF 0.103262

CGAG -0.870998

CGAH 0.0550077

CGAI -0.134889

CGAK -0.365709

CGAL -0.420809

CGAM 0.241517

CGAN -0.296809

CGAP -0.59056

CGAQ -0.150146

CGAR -0.203396

CGAS -0.416866

CGAT -0.314217

CGAV -0.291288

CGAW 0.399242

CGAY 0.142703

CGCA 0.3043

CGCC 2.36937

CGCD 0.0804274

CGCE 0.023948

CGCF 0.899889

CGCG 2.46109

CGCH 0.744009

CGCI 0.632946

CGCK 0.390495

CGCL 0.577325

CGCM 0.732661

CGCN 0.294214

CGCP 0.168763

CGCQ 0.472183

CGCR 0.43492

CGCS 0.314935

CGCT 0.29118

CGCV 0.632862

CGCW 1.1193

CGCY 0.923225

CGDA -0.367579

CGDC 0.0994947

CGDD -0.178904

CGDE -0.212617

CGDF 0.127311

CGDG -0.549111

CGDH 0.269549

CGDI -0.145425

CGDK 0.0477116

CGDL -0.353535

CGDM 0.0242403

CGDN 0.0904238

CGDP -0.324414

CGDQ 0.0279968

CGDR 0.193997

CGDS -0.0949469

CGDT -0.0401268

CGDV -0.237192

CGDW 0.404986

CGDY 0.316225

CGEA -0.433815

CGEC 0.039253

CGED -0.312021

CGEE -0.242435

CGEF 0.0607252

CGEG -0.814755

CGEH 0.180304

CGEI -0.175846

CGEK 0.0137327

CGEL -0.370746

CGEM 0.0654771

CGEN -0.0992372

CGEP -0.462862

CGEQ 0.0185978

CGER 0.143494

CGES -0.308401

CGET -0.198668

CGEV -0.289323

CGEW 0.430039

CGEY 0.185387

CGFA 0.0692644

CGFC 0.917711

CGFD 0.158024

CGFE 0.0667405

CGFF 0.832862

CGFG -0.282625

CGFH 0.685999

CGFI 0.536199

CGFK 0.14463

CGFL 0.255601

CGFM 0.90075

CGFN 0.24348

CGFP 0.139412

CGFQ 0.368378

CGFR 0.322838

CGFS 0.193526

CGFT 0.27579

CGFV 0.364997

CGFW 1.10569

CGFY 0.809233

CGGA -0.878068

CGGC -0.157439

CGGD -0.523459

CGGE -0.660367

CGGF -0.194481

CGGG -1.766

CGGH -0.326557

CGGI -0.479076

CGGK -0.506942

CGGL -0.656889

CGGM -0.2718

CGGN -0.409699

CGGP -0.791554

CGGQ -0.397385

CGGR -0.351668

CGGS -0.532207

CGGT -0.400764

CGGV -0.553972

CGGW -0.0202329

CGGY -0.088594

CGHA -0.0244682

CGHC 0.670135

CGHD 0.326477

CGHE 0.196202

CGHF 0.684125

CGHG -0.335085

CGHH 0.755692

CGHI 0.33214

CGHK 0.0226617

CGHL 0.291533

CGHM 0.556545

CGHN 0.240807

CGHP 0.0407932

CGHQ 0.223278

CGHR 0.327709

CGHS 0.211561

CGHT 0.282618

CGHV 0.282441

CGHW 0.935739

CGHY 0.739078

CGIA -0.132324

CGIC 0.727088

CGID -0.147163

CGIE -0.162529

CGIF 0.548781

CGIG -0.540069

CGIH 0.385543

CGII 0.341695

CGIK -0.0908709

CGIL -0.00569415

CGIM 0.71284

CGIN 0.0045104

CGIP -0.121663

CGIQ 0.13101

CGIR 0.097934

CGIS -0.0831811

CGIT 0.0732758

CGIV 0.138567

CGIW 0.852634

CGIY 0.582772

CGKA -0.408513

CGKC 0.175798

CGKD -0.00461793

CGKE 0.00508666

CGKF 0.127061

CGKG -0.712819

CGKH 0.00383186

CGKI -0.107846

CGKK -0.147371

CGKL -0.322758

CGKM 0.0404091

CGKN -0.063118

CGKP -0.384979

CGKQ 0.0413704

CGKR -0.152068

CGKS -0.218955

CGKT -0.12399

CGKV -0.218322

CGKW 0.314791

CGKY 0.269983

CGLA -0.429491

CGLC 0.70835

CGLD -0.376275

CGLE -0.370057

CGLF 0.263224

CGLG -0.725893

CGLH 0.293067

CGLI -0.0154588

CGLK -0.301543

CGLL -0.351233

CGLM 0.51513

CGLN -0.215693

CGLP -0.281068

CGLQ 0.0200455

CGLR -0.0910964

CGLS -0.328726

CGLT -0.20856

CGLV -0.204009

CGLW 0.766554

CGLY 0.304819

CGMA 0.211044

CGMC 0.813017

CGMD 0.0900791

CGME 0.109382

CGMF 0.902022

CGMG -0.443315

CGMH 0.444682

CGMI 0.676791

CGMK 0.133412

CGML 0.513225

CGMM 0.898564

CGMN 0.120879

CGMP -0.0719435

CGMQ 0.274809

CGMR 0.323669

CGMS 0.0756085

CGMT 0.257796

CGMV 0.534118

CGMW 0.846346

CGMY 0.833094

CGNA -0.288931

CGNC 0.29927

CGND 0.0264003

CGNE -0.0885785

CGNF 0.255483

CGNG -0.447476

CGNH 0.261986

CGNI -0.032557

CGNK 0.0198436

CGNL -0.198631

CGNM 0.131536

CGNN 0.241734

CGNP -0.15076

CGNQ 0.183867

CGNR 0.117521

CGNS 0.00100636

CGNT 0.0556543

CGNV -0.122991

CGNW 0.596142

CGNY 0.435352

CGPA -0.276792

CGPC 0.248371

CGPD -0.0673914

CGPE -0.0374184

CGPF 0.306261

CGPG -0.473042

CGPH 0.225373

CGPI -0.0115535

CGPK -0.124797

CGPL -0.154431

CGPM 0.203025

CGPN 0.00638986

CGPP -0.171049

CGPQ 0.115369

CGPR 0.0895076

CGPS -0.0505748

CGPT 0.00327682

CGPV -0.0182512

CGPW 0.655318

CGPY 0.3983

CGQA -0.126075

CGQC 0.455542

CGQD -0.0214498

CGQE 0.00566077

CGQF 0.41044

CGQG -0.424293

CGQH 0.209678

CGQI 0.0969276

CGQK 0.0743585

CGQL -0.00705981

CGQM 0.225206

CGQN 0.153361

CGQP -0.187046

CGQQ 0.377207

CGQR 0.209411

CGQS 0.0379035

CGQT 0.11995

CGQV 0.0181346

CGQW 0.608502

CGQY 0.514614

CGRA -0.237586

CGRC 0.368873

CGRD 0.155691

CGRE 0.144372

CGRF 0.364901

CGRG -0.547232

CGRH 0.305225

CGRI 0.0983269

CGRK -0.149429

CGRL -0.108311

CGRM 0.355315

CGRN 0.0481308

CGRP -0.128487

CGRQ 0.218407

CGRR 0.170902

CGRS -0.0325565

CGRT -0.00215816

CGRV -0.0249732

CGRW 0.587827

CGRY 0.465951

CGSA -0.415983

CGSC 0.379434

CGSD -0.129701

CGSE -0.176529

CGSF 0.198812

CGSG -0.480958

CGSH 0.225548

CGSI -0.118258

CGSK -0.145374

CGSL -0.300125

CGSM 0.131239

CGSN 0.0205157

CGSP -0.32057

CGSQ 0.121082

CGSR 0.0230298

CGSS -0.0329757

CGST -0.00254631

CGSV -0.215915

CGSW 0.44766

CGSY 0.296412

CGTA -0.30879

CGTC 0.479269

CGTD -0.0810027

CGTE -0.095367

CGTF 0.30695

CGTG -0.474021

CGTH 0.285338

CGTI 0.0467479

CGTK -0.0975344

CGTL -0.199346

CGTM 0.292665

CGTN 0.0695503

CGTP -0.184518

CGTQ 0.180559

CGTR 0.0751042

CGTS -0.0331154

CGTT 0.0706944

CGTV -0.0578358

CGTW 0.567743

CGTY 0.398817

CGVA -0.28122

CGVC 0.671618

CGVD -0.263669

CGVE -0.280198

CGVF 0.382372

CGVG -0.642465

CGVH 0.287969

CGVI 0.132283

CGVK -0.204361

CGVL -0.201484

CGVM 0.556643

CGVN -0.124848

CGVP -0.223325

CGVQ 0.0262613

CGVR -0.0459301

CGVS -0.213934

CGVT -0.0711219

CGVV -0.0224748

CGVW 0.75165

CGVY 0.392168

CGWA 0.33913

CGWC 1.12341

CGWD 0.35475

CGWE 0.339389

CGWF 1.13979

CGWG 0.047972

CGWH 0.72763

CGWI 0.898713

CGWK 0.36717

CGWL 0.776542

CGWM 0.944013

CGWN 0.526037

CGWP 0.356511

CGWQ 0.624421

CGWR 0.650122

CGWS 0.372702

CGWT 0.460274

CGWV 0.788274

CGWW 1.28617

CGWY 1.22793

CGYA 0.0787683

CGYC 1.12841

CGYD 0.28202

CGYE 0.160177

CGYF 0.833974

CGYG -0.117359

CGYH 0.688531

CGYI 0.521211

CGYK 0.258338

CGYL 0.271832

CGYM 0.806324

CGYN 0.410241

CGYP 0.286417

CGYQ 0.498537

CGYR 0.466934

CGYS 0.235591

CGYT 0.343358

CGYV 0.366351

CGYW 1.17696

CGYY 0.899991

CHAA -0.45492

CHAC 0.2153

CHAD -0.427101

CHAE -0.40739

CHAF 0.129009

CHAG -0.880813

CHAH 0.0545337

CHAI -0.103801

CHAK -0.337315

CHAL -0.385027

CHAM 0.242058

CHAN -0.282664

CHAP -0.593516

CHAQ -0.142579

CHAR -0.176321

CHAS -0.400104

CHAT -0.294048

CHAV -0.254339

CHAW 0.390498

CHAY 0.164051

CHCA 0.28542

CHCC 2.37522

CHCD 0.0534174

CHCE -0.00400901

CHCF 0.881739

CHCG -0.0574815

CHCH 1.90487

CHCI 0.619701

CHCK 0.224725

CHCL 0.608335

CHCM 0.701106

CHCN 0.267394

CHCP 0.138293

CHCQ 0.467339

CHCR 0.443743

CHCS 0.305695

CHCT 0.281867

CHCV 0.640558

CHCW 1.08786

CHCY 0.908571

CHDA -0.34585

CHDC 0.071501

CHDD -0.165234

CHDE -0.196317

CHDF 0.135134

CHDG -0.544302

CHDH 0.259876

CHDI -0.130461

CHDK 0.0733731

CHDL -0.329097

CHDM 0.00324845

CHDN 0.102881

CHDP -0.330267

CHDQ 0.0314114

CHDR 0.216092

CHDS -0.0768623

CHDT -0.0215497

CHDV -0.218208

CHDW 0.38352

CHDY 0.329451

CHEA -0.413132

CHEC 0.011802

CHED -0.300992

CHEE -0.220358

CHEF 0.0707879

CHEG -0.816075

CHEH 0.170313

CHEI -0.160473

CHEK 0.0413058

CHEL -0.342834

CHEM 0.0494773

CHEN -0.0911067

CHEP -0.470786

CHEQ 0.0297873

CHER 0.167687

CHES -0.296381

CHET -0.184403

CHEV -0.269309

CHEW 0.41239

CHEY 0.19644

CHFA 0.0925994

CHFC 0.901129

CHFD 0.168013

CHFE 0.0766768

CHFF 0.85745

CHFG -0.283596

CHFH 0.676976

CHFI 0.563685

CHFK 0.155473

CHFL 0.291076

CHFM 0.898631

CHFN 0.244625

CHFP 0.13705

CHFQ 0.367589

CHFR 0.332484

CHFS 0.206208

CHFT 0.289483

CHFV 0.394528

CHFW 1.09518

CHFY 0.827358

CHGA -0.871796

CHGC -0.185811

CHGD -0.513373

CHGE -0.655737

CHGF -0.189884

CHGG -1.79483

CHGH -0.347917

CHGI -0.472097

CHGK -0.498681

CHGL -0.635217

CHGM -0.293864

CHGN -0.410643

CHGP -0.806999

CHGQ -0.404348

CHGR -0.344884

CHGS -0.526009

CHGT -0.388839

CHGV -0.539242

CHGW -0.0465739

CHGY -0.0843673

CHHA -0.0319331

CHHC 0.638839

CHHD 0.318677

CHHE 0.186751

CHHF 0.674951

CHHG -0.358449

CHHH 0.730692

CHHI 0.32274

CHHK 0.00497246

CHHL 0.300414

CHHM 0.526979

CHHN 0.220435

CHHP 0.0153649

CHHQ 0.19723

CHHR 0.312204

CHHS 0.199517

CHHT 0.271236

CHHV 0.280612

CHHW 0.906002

CHHY 0.727973

CHIA -0.102028

CHIC 0.716581

CHID -0.1351

CHIE -0.147683

CHIF 0.576239

CHIG -0.536362

CHIH 0.38255

CHII 0.376297

CHIK -0.0743937

CHIL 0.0339525

CHIM 0.719089

CHIN 0.00971746

CHIP -0.120254

CHIQ 0.134821

CHIR 0.11221

CHIS -0.0662038

CHIT 0.0943964

CHIV 0.175062

CHIW 0.846104

CHIY 0.606662

CHKA -0.389116

CHKC 0.149892

CHKD 0.0181844

CHKE 0.0326364

CHKF 0.136384

CHKG -0.713568

CHKH -0.0140524

CHKI -0.0929904

CHKK -0.131248

CHKL -0.296285

CHKM 0.0206804

CHKN -0.0578032

CHKP -0.39285

CHKQ 0.0434361

CHKR -0.146084

CHKS -0.206672

CHKT -0.109911

CHKV -0.197994

CHKW 0.291193

CHKY 0.281551

CHLA -0.392487

CHLC 0.717075

CHLD -0.353393

CHLE -0.337718

CHLF 0.297446

CHLG -0.710093

CHLH 0.306914

CHLI 0.0233381

CHLK -0.268571

CHLL -0.308153

CHLM 0.533436

CHLN -0.199024

CHLP -0.268567

CHLQ 0.0397298

CHLR -0.062757

CHLS -0.303087

CHLT -0.175098

CHLV -0.163096

CHLW 0.776353

CHLY 0.338723

CHMA 0.210449

CHMC 0.782249

CHMD 0.0714147

CHME 0.0959671

CHMF 0.903954

CHMG -0.470183

CHMH 0.414552

CHMI 0.680276

CHMK 0.116178

CHML 0.530574

CHMM 0.875107

CHMN 0.0952096

CHMP -0.10052

CHMQ 0.248986

CHMR 0.306834

CHMS 0.0569079

CHMT 0.243381

CHMV 0.541878

CHMW 0.815566

CHMY 0.823714

CHNA -0.27963

CHNC 0.270188

CHND 0.0359499

CHNE -0.0802395

CHNF 0.25697

CHNG -0.449404

CHNH 0.244844

CHNI -0.0280938

CHNK 0.0308161

CHNL -0.181686

CHNM 0.106217

CHNN 0.249047

CHNP -0.160904

CHNQ 0.181221

CHNR 0.122024

CHNS 0.00987792

CHNT 0.0643847

CHNV -0.113401

CHNW 0.572369

CHNY 0.440757

CHPA -0.264778

CHPC 0.218923

CHPD -0.0606365

CHPE -0.0247574

CHPF 0.312631

CHPG -0.475061

CHPH 0.206992

CHPI -0.00500035

CHPK -0.120512

CHPL -0.133057

CHPM 0.181824

CHPN 0.00302911

CHPP -0.180816

CHPQ 0.10893

CHPR 0.0946269

CHPS -0.0429902

CHPT 0.0107746

CHPV -0.00057674

CHPW 0.633908

CHPY 0.40292

CHQA -0.115951

CHQC 0.426118

CHQD -0.0224674

CHQE 0.00998616

CHQF 0.410988

CHQG -0.433729

CHQH 0.183933

CHQI 0.0991797

CHQK 0.0788717

CHQL 0.0140026

CHQM 0.198378

CHQN 0.147801

CHQP -0.204903

CHQQ 0.373482

CHQR 0.213544

CHQS 0.0402315

CHQT 0.123564

CHQV 0.0273843

CHQW 0.581202

CHQY 0.515632

CHRA -0.220426

CHRC 0.342371

CHRD 0.176498

CHRE 0.168647

CHRF 0.376001

CHRG -0.551068

CHRH 0.289232

CHRI 0.113598

CHRK -0.143843

CHRL -0.0808396

CHRM 0.33922

CHRN 0.0496047

CHRP -0.133904

CHRQ 0.220117

CHRR 0.181302

CHRS -0.0206423

CHRT 0.00807762

CHRV -0.00450253

CHRW 0.566226

CHRY 0.477125

CHSA -0.395606

CHSC 0.358412

CHSD -0.113457

CHSE -0.158319

CHSF 0.213237

CHSG -0.470288

CHSH 0.223643

CHSI -0.104572

CHSK -0.128006

CHSL -0.272197

CHSM 0.114156

CHSN 0.0297773

CHSP -0.326155

CHSQ 0.12685

CHSR 0.0369725

CHSS -0.0102787

CHST 0.01754

CHSV -0.195476

CHSW 0.427657

CHSY 0.312205

CHTA -0.287946

CHTC 0.459468

CHTD -0.0651011

CHTE -0.0770574

CHTF 0.324547

CHTG -0.470822

CHTH 0.279301

CHTI 0.0693009

CHTK -0.0805933

CHTL -0.170533

CHTM 0.280292

CHTN 0.0792687

CHTP -0.185889

CHTQ 0.187114

CHTR 0.0887938

CHTS -0.0145998

CHTT 0.0928974

CHTV -0.028784

CHTW 0.549981

CHTY 0.413062

CHVA -0.245504

CHVC 0.667987

CHVD -0.24614

CHVE -0.252629

CHVF 0.416397

CHVG -0.635473

CHVH 0.302142

CHVI 0.170632

CHVK -0.180645

CHVL -0.162328

CHVM 0.56563

CHVN -0.105931

CHVP -0.214161

CHVQ 0.0352931

CHVR -0.0252903

CHVS -0.193161

CHVT -0.0469134

CHVV 0.017252

CHVW 0.75107

CHVY 0.418094

CHWA 0.326824

CHWC 1.09204

CHWD 0.331814

CHWE 0.318774

CHWF 1.13056

CHWG 0.0230992

CHWH 0.697027

CHWI 0.893944

CHWK 0.344912

CHWL 0.786879

CHWM 0.913944

CHWN 0.500498

CHWP 0.328227

CHWQ 0.59727

CHWR 0.630524

CHWS 0.350703

CHWT 0.439154

CHWV 0.791862

CHWW 1.25547

CHWY 1.21827

CHYA 0.0960922

CHYC 1.11582

CHYD 0.293633

CHYE 0.171092

CHYF 0.853605

CHYG -0.114865

CHYH 0.678745

CHYI 0.54372

CHYK 0.270248

CHYL 0.30557

CHYM 0.795617

CHYN 0.414218

CHYP 0.285964

CHYQ 0.498869

CHYR 0.477134

CHYS 0.245427

CHYT 0.356857

CHYV 0.390839

CHYW 1.16525

CHYY 0.91844

CIAA -0.540145

CIAC 0.220531

CIAD -0.492187

CIAE -0.478013

CIAF 0.0468812

CIAG -0.917385

CIAH 0.0328672

CIAI -0.0872371

CIAK -0.414003

CIAL -0.442036

CIAM 0.204998

CIAN -0.332659

CIAP -0.615839

CIAQ -0.18921

CIAR -0.250712

CIAS -0.471755

CIAT -0.373516

CIAV -0.349634

CIAW 0.382548

CIAY 0.0876946

CICA 0.292111

CICC 2.33624

CICD 0.0957248

CICE 0.0398829

CICF 0.951787

CICG -0.0224135

CICH 0.765831

CICI 4.03525

CICK 0.248951

CICL 0.619931

CICM 0.857849

CICN 0.315201

CICP 0.190603

CICQ 0.465125

CICR 0.473696

CICS 0.319288

CICT 0.379397

CICV 1.04018

CICW 1.14136

CICY 0.92558

CIDA -0.420728

CIDC 0.110128

CIDD -0.222862

CIDE -0.259784

CIDF 0.0860338

CIDG -0.586034

CIDH 0.252783

CIDI -0.190407

CIDK -0.0101593

CIDL -0.410936

CIDM 0.0249569

CIDN 0.0463912

CIDP -0.346202

CIDQ -0.00249791

CIDR 0.138185

CIDS -0.144712

CIDT -0.0908327

CIDV -0.285177

CIDW 0.404579

CIDY 0.271379

CIEA -0.489147

CIEC 0.0486753

CIED -0.35392

CIEE -0.293972

CIEF 0.0178533

CIEG -0.840353

CIEH 0.163924

CIEI -0.226031

CIEK -0.0486844

CIEL -0.425645

CIEM 0.0624912

CIEN -0.137622

CIEP -0.482292

CIEQ -0.0168087

CIER 0.0846782

CIES -0.3516

CIET -0.245023

CIEV -0.341345

CIEW 0.424075

CIEY 0.149587

CIFA 0.0128765

CIFC 0.910415

CIFD 0.116699

CIFE 0.0246916

CIFF 0.773835

CIFG -0.313041

CIFH 0.669245

CIFI 0.481765

CIFK 0.104039

CIFL 0.185935

CIFM 0.872416

CIFN 0.214356

CIFP 0.114662

CIFQ 0.341325

CIFR 0.287621

CIFS 0.148849

CIFT 0.230509

CIFV 0.315035

CIFW 1.08962

CIFY 0.76081

CIGA -0.915293

CIGC -0.145994

CIGD -0.560756

CIGE -0.696223

CIGF -0.230307

CIGG -1.7546

CIGH -0.325482

CIGI -0.512964

CIGK -0.540443

CIGL -0.71037

CIGM -0.268708

CIGN -0.439335

CIGP -0.800968

CIGQ -0.417123

CIGR -0.387335

CIGS -0.569177

CIGT -0.442154

CIGV -0.593739

CIGW -0.0128763

CIGY -0.119632

CIHA -0.0429518

CIHC 0.690276

CIHD 0.307246

CIHE 0.177698

CIHF 0.666103

CIHG -0.333382

CIHH 0.761378

CIHI 0.336072

CIHK 0.0187602

CIHL 0.252357

CIHM 0.569993

CIHN 0.240148

CIHP 0.0469811

CIHQ 0.230036

CIHR 0.319797

CIHS 0.197565

CIHT 0.267476

CIHV 0.251772

CIHW 0.950231

CIHY 0.723663

CIIA -0.194843

CIIC 0.711106

CIID -0.187466

CIIE -0.209727

CIIF 0.487442

CIIG -0.577275

CIIH 0.364782

CIII 0.29122

CIIK -0.130055

CIIL -0.0801117

CIIM 0.680669

CIIN -0.0321407

CIIP -0.148591

CIIQ 0.0971785

CIIR 0.0492671

CIIS -0.130656

CIIT 0.0211904

CIIV 0.0901585

CIIW 0.832524

CIIY 0.525234

CIKA -0.459904

CIKC 0.18327

CIKD -0.0596554

CIKE -0.0516064

CIKF 0.0849102

CIKG -0.740811

CIKH 6.151e-05

CIKI -0.152467

CIKK -0.19521

CIKL -0.389539

CIKM 0.0372448

CIKN -0.0989509

CIKP -0.404584

CIKQ 0.00925255

CIKR -0.187662

CIKS -0.262156

CIKT -0.170293

CIKV -0.274541

CIKW 0.317785

CIKY 0.233621

CILA -0.507169

CILC 0.669064

CILD -0.428065

CILE -0.435277

CILF 0.196103

CILG -0.77579

CILH 0.263221

CILI -0.0249956

CILK -0.362954

CILL -0.413532

CILM 0.454024

CILN -0.264634

CILP -0.331506

CILQ -0.0340049

CILR -0.152358

CILS -0.390532

CILT -0.269195

CILV -0.263752

CILW 0.726022

CILY 0.236267

CIMA 0.181776

CIMC 0.831097

CIMD 0.0855086

CIME 0.101146

CIMF 0.881426

CIMG -0.435283

CIMH 0.461564

CIMI 0.667382

CIMK 0.126085

CIML 0.463863

CIMM 0.904558

CIMN 0.125268

CIMP -0.0599754

CIMQ 0.281551

CIMR 0.317319

CIMS 0.069732

CIMT 0.245215

CIMV 0.500172

CIMW 0.86442

CIMY 0.815806

CINA -0.32958

CINC 0.312809

CIND -0.013447

CINE -0.127608

CINF 0.222891

CING -0.474513

CINH 0.25974

CINI -0.0659375

CINK -0.0201831

CINL -0.226411

CINM 0.136561

CINN 0.20475

CINP -0.167159

CINQ 0.160176

CINR 0.0825689

CINS -0.0403445

CINT 0.0148637

CINV -0.171117

CINW 0.599072

CINY 0.400889

CIPA -0.320277

CIPC 0.262017

CIPD -0.103231

CIPE -0.0811739

CIPF 0.270336

CIPG -0.499428

CIPH 0.223687

CIPI -0.0468004

CIPK -0.158119

CIPL -0.210618

CIPM 0.204237

CIPN -0.018472

CIPP -0.187554

CIPQ 0.0940759

CIPR 0.0554323

CIPS -0.0892153

CIPT -0.035255

CIPV -0.0667036

CIPW 0.654788

CIPY 0.36345

CIQA -0.166917

CIQC 0.46927

CIQD -0.0489576

CIQE -0.0293128

CIQF 0.37995

CIQG -0.442949

CIQH 0.216852

CIQI 0.0669599

CIQK 0.0426717

CIQL -0.061269

CIQM 0.232736

CIQN 0.13221

CIQP -0.192208

CIQQ 0.354181

CIQR 0.178946

CIQS 0.00660586

CIQT 0.086333

CIQV -0.0216908

CIQW 0.617896

CIQY 0.483784

CIRA -0.288044

CIRC 0.376461

CIRD 0.102717

CIRE 0.0855207

CIRF 0.326962

CIRG -0.57344

CIRH 0.297111

CIRI 0.0705369

CIRK -0.185218

CIRL -0.17141

CIRM 0.348788

CIRN 0.0166857

CIRP -0.15075

CIRQ 0.187757

CIRR 0.127094

CIRS -0.0752807

CIRT -0.0428486

CIRV -0.0834734

CIRW 0.587594

CIRY 0.426845

CISA -0.467395

CISC 0.377835

CISD -0.178905

CISE -0.220479

CISF 0.159954

CISG -0.524633

CISH 0.210226

CISI -0.115161

CISK -0.192744

CISL -0.352053

CISM 0.121332

CISN -0.0198305

CISP -0.344229

CISQ 0.0843475

CISR -0.0226183

CISS -0.0866818

CIST -0.0578365

CISV -0.254934

CISW 0.444426

CISY 0.251634

CITA -0.356024

CITC 0.47644

CITD -0.12886

CITE -0.149257

CITF 0.259999

CITG -0.516603

CITH 0.274014

CITI 0.0316775

CITK -0.144

CITL -0.262811

CITM 0.283621

CITN 0.029175

CITP -0.208555

CITQ 0.143675

CITR 0.0280728

CITS -0.0854764

CITT 0.0145011

CITV -0.119992

CITW 0.561561

CITY 0.354424

CIVA -0.350402

CIVC 0.650895

CIVD -0.314588

CIVE -0.337923

CIVF 0.324801

CIVG -0.68578

CIVH 0.260636

CIVI 0.144706

CIVK -0.257409

CIVL -0.245427

CIVM 0.513708

CIVN -0.166272

CIVP -0.25995

CIVQ -0.0140243

CIVR -0.0963061

CIVS -0.27039

CIVT -0.131743

CIVV -0.0710568

CIVW 0.726192

CIVY 0.3318

CIWA 0.325507

CIWC 1.14372

CIWD 0.356625

CIWE 0.336679

CIWF 1.12239

CIWG 0.0530083

CIWH 0.747178

CIWI 0.877622

CIWK 0.368004

CIWL 0.734479

CIWM 0.959009

CIWN 0.531896

CIWP 0.367629

CIWQ 0.633194

CIWR 0.647088

CIWS 0.372554

CIWT 0.462306

CIWV 0.759518

CIWW 1.30348

CIWY 1.21127

CIYA 0.0354824

CIYC 1.11545

CIYD 0.238303

CIYE 0.118654

CIYF 0.783535

CIYG -0.150188

CIYH 0.67552

CIYI 0.491858

CIYK 0.220687

CIYL 0.215762

CIYM 0.796015

CIYN 0.378593

CIYP 0.257345

CIYQ 0.469071

CIYR 0.422395

CIYS 0.197907

CIYT 0.298968

CIYV 0.306719

CIYW 1.16234

CIYY 0.849054

CKAA -0.492042

CKAC 0.231632

CKAD -0.44269

CKAE -0.431328

CKAF 0.100449

CKAG -0.899558

CKAH 0.0567219

CKAI -0.143163

CKAK -0.328079

CKAL -0.438573

CKAM 0.239514

CKAN -0.300857

CKAP -0.591898

CKAQ -0.150229

CKAR -0.203286

CKAS -0.429162

CKAT -0.323537

CKAV -0.296859

CKAW 0.398734

CKAY 0.13621

CKCA 0.344669

CKCC 2.36885

CKCD 0.0931342

CKCE 0.0477626

CKCF 0.876327

CKCG 0.115595

CKCH 0.751315

CKCI 0.629411

CKCK 2.86131

CKCL 0.775748

CKCM 0.732073

CKCN 0.308403

CKCP 0.172838

CKCQ 0.49243

CKCR 0.648201

CKCS 0.404413

CKCT 0.407683

CKCV 0.593839

CKCW 1.12352

CKCY 0.919147

CKDA -0.373339

CKDC 0.102999

CKDD -0.182368

CKDE -0.213147

CKDF 0.122063

CKDG -0.554136

CKDH 0.268602

CKDI -0.146502

CKDK 0.0437548

CKDL -0.360715

CKDM 0.0258548

CKDN 0.0853317

CKDP -0.325977

CKDQ 0.0252473

CKDR 0.188014

CKDS -0.0992789

CKDT -0.0428276

CKDV -0.242372

CKDW 0.406907

CKDY 0.311562

CKEA -0.441168

CKEC 0.0423558

CKED -0.316321

CKEE -0.245567

CKEF 0.0569077

CKEG -0.815527

CKEH 0.183628

CKEI -0.180892

CKEK 0.0127552

CKEL -0.379858

CKEM 0.0691092

CKEN -0.103598

CKEP -0.463706

CKEQ 0.0141971

CKER 0.134745

CKES -0.313256

CKET -0.20379

CKEV -0.297752

CKEW 0.431204

CKEY 0.180821

CKFA 0.062274

CKFC 0.918652

CKFD 0.154371

CKFE 0.0653429

CKFF 0.825793

CKFG -0.287375

CKFH 0.684975

CKFI 0.529931

CKFK 0.149206

CKFL 0.246583

CKFM 0.898491

CKFN 0.243038

CKFP 0.139177

CKFQ 0.37056

CKFR 0.320662

CKFS 0.18858

CKFT 0.268939

CKFV 0.355311

CKFW 1.10543

CKFY 0.803746

CKGA -0.881094

CKGC -0.153674

CKGD -0.526341

CKGE -0.664831

CKGF -0.197702

CKGG -1.76307

CKGH -0.323036

CKGI -0.483696

CKGK -0.510293

CKGL -0.661512

CKGM -0.269954

CKGN -0.413407

CKGP -0.79113

CKGQ -0.398405

CKGR -0.351255

CKGS -0.537144

CKGT -0.405459

CKGV -0.558588

CKGW -0.0173395

CKGY -0.0919485

CKHA -0.0258186

CKHC 0.674602

CKHD 0.325339

CKHE 0.195521

CKHF 0.683939

CKHG -0.333531

CKHH 0.759562

CKHI 0.330732

CKHK 0.0301294

CKHL 0.286939

CKHM 0.560292

CKHN 0.246492

CKHP 0.0434482

CKHQ 0.225836

CKHR 0.328387

CKHS 0.210684

CKHT 0.282226

CKHV 0.280479

CKHW 0.939568

CKHY 0.739477

CKIA -0.139612

CKIC 0.726835

CKID -0.152519

CKIE -0.170785

CKIF 0.54267

CKIG -0.543937

CKIH 0.383513

CKII 0.330979

CKIK -0.0936422

CKIL -0.0170953

CKIM 0.71067

CKIN 0.00054836

CKIP -0.122909

CKIQ 0.13111

CKIR 0.0894477

CKIS -0.0809557

CKIT 0.0670011

CKIV 0.13449

CKIW 0.851488

CKIY 0.575274

CKKA -0.409576

CKKC 0.178677

CKKD -0.00808406

CKKE -0.00147271

CKKF 0.122798

CKKG -0.715669

CKKH 0.00477695

CKKI -0.11324

CKKK -0.151331

CKKL -0.329659

CKKM 0.0446005

CKKN -0.0670319

CKKP -0.386001

CKKQ 0.0381961

CKKR -0.152673

CKKS -0.223275

CKKT -0.128959

CKKV -0.225866

CKKW 0.317095

CKKY 0.26787

CKLA -0.436937

CKLC 0.705121

CKLD -0.384237

CKLE -0.379311

CKLF 0.254586

CKLG -0.733815

CKLH 0.297725

CKLI -0.0192964

CKLK -0.294566

CKLL -0.370617

CKLM 0.515863

CKLN -0.215122

CKLP -0.291183

CKLQ 0.0135858

CKLR -0.105749

CKLS -0.339783

CKLT -0.213982

CKLV -0.214037

CKLW 0.76282

CKLY 0.302358

CKMA 0.208729

CKMC 0.817262

CKMD 0.0916359

CKME 0.122373

CKMF 0.89939

CKMG -0.440945

CKMH 0.448586

CKMI 0.673771

CKMK 0.138477

CKML 0.50644

CKMM 0.902722

CKMN 0.123061

CKMP -0.0684788

CKMQ 0.27845

CKMR 0.327527

CKMS 0.0770047

CKMT 0.257595

CKMV 0.531994

CKMW 0.850576

CKMY 0.833069

CKNA -0.293262

CKNC 0.303188

CKND 0.024493

CKNE -0.0916846

CKNF 0.253057

CKNG -0.450982

CKNH 0.263476

CKNI -0.0345864

CKNK 0.0225196

CKNL -0.206989

CKNM 0.1342

CKNN 0.239873

CKNP -0.151179

CKNQ 0.181135

CKNR 0.115443

CKNS -0.00253367

CKNT 0.0511835

CKNV -0.12957

CKNW 0.598561

CKNY 0.432656

CKPA -0.281424

CKPC 0.252114

CKPD -0.0678811

CKPE -0.0396304

CKPF 0.305473

CKPG -0.475024

CKPH 0.226859

CKPI -0.0149806

CKPK -0.126651

CKPL -0.16193

CKPM 0.205127

CKPN 0.00430465

CKPP -0.171587

CKPQ 0.113555

CKPR 0.0903063

CKPS -0.0540895

CKPT -0.00083494

CKPV -0.0231192

CKPW 0.657133

CKPY 0.39461

CKQA -0.13012

CKQC 0.459279

CKQD -0.0238979

CKQE 0.00193477

CKQF 0.407637

CKQG -0.426117

CKQH 0.212434

CKQI 0.0943851

CKQK 0.0715895

CKQL -0.0128596

CKQM 0.228097

CKQN 0.15341

CKQP -0.185796

CKQQ 0.375319

CKQR 0.207438

CKQS 0.0355046

CKQT 0.117225

CKQV 0.014369

CKQW 0.611601

CKQY 0.512331

CKRA -0.244894

CKRC 0.371757

CKRD 0.150674

CKRE 0.13761

CKRF 0.359222

CKRG -0.550536

CKRH 0.308067

CKRI 0.0934832

CKRK -0.152655

CKRL -0.116169

CKRM 0.355797

CKRN 0.0449727

CKRP -0.129707

CKRQ 0.216351

CKRR 0.166762

CKRS -0.0361242

CKRT -0.00823784

CKRV -0.0295827

CKRW 0.589753

CKRY 0.461566

CKSA -0.426778

CKSC 0.381117

CKSD -0.135596

CKSE -0.184021

CKSF 0.197242

CKSG -0.486287

CKSH 0.226672

CKSI -0.122989

CKSK -0.139128

CKSL -0.310434

CKSM 0.131622

CKSN 0.0154893

CKSP -0.322958

CKSQ 0.11648

CKSR 0.0117912

CKSS -0.0389009

CKST -0.00845289

CKSV -0.221951

CKSW 0.448825

CKSY 0.293762

CKTA -0.313544

CKTC 0.480625

CKTD -0.0849204

CKTE -0.103973

CKTF 0.303604

CKTG -0.485158

CKTH 0.284249

CKTI 0.0421236

CKTK -0.091593

CKTL -0.209173

CKTM 0.292593

CKTN 0.0677712

CKTP -0.186628

CKTQ 0.181753

CKTR 0.0725913

CKTS -0.0402637

CKTT 0.0604553

CKTV -0.064574

CKTW 0.568892

CKTY 0.393255

CKVA -0.300803

CKVC 0.669443

CKVD -0.269454

CKVE -0.290393

CKVF 0.374862

CKVG -0.651987

CKVH 0.292589

CKVI 0.123024

CKVK -0.191408

CKVL -0.213872

CKVM 0.55669

CKVN -0.133882

CKVP -0.22362

CKVQ 0.0208654

CKVR -0.0392015

CKVS -0.223974

CKVT -0.0833752

CKVV -0.0342455

CKVW 0.749253

CKVY 0.384839

CKWA 0.33909

CKWC 1.1279

CKWD 0.356862

CKWE 0.340325

CKWF 1.13926

CKWG 0.050442

CKWH 0.732267

CKWI 0.897429

CKWK 0.369844

CKWL 0.772223

CKWM 0.948018

CKWN 0.528687

CKWP 0.359926

CKWQ 0.627481

CKWR 0.651017

CKWS 0.374644

CKWT 0.461917

CKWV 0.786112

CKWW 1.29034

CKWY 1.22744

CKYA 0.0761328

CKYC 1.12851

CKYD 0.277654

CKYE 0.153833

CKYF 0.831346

CKYG -0.121343

CKYH 0.687698

CKYI 0.514488

CKYK 0.260611

CKYL 0.262743

CKYM 0.805565

CKYN 0.406959

CKYP 0.283988

CKYQ 0.496388

CKYR 0.458268

CKYS 0.228666

CKYT 0.346421

CKYV 0.361855

CKYW 1.17643

CKYY 0.893331

CLAA -0.69488

CLAC 0.151294

CLAD -0.611743

CLAE -0.61231

CLAF -0.0859947

CLAG -1.02636

CLAH -0.0579627

CLAI -0.337468

CLAK -0.54686

CLAL -0.550716

CLAM 0.107272

CLAN -0.445896

CLAP -0.708654

CLAQ -0.298632

CLAR -0.380513

CLAS -0.591053

CLAT -0.490932

CLAV -0.520227

CLAW 0.302286

CLAY -0.0372562

CLCA 0.28283

CLCC 2.23257

CLCD 0.0459416

CLCE -0.0138433

CLCF 1.03308

CLCG -0.0995452

CLCH 0.732955

CLCI 0.598421

CLCK 0.373777

CLCL 4.71585

CLCM 0.959584

CLCN 0.272215

CLCP 0.148332

CLCQ 0.425805

CLCR 0.419525

CLCS 0.308157

CLCT 0.249825

CLCV 0.904171

CLCW 1.10972

CLCY 0.861678

CLDA -0.545386

CLDC 0.0629117

CLDD -0.337831

CLDE -0.377106

CLDF -0.0226645

CLDG -0.691703

CLDH 0.170993

CLDI -0.302868

CLDK -0.1416

CLDL -0.536673

CLDM -0.0414474

CLDN -0.0680263

CLDP -0.434596

CLDQ -0.101159

CLDR 0.0109615

CLDS -0.265032

CLDT -0.212607

CLDV -0.414137

CLDW 0.342457

CLDY 0.156595

CLEA -0.615325

CLEC 0.00027966

CLED -0.466385

CLEE -0.416877

CLEF -0.0927362

CLEG -0.934646

CLEH 0.0815661

CLEI -0.343344

CLEK -0.184138

CLEL -0.560323

CLEM -0.0137131

CLEN -0.24563

CLEP -0.568169

CLEQ -0.120774

CLER -0.0448351

CLES -0.464885

CLET -0.362742

CLEV -0.464961

CLEW 0.354542

CLEY 0.0312834

CLFA -0.109849

CLFC 0.838744

CLFD 0.00760794

CLFE -0.0855584

CLFF 0.647533

CLFG -0.409371

CLFH 0.58493

CLFI 0.337232

CLFK -0.00799489

CLFL 0.0540581

CLFM 0.780919

CLFN 0.117835

CLFP 0.0204115

CLFQ 0.246008

CLFR 0.172572

CLFS 0.0337276

CLFT 0.112476

CLFV 0.164358

CLFW 1.00892

CLFY 0.637519

CLGA -1.0204

CLGC -0.191993

CLGD -0.669504

CLGE -0.799927

CLGF -0.326635

CLGG -1.80002

CLGH -0.380741

CLGI -0.617729

CLGK -0.656643

CLGL -0.827446

CLGM -0.329995

CLGN -0.533276

CLGP -0.874204

CLGQ -0.502757

CLGR -0.493548

CLGS -0.678531

CLGT -0.555595

CLGV -0.717193

CLGW -0.0645144

CLGY -0.227429

CLHA -0.125414

CLHC 0.658574

CLHD 0.222081

CLHE 0.0942709

CLHF 0.582478

CLHG -0.392392

CLHH 0.707675

CLHI 0.232451

CLHK -0.0482421

CLHL 0.148792

CLHM 0.528615

CLHN 0.176476

CLHP -0.00597453

CLHQ 0.177861

CLHR 0.245216

CLHS 0.118761

CLHT 0.187631

CLHV 0.162278

CLHW 0.909065

CLHY 0.643302

CLIA -0.331819

CLIC 0.630594

CLID -0.305247

CLIE -0.327276

CLIF 0.347269

CLIG -0.678029

CLIH 0.278598

CLII 0.134617

CLIK -0.257963

CLIL -0.221081

CLIM 0.574909

CLIN -0.137228

CLIP -0.251786

CLIQ -0.00414801

CLIR -0.0662019

CLIS -0.249972

CLIT -0.109879

CLIV -0.0808415

CLIW 0.746167

CLIY 0.397153

CLKA -0.581926

CLKC 0.13149

CLKD -0.187406

CLKE -0.193231

CLKF -0.0237558

CLKG -0.836087

CLKH -0.0658221

CLKI -0.274209

CLKK -0.313869

CLKL -0.511947

CLKM -0.0277295

CLKN -0.20394

CLKP -0.489986

CLKQ -0.0907025

CLKR -0.292886

CLKS -0.375984

CLKT -0.283848

CLKV -0.390389

CLKW 0.259877

CLKY 0.114461

CLLA -0.652308

CLLC 0.558795

CLLD -0.556484

CLLE -0.567165

CLLF 0.0433578

CLLG -0.896181

CLLH 0.142455

CLLI -0.23736

CLLK -0.498739

CLLL -0.494927

CLLM 0.342555

CLLN -0.387051

CLLP -0.449024

CLLQ -0.160739

CLLR -0.289368

CLLS -0.523643

CLLT -0.365109

CLLV -0.440779

CLLW 0.611119

CLLY 0.100329

CLMA 0.0885656

CLMC 0.795962

CLMD 0.0186274

CLME 0.0250862

CLMF 0.781986

CLMG -0.485595

CLMH 0.4248

CLMI 0.538899

CLMK 0.0554037

CLML 0.344193

CLMM 0.845287

CLMN 0.0710874

CLMP -0.104717

CLMQ 0.22928

CLMR 0.247154

CLMS 0.00246692

CLMT 0.169177

CLMV 0.385664

CLMW 0.82923

CLMY 0.733647

CLNA -0.440891

CLNC 0.268906

CLND -0.124228

CLNE -0.236048

CLNF 0.123428

CLNG -0.568425

CLNH 0.195514

CLNI -0.170141

CLNK -0.130726

CLNL -0.369652

CLNM 0.0830894

CLNN 0.0983844

CLNP -0.249332

CLNQ 0.0692153

CLNR -0.0216568

CLNS -0.149072

CLNT -0.0922964

CLNV -0.272883

CLNW 0.541649

CLNY 0.296125

CLPA -0.434534

CLPC 0.219562

CLPD -0.209308

CLPE -0.195499

CLPF 0.165867

CLPG -0.593543

CLPH 0.161309

CLPI -0.155754

CLPK -0.261395

CLPL -0.323852

CLPM 0.145706

CLPN -0.110785

CLPP -0.270069

CLPQ 0.00588965

CLPR -0.0487728

CLPS -0.197184

CLPT -0.142213

CLPV -0.191963

CLPW 0.592894

CLPY 0.261434

CLQA -0.277382

CLQC 0.426256

CLQD -0.144396

CLQE -0.133284

CLQF 0.284938

CLQG -0.526458

CLQH 0.165094

CLQI -0.0346045

CLQK -0.0613904

CLQL -0.178749

CLQM 0.183002

CLQN 0.0427175

CLQP -0.261969

CLQQ 0.260028

CLQR 0.0815418

CLQS -0.0944493

CLQT -0.0153196

CLQV -0.133802

CLQW 0.568688

CLQY 0.386403

CLRA -0.407546

CLRC 0.325199

CLRD -0.0219927

CLRE -0.0441856

CLRF 0.209303

CLRG -0.666526

CLRH 0.224756

CLRI -0.0660326

CLRK -0.287883

CLRL -0.282769

CLRM 0.273956

CLRN -0.0823529

CLRP -0.239284

CLRQ 0.0888898

CLRR 0.013463

CLRS -0.187383

CLRT -0.155418

CLRV -0.207691

CLRW 0.525791

CLRY 0.311282

CLSA -0.588171

CLSC 0.315203

CLSD -0.297768

CLSE -0.352573

CLSF 0.0374479

CLSG -0.634356

CLSH 0.131352

CLSI -0.280412

CLSK -0.312867

CLSL -0.456987

CLSM 0.050194

CLSN -0.130745

CLSP -0.432884

CLSQ -0.0210474

CLSR -0.14146

CLSS -0.215039

CLST -0.182087

CLSV -0.397934

CLSW 0.378308

CLSY 0.136743

CLTA -0.490122

CLTC 0.41075

CLTD -0.247283

CLTE -0.270985

CLTF 0.143801

CLTG -0.623343

CLTH 0.189761

CLTI -0.129936

CLTK -0.264437

CLTL -0.38376

CLTM 0.203988

CLTN -0.0773652

CLTP -0.30599

CLTQ 0.0376461

CLTR -0.0883546

CLTS -0.207959

CLTT -0.109376

CLTV -0.255756

CLTW 0.491636

CLTY 0.2348

CLVA -0.492443

CLVC 0.558733

CLVD -0.435782

CLVE -0.460435

CLVF 0.183748

CLVG -0.79647

CLVH 0.165234

CLVI -0.0765305

CLVK -0.383754

CLVL -0.377726

CLVM 0.405482

CLVN -0.27928

CLVP -0.36697

CLVQ -0.125446

CLVR -0.222418

CLVS -0.396975

CLVT -0.264945

CLVV -0.255415

CLVW 0.628304

CLVY 0.198505

CLWA 0.247743

CLWC 1.11193

CLWD 0.298048

CLWE 0.272851

CLWF 1.03966

CLWG -0.00182366

CLWH 0.714216

CLWI 0.784866

CLWK 0.307249

CLWL 0.627351

CLWM 0.919227

CLWN 0.478218

CLWP 0.321285

CLWQ 0.583343

CLWR 0.581866

CLWS 0.311341

CLWT 0.396075

CLWV 0.659656

CLWW 1.26685

CLWY 1.12869

CLYA -0.094305

CLYC 1.03758

CLYD 0.126789

CLYE 0.0133262

CLYF 0.657315

CLYG -0.251936

CLYH 0.601748

CLYI 0.343267

CLYK 0.103673

CLYL 0.0789614

CLYM 0.701801

CLYN 0.272735

CLYP 0.160923

CLYQ 0.372713

CLYR 0.315596

CLYS 0.0858622

CLYT 0.184616

CLYV 0.17195

CLYW 1.08332

CLYY 0.727926

CMAA -0.450181

CMAC 0.212251

CMAD -0.42587

CMAE -0.405396

CMAF 0.12983

CMAG -0.881661

CMAH 0.0493896

CMAI -0.111982

CMAK -0.338759

CMAL -0.38293

CMAM 0.250758

CMAN -0.287092

CMAP -0.595033

CMAQ -0.14069

CMAR -0.182734

CMAS -0.399287

CMAT -0.290172

CMAV -0.256564

CMAW 0.386966

CMAY 0.166269

CMCA 0.277451

CMCC 2.37265

CMCD 0.047982

CMCE -0.006253

CMCF 0.886586

CMCG -0.0609996

CMCH 0.708936

CMCI 0.719549

CMCK 0.213313

CMCL 0.842794

CMCM 1.63736

CMCN 0.261399

CMCP 0.133284

CMCQ 0.438443

CMCR 0.434618

CMCS 0.295317

CMCT 0.279906

CMCV 0.745039

CMCW 1.08439

CMCY 0.895953

CMDA -0.345838

CMDC 0.0671976

CMDD -0.165276

CMDE -0.195324

CMDF 0.135065

CMDG -0.544593

CMDH 0.256679

CMDI -0.132712

CMDK 0.0757411

CMDL -0.326791

CMDM 0.00062871

CMDN 0.103487

CMDP -0.332297

CMDQ 0.0281441

CMDR 0.218341

CMDS -0.0756025

CMDT -0.021131

CMDV -0.216021

CMDW 0.379661

CMDY 0.329766

CMEA -0.409189

CMEC 0.0074904

CMED -0.300893

CMEE -0.221596

CMEF 0.072175

CMEG -0.817662

CMEH 0.168409

CMEI -0.159617

CMEK 0.0465052

CMEL -0.334278

CMEM 0.0458868

CMEN -0.0917623

CMEP -0.472891

CMEQ 0.0231516

CMER 0.16963

CMES -0.296232

CMET -0.183344

CMEV -0.268611

CMEW 0.408906

CMEY 0.196126

CMFA 0.0937438

CMFC 0.897742

CMFD 0.167768

CMFE 0.0771637

CMFF 0.858667

CMFG -0.285377

CMFH 0.674328

CMFI 0.564988

CMFK 0.156295

CMFL 0.297757

CMFM 0.898666

CMFN 0.243123

CMFP 0.135463

CMFQ 0.366274

CMFR 0.331944

CMFS 0.207408

CMFT 0.289982

CMFV 0.396568

CMFW 1.09261

CMFY 0.828805

CMGA -0.872421

CMGC -0.190103

CMGD -0.515412

CMGE -0.656195

CMGF -0.1915

CMGG -1.79913

CMGH -0.353315

CMGI -0.471509

CMGK -0.498513

CMGL -0.63627

CMGM -0.297223

CMGN -0.412023

CMGP -0.810194

CMGQ -0.407152

CMGR -0.345272

CMGS -0.524725

CMGT -0.389129

CMGV -0.538599

CMGW -0.0506766

CMGY -0.0842671

CMHA -0.0356715

CMHC 0.634751

CMHD 0.316558

CMHE 0.185533

CMHF 0.672427

CMHG -0.362391

CMHH 0.726482

CMHI 0.320107

CMHK 0.00112772

CMHL 0.300365

CMHM 0.523081

CMHN 0.21652

CMHP 0.01105

CMHQ 0.193219

CMHR 0.309338

CMHS 0.196761

CMHT 0.268515

CMHV 0.278444

CMHW 0.901717

CMHY 0.725306

CMIA -0.100128

CMIC 0.713717

CMID -0.13481

CMIE -0.146348

CMIF 0.583194

CMIG -0.536944

CMIH 0.376497

CMII 0.378983

CMIK -0.0746274

CMIL 0.0403459

CMIM 0.718967

CMIN 0.00969744

CMIP -0.121412

CMIQ 0.134614

CMIR 0.114444

CMIS -0.065346

CMIT 0.0965407

CMIV 0.179379

CMIW 0.84389

CMIY 0.610028

CMKA -0.3877

CMKC 0.14525

CMKD 0.0202496

CMKE 0.0353372

CMKF 0.136202

CMKG -0.715831

CMKH -0.0192857

CMKI -0.0925179

CMKK -0.130394

CMKL -0.294589

CMKM 0.0169787

CMKN -0.0579214

CMKP -0.395104

CMKQ 0.0428505

CMKR -0.146481

CMKS -0.206261

CMKT -0.109308

CMKV -0.196905

CMKW 0.287172

CMKY 0.281779

CMLA -0.385303

CMLC 0.716188

CMLD -0.352035

CMLE -0.33573

CMLF 0.302359

CMLG -0.709265

CMLH 0.303574

CMLI 0.0230401

CMLK -0.269026

CMLL -0.315098

CMLM 0.537486

CMLN -0.19663

CMLP -0.267766

CMLQ 0.0418456

CMLR -0.0571117

CMLS -0.300784

CMLT -0.181689

CMLV -0.161368

CMLW 0.775794

CMLY 0.3413

CMMA 0.209201

CMMC 0.778078

CMMD 0.067796

CMME 0.0929911

CMMF 0.898807

CMMG -0.474378

CMMH 0.409954

CMMI 0.678188

CMMK 0.112902

CMML 0.531204

CMMM 0.871376

CMMN 0.0930176

CMMP -0.104813

CMMQ 0.245026

CMMR 0.303457

CMMS 0.0541556

CMMT 0.240427

CMMV 0.542194

CMMW 0.811391

CMMY 0.821225

CMNA -0.279193

CMNC 0.26584

CMND 0.0361826

CMNE -0.0803983

CMNF 0.25616

CMNG -0.45212

CMNH 0.237963

CMNI -0.0280633

CMNK 0.0304537

CMNL -0.180021

CMNM 0.102511

CMNN 0.247852

CMNP -0.16352

CMNQ 0.177671

CMNR 0.121897

CMNS 0.00956035

CMNT 0.0640743

CMNV -0.114407

CMNW 0.568306

CMNY 0.440041

CMPA -0.264084

CMPC 0.21458

CMPD -0.0611005

CMPE -0.0244603

CMPF 0.311238

CMPG -0.476452

CMPH 0.200919

CMPI -0.00533938

CMPK -0.121233

CMPL -0.132953

CMPM 0.176643

CMPN 0.00138021

CMPP -0.183308

CMPQ 0.107154

CMPR 0.0940094

CMPS -0.0432906

CMPT 0.0105224

CMPV 0.00032258

CMPW 0.63008

CMPY 0.402332

CMQA -0.116207

CMQC 0.421805

CMQD -0.0239556

CMQE 0.00930572

CMQF 0.40959

CMQG -0.436175

CMQH 0.179407

CMQI 0.0978808

CMQK 0.0776191

CMQL 0.0125101

CMQM 0.194273

CMQN 0.145956

CMQP -0.208257

CMQQ 0.372185

CMQR 0.209215

CMQS 0.039324

CMQT 0.122705

CMQV 0.0278277

CMQW 0.576941

CMQY 0.514817

CMRA -0.220006

CMRC 0.338166

CMRD 0.177853

CMRE 0.172293

CMRF 0.37668

CMRG -0.552463

CMRH 0.286276

CMRI 0.113046

CMRK -0.144292

CMRL -0.0810447

CMRM 0.336322

CMRN 0.0485656

CMRP -0.135904

CMRQ 0.218731

CMRR 0.182014

CMRS -0.02001

CMRT 0.00733423

CMRV -0.0016892

CMRW 0.562376

CMRY 0.477055

CMSA -0.398472

CMSC 0.354825

CMSD -0.112062

CMSE -0.156351

CMSF 0.212338

CMSG -0.471983

CMSH 0.211244

CMSI -0.106037

CMSK -0.126531

CMSL -0.275485

CMSM 0.112643

CMSN 0.0303657

CMSP -0.327903

CMSQ 0.127104

CMSR 0.0375576

CMSS -0.00934267

CMST 0.0206208

CMSV -0.193955

CMSW 0.423971

CMSY 0.309653

CMTA -0.284715

CMTC 0.455706

CMTD -0.06424

CMTE -0.073957

CMTF 0.324299

CMTG -0.470913

CMTH 0.27108

CMTI 0.0670664

CMTK -0.0789702

CMTL -0.1716

CMTM 0.279176

CMTN 0.079118

CMTP -0.187439

CMTQ 0.18734

CMTR 0.09021

CMTS -0.0127907

CMTT 0.0930977

CMTV -0.0275109

CMTW 0.546693

CMTY 0.414208

CMVA -0.248601

CMVC 0.666514

CMVD -0.24471

CMVE -0.256841

CMVF 0.41486

CMVG -0.635592

CMVH 0.285493

CMVI 0.164885

CMVK -0.176993

CMVL -0.163075

CMVM 0.573319

CMVN -0.115252

CMVP -0.216095

CMVQ 0.0365171

CMVR -0.0238178

CMVS -0.191972

CMVT -0.0427196

CMVV 0.0111485

CMVW 0.748965

CMVY 0.421149

CMWA 0.324056

CMWC 1.08791

CMWD 0.327869

CMWE 0.314898

CMWF 1.12811

CMWG 0.019048

CMWH 0.692334

CMWI 0.892487

CMWK 0.3411

CMWL 0.787069

CMWM 0.909992

CMWN 0.496595

CMWP 0.32401

CMWQ 0.593163

CMWR 0.626604

CMWS 0.346858

CMWT 0.435322

CMWV 0.78754

CMWW 1.25119

CMWY 1.21565

CMYA 0.0975466

CMYC 1.11283

CMYD 0.296813

CMYE 0.169515

CMYF 0.854991

CMYG -0.115925

CMYH 0.672148

CMYI 0.54256

CMYK 0.269844

CMYL 0.305896

CMYM 0.800722

CMYN 0.413121

CMYP 0.284644

CMYQ 0.497406

CMYR 0.477309

CMYS 0.244574

CMYT 0.356708

CMYV 0.39408

CMYW 1.16252

CMYY 0.91952

CNAA -0.463657

CNAC 0.224581

CNAD -0.433582

CNAE -0.41797

CNAF 0.117948

CNAG -0.879953

CNAH 0.055979

CNAI -0.1158

CNAK -0.344795

CNAL -0.400139

CNAM 0.242979

CNAN -0.265723

CNAP -0.590405

CNAQ -0.146211

CNAR -0.191615

CNAS -0.399737

CNAT -0.303867

CNAV -0.268117

CNAW 0.396543

CNAY 0.155153

CNCA 0.29309

CNCC 2.37375

CNCD 0.087148

CNCE 0.0156248

CNCF 0.885308

CNCG -0.0424764

CNCH 0.732194

CNCI 0.633871

CNCK 0.246613

CNCL 0.612395

CNCM 0.718369

CNCN 2.20771

CNCP 0.154438

CNCQ 0.521009

CNCR 0.451353

CNCS 0.338671

CNCT 0.297907

CNCV 0.589273

CNCW 1.10398

CNCY 0.916141

CNDA -0.356278

CNDC 0.0866611

CNDD -0.170388

CNDE -0.202998

CNDF 0.132387

CNDG -0.545729

CNDH 0.266076

CNDI -0.137647

CNDK 0.0614064

CNDL -0.340147

CNDM 0.0152361

CNDN 0.0975468

CNDP -0.325726

CNDQ 0.0300171

CNDR 0.206289

CNDS -0.0808258

CNDT -0.029994

CNDV -0.22593

CNDW 0.395751

CNDY 0.326588

CNEA -0.42191

CNEC 0.0267446

CNED -0.30513

CNEE -0.231781

CNEF 0.0671253

CNEG -0.813014

CNEH 0.177801

CNEI -0.166628

CNEK 0.0294683

CNEL -0.355727

CNEM 0.0591848

CNEN -0.0941136

CNEP -0.465357

CNEQ 0.022167

CNER 0.155665

CNES -0.301088

CNET -0.190159

CNEV -0.278537

CNEW 0.422818

CNEY 0.192398

CNFA 0.0815902

CNFC 0.911025

CNFD 0.167036

CNFE 0.0728788

CNFF 0.852313

CNFG -0.282319

CNFH 0.682562

CNFI 0.55081

CNFK 0.151748

CNFL 0.274326

CNFM 0.901534

CNFN 0.249642

CNFP 0.13965

CNFQ 0.369848

CNFR 0.329246

CNFS 0.200559

CNFT 0.283709

CNFV 0.386474

CNFW 1.10205

CNFY 0.819354

CNGA -0.873368

CNGC -0.170514

CNGD -0.518184

CNGE -0.656831

CNGF -0.191117

CNGG -1.77945

CNGH -0.336393

CNGI -0.474029

CNGK -0.501481

CNGL -0.646075

CNGM -0.281896

CNGN -0.407981

CNGP -0.797712

CNGQ -0.39966

CNGR -0.34656

CNGS -0.525109

CNGT -0.394315

CNGV -0.544671

CNGW -0.031707

CNGY -0.0831585

CNHA -0.028271

CNHC 0.655025

CNHD 0.325156

CNHE 0.19291

CNHF 0.681126

CNHG -0.343893

CNHH 0.745605

CNHI 0.328619

CNHK 0.0167561

CNHL 0.297477

CNHM 0.542714

CNHN 0.23715

CNHP 0.0292704

CNHQ 0.211419

CNHR 0.321843

CNHS 0.207686

CNHT 0.278587

CNHV 0.283875

CNHW 0.921775

CNHY 0.73504

CNIA -0.11671

CNIC 0.72342

CNID -0.140027

CNIE -0.154548

CNIF 0.564063

CNIG -0.537074

CNIH 0.383165

CNII 0.359596

CNIK -0.0773988

CNIL 0.012934

CNIM 0.720743

CNIN 0.0169454

CNIP -0.119241

CNIQ 0.134325

CNIR 0.105763

CNIS -0.0740278

CNIT 0.0845973

CNIV 0.158254

CNIW 0.850902

CNIY 0.596216

CNKA -0.396905

CNKC 0.163901

CNKD 0.00656056

CNKE 0.0198328

CNKF 0.133213

CNKG -0.712257

CNKH -0.00420427

CNKI -0.0989618

CNKK -0.138189

CNKL -0.306933

CNKM 0.0321693

CNKN -0.0567889

CNKP -0.387182

CNKQ 0.0433388

CNKR -0.147186

CNKS -0.212806

CNKT -0.11614

CNKV -0.207217

CNKW 0.304421

CNKY 0.278655

CNLA -0.409473

CNLC 0.714528

CNLD -0.362735

CNLE -0.355133

CNLF 0.28135

CNLG -0.718088

CNLH 0.303152

CNLI 0.00555634

CNLK -0.286985

CNLL -0.332535

CNLM 0.525852

CNLN -0.203385

CNLP -0.274418

CNLQ 0.0305731

CNLR -0.0775294

CNLS -0.312996

CNLT -0.192453

CNLV -0.182752

CNLW 0.774283

CNLY 0.32309

CNMA 0.212311

CNMC 0.798306

CNMD 0.0823853

CNME 0.105444

CNMF 0.902425

CNMG -0.455352

CNMH 0.430449

CNMI 0.680254

CNMK 0.130284

CNML 0.52731

CNMM 0.887205

CNMN 0.111375

CNMP -0.0851295

CNMQ 0.262982

CNMR 0.316826

CNMS 0.067446

CNMT 0.252241

CNMV 0.53972

CNMW 0.831635

CNMY 0.829961

CNNA -0.281904

CNNC 0.285823

CNND 0.0335953

CNNE -0.0831459

CNNF 0.257661

CNNG -0.448141

CNNH 0.253176

CNNI -0.0280967

CNNK 0.0263548

CNNL -0.189623

CNNM 0.121349

CNNN 0.258545

CNNP -0.154233

CNNQ 0.182662

CNNR 0.120836

CNNS 0.00685143

CNNT 0.0616462

CNNV -0.117452

CNNW 0.585712

CNNY 0.439776

CNPA -0.26926

CNPC 0.234669

CNPD -0.0627418

CNPE -0.0283341

CNPF 0.310708

CNPG -0.472576

CNPH 0.216444

CNPI -0.00707793

CNPK -0.121058

CNPL -0.139963

CNPM 0.193009

CNPN 0.00740504

CNPP -0.174275

CNPQ 0.113615

CNPR 0.0959272

CNPS -0.0432906

CNPT 0.00812531

CNPV -0.00503993

CNPW 0.646126

CNPY 0.402189

CNQA -0.119576

CNQC 0.441862

CNQD -0.0189712

CNQE 0.00908685

CNQF 0.412429

CNQG -0.427779

CNQH 0.197781

CNQI 0.0997005

CNQK 0.0779309

CNQL 0.00377536

CNQM 0.212938

CNQN 0.154414

CNQP -0.19432

CNQQ 0.377294

CNQR 0.21154

CNQS 0.040262

CNQT 0.123354

CNQV 0.0244455

CNQW 0.596075

CNQY 0.516729

CNRA -0.228611

CNRC 0.356929

CNRD 0.168086

CNRE 0.161619

CNRF 0.371441

CNRG -0.546829

CNRH 0.298979

CNRI 0.107339

CNRK -0.143702

CNRL -0.0935678

CNRM 0.348859

CNRN 0.0501664

CNRP -0.129476

CNRQ 0.220717

CNRR 0.176503

CNRS -0.0253401

CNRT 0.00359106

CNRV -0.0132964

CNRW 0.578533

CNRY 0.472751

CNSA -0.407309

CNSC 0.37045

CNSD -0.121084

CNSE -0.166695

CNSF 0.207692

CNSG -0.474796

CNSH 0.220819

CNSI -0.108966

CNSK -0.134652

CNSL -0.285424

CNSM 0.124154

CNSN 0.0453641

CNSP -0.321826

CNSQ 0.125612

CNSR 0.0306492

CNSS -0.0254397

CNST 0.0108666

CNSV -0.204226

CNSW 0.439162

CNSY 0.303504

CNTA -0.295851

CNTC 0.470911

CNTD -0.0725393

CNTE -0.0830398

CNTF 0.316309

CNTG -0.473357

CNTH 0.282612

CNTI 0.0581324

CNTK -0.0876176

CNTL -0.184577

CNTM 0.287745

CNTN 0.0833018

CNTP -0.183542

CNTQ 0.184805

CNTR 0.0868368

CNTS -0.0224061

CNTT 0.0854926

CNTV -0.0426705

CNTW 0.560457

CNTY 0.407896

CNVA -0.268041

CNVC 0.6715

CNVD -0.253927

CNVE -0.269421

CNVF 0.398894

CNVG -0.639015

CNVH 0.290389

CNVI 0.150192

CNVK -0.191003

CNVL -0.169824

CNVM 0.565206

CNVN -0.107712

CNVP -0.217386

CNVQ 0.0306873

CNVR -0.0341523

CNVS -0.202273

CNVT -0.0561955

CNVV -0.00626326

CNVW 0.752611

CNVY 0.40602

CNWA 0.33641

CNWC 1.10827

CNWD 0.344929

CNWE 0.330414

CNWF 1.13662

CNWG 0.0368373

CNWH 0.712635

CNWI 0.897983

CNWK 0.357588

CNWL 0.783899

CNWM 0.929834

CNWN 0.514719

CNWP 0.34346

CNWQ 0.612036

CNWR 0.641645

CNWS 0.363684

CNWT 0.452468

CNWV 0.790181

CNWW 1.27159

CNWY 1.22495

CNYA 0.088902

CNYC 1.12378

CNYD 0.288545

CNYE 0.165343

CNYF 0.847376

CNYG -0.114984

CNYH 0.68325

CNYI 0.532239

CNYK 0.265483

CNYL 0.288533

CNYM 0.802481

CNYN 0.419838

CNYP 0.289737

CNYQ 0.499839

CNYR 0.473313

CNYS 0.239927

CNYT 0.352073

CNYV 0.380327

CNYW 1.17258

CNYY 0.910891

CPAA -0.45941

CPAC 0.220678

CPAD -0.430381

CPAE -0.413004

CPAF 0.121871

CPAG -0.876444

CPAH 0.0526197

CPAI -0.111485

CPAK -0.338936

CPAL -0.381849

CPAM 0.24284

CPAN -0.288712

CPAP -0.564538

CPAQ -0.142529

CPAR -0.186202

CPAS -0.404719

CPAT -0.295511

CPAV -0.267825

CPAW 0.393003

CPAY 0.158881

CPCA 0.296045

CPCC 2.37384

CPCD 0.0610349

CPCE 0.00532174

CPCF 0.885604

CPCG -0.0490367

CPCH 0.721983

CPCI 0.628163

CPCK 0.229938

CPCL 0.607402

CPCM 0.709145

CPCN 0.273328

CPCP 1.7545

CPCQ 0.452871

CPCR 0.444766

CPCS 0.321666

CPCT 0.28987

CPCV 0.636044

CPCW 1.09625

CPCY 0.917957

CPDA -0.351705

CPDC 0.0795028

CPDD -0.16819

CPDE -0.199415

CPDF 0.1367

CPDG -0.544986

CPDH 0.263053

CPDI -0.136549

CPDK 0.0673282

CPDL -0.33485

CPDM 0.00974917

CPDN 0.100535

CPDP -0.326384

CPDQ 0.0299337

CPDR 0.211437

CPDS -0.0806851

CPDT -0.0258632

CPDV -0.222066

CPDW 0.390112

CPDY 0.326597

CPEA -0.418092

CPEC 0.0196698

CPED -0.302625

CPEE -0.227351

CPEF 0.0714869

CPEG -0.808465

CPEH 0.174517

CPEI -0.161876

CPEK 0.0351083

CPEL -0.350416

CPEM 0.0547292

CPEN -0.092695

CPEP -0.464779

CPEQ 0.0235507

CPER 0.161549

CPES -0.29914

CPET -0.182122

CPEV -0.273972

CPEW 0.417961

CPEY 0.19448

CPFA 0.0865417

CPFC 0.906433

CPFD 0.16639

CPFE 0.0747724

CPFF 0.851621

CPFG -0.282906

CPFH 0.680238

CPFI 0.556687

CPFK 0.156298

CPFL 0.28357

CPFM 0.9002

CPFN 0.245414

CPFP 0.145134

CPFQ 0.370944

CPFR 0.330544

CPFS 0.203539

CPFT 0.286558

CPFV 0.386919

CPFW 1.09904

CPFY 0.82355

CPGA -0.87269

CPGC -0.177794

CPGD -0.517038

CPGE -0.655308

CPGF -0.188185

CPGG -1.78673

CPGH -0.342348

CPGI -0.472912

CPGK -0.497406

CPGL -0.643554

CPGM -0.287664

CPGN -0.409357

CPGP -0.798839

CPGQ -0.401936

CPGR -0.345632

CPGS -0.525836

CPGT -0.391427

CPGV -0.541475

CPGW -0.0388906

CPGY -0.0847397

CPHA -0.0301487

CPHC 0.647195

CPHD 0.321812

CPHE 0.191303

CPHF 0.678801

CPHG -0.35153

CPHH 0.738325

CPHI 0.32591

CPHK 0.0105696

CPHL 0.298945

CPHM 0.535229

CPHN 0.226701

CPHP 0.0257833

CPHQ 0.204938

CPHR 0.317619

CPHS 0.204024

CPHT 0.275217

CPHV 0.282252

CPHW 0.914288

CPHY 0.731999

CPIA -0.109921

CPIC 0.720234

CPID -0.137559

CPIE -0.14589

CPIF 0.569463

CPIG -0.536659

CPIH 0.381103

CPII 0.367633

CPIK -0.0780449

CPIL 0.0238078

CPIM 0.717864

CPIN 0.0111947

CPIP -0.118649

CPIQ 0.135303

CPIR 0.109514

CPIS -0.0685232

CPIT 0.0908105

CPIV 0.164285

CPIW 0.848901

CPIY 0.602117

CPKA -0.392899

CPKC 0.157109

CPKD 0.0129607

CPKE 0.0265415

CPKF 0.134887

CPKG -0.712981

CPKH -0.00963545

CPKI -0.0950971

CPKK -0.134567

CPKL -0.303647

CPKM 0.0269198

CPKN -0.0578723

CPKP -0.392706

CPKQ 0.0442605

CPKR -0.146425

CPKS -0.209171

CPKT -0.112089

CPKV -0.202341

CPKW 0.298249

CPKY 0.279714

CPLA -0.403784

CPLC 0.715718

CPLD -0.358152

CPLE -0.349244

CPLF 0.294321

CPLG -0.714061

CPLH 0.303662

CPLI 0.0099194

CPLK -0.278846

CPLL -0.318519

CPLM 0.529547

CPLN -0.201514

CPLP -0.265158

CPLQ 0.0354545

CPLR -0.070816

CPLS -0.304363

CPLT -0.186317

CPLV -0.131142

CPLW 0.774999

CPLY 0.329866

CPMA 0.211571

CPMC 0.790592

CPMD 0.077307

CPME 0.100976

CPMF 0.901315

CPMG -0.462499

CPMH 0.422553

CPMI 0.680021

CPMK 0.121746

CPML 0.525738

CPMM 0.881342

CPMN 0.102723

CPMP -0.0916374

CPMQ 0.256682

CPMR 0.312407

CPMS 0.0627139

CPMT 0.248022

CPMV 0.540535

CPMW 0.823927

CPMY 0.827027

CPNA -0.281045

CPNC 0.278373

CPND 0.0347035

CPNE -0.0815108

CPNF 0.25769

CPNG -0.449059

CPNH 0.2495

CPNI -0.0280747

CPNK 0.0315762

CPNL -0.184437

CPNM 0.11367

CPNN 0.247553

CPNP -0.154728

CPNQ 0.181462

CPNR 0.122107

CPNS 0.00831294

CPNT 0.06302

CPNV -0.115381

CPNW 0.579389

CPNY 0.440393

CPPA -0.267343

CPPC 0.227165

CPPD -0.061008

CPPE -0.0270529

CPPF 0.311535

CPPG -0.473551

CPPH 0.211047

CPPI -0.00552392

CPPK -0.12033

CPPL -0.138298

CPPM 0.187332

CPPN 0.00500941

CPPP -0.176013

CPPQ 0.111936

CPPR 0.0945573

CPPS -0.0448542

CPPT 0.00957298

CPPV -0.00442767

CPPW 0.640477

CPPY 0.402858

CPQA -0.11716

CPQC 0.434376

CPQD -0.0211575

CPQE 0.00992084

CPQF 0.411863

CPQG -0.431155

CPQH 0.191094

CPQI 0.100101

CPQK 0.0787992

CPQL 0.00734925

CPQM 0.206127

CPQN 0.15057

CPQP -0.198773

CPQQ 0.375938

CPQR 0.211155

CPQS 0.0408475

CPQT 0.123185

CPQV 0.0258341

CPQW 0.589029

CPQY 0.516386

CPRA -0.225002

CPRC 0.350089

CPRD 0.172798

CPRE 0.163458

CPRF 0.373547

CPRG -0.549034

CPRH 0.294714

CPRI 0.111058

CPRK -0.144104

CPRL -0.0836797

CPRM 0.344413

CPRN 0.0501211

CPRP -0.129441

CPRQ 0.220622

CPRR 0.17955

CPRS -0.0226679

CPRT 0.00769663

CPRV -0.00943446

CPRW 0.572807

CPRY 0.475314

CPSA -0.401947

CPSC 0.364669

CPSD -0.116793

CPSE -0.159645

CPSF 0.209634

CPSG -0.47479

CPSH 0.217796

CPSI -0.106663

CPSK -0.130906

CPSL -0.280376

CPSM 0.119625

CPSN 0.0288384

CPSP -0.316586

CPSQ 0.126851

CPSR 0.0342607

CPSS -0.016685

CPST 0.0133739

CPSV -0.200525

CPSW 0.43388

CPSY 0.306482

CPTA -0.292732

CPTC 0.466627

CPTD -0.0683031

CPTE -0.0813179

CPTF 0.319665

CPTG -0.470805

CPTH 0.277873

CPTI 0.0625136

CPTK -0.0838864

CPTL -0.176577

CPTM 0.284118

CPTN 0.0791934

CPTP -0.166697

CPTQ 0.186665

CPTR 0.0881238

CPTS -0.0184269

CPTT 0.0860357

CPTV -0.0379679

CPTW 0.555773

CPTY 0.410794

CPVA -0.257821

CPVC 0.670275

CPVD -0.249773

CPVE -0.259653

CPVF 0.40938

CPVG -0.635084

CPVH 0.288493

CPVI 0.158563

CPVK -0.185086

CPVL -0.177289

CPVM 0.569418

CPVN -0.116365

CPVP -0.196766

CPVQ 0.0341406

CPVR -0.0293829

CPVS -0.199136

CPVT -0.0518205

CPVV 0.00713968

CPVW 0.751948

CPVY 0.412001

CPWA 0.331039

CPWC 1.10042

CPWD 0.33877

CPWE 0.325019

CPWF 1.13438

CPWG 0.0304749

CPWH 0.704846

CPWI 0.896672

CPWK 0.351759

CPWL 0.785375

CPWM 0.922249

CPWN 0.508052

CPWP 0.336991

CPWQ 0.605183

CPWR 0.636423

CPWS 0.357408

CPWT 0.445654

CPWV 0.78977

CPWW 1.26386

CPWY 1.22187

CPYA 0.0922585

CPYC 1.12044

CPYD 0.291296

CPYE 0.168021

CPYF 0.849423

CPYG -0.114725

CPYH 0.679826

CPYI 0.536891

CPYK 0.267927

CPYL 0.295395

CPYM 0.799558

CPYN 0.414097

CPYP 0.292434

CPYQ 0.499609

CPYR 0.475726

CPYS 0.24291

CPYT 0.355784

CPYV 0.385232

CPYW 1.16942

CPYY 0.913608

CQAA -0.471347

CQAC 0.224492

CQAD -0.433565

CQAE -0.4168

CQAF 0.117368

CQAG -0.882436

CQAH 0.0556262

CQAI -0.119721

CQAK -0.351523

CQAL -0.402256

CQAM 0.244817

CQAN -0.292982

CQAP -0.590179

CQAQ -0.135201

CQAR -0.190621

CQAS -0.412911

CQAT -0.304056

CQAV -0.280535

CQAW 0.396387

CQAY 0.15393

CQCA 0.412219

CQCC 2.37207

CQCD 0.0670965

CQCE 0.0420702

CQCF 0.880499

CQCG -0.0429575

CQCH 0.753689

CQCI 0.605345

CQCK 0.25219

CQCL 0.587535

CQCM 0.716963

CQCN 0.342559

CQCP 0.155531

CQCQ 2.3839

CQCR 0.473794

CQCS 0.380847

CQCT 0.326325

CQCV 0.826282

CQCW 1.10486

CQCY 0.903557

CQDA -0.357114

CQDC 0.087302

CQDD -0.17143

CQDE -0.202867

CQDF 0.132215

CQDG -0.545748

CQDH 0.265845

CQDI -0.136112

CQDK 0.0617454

CQDL -0.340538

CQDM 0.015722

CQDN 0.0980642

CQDP -0.325905

CQDQ 0.030957

CQDR 0.206595

CQDS -0.0859003

CQDT -0.0293965

CQDV -0.22708

CQDW 0.396281

CQDY 0.323661

CQEA -0.423145

CQEC 0.0273411

CQED -0.305216

CQEE -0.231319

CQEF 0.0678396

CQEG -0.813536

CQEH 0.177479

CQEI -0.167098

CQEK 0.0297973

CQEL -0.35659

CQEM 0.0594618

CQEN -0.0944107

CQEP -0.463448

CQEQ 0.0253813

CQER 0.156052

CQES -0.30152

CQET -0.19031

CQEV -0.278596

CQEW 0.423189

CQEY 0.192206

CQFA 0.0816936

CQFC 0.911546

CQFD 0.164402

CQFE 0.0717158

CQFF 0.845354

CQFG -0.28277

CQFH 0.684995

CQFI 0.549158

CQFK 0.155563

CQFL 0.27442

CQFM 0.902029

CQFN 0.245257

CQFP 0.140058

CQFQ 0.370523

CQFR 0.329454

CQFS 0.201385

CQFT 0.283859

CQFV 0.379819

CQFW 1.1023

CQFY 0.819416

CQGA -0.874282

CQGC -0.169557

CQGD -0.518993

CQGE -0.65678

CQGF -0.190276

CQGG -1.77876

CQGH -0.336072

CQGI -0.475003

CQGK -0.49993

CQGL -0.644046

CQGM -0.280627

CQGN -0.409101

CQGP -0.797408

CQGQ -0.39834

CQGR -0.346725

CQGS -0.527885

CQGT -0.391877

CQGV -0.545355

CQGW -0.0315344

CQGY -0.0852976

CQHA -0.0240481

CQHC 0.655839

CQHD 0.324474

CQHE 0.193921

CQHF 0.68144

CQHG -0.345278

CQHH 0.745739

CQHI 0.328974

CQHK 0.0155611

CQHL 0.297479

CQHM 0.543448

CQHN 0.231943

CQHP 0.0300615

CQHQ 0.21417

CQHR 0.325115

CQHS 0.207382

CQHT 0.278409

CQHV 0.28249

CQHW 0.922869

CQHY 0.735432

CQIA -0.116166

CQIC 0.723629

CQID -0.140488

CQIE -0.151208

CQIF 0.563231

CQIG -0.53661

CQIH 0.383969

CQII 0.358867

CQIK -0.0821857

CQIL 0.0138443

CQIM 0.716879

CQIN 0.00897741

CQIP -0.118777

CQIQ 0.136443

CQIR 0.106527

CQIS -0.0708129

CQIT 0.0840819

CQIV 0.155232

CQIW 0.851354

CQIY 0.595402

CQKA -0.398042

CQKC 0.164505

CQKD 0.0101016

CQKE 0.0185869

CQKF 0.132824

CQKG -0.71267

CQKH -0.00429964

CQKI -0.0997524

CQKK -0.138727

CQKL -0.309397

CQKM 0.03262

CQKN -0.0571995

CQKP -0.387156

CQKQ 0.0470023

CQKR -0.147757

CQKS -0.209106

CQKT -0.115519

CQKV -0.20478

CQKW 0.304914

CQKY 0.277149

CQLA -0.410728

CQLC 0.712887

CQLD -0.363766

CQLE -0.357357

CQLF 0.284525

CQLG -0.717699

CQLH 0.299186

CQLI 0.00452781

CQLK -0.280785

CQLL -0.32892

CQLM 0.525025

CQLN -0.199568

CQLP -0.275444

CQLQ 0.0339353

CQLR -0.0794683

CQLS -0.318042

CQLT -0.194087

CQLV -0.186207

CQLW 0.770948

CQLY 0.322173

CQMA 0.212101

CQMC 0.799206

CQMD 0.0828488

CQME 0.105091

CQMF 0.902075

CQMG -0.454917

CQMH 0.430875

CQMI 0.679912

CQMK 0.126966

CQML 0.522382

CQMM 0.889257

CQMN 0.109508

CQMP -0.0845058

CQMQ 0.265752

CQMR 0.317079

CQMS 0.0679729

CQMT 0.252244

CQMV 0.539229

CQMW 0.832469

CQMY 0.830883

CQNA -0.279249

CQNC 0.286528

CQND 0.0331562

CQNE -0.0833838

CQNF 0.258722

CQNG -0.448032

CQNH 0.253495

CQNI -0.0290651

CQNK 0.0267277

CQNL -0.186087

CQNM 0.12068

CQNN 0.244749

CQNP -0.153971

CQNQ 0.184614

CQNR 0.123374

CQNS 0.00598216

CQNT 0.0605195

CQNV -0.118536

CQNW 0.585977

CQNY 0.439482

CQPA -0.268244

CQPC 0.235345

CQPD -0.0623431

CQPE -0.0303159

CQPF 0.310601

CQPG -0.472371

CQPH 0.216802

CQPI -0.00659299

CQPK -0.121099

CQPL -0.143479

CQPM 0.193457

CQPN 0.00614047

CQPP -0.174165

CQPQ 0.115175

CQPR 0.0933657

CQPS -0.0460596

CQPT 0.00819731

CQPV -0.0097611

CQPW 0.646642

CQPY 0.402216

CQQA -0.120236

CQQC 0.442663

CQQD -0.0206482

CQQE 0.00905252

CQQF 0.412207

CQQG -0.427521

CQQH 0.198575

CQQI 0.0999117

CQQK 0.0778661

CQQL 0.00350738

CQQM 0.213789

CQQN 0.152156

CQQP -0.193931

CQQQ 0.376814

CQQR 0.211039

CQQS 0.0408418

CQQT 0.123222

CQQV 0.0239477

CQQW 0.596714

CQQY 0.516882

CQRA -0.229303

CQRC 0.357522

CQRD 0.166694

CQRE 0.15697

CQRF 0.371317

CQRG -0.546202

CQRH 0.299468

CQRI 0.10727

CQRK -0.142417

CQRL -0.0945072

CQRM 0.34916

CQRN 0.0498965

CQRP -0.129392

CQRQ 0.219869

CQRR 0.18065

CQRS -0.0257268

CQRT 0.00236607

CQRV -0.0128076

CQRW 0.579041

CQRY 0.472892

CQSA -0.408623

CQSC 0.370789

CQSD -0.121406

CQSE -0.168478

CQSF 0.206771

CQSG -0.47664

CQSH 0.220714

CQSI -0.106956

CQSK -0.134956

CQSL -0.286653

CQSM 0.124498

CQSN 0.0246456

CQSP -0.321716

CQSQ 0.134137

CQSR 0.0295753

CQSS -0.0203552

CQST 0.00796556

CQSV -0.206279

CQSW 0.439585

CQSY 0.303699

CQTA -0.297213

CQTC 0.471357

CQTD -0.0711584

CQTE -0.0843678

CQTF 0.316723

CQTG -0.473256

CQTH 0.280497

CQTI 0.0575068

CQTK -0.0871751

CQTL -0.184862

CQTM 0.287813

CQTN 0.0762532

CQTP -0.183805

CQTQ 0.187915

CQTR 0.08248

CQTS -0.0235314

CQTT 0.0836005

CQTV -0.0472548

CQTW 0.560929

CQTY 0.407527

CQVA -0.276531

CQVC 0.671396

CQVD -0.253855

CQVE -0.267047

CQVF 0.397539

CQVG -0.640018

CQVH 0.28925

CQVI 0.146379

CQVK -0.19248

CQVL -0.177231

CQVM 0.56163

CQVN -0.122489

CQVP -0.218184

CQVQ 0.0410242

CQVR -0.0338757

CQVS -0.206

CQVT -0.0585167

CQVV -0.011292

CQVW 0.752807

CQVY 0.406669

CQWA 0.334507

CQWC 1.10908

CQWD 0.345447

CQWE 0.33051

CQWF 1.13725

CQWG 0.0377443

CQWH 0.7134

CQWI 0.898261

CQWK 0.358002

CQWL 0.783166

CQWM 0.930605

CQWN 0.515275

CQWP 0.344211

CQWQ 0.613304

CQWR 0.641728

CQWS 0.363507

CQWT 0.451575

CQWV 0.789884

CQWW 1.27239

CQWY 1.22494

CQYA 0.0873518

CQYC 1.12398

CQYD 0.288714

CQYE 0.168633

CQYF 0.844415

CQYG -0.114986

CQYH 0.683606

CQYI 0.532134

CQYK 0.266409

CQYL 0.288484

CQYM 0.802763

CQYN 0.412835

CQYP 0.287454

CQYQ 0.501513

CQYR 0.472477

CQYS 0.239998

CQYT 0.350916

CQYV 0.377653

CQYW 1.17283

CQYY 0.910027

CRAA -0.496641

CRAC 0.231095

CRAD -0.449077

CRAE -0.437972

CRAF 0.0936604

CRAG -0.88933

CRAH 0.0527799

CRAI -0.147355

CRAK -0.365275

CRAL -0.431535

CRAM 0.242399

CRAN -0.302344

CRAP -0.590978

CRAQ -0.156406

CRAR -0.165055

CRAS -0.424707

CRAT -0.325216

CRAV -0.302754

CRAW 0.398475

CRAY 0.138721

CRCA 0.311681

CRCC 2.36508

CRCD 0.0893304

CRCE 0.0283668

CRCF 0.899409

CRCG -0.0536907

CRCH 0.756623

CRCI 0.640446

CRCK 0.434491

CRCL 0.607785

CRCM 0.739668

CRCN 0.299433

CRCP 0.173956

CRCQ 0.500324

CRCR 3.13497

CRCS 0.394764

CRCT 0.349966

CRCV 0.670933

CRCW 1.12502

CRCY 0.919428

CRDA -0.374825

CRDC 0.103436

CRDD -0.184787

CRDE -0.219596

CRDF 0.122547

CRDG -0.554316

CRDH 0.271173

CRDI -0.151246

CRDK 0.0389478

CRDL -0.361917

CRDM 0.0262516

CRDN 0.0844128

CRDP -0.326155

CRDQ 0.0243862

CRDR 0.190974

CRDS -0.0985579

CRDT -0.0475936

CRDV -0.243956

CRDW 0.407151

CRDY 0.310205

CREA -0.442264

CREC 0.0430987

CRED -0.317184

CREE -0.248849

CREF 0.0554943

CREG -0.81638

CREH 0.179356

CREI -0.18186

CREK 0.00190473

CREL -0.380015

CREM 0.06615

CREN -0.101417

CREP -0.464352

CREQ 0.0165408

CRER 0.141706

CRES -0.31411

CRET -0.203773

CREV -0.29798

CREW 0.431256

CREY 0.180058

CRFA 0.0610561

CRFC 0.918803

CRFD 0.152211

CRFE 0.0601578

CRFF 0.824587

CRFG -0.286498

CRFH 0.6849

CRFI 0.526978

CRFK 0.142736

CRFL 0.2435

CRFM 0.898595

CRFN 0.239741

CRFP 0.137448

CRFQ 0.365066

CRFR 0.325856

CRFS 0.187297

CRFT 0.27129

CRFV 0.356341

CRFW 1.10517

CRFY 0.802219

CRGA -0.882164

CRGC -0.153321

CRGD -0.528306

CRGE -0.66502

CRGF -0.198575

CRGG -1.76212

CRGH -0.32433

CRGI -0.481569

CRGK -0.505142

CRGL -0.664799

CRGM -0.268689

CRGN -0.413478

CRGP -0.791066

CRGQ -0.398973

CRGR -0.35516

CRGS -0.535285

CRGT -0.406533

CRGV -0.560169

CRGW -0.0165303

CRGY -0.0912032

CRHA -0.0259006

CRHC 0.675511

CRHD 0.325235

CRHE 0.196172

CRHF 0.686307

CRHG -0.33288

CRHH 0.758668

CRHI 0.330803

CRHK 0.024127

CRHL 0.286834

CRHM 0.561042

CRHN 0.24254

CRHP 0.0439818

CRHQ 0.226329

CRHR 0.329519

CRHS 0.213663

CRHT 0.282189

CRHV 0.279729

CRHW 0.94047

CRHY 0.74032

CRIA -0.141849

CRIC 0.726727

CRID -0.15043

CRIE -0.166541

CRIF 0.539581

CRIG -0.543951

CRIH 0.384224

CRII 0.330635

CRIK -0.0980415

CRIL -0.0189846

CRIM 0.708683

CRIN -0.00042486

CRIP -0.123497

CRIQ 0.127683

CRIR 0.115298

CRIS -0.0904257

CRIT 0.0700562

CRIV 0.125277

CRIW 0.851318

CRIY 0.57427

CRKA -0.415144

CRKC 0.179263

CRKD -0.0128872

CRKE -0.00376105

CRKF 0.124168

CRKG -0.715837

CRKH 0.00530624

CRKI -0.114474

CRKK -0.154474

CRKL -0.331034

CRKM 0.0424666

CRKN -0.0668211

CRKP -0.385258

CRKQ 0.0371876

CRKR -0.156656

CRKS -0.224973

CRKT -0.130508

CRKV -0.222053

CRKW 0.317503

CRKY 0.264271

CRLA -0.441313

CRLC 0.703459

CRLD -0.383422

CRLE -0.374505

CRLF 0.252108

CRLG -0.733604

CRLH 0.291008

CRLI -0.0296562

CRLK -0.315859

CRLL -0.361223

CRLM 0.509849

CRLN -0.21996

CRLP -0.29014

CRLQ 0.012871

CRLR -0.0579448

CRLS -0.339583

CRLT -0.220531

CRLV -0.205053

CRLW 0.761293

CRLY 0.294655

CRMA 0.208118

CRMC 0.818147

CRMD 0.0913122

CRME 0.109082

CRMF 0.901546

CRMG -0.440051

CRMH 0.449542

CRMI 0.672956

CRMK 0.133292

CRML 0.506479

CRMM 0.900829

CRMN 0.12496

CRMP -0.0677364

CRMQ 0.277596

CRMR 0.32545

CRMS 0.0772941

CRMT 0.25777

CRMV 0.528965

CRMW 0.851442

CRMY 0.83196

CRNA -0.2951

CRNC 0.303626

CRND 0.0218418

CRNE -0.0939882

CRNF 0.251785

CRNG -0.450918

CRNH 0.263641

CRNI -0.0366168

CRNK 0.0137849

CRNL -0.197186

CRNM 0.134636

CRNN 0.237257

CRNP -0.151469

CRNQ 0.184528

CRNR 0.125005

CRNS -0.00214696

CRNT 0.0508277

CRNV -0.130128

CRNW 0.598833

CRNY 0.431166

CRPA -0.279509

CRPC 0.252856

CRPD -0.0716152

CRPE -0.0430546

CRPF 0.302136

CRPG -0.475386

CRPH 0.227272

CRPI -0.0157621

CRPK -0.126698

CRPL -0.16231

CRPM 0.205499

CRPN 0.00407624

CRPP -0.17176

CRPQ 0.113513

CRPR 0.086

CRPS -0.0557232

CRPT -0.00150919

CRPV -0.025841

CRPW 0.657488

CRPY 0.394354

CRQA -0.131339

CRQC 0.459999

CRQD -0.0240195

CRQE 0.00063348

CRQF 0.406989

CRQG -0.425869

CRQH 0.2129

CRQI 0.0936956

CRQK 0.0735316

CRQL -0.0141513

CRQM 0.228763

CRQN 0.152056

CRQP -0.185886

CRQQ 0.375885

CRQR 0.209153

CRQS 0.0342634

CRQT 0.117129

CRQV 0.0122957

CRQW 0.612265

CRQY 0.511598

CRRA -0.244323

CRRC 0.372369

CRRD 0.148735

CRRE 0.13755

CRRF 0.363142

CRRG -0.550541

CRRH 0.305857

CRRI 0.0920594

CRRK -0.154892

CRRL -0.118012

CRRM 0.356013

CRRN 0.0444353

CRRP -0.129857

CRRQ 0.21616

CRRR 0.169135

CRRS -0.0373278

CRRT -0.00574684

CRRV -0.0330021

CRRW 0.590027

CRRY 0.460236

CRSA -0.42525

CRSC 0.381513

CRSD -0.133263

CRSE -0.183707

CRSF 0.195894

CRSG -0.487282

CRSH 0.224152

CRSI -0.120437

CRSK -0.154051

CRSL -0.310104

CRSM 0.133732

CRSN 0.0152872

CRSP -0.322124

CRSQ 0.117222

CRSR 0.0420136

CRSS -0.0440502

CRST -0.00878811

CRSV -0.224917

CRSW 0.448956

CRSY 0.291438

CRTA -0.31415

CRTC 0.480994

CRTD -0.0883064

CRTE -0.10277

CRTF 0.300054

CRTG -0.480034

CRTH 0.284366

CRTI 0.0388696

CRTK -0.103752

CRTL -0.210194

CRTM 0.29242

CRTN 0.0689843

CRTP -0.18676

CRTQ 0.175617

CRTR 0.0772805

CRTS -0.038187

CRTT 0.0632381

CRTV -0.0668819

CRTW 0.568878

CRTY 0.393132

CRVA -0.295532

CRVC 0.669597

CRVD -0.267566

CRVE -0.290045

CRVF 0.372626

CRVG -0.650748

CRVH 0.284017

CRVI 0.124068

CRVK -0.205551

CRVL -0.215654

CRVM 0.551177

CRVN -0.132526

CRVP -0.22835

CRVQ 0.0206885

CRVR -0.0310671

CRVS -0.225337

CRVT -0.0710003

CRVV -0.0412364

CRVW 0.749004

CRVY 0.388292

CRWA 0.339389

CRWC 1.12881

CRWD 0.357073

CRWE 0.345402

CRWF 1.13906

CRWG 0.051049

CRWH 0.732876

CRWI 0.89685

CRWK 0.369432

CRWL 0.771283

CRWM 0.948925

CRWN 0.529172

CRWP 0.360586

CRWQ 0.628052

CRWR 0.651632

CRWS 0.374846

CRWT 0.462164

CRWV 0.785197

CRWW 1.29115

CRWY 1.22725

CRYA 0.0721855

CRYC 1.12843

CRYD 0.276675

CRYE 0.15395

CRYF 0.8255

CRYG -0.121394

CRYH 0.690808

CRYI 0.51336

CRYK 0.250615

CRYL 0.261971

CRYM 0.807229

CRYN 0.408296

CRYP 0.283938

CRYQ 0.495116

CRYR 0.477684

CRYS 0.228946

CRYT 0.33736

CRYV 0.356834

CRYW 1.17709

CRYY 0.895227

CSAA -0.487703

CSAC 0.233923

CSAD -0.452507

CSAE -0.444502

CSAF 0.0822358

CSAG -0.890647

CSAH 0.0514686

CSAI -0.147424

CSAK -0.366978

CSAL -0.447584

CSAM 0.235285

CSAN -0.307967

CSAP -0.594538

CSAQ -0.160586

CSAR -0.224345

CSAS -0.386686

CSAT -0.312565

CSAV -0.312371

CSAW 0.396595

CSAY 0.124112

CSCA 0.499516

CSCC 2.36654

CSCD 0.101712

CSCE 0.0343673

CSCF 1.19177

CSCG -0.032975

CSCH 0.759275

CSCI 0.626738

CSCK 0.331403

CSCL 0.637118

CSCM 0.741068

CSCN 0.327451

CSCP 0.191556

CSCQ 0.548078

CSCR 0.535465

CSCS 3.12962

CSCT 0.474136

CSCV 0.666695

CSCW 1.12951

CSCY 1.02523

CSDA -0.383484

CSDC 0.108213

CSDD -0.190074

CSDE -0.217042

CSDF 0.115235

CSDG -0.557666

CSDH 0.268909

CSDI -0.156601

CSDK 0.0308444

CSDL -0.36922

CSDM 0.0278332

CSDN 0.0812213

CSDP -0.328816

CSDQ 0.021807

CSDR 0.178661

CSDS -0.0960064

CSDT -0.0538654

CSDV -0.250513

CSDW 0.408849

CSDY 0.305959

CSEA -0.452399

CSEC 0.0462544

CSED -0.322841

CSEE -0.25583

CSEF 0.0512724

CSEG -0.819268

CSEH 0.179047

CSEI -0.188686

CSEK -0.00541615

CSEL -0.390166

CSEM 0.0709426

CSEN -0.102852

CSEP -0.465446

CSEQ 0.0101635

CSER 0.1266

CSES -0.319292

CSET -0.212663

CSEV -0.30445

CSEW 0.431904

CSEY 0.174915

CSFA 0.0529637

CSFC 0.918738

CSFD 0.151451

CSFE 0.0550752

CSFF 0.810754

CSFG -0.288281

CSFH 0.683826

CSFI 0.526224

CSFK 0.135415

CSFL 0.235257

CSFM 0.895479

CSFN 0.237769

CSFP 0.136179

CSFQ 0.362749

CSFR 0.315138

CSFS 0.188796

CSFT 0.263564

CSFV 0.349981

CSFW 1.10504

CSFY 0.79464

CSGA -0.8878

CSGC -0.14982

CSGD -0.529837

CSGE -0.665569

CSGF -0.203275

CSGG -1.75874

CSGH -0.322001

CSGI -0.487932

CSGK -0.515973

CSGL -0.671939

CSGM -0.267534

CSGN -0.413058

CSGP -0.791072

CSGQ -0.400141

CSGR -0.360705

CSGS -0.537307

CSGT -0.409381

CSGV -0.566868

CSGW -0.0138352

CSGY -0.0970855

CSHA -0.0274894

CSHC 0.680369

CSHD 0.324024

CSHE 0.193567

CSHF 0.680629

CSHG -0.328734

CSHH 0.761395

CSHI 0.332898

CSHK 0.0261812

CSHL 0.281814

CSHM 0.565428

CSHN 0.245396

CSHP 0.0465896

CSHQ 0.22918

CSHR 0.327195

CSHS 0.210261

CSHT 0.281686

CSHV 0.27714

CSHW 0.944329

CSHY 0.737173

CSIA -0.155669

CSIC 0.725861

CSID -0.157698

CSIE -0.173893

CSIF 0.527838

CSIG -0.549245

CSIH 0.381395

CSII 0.322089

CSIK -0.103495

CSIL -0.0264776

CSIM 0.704724

CSIN -0.00252056

CSIP -0.129504

CSIQ 0.126909

CSIR 0.0883887

CSIS -0.0948341

CSIT 0.062351

CSIV 0.123573

CSIW 0.850449

CSIY 0.566566

CSKA -0.424341

CSKC 0.18221

CSKD -0.0182574

CSKE -0.0132744

CSKF 0.117576

CSKG -0.718747

CSKH 0.0066638

CSKI -0.119206

CSKK -0.159666

CSKL -0.339888

CSKM 0.0431638

CSKN -0.0718336

CSKP -0.387414

CSKQ 0.0331106

CSKR -0.160474

CSKS -0.232308

CSKT -0.136894

CSKV -0.233345

CSKW 0.320442

CSKY 0.258631

CSLA -0.446685

CSLC 0.700953

CSLD -0.389533

CSLE -0.389112

CSLF 0.232653

CSLG -0.731341

CSLH 0.28195

CSLI -0.0406158

CSLK -0.320637

CSLL -0.379601

CSLM 0.505657

CSLN -0.231326

CSLP -0.289146

CSLQ 0.00556493

CSLR -0.112381

CSLS -0.33858

CSLT -0.227274

CSLV -0.226465

CSLW 0.756547

CSLY 0.283539

CSMA 0.204911

CSMC 0.822751

CSMD 0.0929687

CSME 0.110741

CSMF 0.895927

CSMG -0.437342

CSMH 0.453975

CSMI 0.669492

CSMK 0.13554

CSML 0.503611

CSMM 0.902667

CSMN 0.126019

CSMP -0.0641434

CSMQ 0.280858

CSMR 0.325009

CSMS 0.0788014

CSMT 0.257999

CSMV 0.525583

CSMW 0.856126

CSMY 0.832354

CSNA -0.298137

CSNC 0.307438

CSND 0.0179656

CSNE -0.0973351

CSNF 0.247309

CSNG -0.449445

CSNH 0.265161

CSNI -0.0402179

CSNK 0.00993633

CSNL -0.213835

CSNM 0.137398

CSNN 0.231529

CSNP -0.152773

CSNQ 0.179987

CSNR 0.108417

CSNS -0.00816226

CSNT 0.0445459

CSNV -0.134379

CSNW 0.601721

CSNY 0.427888

CSPA -0.290052

CSPC 0.256703

CSPD -0.0758033

CSPE -0.0483975

CSPF 0.299846

CSPG -0.476831

CSPH 0.228834

CSPI -0.0214617

CSPK -0.132481

CSPL -0.167399

CSPM 0.207436

CSPN 0.00528407

CSPP -0.172829

CSPQ 0.11197

CSPR 0.0820351

CSPS -0.0581737

CSPT -0.00395489

CSPV -0.0325897

CSPW 0.659185

CSPY 0.390535

CSQA -0.133166

CSQC 0.464025

CSQD -0.0270078

CSQE 0.00250411

CSQF 0.40433

CSQG -0.426846

CSQH 0.216065

CSQI 0.0898628

CSQK 0.0666666

CSQL -0.0212486

CSQM 0.233387

CSQN 0.149594

CSQP -0.184801

CSQQ 0.37291

CSQR 0.202983

CSQS 0.0306814

CSQT 0.114161

CSQV 0.00780344

CSQW 0.615605

CSQY 0.508773

CSRA -0.247235

CSRC 0.375377

CSRD 0.1429

CSRE 0.12909

CSRF 0.354184

CSRG -0.549881

CSRH 0.306601

CSRI 0.0865247

CSRK -0.154186

CSRL -0.124033

CSRM 0.356294

CSRN 0.0422404

CSRP -0.131876

CSRQ 0.212173

CSRR 0.157748

CSRS -0.0429912

CSRT -0.0136662

CSRV -0.0383041

CSRW 0.591798

CSRY 0.455658

CSSA -0.416074

CSSC 0.382666

CSSD -0.144888

CSSE -0.183609

CSSF 0.184477

CSSG -0.491808

CSSH 0.224053

CSSI -0.132406

CSSK -0.160262

CSSL -0.320333

CSSM 0.132965

CSSN 0.00750995

CSSP -0.324712

CSSQ 0.110512

CSSR 0.0116129

CSSS -0.0292735

CSST -0.0148563

CSSV -0.232839

CSSW 0.450446

CSSY 0.282174

CSTA -0.323681

CSTC 0.483077

CSTD -0.0923996

CSTE -0.104934

CSTF 0.293798

CSTG -0.480356

CSTH 0.286819

CSTI 0.0348785

CSTK -0.111276

CSTL -0.214205

CSTM 0.291589

CSTN 0.059787

CSTP -0.190011

CSTQ 0.171331

CSTR 0.0699286

CSTS -0.0422468

CSTT 0.0681295

CSTV -0.073478

CSTW 0.570616

CSTY 0.384657

CSVA -0.287051

CSVC 0.664726

CSVD -0.277412

CSVE -0.298969

CSVF 0.355415

CSVG -0.6552

CSVH 0.292188

CSVI 0.118318

CSVK -0.225932

CSVL -0.235115

CSVM 0.555224

CSVN -0.146757

CSVP -0.23017

CSVQ 0.0165038

CSVR -0.0611374

CSVS -0.19713

CSVT -0.0877597

CSVV -0.0431147

CSVW 0.745133

CSVY 0.373999

CSWA 0.338273

CSWC 1.13369

CSWD 0.359139

CSWE 0.342181

CSWF 1.13783

CSWG 0.0539739

CSWH 0.737605

CSWI 0.894935

CSWK 0.372085

CSWL 0.76621

CSWM 0.953001

CSWN 0.532309

CSWP 0.364073

CSWQ 0.630375

CSWR 0.658856

CSWS 0.377551

CSWT 0.464824

CSWV 0.782389

CSWW 1.29551

CSWY 1.22632

CSYA 0.064919

CSYC 1.12881

CSYD 0.271506

CSYE 0.150683

CSYF 0.828456

CSYG -0.125691

CSYH 0.689025

CSYI 0.506164

CSYK 0.251207

CSYL 0.2507

CSYM 0.807413

CSYN 0.402934

CSYP 0.281928

CSYQ 0.491778

CSYR 0.454097

CSYS 0.25292

CSYT 0.332173

CSYV 0.349666

CSYW 1.17614

CSYY 0.88877

CTAA -0.49435

CTAC 0.231076

CTAD -0.44965

CTAE -0.437334

CTAF 0.097611

CTAG -0.889991

CTAH 0.053808

CTAI -0.14314

CTAK -0.366785

CTAL -0.428864

CTAM 0.239023

CTAN -0.301708

CTAP -0.590537

CTAQ -0.15573

CTAR -0.212638

CTAS -0.425066

CTAT -0.288444

CTAV -0.294023

CTAW 0.398327

CTAY 0.139205

CTCA 0.456001

CTCC 2.36816

CTCD 0.0888846

CTCE 0.0367472

CTCF 0.841488

CTCG -0.0427196

CTCH 0.749457

CTCI 0.700857

CTCK 0.348683

CTCL 0.592796

CTCM 0.739666

CTCN 0.300697

CTCP 0.17377

CTCQ 0.507565

CTCR 0.504676

CTCS 0.488146

CTCT 2.94403

CTCV 0.657727

CTCW 1.12387

CTCY 0.923329

CTDA -0.374266

CTDC 0.102783

CTDD -0.184112

CTDE -0.218692

CTDF 0.124042

CTDG -0.553975

CTDH 0.268892

CTDI -0.150413

CTDK 0.0411742

CTDL -0.360498

CTDM 0.0257423

CTDN 0.085017

CTDP -0.323283

CTDQ 0.025058

CTDR 0.186957

CTDS -0.102378

CTDT -0.0415659

CTDV -0.243327

CTDW 0.40688

CTDY 0.311473

CTEA -0.439671

CTEC 0.0425513

CTED -0.316289

CTEE -0.249537

CTEF 0.0562129

CTEG -0.816236

CTEH 0.179959

CTEI -0.181414

CTEK 0.00509858

CTEL -0.375528

CTEM 0.065942

CTEN -0.104202

CTEP -0.464239

CTEQ 0.0147355

CTER 0.135833

CTES -0.311999

CTET -0.200207

CTEV -0.296819

CTEW 0.431199

CTEY 0.180683

CTFA 0.0614362

CTFC 0.918561

CTFD 0.152827

CTFE 0.0640082

CTFF 0.827259

CTFG -0.286604

CTFH 0.685533

CTFI 0.527918

CTFK 0.140555

CTFL 0.245445

CTFM 0.898384

CTFN 0.240452

CTFP 0.137216

CTFQ 0.366247

CTFR 0.320524

CTFS 0.186978

CTFT 0.272457

CTFV 0.36125

CTFW 1.10558

CTFY 0.803152

CTGA -0.88171

CTGC -0.153952

CTGD -0.528134

CTGE -0.66177

CTGF -0.1978

CTGG -1.76148

CTGH -0.324434

CTGI -0.482756

CTGK -0.5127

CTGL -0.664226

CTGM -0.269998

CTGN -0.406179

CTGP -0.79084

CTGQ -0.39817

CTGR -0.354801

CTGS -0.537217

CTGT -0.404489

CTGV -0.557359

CTGW -0.0172842

CTGY -0.0912104

CTHA -0.0257237

CTHC 0.674754

CTHD 0.325461

CTHE 0.197562

CTHF 0.683509

CTHG -0.333333

CTHH 0.758381

CTHI 0.332623

CTHK 0.0240054

CTHL 0.287645

CTHM 0.560349

CTHN 0.242424

CTHP 0.0443747

CTHQ 0.225917

CTHR 0.330941

CTHS 0.212707

CTHT 0.282286

CTHV 0.281287

CTHW 0.939626

CTHY 0.738757

CTIA -0.142802

CTIC 0.726598

CTID -0.152872

CTIE -0.170869

CTIF 0.541548

CTIG -0.544125

CTIH 0.384534

CTII 0.334081

CTIK -0.096415

CTIL -0.0172355

CTIM 0.709034

CTIN -0.00237513

CTIP -0.124483

CTIQ 0.129983

CTIR 0.0905077

CTIS -0.0861194

CTIT 0.0663512

CTIV 0.139136

CTIW 0.851117

CTIY 0.576027

CTKA -0.414477

CTKC 0.178751

CTKD -0.0120194

CTKE -0.00396514

CTKF 0.122462

CTKG -0.715297

CTKH 0.0050559

CTKI -0.111315

CTKK -0.152739

CTKL -0.330907

CTKM 0.0418468

CTKN -0.067606

CTKP -0.384931

CTKQ 0.0386844

CTKR -0.155844

CTKS -0.223129

CTKT -0.125625

CTKV -0.224964

CTKW 0.317113

CTKY 0.265828

CTLA -0.436159

CTLC 0.703902

CTLD -0.384424

CTLE -0.376212

CTLF 0.254899

CTLG -0.733866

CTLH 0.288232

CTLI -0.0256107

CTLK -0.305138

CTLL -0.347191

CTLM 0.510991

CTLN -0.220121

CTLP -0.287077

CTLQ 0.0142133

CTLR -0.0994244

CTLS -0.333285

CTLT -0.210645

CTLV -0.21926

CTLW 0.761723

CTLY 0.297269

CTMA 0.208637

CTMC 0.817343

CTMD 0.0914719

CTME 0.109612

CTMF 0.899668

CTMG -0.440395

CTMH 0.448854

CTMI 0.673445

CTMK 0.13328

CTML 0.506498

CTMM 0.902468

CTMN 0.123434

CTMP -0.0678055

CTMQ 0.277482

CTMR 0.324742

CTMS 0.077163

CTMT 0.262362

CTMV 0.529733

CTMW 0.850649

CTMY 0.832672

CTNA -0.294966

CTNC 0.30301

CTND 0.0217006

CTNE -0.0917566

CTNF 0.252631

CTNG -0.450919

CTNH 0.263562

CTNI -0.0356669

CTNK 0.0156531

CTNL -0.206127

CTNM 0.13464

CTNN 0.237526

CTNP -0.151401

CTNQ 0.181762

CTNR 0.113423

CTNS -0.00156093

CTNT 0.0531619

CTNV -0.128322

CTNW 0.598435

CTNY 0.432613

CTPA -0.282581

CTPC 0.252219

CTPD -0.0705986

CTPE -0.0421791

CTPF 0.302877

CTPG -0.475177

CTPH 0.227038

CTPI -0.0141404

CTPK -0.128262

CTPL -0.160815

CTPM 0.205187

CTPN 0.00476098

CTPP -0.171335

CTPQ 0.11537

CTPR 0.0862508

CTPS -0.055171

CTPT -0.00101662

CTPV -0.0237486

CTPW 0.657184

CTPY 0.395542

CTQA -0.130562

CTQC 0.459353

CTQD -0.0237992

CTQE 0.00554109

CTQF 0.411687

CTQG -0.425679

CTQH 0.212651

CTQI 0.0945134

CTQK 0.070869

CTQL -0.0133102

CTQM 0.228118

CTQN 0.152236

CTQP -0.18606

CTQQ 0.375037

CTQR 0.206516

CTQS 0.0350926

CTQT 0.116556

CTQV 0.0142879

CTQW 0.611578

CTQY 0.511494

CTRA -0.244811

CTRC 0.37215

CTRD 0.149327

CTRE 0.138017

CTRF 0.35936

CTRG -0.550365

CTRH 0.306033

CTRI 0.0920885

CTRK -0.153914

CTRL -0.117044

CTRM 0.358714

CTRN 0.047878

CTRP -0.130084

CTRQ 0.215434

CTRR 0.168496

CTRS -0.0386415

CTRT -0.00365114

CTRV -0.0314047

CTRW 0.589757

CTRY 0.461336

CTSA -0.428619

CTSC 0.381621

CTSD -0.134813

CTSE -0.185044

CTSF 0.193625

CTSG -0.48353

CTSH 0.224571

CTSI -0.122696

CTSK -0.149433

CTSL -0.308353

CTSM 0.132333

CTSN 0.0152271

CTSP -0.323244

CTSQ 0.116512

CTSR 0.015132

CTSS -0.0426502

CTST 0.00016737

CTSV -0.218574

CTSW 0.449874

CTSY 0.290603

CTTA -0.316423

CTTC 0.481047

CTTD -0.0861177

CTTE -0.101107

CTTF 0.301796

CTTG -0.482059

CTTH 0.284446

CTTI 0.0390141

CTTK -0.103873

CTTL -0.206273

CTTM 0.291991

CTTN 0.0637586

CTTP -0.187248

CTTQ 0.178403

CTTR 0.0701389

CTTS -0.0406022

CTTT 0.0774827

CTTV -0.0664775

CTTW 0.568726

CTTY 0.393462

CTVA -0.289067

CTVC 0.672153

CTVD -0.26895

CTVE -0.270133

CTVF 0.374514

CTVG -0.650982

CTVH 0.284799

CTVI 0.121817

CTVK -0.208445

CTVL -0.215019

CTVM 0.550355

CTVN -0.0793982

CTVP -0.227256

CTVQ 0.0194268

CTVR -0.0542057

CTVS -0.224556

CTVT -0.0649655

CTVV -0.0314279

CTVW 0.752384

CTVY 0.383133

CTWA 0.338907

CTWC 1.12805

CTWD 0.356911

CTWE 0.341757

CTWF 1.13925

CTWG 0.0505288

CTWH 0.73219

CTWI 0.897154

CTWK 0.369007

CTWL 0.772227

CTWM 0.948084

CTWN 0.528732

CTWP 0.360026

CTWQ 0.627739

CTWR 0.651711

CTWS 0.374479

CTWT 0.46217

CTWV 0.785692

CTWW 1.29047

CTWY 1.22738

CTYA 0.0730495

CTYC 1.12846

CTYD 0.276782

CTYE 0.162733

CTYF 0.826881

CTYG -0.121155

CTYH 0.688228

CTYI 0.512964

CTYK 0.252934

CTYL 0.262536

CTYM 0.807507

CTYN 0.406408

CTYP 0.283809

CTYQ 0.495477

CTYR 0.463725

CTYS 0.231251

CTYT 0.341372

CTYV 0.358715

CTYW 1.17653

CTYY 0.893621

CVAA -0.611833

CVAC 0.197146

CVAD -0.539402

CVAE -0.539024

CVAF -0.00480461

CVAG -0.965823

CVAH -0.00242829

CVAI -0.209499

CVAK -0.454304

CVAL -0.591393

CVAM 0.177765

CVAN -0.384071

CVAP -0.652546

CVAQ -0.239455

CVAR -0.297882

CVAS -0.514427

CVAT -0.415926

CVAV -0.331915

CVAW 0.356584

CVAY 0.0338368

CVCA 0.500551

CVCC 2.3006

CVCD 0.0849068

CVCE 0.0118926

CVCF 0.915933

CVCG -0.0368578

CVCH 0.772328

CVCI 1.02582

CVCK 0.199019

CVCL 0.911322

CVCM 0.868979

CVCN 0.256244

CVCP 0.184124

CVCQ 0.671702

CVCR 0.489824

CVCS 0.344885

CVCT 0.321907

CVCV 4.43802

CVCW 1.13908

CVCY 0.928694

CVDA -0.470217

CVDC 0.0986106

CVDD -0.271607

CVDE -0.310763

CVDF 0.0414953

CVDG -0.629364

CVDH 0.227716

CVDI -0.234718

CVDK -0.0679295

CVDL -0.470599

CVDM 0.00209165

CVDN -0.00190902

CVDP -0.380192

CVDQ -0.0425355

CVDR 0.0856948

CVDS -0.19824

CVDT -0.133685

CVDV -0.334353

CVDW 0.384659

CVDY 0.223458

CVEA -0.543217

CVEC 0.0361397

CVED -0.400452

CVEE -0.34757

CVEF -0.0262775

CVEG -0.876991

CVEH 0.136035

CVEI -0.267971

CVEK -0.107883

CVEL -0.498947

CVEM 0.0325201

CVEN -0.181841

CVEP -0.514953

CVEQ -0.0606682

CVER 0.0300541

CVES -0.399082

CVET -0.29604

CVEV -0.403015

CVEW 0.400491

CVEY 0.0958223

CVFA -0.0395007

CVFC 0.884151

CVFD 0.0713201

CVFE -0.021296

CVFF 0.719875

CVFG -0.350627

CVFH 0.636773

CVFI 0.431988

CVFK 0.0575049

CVFL 0.113014

CVFM 0.836962

CVFN 0.17397

CVFP 0.0774107

CVFQ 0.303783

CVFR 0.236357

CVFS 0.100117

CVFT 0.184227

CVFV 0.244776

CVFW 1.06116

CVFY 0.705643

CVGA -0.953588

CVGC -0.157422

CVGD -0.604274

CVGE -0.738127

CVGF -0.271572

CVGG -1.76566

CVGH -0.339755

CVGI -0.553162

CVGK -0.593329

CVGL -0.768396

CVGM -0.288924

CVGN -0.47704

CVGP -0.826644

CVGQ -0.4492

CVGR -0.431437

CVGS -0.615032

CVGT -0.491508

CVGV -0.654268

CVGW -0.0272892

CVGY -0.162139

CVHA -0.0760448

CVHC 0.685961

CVHD 0.274884

CVHE 0.153106

CVHF 0.635189

CVHG -0.351351

CVHH 0.745407

CVHI 0.277709

CVHK -0.00253296

CVHL 0.206225

CVHM 0.561338

CVHN 0.220095

CVHP 0.0316594

CVHQ 0.213883

CVHR 0.290654

CVHS 0.168421

CVHT 0.24071

CVHV 0.232471

CVHW 0.941426

CVHY 0.696846

CVIA -0.25462

CVIC 0.681987

CVID -0.23823

CVIE -0.262926

CVIF 0.434887

CVIG -0.61567

CVIH 0.331953

CVII 0.215869

CVIK -0.184324

CVIL -0.14583

CVIM 0.631452

CVIN -0.0747738

CVIP -0.192463

CVIQ 0.0554504

CVIR -0.00130105

CVIS -0.181804

CVIT -0.0356371

CVIV 0.0180149

CVIW 0.794512

CVIY 0.470825

CVKA -0.512679

CVKC 0.168801

CVKD -0.114272

CVKE -0.110151

CVKF 0.0442722

CVKG -0.77784

CVKH -0.0217137

CVKI -0.202856

CVKK -0.244969

CVKL -0.438889

CVKM 0.0166469

CVKN -0.143038

CVKP -0.435646

CVKQ -0.0316401

CVKR -0.230327

CVKS -0.311394

CVKT -0.219376

CVKV -0.328424

CVKW 0.300041

CVKY 0.179825

CVLA -0.565452

CVLC 0.642175

CVLD -0.489883

CVLE -0.488827

CVLF 0.121749

CVLG -0.828021

CVLH 0.210792

CVLI -0.141845

CVLK -0.420226

CVLL -0.520693

CVLM 0.418775

CVLN -0.316986

CVLP -0.379792

CVLQ -0.0852511

CVLR -0.218069

CVLS -0.443066

CVLT -0.337873

CVLV -0.305673

CVLW 0.709243

CVLY 0.173691

CVMA 0.147695

CVMC 0.823889

CVMD 0.0634778

CVME 0.0751178

CVMF 0.838542

CVMG -0.448838

CVMH 0.454678

CVMI 0.607513

CVMK 0.102715

CVML 0.408036

CVMM 0.883745

CVMN 0.110228

CVMP -0.0707014

CVMQ 0.265342

CVMR 0.29312

CVMS 0.0529392

CVMT 0.225705

CVMV 0.455299

CVMW 0.856542

CVMY 0.786513

CVNA -0.37404

CVNC 0.301925

CVND -0.0594056

CVNE -0.1723

CVNF 0.186707

CVNG -0.511492

CVNH 0.238661

CVNI -0.105476

CVNK -0.0652471

CVNL -0.305694

CVNM 0.121725

CVNN 0.163579

CVNP -0.197537

CVNQ 0.123982

CVNR 0.0399897

CVNS -0.0725815

CVNT -0.0278533

CVNV -0.198222

CVNW 0.583966

CVNY 0.357205

CVPA -0.369515

CVPC 0.253084

CVPD -0.14631

CVPE -0.127845

CVPF 0.230394

CVPG -0.536242

CVPH 0.20285

CVPI -0.0957239

CVPK -0.199898

CVPL -0.261953

CVPM 0.185761

CVPN -0.0542853

CVPP -0.2184

CVPQ 0.0599196

CVPR 0.0143318

CVPS -0.129325

CVPT -0.0797334

CVPV -0.11193

CVPW 0.63445

CVPY 0.321193

CVQA -0.211212

CVQC 0.459189

CVQD -0.0867121

CVQE -0.0638626

CVQF 0.340939

CVQG -0.474254

CVQH 0.202294

CVQI 0.0243444

CVQK -0.00141096

CVQL -0.110411

CVQM 0.219642

CVQN 0.0970025

CVQP -0.216143

CVQQ 0.315948

CVQR 0.141275

CVQS -0.0330279

CVQT 0.0458939

CVQV -0.0616398

CVQW 0.604918

CVQY 0.445669

CVRA -0.33852

CVRC 0.363035

CVRD 0.0492296

CVRE 0.0308099

CVRF 0.273673

CVRG -0.610094

CVRH 0.272067

CVRI -0.00024819

CVRK -0.229239

CVRL -0.229957

CVRM 0.321964

CVRN -0.0177748

CVRP -0.184597

CVRQ 0.149535

CVRR 0.0794334

CVRS -0.123419

CVRT -0.0908227

CVRV -0.120724

CVRW 0.567743

CVRY 0.376615

CVSA -0.525485

CVSC 0.359665

CVSD -0.226835

CVSE -0.280056

CVSF 0.105571

CVSG -0.569211

CVSH 0.182312

CVSI -0.217592

CVSK -0.24256

CVSL -0.419008

CVSM 0.100853

CVSN -0.0660121

CVSP -0.378118

CVSQ 0.0383475

CVSR -0.0735335

CVSS -0.144094

CVST -0.105817

CVSV -0.30401

CVSW 0.425678

CVSY 0.203526

CVTA -0.410332

CVTC 0.453659

CVTD -0.175933

CVTE -0.201272

CVTF 0.209472

CVTG -0.5603

CVTH 0.240185

CVTI -0.0473607

CVTK -0.193202

CVTL -0.319521

CVTM 0.249097

CVTN -0.0156934

CVTP -0.250087

CVTQ 0.100248

CVTR -0.0192266

CVTS -0.13688

CVTT -0.040029

CVTV -0.14367

CVTW 0.537032

CVTY 0.301359

CVVA -0.420939

CVVC 0.603219

CVVD -0.363291

CVVE -0.388132

CVVF 0.263074

CVVG -0.733265

CVVH 0.227261

CVVI 0.0147014

CVVK -0.312245

CVVL -0.361498

CVVM 0.474874

CVVN -0.214385

CVVP -0.301996

CVVQ -0.0644178

CVVR -0.145995

CVVS -0.327855

CVVT -0.186408

CVVV -0.082561

CVVW 0.68355

CVVY 0.274375

CVWA 0.296858

CVWC 1.13951

CVWD 0.33771

CVWE 0.323289

CVWF 1.09118

CVWG 0.0364521

CVWH 0.742291

CVWI 0.840077

CVWK 0.348885

CVWL 0.688136

CVWM 0.951681

CVWN 0.516288

CVWP 0.356091

CVWQ 0.620079

CVWR 0.625141

CVWS 0.357074

CVWT 0.438282

CVWV 0.721178

CVWW 1.29658

CVWY 1.18123

CVYA -0.0238533

CVYC 1.08909

CVYD 0.190628

CVYE 0.0709476

CVYF 0.728704

CVYG -0.19142

CVYH 0.650559

CVYI 0.407606

CVYK 0.171606

CVYL 0.136979

CVYM 0.755147

CVYN 0.336816

CVYP 0.220264

CVYQ 0.430408

CVYR 0.37695

CVYS 0.147948

CVYT 0.261033

CVYV 0.294073

CVYW 1.1338

CVYY 0.797488

CWAA -0.448607

CWAC 0.212051

CWAD -0.424589

CWAE -0.404546

CWAF 0.131229

CWAG -0.880344

CWAH 0.0486882

CWAI -0.100859

CWAK -0.336953

CWAL -0.378421

CWAM 0.241791

CWAN -0.285586

CWAP -0.5947

CWAQ -0.140377

CWAR -0.181193

CWAS -0.397914

CWAT -0.291293

CWAV -0.252283

CWAW 0.388153

CWAY 0.166625

CWCA 0.282941

CWCC 2.37314

CWCD 0.0477045

CWCE -0.00832009

CWCF 0.878351

CWCG -0.061692

CWCH 0.708356

CWCI 0.615733

CWCK 0.217431

CWCL 0.605597

CWCM 0.697057

CWCN 0.259683

CWCP 0.133055

CWCQ 0.439006

CWCR 0.432642

CWCS 0.296433

CWCT 0.276784

CWCV 0.627814

CWCW 2.02549

CWCY 0.900357

CWDA -0.344591

CWDC 0.0666254

CWDD -0.164368

CWDE -0.194118

CWDF 0.135907

CWDG -0.543903

CWDH 0.256922

CWDI -0.131437

CWDK 0.077009

CWDL -0.325871

CWDM -0.00071311

CWDN 0.104378

CWDP -0.332032

CWDQ 0.0286472

CWDR 0.219554

CWDS -0.0745273

CWDT -0.0194025

CWDV -0.215549

CWDW 0.379369

CWDY 0.330577

CWEA -0.409977

CWEC 0.00696254

CWED -0.300058

CWEE -0.220422

CWEF 0.0716186

CWEG -0.817338

CWEH 0.168376

CWEI -0.158487

CWEK 0.0461781

CWEL -0.338874

CWEM 0.0460575

CWEN -0.0909674

CWEP -0.472731

CWEQ 0.0242269

CWER 0.171093

CWES -0.295335

CWET -0.182542

CWEV -0.266463

CWEW 0.408743

CWEY 0.197493

CWFA 0.0949397

CWFC 0.897642

CWFD 0.169108

CWFE 0.0776739

CWFF 0.861184

CWFG -0.284842

CWFH 0.674504

CWFI 0.567461

CWFK 0.156982

CWFL 0.296934

CWFM 0.897397

CWFN 0.244028

CWFP 0.135845

CWFQ 0.36676

CWFR 0.332882

CWFS 0.207571

CWFT 0.29116

CWFV 0.399128

CWFW 1.09323

CWFY 0.829698

CWGA -0.871632

CWGC -0.190712

CWGD -0.515728

CWGE -0.655441

CWGF -0.19047

CWGG -1.79975

CWGH -0.35342

CWGI -0.47144

CWGK -0.497534

CWGL -0.63363

CWGM -0.298641

CWGN -0.411374

CWGP -0.810265

CWGQ -0.406939

CWGR -0.344434

CWGS -0.524238

CWGT -0.38839

CWGV -0.53737

CWGW -0.0512054

CWGY -0.0837884

CWHA -0.0356052

CWHC 0.633911

CWHD 0.316593

CWHE 0.184558

CWHF 0.67263

CWHG -0.362695

CWHH 0.726062

CWHI 0.320487

CWHK 0.00098467

CWHL 0.301145

CWHM 0.522006

CWHN 0.216289

CWHP 0.0105913

CWHQ 0.192766

CWHR 0.309406

CWHS 0.196897

CWHT 0.268619

CWHV 0.279523

CWHW 0.90116

CWHY 0.725517

CWIA -0.0979946

CWIC 0.713805

CWID -0.133869

CWIE -0.144752

CWIF 0.580914

CWIG -0.536484

CWIH 0.37625

CWII 0.381454

CWIK -0.0728331

CWIL 0.0397933

CWIM 0.718398

CWIN 0.0108438

CWIP -0.120733

CWIQ 0.135332

CWIR 0.114335

CWIS -0.0639942

CWIT 0.097451

CWIV 0.18235

CWIW 0.844821

CWIY 0.610163

CWKA -0.386128

CWKC 0.144747

CWKD 0.0214288

CWKE 0.0368326

CWKF 0.137114

CWKG -0.715418

CWKH -0.0194869

CWKI -0.0913668

CWKK -0.129423

CWKL -0.292514

CWKM 0.016819

CWKN -0.0572891

CWKP -0.394981

CWKQ 0.043437

CWKR -0.145843

CWKS -0.205399

CWKT -0.108148

CWKV -0.194891

CWKW 0.28693

CWKY 0.282721

CWLA -0.385831

CWLC 0.717583

CWLD -0.350164

CWLE -0.338027

CWLF 0.30479

CWLG -0.708252

CWLH 0.303986

CWLI 0.0297263

CWLK -0.267705

CWLL -0.299469

CWLM 0.536499

CWLN -0.195571

CWLP -0.266861

CWLQ 0.0429032

CWLR -0.0599546

CWLS -0.299354

CWLT -0.174367

CWLV -0.156862

CWLW 0.778378

CWLY 0.342833

CWMA 0.20984

CWMC 0.777311

CWMD 0.0676625

CWME 0.0929692

CWMF 0.898607

CWMG -0.474884

CWMH 0.409276

CWMI 0.681088

CWMK 0.112908

CWML 0.532762

CWMM 0.869746

CWMN 0.0907292

CWMP -0.10543

CWMQ 0.244606

CWMR 0.303348

CWMS 0.0532687

CWMT 0.240363

CWMV 0.542276

CWMW 0.810587

CWMY 0.821813

CWNA -0.278155

CWNC 0.265227

CWND 0.0370376

CWNE -0.0792425

CWNF 0.256845

CWNG -0.451644

CWNH 0.237694

CWNI -0.0279646

CWNK 0.0314784

CWNL -0.179105

CWNM 0.101642

CWNN 0.248557

CWNP -0.163385

CWNQ 0.178011

CWNR 0.122442

CWNS 0.0107553

CWNT 0.0650785

CWNV -0.11249

CWNW 0.56829

CWNY 0.440716

CWPA -0.263284

CWPC 0.213937

CWPD -0.0605021

CWPE -0.0235596

CWPF 0.312074

CWPG -0.476098

CWPH 0.200699

CWPI -0.00456786

CWPK -0.120542

CWPL -0.130058

CWPM 0.175914

CWPN 0.00171828

CWPP -0.183212

CWPQ 0.107439

CWPR 0.0946698

CWPS -0.0424924

CWPT 0.0113239

CWPV 0.00168109

CWPW 0.629948

CWPY 0.403031

CWQA -0.115409

CWQC 0.421126

CWQD -0.0234859

CWQE 0.0101798

CWQF 0.410185

CWQG -0.435954

CWQH 0.178805

CWQI 0.0988574

CWQK 0.0783844

CWQL 0.014353

CWQM 0.193679

CWQN 0.14627

CWQP -0.208451

CWQQ 0.372552

CWQR 0.209782

CWQS 0.0398929

CWQT 0.123338

CWQV 0.0283146

CWQW 0.576431

CWQY 0.514891

CWRA -0.218967

CWRC 0.337674

CWRD 0.179014

CWRE 0.172533

CWRF 0.376604

CWRG -0.552087

CWRH 0.286183

CWRI 0.115247

CWRK -0.14342

CWRL -0.0769334

CWRM 0.335847

CWRN 0.0489628

CWRP -0.1356

CWRQ 0.219282

CWRR 0.18286

CWRS -0.0195947

CWRT 0.00831461

CWRV -0.00176501

CWRW 0.56215

CWRY 0.478123

CWSA -0.396994

CWSC 0.354323

CWSD -0.111529

CWSE -0.155811

CWSF 0.21397

CWSG -0.471121

CWSH 0.211383

CWSI -0.102107

CWSK -0.125714

CWSL -0.268527

CWSM 0.110691

CWSN 0.0314209

CWSP -0.327664

CWSQ 0.127774

CWSR 0.0384412

CWSS -0.00820398

CWST 0.020627

CWSV -0.192186

CWSW 0.424524

CWSY 0.31046

CWTA -0.284393

CWTC 0.455572

CWTD -0.0630841

CWTE -0.0739794

CWTF 0.324693

CWTG -0.470294

CWTH 0.271299

CWTI 0.0693061

CWTK -0.078409

CWTL -0.165022

CWTM 0.276874

CWTN 0.0802381

CWTP -0.186951

CWTQ 0.188019

CWTR 0.0911684

CWTS -0.0117264

CWTT 0.094377

CWTV -0.0277808

CWTW 0.547118

CWTY 0.415721

CWVA -0.245923

CWVC 0.666526

CWVD -0.243705

CWVE -0.256135

CWVF 0.41804

CWVG -0.634708

CWVH 0.286127

CWVI 0.173214

CWVK -0.177592

CWVL -0.155447

CWVM 0.566462

CWVN -0.113829

CWVP -0.215145

CWVQ 0.0367246

CWVR -0.0224655

CWVS -0.190803

CWVT -0.0426333

CWVV 0.0269518

CWVW 0.750817

CWVY 0.422229

CWWA 0.324005

CWWC 1.0871

CWWD 0.327568

CWWE 0.31464

CWWF 1.1284

CWWG 0.0186088

CWWH 0.691544

CWWI 0.892548

CWWK 0.3408

CWWL 0.788029

CWWM 0.909001

CWWN 0.49604

CWWP 0.323327

CWWQ 0.592574

CWWR 0.626411

CWWS 0.346606

CWWT 0.435154

CWWV 0.788187

CWWW 1.2505

CWWY 1.21582

CWYA 0.0986242

CWYC 1.11292

CWYD 0.294254

CWYE 0.170682

CWYF 0.855692

CWYG -0.115337

CWYH 0.672507

CWYI 0.544472

CWYK 0.270973

CWYL 0.309499

CWYM 0.793134

CWYN 0.414048

CWYP 0.285012

CWYQ 0.497959

CWYR 0.478574

CWYS 0.245886

CWYT 0.357885

CWYV 0.394778

CWYW 1.16325

CWYY 0.919605

CYAA -0.520582

CYAC 0.230377

CYAD -0.465512

CYAE -0.455923

CYAF 0.0734458

CYAG -0.898509

CYAH 0.0485938

CYAI -0.165698

CYAK -0.387753

CYAL -0.455802

CYAM 0.230546

CYAN -0.307038

CYAP -0.599985

CYAQ -0.164716

CYAR -0.224523

CYAS -0.445297

CYAT -0.341615

CYAV -0.322408

CYAW 0.395262

CYAY 0.134819

CYCA 0.335498

CYCC 2.35575

CYCD 0.0941021

CYCE 0.0372846

CYCF 1.0649

CYCG -0.021445

CYCH 0.76539

CYCI 0.63627

CYCK 0.249377

CYCL 0.593879

CYCM 0.744944

CYCN 0.308161

CYCP 0.191087

CYCQ 0.474027

CYCR 0.463368

CYCS 0.428474

CYCT 0.312558

CYCV 0.653744

CYCW 1.13668

CYCY 4.00472

CYDA -0.392196

CYDC 0.110741

CYDD -0.198137

CYDE -0.233912

CYDF 0.108458

CYDG -0.565529

CYDH 0.26553

CYDI -0.164667

CYDK 0.0208647

CYDL -0.381064

CYDM 0.0289872

CYDN 0.0700152

CYDP -0.330586

CYDQ 0.0159252

CYDR 0.167408

CYDS -0.116845

CYDT -0.0640302

CYDV -0.261622

CYDW 0.410046

CYDY 0.298478

CYEA -0.456174

CYEC 0.0499666

CYED -0.330828

CYEE -0.266432

CYEF 0.0422721

CYEG -0.823745

CYEH 0.176718

CYEI -0.19597

CYEK -0.0159638

CYEL -0.399857

CYEM 0.0667336

CYEN -0.116439

CYEP -0.468896

CYEQ 0.00376916

CYER 0.115273

CYES -0.327898

CYET -0.215949

CYEV -0.316852

CYEW 0.431786

CYEY 0.167937

CYFA 0.0421267

CYFC 0.919384

CYFD 0.139506

CYFE 0.0486646

CYFF 0.802989

CYFG -0.295021

CYFH 0.681128

CYFI 0.505672

CYFK 0.128247

CYFL 0.221472

CYFM 0.891181

CYFN 0.233172

CYFP 0.130131

CYFQ 0.358329

CYFR 0.30517

CYFS 0.173147

CYFT 0.254725

CYFV 0.33679

CYFW 1.10184

CYFY 0.796782

CYGA -0.892289

CYGC -0.144951

CYGD -0.540023

CYGE -0.675739

CYGF -0.207227

CYGG -1.75438

CYGH -0.318973

CYGI -0.493805

CYGK -0.524239

CYGL -0.682763

CYGM -0.265541

CYGN -0.421215

CYGP -0.791303

CYGQ -0.403083

CYGR -0.366444

CYGS -0.549131

CYGT -0.419693

CYGV -0.575519

CYGW -0.0106456

CYGY -0.0987821

CYHA -0.029608

CYHC 0.686398

CYHD 0.320924

CYHE 0.191858

CYHF 0.680597

CYHG -0.328964

CYHH 0.764306

CYHI 0.327098

CYHK 0.0263367

CYHL 0.274168

CYHM 0.56969

CYHN 0.246494

CYHP 0.049597

CYHQ 0.232647

CYHR 0.32759

CYHS 0.209018

CYHT 0.279683

CYHV 0.273135

CYHW 0.94908

CYHY 0.739086

CYIA -0.164242

CYIC 0.723836

CYID -0.166774

CYIE -0.18528

CYIF 0.51491

CYIG -0.554686

CYIH 0.378799

CYII 0.307276

CYIK -0.112417

CYIL -0.0418093

CYIM 0.698163

CYIN -0.0117135

CYIP -0.133941

CYIQ 0.118823

CYIR 0.0765579

CYIS -0.102318

CYIT 0.0464151

CYIV 0.102114

CYIW 0.845916

CYIY 0.559282

CYKA -0.431606

CYKC 0.185325

CYKD -0.0302289

CYKE -0.0252087

CYKF 0.109696

CYKG -0.723545

CYKH 0.00697327

CYKI -0.129579

CYKK -0.169152

CYKL -0.352042

CYKM 0.0445871

CYKN -0.0783067

CYKP -0.390318

CYKQ 0.0285525

CYKR -0.166869

CYKS -0.237961

CYKT -0.145036

CYKV -0.243294

CYKW 0.321765

CYKY 0.256712

CYLA -0.463712

CYLC 0.691326

CYLD -0.403284

CYLE -0.400665

CYLF 0.231728

CYLG -0.74898

CYLH 0.283852

CYLI -0.0462148

CYLK -0.328589

CYLL -0.39047

CYLM 0.490988

CYLN -0.237978

CYLP -0.30339

CYLQ -0.00557351

CYLR -0.122498

CYLS -0.356561

CYLT -0.237248

CYLV -0.244203

CYLW 0.748403

CYLY 0.278956

CYMA 0.200109

CYMC 0.828316

CYMD 0.0930245

CYME 0.11118

CYMF 0.892853

CYMG -0.433646

CYMH 0.459311

CYMI 0.661963

CYMK 0.134614

CYML 0.488632

CYMM 0.908109

CYMN 0.131131

CYMP -0.0598805

CYMQ 0.284137

CYMR 0.326015

CYMS 0.0787494

CYMT 0.25799

CYMV 0.519274

CYMW 0.86148

CYMY 0.828725

CYNA -0.307729

CYNC 0.311906

CYND 0.0091517

CYNE -0.105079

CYNF 0.243493

CYNG -0.457585

CYNH 0.266267

CYNI -0.0466752

CYNK 0.00311184

CYNL -0.223323

CYNM 0.140754

CYNN 0.22635

CYNP -0.153948

CYNQ 0.175408

CYNR 0.103614

CYNS -0.0165312

CYNT 0.0391996

CYNV -0.13745

CYNW 0.603492

CYNY 0.423486

CYPA -0.296302

CYPC 0.26157

CYPD -0.0823011

CYPE -0.0566468

CYPF 0.29154

CYPG -0.482146

CYPH 0.230018

CYPI -0.0277231

CYPK -0.138415

CYPL -0.181149

CYPM 0.209323

CYPN -0.0025661

CYPP -0.173752

CYPQ 0.108602

CYPR 0.0756555

CYPS -0.0671778

CYPT -0.0133119

CYPV -0.0415876

CYPW 0.660396

CYPY 0.384394

CYQA -0.14402

CYQC 0.468438

CYQD -0.0317419

CYQE -0.00889659

CYQF 0.399057

CYQG -0.429686

CYQH 0.219072

CYQI 0.0843015

CYQK 0.0608158

CYQL -0.0309966

CYQM 0.235378

CYQN 0.146208

CYQP -0.184773

CYQQ 0.368405

CYQR 0.197717

CYQS 0.0254438

CYQT 0.105755

CYQV 0.00176144

CYQW 0.61903

CYQY 0.503042

CYRA -0.261538

CYRC 0.378557

CYRD 0.130597

CYRE 0.116929

CYRF 0.346115

CYRG -0.557356

CYRH 0.306806

CYRI 0.076853

CYRK -0.164782

CYRL -0.138283

CYRM 0.356175

CYRN 0.0361817

CYRP -0.135644

CYRQ 0.206464

CYRR 0.151895

CYRS -0.0516424

CYRT -0.0208955

CYRV -0.0501921

CYRW 0.593567

CYRY 0.448572

CYSA -0.440584

CYSC 0.38431

CYSD -0.151214

CYSE -0.200332

CYSF 0.178529

CYSG -0.500266

CYSH 0.224441

CYSI -0.139193

CYSK -0.164402

CYSL -0.331923

CYSM 0.133612

CYSN 0.00252509

CYSP -0.32866

CYSQ 0.106724

CYSR 0.00313187

CYSS -0.0604925

CYST -0.0285378

CYSV -0.238417

CYSW 0.452015

CYSY 0.294998

CYTA -0.334517

CYTC 0.482777

CYTD -0.102729

CYTE -0.121229

CYTF 0.283496

CYTG -0.494461

CYTH 0.282176

CYTI 0.0216382

CYTK -0.117801

CYTL -0.232043

CYTM 0.292246

CYTN 0.0518124

CYTP -0.194922

CYTQ 0.165596

CYTR 0.0540948

CYTS -0.0583444

CYTT 0.0436106

CYTV -0.0882962

CYTW 0.572116

CYTY 0.385457

CYVA -0.322529

CYVC 0.6629

CYVD -0.286931

CYVE -0.306808

CYVF 0.346958

CYVG -0.663999

CYVH 0.277293

CYVI 0.0966239

CYVK -0.22716

CYVL -0.241189

CYVM 0.539243

CYVN -0.143945

CYVP -0.239193

CYVQ 0.00837708

CYVR -0.0687521

CYVS -0.245538

CYVT -0.0995324

CYVV -0.0655913

CYVW 0.743287

CYVY 0.374198

CYWA 0.336795

CYWC 1.13973

CYWD 0.361364

CYWE 0.34361

CYWF 1.136

CYWG 0.0561368

CYWH 0.744309

CYWI 0.891595

CYWK 0.373371

CYWL 0.759062

CYWM 0.957918

CYWN 0.53492

CYWP 0.368098

CYWQ 0.634716

CYWR 0.65352

CYWS 0.378004

CYWT 0.465177

CYWV 0.776343

CYWW 1.30091

CYWY 1.22421

CYYA 0.0597897

CYYC 1.12651

CYYD 0.263842

CYYE 0.142844

CYYF 0.80749

CYYG -0.130446

CYYH 0.68685

CYYI 0.494756

CYYK 0.240751

CYYL 0.241472

CYYM 0.802895

CYYN 0.397279

CYYP 0.275424

CYYQ 0.487953

CYYR 0.44945

CYYS 0.21728

CYYT 0.324326

CYYV 0.339846

CYYW 1.17601

CYYY 0.886271

DAAA -0.768248

DAAC -0.0392234

DAAD -0.841197

DAAE -0.83188

DAAF -0.306003

DAAG -1.20041

DAAH -0.255469

DAAI -0.58578

DAAK -0.763123

DAAL -0.899177

DAAM -0.0984523

DAAN -0.646912

DAAP -0.915078

DAAQ -0.491847

DAAR -0.61731

DAAS -0.801963

DAAT -0.722859

DAAV -0.729076

DAAW 0.106203

DAAY -0.278064

DACA 0.020993

DACC 2.02139

DACD -0.119462

DACE -0.181654

DACF 0.635887

DACG -0.24673

DACH 0.57649

DACI 0.355482

DACK 0.0224109

DACL 0.252334

DACM 0.568672

DACN 0.101262

DACP -0.0130551

DACQ 0.278876

DACR 0.234635

DACS 0.0852759

DACT 0.0804842

DACV 0.320353

DACW 0.957249

DACY 0.673324

DADA 4.14246

DADC -0.0554202

DADD -0.484821

DADE -0.325776

DADF -0.130517

DADG -0.70098

DADH 0.00226378

DADI -0.441223

DADK -0.284059

DADL -0.61972

DADM -0.185265

DADN -0.206207

DADP -0.516002

DADQ -0.194335

DADR -0.131511

DADS 0.181017

DADT -0.131307

DADV -0.401241

DADW 0.182632

DADY -0.0310707

DAEA -0.416976

DAEC -0.159453

DAED -0.673243

DAEE -0.58341

DAEF -0.323977

DAEG -1.13096

DAEH -0.111964

DAEI -0.568412

DAEK -0.371004

DAEL -0.818164

DAEM -0.207417

DAEN -0.459587

DAEP -0.758013

DAEQ -0.344033

DAER -0.209332

DAES -0.630941

DAET -0.587859

DAEV -0.575946

DAEW 0.166979

DAEY -0.182255

DAFA -0.296867

DAFC 0.651591

DAFD -0.214793

DAFE -0.2977

DAFF 0.404775

DAFG -0.621448

DAFH 0.387078

DAFI 0.100133

DAFK -0.22882

DAFL -0.207003

DAFM 0.573875

DAFN -0.0970461

DAFP -0.186569

DAFQ 0.0424345

DAFR -0.0521355

DAFS -0.196054

DAFT -0.114941

DAFV -0.0822842

DAFW 0.812243

DAFY 0.414048

DAGA -1.1726

DAGC -0.357382

DAGD -0.884914

DAGE -1.01324

DAGF -0.546439

DAGG -1.96486

DAGH -0.555438

DAGI -0.837976

DAGK -0.87197

DAGL -1.07559

DAGM -0.508095

DAGN -0.740532

DAGP -1.05557

DAGQ -0.705506

DAGR -0.717772

DAGS -0.906166

DAGT -0.776173

DAGV -0.941338

DAGW -0.233927

DAGY -0.43655

DAHA -0.303538

DAHC 0.506814

DAHD 0.0362427

DAHE -0.089607

DAHF 0.389146

DAHG -0.561257

DAHH 0.533927

DAHI 0.0347972

DAHK -0.236716

DAHL -0.0808651

DAHM 0.372609

DAHN -0.00681734

DAHP -0.180528

DAHQ 0.00684452

DAHR 0.0566432

DAHS -0.0652416

DAHT -0.00426173

DAHV -0.0493994

DAHW 0.748859

DAHY 0.452811

DAIA -0.581415

DAIC 0.43518

DAID -0.530196

DAIE -0.558307

DAIF 0.115086

DAIG -0.88401

DAIH 0.0767703

DAII -0.120996

DAIK -0.487566

DAIL -0.486854

DAIM 0.356144

DAIN -0.352385

DAIP -0.462339

DAIQ -0.219535

DAIR -0.293947

DAIS -0.477491

DAIT -0.341165

DAIV -0.32385

DAIW 0.543003

DAIY 0.156135

DAKA -0.736346

DAKC -0.0398507

DAKD -0.421533

DAKE -0.411613

DAKF -0.240637

DAKG -1.03663

DAKH -0.231467

DAKI -0.500436

DAKK -0.534279

DAKL -0.722941

DAKM -0.213618

DAKN -0.402114

DAKP -0.688811

DAKQ -0.310026

DAKR -0.504672

DAKS -0.612452

DAKT -0.511199

DAKV -0.557879

DAKW 0.0832043

DAKY -0.101212

DALA -0.863107

DALC 0.339936

DALD -0.79294

DALE -0.796337

DALF -0.204006

DALG -1.11705

DALH -0.0792005

DALI -0.499611

DALK -0.732619

DALL -0.841851

DALM 0.118241

DALN -0.61811

DALP -0.675506

DALQ -0.386053

DALR -0.527633

DALS -0.766832

DALT -0.658488

DALV -0.688291

DALW 0.392433

DALY -0.151273

DAMA -0.122646

DAMC 0.643782

DAMD -0.165608

DAME -0.160797

DAMF 0.574625

DAMG -0.657484

DAMH 0.271133

DAMI 0.326713

DAMK -0.132638

DAML 0.109595

DAMM 0.678041

DAMN -0.092093

DAMP -0.268536

DAMQ 0.0603666

DAMR 0.0599287

DAMS -0.184342

DAMT -0.0166051

DAMV 0.166413

DAMW 0.676995

DAMY 0.535397

DANA -0.327024

DANC 0.108735

DAND -0.325313

DANE -0.445326

DANF -0.0950515

DANG -0.774325

DANH 0.0167494

DANI -0.399362

DANK -0.34291

DANL -0.575072

DANM -0.0895572

DANN -0.122047

DANP -0.438257

DANQ -0.133572

DANR -0.233785

DANS -0.335395

DANT -0.301441

DANV -0.454333

DANW 0.363245

DANY 0.105359

DAPA -0.626662

DAPC 0.0598185

DAPD -0.422697

DAPE -0.393852

DAPF -0.0493808

DAPG -0.799218

DAPH -0.0225093

DAPI -0.375268

DAPK -0.482578

DAPL -0.566513

DAPM -0.0326288

DAPN -0.308007

DAPP -0.468657

DAPQ -0.183421

DAPR -0.261802

DAPS -0.410382

DAPT -0.364443

DAPV -0.421668

DAPW 0.412538

DAPY 0.0439725

DAQA -0.441682

DAQC 0.262879

DAQD -0.352413

DAQE -0.336503

DAQF 0.0711448

DAQG -0.729847

DAQH -0.00912476

DAQI -0.244263

DAQK -0.280655

DAQL -0.3775

DAQM 0.0186386

DAQN -0.152895

DAQP -0.44937

DAQQ 0.061502

DAQR -0.132904

DAQS -0.310363

DAQT -0.232285

DAQV -0.357845

DAQW 0.400624

DAQY 0.170331

DARA -0.637678

DARC 0.155787

DARD -0.253844

DARE -0.288769

DARF -0.0141063

DARG -0.873805

DARH 0.0336096

DARI -0.293539

DARK -0.508645

DARL -0.540437

DARM 0.089699

DARN -0.300716

DARP -0.439546

DARQ -0.11048

DARR -0.213488

DARS -0.393041

DART -0.371096

DARV -0.445469

DARW 0.346049

DARY 0.0885673

DASA -0.75977

DASC 0.13608

DASD -0.496873

DASE -0.566343

DASF -0.181578

DASG -0.847486

DASH -0.053354

DASI -0.501206

DASK -0.532158

DASL -0.737072

DASM -0.131139

DASN -0.338167

DASP -0.637156

DASQ -0.249867

DASR -0.372747

DASS -0.461689

DAST -0.419671

DASV -0.617755

DASW 0.194806

DASY -0.0876884

DATA -0.704388

DATC 0.228134

DATD -0.47189

DATE -0.482176

DATF -0.0928755

DATG -0.831501

DATH -0.00127912

DATI -0.370974

DATK -0.488266

DATL -0.644484

DATM 0.00812936

DATN -0.287526

DATP -0.512235

DATQ -0.176646

DATR -0.317232

DATS -0.449105

DATT -0.32524

DATV -0.481205

DATW 0.306444

DATY -0.00345087

DAVA -0.710636

DAVC 0.347423

DAVD -0.664803

DAVE -0.65416

DAVF -0.0645888

DAVG -1.01891

DAVH -0.0342135

DAVI -0.32319

DAVK -0.619463

DAVL -0.692873

DAVM 0.189562

DAVN -0.507109

DAVP -0.587433

DAVQ -0.340381

DAVR -0.443571

DAVS -0.626164

DAVT -0.505077

DAVV -0.492105

DAVW 0.419618

DAVY -0.0233428

DAWA 0.0624812

DAWC 0.96115

DAWD 0.121007

DAWE 0.102719

DAWF 0.840607

DAWG -0.175426

DAWH 0.564711

DAWI 0.580527

DAWK 0.128394

DAWL 0.398953

DAWM 0.76607

DAWN 0.31622

DAWP 0.156339

DAWQ 0.414412

DAWR 0.399797

DAWS 0.136972

DAWT 0.220836

DAWV 0.450223

DAWW 1.11098

DAWY 0.931129

DAYA -0.2952

DAYC 0.844873

DAYD -0.100842

DAYE -0.212551

DAYF 0.427469

DAYG -0.460318

DAYH 0.406478

DAYI 0.104127

DAYK -0.121222

DAYL -0.178601

DAYM 0.513159

DAYN 0.0603788

DAYP -0.0497818

DAYQ 0.16004

DAYR 0.0977888

DAYS -0.147399

DAYT -0.0344148

DAYV -0.0594056

DAYW 0.889451

DAYY 0.505092

DCAA -0.474744

DCAC 0.238168

DCAD -0.437655

DCAE -0.419665

DCAF 0.126617

DCAG -0.8849

DCAH 0.0545533

DCAI -0.1259

DCAK -0.355141

DCAL -0.412668

DCAM 0.242423

DCAN -0.289513

DCAP -0.59115

DCAQ -0.145827

DCAR -0.197119

DCAS -0.413574

DCAT -0.308846

DCAV -0.281281

DCAW 0.397103

DCAY 0.148707

DCCA 0.29526

DCCC 2.37339

DCCD 0.0733383

DCCE 0.0167758

DCCF 0.893901

DCCG -0.0378149

DCCH 0.735681

DCCI 0.628178

DCCK 0.24059

DCCL 0.602472

DCCM 0.723548

DCCN 0.286041

DCCP 0.159993

DCCQ 0.465132

DCCR 0.454736

DCCS 0.318813

DCCT 0.299373

DCCV 0.631021

DCCW 1.11031

DCCY 0.918101

DCDA -0.31358

DCDC 2.27365

DCDD -0.170452

DCDE -0.199621

DCDF 0.127787

DCDG -0.546709

DCDH 0.269393

DCDI -0.123843

DCDK 0.061641

DCDL -0.337121

DCDM 0.02898

DCDN 0.102967

DCDP -0.323388

DCDQ 0.0348737

DCDR 0.2117

DCDS -0.0665855

DCDT -0.0220714

DCDV -0.199613

DCDW 0.400727

DCDY 0.327062

DCEA -0.425132

DCEC 0.0496862

DCED -0.308578

DCEE -0.237536

DCEF 0.0636353

DCEG -0.814025

DCEH 0.178142

DCEI -0.159483

DCEK 0.0278623

DCEL -0.36746

DCEM 0.0623763

DCEN -0.0940378

DCEP -0.46455

DCEQ 0.0197499

DCER 0.153584

DCES -0.30214

DCET -0.188897

DCEV -0.280773

DCEW 0.425743

DCEY 0.193174

DCFA 0.0764842

DCFC 0.909078

DCFD 0.161645

DCFE 0.0700722

DCFF 0.840492

DCFG -0.283226

DCFH 0.684208

DCFI 0.544734

DCFK 0.149246

DCFL 0.266016

DCFM 0.900953

DCFN 0.244782

DCFP 0.13975

DCFQ 0.369481

DCFR 0.327009

DCFS 0.197789

DCFT 0.280273

DCFV 0.373815

DCFW 1.10357

DCFY 0.815005

DCGA -0.876066

DCGC -0.159574

DCGD -0.518337

DCGE -0.6588

DCGF -0.192852

DCGG -1.77393

DCGH -0.332259

DCGI -0.47702

DCGK -0.504235

DCGL -0.649145

DCGM -0.277685

DCGN -0.409644

DCGP -0.795277

DCGQ -0.398435

DCGR -0.347421

DCGS -0.529774

DCGT -0.397082

DCGV -0.548397

DCGW -0.0271204

DCGY -0.086915

DCHA -0.0262105

DCHC 0.662323

DCHD 0.324969

DCHE 0.194903

DCHF 0.682224

DCHG -0.339817

DCHH 0.749289

DCHI 0.329785

DCHK 0.018012

DCHL 0.29436

DCHM 0.548862

DCHN 0.235923

DCHP 0.0340889

DCHQ 0.21645

DCHR 0.324209

DCHS 0.209957

DCHT 0.283082

DCHV 0.282249

DCHW 0.927622

DCHY 0.736998

DCIA -0.12363

DCIC 0.725298

DCID -0.143099

DCIE -0.157704

DCIF 0.556599

DCIG -0.538583

DCIH 0.387752

DCII 0.35176

DCIK -0.0855169

DCIL 0.00551534

DCIM 0.715214

DCIN 0.00690603

DCIP -0.120216

DCIQ 0.132864

DCIR 0.102535

DCIS -0.0769804

DCIT 0.0797207

DCIV 0.149759

DCIW 0.851683

DCIY 0.590124

DCKA -0.400317

DCKC 0.17896

DCKD 0.00348163

DCKE 0.0135472

DCKF 0.130301

DCKG -0.712956

DCKH -0.00120163

DCKI -0.104253

DCKK -0.141537

DCKL -0.312796

DCKM 0.0362906

DCKN -0.0611906

DCKP -0.386414

DCKQ 0.0432854

DCKR -0.149503

DCKS -0.215171

DCKT -0.119526

DCKV -0.21383

DCKW 0.309128

DCKY 0.277307

DCLA -0.417562

DCLC 0.71204

DCLD -0.369186

DCLE -0.362138

DCLF 0.273807

DCLG -0.722012

DCLH 0.297295

DCLI -0.00377393

DCLK -0.291398

DCLL -0.339406

DCLM 0.521765

DCLN -0.209526

DCLP -0.278682

DCLQ 0.0262077

DCLR -0.0839782

DCLS -0.321783

DCLT -0.200662

DCLV -0.191581

DCLW 0.770258

DCLY 0.315148

DCMA 0.211595

DCMC 0.804179

DCMD 0.0856121

DCME 0.107575

DCMF 0.902577

DCMG -0.450574

DCMH 0.436211

DCMI 0.678366

DCMK 0.12921

DCML 0.518656

DCMM 0.892174

DCMN 0.114082

DCMP -0.0796959

DCMQ 0.268215

DCMR 0.319998

DCMS 0.071089

DCMT 0.255812

DCMV 0.53721

DCMW 0.837778

DCMY 0.831382

DCNA -0.289492

DCNC 0.325106

DCND 0.029331

DCNE -0.084254

DCNF 0.257365

DCNG -0.444716

DCNH 0.256721

DCNI -0.0318217

DCNK 0.0269542

DCNL -0.189832

DCNM 0.124577

DCNN 0.245294

DCNP -0.153075

DCNQ 0.182815

DCNR 0.118403

DCNS 0.00102401

DCNT 0.0578148

DCNV -0.119875

DCNW 0.590032

DCNY 0.445292

DCPA -0.272338

DCPC 0.244566

DCPD -0.0633936

DCPE -0.0333691

DCPF 0.309147

DCPG -0.471103

DCPH 0.220217

DCPI -0.00907922

DCPK -0.121657

DCPL -0.148126

DCPM 0.197198

DCPN 0.00611472

DCPP -0.173426

DCPQ 0.114288

DCPR 0.0920625

DCPS -0.0481706

DCPT 0.00586557

DCPV -0.0123956

DCPW 0.650003

DCPY 0.400225

DCQA -0.119701

DCQC 0.444409

DCQD -0.0211885

DCQE 0.00825858

DCQF 0.411186

DCQG -0.42659

DCQH 0.204045

DCQI 0.0968461

DCQK 0.0760088

DCQL 0.000139

DCQM 0.219515

DCQN 0.154096

DCQP -0.191407

DCQQ 0.376959

DCQR 0.210243

DCQS 0.0389807

DCQT 0.121877

DCQV 0.0215225

DCQW 0.601289

DCQY 0.51537

DCRA -0.233388

DCRC 0.370986

DCRD 0.162177

DCRE 0.153149

DCRF 0.368032

DCRG -0.548273

DCRH 0.30176

DCRI 0.102906

DCRK -0.147092

DCRL -0.0953503

DCRM 0.351282

DCRN 0.0489757

DCRP -0.129155

DCRQ 0.219501

DCRR 0.174071

DCRS -0.0286183

DCRT 0.00054836

DCRV -0.0180352

DCRW 0.582367

DCRY 0.46955

DCSA -0.414433

DCSC 0.394426

DCSD -0.124904

DCSE -0.171552

DCSF 0.206677

DCSG -0.478872

DCSH 0.223637

DCSI -0.114566

DCSK -0.139061

DCSL -0.289521

DCSM 0.126772

DCSN 0.023576

DCSP -0.319623

DCSQ 0.123926

DCSR 0.0263252

DCSS -0.0265927

DCST 0.00638628

DCSV -0.206693

DCSW 0.442524

DCSY 0.298918

DCTA -0.295611

DCTC 0.478114

DCTD -0.0757003

DCTE -0.090703

DCTF 0.311168

DCTG -0.475637

DCTH 0.282675

DCTI 0.0519435

DCTK -0.0871961

DCTL -0.187706

DCTM 0.289459

DCTN 0.0729468

DCTP -0.184343

DCTQ 0.183506

DCTR 0.0797486

DCTS -0.0282588

DCTT 0.0763507

DCTV -0.0516703

DCTW 0.563576

DCTY 0.402879

DCVA -0.274488

DCVC 0.675753

DCVD -0.257918

DCVE -0.273826

DCVF 0.391065

DCVG -0.642048

DCVH 0.288748

DCVI 0.142636

DCVK -0.194827

DCVL -0.187544

DCVM 0.559976

DCVN -0.118471

DCVP -0.219847

DCVQ 0.0326567

DCVR -0.0376966

DCVS -0.207639

DCVT -0.0608914

DCVV -0.0141883

DCVW 0.752256

DCVY 0.399768

DCWA 0.336529

DCWC 1.11455

DCWD 0.348949

DCWE 0.334221

DCWF 1.13813

DCWG 0.0415666

DCWH 0.718887

DCWI 0.898344

DCWK 0.361705

DCWL 0.780353

DCWM 0.935746

DCWN 0.519442

DCWP 0.348957

DCWQ 0.6173

DCWR 0.645236

DCWS 0.367175

DCWT 0.455047

DCWV 0.78943

DCWW 1.27768

DCWY 1.22607

DCYA 0.0847435

DCYC 1.12701

DCYD 0.285806

DCYE 0.163153

DCYF 0.840614

DCYG -0.116534

DCYH 0.685535

DCYI 0.52682

DCYK 0.265637

DCYL 0.280597

DCYM 0.804529

DCYN 0.411727

DCYP 0.287103

DCYQ 0.499308

DCYR 0.471303

DCYS 0.240067

DCYT 0.348365

DCYV 0.374151

DCYW 1.17455

DCYY 0.904985

DDAA -0.803604

DDAC 0.0635436

DDAD -0.593754

DDAE -0.679921

DDAF -0.203812

DDAG -1.12863

DDAH -0.144936

DDAI -0.456979

DDAK -0.644593

DDAL -0.769724

DDAM 0.0101125

DDAN -0.551731

DDAP -0.805592

DDAQ -0.405402

DDAR -0.496095

DDAS -0.690818

DDAT -0.602299

DDAV -0.624243

DDAW 0.206568

DDAY -0.149695

DDCA 0.121411

DDCC 2.13204

DDCD -0.0214741

DDCE -0.0909712

DDCF 0.734509

DDCG -0.154204

DDCH 0.660956

DDCI 0.457198

DDCK 0.117232

DDCL 0.36374

DDCM 0.653084

DDCN 0.191002

DDCP 0.0743101

DDCQ 0.36841

DDCR 0.328523

DDCS 0.182536

DDCT 0.174488

DDCV 0.422812

DDCW 1.04132

DDCY 0.771233

DDDA -0.580971

DDDC -0.00844169

DDDD 4.17359

DDDE -0.0838904

DDDF -0.112239

DDDG -0.721741

DDDH 0.105087

DDDI -0.399692

DDDK -0.134446

DDDL -0.653693

DDDM -0.122673

DDDN 0.280974

DDDP -0.51258

DDDQ -0.106508

DDDR -0.0423403

DDDS -0.258111

DDDT -0.233818

DDDV -0.494822

DDDW 0.266798

DDDY 0.0760627

DDEA -0.73145

DDEC -0.0741208

DDED -0.149228

DDEE -0.396441

DDEF -0.196985

DDEG -1.0018

DDEH -0.0118864

DDEI -0.451446

DDEK -0.26701

DDEL -0.694066

DDEM -0.105026

DDEN -0.349262

DDEP -0.662429

DDEQ -0.214956

DDER -0.16252

DDES -0.561242

DDET -0.44921

DDEV -0.583699

DDEW 0.26659

DDEY -0.084312

DDFA -0.228109

DDFC 0.75147

DDFD -0.0822468

DDFE -0.192347

DDFF 0.529422

DDFG -0.513997

DDFH 0.490893

DDFI 0.230011

DDFK -0.114082

DDFL -0.0721064

DDFM 0.680824

DDFN 0.0159442

DDFP -0.077795

DDFQ 0.144307

DDFR 0.0762644

DDFS -0.0772505

DDFT 0.00134754

DDFV 0.0425222

DDFW 0.916091

DDFY 0.538157

DDGA -1.11968

DDGC -0.266695

DDGD -0.672246

DDGE -0.910938

DDGF -0.430779

DDGG -1.87444

DDGH -0.463088

DDGI -0.730184

DDGK -0.754359

DDGL -0.937191

DDGM -0.412882

DDGN -0.630988

DDGP -0.962686

DDGQ -0.601515

DDGR -0.605726

DDGS -0.785532

DDGT -0.66519

DDGV -0.831956

DDGW -0.143065

DDGY -0.327716

DDHA -0.213311

DDHC 0.591277

DDHD 0.159082

DDHE 0.00666928

DDHF 0.488613

DDHG -0.473342

DDHH 0.634223

DDHI 0.136738

DDHK -0.120329

DDHL 0.034941

DDHM 0.455904

DDHN 0.10545

DDHP -0.0840366

DDHQ 0.105035

DDHR 0.15369

DDHS 0.0393994

DDHT 0.0979421

DDHV 0.0584803

DDHW 0.837954

DDHY 0.554106

DDIA -0.456259

DDIC 0.540008

DDID -0.405129

DDIE -0.443905

DDIF 0.233517

DDIG -0.782047

DDIH 0.184571

DDII 0.0067234

DDIK -0.371231

DDIL -0.356773

DDIM 0.466835

DDIN -0.243842

DDIP -0.353788

DDIQ -0.111158

DDIR -0.178962

DDIS -0.355074

DDIT -0.222462

DDIV -0.196619

DDIW 0.648448

DDIY 0.278133

DDKA -0.67721

DDKC 0.0580959

DDKD -0.249698

DDKE -0.31665

DDKF -0.130984

DDKG -0.935231

DDKH -0.140571

DDKI -0.383976

DDKK -0.397651

DDKL -0.624777

DDKM -0.113449

DDKN -0.304494

DDKP -0.583983

DDKQ -0.194949

DDKR -0.403065

DDKS -0.474513

DDKT -0.391293

DDKV -0.503055

DDKW 0.178221

DDKY 0.00333095

DDLA -0.774923

DDLC 0.453633

DDLD -0.658319

DDLE -0.682458

DDLF -0.0756478

DDLG -1.00974

DDLH 0.0352652

DDLI -0.366719

DDLK -0.620022

DDLL -0.716528

DDLM 0.231436

DDLN -0.49851

DDLP -0.542921

DDLQ -0.267753

DDLR -0.402029

DDLS -0.631902

DDLT -0.52819

DDLV -0.562684

DDLW 0.510435

DDLY -0.026916

DDMA -0.00891948

DDMC 0.727584

DDMD -0.06692

DDME -0.0625379

DDMF 0.681574

DDMG -0.561714

DDMH 0.353624

DDMI 0.438558

DDMK -0.0328789

DDML 0.230487

DDMM 0.766361

DDMN -0.00218105

DDMP -0.178367

DDMQ 0.151197

DDMR 0.159691

DDMS -0.0826972

DDMT 0.0849488

DDMV 0.282725

DDMW 0.760321

DDMY 0.643572

DDNA -0.551231

DDNC 0.195257

DDND 0.103396

DDNE -0.294065

DDNF 0.0233557

DDNG -0.648993

DDNH 0.112864

DDNI -0.259967

DDNK -0.214847

DDNL -0.461239

DDNM 0.00645113

DDNN -0.00115967

DDNP -0.345786

DDNQ -0.00972891

DDNR -0.124938

DDNS -0.234873

DDNT -0.195373

DDNV -0.395464

DDNW 0.461928

DDNY 0.197087

DDPA -0.542137

DDPC 0.146769

DDPD -0.263398

DDPE -0.312678

DDPF 0.0615621

DDPG -0.69278

DDPH 0.0762761

DDPI -0.25146

DDPK -0.358962

DDPL -0.454794

DDPM 0.0680177

DDPN -0.207506

DDPP -0.358847

DDPQ -0.0897057

DDPR -0.146631

DDPS -0.295425

DDPT -0.250352

DDPV -0.294166

DDPW 0.508553

DDPY 0.157773

DDQA -0.377656

DDQC 0.352891

DDQD -0.231298

DDQE -0.245472

DDQF 0.179297

DDQG -0.626294

DDQH 0.0865755

DDQI -0.13794

DDQK -0.168783

DDQL -0.298725

DDQM 0.111331

DDQN -0.0488071

DDQP -0.345423

DDQQ 0.160417

DDQR -0.0203102

DDQS -0.200753

DDQT -0.123271

DDQV -0.240454

DDQW 0.492354

DDQY 0.285923

DDRA -0.527394

DDRC 0.24692

DDRD -0.123906

DDRE -0.159866

DDRF 0.101096

DDRG -0.762108

DDRH 0.134957

DDRI -0.17741

DDRK -0.393928

DDRL -0.421751

DDRM 0.186696

DDRN -0.173815

DDRP -0.336342

DDRQ -0.0144112

DDRR -0.0860052

DDRS -0.299613

DDRT -0.249019

DDRV -0.307698

DDRW 0.442157

DDRY 0.214263

DDSA -0.697461

DDSC 0.23151

DDSD -0.303444

DDSE -0.462415

DDSF -0.0534339

DDSG -0.714208

DDSH 0.0498569

DDSI -0.394069

DDSK -0.410398

DDSL -0.614059

DDSM -0.0212333

DDSN -0.207962

DDSP -0.528532

DDSQ -0.130163

DDSR -0.251329

DDSS -0.32637

DDST -0.30601

DDSV -0.509196

DDSW 0.312876

DDSY 0.0361104

DDTA -0.589235

DDTC 0.325631

DDTD -0.355134

DDTE -0.3433

DDTF 0.027348

DDTG -0.732373

DDTH 0.102227

DDTI -0.240899

DDTK -0.371595

DDTL -0.51658

DDTM 0.111561

DDTN -0.192653

DDTP -0.407616

DDTQ -0.0698769

DDTR -0.198499

DDTS -0.318667

DDTT -0.229773

DDTV -0.364961

DDTW 0.404445

DDTY 0.119012

DDVA -0.611655

DDVC 0.457304

DDVD -0.55578

DDVE -0.582775

DDVF 0.0653889

DDVG -0.90852

DDVH 0.0688262

DDVI -0.207831

DDVK -0.485332

DDVL -0.559394

DDVM 0.302932

DDVN -0.38919

DDVP -0.47255

DDVQ -0.227532

DDVR -0.327135

DDVS -0.513662

DDVT -0.383666

DDVV -0.368433

DDVW 0.528059

DDVY 0.0791681

DDWA 0.155355

DDWC 1.04528

DDWD 0.222025

DDWE 0.188914

DDWF 0.943689

DDWG -0.0819318

DDWH 0.650455

DDWI 0.68793

DDWK 0.224462

DDWL 0.514621

DDWM 0.84987

DDWN 0.398887

DDWP 0.247773

DDWQ 0.506063

DDWR 0.498561

DDWS 0.240058

DDWT 0.31079

DDWV 0.563232

DDWW 1.19743

DDWY 1.03533

DDYA -0.202452

DDYC 0.94682

DDYD 0.104016

DDYE -0.0913115

DDYF 0.543936

DDYG -0.355008

DDYH 0.507076

DDYI 0.22437

DDYK -0.00036264

DDYL -0.0542479

DDYM 0.6168

DDYN 0.174351

DDYP 0.0609393

DDYQ 0.27052

DDYR 0.20373

DDYS -0.006495

DDYT 0.0811839

DDYV 0.0589602

DDYW 0.991933

DDYY 0.631821

DEAA -0.813041

DEAC 0.0208528

DEAD -0.717135

DEAE -0.679519

DEAF -0.250731

DEAG -1.17447

DEAH -0.184216

DEAI -0.506254

DEAK -0.688397

DEAL -0.817839

DEAM -0.0315025

DEAN -0.59916

DEAP -0.854074

DEAQ -0.446052

DEAR -0.53706

DEAS -0.759039

DEAT -0.652069

DEAV -0.667024

DEAW 0.169135

DEAY -0.197779

DECA 0.0777738

DECC 2.09006

DECD -0.0640876

DECE -0.121113

DECF 0.695776

DECG -0.189277

DECH 0.628513

DECI 0.418916

DECK 0.0850935

DECL 0.323105

DECM 0.621676

DECN 0.157032

DECP 0.0417254

DECQ 0.335613

DECR 0.292354

DECS 0.154208

DECT 0.138548

DECV 0.382297

DECW 1.01021

DECY 0.734098

DEDA -0.423285

DEDC -0.0389707

DEDD -0.0852504

DEDE 4.20874

DEDF -0.127017

DEDG -0.798968

DEDH 0.076215

DEDI -0.41861

DEDK -0.0849731

DEDL -0.622344

DEDM -0.142413

DEDN -0.146669

DEDP -0.543739

DEDQ 0.0955622

DEDR -0.0383806

DEDS -0.239657

DEDT -0.170081

DEDV -0.509997

DEDW 0.221388

DEDY 0.0280409

DEEA -0.745422

DEEC -0.107879

DEED -0.595721

DEEE -0.102642

DEEF -0.241848

DEEG -1.06782

DEEH -0.0453885

DEEI -0.499262

DEEK -0.264849

DEEL -0.727076

DEEM -0.130307

DEEN -0.386269

DEEP -0.691813

DEEQ -0.219726

DEER -0.210518

DEES -0.620275

DEET -0.498647

DEEV -0.599

DEEW 0.229798

DEEY -0.113457

DEFA -0.27793

DEFC 0.714244

DEFD -0.138558

DEFE -0.230262

DEFF 0.48926

DEFG -0.557847

DEFH 0.451475

DEFI 0.182773

DEFK -0.161433

DEFL -0.135154

DEFM 0.647215

DEFN -0.01772

DEFP -0.12103

DEFQ 0.10232

DEFR 0.0313139

DEFS -0.12288

DEFT -0.0431609

DEFV -0.00574899

DEFW 0.87644

DEFY 0.476337

DEGA -1.16071

DEGC -0.30039

DEGD -0.814136

DEGE -0.845201

DEGF -0.480072

DEGG -1.9083

DEGH -0.502061

DEGI -0.775176

DEGK -0.821031

DEGL -0.999403

DEGM -0.448097

DEGN -0.675038

DEGP -0.995459

DEGQ -0.634518

DEGR -0.635373

DEGS -0.815191

DEGT -0.701293

DEGV -0.867663

DEGW -0.176455

DEGY -0.370202

DEHA -0.262467

DEHC 0.560464

DEHD 0.109825

DEHE -0.00139356

DEHF 0.449725

DEHG -0.509426

DEHH 0.595198

DEHI 0.0964365

DEHK -0.171581

DEHL -0.0109799

DEHM 0.428644

DEHN 0.0532193

DEHP -0.116717

DEHQ 0.0607615

DEHR 0.124474

DEHS -0.0173571

DEHT 0.0584772

DEHV 0.0247836

DEHW 0.803532

DEHY 0.51142

DEIA -0.499844

DEIC 0.499154

DEID -0.458507

DEIE -0.482645

DEIF 0.185989

DEIG -0.819338

DEIH 0.143013

DEII -0.0451012

DEIK -0.413132

DEIL -0.404657

DEIM 0.442865

DEIN -0.286586

DEIP -0.389127

DEIQ -0.150487

DEIR -0.223295

DEIS -0.407632

DEIT -0.258717

DEIV -0.249318

DEIW 0.609138

DEIY 0.231176

DEKA -0.725987

DEKC 0.0185523

DEKD -0.316929

DEKE -0.278963

DEKF -0.177785

DEKG -0.976687

DEKH -0.190994

DEKI -0.433185

DEKK -0.455434

DEKL -0.678801

DEKM -0.148582

DEKN -0.33642

DEKP -0.626058

DEKQ -0.205544

DEKR -0.441845

DEKS -0.526915

DEKT -0.438671

DEKV -0.566952

DEKW 0.143387

DEKY -0.0387404

DELA -0.820928

DELC 0.410905

DELD -0.724473

DELE -0.723557

DELF -0.124156

DELG -1.04714

DELH -0.00438333

DELI -0.409885

DELK -0.662427

DELL -0.78126

DELM 0.185171

DELN -0.545241

DELP -0.598691

DELQ -0.315584

DELR -0.444496

DELS -0.686953

DELT -0.59191

DELV -0.611837

DELW 0.463405

DELY -0.0731173

DEMA -0.0569875

DEMC 0.695859

DEMD -0.0920541

DEME -0.0916893

DEMF 0.641337

DEMG -0.597895

DEMH 0.321572

DEMI 0.395651

DEMK -0.0717678

DEML 0.204051

DEMM 0.73044

DEMN -0.0414639

DEMP -0.212928

DEMQ 0.115775

DEMR 0.121815

DEMS -0.117673

DEMT 0.0444801

DEMV 0.239504

DEMW 0.728732

DEMY 0.599936

DENA -0.599581

DENC 0.162111

DEND -0.212177

DENE -0.0461919

DENF -0.0122693

DENG -0.696845

DENH 0.0761743

DENI -0.314346

DENK -0.267155

DENL -0.520901

DENM -0.0252032

DENN -0.0126333

DENP -0.374418

DENQ -0.0364428

DENR -0.148634

DENS -0.285867

DENT -0.248218

DENV -0.431311

DENW 0.42586

DENY 0.155655

DEPA -0.586146

DEPC 0.11394

DEPD -0.355198

DEPE -0.303652

DEPF 0.0205579

DEPG -0.735778

DEPH 0.0435417

DEPI -0.298608

DEPK -0.402914

DEPL -0.479538

DEPM 0.0276673

DEPN -0.25205

DEPP -0.404325

DEPQ -0.128631

DEPR -0.19942

DEPS -0.347394

DEPT -0.295422

DEPV -0.353161

DEPW 0.476798

DEPY 0.111324

DEQA -0.427512

DEQC 0.319599

DEQD -0.296094

DEQE -0.201706

DEQF 0.134108

DEQG -0.664006

DEQH 0.0512242

DEQI -0.183592

DEQK -0.211495

DEQL -0.336307

DEQM 0.0722022

DEQN -0.0941782

DEQP -0.387284

DEQQ 0.127757

DEQR -0.0452688

DEQS -0.23694

DEQT -0.16414

DEQV -0.276239

DEQW 0.458464

DEQY 0.242546

DERA -0.574983

DERC 0.212512

DERD -0.188391

DERE -0.15549

DERF 0.0562725

DERG -0.808259

DERH 0.0963809

DERI -0.222357

DERK -0.431575

DERL -0.470348

DERM 0.149145

DERN -0.225503

DERP -0.375888

DERQ -0.0656469

DERR -0.111024

DERS -0.324157

DERT -0.3081

DERV -0.365955

DERW 0.406419

DERY 0.161347

DESA -0.748831

DESC 0.19729

DESD -0.406989

DESE -0.377122

DESF -0.112976

DESG -0.788064

DESH 0.0105956

DESI -0.435616

DESK -0.470022

DESL -0.643738

DESM -0.0765107

DESN -0.272777

DESP -0.562816

DESQ -0.172255

DESR -0.301271

DESS -0.381127

DEST -0.343438

DESV -0.561405

DESW 0.257312

DESY 0.0056324

DETA -0.638465

DETC 0.288243

DETD -0.387529

DETE -0.403561

DETF -0.00579309

DETG -0.771815

DETH 0.0545433

DETI -0.291047

DETK -0.407268

DETL -0.569755

DETM 0.0803683

DETN -0.231925

DETP -0.442672

DETQ -0.101379

DETR -0.244388

DETS -0.370905

DETT -0.285816

DETV -0.409621

DETW 0.367152

DETY 0.0851145

DEVA -0.668997

DEVC 0.416407

DEVD -0.601426

DEVE -0.557832

DEVF 0.0106766

DEVG -0.950867

DEVH 0.0222063

DEVI -0.258337

DEVK -0.537379

DEVL -0.612626

DEVM 0.266365

DEVN -0.435113

DEVP -0.513653

DEVQ -0.277994

DEVR -0.38408

DEVS -0.559143

DEVT -0.417964

DEVV -0.42612

DEVW 0.486505

DEVY 0.0404885

DEWA 0.116915

DEWC 1.01415

DEWD 0.178907

DEWE 0.158838

DEWF 0.905641

DEWG -0.116796

DEWH 0.616202

DEWI 0.649658

DEWK 0.188093

DEWL 0.473894

DEWM 0.816723

DEWN 0.363462

DEWP 0.212723

DEWQ 0.471729

DEWR 0.462569

DEWS 0.192347

DEWT 0.276806

DEWV 0.518026

DEWW 1.16472

DEWY 0.99643

DEYA -0.251874

DEYC 0.90815

DEYD -0.0187445

DEYE -0.123716

DEYF 0.496421

DEYG -0.392109

DEYH 0.469655

DEYI 0.180716

DEYK -0.0456488

DEYL -0.10213

DEYM 0.576771

DEYN 0.133145

DEYP 0.0212135

DEYQ 0.234277

DEYR 0.165159

DEYS -0.0746937

DEYT 0.0365081

DEYV 0.0142953

DEYW 0.951257

DEYY 0.576346

DFAA -0.784801

DFAC 0.105083

DFAD -0.666704

DFAE -0.67437

DFAF -0.0330315

DFAG -1.08309

DFAH -0.0990846

DFAI -0.403367

DFAK -0.575023

DFAL -0.686275

DFAM 0.0608656

DFAN -0.504285

DFAP -0.767711

DFAQ -0.328043

DFAR -0.444285

DFAS -0.671435

DFAT -0.565278

DFAV -0.527663

DFAW 0.247189

DFAY -0.116629

DFCA 0.160903

DFCC 2.18031

DFCD 0.00793338

DFCE -0.0525682

DFCF 0.782388

DFCG -0.115089

DFCH 0.694224

DFCI 0.498994

DFCK 0.154564

DFCL 0.411806

DFCM 0.686325

DFCN 0.226677

DFCP 0.1121

DFCQ 0.404955

DFCR 0.368758

DFCS 0.220745

DFCT 0.215542

DFCV 0.468815

DFCW 1.0745

DFCY 0.810728

DFDA -0.493766

DFDC 0.0226972

DFDD -0.379339

DFDE -0.392757

DFDF 4.36849

DFDG -0.74455

DFDH 0.144347

DFDI -0.186419

DFDK -0.204521

DFDL -0.289904

DFDM -0.00677896

DFDN -0.113856

DFDP -0.485285

DFDQ -0.154449

DFDR -0.0354843

DFDS -0.229304

DFDT -0.13707

DFDV -0.460413

DFDW 0.352666

DFDY 0.679436

DFEA -0.677801

DFEC -0.0391233

DFED -0.523608

DFEE -0.48662

DFEF 0.151046

DFEG -0.984297

DFEH 0.0312936

DFEI -0.413382

DFEK -0.245309

DFEL -0.607554

DFEM -0.0553062

DFEN -0.302578

DFEP -0.619521

DFEQ -0.177769

DFER -0.0916085

DFES -0.533284

DFET -0.427125

DFEV -0.523491

DFEW 0.30705

DFEY 0.042995

DFFA -0.185019

DFFC 0.793829

DFFD -0.0506926

DFFE -0.141438

DFFF 0.595206

DFFG -0.462001

DFFH 0.540544

DFFI 0.282277

DFFK -0.0640085

DFFL -0.0201321

DFFM 0.730576

DFFN 0.064873

DFFP -0.0321784

DFFQ 0.19274

DFFR 0.120975

DFFS -0.0304375

DFFT 0.0521598

DFFV 0.0864751

DFFW 0.959764

DFFY 0.572858

DFGA -1.06969

DFGC -0.230479

DFGD -0.727389

DFGE -0.857604

DFGF -0.296086

DFGG -1.83821

DFGH -0.427383

DFGI -0.684125

DFGK -0.707619

DFGL -0.896391

DFGM -0.373333

DFGN -0.592889

DFGP -0.919583

DFGQ -0.553864

DFGR -0.552632

DFGS -0.726019

DFGT -0.615957

DFGV -0.685496

DFGW -0.10084

DFGY -0.278432

DFHA -0.179039

DFHC 0.625256

DFHD 0.171418

DFHE 0.0467484

DFHF 0.570326

DFHG -0.435787

DFHH 0.66918

DFHI 0.193162

DFHK -0.0933838

DFHL 0.0896814

DFHM 0.49309

DFHN 0.134619

DFHP -0.0486062

DFHQ 0.139625

DFHR 0.198531

DFHS 0.0704291

DFHT 0.12474

DFHV 0.126827

DFHW 0.872303

DFHY 0.612223

DFIA -0.376704

DFIC 0.582744

DFID -0.362141

DFIE -0.39036

DFIF 0.291974

DFIG -0.728307

DFIH 0.226635

DFII 0.0660024

DFIK -0.315485

DFIL -0.274691

DFIM 0.516304

DFIN -0.193283

DFIP -0.302958

DFIQ -0.0506001

DFIR -0.127458

DFIS -0.313038

DFIT -0.176315

DFIV -0.146583

DFIW 0.694102

DFIY 0.333112

DFKA -0.640952

DFKC 0.0919533

DFKD -0.251198

DFKE -0.257468

DFKF 0.0220721

DFKG -0.88803

DFKH -0.0998077

DFKI -0.327182

DFKK -0.370304

DFKL -0.57426

DFKM -0.0606627

DFKN -0.265179

DFKP -0.535833

DFKQ -0.146794

DFKR -0.355287

DFKS -0.439383

DFKT -0.3549

DFKV -0.470339

DFKW 0.217382

DFKY 0.0774105

DFLA -0.72524

DFLC 0.504961

DFLD -0.613503

DFLE -0.627037

DFLF -0.00915051

DFLG -0.962328

DFLH 0.0872238

DFLI -0.302223

DFLK -0.559463

DFLL -0.66956

DFLM 0.283212

DFLN -0.44111

DFLP -0.504094

DFLQ -0.217681

DFLR -0.344567

DFLS -0.596294

DFLT -0.477588

DFLV -0.512039

DFLW 0.556231

DFLY 0.0304537

DFMA 0.0294158

DFMC 0.761634

DFMD -0.0258353

DFME -0.0196068

DFMF 0.736517

DFMG -0.524878

DFMH 0.398911

DFMI 0.485007

DFMK 0.0105996

DFML 0.307617

DFMM 0.810244

DFMN 0.0318818

DFMP -0.142761

DFMQ 0.189574

DFMR 0.201875

DFMS -0.0404665

DFMT 0.130807

DFMV 0.330991

DFMW 0.796282

DFMY 0.686622

DFNA -0.488558

DFNC 0.231034

DFND -0.190581

DFNE -0.299608

DFNF 0.268564

DFNG -0.624722

DFNH 0.155942

DFNI -0.229197

DFNK -0.190172

DFNL -0.367405

DFNM 0.0472388

DFNN 0.0429449

DFNP -0.294605

DFNQ 0.0246482

DFNR -0.0852787

DFNS -0.237398

DFNT -0.157709

DFNV -0.338707

DFNW 0.501147

DFNY 0.255811

DFPA -0.49578

DFPC 0.182301

DFPD -0.264817

DFPE -0.253884

DFPF 0.149703

DFPG -0.644557

DFPH 0.116189

DFPI -0.210336

DFPK -0.315178

DFPL -0.389384

DFPM 0.105355

DFPN -0.160657

DFPP -0.318826

DFPQ -0.0432222

DFPR -0.103505

DFPS -0.251544

DFPT -0.203787

DFPV -0.239002

DFPW 0.551475

DFPY 0.202565

DFQA -0.334135

DFQC 0.389417

DFQD -0.199509

DFQE -0.175504

DFQF 0.285553

DFQG -0.575371

DFQH 0.125192

DFQI -0.0884194

DFQK -0.109631

DFQL -0.248254

DFQM 0.141471

DFQN -6.056e-05

DFQP -0.30441

DFQQ 0.207852

DFQR 0.0273626

DFQS -0.15163

DFQT -0.0727375

DFQV -0.143079

DFQW 0.526618

DFQY 0.331747

DFRA -0.466072

DFRC 0.28808

DFRD -0.0815301

DFRE -0.109689

DFRF 0.160754

DFRG -0.717751

DFRH 0.181363

DFRI -0.123811

DFRK -0.342014

DFRL -0.343237

DFRM 0.227327

DFRN -0.136666

DFRP -0.288994

DFRQ 0.035814

DFRR -0.039238

DFRS -0.250621

DFRT -0.214825

DFRV -0.267084

DFRW 0.482222

DFRY 0.24328

DFSA -0.649014

DFSC 0.272585

DFSD -0.362307

DFSE -0.404163

DFSF 0.140733

DFSG -0.698503

DFSH 0.0758755

DFSI -0.325712

DFSK -0.375025

DFSL -0.559273

DFSM 0.00838447

DFSN -0.224801

DFSP -0.486982

DFSQ -0.085387

DFSR -0.214027

DFSS 0.0154905

DFST -0.236221

DFSV -0.399343

DFSW 0.334032

DFSY 0.171955

DFTA -0.214173

DFTC 0.365797

DFTD -0.311256

DFTE -0.339987

DFTF 0.141305

DFTG -0.692702

DFTH 0.151032

DFTI -0.209439

DFTK -0.310989

DFTL -0.310112

DFTM 0.147671

DFTN -0.146587

DFTP -0.365831

DFTQ -0.0212829

DFTR -0.147687

DFTS -0.34431

DFTT -0.171013

DFTV -0.306374

DFTW 0.446693

DFTY 0.189071

DFVA -0.573883

DFVC 0.503887

DFVD -0.49427

DFVE -0.512624

DFVF 0.160508

DFVG -0.851305

DFVH 0.118072

DFVI -0.154661

DFVK -0.455534

DFVL -0.518475

DFVM 0.348265

DFVN -0.327932

DFVP -0.419823

DFVQ -0.184974

DFVR -0.267018

DFVS -0.454016

DFVT -0.327122

DFVV -0.313603

DFVW 0.571674

DFVY 0.134618

DFWA 0.199401

DFWC 1.07859

DFWD 0.255526

DFWE 0.230982

DFWF 0.991136

DFWG -0.0425446

DFWH 0.684082

DFWI 0.736452

DFWK 0.264763

DFWL 0.566508

DFWM 0.885935

DFWN 0.436948

DFWP 0.283598

DFWQ 0.544127

DFWR 0.539364

DFWS 0.267246

DFWT 0.351365

DFWV 0.607818

DFWW 1.23094

DFWY 1.08033

DFYA -0.153088

DFYC 0.990941

DFYD 0.0673232

DFYE -0.0449986

DFYF 0.632668

DFYG -0.308092

DFYH 0.557926

DFYI 0.276156

DFYK 0.0469201

DFYL 0.00695372

DFYM 0.655071

DFYN 0.214896

DFYP 0.113608

DFYQ 0.319453

DFYR 0.25496

DFYS 0.0894079

DFYT 0.121686

DFYV 0.120054

DFYW 1.03112

DFYY 0.673128

DGAA -0.706605

DGAC 0.128416

DGAD -0.643775

DGAE -0.633787

DGAF -0.114796

DGAG -0.995281

DGAH -0.075495

DGAI -0.366807

DGAK -0.566279

DGAL -0.673903

DGAM 0.0912311

DGAN -0.447814

DGAP -0.725351

DGAQ -0.327558

DGAR -0.383296

DGAS -0.628225

DGAT -0.519509

DGAV -0.529709

DGAW 0.278084

DGAY -0.0657406

DGCA 0.189156

DGCC 2.20939

DGCD 0.0288942

DGCE -0.0312397

DGCF 0.801477

DGCG -0.0927112

DGCH 0.713587

DGCI 0.525725

DGCK 0.177164

DGCL 0.441567

DGCM 0.705467

DGCN 0.247361

DGCP 0.129213

DGCQ 0.425791

DGCR 0.390694

DGCS 0.246644

DGCT 0.236324

DGCV 0.496756

DGCW 1.09349

DGCY 0.835541

DGDA -0.365431

DGDC 0.0470011

DGDD -0.290041

DGDE -0.365909

DGDF -0.0457506

DGDG 3.63445

DGDH 0.167807

DGDI -0.325642

DGDK -0.120162

DGDL -0.540546

DGDM -0.0476773

DGDN 0.0200741

DGDP -0.439433

DGDQ -0.116019

DGDR 0.0200734

DGDS -0.193514

DGDT -0.241413

DGDV -0.43698

DGDW 0.325943

DGDY 0.141079

DGEA -0.619219

DGEC -0.0161135

DGED -0.493042

DGEE -0.444112

DGEF -0.117011

DGEG -0.790349

DGEH 0.0611131

DGEI -0.36837

DGEK -0.183738

DGEL -0.599444

DGEM -0.0355527

DGEN -0.26566

DGEP -0.592382

DGEQ -0.140457

DGER -0.0793576

DGES -0.486533

DGET -0.381585

DGEV -0.494649

DGEW 0.333132

DGEY 0.00559139

DGFA -0.144446

DGFC 0.82018

DGFD -0.0220547

DGFE -0.11356

DGFF 0.61586

DGFG -0.440406

DGFH 0.562863

DGFI 0.313097

DGFK -0.03246

DGFL 0.0104322

DGFM 0.757076

DGFN 0.0914776

DGFP -0.00453472

DGFQ 0.223107

DGFR 0.14811

DGFS 0.0105271

DGFT 0.0855069

DGFV 0.133462

DGFW 0.986928

DGFY 0.608054

DGGA -1.03675

DGGC -0.208736

DGGD -0.664786

DGGE -0.822102

DGGF -0.358253

DGGG -1.81277

DGGH -0.405215

DGGI -0.646077

DGGK -0.67138

DGGL -0.863255

DGGM -0.350207

DGGN -0.520534

DGGP -0.896416

DGGQ -0.522124

DGGR -0.523706

DGGS -0.698046

DGGT -0.581185

DGGV -0.747641

DGGW -0.0816696

DGGY -0.247042

DGHA -0.141943

DGHC 0.644872

DGHD 0.201509

DGHE 0.0754988

DGHF 0.56044

DGHG -0.391818

DGHH 0.688672

DGHI 0.2094

DGHK -0.0704641

DGHL 0.115281

DGHM 0.512097

DGHN 0.155622

DGHP -0.0241845

DGHQ 0.159115

DGHR 0.222282

DGHS 0.0959494

DGHT 0.170345

DGHV 0.137268

DGHW 0.892689

DGHY 0.622119

DGIA -0.368228

DGIC 0.609347

DGID -0.316692

DGIE -0.355866

DGIF 0.323248

DGIG -0.683227

DGIH 0.256124

DGII 0.0973339

DGIK -0.289738

DGIL -0.26268

DGIM 0.54799

DGIN -0.154783

DGIP -0.277247

DGIQ -0.0359774

DGIR -0.0947473

DGIS -0.280782

DGIT -0.133615

DGIV -0.106551

DGIW 0.722672

DGIY 0.366197

DGKA -0.614383

DGKC 0.118188

DGKD -0.197094

DGKE -0.216101

DGKF -0.0492566

DGKG -0.823972

DGKH -0.0811954

DGKI -0.297428

DGKK -0.34014

DGKL -0.54061

DGKM -0.0428028

DGKN -0.229938

DGKP -0.507358

DGKQ -0.116056

DGKR -0.320384

DGKS -0.412218

DGKT -0.318446

DGKV -0.429479

DGKW 0.243946

DGKY 0.0869448

DGLA -0.686147

DGLC 0.534264

DGLD -0.588623

DGLE -0.599153

DGLF 0.0210533

DGLG -0.91333

DGLH 0.117788

DGLI -0.27211

DGLK -0.520976

DGLL -0.618125

DGLM 0.317202

DGLN -0.403802

DGLP -0.476165

DGLQ -0.18636

DGLR -0.319193

DGLS -0.529133

DGLT -0.437385

DGLV -0.465322

DGLW 0.588473

DGLY 0.0668592

DGMA 0.0626166

DGMC 0.78116

DGMD -0.00130677

DGME 0.00519729

DGMF 0.758046

DGMG -0.492975

DGMH 0.408254

DGMI 0.516774

DGMK 0.0351615

DGML 0.315012

DGMM 0.828044

DGMN 0.0548215

DGMP -0.114784

DGMQ 0.211727

DGMR 0.228657

DGMS -0.0163677

DGMT 0.151395

DGMV 0.362635

DGMW 0.814036

DGMY 0.711197

DGNA -0.445435

DGNC 0.251623

DGND -0.129612

DGNE -0.259779

DGNF 0.0991223

DGNG -0.210179

DGNH 0.172666

DGNI -0.196068

DGNK -0.153562

DGNL -0.40289

DGNM 0.0651183

DGNN 0.0771379

DGNP -0.271116

DGNQ 0.056138

DGNR -0.0409606

DGNS -0.179479

DGNT -0.11199

DGNV -0.288651

DGNW 0.522151

DGNY 0.266543

DGPA -0.456824

DGPC 0.205386

DGPD -0.223922

DGPE -0.223441

DGPF 0.141284

DGPG -0.612935

DGPH 0.142631

DGPI -0.180658

DGPK -0.27425

DGPL -0.364654

DGPM 0.127743

DGPN -0.120498

DGPP -0.293011

DGPQ -0.0184643

DGPR -0.0735912

DGPS -0.208748

DGPT -0.170209

DGPV -0.217057

DGPW 0.57312

DGPY 0.237425

DGQA -0.305057

DGQC 0.409648

DGQD -0.172741

DGQE -0.130803

DGQF 0.25723

DGQG -0.572938

DGQH 0.148163

DGQI -0.0601025

DGQK -0.0838795

DGQL -0.204375

DGQM 0.166563

DGQN 0.0203528

DGQP -0.28204

DGQQ 0.238532

DGQR 0.0531442

DGQS -0.116347

DGQT -0.040539

DGQV -0.159523

DGQW 0.552619

DGQY 0.361939

DGRA -0.428651

DGRC 0.306947

DGRD -0.0498624

DGRE -0.0742006

DGRF 0.183478

DGRG -0.655737

DGRH 0.204895

DGRI -0.0934694

DGRK -0.313359

DGRL -0.331106

DGRM 0.254624

DGRN -0.0976274

DGRP -0.262125

DGRQ 0.0625494

DGRR -0.0126061

DGRS -0.210803

DGRT -0.182433

DGRV -0.23137

DGRW 0.505975

DGRY 0.287126

DGSA -0.605891

DGSC 0.296469

DGSD -0.29665

DGSE -0.384126

DGSF 0.0119009

DGSG -0.512879

DGSH 0.13939

DGSI -0.309969

DGSK -0.331674

DGSL -0.526244

DGSM 0.0336378

DGSN -0.147166

DGSP -0.467981

DGSQ -0.0629065

DGSR -0.162569

DGSS -0.236135

DGST -0.208975

DGSV -0.429121

DGSW 0.360828

DGSY 0.116605

DGTA -0.518508

DGTC 0.390651

DGTD -0.26458

DGTE -0.307416

DGTF 0.111831

DGTG -0.481208

DGTH 0.175908

DGTI -0.17048

DGTK -0.286996

DGTL -0.433097

DGTM 0.181461

DGTN -0.113489

DGTP -0.329283

DGTQ 0.015641

DGTR -0.115415

DGTS -0.233761

DGTT -0.132245

DGTV -0.27514

DGTW 0.475253

DGTY 0.207954

DGVA -0.526105

DGVC 0.531875

DGVD -0.465508

DGVE -0.488492

DGVF 0.150528

DGVG -0.711718

DGVH 0.142866

DGVI -0.112809

DGVK -0.418307

DGVL -0.463354

DGVM 0.382929

DGVN -0.302357

DGVP -0.39071

DGVQ -0.154693

DGVR -0.248895

DGVS -0.416842

DGVT -0.293591

DGVV -0.279644

DGVW 0.604141

DGVY 0.175712

DGWA 0.226716

DGWC 1.09744

DGWD 0.284748

DGWE 0.253787

DGWF 1.01654

DGWG -0.0192573

DGWH 0.699256

DGWI 0.762014

DGWK 0.287504

DGWL 0.597468

DGWM 0.904907

DGWN 0.463167

DGWP 0.304427

DGWQ 0.565819

DGWR 0.564229

DGWS 0.293364

DGWT 0.376233

DGWV 0.636443

DGWW 1.25183

DGWY 1.10675

DGYA -0.1182

DGYC 1.01619

DGYD 0.101436

DGYE -0.0157051

DGYF 0.630504

DGYG -0.261875

DGYH 0.578126

DGYI 0.31282

DGYK 0.0793216

DGYL 0.0381131

DGYM 0.688797

DGYN 0.249858

DGYP 0.138447

DGYQ 0.349233

DGYR 0.28358

DGYS 0.0669246

DGYT 0.160521

DGYV 0.148872

DGYW 1.06265

DGYY 0.706893

DHAA -0.569448

DHAC 0.211245

DHAD -0.509647

DHAE -0.507917

DHAF 0.0263371

DHAG -0.940086

DHAH 0.0311458

DHAI -0.227097

DHAK -0.435663

DHAL -0.524341

DHAM 0.198388

DHAN -0.354143

DHAP -0.627971

DHAQ -0.210966

DHAR -0.280334

DHAS -0.503903

DHAT -0.382191

DHAV -0.382452

DHAW 0.370178

DHAY 0.0671

DHCA 0.27418

DHCC 2.31857

DHCD 0.0887191

DHCE 0.030978

DHCF 0.883248

DHCG -0.0286005

DHCH 0.763216

DHCI 0.609993

DHCK 0.245352

DHCL 0.549713

DHCM 0.752189

DHCN 0.304616

DHCP 0.182703

DHCQ 0.482835

DHCR 0.461633

DHCS 0.318669

DHCT 0.304641

DHCV 0.599286

DHCW 1.13976

DHCY 0.91205

DHDA -0.416736

DHDC 0.108553

DHDD -0.217763

DHDE -0.245275

DHDF 0.0885968

DHDG -0.586742

DHDH 3.89339

DHDI -0.201713

DHDK 0.0643055

DHDL -0.415461

DHDM 0.0394995

DHDN 0.118195

DHDP -0.357439

DHDQ 0.0698655

DHDR 0.220332

DHDS -0.155568

DHDT -0.0769172

DHDV -0.293844

DHDW 0.403092

DHDY 0.369303

DHEA -0.495999

DHEC 0.044287

DHED -0.369146

DHEE -0.305556

DHEF -0.0006938

DHEG -0.857668

DHEH 0.281072

DHEI -0.242773

DHEK -0.0368259

DHEL -0.45532

DHEM 0.0497668

DHEN -0.150116

DHEP -0.493003

DHEQ -0.0328724

DHER 0.0982065

DHES -0.368701

DHET -0.265691

DHEV -0.364743

DHEW 0.415348

DHEY 0.133642

DHFA -0.0122385

DHFC 0.900735

DHFD 0.103079

DHFE 0.00347662

DHFF 0.749325

DHFG -0.331382

DHFH 0.65572

DHFI 0.449939

DHFK 0.0841568

DHFL 0.155341

DHFM 0.859469

DHFN 0.197244

DHFP 0.0977721

DHFQ 0.32609

DHFR 0.266212

DHFS 0.126649

DHFT 0.20753

DHFV 0.26855

DHFW 1.07845

DHFY 0.740314

DHGA -0.930332

DHGC -0.149138

DHGD -0.581707

DHGE -0.711009

DHGF -0.248057

DHGG -1.75763

DHGH -0.316654

DHGI -0.537151

DHGK -0.568328

DHGL -0.734046

DHGM -0.277289

DHGN -0.439779

DHGP -0.811852

DHGQ -0.431117

DHGR -0.407443

DHGS -0.592728

DHGT -0.463817

DHGV -0.622483

DHGW -0.0171888

DHGY -0.137877

DHHA -0.0558655

DHHC 0.690427

DHHD 0.293998

DHHE 0.164418

DHHF 0.657001

DHHG -0.340022

DHHH 0.766425

DHHI 0.301295

DHHK 0.0093255

DHHL 0.233162

DHHM 0.568895

DHHN 0.237379

DHHP 0.0408685

DHHQ 0.225386

DHHR 0.308392

DHHS 0.186116

DHHT 0.254055

DHHV 0.238864

DHHW 0.94772

DHHY 0.709594

DHIA -0.224623

DHIC 0.699569

DHID -0.212541

DHIE -0.230156

DHIF 0.460495

DHIG -0.593092

DHIH 0.356026

DHII 0.242707

DHIK -0.14958

DHIL -0.112216

DHIM 0.666444

DHIN -0.0522275

DHIP -0.162278

DHIQ 0.0826654

DHIR 0.0275109

DHIS -0.153883

DHIT -0.00925469

DHIV 0.0397549

DHIW 0.818005

DHIY 0.501608

DHKA -0.477522

DHKC 0.180449

DHKD -0.0758226

DHKE -0.0748441

DHKF 0.0681541

DHKG -0.750048

DHKH 0.0263901

DHKI -0.178246

DHKK -0.22158

DHKL -0.408787

DHKM 0.0291948

DHKN -0.115323

DHKP -0.416964

DHKQ -0.00957298

DHKR -0.203521

DHKS -0.282996

DHKT -0.180801

DHKV -0.295753

DHKW 0.311701

DHKY 0.206612

DHLA -0.527819

DHLC 0.650076

DHLD -0.458589

DHLE -0.45595

DHLF 0.163226

DHLG -0.799039

DHLH 0.247021

DHLI -0.12131

DHLK -0.372343

DHLL -0.461911

DHLM 0.442462

DHLN -0.286529

DHLP -0.354591

DHLQ -0.0559571

DHLR -0.183619

DHLS -0.415959

DHLT -0.300241

DHLV -0.310981

DHLW 0.705895

DHLY 0.210804

DHMA 0.166456

DHMC 0.830667

DHMD 0.0768878

DHME 0.0918524

DHMF 0.860595

DHMG -0.439217

DHMH 0.462504

DHMI 0.625751

DHMK 0.117064

DHML 0.439536

DHMM 0.896733

DHMN 0.121122

DHMP -0.0630257

DHMQ 0.276901

DHMR 0.307735

DHMS 0.0622437

DHMT 0.236111

DHMV 0.47683

DHMW 0.86384

DHMY 0.802569

DHNA -0.350627

DHNC 0.309409

DHND -0.0254257

DHNE -0.151008

DHNF 0.206875

DHNG -0.49265

DHNH 0.307645

DHNI -0.0855088

DHNK -0.0230598

DHNL -0.262316

DHNM 0.130675

DHNN 0.196056

DHNP -0.178222

DHNQ 0.161373

DHNR 0.0735552

DHNS -0.0672076

DHNT 0.00683045

DHNV -0.181543

DHNW 0.593535

DHNY 0.391036

DHPA -0.336762

DHPC 0.259639

DHPD -0.120054

DHPE -0.100545

DHPF 0.252456

DHPG -0.51501

DHPH 0.218795

DHPI -0.0685031

DHPK -0.170573

DHPL -0.232238

DHPM 0.20113

DHPN -0.0335371

DHPP -0.200952

DHPQ 0.0784247

DHPR 0.036593

DHPS -0.113791

DHPT -0.0543423

DHPV -0.0899026

DHPW 0.647327

DHPY 0.343912

DHQA -0.188169

DHQC 0.466982

DHQD -0.0652897

DHQE -0.0519822

DHQF 0.361692

DHQG -0.456168

DHQH 0.217275

DHQI 0.0462537

DHQK 0.0243835

DHQL -0.0789869

DHQM 0.229257

DHQN 0.117049

DHQP -0.202521

DHQQ 0.334275

DHQR 0.160609

DHQS -0.0119021

DHQT 0.0696189

DHQV -0.0401931

DHQW 0.613828

DHQY 0.469212

DHRA -0.312396

DHRC 0.371774

DHRD 0.0772324

DHRE 0.0601244

DHRF 0.30175

DHRG -0.589576

DHRH 0.288689

DHRI 0.0285289

DHRK -0.19975

DHRL -0.190944

DHRM 0.337083

DHRN 0.0019052

DHRP -0.165878

DHRQ 0.180682

DHRR 0.111991

DHRS -0.0971818

DHRT -0.0637116

DHRV -0.101844

DHRW 0.581635

DHRY 0.407815

DHSA -0.500121

DHSC 0.370723

DHSD -0.199389

DHSE -0.246452

DHSF 0.130186

DHSG -0.546322

DHSH 0.221718

DHSI -0.187314

DHSK -0.217847

DHSL -0.392792

DHSM 0.115931

DHSN -0.0328062

DHSP -0.358446

DHSQ 0.0621884

DHSR -0.0471377

DHSS 0.0128665

DHST -0.104169

DHSV -0.295076

DHSW 0.439629

DHSY 0.235845

DHTA -0.379841

DHTC 0.468014

DHTD -0.15347

DHTE -0.156934

DHTF 0.23891

DHTG -0.536273

DHTH 0.27636

DHTI -0.026159

DHTK -0.167715

DHTL -0.284543

DHTM 0.268391

DHTN 0.00236821

DHTP -0.225014

DHTQ 0.131407

DHTR 0.0106068

DHTS -0.129601

DHTT 0.00096035

DHTV -0.144097

DHTW 0.552217

DHTY 0.337986

DHVA -0.370996

DHVC 0.631898

DHVD -0.335764

DHVE -0.356143

DHVF 0.290454

DHVG -0.706853

DHVH 0.246705

DHVI 0.0323524

DHVK -0.289541

DHVL -0.309277

DHVM 0.497037

DHVN -0.186437

DHVP -0.274697

DHVQ -0.0333037

DHVR -0.117808

DHVS -0.288146

DHVT -0.157166

DHVV -0.129601

DHVW 0.70763

DHVY 0.314821

DHWA 0.314183

DHWC 1.14391

DHWD 0.350147

DHWE 0.329443

DHWF 1.10937

DHWG 0.0481107

DHWH 0.747567

DHWI 0.860553

DHWK 0.360614

DHWL 0.718708

DHWM 0.957882

DHWN 0.526876

DHWP 0.364319

DHWQ 0.630476

DHWR 0.638771

DHWS 0.365011

DHWT 0.451096

DHWV 0.741319

DHWW 1.30241

DHWY 1.1989

DHYA 0.00432253

DHYC 1.10425

DHYD 0.218915

DHYE 0.100228

DHYF 0.758228

DHYG -0.167121

DHYH 0.672873

DHYI 0.442806

DHYK 0.199979

DHYL 0.179766

DHYM 0.77809

DHYN 0.364296

DHYP 0.240619

DHYQ 0.451656

DHYR 0.400669

DHYS 0.177685

DHYT 0.276886

DHYV 0.284582

DHYW 1.15013

DHYY 0.831747

DIAA -0.798872

DIAC 0.0644786

DIAD -0.713521

DIAE -0.715361

DIAF -0.191489

DIAG -1.12604

DIAH -0.14873

DIAI -0.391801

DIAK -0.639964

DIAL -0.756619

DIAM 0.0145657

DIAN -0.550813

DIAP -0.562751

DIAQ -0.394377

DIAR -0.494986

DIAS -0.693961

DIAT -0.596108

DIAV -0.622784

DIAW 0.210993

DIAY -0.155729

DICA 0.123353

DICC 2.13854

DICD -0.0235584

DICE -0.083955

DICF 0.743144

DICG -0.148925

DICH 0.667529

DICI 0.461453

DICK 0.124898

DICL 0.370503

DICM 0.657449

DICN 0.195543

DICP 0.0777514

DICQ 0.375528

DICR 0.333864

DICS 0.187809

DICT 0.179987

DICV 0.431502

DICW 1.04605

DICY 0.776503

DIDA -0.565573

DIDC 0.00996709

DIDD -0.427892

DIDE -0.44545

DIDF 0.0524807

DIDG -0.785542

DIDH 0.0929372

DIDI 4.18458

DIDK -0.254775

DIDL -0.290795

DIDM 0.035269

DIDN -0.132812

DIDP -0.509824

DIDQ -0.168137

DIDR -0.0569043

DIDS -0.286399

DIDT -0.0354567

DIDV 0.271095

DIDW 0.276837

DIDY 0.115409

DIEA -0.719352

DIEC -0.069648

DIED -0.56474

DIEE -0.513202

DIEF -0.195771

DIEG -1.0274

DIEH -0.00751472

DIEI -0.179295

DIEK -0.321495

DIEL -0.554686

DIEM -0.102562

DIEN -0.337745

DIEP -0.627823

DIEQ -0.221543

DIER -0.149022

DIES -0.561114

DIET -0.456596

DIEV -0.290992

DIEW 0.275749

DIEY -0.0648136

DIFA -0.221046

DIFC 0.757451

DIFD -0.0952082

DIFE -0.187734

DIFF 0.533069

DIFG -0.503313

DIFH 0.497768

DIFI 0.267708

DIFK -0.107262

DIFL -0.0690956

DIFM 0.684633

DIFN 0.0245121

DIFP -0.0759039

DIFQ 0.153111

DIFR 0.0748706

DIFS -0.0711298

DIFT 0.0124388

DIFV 0.0475962

DIFW 0.921691

DIFY 0.533891

DIGA -1.12178

DIGC -0.261564

DIGD -0.763708

DIGE -0.88904

DIGF -0.429244

DIGG -1.86911

DIGH -0.461393

DIGI -0.661654

DIGK -0.759351

DIGL -0.973037

DIGM -0.406087

DIGN -0.628583

DIGP -0.943131

DIGQ -0.590029

DIGR -0.592373

DIGS -0.776183

DIGT -0.659481

DIGV -0.830525

DIGW -0.135541

DIGY -0.325414

DIHA -0.220167

DIHC 0.599258

DIHD 0.134001

DIHE 0.020077

DIHF 0.496419

DIHG -0.466228

DIHH 0.63288

DIHI 0.165285

DIHK -0.130619

DIHL 0.0458319

DIHM 0.468849

DIHN 0.0981197

DIHP -0.0779011

DIHQ 0.105396

DIHR 0.161385

DIHS 0.0345852

DIHT 0.111355

DIHV 0.0922575

DIHW 0.843748

DIHY 0.561472

DIIA -0.440369

DIIC 0.544645

DIID -0.406214

DIIE -0.433054

DIIF 0.24641

DIIG -0.773431

DIIH 0.196028

DIII 0.0300732

DIIK -0.361658

DIIL -0.338509

DIIM 0.473749

DIIN -0.234152

DIIP -0.347757

DIIQ -0.101737

DIIR -0.15874

DIIS -0.351618

DIIT -0.213106

DIIV -0.192564

DIIW 0.659013

DIIY 0.282448

DIKA -0.685033

DIKC 0.057272

DIKD -0.299618

DIKE -0.297817

DIKF -0.134842

DIKG -0.923158

DIKH -0.146524

DIKI -0.350716

DIKK -0.417303

DIKL -0.627998

DIKM -0.102221

DIKN -0.285062

DIKP -0.596786

DIKQ -0.187675

DIKR -0.389486

DIKS -0.478576

DIKT -0.374159

DIKV -0.526451

DIKW 0.189632

DIKY 0.0157211

DILA -0.757019

DILC 0.461299

DILD -0.669062

DILE -0.679359

DILF -0.0710001

DILG -0.995532

DILH 0.0431483

DILI -0.330048

DILK -0.604555

DILL -0.706806

DILM 0.239527

DILN -0.483787

DILP -0.559763

DILQ -0.26026

DILR -0.403427

DILS -0.622363

DILT -0.523479

DILV -0.569311

DILW 0.514601

DILY -0.0176492

DIMA -0.0108531

DIMC 0.732632

DIMD -0.060817

DIME -0.0573437

DIMF 0.688793

DIMG -0.555388

DIMH 0.358917

DIMI 0.438503

DIMK -0.0259972

DIML 0.235541

DIMM 0.769999

DIMN 0.00011015

DIMP -0.170589

DIMQ 0.158411

DIMR 0.167732

DIMS -0.075484

DIMT 0.0874202

DIMV 0.290157

DIMW 0.76512

DIMY 0.646418

DINA -0.539461

DINC 0.19977

DIND -0.22657

DINE -0.335043

DINF 0.0368807

DING -0.664034

DINH 0.113969

DINI -0.0260849

DINK -0.213284

DINL -0.457509

DINM 0.0112743

DINN -0.00532198

DINP -0.339294

DINQ -0.013504

DINR -0.119975

DINS -0.24047

DINT -0.13087

DINV -0.365565

DINW 0.463647

DINY 0.299452

DIPA -0.543162

DIPC 0.151537

DIPD -0.308235

DIPE -0.296736

DIPF 0.0685706

DIPG -0.685382

DIPH 0.0808318

DIPI -0.254055

DIPK -0.354518

DIPL -0.447073

DIPM 0.0695536

DIPN -0.199542

DIPP -0.266255

DIPQ -0.0822589

DIPR -0.14221

DIPS -0.302319

DIPT -0.233501

DIPV -0.299803

DIPW 0.517056

DIPY 0.163802

DIQA -0.369937

DIQC 0.358419

DIQD -0.240945

DIQE -0.233774

DIQF 0.182263

DIQG -0.614221

DIQH 0.0914402

DIQI -0.00469828

DIQK -0.14876

DIQL -0.318255

DIQM 0.0977602

DIQN -0.0482898

DIQP -0.348118

DIQQ 0.159441

DIQR -0.00608754

DIQS -0.180414

DIQT -0.123823

DIQV -0.0644784

DIQW 0.49721

DIQY 0.293021

DIRA -0.497381

DIRC 0.255908

DIRD -0.128931

DIRE -0.154043

DIRF 0.111455

DIRG -0.759491

DIRH 0.142813

DIRI -0.133173

DIRK -0.389168

DIRL -0.449138

DIRM 0.188792

DIRN -0.185783

DIRP -0.339312

DIRQ -0.00391316

DIRR -0.0895696

DIRS -0.284243

DIRT -0.25282

DIRV -0.3083

DIRW 0.447757

DIRY 0.207052

DISA -0.694627

DISC 0.241269

DISD -0.397549

DISE -0.454856

DISF -0.0291657

DISG -0.745628

DISH 0.042186

DISI -0.252279

DISK -0.416262

DISL -0.525712

DISM -0.0298984

DISN -0.230352

DISP -0.508851

DISQ -0.119961

DISR -0.23974

DISS -0.326218

DIST -0.292752

DISV -0.393751

DISW 0.297914

DISY 0.0510449

DITA -0.60704

DITC 0.329197

DITD -0.353157

DITE -0.379267

DITF 0.0424132

DITG -0.724207

DITH 0.108701

DITI -0.186369

DITK -0.364981

DITL -0.540618

DITM 0.112301

DITN -0.185071

DITP -0.400103

DITQ -0.0594213

DITR -0.192586

DITS -0.316025

DITT -0.227399

DITV -0.374656

DITW 0.411687

DITY 0.171043

DIVA -0.613112

DIVC 0.46343

DIVD -0.539809

DIVE -0.570009

DIVF 0.0657218

DIVG -0.897879

DIVH 0.0766811

DIVI -0.158488

DIVK -0.486532

DIVL -0.541809

DIVM 0.302577

DIVN -0.374784

DIVP -0.477584

DIVQ -0.226451

DIVR -0.32744

DIVS -0.500407

DIVT -0.377702

DIVV -0.365548

DIVW 0.535036

DIVY 0.0884359

DIWA 0.162909

DIWC 1.05003

DIWD 0.222823

DIWE 0.195349

DIWF 0.953505

DIWG -0.0769751

DIWH 0.651385

DIWI 0.70942

DIWK 0.230301

DIWL 0.520903

DIWM 0.856508

DIWN 0.404637

DIWP 0.251518

DIWQ 0.514716

DIWR 0.501364

DIWS 0.234241

DIWT 0.317877

DIWV 0.563327

DIWW 1.2015

DIWY 1.04156

DIYA -0.199148

DIYC 0.953197

DIYD 0.0264702

DIYE -0.088727

DIYF 0.552888

DIYG -0.344824

DIYH 0.51355

DIYI 0.269419

DIYK 0.00287032

DIYL -0.0405598

DIYM 0.617883

DIYN 0.177501

DIYP 0.0648098

DIYQ 0.279892

DIYR 0.210219

DIYS -0.0211382

DIYT 0.0901151

DIYV 0.0659029

DIYW 1.00049

DIYY 0.622352

DKAA -1.04321

DKAC -0.13663

DKAD -0.940187

DKAE -0.900768

DKAF -0.431645

DKAG -1.33416

DKAH -0.360768

DKAI -0.692881

DKAK -0.642754

DKAL -1.01543

DKAM -0.196618

DKAN -0.784553

DKAP -1.0151

DKAQ -0.620306

DKAR -0.6874

DKAS -0.929585

DKAT -0.823644

DKAV -0.849249

DKAW 0.00681996

DKAY -0.367599

DKCA -0.0780628

DKCC 1.91838

DKCD -0.204247

DKCE -0.270237

DKCF 0.541897

DKCG -0.336699

DKCH 0.490453

DKCI 0.26079

DKCK -0.0657549

DKCL 0.15086

DKCM 0.484991

DKCN 0.0136862

DKCP -0.099282

DKCQ 0.192475

DKCR 0.143148

DKCS -0.005656

DKCT -0.00933623

DKCV 0.21738

DKCW 0.87359

DKCY 0.581125

DKDA -0.78932

DKDC -0.185459

DKDD -0.543557

DKDE -0.492723

DKDF -0.346532

DKDG -0.960972

DKDH -0.0219548

DKDI -0.635685

DKDK 4.64726

DKDL -0.820379

DKDM -0.259166

DKDN -0.0250471

DKDP -0.715998

DKDQ -0.124791

DKDR 0.356374

DKDS -0.197244

DKDT -0.24666

DKDV -0.695886

DKDW 0.0665452

DKDY -0.177782

DKEA -0.831352

DKEC -0.254526

DKED -0.798186

DKEE -0.678663

DKEF -0.417836

DKEG -1.23996

DKEH -0.18052

DKEI -0.668176

DKEK 0.339023

DKEL -0.874567

DKEM -0.296991

DKEN -0.548364

DKEP -0.862533

DKEQ -0.369153

DKER -0.281397

DKES -0.808858

DKET -0.669715

DKEV -0.799688

DKEW 0.0755007

DKEY -0.284878

DKFA -0.470165

DKFC 0.557611

DKFD -0.316685

DKFE -0.422044

DKFF 0.300176

DKFG -0.374469

DKFH 0.288997

DKFI -0.00230765

DKFK -0.33311

DKFL -0.327816

DKFM 0.47326

DKFN -0.197767

DKFP -0.26209

DKFQ -0.0646112

DKFR -0.157817

DKFS -0.307897

DKFT -0.224299

DKFV -0.186405

DKFW 0.715248

DKFY 0.302397

DKGA -1.335

DKGC -0.445345

DKGD -0.996511

DKGE -1.11308

DKGF -0.64685

DKGG -2.05288

DKGH -0.651994

DKGI -0.917064

DKGK -0.818249

DKGL -1.18085

DKGM -0.600815

DKGN -0.833257

DKGP -1.15835

DKGQ -0.813571

DKGR -0.731611

DKGS -1.01587

DKGT -0.873512

DKGV -1.05535

DKGW -0.325052

DKGY -0.547882

DKHA -0.428195

DKHC 0.423071

DKHD -0.0627367

DKHE -0.203206

DKHF 0.29135

DKHG -0.6611

DKHH 0.44838

DKHI -0.0596218

DKHK -0.267589

DKHL -0.19299

DKHM 0.283862

DKHN -0.0772619

DKHP -0.269542

DKHQ -0.0862856

DKHR -0.0192745

DKHS -0.160285

DKHT -0.105538

DKHV -0.131529

DKHW 0.66025

DKHY 0.345316

DKIA -0.699403

DKIC 0.338237

DKID -0.626141

DKIE -0.660825

DKIF 0.00261235

DKIG -0.970627

DKIH -0.0212345

DKII -0.220866

DKIK -0.563224

DKIL -0.597659

DKIM 0.255488

DKIN -0.458469

DKIP -0.565836

DKIQ -0.318319

DKIR -0.400984

DKIS -0.586416

DKIT -0.446172

DKIV -0.435118

DKIW 0.444719

DKIY 0.0518153

DKKA -0.900149

DKKC -0.121201

DKKD -0.519974

DKKE -0.520908

DKKF -0.350504

DKKG -1.14029

DKKH -0.347399

DKKI -0.607928

DKKK -0.587544

DKKL -0.847597

DKKM -0.304438

DKKN -0.518979

DKKP -0.788421

DKKQ -0.379484

DKKR -0.602559

DKKS -0.665294

DKKT -0.593811

DKKV -0.734674

DKKW -0.00497198

DKKY -0.217244

DKLA -1.00217

DKLC 0.237721

DKLD -0.90663

DKLE -0.941727

DKLF -0.310947

DKLG -1.25069

DKLH -0.186819

DKLI -0.609323

DKLK -0.837165

DKLL -0.960591

DKLM 0.00678468

DKLN -0.712784

DKLP -0.778824

DKLQ -0.497331

DKLR -0.626904

DKLS -0.869916

DKLT -0.771424

DKLV -0.814979

DKLW 0.28836

DKLY -0.261036

DKMA -0.228462

DKMC 0.557426

DKMD -0.260458

DKME -0.260618

DKMF 0.473287

DKMG -0.746557

DKMH 0.182135

DKMI 0.223385

DKMK -0.206706

DKML 0.0299022

DKMM 0.586156

DKMN -0.186902

DKMP -0.358855

DKMQ -0.0294943

DKMR -0.0387967

DKMS -0.266707

DKMT -0.113845

DKMV 0.0644364

DKMW 0.592182

DKMY 0.438369

DKNA -0.715635

DKNC 0.0173478

DKND -0.404007

DKNE -0.508466

DKNF -0.183939

DKNG -0.879501

DKNH -0.0628376

DKNI -0.461464

DKNK 0.0551629

DKNL -0.71353

DKNM -0.164256

DKNN -0.172027

DKNP -0.531203

DKNQ -0.203052

DKNR -0.279241

DKNS -0.467407

DKNT -0.40852

DKNV -0.554019

DKNW 0.281403

DKNY -0.00549722

DKPA -0.755884

DKPC -0.0291965

DKPD -0.512133

DKPE -0.526615

DKPF -0.151

DKPG -0.898438

DKPH -0.106605

DKPI -0.475393

DKPK -0.505481

DKPL -0.685469

DKPM -0.118132

DKPN -0.415773

DKPP -0.565881

DKPQ -0.235814

DKPR -0.355852

DKPS -0.523957

DKPT -0.452686

DKPV -0.52268

DKPW 0.323319

DKPY -0.0545249

DKQA -0.608944

DKQC 0.177357

DKQD -0.450238

DKQE -0.441813

DKQF -0.0369613

DKQG -0.827394

DKQH -0.0985675

DKQI -0.345664

DKQK -0.246966

DKQL -0.473107

DKQM -0.0653043

DKQN -0.253018

DKQP -0.547812

DKQQ -0.0498352

DKQR -0.257052

DKQS -0.423365

DKQT -0.340868

DKQV -0.458841

DKQW 0.304342

DKQY 0.0737021

DKRA -0.740559

DKRC 0.0646417

DKRD -0.367428

DKRE -0.377443

DKRF -0.117278

DKRG -0.975162

DKRH -0.0575712

DKRI -0.402436

DKRK -0.569316

DKRL -0.657807

DKRM -0.0083859

DKRN -0.391127

DKRP -0.543962

DKRQ -0.23652

DKRR -0.302132

DKRS -0.516536

DKRT -0.47974

DKRV -0.53265

DKRW 0.254908

DKRY -0.014998

DKSA -0.899844

DKSC 0.043216

DKSD -0.586651

DKSE -0.647413

DKSF -0.298884

DKSG -0.946224

DKSH -0.164595

DKSI -0.624419

DKSK -0.54421

DKSL -0.858818

DKSM -0.229878

DKSN -0.411879

DKSP -0.739876

DKSQ -0.365337

DKSR -0.403486

DKSS -0.577578

DKST -0.532883

DKSV -0.73396

DKSW 0.116425

DKSY -0.172788

DKTA -0.829856

DKTC 0.135425

DKTD -0.576009

DKTE -0.593763

DKTF -0.192077

DKTG -0.960791

DKTH -0.107283

DKTI -0.466477

DKTK -0.494464

DKTL -0.762429

DKTM -0.086828

DKTN -0.39529

DKTP -0.574239

DKTQ -0.238901

DKTR -0.432655

DKTS -0.572512

DKTT -0.444978

DKTV -0.607729

DKTW 0.212023

DKTY -0.11832

DKVA -0.843683

DKVC 0.249762

DKVD -0.768279

DKVE -0.809045

DKVF -0.178863

DKVG -1.13086

DKVH -0.13724

DKVI -0.434753

DKVK -0.631291

DKVL -0.775476

DKVM 0.0809884

DKVN -0.595768

DKVP -0.68948

DKVQ -0.448304

DKVR -0.554491

DKVS -0.730435

DKVT -0.557982

DKVV -0.627993

DKVW 0.316208

DKVY -0.140663

DKWA -0.0348775

DKWC 0.877475

DKWD 0.0273979

DKWE -0.00026679

DKWF 0.743266

DKWG -0.28569

DKWH 0.478015

DKWI 0.485253

DKWK 0.0353718

DKWL 0.297211

DKWM 0.676551

DKWN 0.216824

DKWP 0.0677774

DKWQ 0.325911

DKWR 0.306087

DKWS 0.0387838

DKWT 0.123683

DKWV 0.353714

DKWW 1.02464

DKWY 0.834058

DKYA -0.434148

DKYC 0.748726

DKYD -0.194075

DKYE -0.323282

DKYF 0.319035

DKYG -0.651134

DKYH 0.308324

DKYI 0.00403953

DKYK -0.196212

DKYL -0.290039

DKYM 0.416833

DKYN -0.0434334

DKYP -0.15236

DKYQ 0.0567958

DKYR -0.0133553

DKYS -0.229043

DKYT -0.133326

DKYV -0.164692

DKYW 0.79154

DKYY 0.387319

DLAA -1.03795

DLAC -0.120626

DLAD -0.930943

DLAE -0.937809

DLAF -0.413129

DLAG -1.30923

DLAH -0.339887

DLAI -0.655557

DLAK -0.835671

DLAL -0.788669

DLAM -0.171209

DLAN -0.746422

DLAP -1.0135

DLAQ -0.60688

DLAR -0.687037

DLAS -0.88998

DLAT -0.818336

DLAV -0.894065

DLAW 0.0271423

DLAY -0.361108

DLCA -0.06406

DLCC 1.93543

DLCD -0.19259

DLCE -0.253731

DLCF 0.556083

DLCG -0.321405

DLCH 0.504523

DLCI 0.276554

DLCK -0.0536847

DLCL 0.172328

DLCM 0.498999

DLCN 0.0286736

DLCP -0.0849202

DLCQ 0.206732

DLCR 0.158823

DLCS 0.0108197

DLCT 0.00553203

DLCV 0.231251

DLCW 0.887249

DLCY 0.595736

DLDA -0.70844

DLDC -0.167681

DLDD -0.646263

DLDE -0.613554

DLDF -0.0153742

DLDG -0.964816

DLDH -0.0851805

DLDI -0.255165

DLDK -0.403838

DLDL 4.24131

DLDM -0.113062

DLDN -0.364652

DLDP -0.708849

DLDQ -0.333965

DLDR -0.248539

DLDS -0.407832

DLDT -0.261778

DLDV 0.424321

DLDW 0.123296

DLDY -0.153903

DLEA -0.898645

DLEC -0.240299

DLED -0.775039

DLEE -0.744632

DLEF -0.377677

DLEG -1.2246

DLEH -0.197937

DLEI -0.613247

DLEK -0.519017

DLEL -0.391986

DLEM -0.195359

DLEN -0.549897

DLEP -0.844044

DLEQ -0.415664

DLER -0.369961

DLES -0.75013

DLET -0.690246

DLEV -0.792763

DLEW 0.0872133

DLEY -0.277222

DLFA -0.437067

DLFC 0.573335

DLFD -0.302395

DLFE -0.391329

DLFF 0.30928

DLFG -0.704523

DLFH 0.309961

DLFI 0.00916791

DLFK -0.313375

DLFL -0.26057

DLFM 0.490585

DLFN -0.17337

DLFP -0.268178

DLFQ -0.0465815

DLFR -0.132547

DLFS -0.279457

DLFT -0.202804

DLFV -0.173346

DLFW 0.743443

DLFY 0.315732

DLGA -1.32282

DLGC -0.427849

DLGD -0.972173

DLGE -1.10485

DLGF -0.632497

DLGG -2.03817

DLGH -0.637355

DLGI -0.941211

DLGK -0.948267

DLGL -0.953569

DLGM -0.579845

DLGN -0.82692

DLGP -1.14116

DLGQ -0.787667

DLGR -0.780583

DLGS -0.971628

DLGT -0.872499

DLGV -1.02839

DLGW -0.31008

DLGY -0.529737

DLHA -0.404738

DLHC 0.436919

DLHD -0.0608518

DLHE -0.180459

DLHF 0.313171

DLHG -0.64627

DLHH 0.458296

DLHI -0.0387163

DLHK -0.313242

DLHL -0.0886829

DLHM 0.300897

DLHN -0.0778804

DLHP -0.25028

DLHQ -0.0711555

DLHR -0.0261376

DLHS -0.162148

DLHT -0.0920475

DLHV -0.147278

DLHW 0.679458

DLHY 0.376184

DLIA -0.677122

DLIC 0.354156

DLID -0.614303

DLIE -0.649995

DLIF 0.014396

DLIG -0.979553

DLIH -0.00258017

DLII -0.201396

DLIK -0.574759

DLIL -0.580609

DLIM 0.283308

DLIN -0.43604

DLIP -0.549576

DLIQ -0.297749

DLIR -0.368662

DLIS -0.571296

DLIT -0.433123

DLIV -0.409075

DLIW 0.464102

DLIY 0.0736873

DLKA -0.859187

DLKC -0.11532

DLKD -0.517795

DLKE -0.525352

DLKF -0.326582

DLKG -1.12632

DLKH -0.328372

DLKI -0.546191

DLKK -0.631013

DLKL -0.817892

DLKM -0.294524

DLKN -0.489857

DLKP -0.774971

DLKQ -0.385288

DLKR -0.59441

DLKS -0.696847

DLKT -0.578915

DLKV -0.720421

DLKW 0.00545835

DLKY -0.180451

DLLA -0.98596

DLLC 0.253978

DLLD -0.888404

DLLE -0.904422

DLLF -0.292589

DLLG -1.21049

DLLH -0.163928

DLLI -0.607136

DLLK -0.834786

DLLL -0.911123

DLLM 0.0192444

DLLN -0.706065

DLLP -0.748303

DLLQ -0.476796

DLLR -0.607532

DLLS -0.811561

DLLT -0.73785

DLLV -0.749002

DLLW 0.306918

DLLY -0.239133

DLMA -0.211074

DLMC 0.570956

DLMD -0.241375

DLME -0.244596

DLMF 0.490111

DLMG -0.729127

DLMH 0.199864

DLMI 0.250904

DLMK -0.210606

DLML 0.0195062

DLMM 0.593023

DLMN -0.174137

DLMP -0.342283

DLMQ -0.0173616

DLMR -0.0193961

DLMS -0.253822

DLMT -0.0997331

DLMV 0.0997834

DLMW 0.602842

DLMY 0.456959

DLNA -0.744608

DLNC 0.0380797

DLND -0.411963

DLNE -0.539678

DLNF -0.163985

DLNG -0.854319

DLNH -0.0655828

DLNI -0.429976

DLNK -0.437808

DLNL -0.392023

DLNM -0.153134

DLNN -0.190855

DLNP -0.51935

DLNQ -0.21518

DLNR -0.321313

DLNS -0.442341

DLNT -0.429865

DLNV -0.584776

DLNW 0.284109

DLNY -0.0252016

DLPA -0.745574

DLPC -0.0147412

DLPD -0.512278

DLPE -0.506896

DLPF -0.12905

DLPG -0.883526

DLPH -0.0981324

DLPI -0.445944

DLPK -0.554966

DLPL -0.621428

DLPM -0.110078

DLPN -0.391271

DLPP -0.562795

DLPQ -0.282159

DLPR -0.338583

DLPS -0.506524

DLPT -0.444141

DLPV -0.505201

DLPW 0.347917

DLPY -0.0416217

DLQA -0.596274

DLQC 0.191494

DLQD -0.439778

DLQE -0.423311

DLQF -0.0219142

DLQG -0.807094

DLQH -0.0791392

DLQI -0.378251

DLQK -0.362001

DLQL -0.437474

DLQM -0.0519733

DLQN -0.235968

DLQP -0.527876

DLQQ -0.0401239

DLQR -0.186261

DLQS -0.390988

DLQT -0.316051

DLQV -0.343544

DLQW 0.331672

DLQY 0.0932319

DLRA -0.734354

DLRC 0.0784771

DLRD -0.34426

DLRE -0.366482

DLRF -0.101036

DLRG -0.95704

DLRH -0.0440543

DLRI -0.366819

DLRK -0.592602

DLRL -0.563595

DLRM 0.0135224

DLRN -0.391679

DLRP -0.514498

DLRQ -0.206501

DLRR -0.295382

DLRS -0.486704

DLRT -0.466299

DLRV -0.541028

DLRW 0.267609

DLRY -0.00050545

DLSA -0.883152

DLSC 0.0586393

DLSD -0.608263

DLSE -0.661922

DLSF -0.261053

DLSG -0.964361

DLSH -0.143669

DLSI -0.564709

DLSK -0.632214

DLSL -0.695429

DLSM -0.211536

DLSN -0.440108

DLSP -0.730494

DLSQ -0.307886

DLSR -0.461949

DLSS -0.553359

DLST -0.512547

DLSV -0.772755

DLSW 0.123265

DLSY -0.179333

DLTA -0.84265

DLTC 0.151256

DLTD -0.551586

DLTE -0.600665

DLTF -0.182312

DLTG -0.928204

DLTH -0.0828402

DLTI -0.399822

DLTK -0.577657

DLTL -0.68825

DLTM -0.0706036

DLTN -0.372487

DLTP -0.587932

DLTQ -0.267437

DLTR -0.406025

DLTS -0.544147

DLTT -0.395505

DLTV -0.588004

DLTW 0.224941

DLTY -0.0984468

DLVA -0.811581

DLVC 0.270456

DLVD -0.747947

DLVE -0.79001

DLVF -0.150866

DLVG -1.1056

DLVH -0.129735

DLVI -0.412584

DLVK -0.709313

DLVL -0.798337

DLVM 0.108078

DLVN -0.59226

DLVP -0.663534

DLVQ -0.428434

DLVR -0.52192

DLVS -0.71611

DLVT -0.58894

DLVV -0.62778

DLVW 0.337674

DLVY -0.132113

DLWA -0.0220082

DLWC 0.891164

DLWD 0.0429862

DLWE 0.0147984

DLWF 0.759675

DLWG -0.250746

DLWH 0.49525

DLWI 0.49671

DLWK 0.0508428

DLWL 0.315092

DLWM 0.693132

DLWN 0.230994

DLWP 0.0828159

DLWQ 0.339273

DLWR 0.329836

DLWS 0.0541828

DLWT 0.147063

DLWV 0.36796

DLWW 1.03895

DLWY 0.851332

DLYA -0.401454

DLYC 0.764451

DLYD -0.179419

DLYE -0.292751

DLYF 0.349002

DLYG -0.549905

DLYH 0.325417

DLYI 0.0259922

DLYK -0.200082

DLYL -0.219054

DLYM 0.442416

DLYN -0.0234268

DLYP -0.129328

DLYQ 0.0749409

DLYR 0.0031743

DLYS -0.23232

DLYT -0.127192

DLYV -0.160202

DLYW 0.810099

DLYY 0.405168

DMAA -0.509168

DMAC 0.231448

DMAD -0.464417

DMAE -0.453402

DMAF 0.0790648

DMAG -0.901864

DMAH 0.0518281

DMAI -0.171807

DMAK -0.375914

DMAL -0.44973

DMAM 0.255194

DMAN -0.315517

DMAP -0.60401

DMAQ -0.167561

DMAR -0.223863

DMAS -0.436863

DMAT -0.339313

DMAV -0.324716

DMAW 0.394138

DMAY 0.120901

DMCA 0.295796

DMCC 2.35669

DMCD 0.089144

DMCE 0.033076

DMCF 0.89806

DMCG -0.0245998

DMCH 0.755898

DMCI 0.631477

DMCK 0.251915

DMCL 0.586583

DMCM 0.745449

DMCN 0.303086

DMCP 0.178709

DMCQ 0.482022

DMCR 0.466022

DMCS 0.326903

DMCT 0.310802

DMCV 0.622972

DMCW 1.13144

DMCY 0.924817

DMDA -0.354955

DMDC 0.11745

DMDD -0.196213

DMDE -0.214593

DMDF 0.186781

DMDG -0.552917

DMDH 0.28881

DMDI -0.010071

DMDK 0.0764043

DMDL -0.194032

DMDM 2.99803

DMDN 0.117369

DMDP -0.324537

DMDQ 0.0662944

DMDR 0.168214

DMDS -0.0588965

DMDT 0.0596633

DMDV -0.233839

DMDW 0.414427

DMDY 0.301781

DMEA -0.453292

DMEC 0.046222

DMED -0.330206

DMEE -0.257835

DMEF 0.0398698

DMEG -0.824613

DMEH 0.182153

DMEI -0.193526

DMEK 0.0103867

DMEL -0.349644

DMEM 0.146066

DMEN -0.117853

DMEP -0.47103

DMEQ 0.00291181

DMER 0.112781

DMES -0.323613

DMET -0.220265

DMEV -0.328817

DMEW 0.429779

DMEY 0.165958

DMFA 0.0461087

DMFC 0.917185

DMFD 0.140825

DMFE 0.0487266

DMFF 0.806354

DMFG -0.295697

DMFH 0.680507

DMFI 0.506158

DMFK 0.12832

DMFL 0.220564

DMFM 0.894981

DMFN 0.231581

DMFP 0.1309

DMFQ 0.359947

DMFR 0.306361

DMFS 0.173477

DMFT 0.261327

DMFV 0.341091

DMFW 1.10212

DMFY 0.787046

DMGA -0.892986

DMGC -0.149175

DMGD -0.538071

DMGE -0.675251

DMGF -0.207062

DMGG -1.7574

DMGH -0.318338

DMGI -0.495691

DMGK -0.515418

DMGL -0.677521

DMGM -0.259861

DMGN -0.417341

DMGP -0.79332

DMGQ -0.404758

DMGR -0.368011

DMGS -0.546465

DMGT -0.419545

DMGV -0.572637

DMGW -0.0136154

DMGY -0.0968089

DMHA -0.0302103

DMHC 0.682208

DMHD 0.319885

DMHE 0.189775

DMHF 0.676653

DMHG -0.331813

DMHH 0.761267

DMHI 0.334282

DMHK 0.024478

DMHL 0.274553

DMHM 0.571453

DMHN 0.243912

DMHP 0.0464032

DMHQ 0.228725

DMHR 0.32585

DMHS 0.208525

DMHT 0.279027

DMHV 0.270533

DMHW 0.94567

DMHY 0.737553

DMIA -0.159256

DMIC 0.722772

DMID -0.160527

DMIE -0.180762

DMIF 0.521128

DMIG -0.554326

DMIH 0.378098

DMII 0.30701

DMIK -0.111866

DMIL -0.0335858

DMIM 0.701542

DMIN -0.0102186

DMIP -0.13325

DMIQ 0.121429

DMIR 0.0769513

DMIS -0.102648

DMIT 0.0490119

DMIV 0.119248

DMIW 0.845629

DMIY 0.558004

DMKA -0.425082

DMKC 0.186203

DMKD -0.0296481

DMKE -0.0268958

DMKF 0.109168

DMKG -0.723873

DMKH 0.00484705

DMKI -0.101615

DMKK -0.16603

DMKL -0.348883

DMKM 0.0519485

DMKN -0.0764666

DMKP -0.390795

DMKQ 0.028625

DMKR -0.16401

DMKS -0.23811

DMKT -0.140879

DMKV -0.240305

DMKW 0.31858

DMKY 0.25017

DMLA -0.453268

DMLC 0.692599

DMLD -0.401388

DMLE -0.398325

DMLF 0.23774

DMLG -0.748028

DMLH 0.285817

DMLI -0.054116

DMLK -0.324748

DMLL -0.390292

DMLM 0.496179

DMLN -0.237496

DMLP -0.304301

DMLQ -0.00345016

DMLR -0.119709

DMLS -0.356681

DMLT -0.231056

DMLV -0.242831

DMLW 0.749881

DMLY 0.27527

DMMA 0.200289

DMMC 0.824357

DMMD 0.0914085

DMME 0.108929

DMMF 0.891963

DMMG -0.436704

DMMH 0.455283

DMMI 0.67249

DMMK 0.132966

DMML 0.489946

DMMM 0.902691

DMMN 0.126519

DMMP -0.0632961

DMMQ 0.281126

DMMR 0.323655

DMMS 0.0763085

DMMT 0.255565

DMMV 0.519812

DMMW 0.857655

DMMY 0.828007

DMNA -0.310966

DMNC 0.307775

DMND 0.0147774

DMNE -0.0951068

DMNF 0.239509

DMNG -0.456324

DMNH 0.270204

DMNI -0.041739

DMNK 8.154e-05

DMNL -0.201722

DMNM 0.185679

DMNN 0.22386

DMNP -0.156677

DMNQ 0.179624

DMNR 0.102248

DMNS -0.0145347

DMNT 0.0393202

DMNV -0.124842

DMNW 0.601294

DMNY 0.424127

DMPA -0.294387

DMPC 0.257661

DMPD -0.0819287

DMPE -0.0524807

DMPF 0.292316

DMPG -0.482687

DMPH 0.227326

DMPI -0.0267637

DMPK -0.13921

DMPL -0.179765

DMPM 0.213909

DMPN -0.00277734

DMPP -0.177571

DMPQ 0.10729

DMPR 0.0766001

DMPS -0.0664544

DMPT -0.0120082

DMPV -0.0407569

DMPW 0.657888

DMPY 0.384893

DMQA -0.141956

DMQC 0.46497

DMQD -0.032634

DMQE -0.00172353

DMQF 0.397588

DMQG -0.430107

DMQH 0.216109

DMQI 0.0830159

DMQK 0.0615134

DMQL -0.0294421

DMQM 0.234176

DMQN 0.145121

DMQP -0.186874

DMQQ 0.367085

DMQR 0.200334

DMQS 0.0242846

DMQT 0.107977

DMQV 0.00538492

DMQW 0.615833

DMQY 0.503197

DMRA -0.249539

DMRC 0.37486

DMRD 0.133197

DMRE 0.119377

DMRF 0.354658

DMRG -0.557607

DMRH 0.304261

DMRI 0.073725

DMRK -0.164747

DMRL -0.133989

DMRM 0.353179

DMRN 0.0355661

DMRP -0.136189

DMRQ 0.206961

DMRR 0.151656

DMRS -0.0508237

DMRT -0.0174346

DMRV -0.0452368

DMRW 0.591302

DMRY 0.452237

DMSA -0.441296

DMSC 0.381509

DMSD -0.151392

DMSE -0.194266

DMSF 0.184802

DMSG -0.499712

DMSH 0.220142

DMSI -0.135037

DMSK -0.165725

DMSL -0.33351

DMSM 0.154152

DMSN 0.00094676

DMSP -0.329153

DMSQ 0.1058

DMSR 0.00178671

DMSS -0.0585256

DMST -0.0229912

DMSV -0.250946

DMSW 0.44802

DMSY 0.277987

DMTA -0.337287

DMTC 0.480825

DMTD -0.101932

DMTE -0.120513

DMTF 0.285124

DMTG -0.489335

DMTH 0.280415

DMTI 0.028239

DMTK -0.119333

DMTL -0.233882

DMTM 0.297217

DMTN 0.0551331

DMTP -0.194821

DMTQ 0.165143

DMTR 0.0591869

DMTS -0.0592484

DMTT 0.0590739

DMTV -0.0766194

DMTW 0.567764

DMTY 0.376292

DMVA -0.314922

DMVC 0.662909

DMVD -0.285189

DMVE -0.305272

DMVF 0.351986

DMVG -0.663214

DMVH 0.279882

DMVI 0.100254

DMVK -0.226479

DMVL -0.232583

DMVM 0.543674

DMVN -0.127882

DMVP -0.236467

DMVQ 0.0109711

DMVR -0.0673244

DMVS -0.238816

DMVT -0.0999448

DMVV -0.0637231

DMVW 0.741048

DMVY 0.365633

DMWA 0.335087

DMWC 1.13563

DMWD 0.358487

DMWE 0.341352

DMWF 1.13448

DMWG 0.0532119

DMWH 0.739437

DMWI 0.891426

DMWK 0.370286

DMWL 0.757665

DMWM 0.956316

DMWN 0.531562

DMWP 0.366372

DMWQ 0.632847

DMWR 0.651168

DMWS 0.378864

DMWT 0.462199

DMWV 0.776392

DMWW 1.29703

DMWY 1.22303

DMYA 0.0568023

DMYC 1.12534

DMYD 0.263622

DMYE 0.148774

DMYF 0.810917

DMYG -0.130566

DMYH 0.685027

DMYI 0.505169

DMYK 0.244162

DMYL 0.24837

DMYM 0.817413

DMYN 0.396759

DMYP 0.278734

DMYQ 0.486497

DMYR 0.447986

DMYS 0.2158

DMYT 0.329913

DMYV 0.338717

DMYW 1.17406

DMYY 0.881606

DNAA -0.811541

DNAC 0.0623872

DNAD -0.717419

DNAE -0.715269

DNAF -0.197306

DNAG -1.12928

DNAH -0.155041

DNAI -0.46058

DNAK -0.651633

DNAL -0.765083

DNAM 0.0252512

DNAN -0.487847

DNAP -0.797157

DNAQ -0.390224

DNAR -0.477262

DNAS -0.685599

DNAT -0.597379

DNAV -0.629719

DNAW 0.208566

DNAY -0.151145

DNCA 0.121375

DNCC 2.1352

DNCD -0.0220187

DNCE -0.0868151

DNCF 0.738709

DNCG -0.150646

DNCH 0.663301

DNCI 0.460681

DNCK 0.119162

DNCL 0.367774

DNCM 0.656077

DNCN 0.193167

DNCP 0.077265

DNCQ 0.372134

DNCR 0.331953

DNCS 0.188061

DNCT 0.177847

DNCV 0.426741

DNCW 1.0443

DNCY 0.773615

DNDA -0.568127

DNDC -0.00079417

DNDD 0.0152035

DNDE -0.411079

DNDF -0.112526

DNDG -0.677396

DNDH 0.175275

DNDI -0.370383

DNDK 0.118293

DNDL -0.637853

DNDM -0.0748613

DNDN 4.43214

DNDP -0.491642

DNDQ -0.0988152

DNDR -0.00178862

DNDS -0.188212

DNDT -0.0319643

DNDV -0.503486

DNDW 0.280457

DNDY 0.117005

DNEA -0.704705

DNEC -0.0740101

DNED -0.590852

DNEE -0.524016

DNEF -0.193562

DNEG -1.00911

DNEH -0.00490355

DNEI -0.449898

DNEK -0.281656

DNEL -0.687108

DNEM -0.104555

DNEN -0.0852396

DNEP -0.644999

DNEQ -0.208925

DNER -0.125392

DNES -0.557241

DNET -0.3921

DNEV -0.574886

DNEW 0.270436

DNEY -0.0757008

DNFA -0.223911

DNFC 0.755325

DNFD -0.0968981

DNFE -0.188726

DNFF 0.532857

DNFG -0.517652

DNFH 0.49411

DNFI 0.226232

DNFK -0.112591

DNFL -0.0809588

DNFM 0.685361

DNFN 0.0229623

DNFP -0.077364

DNFQ 0.158345

DNFR 0.0819039

DNFS -0.0798821

DNFT 0.0119734

DNFV 0.0457098

DNFW 0.922707

DNFY 0.527145

DNGA -1.11548

DNGC -0.263629

DNGD -0.748943

DNGE -0.903944

DNGF -0.433944

DNGG -1.87097

DNGH -0.458292

DNGI -0.725107

DNGK -0.766641

DNGL -0.944051

DNGM -0.408252

DNGN -0.574228

DNGP -0.959761

DNGQ -0.586088

DNGR -0.595359

DNGS -0.773202

DNGT -0.656498

DNGV -0.826731

DNGW -0.13686

DNGY -0.309919

DNHA -0.211333

DNHC 0.599471

DNHD 0.126537

DNHE 0.00591779

DNHF 0.503515

DNHG -0.470285

DNHH 0.633857

DNHI 0.141552

DNHK -0.116523

DNHL 0.0390313

DNHM 0.458784

DNHN 0.124268

DNHP -0.0809419

DNHQ 0.0999846

DNHR 0.157408

DNHS 0.032325

DNHT 0.102298

DNHV 0.0699692

DNHW 0.839928

DNHY 0.554254

DNIA -0.450101

DNIC 0.542152

DNID -0.405952

DNIE -0.437925

DNIF 0.240272

DNIG -0.774011

DNIH 0.185947

DNII 0.00555086

DNIK -0.366591

DNIL -0.353397

DNIM 0.480165

DNIN -0.228571

DNIP -0.348634

DNIQ -0.10567

DNIR -0.174563

DNIS -0.360883

DNIT -0.218237

DNIV -0.200123

DNIW 0.652414

DNIY 0.27995

DNKA -0.687591

DNKC 0.0601029

DNKD -0.29467

DNKE -0.277907

DNKF -0.115486

DNKG -0.929882

DNKH -0.144187

DNKI -0.37837

DNKK -0.408713

DNKL -0.6352

DNKM -0.108256

DNKN -0.251047

DNKP -0.577958

DNKQ -0.190102

DNKR -0.395215

DNKS -0.492415

DNKT -0.386175

DNKV -0.50933

DNKW 0.181447

DNKY 0.0150306

DNLA -0.768194

DNLC 0.458003

DNLD -0.673776

DNLE -0.683108

DNLF -0.0695024

DNLG -0.996855

DNLH 0.0452397

DNLI -0.362942

DNLK -0.612853

DNLL -0.717771

DNLM 0.256884

DNLN -0.466085

DNLP -0.556379

DNLQ -0.264306

DNLR -0.405627

DNLS -0.621379

DNLT -0.526163

DNLV -0.553873

DNLW 0.510689

DNLY -0.0159521

DNMA -0.00971437

DNMC 0.730543

DNMD -0.0641215

DNME -0.0590394

DNMF 0.686346

DNMG -0.560932

DNMH 0.360689

DNMI 0.446913

DNMK -0.0286655

DNML 0.232105

DNMM 0.769011

DNMN 0.00818491

DNMP -0.175849

DNMQ 0.153966

DNMR 0.163071

DNMS -0.0809691

DNMT 0.0845568

DNMV 0.286258

DNMW 0.763352

DNMY 0.642401

DNNA -0.549762

DNNC 0.201427

DNND -0.140837

DNNE -0.329625

DNNF 0.021467

DNNG -0.642665

DNNH 0.128414

DNNI -0.26333

DNNK -0.154991

DNNL -0.447407

DNNM 0.00886774

DNNN 0.314347

DNNP -0.343395

DNNQ -0.00459814

DNNR -0.117626

DNNS -0.241278

DNNT -0.145189

DNNV -0.385287

DNNW 0.465803

DNNY 0.200953

DNPA -0.533882

DNPC 0.149382

DNPD -0.305509

DNPE -0.283016

DNPF 0.0765362

DNPG -0.688727

DNPH 0.0800941

DNPI -0.25515

DNPK -0.364937

DNPL -0.447264

DNPM 0.0659854

DNPN -0.191962

DNPP -0.360885

DNPQ -0.0792267

DNPR -0.150384

DNPS -0.306266

DNPT -0.246735

DNPV -0.30507

DNPW 0.512891

DNPY 0.159778

DNQA -0.384691

DNQC 0.357005

DNQD -0.237751

DNQE -0.214032

DNQF 0.177921

DNQG -0.618032

DNQH 0.0868335

DNQI -0.123298

DNQK -0.164682

DNQL -0.291092

DNQM 0.109504

DNQN -0.00912428

DNQP -0.346094

DNQQ 0.161974

DNQR -0.0071857

DNQS -0.187003

DNQT -0.127601

DNQV -0.241185

DNQW 0.495253

DNQY 0.290485

DNRA -0.489478

DNRC 0.250999

DNRD -0.121065

DNRE -0.163928

DNRF 0.103776

DNRG -0.763717

DNRH 0.13795

DNRI -0.174683

DNRK -0.391794

DNRL -0.41823

DNRM 0.191101

DNRN -0.116657

DNRP -0.334871

DNRQ -0.00683427

DNRR -0.0903616

DNRS -0.282352

DNRT -0.253881

DNRV -0.317668

DNRW 0.446084

DNRY 0.211001

DNSA -0.712546

DNSC 0.237362

DNSD -0.368037

DNSE -0.455596

DNSF -0.0726242

DNSG -0.733682

DNSH 0.0491855

DNSI -0.379064

DNSK -0.416131

DNSL -0.627771

DNSM -0.0298946

DNSN -0.108522

DNSP -0.515095

DNSQ -0.137513

DNSR -0.2528

DNSS -0.319324

DNST -0.295877

DNSV -0.40401

DNSW 0.317104

DNSY 0.0809231

DNTA -0.597054

DNTC 0.328114

DNTD -0.328084

DNTE -0.396234

DNTF 0.0333719

DNTG -0.73468

DNTH 0.100629

DNTI -0.243654

DNTK -0.372685

DNTL -0.508888

DNTM 0.11468

DNTN -0.163794

DNTP -0.373045

DNTQ -0.0605814

DNTR -0.192737

DNTS -0.302496

DNTT -0.216346

DNTV -0.371632

DNTW 0.40988

DNTY 0.117048

DNVA -0.633796

DNVC 0.4623

DNVD -0.538461

DNVE -0.567128

DNVF 0.0620983

DNVG -0.898645

DNVH 0.0690184

DNVI -0.19616

DNVK -0.493861

DNVL -0.549298

DNVM 0.302782

DNVN -0.353501

DNVP -0.469071

DNVQ -0.230237

DNVR -0.331283

DNVS -0.498839

DNVT -0.356381

DNVV -0.375031

DNVW 0.534215

DNVY 0.0899508

DNWA 0.158244

DNWC 1.04826

DNWD 0.23471

DNWE 0.191719

DNWF 0.949315

DNWG -0.0759089

DNWH 0.650425

DNWI 0.691439

DNWK 0.227663

DNWL 0.520871

DNWM 0.852641

DNWN 0.404858

DNWP 0.249358

DNWQ 0.513407

DNWR 0.501633

DNWS 0.234881

DNWT 0.319019

DNWV 0.562578

DNWW 1.19955

DNWY 1.0393

DNYA -0.207905

DNYC 0.95042

DNYD 0.0446358

DNYE -0.0954471

DNYF 0.550767

DNYG -0.346888

DNYH 0.514796

DNYI 0.232346

DNYK -0.00115085

DNYL -0.0518479

DNYM 0.619458

DNYN 0.20254

DNYP 0.0641828

DNYQ 0.27506

DNYR 0.250144

DNYS 0.0112472

DNYT 0.0870051

DNYV 0.0642998

DNYW 0.993588

DNYY 0.629642

DPAA -0.610984

DPAC 0.197218

DPAD -0.516398

DPAE -0.530532

DPAF -0.00849581

DPAG -0.961326

DPAH -0.00114942

DPAI -0.25581

DPAK -0.465988

DPAL -0.548416

DPAM 0.173851

DPAN -0.379298

DPAP -0.639335

DPAQ -0.231965

DPAR -0.307219

DPAS -0.48897

DPAT -0.417685

DPAV -0.420471

DPAW 0.353327

DPAY 0.0394955

DPCA 0.262126

DPCC 2.29817

DPCD 0.0812294

DPCE 0.0242307

DPCF 0.868839

DPCG -0.0375645

DPCH 0.758072

DPCI 0.596007

DPCK 0.235773

DPCL 0.529124

DPCM 0.748213

DPCN 0.297814

DPCP 0.178265

DPCQ 0.476085

DPCR 0.449305

DPCS 0.305944

DPCT 0.297467

DPCV 0.576377

DPCW 1.13581

DPCY 0.900934

DPDA -0.350002

DPDC 0.100772

DPDD -0.250431

DPDE -0.28023

DPDF 0.0439644

DPDG -0.608983

DPDH 0.227561

DPDI -0.219475

DPDK -0.0447376

DPDL -0.454129

DPDM 0.0111525

DPDN 0.0362785

DPDP 3.48013

DPDQ -0.0192683

DPDR 0.116971

DPDS -0.0445995

DPDT -0.129118

DPDV -0.326919

DPDW 0.387633

DPDY 0.233607

DPEA -0.545542

DPEC 0.0366046

DPED -0.401056

DPEE -0.338515

DPEF -0.0077343

DPEG -0.87636

DPEH 0.133602

DPEI -0.270742

DPEK -0.099829

DPEL -0.476298

DPEM 0.0398896

DPEN -0.187282

DPEP -0.283804

DPEQ -0.0495198

DPER 0.0510411

DPES -0.381042

DPET -0.291485

DPEV -0.395652

DPEW 0.404081

DPEY 0.0966311

DPFA -0.0400119

DPFC 0.887345

DPFD 0.0726418

DPFE -0.0196195

DPFF 0.720535

DPFG -0.349772

DPFH 0.639076

DPFI 0.424827

DPFK 0.0607364

DPFL 0.132056

DPFM 0.839391

DPFN 0.175528

DPFP 0.0889912

DPFQ 0.305975

DPFR 0.239671

DPFS 0.102872

DPFT 0.183189

DPFV 0.242698

DPFW 1.0638

DPFY 0.712696

DPGA -0.956842

DPGC -0.156583

DPGD -0.603826

DPGE -0.72753

DPGF -0.267503

DPGG -1.76496

DPGH -0.33783

DPGI -0.559732

DPGK -0.592509

DPGL -0.761415

DPGM -0.28769

DPGN -0.473345

DPGP -0.819108

DPGQ -0.444658

DPGR -0.430235

DPGS -0.614689

DPGT -0.487591

DPGV -0.643459

DPGW -0.0266035

DPGY -0.161622

DPHA -0.0737989

DPHC 0.686496

DPHD 0.275647

DPHE 0.153296

DPHF 0.638043

DPHG -0.348176

DPHH 0.746223

DPHI 0.290358

DPHK -0.00354242

DPHL 0.209255

DPHM 0.561783

DPHN 0.220291

DPHP 0.0587351

DPHQ 0.215078

DPHR 0.29801

DPHS 0.172115

DPHT 0.23951

DPHV 0.218852

DPHW 0.943577

DPHY 0.694846

DPIA -0.255032

DPIC 0.683673

DPID -0.235595

DPIE -0.25917

DPIF 0.431014

DPIG -0.613654

DPIH 0.334339

DPII 0.214829

DPIK -0.186161

DPIL -0.141142

DPIM 0.6373

DPIN -0.0731859

DPIP -0.189383

DPIQ 0.058846

DPIR 0.0065763

DPIS -0.179657

DPIT -0.0291374

DPIV 0.00653839

DPIW 0.80089

DPIY 0.474831

DPKA -0.510723

DPKC 0.169775

DPKD -0.112519

DPKE -0.108675

DPKF 0.0424883

DPKG -0.77766

DPKH -0.0184879

DPKI -0.200665

DPKK -0.239549

DPKL -0.443251

DPKM 0.0177441

DPKN -0.140841

DPKP -0.427957

DPKQ -0.0306134

DPKR -0.230319

DPKS -0.312049

DPKT -0.218073

DPKV -0.325116

DPKW 0.300341

DPKY 0.18153

DPLA -0.565018

DPLC 0.626902

DPLD -0.480519

DPLE -0.489686

DPLF 0.131043

DPLG -0.821373

DPLH 0.212486

DPLI -0.15353

DPLK -0.417036

DPLL -0.500492

DPLM 0.417296

DPLN -0.311924

DPLP -0.349627

DPLQ -0.0842435

DPLR -0.211063

DPLS -0.444329

DPLT -0.328879

DPLV -0.34531

DPLW 0.682226

DPLY 0.177924

DPMA 0.146794

DPMC 0.825867

DPMD 0.0640886

DPME 0.0762694

DPMF 0.840748

DPMG -0.448272

DPMH 0.454882

DPMI 0.604374

DPMK 0.10289

DPML 0.419167

DPMM 0.886944

DPMN 0.112104

DPMP -0.0721362

DPMQ 0.268001

DPMR 0.296879

DPMS 0.0523517

DPMT 0.221941

DPMV 0.453746

DPMW 0.859014

DPMY 0.786578

DPNA -0.36693

DPNC 0.302576

DPND -0.0535533

DPNE -0.121495

DPNF 0.189689

DPNG -0.508254

DPNH 0.241954

DPNI -0.103765

DPNK -0.0631723

DPNL -0.286166

DPNM 0.125641

DPNN 0.162086

DPNP -0.0268333

DPNQ 0.144647

DPNR 0.0540493

DPNS -0.0630615

DPNT -0.0131795

DPNV -0.208689

DPNW 0.594708

DPNY 0.356702

DPPA -0.370529

DPPC 0.253498

DPPD -0.133902

DPPE -0.12832

DPPF 0.230284

DPPG -0.534465

DPPH 0.20407

DPPI -0.0911605

DPPK -0.197616

DPPL -0.259331

DPPM 0.187297

DPPN -0.0538051

DPPP -0.195651

DPPQ 0.0661404

DPPR 0.017406

DPPS -0.132867

DPPT -0.0744181

DPPV -0.108973

DPPW 0.635641

DPPY 0.32566

DPQA -0.211285

DPQC 0.460109

DPQD -0.085603

DPQE -0.068536

DPQF 0.341493

DPQG -0.473005

DPQH 0.208363

DPQI 0.0255556

DPQK -0.00640392

DPQL -0.116708

DPQM 0.221212

DPQN 0.101317

DPQP -0.198784

DPQQ 0.317221

DPQR 0.14219

DPQS -0.0332081

DPQT 0.049814

DPQV -0.0669985

DPQW 0.605086

DPQY 0.451586

DPRA -0.337567

DPRC 0.363268

DPRD 0.0505228

DPRE 0.0317769

DPRF 0.277654

DPRG -0.603247

DPRH 0.273283

DPRI 0.0038321

DPRK -0.225232

DPRL -0.224452

DPRM 0.325957

DPRN -0.017638

DPRP -0.176867

DPRQ 0.157434

DPRR 0.0888915

DPRS -0.119393

DPRT -0.0886369

DPRV -0.128768

DPRW 0.569207

DPRY 0.380401

DPSA -0.509472

DPSC 0.359093

DPSD -0.218762

DPSE -0.273442

DPSF 0.10808

DPSG -0.568757

DPSH 0.207551

DPSI -0.212965

DPSK -0.239388

DPSL -0.409099

DPSM 0.100962

DPSN -0.0596774

DPSP -0.343157

DPSQ 0.0419433

DPSR -0.0577817

DPSS -0.136897

DPST -0.102658

DPSV -0.319964

DPSW 0.422534

DPSY 0.209535

DPTA -0.412922

DPTC 0.456002

DPTD -0.176529

DPTE -0.20035

DPTF 0.212677

DPTG -0.555923

DPTH 0.245044

DPTI -0.0560424

DPTK -0.183469

DPTL -0.323803

DPTM 0.25406

DPTN -0.0162461

DPTP -0.223571

DPTQ 0.1027

DPTR -0.0129313

DPTS -0.140072

DPTT -0.0347881

DPTV -0.168

DPTW 0.538759

DPTY 0.305242

DPVA -0.411876

DPVC 0.613651

DPVD -0.363782

DPVE -0.388355

DPVF 0.263377

DPVG -0.731287

DPVH 0.226021

DPVI 0.00117493

DPVK -0.30977

DPVL -0.352662

DPVM 0.474982

DPVN -0.20773

DPVP -0.253748

DPVQ -0.0594978

DPVR -0.145931

DPVS -0.319201

DPVT -0.188489

DPVV -0.161318

DPVW 0.685627

DPVY 0.286345

DPWA 0.299163

DPWC 1.13991

DPWD 0.341624

DPWE 0.317767

DPWF 1.09292

DPWG 0.0380194

DPWH 0.742764

DPWI 0.84261

DPWK 0.34984

DPWL 0.691164

DPWM 0.95248

DPWN 0.517517

DPWP 0.359284

DPWQ 0.620707

DPWR 0.627453

DPWS 0.354011

DPWT 0.440993

DPWV 0.72157

DPWW 1.29761

DPWY 1.18315

DPYA -0.0121403

DPYC 1.08905

DPYD 0.199405

DPYE 0.0740542

DPYF 0.732215

DPYG -0.189344

DPYH 0.649206

DPYI 0.414733

DPYK 0.175405

DPYL 0.145142

DPYM 0.762386

DPYN 0.335503

DPYP 0.261756

DPYQ 0.432781

DPYR 0.378041

DPYS 0.146624

DPYT 0.258117

DPYV 0.25374

DPYW 1.13445

DPYY 0.801788

DQAA -0.648162

DQAC 0.157569

DQAD -0.593938

DQAE -0.589996

DQAF -0.0691509

DQAG -1.00646

DQAH -0.0471056

DQAI -0.320986

DQAK -0.523028

DQAL -0.62835

DQAM 0.132636

DQAN -0.430776

DQAP -0.690599

DQAQ -0.253447

DQAR -0.382839

DQAS -0.523082

DQAT -0.478521

DQAV -0.489179

DQAW 0.311174

DQAY -0.0247183

DQCA 0.219273

DQCC 2.24719

DQCD 0.0525453

DQCE -0.00767255

DQCF 0.831739

DQCG -0.0683434

DQCH 0.736561

DQCI 0.556522

DQCK 0.203807

DQCL 0.479371

DQCM 0.725464

DQCN 0.270367

DQCP 0.151394

DQCQ 0.449757

DQCR 0.416656

DQCS 0.273377

DQCT 0.262618

DQCV 0.530844

DQCW 1.1133

DQCY 0.864255

DQDA -0.411525

DQDC 0.0758436

DQDD -0.227548

DQDE -0.0241179

DQDF -0.008389

DQDG -0.668758

DQDH 0.271675

DQDI -0.260978

DQDK 0.163279

DQDL -0.462435

DQDM 0.0187943

DQDN 0.0459149

DQDP -0.402458

DQDQ 4.05457

DQDR 0.317214

DQDS -0.188101

DQDT 0.0382075

DQDV -0.289564

DQDW 0.352018

DQDY 0.173932

DQEA -0.566509

DQEC 0.00905442

DQED -0.453542

DQEE -0.355863

DQEF -0.0736527

DQEG -0.904889

DQEH 0.106897

DQEI -0.322469

DQEK -0.0432088

DQEL -0.544977

DQEM -0.00181031

DQEN -0.203943

DQEP -0.557629

DQEQ 0.161706

DQER 0.0231032

DQES -0.471845

DQET -0.272454

DQEV -0.424327

DQEW 0.366569

DQEY 0.054976

DQFA -0.0853214

DQFC 0.849018

DQFD 0.027739

DQFE -0.0746355

DQFF 0.659054

DQFG -0.405732

DQFH 0.595049

DQFI 0.35789

DQFK 0.0135214

DQFL 0.0562768

DQFM 0.792292

DQFN 0.127931

DQFP 0.0366969

DQFQ 0.258146

DQFR 0.183659

DQFS 0.0492001

DQFT 0.133494

DQFV 0.178455

DQFW 1.01925

DQFY 0.65157

DQGA -1.0089

DQGC -0.18541

DQGD -0.652203

DQGE -0.788988

DQGF -0.319312

DQGG -1.79283

DQGH -0.376585

DQGI -0.612512

DQGK -0.657521

DQGL -0.822782

DQGM -0.321156

DQGN -0.526091

DQGP -0.853453

DQGQ -0.46771

DQGR -0.487095

DQGS -0.598289

DQGT -0.53501

DQGV -0.704635

DQGW -0.0562947

DQGY -0.214735

DQHA -0.110645

DQHC 0.664173

DQHD 0.233518

DQHE 0.116594

DQHF 0.592685

DQHG -0.385012

DQHH 0.712501

DQHI 0.241605

DQHK -0.035686

DQHL 0.162786

DQHM 0.534155

DQHN 0.186343

DQHP 0.00085521

DQHQ 0.212

DQHR 0.260295

DQHS 0.13179

DQHT 0.205266

DQHV 0.174089

DQHW 0.915938

DQHY 0.660737

DQIA -0.304476

DQIC 0.642564

DQID -0.287571

DQIE -0.315084

DQIF 0.367649

DQIG -0.665507

DQIH 0.291763

DQII 0.144609

DQIK -0.202106

DQIL -0.214301

DQIM 0.587229

DQIN -0.12519

DQIP -0.238919

DQIQ 0.0158386

DQIR -0.0565107

DQIS -0.237057

DQIT -0.0940773

DQIV -0.0628333

DQIW 0.759807

DQIY 0.409089

DQKA -0.566352

DQKC 0.138448

DQKD -0.155183

DQKE -0.157917

DQKF -0.0075624

DQKG -0.828166

DQKH -0.0607958

DQKI -0.251117

DQKK -0.302269

DQKL -0.501389

DQKM -0.0197287

DQKN -0.189017

DQKP -0.477262

DQKQ -0.0692382

DQKR -0.267574

DQKS -0.357574

DQKT -0.272661

DQKV -0.376894

DQKW 0.267824

DQKY 0.143007

DQLA -0.635376

DQLC 0.574027

DQLD -0.528992

DQLE -0.5415

DQLF 0.0647707

DQLG -0.882099

DQLH 0.159684

DQLI -0.216688

DQLK -0.488366

DQLL -0.56881

DQLM 0.360819

DQLN -0.375489

DQLP -0.432606

DQLQ -0.143545

DQLR -0.275085

DQLS -0.496553

DQLT -0.395218

DQLV -0.418095

DQLW 0.628005

DQLY 0.115379

DQMA 0.0993459

DQMC 0.801959

DQMD 0.0258229

DQME 0.0363858

DQMF 0.793997

DQMG -0.47801

DQMH 0.429864

DQMI 0.554437

DQMK 0.0634069

DQML 0.355783

DQMM 0.854368

DQMN 0.0825329

DQMP -0.0981648

DQMQ 0.236255

DQMR 0.260339

DQMS 0.0106947

DQMT 0.181545

DQMV 0.401023

DQMW 0.834873

DQMY 0.743744

DQNA -0.402243

DQNC 0.275903

DQND -0.116189

DQNE -0.177695

DQNF 0.135448

DQNG -0.554724

DQNH 0.216694

DQNI -0.14814

DQNK -0.0363641

DQNL -0.345149

DQNM 0.0902491

DQNN 0.110361

DQNP -0.242645

DQNQ 0.222112

DQNR 0.0181835

DQNS -0.127482

DQNT -0.0466797

DQNV -0.260158

DQNW 0.548668

DQNY 0.313908

DQPA -0.413416

DQPC 0.226942

DQPD -0.196897

DQPE -0.173167

DQPF 0.183829

DQPG -0.582167

DQPH 0.168644

DQPI -0.14139

DQPK -0.250174

DQPL -0.3243

DQPM 0.153455

DQPN -0.0984323

DQPP -0.256166

DQPQ 0.0226471

DQPR -0.0381761

DQPS -0.186378

DQPT -0.132611

DQPV -0.169848

DQPW 0.601806

DQPY 0.273953

DQQA -0.272016

DQQC 0.432505

DQQD -0.112917

DQQE -0.127117

DQQF 0.293802

DQQG -0.52476

DQQH 0.170888

DQQI -0.022203

DQQK -0.0527329

DQQL -0.175762

DQQM 0.190943

DQQN 0.0469956

DQQP -0.248101

DQQQ 0.331632

DQQR 0.129755

DQQS -0.0695212

DQQT 0.0033915

DQQV -0.11474

DQQW 0.575068

DQQY 0.395153

DQRA -0.392983

DQRC 0.33246

DQRD -0.0141587

DQRE -0.022296

DQRF 0.227247

DQRG -0.65315

DQRH 0.232464

DQRI -0.052438

DQRK -0.280007

DQRL -0.277793

DQRM 0.284553

DQRN -0.0720336

DQRP -0.225207

DQRQ 0.108932

DQRR 0.0336075

DQRS -0.167452

DQRT -0.150561

DQRV -0.177645

DQRW 0.534208

DQRY 0.32509

DQSA -0.576037

DQSC 0.325994

DQSD -0.258925

DQSE -0.318585

DQSF 0.0522637

DQSG -0.624022

DQSH 0.138855

DQSI -0.273098

DQSK -0.284167

DQSL -0.448032

DQSM 0.0600269

DQSN -0.116883

DQSP -0.422891

DQSQ 0.0539596

DQSR -0.128732

DQSS -0.176068

DQST -0.155578

DQSV -0.392187

DQSW 0.386954

DQSY 0.1419

DQTA -0.46856

DQTC 0.418812

DQTD -0.230273

DQTE -0.230284

DQTF 0.149736

DQTG -0.611971

DQTH 0.199253

DQTI -0.114911

DQTK -0.256813

DQTL -0.394868

DQTM 0.219959

DQTN -0.076021

DQTP -0.294844

DQTQ 0.0436752

DQTR -0.0549421

DQTS -0.13617

DQTT -0.123659

DQTV -0.237889

DQTW 0.501473

DQTY 0.271118

DQVA -0.471382

DQVC 0.56696

DQVD -0.424274

DQVE -0.431966

DQVF 0.194783

DQVG -0.785245

DQVH 0.170948

DQVI -0.0672994

DQVK -0.37317

DQVL -0.408379

DQVM 0.42231

DQVN -0.264045

DQVP -0.355792

DQVQ -0.107923

DQVR -0.210122

DQVS -0.381442

DQVT -0.248098

DQVV -0.227841

DQVW 0.638669

DQVY 0.214075

DQWA 0.258544

DQWC 1.11737

DQWD 0.304956

DQWE 0.281922

DQWF 1.04988

DQWG 0.00590634

DQWH 0.719417

DQWI 0.796998

DQWK 0.318502

DQWL 0.637983

DQWM 0.926689

DQWN 0.486443

DQWP 0.328373

DQWQ 0.59262

DQWR 0.590522

DQWS 0.319594

DQWT 0.404175

DQWV 0.67311

DQWW 1.27246

DQWY 1.1398

DQYA -0.0833859

DQYC 1.04826

DQYD 0.137447

DQYE 0.0303798

DQYF 0.672743

DQYG -0.236195

DQYH 0.614374

DQYI 0.356906

DQYK 0.111136

DQYL 0.0954723

DQYM 0.718699

DQYN 0.286632

DQYP 0.173785

DQYQ 0.401714

DQYR 0.323442

DQYS 0.097836

DQYT 0.20285

DQYV 0.191478

DQYW 1.09509

DQYY 0.749489

DRAA -0.986342

DRAC -0.0819724

DRAD -0.877405

DRAE -0.887087

DRAF -0.371941

DRAG -1.28865

DRAH -0.298736

DRAI -0.618329

DRAK -0.80385

DRAL -0.921617

DRAM -0.131116

DRAN -0.701932

DRAP -0.95315

DRAQ -0.579891

DRAR -0.434072

DRAS -0.759301

DRAT -0.787593

DRAV -0.808653

DRAW 0.0608876

DRAY -0.316627

DRCA -0.0245721

DRCC 1.97718

DRCD -0.156544

DRCE -0.218803

DRCF 0.596422

DRCG -0.284343

DRCH 0.539242

DRCI 0.315205

DRCK -0.0154262

DRCL 0.209492

DRCM 0.533661

DRCN 0.0646391

DRCP -0.0491035

DRCQ 0.243395

DRCR 0.201244

DRCS 0.0482299

DRCT 0.0425169

DRCV 0.277074

DRCW 0.922204

DRCY 0.633738

DRDA -0.730701

DRDC -0.12933

DRDD -0.545381

DRDE -0.540061

DRDF -0.271425

DRDG -0.914667

DRDH 0.0401418

DRDI -0.531745

DRDK 0.262444

DRDL -0.75901

DRDM -0.261286

DRDN -0.239059

DRDP -0.648218

DRDQ -0.0647857

DRDR 4.75261

DRDS -0.422999

DRDT -0.387666

DRDV -0.683729

DRDW 0.131774

DRDY -0.053833

DREA -0.861016

DREC -0.199814

DRED -0.729174

DREE -0.697196

DREF -0.355869

DREG -1.17922

DREH -0.152621

DREI -0.617277

DREK -0.288429

DREL -0.837496

DREM -0.235252

DREN -0.497266

DREP -0.813015

DREQ -0.377944

DRER 0.171896

DRES -0.765647

DRET -0.521048

DREV -0.735653

DREW 0.131616

DREY -0.221732

DRFA -0.397916

DRFC 0.612508

DRFD -0.260314

DRFE -0.358435

DRFF 0.357209

DRFG -0.681495

DRFH 0.344732

DRFI 0.0548551

DRFK -0.27051

DRFL -0.254932

DRFM 0.531146

DRFN -0.135515

DRFP -0.230289

DRFQ 0.00208545

DRFR -0.0458698

DRFS -0.235107

DRFT -0.160363

DRFV -0.124614

DRFW 0.775096

DRFY 0.359819

DRGA -1.28107

DRGC -0.395468

DRGD -0.936134

DRGE -1.07081

DRGF -0.58697

DRGG -1.99897

DRGH -0.59773

DRGI -0.886381

DRGK -0.742598

DRGL -1.11688

DRGM -0.545945

DRGN -0.797559

DRGP -1.1084

DRGQ -0.760821

DRGR -0.440227

DRGS -0.85403

DRGT -0.818273

DRGV -0.993286

DRGW -0.274717

DRGY -0.489188

DRHA -0.372201

DRHC 0.476614

DRHD -0.0202157

DRHE -0.146549

DRHF 0.342841

DRHG -0.608873

DRHH 0.50194

DRHI 0.00216198

DRHK -0.249695

DRHL -0.113335

DRHM 0.331597

DRHN -0.0410786

DRHP -0.219618

DRHQ -0.0331469

DRHR 0.0613925

DRHS -0.115471

DRHT -0.0446408

DRHV -0.0940008

DRHW 0.711658

DRHY 0.408646

DRIA -0.607703

DRIC 0.39454

DRID -0.574646

DRIE -0.601714

DRIF 0.0613611

DRIG -0.931076

DRIH 0.0353651

DRII -0.171803

DRIK -0.517278

DRIL -0.532535

DRIM 0.311362

DRIN -0.3973

DRIP -0.501482

DRIQ -0.261096

DRIR -0.347347

DRIS -0.528148

DRIT -0.387485

DRIV -0.360465

DRIW 0.501801

DRIY 0.110583

DRKA -0.863343

DRKC -0.0762854

DRKD -0.447195

DRKE -0.465264

DRKF -0.289774

DRKG -1.08989

DRKH -0.291664

DRKI -0.522534

DRKK -0.579424

DRKL -0.815441

DRKM -0.241977

DRKN -0.466532

DRKP -0.733228

DRKQ -0.338983

DRKR -0.533995

DRKS -0.642503

DRKT -0.550037

DRKV -0.67041

DRKW 0.050282

DRKY -0.143629

DRLA -0.955902

DRLC 0.296816

DRLD -0.840626

DRLE -0.856831

DRLF -0.246035

DRLG -1.16305

DRLH -0.112953

DRLI -0.542059

DRLK -0.774182

DRLL -0.899194

DRLM 0.068013

DRLN -0.650066

DRLP -0.710423

DRLQ -0.419748

DRLR -0.538713

DRLS -0.815169

DRLT -0.706419

DRLV -0.741681

DRLW 0.34844

DRLY -0.197213

DRMA -0.166212

DRMC 0.606564

DRMD -0.205252

DRME -0.207734

DRMF 0.532352

DRMG -0.693274

DRMH 0.230907

DRMI 0.28426

DRMK -0.175706

DRML 0.0675142

DRMM 0.633635

DRMN -0.139218

DRMP -0.30604

DRMQ 0.0208874

DRMR 0.0242908

DRMS -0.210641

DRMT -0.0545132

DRMV 0.121285

DRMW 0.639363

DRMY 0.494906

DRNA -0.682162

DRNC 0.0684438

DRND -0.368109

DRNE -0.4592

DRNF -0.126464

DRNG -0.813908

DRNH -0.0210538

DRNI -0.424096

DRNK -0.330275

DRNL -0.632062

DRNM -0.125459

DRNN -0.139955

DRNP -0.481169

DRNQ -0.142466

DRNR -0.0699165

DRNS -0.411571

DRNT -0.338718

DRNV -0.517284

DRNW 0.329803

DRNY 0.0310047

DRPA -0.708017

DRPC 0.0217783

DRPD -0.466898

DRPE -0.467713

DRPF -0.0942202

DRPG -0.842339

DRPH -0.0620282

DRPI -0.417825

DRPK -0.519846

DRPL -0.611116

DRPM -0.0704124

DRPN -0.347052

DRPP -0.506699

DRPQ -0.222946

DRPR -0.284534

DRPS -0.433973

DRPT -0.408783

DRPV -0.460571

DRPW 0.373226

DRPY 0.00645161

DRQA -0.542336

DRQC 0.226658

DRQD -0.387658

DRQE -0.391896

DRQF 0.0233772

DRQG -0.768056

DRQH -0.0489135

DRQI -0.289766

DRQK -0.338334

DRQL -0.474385

DRQM -0.0218492

DRQN -0.188792

DRQP -0.471768

DRQQ 0.0089016

DRQR -0.00782132

DRQS -0.311233

DRQT -0.255068

DRQV -0.409964

DRQW 0.361506

DRQY 0.134385

DRRA -0.661269

DRRC 0.118312

DRRD -0.296371

DRRE -0.325886

DRRF -0.0546966

DRRG -0.908514

DRRH -0.00461698

DRRI -0.340796

DRRK -0.551362

DRRL -0.592604

DRRM 0.0479157

DRRN -0.339354

DRRP -0.480266

DRRQ -0.164499

DRRR -0.224768

DRRS -0.438305

DRRT -0.423508

DRRV -0.484838

DRRW 0.306616

DRRY 0.0434628

DRSA -0.851628

DRSC 0.0957906

DRSD -0.554326

DRSE -0.629593

DRSF -0.227922

DRSG -0.90148

DRSH -0.0931408

DRSI -0.557385

DRSK -0.598233

DRSL -0.788515

DRSM -0.173548

DRSN -0.416868

DRSP -0.6814

DRSQ -0.290223

DRSR 0.0398831

DRSS -0.456221

DRST -0.464612

DRSV -0.691859

DRSW 0.151634

DRSY -0.125469

DRTA -0.786242

DRTC 0.193517

DRTD -0.51711

DRTE -0.514271

DRTF -0.127584

DRTG -0.886133

DRTH -0.0480225

DRTI -0.391565

DRTK -0.523607

DRTL -0.702529

DRTM -0.0306957

DRTN -0.347606

DRTP -0.556675

DRTQ -0.213136

DRTR -0.338448

DRTS -0.50994

DRTT -0.379998

DRTV -0.529664

DRTW 0.266803

DRTY -0.0466714

DRVA -0.79712

DRVC 0.308144

DRVD -0.707501

DRVE -0.732676

DRVF -0.113816

DRVG -1.06157

DRVH -0.08254

DRVI -0.365426

DRVK -0.661363

DRVL -0.738633

DRVM 0.144541

DRVN -0.542846

DRVP -0.629462

DRVQ -0.397318

DRVR -0.437912

DRVS -0.674888

DRVT -0.546826

DRVV -0.553037

DRVW 0.380526

DRVY -0.0877769

DRWA 0.0114815

DRWC 0.92608

DRWD 0.0819132

DRWE 0.0554364

DRWF 0.799767

DRWG -0.211877

DRWH 0.526498

DRWI 0.539413

DRWK 0.0895848

DRWL 0.35706

DRWM 0.72623

DRWN 0.269208

DRWP 0.118999

DRWQ 0.376947

DRWR 0.364868

DRWS 0.0993903

DRWT 0.181347

DRWV 0.408448

DRWW 1.07446

DRWY 0.890912

DRYA -0.365089

DRYC 0.803892

DRYD -0.128967

DRYE -0.23801

DRYF 0.381073

DRYG -0.502945

DRYH 0.367975

DRYI 0.0663698

DRYK -0.172002

DRYL -0.220014

DRYM 0.473431

DRYN 0.0173018

DRYP -0.0900435

DRYQ 0.116766

DRYR 0.0687976

DRYS -0.183919

DRYT -0.0832448

DRYV -0.107809

DRYW 0.846506

DRYY 0.455625

DSAA -0.901479

DSAC -0.0023191

DSAD -0.784364

DSAE -0.790617

DSAF -0.28342

DSAG -1.21539

DSAH -0.223231

DSAI -0.54598

DSAK -0.733742

DSAL -0.849085

DSAM -0.0617907

DSAN -0.634297

DSAP -0.882142

DSAQ -0.484951

DSAR -0.565463

DSAS -0.705359

DSAT -0.685261

DSAV -0.712783

DSAW 0.136937

DSAY -0.228196

DSCA 0.0490792

DSCC 2.05901

DSCD -0.0880387

DSCE -0.148197

DSCF 0.667647

DSCG -0.214587

DSCH 0.604988

DSCI 0.391008

DSCK 0.0559812

DSCL 0.292198

DSCM 0.598418

DSCN 0.136224

DSCP 0.0174749

DSCQ 0.3096

DSCR 0.270412

DSCS 0.132263

DSCT 0.111882

DSCV 0.35184

DSCW 0.986823

DSCY 0.708184

DSDA -0.0594726

DSDC -0.0489156

DSDD -0.402451

DSDE -0.382638

DSDF -0.106544

DSDG -0.769554

DSDH 0.0229418

DSDI -0.402539

DSDK 0.0675261

DSDL -0.559602

DSDM -0.129697

DSDN -0.0667822

DSDP -0.451089

DSDQ -0.211401

DSDR -0.0642986

DSDS 4.3627

DSDT 0.380321

DSDV -0.583669

DSDW 0.206666

DSDY 0.0451798

DSEA -0.772811

DSEC -0.136327

DSED -0.649416

DSEE -0.565542

DSEF -0.283216

DSEG -1.09434

DSEH -0.0818708

DSEI -0.525111

DSEK -0.374983

DSEL -0.778372

DSEM -0.168088

DSEN -0.412059

DSEP -0.739706

DSEQ -0.3032

DSER -0.222092

DSES -0.366281

DSET -0.5688

DSEV -0.683482

DSEW 0.201696

DSEY -0.158

DSFA -0.313124

DSFC 0.688771

DSFD -0.171743

DSFE -0.267579

DSFF 0.446373

DSFG -0.594684

DSFH 0.422595

DSFI 0.14213

DSFK -0.192861

DSFL -0.155315

DSFM 0.61028

DSFN -0.0513961

DSFP -0.152858

DSFQ 0.0716698

DSFR 0.00911617

DSFS -0.11271

DSFT -0.0679679

DSFV -0.041261

DSFW 0.848357

DSFY 0.446983

DSGA -1.18724

DSGC -0.325957

DSGD -0.857152

DSGE -0.969178

DSGF -0.508535

DSGG -1.93247

DSGH -0.530099

DSGI -0.797435

DSGK -0.848517

DSGL -1.03183

DSGM -0.475351

DSGN -0.706514

DSGP -1.03192

DSGQ -0.663587

DSGR -0.667066

DSGS -0.822821

DSGT -0.735517

DSGV -0.898908

DSGW -0.202171

DSGY -0.40289

DSHA -0.293225

DSHC 0.536665

DSHD 0.0572178

DSHE -0.0586131

DSHF 0.424056

DSHG -0.536819

DSHH 0.565016

DSHI 0.0740628

DSHK -0.192903

DSHL -0.0349514

DSHM 0.397271

DSHN 0.030942

DSHP -0.144834

DSHQ 0.0397091

DSHR 0.0896246

DSHS 0.00907683

DSHT 0.0303619

DSHV -0.0104618

DSHW 0.779482

DSHY 0.484408

DSIA -0.544045

DSIC 0.473963

DSID -0.491375

DSIE -0.520938

DSIF 0.146126

DSIG -0.847074

DSIH 0.112801

DSII -0.0796084

DSIK -0.453094

DSIL -0.440494

DSIM 0.394598

DSIN -0.320875

DSIP -0.41161

DSIQ -0.165943

DSIR -0.255819

DSIS -0.432062

DSIT -0.293356

DSIV -0.279181

DSIW 0.578813

DSIY 0.198651

DSKA -0.768337

DSKC -0.00696373

DSKD -0.395484

DSKE -0.388809

DSKF -0.204268

DSKG -1.01102

DSKH -0.216108

DSKI -0.461717

DSKK -0.507745

DSKL -0.711903

DSKM -0.17661

DSKN -0.381593

DSKP -0.655991

DSKQ -0.263566

DSKR -0.476376

DSKS -0.490884

DSKT -0.486181

DSKV -0.574918

DSKW 0.115951

DSKY -0.0711515

DSLA -0.863594

DSLC 0.378593

DSLD -0.752932

DSLE -0.760895

DSLF -0.16559

DSLG -1.07197

DSLH -0.039366

DSLI -0.439934

DSLK -0.689098

DSLL -0.799425

DSLM 0.15411

DSLN -0.566412

DSLP -0.635768

DSLQ -0.353344

DSLR -0.486288

DSLS -0.682237

DSLT -0.608113

DSLV -0.648332

DSLW 0.432638

DSLY -0.102024

DSMA -0.0803053

DSMC 0.672058

DSMD -0.129306

DSME -0.128807

DSMF 0.611285

DSMG -0.624511

DSMH 0.296905

DSMI 0.370527

DSMK -0.0912728

DSML 0.152055

DSMM 0.704599

DSMN -0.0689683

DSMP -0.239974

DSMQ 0.0906825

DSMR 0.0947201

DSMS -0.128747

DSMT 0.012059

DSMV 0.205753

DSMW 0.704764

DSMY 0.572976

DSNA -0.533909

DSNC 0.135551

DSND -0.269525

DSNE -0.363489

DSNF -0.0421221

DSNG -0.718057

DSNH 0.0539618

DSNI -0.349997

DSNK -0.29309

DSNL -0.561002

DSNM -0.0536704

DSNN -0.0517082

DSNP -0.395426

DSNQ -0.0825291

DSNR -0.132548

DSNS -0.0238063

DSNT -0.293881

DSNV -0.471985

DSNW 0.400635

DSNY 0.118987

DSPA -0.624655

DSPC 0.0911915

DSPD -0.379161

DSPE -0.386625

DSPF -0.0124907

DSPG -0.762076

DSPH 0.0215914

DSPI -0.337382

DSPK -0.41103

DSPL -0.527581

DSPM 0.00253415

DSPN -0.27528

DSPP -0.425026

DSPQ -0.165993

DSPR -0.21699

DSPS -0.31299

DSPT -0.327573

DSPV -0.387529

DSPW 0.445782

DSPY 0.0815949

DSQA -0.453574

DSQC 0.29568

DSQD -0.297661

DSQE -0.312252

DSQF 0.11696

DSQG -0.691478

DSQH 0.0323963

DSQI -0.211678

DSQK -0.216379

DSQL -0.380441

DSQM 0.0525899

DSQN -0.120083

DSQP -0.407582

DSQQ 0.0879855

DSQR -0.0893509

DSQS -0.283394

DSQT -0.169876

DSQV -0.320498

DSQW 0.431155

DSQY 0.215503

DSRA -0.598713

DSRC 0.189485

DSRD -0.175014

DSRE -0.238042

DSRF 0.0296307

DSRG -0.836503

DSRH 0.0799248

DSRI -0.252137

DSRK -0.459914

DSRL -0.445789

DSRM 0.120222

DSRN -0.250057

DSRP -0.399858

DSRQ -0.0805433

DSRR -0.173965

DSRS -0.371271

DSRT -0.342643

DSRV -0.391381

DSRW 0.381088

DSRY 0.12271

DSSA -0.745749

DSSC 0.170433

DSSD -0.468024

DSSE -0.527617

DSSF -0.147484

DSSG -0.745672

DSSH -0.0326302

DSSI -0.458567

DSSK -0.496659

DSSL -0.677123

DSSM -0.0985062

DSSN -0.318905

DSSP -0.602305

DSSQ -0.205077

DSSR -0.320416

DSSS -0.35074

DSST -0.371377

DSSV -0.598496

DSSW 0.237189

DSSY -0.055696

DSTA -0.683551

DSTC 0.260998

DSTD -0.41484

DSTE -0.459653

DSTF -0.0356283

DSTG -0.792823

DSTH 0.0279558

DSTI -0.323194

DSTK -0.46372

DSTL -0.618753

DSTM 0.0447838

DSTN -0.268363

DSTP -0.480225

DSTQ -0.148619

DSTR -0.277319

DSTS -0.350271

DSTT -0.326135

DSTV -0.441859

DSTW 0.344144

DSTY 0.0700774

DSVA -0.690453

DSVC 0.385452

DSVD -0.628261

DSVE -0.6586

DSVF -0.0280859

DSVG -0.990925

DSVH -0.00372267

DSVI -0.29079

DSVK -0.578426

DSVL -0.643343

DSVM 0.228064

DSVN -0.464983

DSVP -0.553776

DSVQ -0.293468

DSVR -0.418631

DSVS -0.550474

DSVT -0.456133

DSVV -0.465402

DSVW 0.458114

DSVY -0.00539041

DSWA 0.0859277

DSWC 0.990775

DSWD 0.159116

DSWE 0.123902

DSWF 0.875989

DSWG -0.141642

DSWH 0.591378

DSWI 0.620732

DSWK 0.161026

DSWL 0.440722

DSWM 0.792521

DSWN 0.338342

DSWP 0.187358

DSWQ 0.446746

DSWR 0.434216

DSWS 0.17396

DSWT 0.254795

DSWV 0.487059

DSWW 1.14082

DSWY 0.968091

DSYA -0.283129

DSYC 0.879518

DSYD -0.0557652

DSYE -0.167196

DSYF 0.465343

DSYG -0.423872

DSYH 0.441236

DSYI 0.146614

DSYK -0.0790517

DSYL -0.135959

DSYM 0.548174

DSYN 0.109282

DSYP -0.00479126

DSYQ 0.196357

DSYR 0.154014

DSYS -0.0369782

DSYT 0.0216289

DSYV -0.0143902

DSYW 0.922011

DSYY 0.530787

DTAA -0.882857

DTAC -0.00570893

DTAD -0.806862

DTAE -0.807244

DTAF -0.292606

DTAG -1.20649

DTAH -0.210285

DTAI -0.463434

DTAK -0.743548

DTAL -0.876861

DTAM -0.061559

DTAN -0.633087

DTAP -0.882369

DTAQ -0.472755

DTAR -0.576198

DTAS -0.80343

DTAT -0.675754

DTAV -0.6523

DTAW 0.136331

DTAY -0.240861

DTCA 0.0509331

DTCC 2.05985

DTCD -0.0868347

DTCE -0.147132

DTCF 0.671904

DTCG -0.213377

DTCH 0.60669

DTCI 0.391277

DTCK 0.0588226

DTCL 0.291013

DTCM 0.599279

DTCN 0.133446

DTCP 0.018687

DTCQ 0.312494

DTCR 0.267767

DTCS 0.120795

DTCT 0.119084

DTCV 0.362785

DTCW 0.988338

DTCY 0.70739

DTDA -0.425407

DTDC -0.0580113

DTDD -0.431767

DTDE -0.366671

DTDF -0.0679197

DTDG -0.871062

DTDH 0.0479829

DTDI -0.205207

DTDK -0.0354998

DTDL -0.467158

DTDM -0.0647471

DTDN 0.035856

DTDP -0.589218

DTDQ -0.0387022

DTDR -0.0825753

DTDS 0.326711

DTDT 4.41804

DTDV -0.527862

DTDW 0.199447

DTDY 0.00300741

DTEA -0.783266

DTEC -0.13535

DTED -0.651398

DTEE -0.632516

DTEF -0.272122

DTEG -1.10595

DTEH -0.0785263

DTEI -0.514309

DTEK -0.335107

DTEL -0.738642

DTEM -0.171593

DTEN -0.424031

DTEP -0.731437

DTEQ -0.306033

DTER -0.244876

DTES -0.686342

DTET -0.141056

DTEV -0.681262

DTEW 0.206037

DTEY -0.0818028

DTFA -0.313275

DTFC 0.68813

DTFD -0.173162

DTFE -0.27344

DTFF 0.443817

DTFG -0.590626

DTFH 0.423164

DTFI 0.142383

DTFK -0.191033

DTFL -0.162611

DTFM 0.612259

DTFN -0.059808

DTFP -0.125062

DTFQ 0.0750291

DTFR -0.0104604

DTFS -0.111229

DTFT -0.045167

DTFV -0.0470822

DTFW 0.852678

DTFY 0.446587

DTGA -1.19274

DTGC -0.32416

DTGD -0.829305

DTGE -0.967583

DTGF -0.500341

DTGG -1.93234

DTGH -0.526732

DTGI -0.807682

DTGK -0.823167

DTGL -1.0317

DTGM -0.473032

DTGN -0.703574

DTGP -1.02962

DTGQ -0.664083

DTGR -0.672877

DTGS -0.861595

DTGT -0.713418

DTGV -0.893343

DTGW -0.197441

DTGY -0.405917

DTHA -0.281152

DTHC 0.538729

DTHD 0.0687816

DTHE -0.0624769

DTHF 0.416188

DTHG -0.535859

DTHH 0.568825

DTHI 0.0693889

DTHK -0.197782

DTHL -0.0389574

DTHM 0.402556

DTHN 0.0277185

DTHP -0.139353

DTHQ 0.0443883

DTHR 0.0897753

DTHS -0.0309136

DTHT 0.0523241

DTHV -0.0162697

DTHW 0.779458

DTHY 0.493742

DTIA -0.52463

DTIC 0.47206

DTID -0.477343

DTIE -0.513494

DTIF 0.14588

DTIG -0.853733

DTIH 0.120287

DTII -0.0733814

DTIK -0.41586

DTIL -0.435778

DTIM 0.395274

DTIN -0.316731

DTIP -0.423239

DTIQ -0.185504

DTIR -0.254828

DTIS -0.446224

DTIT -0.302103

DTIV -0.290757

DTIW 0.587496

DTIY 0.204527

DTKA -0.765064

DTKC -0.00630808

DTKD -0.371895

DTKE -0.380426

DTKF -0.203631

DTKG -1.0095

DTKH -0.214902

DTKI -0.460661

DTKK -0.510971

DTKL -0.721514

DTKM -0.174229

DTKN -0.365819

DTKP -0.646952

DTKQ -0.27014

DTKR -0.47435

DTKS -0.57916

DTKT -0.447225

DTKV -0.578604

DTKW 0.119598

DTKY -0.0942276

DTLA -0.851571

DTLC 0.380829

DTLD -0.752844

DTLE -0.762106

DTLF -0.162023

DTLG -1.09129

DTLH -0.0351989

DTLI -0.45342

DTLK -0.692855

DTLL -0.807746

DTLM 0.154786

DTLN -0.572078

DTLP -0.638664

DTLQ -0.35022

DTLR -0.48761

DTLS -0.742612

DTLT -0.615519

DTLV -0.636125

DTLW 0.434171

DTLY -0.103844

DTMA -0.0833499

DTMC 0.67287

DTMD -0.131065

DTME -0.118462

DTMF 0.61218

DTMG -0.619898

DTMH 0.298546

DTMI 0.366127

DTMK -0.0937948

DTML 0.152687

DTMM 0.706619

DTMN -0.063664

DTMP -0.23568

DTMQ 0.0923448

DTMR 0.100672

DTMS -0.15309

DTMT 0.0194981

DTMV 0.217388

DTMW 0.705197

DTMY 0.570435

DTNA -0.620029

DTNC 0.13847

DTND -0.280126

DTNE -0.420595

DTNF -0.0599263

DTNG -0.739449

DTNH 0.0551147

DTNI -0.35669

DTNK -0.225173

DTNL -0.567244

DTNM -0.0625763

DTNN -0.0785375

DTNP -0.409405

DTNQ -0.0902967

DTNR -0.210507

DTNS -0.337917

DTNT 0.0453475

DTNV -0.454512

DTNW 0.40324

DTNY 0.157562

DTPA -0.62235

DTPC 0.0892603

DTPD -0.389216

DTPE -0.37188

DTPF -0.00843763

DTPG -0.761666

DTPH 0.0214293

DTPI -0.343408

DTPK -0.433339

DTPL -0.521832

DTPM 0.00302672

DTPN -0.280547

DTPP -0.429355

DTPQ -0.157151

DTPR -0.225219

DTPS -0.363482

DTPT -0.322649

DTPV -0.306946

DTPW 0.447016

DTPY 0.0784378

DTQA -0.465334

DTQC 0.297407

DTQD -0.313467

DTQE -0.30553

DTQF 0.164712

DTQG -0.686304

DTQH 0.0411167

DTQI -0.219705

DTQK -0.22361

DTQL -0.37005

DTQM 0.0479841

DTQN -0.132243

DTQP -0.414227

DTQQ 0.0906396

DTQR -0.0880253

DTQS -0.234879

DTQT -0.200489

DTQV -0.299969

DTQW 0.428533

DTQY 0.228984

DTRA -0.599826

DTRC 0.190031

DTRD -0.215645

DTRE -0.24262

DTRF 0.0198522

DTRG -0.835066

DTRH 0.0693004

DTRI -0.25283

DTRK -0.451091

DTRL -0.511294

DTRM 0.124945

DTRN -0.253945

DTRP -0.408082

DTRQ -0.080013

DTRR -0.15539

DTRS -0.373238

DTRT -0.331478

DTRV -0.395455

DTRW 0.381937

DTRY 0.140902

DTSA -0.796056

DTSC 0.169074

DTSD -0.462836

DTSE -0.538357

DTSF -0.162296

DTSG -0.797077

DTSH -0.0242107

DTSI -0.473972

DTSK -0.511225

DTSL -0.709998

DTSM -0.0939114

DTSN -0.322033

DTSP -0.597476

DTSQ -0.21857

DTSR -0.353505

DTSS -0.141463

DTST -0.226821

DTSV -0.527166

DTSW 0.232941

DTSY -0.0561266

DTTA -0.70126

DTTC 0.265509

DTTD -0.453091

DTTE -0.463381

DTTF -0.0386381

DTTG -0.810326

DTTH 0.0259535

DTTI -0.309985

DTTK -0.399151

DTTL -0.621426

DTTM 0.0453198

DTTN -0.270035

DTTP -0.472435

DTTQ -0.137206

DTTR -0.275259

DTTS -0.322816

DTTT -0.334775

DTTV -0.470011

DTTW 0.341441

DTTY 0.298088

DTVA -0.705298

DTVC 0.391563

DTVD -0.619973

DTVE -0.654446

DTVF -0.0188568

DTVG -0.977109

DTVH -0.00462008

DTVI -0.298925

DTVK -0.591772

DTVL -0.645674

DTVM 0.224067

DTVN -0.457321

DTVP -0.547047

DTVQ -0.311209

DTVR -0.405323

DTVS -0.592638

DTVT -0.421583

DTVV -0.456315

DTVW 0.457759

DTVY -0.030051

DTWA 0.0941513

DTWC 0.991942

DTWD 0.151982

DTWE 0.130954

DTWF 0.877841

DTWG -0.141864

DTWH 0.592259

DTWI 0.618234

DTWK 0.165432

DTWL 0.443566

DTWM 0.793798

DTWN 0.339688

DTWP 0.188687

DTWQ 0.447884

DTWR 0.433307

DTWS 0.165584

DTWT 0.252812

DTWV 0.48936

DTWW 1.14644

DTWY 0.969611

DTYA -0.285907

DTYC 0.881119

DTYD -0.059042

DTYE -0.16637

DTYF 0.468488

DTYG -0.427725

DTYH 0.443775

DTYI 0.153183

DTYK -0.0424411

DTYL -0.141841

DTYM 0.553244

DTYN 0.0995777

DTYP -0.011085

DTYQ 0.196451

DTYR 0.122599

DTYS -0.0961909

DTYT 0.0119224

DTYV -0.0171525

DTYW 0.926762

DTYY 0.53195

DVAA -0.909402

DVAC -0.0262086

DVAD -0.818885

DVAE -0.828249

DVAF -0.289081

DVAG -1.23001

DVAH -0.240008

DVAI -0.582763

DVAK -0.759837

DVAL -0.851867

DVAM -0.0833857

DVAN -0.651899

DVAP -0.888393

DVAQ -0.493493

DVAR -0.602713

DVAS -0.809134

DVAT -0.705053

DVAV -0.642253

DVAW 0.117444

DVAY -0.251563

DVCA 0.0313542

DVCC 2.03726

DVCD -0.105266

DVCE -0.166895

DVCF 0.652043

DVCG -0.232462

DVCH 0.588012

DVCI 0.374913

DVCK 0.0372581

DVCL 0.267463

DVCM 0.582369

DVCN 0.115489

DVCP 0.0004642

DVCQ 0.29279

DVCR 0.249384

DVCS 0.102126

DVCT 0.0953023

DVCV 0.340097

DVCW 0.970171

DVCY 0.687778

DVDA -0.517361

DVDC -0.0575736

DVDD -0.514792

DVDE -0.528607

DVDF -0.213283

DVDG -0.88865

DVDH 0.00903535

DVDI 0.279324

DVDK -0.306746

DVDL 0.396921

DVDM -0.180269

DVDN -0.257686

DVDP -0.609039

DVDQ -0.188494

DVDR -0.200659

DVDS -0.4593

DVDT -0.349883

DVDV 4.25327

DVDW 0.179523

DVDY 0.0216455

DVEA -0.793997

DVEC -0.153125

DVED -0.67549

DVEE -0.631504

DVEF -0.292676

DVEG -1.12091

DVEH -0.0947468

DVEI -0.580846

DVEK -0.354762

DVEL -0.836472

DVEM -0.16163

DVEN -0.443252

DVEP -0.758321

DVEQ -0.264099

DVER -0.267623

DVES -0.67351

DVET -0.569141

DVEV -0.232282

DVEW 0.187413

DVEY -0.174045

DVFA -0.33373

DVFC 0.667513

DVFD -0.195123

DVFE -0.283754

DVFF 0.431742

DVFG -0.603561

DVFH 0.402787

DVFI 0.11352

DVFK -0.210801

DVFL -0.194337

DVFM 0.589041

DVFN -0.0798604

DVFP -0.170256

DVFQ 0.0530746

DVFR -0.031353

DVFS -0.168643

DVFT -0.0944376

DVFV -0.0290167

DVFW 0.827042

DVFY 0.426982

DVGA -1.21668

DVGC -0.343759

DVGD -0.868451

DVGE -0.996411

DVGF -0.531119

DVGG -1.95103

DVGH -0.543603

DVGI -0.818356

DVGK -0.858159

DVGL -1.01462

DVGM -0.496944

DVGN -0.727073

DVGP -1.05131

DVGQ -0.686629

DVGR -0.699804

DVGS -0.883272

DVGT -0.750894

DVGV -0.911519

DVGW -0.220201

DVGY -0.414412

DVHA -0.306945

DVHC 0.519949

DVHD 0.0386369

DVHE -0.0864713

DVHF 0.402373

DVHG -0.555538

DVHH 0.549089

DVHI 0.0516629

DVHK -0.206813

DVHL -0.0668066

DVHM 0.380614

DVHN 0.0129285

DVHP -0.158877

DVHQ 0.0188103

DVHR 0.0691741

DVHS -0.0532696

DVHT 0.0104373

DVHV 0.0131307

DVHW 0.76123

DVHY 0.461987

DVIA -0.559279

DVIC 0.452612

DVID -0.508742

DVIE -0.538718

DVIF 0.12947

DVIG -0.875633

DVIH 0.0932937

DVII -0.0972223

DVIK -0.464969

DVIL -0.466737

DVIM 0.375771

DVIN -0.337039

DVIP -0.441754

DVIQ -0.19852

DVIR -0.281385

DVIS -0.458499

DVIT -0.318124

DVIV -0.315274

DVIW 0.559274

DVIY 0.176249

DVKA -0.800097

DVKC -0.0196953

DVKD -0.402745

DVKE -0.409144

DVKF -0.240714

DVKG -1.02906

DVKH -0.232577

DVKI -0.465211

DVKK -0.523322

DVKL -0.743843

DVKM -0.201373

DVKN -0.409041

DVKP -0.67012

DVKQ -0.292657

DVKR -0.487314

DVKS -0.582977

DVKT -0.492206

DVKV -0.579707

DVKW 0.0963812

DVKY -0.0813944

DVLA -0.853499

DVLC 0.359034

DVLD -0.778221

DVLE -0.789827

DVLF -0.177329

DVLG -1.10889

DVLH -0.0591519

DVLI -0.47867

DVLK -0.71231

DVLL -0.845757

DVLM 0.130637

DVLN -0.596925

DVLP -0.661821

DVLQ -0.378968

DVLR -0.506032

DVLS -0.753685

DVLT -0.632475

DVLV -0.650521

DVLW 0.410672

DVLY -0.128215

DVMA -0.0954373

DVMC 0.655068

DVMD -0.150502

DVME -0.140972

DVMF 0.590543

DVMG -0.640114

DVMH 0.280475

DVMI 0.34597

DVMK -0.115858

DVML 0.12446

DVMM 0.690675

DVMN -0.0843291

DVMP -0.255583

DVMQ 0.0739346

DVMR 0.0799725

DVMS -0.160843

DVMT -0.00266337

DVMV 0.180436

DVMW 0.688076

DVMY 0.552088

DVNA -0.640603

DVNC 0.118109

DVND -0.32832

DVNE -0.429685

DVNF -0.0628874

DVNG -0.76353

DVNH 0.0292392

DVNI -0.37559

DVNK -0.33322

DVNL -0.618138

DVNM -0.0726261

DVNN -0.10573

DVNP -0.433273

DVNQ -0.124736

DVNR -0.214389

DVNS -0.341475

DVNT -0.293846

DVNV -0.30251

DVNW 0.382719

DVNY 0.0878446

DVPA -0.630166

DVPC 0.0721834

DVPD -0.407866

DVPE -0.402679

DVPF -0.0335913

DVPG -0.786124

DVPH -0.00678277

DVPI -0.346846

DVPK -0.454284

DVPL -0.550045

DVPM -0.0172074

DVPN -0.302415

DVPP -0.410724

DVPQ -0.176895

DVPR -0.249794

DVPS -0.402728

DVPT -0.340931

DVPV -0.401023

DVPW 0.425395

DVPY 0.0619521

DVQA -0.462496

DVQC 0.278087

DVQD -0.335779

DVQE -0.327288

DVQF 0.0913618

DVQG -0.711956

DVQH 0.00771379

DVQI -0.239616

DVQK -0.267222

DVQL -0.338986

DVQM 0.0226059

DVQN -0.154384

DVQP -0.439947

DVQQ 0.109316

DVQR -0.118102

DVQS -0.292516

DVQT -0.2163

DVQV -0.173497

DVQW 0.413972

DVQY 0.191197

DVRA -0.618987

DVRC 0.174069

DVRD -0.236015

DVRE -0.262935

DVRF 0.00641251

DVRG -0.857251

DVRH 0.0576441

DVRI -0.282189

DVRK -0.489477

DVRL -0.503256

DVRM 0.101746

DVRN -0.224566

DVRP -0.427927

DVRQ -0.108197

DVRR -0.193284

DVRS -0.401955

DVRT -0.356298

DVRV -0.404887

DVRW 0.363528

DVRY 0.108044

DVSA -0.797305

DVSC 0.148904

DVSD -0.502361

DVSE -0.55823

DVSF -0.163281

DVSG -0.770087

DVSH -0.0490582

DVSI -0.484943

DVSK -0.51465

DVSL -0.713717

DVSM -0.122146

DVSN -0.333599

DVSP -0.625857

DVSQ -0.231201

DVSR -0.346347

DVSS -0.44728

DVST -0.392937

DVSV -0.545201

DVSW 0.212639

DVSY -0.0770931

DVTA -0.694477

DVTC 0.243449

DVTD -0.456281

DVTE -0.471283

DVTF -0.0679755

DVTG -0.831814

DVTH 0.0102584

DVTI -0.350781

DVTK -0.466435

DVTL -0.639674

DVTM 0.032243

DVTN -0.287717

DVTP -0.500195

DVTQ -0.161297

DVTR -0.294087

DVTS -0.417434

DVTT -0.332814

DVTV -0.466739

DVTW 0.324407

DVTY 0.0215287

DVVA -0.721363

DVVC 0.365285

DVVD -0.648135

DVVE -0.67648

DVVF -0.0437243

DVVG -1.00093

DVVH -0.0179887

DVVI -0.306961

DVVK -0.596676

DVVL -0.677396

DVVM 0.199932

DVVN -0.483883

DVVP -0.566877

DVVQ -0.331223

DVVR -0.432461

DVVS -0.611648

DVVT -0.486398

DVVV -0.458209

DVVW 0.436852

DVVY -0.0137928

DVWA 0.0710275

DVWC 0.974101

DVWD 0.134978

DVWE 0.107694

DVWF 0.858032

DVWG -0.160912

DVWH 0.574618

DVWI 0.596278

DVWK 0.144392

DVWL 0.418676

DVWM 0.775093

DVWN 0.320932

DVWP 0.170097

DVWQ 0.428899

DVWR 0.413884

DVWS 0.147543

DVWT 0.228944

DVWV 0.46728

DVWW 1.12626

DVWY 0.947819

DVYA -0.306707

DVYC 0.860461

DVYD -0.076097

DVYE -0.197573

DVYF 0.441003

DVYG -0.44826

DVYH 0.423473

DVYI 0.130985

DVYK -0.101377

DVYL -0.174392

DVYM 0.529133

DVYN 0.074384

DVYP -0.0310516

DVYQ 0.180375

DVYR 0.109594

DVYS -0.118709

DVYT -0.0192528

DVYV -0.00362229

DVYW 0.904104

DVYY 0.527943

DWAA -0.502502

DWAC 0.230929

DWAD -0.461173

DWAE -0.450939

DWAF 0.0916753

DWAG -0.894785

DWAH 0.0493519

DWAI -0.141104

DWAK -0.383336

DWAL -0.454282

DWAM 0.231922

DWAN -0.310431

DWAP -0.598539

DWAQ -0.163691

DWAR -0.221822

DWAS -0.435336

DWAT -0.335581

DWAV -0.320837

DWAW 0.430229

DWAY 0.124503

DWCA 0.297309

DWCC 2.35943

DWCD 0.0899799

DWCE 0.0327227

DWCF 0.900472

DWCG -0.0236046

DWCH 0.75642

DWCI 0.631349

DWCK 0.253217

DWCL 0.590737

DWCM 0.744731

DWCN 0.303851

DWCP 0.179322

DWCQ 0.482831

DWCR 0.46717

DWCS 0.328169

DWCT 0.31219

DWCV 0.625788

DWCW 1.13177

DWCY 0.926126

DWDA -0.366808

DWDC 0.109447

DWDD -0.186492

DWDE -0.230543

DWDF 0.166475

DWDG -0.559047

DWDH 0.272651

DWDI -0.148254

DWDK 0.0223653

DWDL -0.337425

DWDM 0.0346768

DWDN 0.0929368

DWDP -0.327807

DWDQ 0.019768

DWDR 0.181524

DWDS -0.102284

DWDT -0.0558934

DWDV -0.253797

DWDW 3.35503

DWDY 0.343596

DWEA -0.455855

DWEC 0.049021

DWED -0.326301

DWEE -0.261135

DWEF 0.0476503

DWEG -0.822522

DWEH 0.179111

DWEI -0.188774

DWEK -0.0101254

DWEL -0.386919

DWEM 0.0665872

DWEN -0.109533

DWEP -0.470518

DWEQ 0.0088861

DWER 0.125816

DWES -0.323881

DWET -0.215962

DWEV -0.31224

DWEW 0.47895

DWEY 0.170135

DWFA 0.0483918

DWFC 0.918942

DWFD 0.143834

DWFE 0.0586233

DWFF 0.815443

DWFG -0.292721

DWFH 0.682287

DWFI 0.514935

DWFK 0.131006

DWFL 0.225509

DWFM 0.895021

DWFN 0.237975

DWFP 0.132721

DWFQ 0.35994

DWFR 0.309368

DWFS 0.176176

DWFT 0.267145

DWFV 0.339079

DWFW 1.105

DWFY 0.79264

DWGA -0.891073

DWGC -0.148237

DWGD -0.536396

DWGE -0.672342

DWGF -0.20425

DWGG -1.757

DWGH -0.321702

DWGI -0.492567

DWGK -0.51999

DWGL -0.677616

DWGM -0.264828

DWGN -0.418955

DWGP -0.791426

DWGQ -0.399696

DWGR -0.364315

DWGS -0.535692

DWGT -0.415612

DWGV -0.571419

DWGW -0.0039742

DWGY -0.0990925

DWHA -0.0292356

DWHC 0.682667

DWHD 0.321425

DWHE 0.207858

DWHF 0.681552

DWHG -0.330321

DWHH 0.762212

DWHI 0.327898

DWHK 0.025353

DWHL 0.281535

DWHM 0.566493

DWHN 0.245828

DWHP 0.0475023

DWHQ 0.230114

DWHR 0.326479

DWHS 0.209026

DWHT 0.284733

DWHV 0.27921

DWHW 0.949645

DWHY 0.734239

DWIA -0.160075

DWIC 0.724705

DWID -0.162455

DWIE -0.180053

DWIF 0.536929

DWIG -0.551471

DWIH 0.380824

DWII 0.315474

DWIK -0.10774

DWIL -0.0392873

DWIM 0.701363

DWIN -0.00791502

DWIP -0.130524

DWIQ 0.12047

DWIR 0.0871317

DWIS -0.0992014

DWIT 0.0526035

DWIV 0.111376

DWIW 0.852609

DWIY 0.560054

DWKA -0.423471

DWKC 0.185597

DWKD -0.00349259

DWKE -0.0123165

DWKF 0.117379

DWKG -0.720565

DWKH 0.00613165

DWKI -0.126986

DWKK -0.165041

DWKL -0.357843

DWKM 0.0510626

DWKN -0.0753269

DWKP -0.389138

DWKQ 0.0311861

DWKR -0.164028

DWKS -0.234843

DWKT -0.142689

DWKV -0.220297

DWKW 0.332419

DWKY 0.256619

DWLA -0.45664

DWLC 0.695636

DWLD -0.396665

DWLE -0.394363

DWLF 0.275562

DWLG -0.744504

DWLH 0.280169

DWLI -0.0462492

DWLK -0.321656

DWLL -0.384557

DWLM 0.497876

DWLN -0.232745

DWLP -0.300334

DWLQ 0.00081801

DWLR -0.115795

DWLS -0.353076

DWLT -0.235056

DWLV -0.234355

DWLW 0.749896

DWLY 0.278816

DWMA 0.203093

DWMC 0.824862

DWMD 0.0925992

DWME 0.110902

DWMF 0.893008

DWMG -0.435691

DWMH 0.455951

DWMI 0.666259

DWMK 0.134568

DWML 0.494622

DWMM 0.904547

DWMN 0.12777

DWMP -0.0624506

DWMQ 0.282217

DWMR 0.325476

DWMS 0.0781896

DWMT 0.25758

DWMV 0.521396

DWMW 0.85824

DWMY 0.829712

DWNA -0.299258

DWNC 0.308944

DWND 0.0125539

DWNE -0.0952203

DWNF 0.246871

DWNG -0.45686

DWNH 0.265649

DWNI -0.0418873

DWNK 0.00677872

DWNL -0.208195

DWNM 0.137621

DWNN 0.236103

DWNP -0.154217

DWNQ 0.176926

DWNR 0.116534

DWNS -0.0149267

DWNT 0.0416682

DWNV -0.137072

DWNW 0.686887

DWNY 0.420937

DWPA -0.292382

DWPC 0.258561

DWPD -0.0789065

DWPE -0.0510969

DWPF 0.300771

DWPG -0.479172

DWPH 0.228929

DWPI -0.025367

DWPK -0.13489

DWPL -0.173295

DWPM 0.207971

DWPN 0.0004704

DWPP -0.174247

DWPQ 0.110117

DWPR 0.0788145

DWPS -0.0635357

DWPT -0.00861216

DWPV -0.0255864

DWPW 0.665592

DWPY 0.38588

DWQA -0.139724

DWQC 0.465238

DWQD -0.0303018

DWQE -0.00674605

DWQF 0.395683

DWQG -0.428288

DWQH 0.216775

DWQI 0.085896

DWQK 0.064889

DWQL -0.0240753

DWQM 0.234256

DWQN 0.147352

DWQP -0.186676

DWQQ 0.370399

DWQR 0.200175

DWQS 0.0277593

DWQT 0.108904

DWQV 0.00317812

DWQW 0.629522

DWQY 0.507062

DWRA -0.256888

DWRC 0.376252

DWRD 0.135722

DWRE 0.120869

DWRF 0.360438

DWRG -0.555548

DWRH 0.305903

DWRI 0.0813773

DWRK -0.159859

DWRL -0.136562

DWRM 0.356261

DWRN 0.0381958

DWRP -0.13368

DWRQ 0.209214

DWRR 0.154507

DWRS -0.0444164

DWRT -0.0154791

DWRV -0.0431716

DWRW 0.605492

DWRY 0.454478

DWSA -0.438241

DWSC 0.384561

DWSD -0.147986

DWSE -0.195236

DWSF 0.177778

DWSG -0.496249

DWSH 0.226979

DWSI -0.135309

DWSK -0.158612

DWSL -0.327546

DWSM 0.13263

DWSN 0.0085156

DWSP -0.326844

DWSQ 0.107717

DWSR 0.00761032

DWSS -0.0571628

DWST -0.021204

DWSV -0.236966

DWSW 0.486647

DWSY 0.298161

DWTA -0.328534

DWTC 0.48175

DWTD -0.0911374

DWTE -0.10311

DWTF 0.315095

DWTG -0.491453

DWTH 0.282005

DWTI 0.0267818

DWTK -0.114153

DWTL -0.22335

DWTM 0.29209

DWTN 0.0551336

DWTP -0.193082

DWTQ 0.172794

DWTR 0.0581498

DWTS -0.05339

DWTT 0.054297

DWTV -0.0831201

DWTW 0.577898

DWTY 0.382095

DWVA -0.304259

DWVC 0.665061

DWVD -0.282766

DWVE -0.302463

DWVF 0.35546

DWVG -0.659332

DWVH 0.280305

DWVI 0.10302

DWVK -0.221172

DWVL -0.229441

DWVM 0.543087

DWVN -0.139971

DWVP -0.234914

DWVQ 0.0116458

DWVR -0.0635149

DWVS -0.235512

DWVT -0.0827692

DWVV -0.0557013

DWVW 0.747694

DWVY 0.367795

DWWA 0.338274

DWWC 1.13602

DWWD 0.359583

DWWE 0.342725

DWWF 1.14376

DWWG 0.0543993

DWWH 0.740466

DWWI 0.892502

DWWK 0.371984

DWWL 0.761722

DWWM 0.95481

DWWN 0.533103

DWWP 0.365915

DWWQ 0.632546

DWWR 0.653042

DWWS 0.375917

DWWT 0.463813

DWWV 0.778237

DWWW 1.29734

DWWY 1.22513

DWYA 0.0568771

DWYC 1.12718

DWYD 0.275121

DWYE 0.147048

DWYF 0.843912

DWYG -0.126597

DWYH 0.690747

DWYI 0.504875

DWYK 0.243683

DWYL 0.236002

DWYM 0.82036

DWYN 0.399701

DWYP 0.278177

DWYQ 0.49551

DWYR 0.454165

DWYS 0.223158

DWYT 0.327918

DWYV 0.349244

DWYW 1.19529

DWYY 0.875954

DYAA -0.817272

DYAC 0.0592668

DYAD -0.696921

DYAE -0.72582

DYAF -0.211676

DYAG -1.1318

DYAH -0.158569

DYAI -0.458229

DYAK -0.652847

DYAL -0.775255

DYAM 0.0144522

DYAN -0.555634

DYAP -0.810802

DYAQ -0.403802

DYAR -0.488707

DYAS -0.697039

DYAT -0.609532

DYAV -0.614806

DYAW 0.209176

DYAY -0.100564

DYCA 0.117318

DYCC 2.13087

DYCD -0.0293176

DYCE -0.0893953

DYCF 0.734897

DYCG -0.154155

DYCH 0.660561

DYCI 0.456359

DYCK 0.116082

DYCL 0.363085

DYCM 0.653047

DYCN 0.192898

DYCP 0.0740173

DYCQ 0.368422

DYCR 0.328435

DYCS 0.183489

DYCT 0.174993

DYCV 0.422798

DYCW 1.04119

DYCY 0.772768

DYDA -0.622301

DYDC -0.00600791

DYDD -0.419017

DYDE -0.465679

DYDF 0.451457

DYDG -0.7857

DYDH 0.197073

DYDI -0.351471

DYDK -0.263752

DYDL -0.656414

DYDM -0.119759

DYDN -0.112305

DYDP -0.523623

DYDQ -0.200108

DYDR -0.0458727

DYDS -0.30556

DYDT -0.294123

DYDV -0.453464

DYDW 0.301806

DYDY 4.67867

DYEA -0.721726

DYEC -0.0733445

DYED -0.574883

DYEE -0.528156

DYEF -0.175562

DYEG -1.02913

DYEH 0.0044663

DYEI -0.452492

DYEK -0.300686

DYEL -0.675497

DYEM -0.0988605

DYEN -0.335853

DYEP -0.662374

DYEQ -0.228958

DYER -0.15347

DYES -0.574303

DYET -0.474964

DYEV -0.59506

DYEW 0.276529

DYEY 0.144731

DYFA -0.234158

DYFC 0.751838

DYFD -0.0982151

DYFE -0.194603

DYFF 0.52504

DYFG -0.513029

DYFH 0.490029

DYFI 0.220518

DYFK -0.114

DYFL -0.0843096

DYFM 0.680491

DYFN 0.0158861

DYFP -0.0786457

DYFQ 0.160217

DYFR 0.0669565

DYFS -0.0616283

DYFT 0.00039625

DYFV 0.0675676

DYFW 0.918488

DYFY 0.549463

DYGA -1.12644

DYGC -0.266901

DYGD -0.76408

DYGE -0.903813

DYGF -0.44456

DYGG -1.87475

DYGH -0.458956

DYGI -0.719519

DYGK -0.765136

DYGL -0.947977

DYGM -0.410746

DYGN -0.630892

DYGP -0.963465

DYGQ -0.59277

DYGR -0.601419

DYGS -0.788148

DYGT -0.66236

DYGV -0.832039

DYGW -0.142121

DYGY -0.298883

DYHA -0.219736

DYHC 0.591455

DYHD 0.124431

DYHE 0.00178695

DYHF 0.485094

DYHG -0.474114

DYHH 0.632936

DYHI 0.132944

DYHK -0.135559

DYHL 0.0379336

DYHM 0.456319

DYHN 0.0915346

DYHP -0.0837305

DYHQ 0.0976872

DYHR 0.149401

DYHS 0.0233166

DYHT 0.113326

DYHV 0.0565009

DYHW 0.834106

DYHY 0.611262

DYIA -0.414321

DYIC 0.53815

DYID -0.413287

DYIE -0.44211

DYIF 0.235927

DYIG -0.780614

DYIH 0.196626

DYII 0.00379372

DYIK -0.365573

DYIL -0.357966

DYIM 0.467577

DYIN -0.245142

DYIP -0.347127

DYIQ -0.109753

DYIR -0.180177

DYIS -0.363379

DYIT -0.225362

DYIV -0.206141

DYIW 0.6474

DYIY 0.300303

DYKA -0.686877

DYKC 0.0535007

DYKD -0.302057

DYKE -0.31447

DYKF -0.11742

DYKG -0.935717

DYKH -0.141764

DYKI -0.388181

DYKK -0.42809

DYKL -0.61956

DYKM -0.113998

DYKN -0.305195

DYKP -0.582917

DYKQ -0.191921

DYKR -0.393181

DYKS -0.49139

DYKT -0.398528

DYKV -0.498325

DYKW 0.187047

DYKY 0.056236

DYLA -0.777921

DYLC 0.453029

DYLD -0.674306

DYLE -0.68793

DYLF -0.0733948

DYLG -1.00675

DYLH 0.0358179

DYLI -0.36433

DYLK -0.610721

DYLL -0.716492

DYLM 0.231183

DYLN -0.496499

DYLP -0.557921

DYLQ -0.270943

DYLR -0.41129

DYLS -0.64296

DYLT -0.50325

DYLV -0.552127

DYLW 0.507241

DYLY -0.0353985

DYMA -0.0157106

DYMC 0.726995

DYMD -0.0677884

DYME -0.0630119

DYMF 0.680864

DYMG -0.563264

DYMH 0.351857

DYMI 0.437081

DYMK -0.0318847

DYML 0.231839

DYMM 0.765659

DYMN -0.00286388

DYMP -0.179384

DYMQ 0.151161

DYMR 0.160088

DYMS -0.0842311

DYMT 0.0773828

DYMV 0.280769

DYMW 0.759583

DYMY 0.654684

DYNA -0.554069

DYNC 0.1938

DYND -0.236233

DYNE -0.337089

DYNF 0.0394747

DYNG -0.657568

DYNH 0.12882

DYNI -0.269685

DYNK -0.243152

DYNL -0.463743

DYNM 0.00432873

DYNN -0.00244045

DYNP -0.339171

DYNQ -0.0289993

DYNR -0.111518

DYNS -0.183711

DYNT -0.200827

DYNV -0.394664

DYNW 0.465825

DYNY 0.415047

DYPA -0.539162

DYPC 0.146401

DYPD -0.315568

DYPE -0.305787

DYPF 0.0565724

DYPG -0.691076

DYPH 0.0755589

DYPI -0.241989

DYPK -0.365109

DYPL -0.450803

DYPM 0.0664661

DYPN -0.21036

DYPP -0.364331

DYPQ -0.0895808

DYPR -0.152857

DYPS -0.305527

DYPT -0.233395

DYPV -0.303871

DYPW 0.508785

DYPY 0.177842

DYQA -0.381693

DYQC 0.353137

DYQD -0.243418

DYQE -0.230332

DYQF 0.193319

DYQG -0.622195

DYQH 0.092494

DYQI -0.142661

DYQK -0.168351

DYQL -0.295916

DYQM 0.106841

DYQN -0.0556316

DYQP -0.350652

DYQQ 0.162093

DYQR -0.0203135

DYQS -0.201344

DYQT -0.122082

DYQV -0.228439

DYQW 0.490345

DYQY 0.262056

DYRA -0.522313

DYRC 0.247994

DYRD -0.138384

DYRE -0.162427

DYRF 0.106545

DYRG -0.763914

DYRH 0.13325

DYRI -0.173728

DYRK -0.3956

DYRL -0.417905

DYRM 0.186187

DYRN -0.184142

DYRP -0.337576

DYRQ -0.0109379

DYRR -0.0849395

DYRS -0.29478

DYRT -0.248303

DYRV -0.316557

DYRW 0.441249

DYRY 0.236536

DYSA -0.696395

DYSC 0.230648

DYSD -0.401355

DYSE -0.4616

DYSF -0.0738897

DYSG -0.739448

DYSH 0.0341504

DYSI -0.381022

DYSK -0.42903

DYSL -0.611395

DYSM -0.0354125

DYSN -0.219668

DYSP -0.530064

DYSQ -0.128247

DYSR -0.254611

DYSS -0.315817

DYST -0.30007

DYSV -0.52825

DYSW 0.292571

DYSY 0.206803

DYTA -0.543043

DYTC 0.327801

DYTD -0.36226

DYTE -0.387132

DYTF 0.0397239

DYTG -0.735173

DYTH 0.111712

DYTI -0.252063

DYTK -0.363722

DYTL -0.511228

DYTM 0.110279

DYTN -0.188406

DYTP -0.398266

DYTQ -0.0715187

DYTR -0.192454

DYTS -0.251574

DYTT -0.243799

DYTV -0.340023

DYTW 0.403365

DYTY 0.256859

DYVA -0.611586

DYVC 0.461392

DYVD -0.550459

DYVE -0.558783

DYVF 0.047848

DYVG -0.905783

DYVH 0.0707679

DYVI -0.21174

DYVK -0.500366

DYVL -0.558599

DYVM 0.30484

DYVN -0.386863

DYVP -0.475043

DYVQ -0.235129

DYVR -0.33314

DYVS -0.504626

DYVT -0.405458

DYVV -0.370496

DYVW 0.533228

DYVY 0.209441

DYWA 0.154573

DYWC 1.04514

DYWD 0.216952

DYWE 0.189322

DYWF 0.946757

DYWG -0.0809009

DYWH 0.646157

DYWI 0.688246

DYWK 0.224649

DYWL 0.515958

DYWM 0.848956

DYWN 0.403738

DYWP 0.248038

DYWQ 0.506393

DYWR 0.49671

DYWS 0.226832

DYWT 0.321974

DYWV 0.560889

DYWW 1.19677

DYWY 1.03579

DYYA -0.204633

DYYC 0.946399

DYYD 0.026463

DYYE -0.100745

DYYF 0.537466

DYYG -0.350336

DYYH 0.50876

DYYI 0.223016

DYYK -0.0058167

DYYL -0.0581903

DYYM 0.617976

DYYN 0.172235

DYYP 0.0612121

DYYQ 0.267905

DYYR 0.204136

DYYS -0.0114374

DYYT 0.096621

DYYV 0.06973

DYYW 1.00018

DYYY 0.655489

EAAA -0.629242

EAAC -0.0618117

EAAD -0.837878

EAAE -0.849162

EAAF -0.344455

EAAG -1.26589

EAAH -0.275951

EAAI -0.590038

EAAK -0.711437

EAAL -0.906237

EAAM -0.122939

EAAN -0.664603

EAAP -0.947072

EAAQ -0.522453

EAAR -0.600508

EAAS -0.832778

EAAT -0.748272

EAAV -0.762748

EAAW 0.0783503

EAAY -0.288575

EACA 0.00155234

EACC 1.99696

EACD -0.139428

EACE -0.201679

EACF 0.613659

EACG -0.266105

EACH 0.556067

EACI 0.333923

EACK 0.00144196

EACL 0.229255

EACM 0.552181

EACN 0.0824895

EACP -0.032583

EACQ 0.259705

EACR 0.214056

EACS 0.0632203

EACT 0.0619385

EACV 0.294213

EACW 0.938723

EACY 0.656205

EADA -0.370116

EADC -0.120112

EADD -0.58844

EADE -0.601053

EADF -0.267692

EADG -0.907909

EADH -0.0301397

EADI -0.548142

EADK -0.279232

EADL -0.763066

EADM -0.236742

EADN -0.295925

EADP -0.664682

EADQ -0.302459

EADR -0.214966

EADS -0.485461

EADT -0.442306

EADV -0.631017

EADW 0.140445

EADY -0.0836363

EAEA 4.0914

EAEC -0.153732

EAED -0.658059

EAEE -0.409639

EAEF -0.29801

EAEG -1.07549

EAEH -0.0928285

EAEI -0.518338

EAEK -0.309945

EAEL -0.661527

EAEM -0.175387

EAEN -0.181629

EAEP -0.628549

EAEQ -0.0987608

EAER -0.166065

EAES -0.306914

EAET -0.310812

EAEV -0.41721

EAEW 0.147575

EAEY -0.164619

EAFA -0.333142

EAFC 0.630294

EAFD -0.237772

EAFE -0.32474

EAFF 0.379022

EAFG -0.636957

EAFH 0.366692

EAFI 0.0727985

EAFK -0.255416

EAFL -0.238605

EAFM 0.557862

EAFN -0.11739

EAFP -0.212286

EAFQ 0.0339844

EAFR -0.0673261

EAFS -0.211384

EAFT -0.127938

EAFV -0.113132

EAFW 0.790807

EAFY 0.38686

EAGA -1.2126

EAGC -0.373264

EAGD -0.897335

EAGE -1.02144

EAGF -0.576336

EAGG -1.98292

EAGH -0.583609

EAGI -0.871692

EAGK -0.891909

EAGL -1.07751

EAGM -0.511166

EAGN -0.766377

EAGP -1.07973

EAGQ -0.729574

EAGR -0.73054

EAGS -0.930199

EAGT -0.806298

EAGV -0.963968

EAGW -0.251667

EAGY -0.449592

EAHA -0.283917

EAHC 0.487158

EAHD -0.00394082

EAHE -0.119749

EAHF 0.364348

EAHG -0.584768

EAHH 0.523857

EAHI 0.0292678

EAHK -0.241666

EAHL -0.104423

EAHM 0.349387

EAHN -0.0260706

EAHP -0.203974

EAHQ 0.00472498

EAHR 0.0353134

EAHS -0.0843413

EAHT -0.0149462

EAHV -0.0616374

EAHW 0.728979

EAHY 0.425957

EAIA -0.546157

EAIC 0.411674

EAID -0.55755

EAIE -0.569691

EAIF 0.0702164

EAIG -0.923113

EAIH 0.0541086

EAII -0.14614

EAIK -0.503627

EAIL -0.503364

EAIM 0.330459

EAIN -0.374454

EAIP -0.487056

EAIQ -0.240151

EAIR -0.322282

EAIS -0.5077

EAIT -0.360587

EAIV -0.358284

EAIW 0.523068

EAIY 0.134382

EAKA -0.679328

EAKC -0.0540528

EAKD -0.449593

EAKE -0.389824

EAKF -0.233443

EAKG -1.07125

EAKH -0.244509

EAKI -0.495469

EAKK -0.559291

EAKL -0.772353

EAKM -0.237396

EAKN -0.398222

EAKP -0.723309

EAKQ -0.291625

EAKR -0.526423

EAKS -0.577218

EAKT -0.50101

EAKV -0.618519

EAKW 0.0611148

EAKY -0.130592

EALA -0.843968

EALC 0.318084

EALD -0.814814

EALE -0.834053

EALF -0.228853

EALG -1.14011

EALH -0.0957692

EALI -0.518234

EALK -0.769638

EALL -0.902164

EALM 0.0988839

EALN -0.647972

EALP -0.69517

EALQ -0.416396

EALR -0.539148

EALS -0.801651

EALT -0.692452

EALV -0.722562

EALW 0.369714

EALY -0.171555

EAMA -0.120784

EAMC 0.62297

EAMD -0.177282

EAME -0.189446

EAMF 0.551562

EAMG -0.670787

EAMH 0.247399

EAMI 0.313902

EAMK -0.155455

EAML 0.091604

EAMM 0.649707

EAMN -0.118203

EAMP -0.291317

EAMQ 0.0379672

EAMR 0.0383475

EAMS -0.203653

EAMT -0.0321987

EAMV 0.144082

EAMW 0.655739

EAMY 0.518039

EANA -0.602241

EANC 0.0933318

EAND -0.36824

EANE -0.464678

EANF -0.108919

EANG -0.803516

EANH 0.00424099

EANI -0.399873

EANK -0.375185

EANL -0.623335

EANM -0.112594

EANN -0.140397

EANP -0.462362

EANQ -0.140192

EANR -0.245814

EANS -0.372347

EANT -0.33194

EANV -0.513118

EANW 0.344297

EANY 0.0811989

EAPA -0.650865

EAPC 0.0430071

EAPD -0.44698

EAPE -0.439595

EAPF -0.075151

EAPG -0.825451

EAPH -0.0420644

EAPI -0.397771

EAPK -0.488722

EAPL -0.559947

EAPM -0.0529702

EAPN -0.333326

EAPP -0.491354

EAPQ -0.216438

EAPR -0.292077

EAPS -0.413575

EAPT -0.386026

EAPV -0.448277

EAPW 0.391768

EAPY 0.0305352

EAQA -0.203106

EAQC 0.236568

EAQD -0.353968

EAQE -0.335571

EAQF 0.0451868

EAQG -0.750864

EAQH -0.0240545

EAQI -0.265483

EAQK -0.312606

EAQL -0.43795

EAQM -0.0066843

EAQN -0.167329

EAQP -0.368284

EAQQ 0.0677519

EAQR -0.14762

EAQS -0.338504

EAQT -0.244911

EAQV -0.341763

EAQW 0.379748

EAQY 0.146085

EARA -0.51613

EARC 0.142473

EARD -0.279553

EARE -0.27905

EARF -0.0505805

EARG -0.895865

EARH 0.00888896

EARI -0.320577

EARK -0.510426

EARL -0.560012

EARM 0.0661309

EARN -0.326952

EARP -0.459469

EARQ -0.14856

EARR -0.236444

EARS -0.370594

EART -0.411336

EARV -0.459604

EARW 0.328766

EARY 0.0684252

EASA -0.663666

EASC 0.114097

EASD -0.546831

EASE -0.60212

EASF -0.168038

EASG -0.873378

EASH -0.0893595

EASI -0.514379

EASK -0.589955

EASL -0.765493

EASM -0.160869

EASN -0.367066

EASP -0.669005

EASQ -0.275638

EASR -0.410701

EASS -0.514832

EAST -0.458973

EASV -0.639254

EASW 0.184677

EASY -0.121937

EATA -0.541344

EATC 0.211343

EATD -0.504156

EATE -0.522583

EATF -0.0970459

EATG -0.854482

EATH -0.0260565

EATI -0.372274

EATK -0.505625

EATL -0.691827

EATM -0.00398374

EATN -0.311766

EATP -0.544259

EATQ -0.214287

EATR -0.333215

EATS -0.358377

EATT -0.304606

EATV -0.54881

EATW 0.28493

EATY -0.0276742

EAVA -0.629057

EAVC 0.330194

EAVD -0.68003

EAVE -0.695113

EAVF -0.0953095

EAVG -1.02468

EAVH -0.057878

EAVI -0.368724

EAVK -0.645558

EAVL -0.704276

EAVM 0.158981

EAVN -0.537894

EAVP -0.611037

EAVQ -0.36496

EAVR -0.469063

EAVS -0.667897

EAVT -0.532632

EAVV -0.533672

EAVW 0.395639

EAVY -0.0711949

EAWA 0.0364292

EAWC 0.942647

EAWD 0.0999944

EAWE 0.0700052

EAWF 0.818505

EAWG -0.196734

EAWH 0.543202

EAWI 0.55903

EAWK 0.108906

EAWL 0.377893

EAWM 0.743048

EAWN 0.283359

EAWP 0.136652

EAWQ 0.394967

EAWR 0.377933

EAWS 0.112402

EAWT 0.193611

EAWV 0.428491

EAWW 1.09172

EAWY 0.910307

EAYA -0.311807

EAYC 0.823673

EAYD -0.115857

EAYE -0.239432

EAYF 0.399751

EAYG -0.48925

EAYH 0.381725

EAYI 0.0786459

EAYK -0.141176

EAYL -0.18195

EAYM 0.489418

EAYN 0.0218666

EAYP -0.0610065

EAYQ 0.129066

EAYR 0.0609941

EAYS -0.162799

EAYT -0.0577083

EAYV -0.0899518

EAYW 0.865035

EAYY 0.471235

ECAA -0.476347

ECAC 0.243804

ECAD -0.438554

ECAE -0.418034

ECAF 0.109524

ECAG -0.883965

ECAH 0.0551722

ECAI -0.129786

ECAK -0.35693

ECAL -0.415359

ECAM 0.242558

ECAN -0.294365

ECAP -0.591301

ECAQ -0.147691

ECAR -0.198651

ECAS -0.409751

ECAT -0.311547

ECAV -0.283251

ECAW 0.397887

ECAY 0.146122

ECCA 0.296369

ECCC 2.37207

ECCD 0.0758307

ECCE 0.0192339

ECCF 0.895574

ECCG -0.0355275

ECCH 0.738512

ECCI 0.629255

ECCK 0.242476

ECCL 0.60237

ECCM 0.72637

ECCN 0.288654

ECCP 0.162689

ECCQ 0.467701

ECCR 0.456928

ECCS 0.319383

ECCT 0.301671

ECCV 0.631079

ECCW 1.11317

ECCY 0.919856

ECDA -0.355083

ECDC 0.112216

ECDD -0.173601

ECDE -0.205999

ECDF 0.128496

ECDG -0.547294

ECDH 0.267657

ECDI -0.140929

ECDK 0.0551035

ECDL -0.34721

ECDM 0.0202816

ECDN 0.0922797

ECDP -0.325026

ECDQ 0.0306141

ECDR 0.203746

ECDS -0.0914669

ECDT -0.0368805

ECDV -0.232635

ECDW 0.402831

ECDY 0.322255

ECEA -0.396222

ECEC 2.30778

ECED -0.309121

ECEE -0.238292

ECEF 0.0656848

ECEG -0.812888

ECEH 0.184822

ECEI -0.161295

ECEK 0.0224349

ECEL -0.351485

ECEM 0.0635035

ECEN -0.0968392

ECEP -0.463789

ECEQ 0.0207064

ECER 0.150271

ECES -0.284244

ECET -0.188274

ECEV -0.259657

ECEW 0.427903

ECEY 0.193009

ECFA 0.0742106

ECFC 0.918371

ECFD 0.160172

ECFE 0.0719905

ECFF 0.838

ECFG -0.283419

ECFH 0.684703

ECFI 0.541928

ECFK 0.148025

ECFL 0.262609

ECFM 0.900879

ECFN 0.244565

ECFP 0.139804

ECFQ 0.369508

ECFR 0.324283

ECFS 0.196337

ECFT 0.278757

ECFV 0.371181

ECFW 1.10442

ECFY 0.816818

ECGA -0.875297

ECGC -0.163429

ECGD -0.521088

ECGE -0.659365

ECGF -0.193861

ECGG -1.77141

ECGH -0.330284

ECGI -0.475819

ECGK -0.503205

ECGL -0.648484

ECGM -0.275799

ECGN -0.409639

ECGP -0.792304

ECGQ -0.397306

ECGR -0.349705

ECGS -0.530981

ECGT -0.397997

ECGV -0.551162

ECGW -0.0249207

ECGY -0.0807958

ECHA -0.0266745

ECHC 0.665588

ECHD 0.325287

ECHE 0.194479

ECHF 0.683653

ECHG -0.339382

ECHH 0.751328

ECHI 0.330752

ECHK 0.0468354

ECHL 0.293327

ECHM 0.551195

ECHN 0.237556

ECHP 0.0373366

ECHQ 0.21842

ECHR 0.324181

ECHS 0.209285

ECHT 0.280889

ECHV 0.28221

ECHW 0.930176

ECHY 0.737228

ECIA -0.126043

ECIC 0.753762

ECID -0.144181

ECIE -0.159487

ECIF 0.553974

ECIG -0.539713

ECIH 0.386727

ECII 0.346611

ECIK -0.0864553

ECIL 0.00137019

ECIM 0.715013

ECIN 0.0058136

ECIP -0.120882

ECIQ 0.132863

ECIR 0.100901

ECIS -0.0785525

ECIT 0.0807397

ECIV 0.148074

ECIW 0.852148

ECIY 0.587636

ECKA -0.396334

ECKC 0.183546

ECKD 0.00203586

ECKE 0.0112002

ECKF 0.130641

ECKG -0.712007

ECKH 0.00019884

ECKI -0.098578

ECKK -0.144667

ECKL -0.32124

ECKM 0.0361018

ECKN -0.0620174

ECKP -0.385157

ECKQ 0.042057

ECKR -0.150406

ECKS -0.216157

ECKT -0.121585

ECKV -0.213901

ECKW 0.310596

ECKY 0.277078

ECLA -0.423875

ECLC 0.718756

ECLD -0.37208

ECLE -0.364771

ECLF 0.272073

ECLG -0.723273

ECLH 0.296039

ECLI -0.00942016

ECLK -0.294576

ECLL -0.342176

ECLM 0.519827

ECLN -0.211347

ECLP -0.277591

ECLQ 0.0244935

ECLR -0.0822849

ECLS -0.32345

ECLT -0.198584

ECLV -0.200526

ECLW 0.76894

ECLY 0.311938

ECMA 0.211691

ECMC 0.806497

ECMD 0.0871608

ECME 0.108713

ECMF 0.902266

ECMG -0.448261

ECMH 0.438992

ECMI 0.684189

ECMK 0.13012

ECML 0.515306

ECMM 0.895707

ECMN 0.116292

ECMP -0.0761998

ECMQ 0.270487

ECMR 0.321407

ECMS 0.0729077

ECMT 0.255727

ECMV 0.538463

ECMW 0.840537

ECMY 0.831874

ECNA -0.286992

ECNC 0.297987

ECND 0.0276363

ECNE -0.0866759

ECNF 0.258121

ECNG -0.44856

ECNH 0.258643

ECNI -0.0327926

ECNK 0.0207167

ECNL -0.1951

ECNM 0.12924

ECNN 0.243335

ECNP -0.152253

ECNQ 0.18382

ECNR 0.123494

ECNS 0.00358605

ECNT 0.0581391

ECNV -0.119872

ECNW 0.591958

ECNY 0.435731

ECPA -0.268252

ECPC 0.1753

ECPD -0.0645876

ECPE -0.0340614

ECPF 0.309853

ECPG -0.472388

ECPH 0.222009

ECPI -0.00691819

ECPK -0.123389

ECPL -0.149181

ECPM 0.199263

ECPN 0.00695348

ECPP -0.171862

ECPQ 0.115356

ECPR 0.0916553

ECPS -0.0476213

ECPT 0.00978565

ECPV -0.0123899

ECPW 0.651743

ECPY 0.401507

ECQA -0.126247

ECQC 0.594567

ECQD -0.0202043

ECQE 0.00537467

ECQF 0.41074

ECQG -0.426749

ECQH 0.204974

ECQI 0.0997229

ECQK 0.0754561

ECQL -0.00543046

ECQM 0.220212

ECQN 0.153532

ECQP -0.190183

ECQQ 0.376223

ECQR 0.209875

ECQS 0.0361102

ECQT 0.119827

ECQV 0.0194826

ECQW 0.603589

ECQY 0.515662

ECRA -0.232149

ECRC 0.362854

ECRD 0.159988

ECRE 0.148019

ECRF 0.365924

ECRG -0.548849

ECRH 0.301907

ECRI 0.104893

ECRK -0.145889

ECRL -0.104272

ECRM 0.352732

ECRN 0.0486872

ECRP -0.129064

ECRQ 0.220401

ECRR 0.172334

ECRS -0.0279121

ECRT 0.0006218

ECRV -0.0213711

ECRW 0.584538

ECRY 0.467205

ECSA -0.413161

ECSC 0.372186

ECSD -0.127569

ECSE -0.170115

ECSF 0.205077

ECSG -0.479923

ECSH 0.223501

ECSI -0.111705

ECSK -0.141091

ECSL -0.292995

ECSM 0.128242

ECSN 0.0226166

ECSP -0.321152

ECSQ 0.123486

ECSR 0.0256176

ECSS -0.0271173

ECST 0.0015378

ECSV -0.212137

ECSW 0.445505

ECSY 0.298255

ECTA -0.306469

ECTC 0.523748

ECTD -0.0771999

ECTE -0.0934114

ECTF 0.31138

ECTG -0.476086

ECTH 0.283406

ECTI 0.0504768

ECTK -0.0921195

ECTL -0.189179

ECTM 0.290563

ECTN 0.0758021

ECTP -0.183691

ECTQ 0.182779

ECTR 0.0779591

ECTS -0.0301332

ECTT 0.0766692

ECTV -0.0545332

ECTW 0.564842

ECTY 0.403242

ECVA -0.277678

ECVC 0.658174

ECVD -0.259636

ECVE -0.274455

ECVF 0.389195

ECVG -0.642319

ECVH 0.288383

ECVI 0.146143

ECVK -0.19761

ECVL -0.193031

ECVM 0.559181

ECVN -0.1234

ECVP -0.221284

ECVQ 0.0277596

ECVR -0.0404732

ECVS -0.209594

ECVT -0.0655696

ECVV -0.0347462

ECVW 0.752502

ECVY 0.397339

ECWA 0.337759

ECWC 1.11741

ECWD 0.350906

ECWE 0.335708

ECWF 1.13872

ECWG 0.0436919

ECWH 0.72173

ECWI 0.898469

ECWK 0.363429

ECWL 0.779146

ECWM 0.938571

ECWN 0.521674

ECWP 0.351596

ECWQ 0.61953

ECWR 0.646973

ECWS 0.368423

ECWT 0.456784

ECWV 0.789198

ECWW 1.28044

ECWY 1.22679

ECYA 0.084621

ECYC 1.1236

ECYD 0.286533

ECYE 0.163255

ECYF 0.837

ECYG -0.116593

ECYH 0.686477

ECYI 0.524979

ECYK 0.261039

ECYL 0.278415

ECYM 0.804885

ECYN 0.411496

ECYP 0.28849

ECYQ 0.499335

ECYR 0.470078

ECYS 0.237282

ECYT 0.347272

ECYV 0.371606

ECYW 1.17538

ECYY 0.907646

EDAA -0.765308

EDAC 0.0816553

EDAD -0.51494

EDAE -0.693489

EDAF -0.172438

EDAG -1.10508

EDAH -0.130669

EDAI -0.425396

EDAK -0.633356

EDAL -0.734153

EDAM 0.0364807

EDAN -0.535589

EDAP -0.785172

EDAQ -0.375245

EDAR -0.466517

EDAS -0.676705

EDAT -0.584533

EDAV -0.595545

EDAW 0.229828

EDAY -0.125781

EDCA 0.140064

EDCC 2.15603

EDCD -0.00937438

EDCE -0.0714543

EDCF 0.758981

EDCG -0.134221

EDCH 0.678298

EDCI 0.47924

EDCK 0.136379

EDCL 0.388285

EDCM 0.670568

EDCN 0.209361

EDCP 0.0929749

EDCQ 0.387724

EDCR 0.349227

EDCS 0.202869

EDCT 0.19541

EDCV 0.447069

EDCW 1.05873

EDCY 0.791641

EDDA -0.616293

EDDC 0.00653243

EDDD 0.00387192

EDDE -0.441261

EDDF -0.103408

EDDG -0.771641

EDDH 0.106804

EDDI -0.38344

EDDK -0.235976

EDDL -0.629369

EDDM -0.103566

EDDN -0.171982

EDDP -0.510106

EDDQ -0.179402

EDDR -0.0730338

EDDS -0.351976

EDDT -0.300348

EDDV -0.50242

EDDW 0.280089

EDDY 0.073297

EDEA -0.647968

EDEC -0.0565407

EDED 4.00724

EDEE -0.279303

EDEF -0.164296

EDEG -1.00407

EDEH 0.028085

EDEI -0.419072

EDEK -0.210797

EDEL -0.649432

EDEM -0.0729077

EDEN -0.184627

EDEP -0.629889

EDEQ -0.171991

EDER -0.129693

EDES -0.498007

EDET -0.426801

EDEV -0.545568

EDEW 0.290002

EDEY -0.0412846

EDFA -0.202269

EDFC 0.773437

EDFD -0.0767727

EDFE -0.170232

EDFF 0.555123

EDFG -0.486353

EDFH 0.514516

EDFI 0.251699

EDFK -0.0860641

EDFL -0.0543084

EDFM 0.705821

EDFN 0.035352

EDFP -0.0541925

EDFQ 0.170452

EDFR 0.092361

EDFS -0.0488367

EDFT 0.0317249

EDFV 0.0733802

EDFW 0.939104

EDFY 0.550427

EDGA -1.09977

EDGC -0.246786

EDGD -0.657534

EDGE -0.872762

EDGF -0.40931

EDGG -1.85573

EDGH -0.446015

EDGI -0.704294

EDGK -0.737972

EDGL -0.922305

EDGM -0.389598

EDGN -0.591295

EDGP -0.936422

EDGQ -0.576634

EDGR -0.569741

EDGS -0.757303

EDGT -0.638705

EDGV -0.801492

EDGW -0.122452

EDGY -0.303807

EDHA -0.192344

EDHC 0.609084

EDHD 0.157924

EDHE 0.0331481

EDHF 0.512601

EDHG -0.453641

EDHH 0.647099

EDHI 0.161579

EDHK -0.114743

EDHL 0.0610096

EDHM 0.475056

EDHN 0.115357

EDHP -0.0625927

EDHQ 0.118912

EDHR 0.178417

EDHS 0.0498798

EDHT 0.120013

EDHV 0.0952678

EDHW 0.854961

EDHY 0.571982

EDIA -0.420804

EDIC 0.561349

EDID -0.377199

EDIE -0.409692

EDIF 0.261464

EDIG -0.752257

EDIH 0.206345

EDII 0.0339799

EDIK -0.342508

EDIL -0.327611

EDIM 0.494619

EDIN -0.214802

EDIP -0.327077

EDIQ -0.081861

EDIR -0.150983

EDIS -0.333931

EDIT -0.194532

EDIV -0.171666

EDIW 0.672516

EDIY 0.305992

EDKA -0.670571

EDKC 0.0731659

EDKD -0.131371

EDKE -0.262045

EDKF -0.102048

EDKG -0.91156

EDKH -0.126984

EDKI -0.355973

EDKK -0.394817

EDKL -0.609205

EDKM -0.083137

EDKN -0.275201

EDKP -0.559228

EDKQ -0.166818

EDKR -0.370806

EDKS -0.453834

EDKT -0.373707

EDKV -0.479749

EDKW 0.201363

EDKY 0.0348175

EDLA -0.744053

EDLC 0.479754

EDLD -0.647744

EDLE -0.652117

EDLF -0.0470114

EDLG -0.980998

EDLH 0.0628579

EDLI -0.335604

EDLK -0.585656

EDLL -0.687403

EDLM 0.262572

EDLN -0.468792

EDLP -0.534661

EDLQ -0.244537

EDLR -0.371879

EDLS -0.620957

EDLT -0.499792

EDLV -0.528673

EDLW 0.532895

EDLY 0.00523138

EDMA 0.00962234

EDMC 0.745369

EDMD -0.0378668

EDME -0.0428574

EDMF 0.706733

EDMG -0.543542

EDMH 0.372141

EDMI 0.463479

EDMK -0.00801802

EDML 0.256571

EDMM 0.788649

EDMN 0.0140104

EDMP -0.159946

EDMQ 0.17084

EDMR 0.182009

EDMS -0.0543101

EDMT 0.104539

EDMV 0.308756

EDMW 0.778528

EDMY 0.663028

EDNA -0.525066

EDNC 0.213953

EDND -0.170387

EDNE -0.309947

EDNF 0.0474994

EDNG -0.643992

EDNH 0.132768

EDNI -0.249803

EDNK -0.214178

EDNL -0.455195

EDNM 0.0247946

EDNN 0.0140338

EDNP -0.318473

EDNQ -0.00953007

EDNR -0.102025

EDNS -0.230283

EDNT -0.177684

EDNV -0.362379

EDNW 0.483789

EDNY 0.220098

EDPA -0.517849

EDPC 0.165446

EDPD -0.266172

EDPE -0.278965

EDPF 0.0873899

EDPG -0.668542

EDPH 0.0965579

EDPI -0.234401

EDPK -0.34111

EDPL -0.423567

EDPM 0.084152

EDPN -0.177873

EDPP -0.339281

EDPQ -0.0671308

EDPR -0.127736

EDPS -0.279506

EDPT -0.221303

EDPV -0.275067

EDPW 0.530139

EDPY 0.186277

EDQA -0.357445

EDQC 0.372153

EDQD 0.0899403

EDQE -0.18819

EDQF 0.204055

EDQG -0.599859

EDQH 0.103982

EDQI -0.116269

EDQK -0.144594

EDQL -0.264554

EDQM 0.125283

EDQN -0.0420094

EDQP -0.328718

EDQQ 0.186038

EDQR 0.00175452

EDQS -0.175488

EDQT -0.10258

EDQV -0.210735

EDQW 0.512027

EDQY 0.308138

EDRA -0.491398

EDRC 0.268783

EDRD -0.0593252

EDRE -0.126163

EDRF 0.127384

EDRG -0.739609

EDRH 0.159999

EDRI -0.147245

EDRK -0.355367

EDRL -0.394652

EDRM 0.209474

EDRN -0.161427

EDRP -0.311515

EDRQ 0.00805449

EDRR -0.0684719

EDRS -0.274486

EDRT -0.228762

EDRV -0.288333

EDRW 0.46325

EDRY 0.231153

EDSA -0.676543

EDSC 0.253968

EDSD -0.301735

EDSE -0.437173

EDSF -0.0450335

EDSG -0.717432

EDSH 0.059716

EDSI -0.36827

EDSK -0.379894

EDSL -0.581296

EDSM -0.0142162

EDSN -0.213813

EDSP -0.502612

EDSQ -0.102348

EDSR -0.219819

EDSS -0.305035

EDST -0.272241

EDSV -0.481344

EDSW 0.313611

EDSY 0.0606995

EDTA -0.577219

EDTC 0.34655

EDTD -0.117208

EDTE -0.368728

EDTF 0.0549898

EDTG -0.700397

EDTH 0.117841

EDTI -0.21683

EDTK -0.346049

EDTL -0.488392

EDTM 0.135508

EDTN -0.161061

EDTP -0.383719

EDTQ -0.0468013

EDTR -0.170644

EDTS -0.304755

EDTT -0.196763

EDTV -0.339998

EDTW 0.428859

EDTY 0.150198

EDVA -0.590689

EDVC 0.481376

EDVD -0.505109

EDVE -0.533944

EDVF 0.0892165

EDVG -0.878211

EDVH 0.0920835

EDVI -0.177934

EDVK -0.467233

EDVL -0.527622

EDVM 0.326552

EDVN -0.364306

EDVP -0.44734

EDVQ -0.21124

EDVR -0.308949

EDVS -0.475934

EDVT -0.353185

EDVV -0.341301

EDVW 0.552858

EDVY 0.111163

EDWA 0.179417

EDWC 1.0627

EDWD 0.236504

EDWE 0.210145

EDWF 0.967441

EDWG -0.0616338

EDWH 0.664443

EDWI 0.712111

EDWK 0.247713

EDWL 0.541611

EDWM 0.868086

EDWN 0.420602

EDWP 0.265942

EDWQ 0.525941

EDWR 0.51805

EDWS 0.249071

EDWT 0.335829

EDWV 0.584428

EDWW 1.215

EDWY 1.05842

EDYA -0.177834

EDYC 0.969312

EDYD 0.0497141

EDYE -0.0642838

EDYF 0.569411

EDYG -0.329907

EDYH 0.531597

EDYI 0.254275

EDYK 0.0239384

EDYL -0.0243587

EDYM 0.640017

EDYN 0.197402

EDYP 0.0859771

EDYQ 0.297548

EDYR 0.229883

EDYS 0.00176716

EDYT 0.103853

EDYV 0.0890405

EDYW 1.01472

EDYY 0.64318

EEAA -0.894494

EEAC -0.0227602

EEAD -0.795167

EEAE -0.487087

EEAF -0.305274

EEAG -1.21858

EEAH -0.246525

EEAI -0.56904

EEAK -0.709164

EEAL -0.886278

EEAM -0.0759647

EEAN -0.621808

EEAP -0.874629

EEAQ -0.492904

EEAR -0.587083

EEAS -0.783563

EEAT -0.706088

EEAV -0.737307

EEAW 0.111498

EEAY -0.241754

EECA 0.0325911

EECC 2.04106

EECD -0.10536

EECE -0.151213

EECF 0.652414

EECG -0.231386

EECH 0.592182

EECI 0.372438

EECK 0.0403829

EECL 0.271043

EECM 0.583114

EECN 0.115958

EECP 0.00149846

EECQ 0.294871

EECR 0.25081

EECS 0.10313

EECT 0.095413

EECV 0.333199

EECW 0.971618

EECY 0.689287

EEDA -0.69321

EEDC -0.0891769

EEDD -0.410091

EEDE -0.114933

EEDF -0.233171

EEDG -0.889462

EEDH 0.00364327

EEDI -0.498652

EEDK -0.283203

EEDL -0.765712

EEDM -0.197945

EEDN -0.271897

EEDP -0.614315

EEDQ -0.248473

EEDR -0.207806

EEDS -0.434852

EEDT -0.448217

EEDV -0.625184

EEDW 0.178505

EEDY -0.0467272

EEEA -0.566299

EEEC -0.152462

EEED -0.446053

EEEE 4.23604

EEEF -0.273995

EEEG -1.0664

EEEH -0.0596526

EEEI -0.540422

EEEK -0.118316

EEEL -0.742838

EEEM -0.165506

EEEN -0.390388

EEEP -0.730261

EEEQ -0.0465267

EEER -0.146467

EEES -0.625705

EEET -0.486795

EEEV -0.605684

EEEW 0.186481

EEEY -0.163023

EEFA -0.328052

EEFC 0.668375

EEFD -0.195877

EEFE -0.281389

EEFF 0.422012

EEFG -0.606162

EEFH 0.407367

EEFI 0.11817

EEFK -0.202977

EEFL -0.169549

EEFM 0.591173

EEFN -0.069371

EEFP -0.167053

EEFQ 0.0621431

EEFR -0.0276523

EEFS -0.174934

EEFT -0.0940118

EEFV -0.0623596

EEFW 0.828845

EEFY 0.425237

EEGA -1.21388

EEGC -0.341864

EEGD -0.882235

EEGE -0.879761

EEGF -0.518733

EEGG -1.94955

EEGH -0.539257

EEGI -0.825351

EEGK -0.83594

EEGL -1.04479

EEGM -0.4911

EEGN -0.731292

EEGP -1.04183

EEGQ -0.692943

EEGR -0.704332

EEGS -0.88325

EEGT -0.76254

EEGV -0.921495

EEGW -0.219926

EEGY -0.422092

EEHA -0.312046

EEHC 0.521657

EEHD 0.0666106

EEHE -0.044205

EEHF 0.398783

EEHG -0.551304

EEHH 0.557106

EEHI 0.0626864

EEHK -0.217797

EEHL -0.0588386

EEHM 0.381253

EEHN 0.015975

EEHP -0.165179

EEHQ 0.0254469

EEHR 0.0999444

EEHS -0.0556085

EEHT 0.014106

EEHV -0.020545

EEHW 0.762817

EEHY 0.502495

EEIA -0.557542

EEIC 0.45246

EEID -0.506916

EEIE -0.522589

EEIF 0.127815

EEIG -0.867796

EEIH 0.0950084

EEII -0.108047

EEIK -0.465373

EEIL -0.460337

EEIM 0.370676

EEIN -0.329114

EEIP -0.444443

EEIQ -0.207554

EEIR -0.280418

EEIS -0.459651

EEIT -0.31744

EEIV -0.30447

EEIW 0.561594

EEIY 0.176418

EEKA -0.795134

EEKC -0.0188413

EEKD -0.351956

EEKE -0.0476487

EEKF -0.201712

EEKG -1.02656

EEKH -0.235054

EEKI -0.472457

EEKK -0.553522

EEKL -0.740017

EEKM -0.186821

EEKN -0.385778

EEKP -0.666806

EEKQ -0.269964

EEKR -0.483347

EEKS -0.578484

EEKT -0.466191

EEKV -0.625711

EEKW 0.0989752

EEKY -0.0843227

EELA -0.86398

EELC 0.359712

EELD -0.760312

EELE -0.766283

EELF -0.183437

EELG -1.10511

EELH -0.0600765

EELI -0.477467

EELK -0.720228

EELL -0.83232

EELM 0.137128

EELN -0.582283

EELP -0.64988

EELQ -0.367149

EELR -0.522695

EELS -0.743939

EELT -0.655055

EELV -0.675418

EELW 0.412906

EELY -0.117814

EEMA -0.0941622

EEMC 0.656587

EEMD -0.153113

EEME -0.135796

EEMF 0.591798

EEMG -0.639233

EEMH 0.280858

EEMI 0.344307

EEMK -0.108904

EEML 0.134991

EEMM 0.686934

EEMN -0.0857205

EEMP -0.253494

EEMQ 0.072659

EEMR 0.0773251

EEMS -0.165934

EEMT -0.00164914

EEMV 0.18996

EEMW 0.689213

EEMY 0.551729

EENA -0.62755

EENC 0.120549

EEND -0.316665

EENE -0.36146

EENF -0.066397

EENG -0.757628

EENH 0.0263615

EENI -0.374489

EENK -0.329021

EENL -0.581784

EENM -0.0687971

EENN -0.0999155

EENP -0.428022

EENQ -0.112718

EENR -0.212772

EENS -0.341622

EENT -0.298868

EENV -0.407306

EENW 0.390246

EENY 0.129526

EEPA -0.628799

EEPC 0.0727909

EEPD -0.40024

EEPE -0.350945

EEPF -0.0271606

EEPG -0.768503

EEPH -0.00564742

EEPI -0.353702

EEPK -0.452584

EEPL -0.552903

EEPM -0.0161402

EEPN -0.297217

EEPP -0.444841

EEPQ -0.184034

EEPR -0.238828

EEPS -0.385768

EEPT -0.345636

EEPV -0.394888

EEPW 0.428797

EEPY 0.0601649

EEQA -0.47088

EEQC 0.279439

EEQD -0.25329

EEQE 0.392221

EEQF 0.0914166

EEQG -0.713619

EEQH 0.0176411

EEQI -0.23334

EEQK -0.28279

EEQL -0.401578

EEQM 0.0304976

EEQN -0.125004

EEQP -0.411994

EEQQ 0.0657487

EEQR -0.131761

EEQS -0.261359

EEQT -0.216793

EEQV -0.306933

EEQW 0.415586

EEQY 0.190098

EERA -0.599521

EERC 0.169649

EERD -0.243329

EERE 0.0129328

EERF 0.0111089

EERG -0.859257

EERH 0.0484178

EERI -0.274923

EERK -0.465976

EERL -0.52394

EERM 0.10671

EERN -0.268471

EERP -0.424747

EERQ -0.126594

EERR -0.181076

EERS -0.388336

EERT -0.37039

EERV -0.414438

EERW 0.362048

EERY 0.101965

EESA -0.790762

EESC 0.151087

EESD -0.517871

EESE -0.436538

EESF -0.170572

EESG -0.839216

EESH -0.0503747

EESI -0.451464

EESK -0.485973

EESL -0.740379

EESM -0.107346

EESN -0.329148

EESP -0.625231

EESQ -0.227248

EESR -0.369354

EESS -0.428441

EEST -0.406235

EESV -0.58737

EESW 0.210766

EESY -0.0760183

EETA -0.689056

EETC 0.244962

EETD -0.448843

EETE -0.0242314

EETF -0.0588675

EETG -0.824911

EETH 0.106372

EETI -0.343926

EETK -0.469974

EETL -0.63438

EETM 0.0342324

EETN -0.275486

EETP -0.501047

EETQ -0.165071

EETR -0.280092

EETS -0.399673

EETT -0.322844

EETV -0.479957

EETW 0.327581

EETY 0.0271859

EEVA -0.742219

EEVC 0.36777

EEVD -0.64488

EEVE -0.545434

EEVF -0.0456712

EEVG -1.00957

EEVH -0.0332456

EEVI -0.27577

EEVK -0.595201

EEVL -0.663123

EEVM 0.22382

EEVN -0.475771

EEVP -0.566821

EEVQ -0.322032

EEVR -0.401474

EEVS -0.606224

EEVT -0.480346

EEVV -0.453212

EEVW 0.426911

EEVY -0.0190737

EEWA 0.0701993

EEWC 0.975536

EEWD 0.139466

EEWE 0.110924

EEWF 0.857653

EEWG -0.160286

EEWH 0.575886

EEWI 0.598982

EEWK 0.150379

EEWL 0.419939

EEWM 0.777221

EEWN 0.320598

EEWP 0.171073

EEWQ 0.428804

EEWR 0.414232

EEWS 0.151857

EEWT 0.232349

EEWV 0.468482

EEWW 1.12519

EEWY 0.948497

EEYA -0.294999

EEYC 0.861661

EEYD -0.0780735

EEYE -0.173202

EEYF 0.438177

EEYG -0.444684

EEYH 0.421091

EEYI 0.125895

EEYK -0.105015

EEYL -0.145711

EEYM 0.52936

EEYN 0.0721872

EEYP -0.0320201

EEYQ 0.177485

EEYR 0.106679

EEYS -0.124148

EEYT -0.0176105

EEYV -0.0444262

EEYW 0.901286

EEYY 0.518268

EFAA -0.766561

EFAC 0.0904524

EFAD -0.670158

EFAE -0.685544

EFAF -0.0182424

EFAG -1.09503

EFAH -0.115051

EFAI -0.381552

EFAK -0.621371

EFAL -0.710354

EFAM 0.0428069

EFAN -0.52244

EFAP -0.777937

EFAQ -0.345563

EFAR -0.447495

EFAS -0.670376

EFAT -0.565828

EFAV -0.604533

EFAW 0.224482

EFAY -0.108882

EFCA 0.149316

EFCC 2.1665
[truncated: 2,096,779 more chars]
